# Photo and copper dual catalysis for allene syntheses from propargylic derivatives via one-electron process

**DOI:** 10.1038/s41467-022-30655-3

**Published:** 2022-06-08

**Authors:** Qi Liu, Jian Zheng, Xue Zhang, Shengming Ma

**Affiliations:** 1 State Key Laboratory of Organometallic Chemistry, Shanghai Institute of Organic Chemistry, Chinese Academy of Sciences, 345 Lingling Lu, Shanghai, 200032 P. R. China; 2 University of Chinese Academy of Sciences, Beijing, 100049 P. R. China; 3 Laboratory of Molecular Recognition and Synthesis, Department of Chemistry, Zhejiang University, Hangzhou, 310027 Zhejiang P. R. China; 4 Research Center for Molecular Recognition and Synthesis, Department of Chemistry, Fudan University, 220 Handan Lu, Shanghai, 200433 P. R. China

**Keywords:** Synthetic chemistry methodology, Catalytic mechanisms

## Abstract

Different from the traditional two-electron oxidative addition-transmetalation-reductive elimination coupling strategy, visible light has been successfully integrated into transition metal-catalyzed coupling reaction of propargylic alcohol derivatives highly selectively forming allenenitriles: specifically speaking, visible light-mediated Cu-catalyzed cyanation of propargylic oxalates has been realized for the general, efficient, and exclusive syntheses of di-, tri, and tetra-substituted allenenitriles bearing various synthetically versatile functional groups. A set of mechanistic studies, including fluorescence quenching experiments, cyclic voltammetric measurements, radical trapping experiments, control experiments with different photocatalyst, and DFT calculation studies have proven that the current reaction proceeds via visible light-induced redox-neutral reductive quenching radical mechanism, which is a completely different approach as compared to the traditional transition metal-catalyzed two-electron oxidative addition processes.

## Abstract (web summary)

Transition-metal-catalyzed couplings of propargylic alcohol derivatives with organometallic reagents proceeds via two-electron transformations, which present limitations in scope and selectivity. Here, the authors present visible-light-mediated copper-catalyzed cyanation of propargylic oxalates to form allenenitriles via a one-electron pathway

## Introduction

**Fig. 1:**
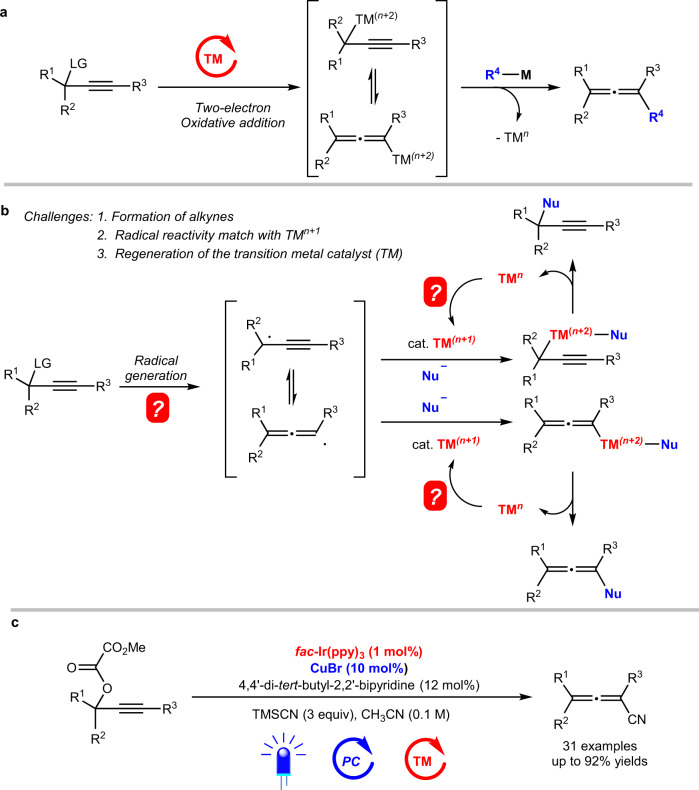
Coupling reactions involving propargylic derivatives. **a** Traditional transition metal-catalyzed two-electron cross-coupling reactions. **b** A concept of one-electron process for cross-coupling reactions. **c** This work: an example of such a concept for allenenitrile synthesis (visible light/transition metal dual catalysis).

Due to the wide existence of allene unit in natural products, bioactive molecules^1^, and functional materials^2^, development of methods for efficient allene syntheses is of high current interest^3–11^. A few strategies such as allenylic substitution with 2-halo-1,3-butadienes^12,13^ or allenyl esters^14–18^, 1,4-difunctionalization of 1,3-enynes^19–32^, allenation of the terminal alkynes (ATA) reaction^33,34^, and coupling reactions involving propargylic substrates^35–53^, have been extensively and well established. For the last reaction, in addition to the S_N_2′-type substitution of propargylic substrates^38–46^, transition metal-catalyzed coupling reaction of propargylic alcohol derivatives with organometallic reagents^47–53^ involves a two-electron oxidative addition-transmetalation-reductive elimination process (Fig. 1a). However, scope and selectivity limitation remain due to the issues of the intrinsic two-electron mechanism^54–56^. Allenenitriles have been frequently employed as useful synthetic precursors for various organic motifs^57–59^, while the classic synthetic method relies on stoichiometric amount of CuCN-mediated cyanation of propargylic alcohols with KCN (1.5 equiv) in the presence of HBr (2.5 equiv)^60^. We envisioned a concept for allenenitrile syntheses via the coupling reaction from propargylic derivatives involving a one-electron process (Fig. 1b). The challenges here (Fig. 1b) are (1) the regioselectivity issue on possible formation of alkyne products^61,62^, (2) the match of radical reactivity with the transition metal species, and (3) the regeneration of the catalytically transition metal catalyst.

In this work, we wish to report such a concept-a radical-based efficient syntheses of allenenitriles from propargylic oxalates and TMSCN under the dual catalysis of photo and copper (Fig. 1c)^60^.

## Results

### Optimization of reaction conditions

We began our study on the coupling reaction of propargylic oxalate **1a** with trimethylsilyl cyanide (TMSCN) under blue light irradiation in the presence of CuBr and photocatalyst, *fac*-Ir(ppy)_3_. The desired allenenitrile **2a** was formed in DMF for 24 h in 29% NMR yield with 18% recovery of **1a** (Table 1, entry 1). After evaluation of a series of 2,2′-dipyridine ligands, we were glad to find that 4,4′-di-*tert*-butyl-2,2′-bipyridine (dtbbpy, **L5**) was the optimal ligand (Table 1, entries 2–6). Notably, no propargylic isomer **3a** was detected in the crude reaction mixture. The reaction performed in CH_3_CN gave higher yield than other checked solvents such as DMAC, NMP, DMPU, THF, and DCM (Table 1, entry 7, for details on solvent screening, see the Supplementary Information). Increasing the loading of TMSCN (Table 1, entry 8) and running the reaction at a concentration of 0.1 M (Table 1, entry 9) further promoted the formation of **2a**, which could be isolated in 89% yield on a 0.5 mmol reaction scale. As expected, CuBr_2_ was totally ineffective (Table 1, entry 10). No reaction occurred in the absence of the light (Table 1, entries 11 and 12) or photocatalyst *fac*-Ir(ppy)_3_ (Table 1, entry 13), suggesting that both the light and photocatalyst were indispensable for the transformation.

**Table 1:** Optimization of the reaction conditions.


Entry	Ligand	Solvent	Yield of 2a^a^	Recovery of 1a^a^
1	–	DMF	29	18
2	**L1**	DMF	7	88
3	**L2**	DMF	28	65
4	**L3**	DMF	52	38
5	**L4**	DMF	53	32
6	**L5**	DMF	61	33
7	**L5**	CH_3_CN	80	14
8^b^	**L5**	CH_3_CN	87	11
9^c^	**L5**	CH_3_CN	94(89^d^)	Trace
10^e^	**L5**	CH_3_CN	0	99
11^c,f^	**L5**	CH_3_CN	0	100
12^c,g^	**L5**	CH_3_CN	0	99
13^c,h^	**L5**	CH_3_CN	0	100

^a^Determined by ^1^H NMR analysis with CH_2_Br_2_ as the internal standard.

^b^3 equivalents of TMSCN were used.

^c^The reaction was conducted on 0.5 mmol scale using TMSCN (3 equiv) in CH_3_CN (5 mL).

^d^Isolated yield.

^e^CuBr_2_ was used instead of CuBr.

^f^Without light.

^g^The reaction was conducted in 50 °C oil bath without light.

^h^Without *fac*-Ir(ppy)_3_.

### Substrate scope

**Fig. 2:**
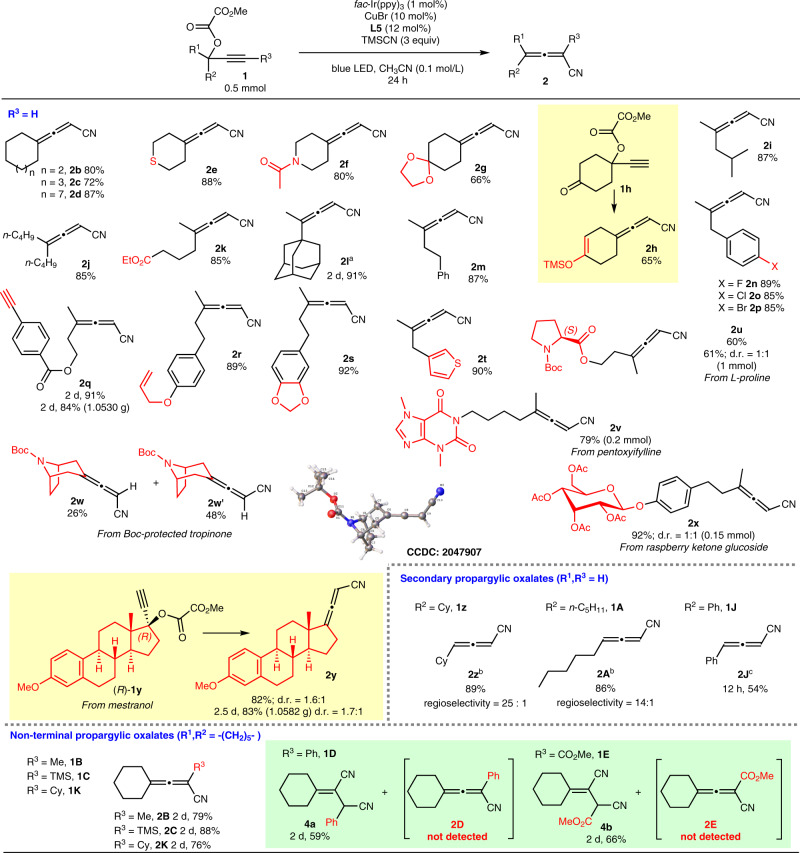
Substrate scope study. ^a^CuBr (15 mol%) and **L5** (18 mol%) were used. ^b^Due to the difficulty of separating the two regioisomers, the yield value refers to the isolated yield of a mixture of alkyne and allene; the regioselectivity was determined by ^1^H NMR analysis. ^c^The reaction was conducted in 10 mL CH_3_CN.

With the optimized reaction conditions in hand, we set out to investigate the substrate scope of this method (Fig. 2). Overall, a variety of terminal tertiary propargylic oxalates smoothly underwent cyanation to form trisubstituted allenenitriles as exclusive regioisomer in good to excellent yields. No obvious yield difference among cyclic (**2a**, **2b**, **2c**, **2d**) and acyclic (**2i**, **2j**, **2m**) substrates was observed. Even with the sterically hindered adamantyl-containing oxalate **1l**, the yield of **2l** was 91% after increasing the catalyst loadings of CuBr and **L5** to 15 mol% and 18 mol%, respectively. A wide range of reactive yet synthetic useful functional groups, such as sulfide (**2e**, easily poisoning Cu catalysis), amide (**2f**), halogen (**2n**, **2o**, **2p**), ester (**2k**, **2q**), ketal (**2g**, **2s**), terminal alkyne (**2q**), and terminal olefin (**2r**) were intact under the standard mild reaction conditions. Interestingly, under the standard conditions the propargylic oxalate **1h** with a ketone unit was converted to nitrile **2h** with the in situ formation of a synthetically useful enol silyl ether entity^63,64^ in 65% yield. The thiophene unit in substrate **1t** was also accommodated. Furthermore, products incorporating Boc-protected L-proline **2u**, pentoxyifylline **2v**, Boc-protected tropinone **2w** and **2w’**, and raspberry ketone tetra-*O*-acetyl-β-D-glucopyranoside **2x**, mestranol **2y** worked well without affecting the other fragile functionalities. The structure of **2w’** was unambiguously established by its X-ray analysis. The reaction could be easily conducted on gram-scales (**2q** and **2y**), demonstrating the practicality of this protocol. Even the reaction of terminal secondary propargylic oxalates **1z** and **1A** still afforded 1,3-disubstituted allenenitriles **2z** and **2A** as the products in decent yields and a very high allene/alkyne selectivity (25:1 and 14:1). 4-Phenylallenenitrile **2J** could also be obtained via the current method in 54% yield as the only isomer, and the slightlylower isolated yield may be attributed to its instability.

The reaction could be further extended to non-terminal propargylic oxalates, such as **1B**, **1C**, and **1K**. When trimethylsilyl-substituted alkyne **1C** was used, TMS-substituted allenenitrile **2C** was produced exclusively in 88% yield, which was not readily accessible by other ways^65^ and very useful in propargylation reaction^66,67^. For non-terminal propargylic oxalates with R^3^ being Ph (**1D**) and CO_2_Me (**1E**), dinitrile products **4a** and **4b** were obtained, which must be produced from the subsequent conjugate addition of TMSCN with the in situ formed allenenitrile intermediate **2D** and **2E**, respectively.

**Fig. 3:**
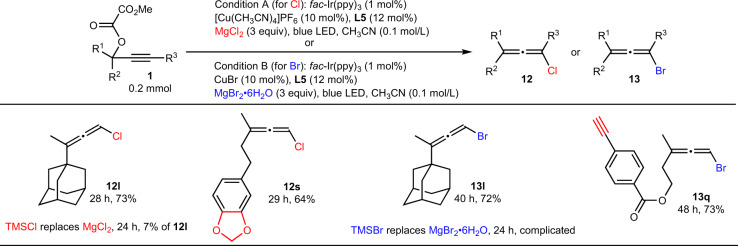
Reaction with MgCl_2_ or MgBr_2_•6H_2_O instead of TMSCN. The reaction condition A was used for the synthesize of cholorallenes (present in red color), and the reaction condition B was used for the synthesize of bromoallenes (present in blue color).

Interestingly, when MgCl_2_ or MgBr_2_•6H_2_O replaced TMSCN as the nucleophile, various chloroallene or bromoallene bearing sterically hindered adamantyl (**12l** or **13l**), ketal (**12s**), ester, or terminal alkyne (**13q**) could be obtained in decent yields. As a comparison, TMSBr or TMSCl gave inferior results (Fig. 3).

### Synthetic applications

**Fig. 4:**
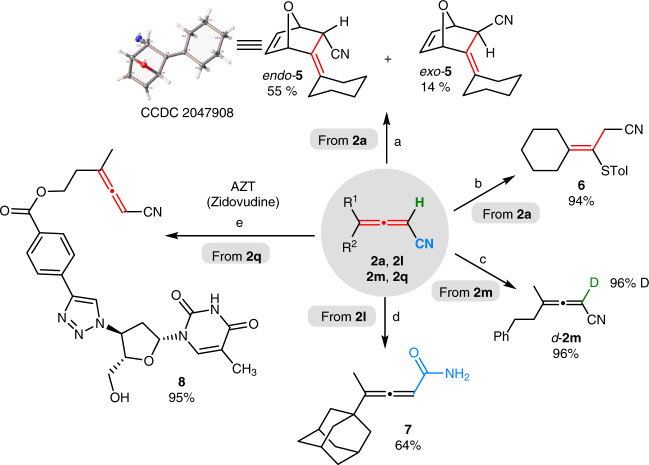
Synthetic transformations of allenenitriles. Reagents and conditions: (a) **2a** (0.2 mmol), Cu(CH_3_CN)_4_BF_4_ (20 mol%), freshly distilled furan (2 mL), 50 °C, 2 d; (b) **2a** (0.4 mmol), 4-methylbenzenethiol (1.2 equiv), Et_3_N (2.0 equiv), CHCl_3_, rt, 24 h; (c) **2** **m** (0.27 mmol), K_2_CO_3_ (5.0 equiv), *n*-Bu_4_NBr (1.0 equiv), Toluene/D_2_O = 9:11, rt, 2.5 d; (d) **2** **l** (0.2 mmol), NaOH (20 mol%), Na_2_CO_3_ (1.0 equiv), H_2_O_2_ (3.9 equiv), EtOH/H_2_O = 5:1, rt, 24 h; (e) **2q** (0.4 mmol), AZT (1.0 equiv), CuSO_4_·5H_2_O (5 mol%), sodium ascorbate (15 mol%), DCM/H_2_O = 1:1.

These allenenitriles are synthetic versatile as shown in Fig. 4: The Cu(I) catalyzed [4 + 2] cycloaddition^68^ of **2a** (R^1^, R^2^ = -(CH_2_)_5_-) with furan provided 7-oxa-bicyclo-[2.2.1]heptene derivatives *endo*-**5** and *exo*-**5** in 55 and 14% yield, respectively. The configuration of *endo*-**5** was unambiguously identified by X-ray analysis. Conjugate addition of 4-methylbenzenethiol with **2a** afforded sulfur-substituted tetrasubstituted alkene **6** in an excellent yield^69^. Deuteration of α-H of **2m** (R^1^ = Me, R^2^ = -(CH_2_)_2_Ph) with D_2_O in the presence of K_2_CO_3_ and *n*-Bu_4_NBr readily yielded *d*-**2m** in 96% yield with 96% D-incorporation. Hydrolysis of nitrile group in **2l** (R^1^ = Me, R^2^ = 1-adamantyl) with a base produced allenyl amide **7** in 64% yield^70^. In addition, the ethynyl group in **2q** underwent the Cu-catalyzed click reaction with anti-HIV drug AZT (Zidovudine)^71^ while the allenenitrile unit remained unreacted, offering useful handle for further synthetic elaboration.

### Mechanistic studies

**Fig. 5:**
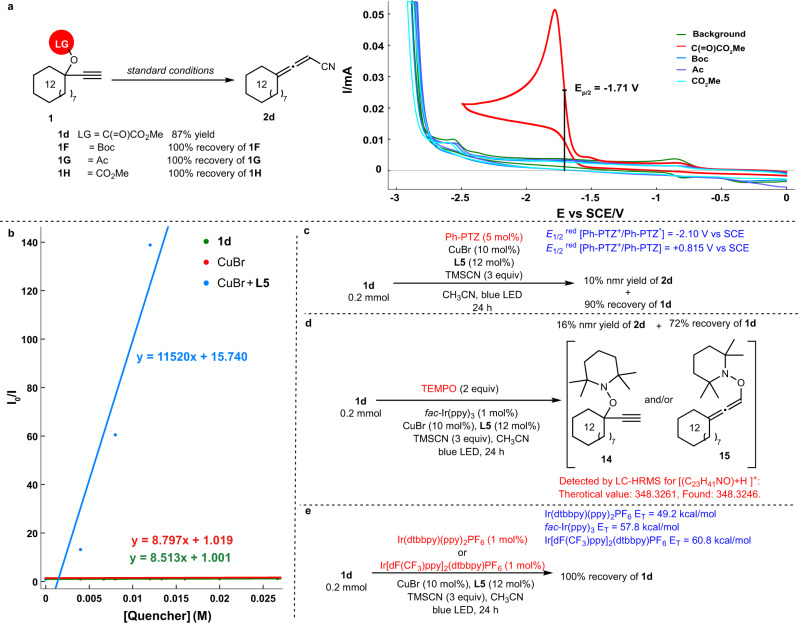
Mechanistic studies. **a** Experiments and cyclic voltammograms of different propargylic compounds. **b** Stern-Volmer quenching experiments of *fac*-Ir(ppy)_3_. **c** Reaction with Ph-PTZ photocatalyst. **d** The radical trapping experiment with TEMPO. **e** Reaction with Ir(dtbbpy)(ppy)_2_PF_6_ or Ir[dF(CF_3_)ppy]_2_(dtbbpy)PF_6_ as photocatalyst.

To probe the reaction mechanism, we conducted a set of mechanistic studies. First, several propargylic compounds with different leaving groups **1F** (Boc), **1G** (Ac), **1H** (CO_2_Me) were prepared. The Cyclic Voltammetry (CV) experiments were performed to measure the reduction potential of these substrates **1d**, **1F**, **1G**, and **1H** (Fig. 5a). The half peak potential of redox active oxalate **1d** was determined to be *E*_p/2_ [**1d**/**1d**^•-^] = −1.71 V vs SCE (Saturated calomel electrode) in CH_3_CN. However, under the same measurement conditions for **1F** (Boc), **1G** (Ac), and **1H** (CO_2_Me), no apparent anodic and cathodic current peaks could be observed in the range of −3.0 to 0 V, suggesting that these were redox-inactive leaving groups. Indeed, when **1F** (Boc), **1G** (Ac), or **1H** (CO_2_Me) were subjected to the optimal conditions, 100% of the corresponding unreacted starting materials were recovered.

**Fig. 6:**
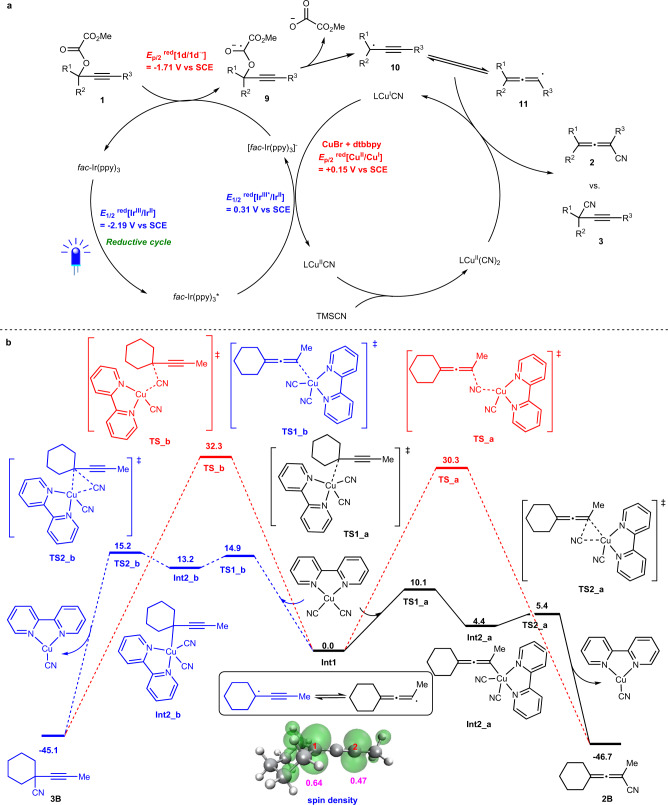
Possible mechanism. **a** Proposed mechanism via reductive quenching cycle. **b** Free energy profiles calculated for the reaction of **L4**Cu^II^(CN)_2_ with **Int1**. Relative free energies are given in kcal/mol.

Two possible reaction pathways for this transformation based on CV data were proposed as shown in Fig. 6a and Supplementary Fig. 5. In oxidative quenching cycle (Supplementary Fig. 5), first, the excited state of *fac*-Ir(ppy)_3_* (*E*_1/2_^red^ [Ir^IV^/Ir^III*^] = −1.73 V vs SCE in CH_3_CN)^72^ could be quenched with oxalate **1** (*E*_p/2_ [**1d**/**1d**^•-^] = −1.71 V vs SCE in CH_3_CN) to generate [*fac*-Ir(ppy)_3_]^+^ species and anionic radical intermediate **9**, which would form propargylic radical **10** by releasing oxalate anion. Then LCu^I^CN (*E*_p/2_^red^ [Cu^II^/Cu^I^] = +0.15 V vs SCE in CH_3_CN, see Supplementary Information for details) would be oxidized by [*fac*-Ir(ppy)_3_]^+^ (*E*_1/2_^red^ [Ir^IV^/Ir^III^] = +0.77 V vs SCE in CH_3_CN)^72^ to produce LCu^II^CN, which would further react with TMSCN to yield LCu^II^(CN)_2_. Alternatively, in reductive quenching cycle (Fig. 6a), the excited state of *fac*-Ir(ppy)_3_* (*E*_1/2_^red^ [Ir^III*^/Ir^II^] = +0.31 V vs SCE in CH_3_CN)^72^ could be quenched with LCu^I^CN to generate LCu^II^(CN) and [*fac*-Ir(ppy)_3_]^−^ species (*E*_1/2_^red^ [Ir^III^/Ir^II^] = −2.19 V vs SCE in CH_3_CN)^72^. Oxalate **1** could be reduced by [*fac*-Ir(ppy)_3_]^−^ to yield anionic radial intermediate **9** via one-electron reduction. Finally, in both pathway the radical intermediate **10** may isomerize to allenyl radical **11**^73,74^, which may bind with LCu^II^(CN)_2_, followed by reductive elimination to deliver allenenitrile **2** and regenerate the catalytically active species LCu^I^CN. Another possible pathway, **11** could abstract the CN group from LCu^II^(CN)_2_ to afford allenenitrile **2**^32,75,76^. The steric effect of R^1^, R^2^, and R^3^ may play an important role in the reaction selectivity for forming **2** or **3**.

To distinguish the two pathways, Stern-Volmer quenching experiments of *fac*-Ir(ppy)_3_ were carried out. As shown in Fig. 5b, the excited state of the photocatalyst *fac*-Ir(ppy)_3_ was efficiently quenched by the CuBr/**L5** catalyst. Furthermore, if the cyanation of **1d** would be realized via oxidative quenching cycle, considering the redox-potential window of typical photocatalysis (Ir/Ru/organic-PC etc.)^77^, the Ph-PTZ was selected as another potential photocatalyst for this transformation. The reaction in the presence of photocatalyst Ph-PTZ instead of *fac*-Ir(ppy)_3_ would provide readily radical **10** or **11**, the subsequent SET process between the oxidized state of Ph-PTZ^+^ (*E*_1/2_[Ph-PTZ^+^/Ph-PTZ] = +0.815 V vs SCE in CH_3_CN)^61^ and LCu^I^CN (*E*_p/2_^red^ [Cu^II^/Cu^I^] = +0.15 V vs SCE in CH_3_CN) would form LCu^II^CN, which could yield **2d**. However, such a reaction only afforded 10% of **2d** with 90% of **1d** being recovered (Fig. 5c). When 2 equiv of TEMPO were used as the radical trapping agent in the reaction of **1d**, the formation of **2d** was obviously reduced (16% vs 87%), and the TEMPO-trapped product **14** and/or **15** could be detected by LC-HRMS analysis, which supports the involvement of radical intermediates in the current transformation (Fig. 5d). Furthermore, in order to check the possible triplet energy transfer mechanism, other ruthenium- or iridium-based dyes or organic photocatalysts were tested under standard conditions (for details on photocatalyst screening, see the Supplementary Information): Photocatalysts (Ir(dtbbpy)(ppy)_2_PF_6_, *E*_T_ = 49.2 kcal/mol and Ir[dF(CF_3_)ppy]_2_(dtbbpy)PF_6_, *E*_T_ = 60.8 kcal/mol) with its triplet energy similar to that of *fac*-Ir(ppy)_3_ (*E*_T_ = 57.8 kcal/mol) did not provide **2d** at all (Fig. 5e)^78,79^.

To further elucidate the reaction mechanism, density functional theory (DFT) calculations were preformed to survey the reaction of **1B** using ligand **L4** (For details on DFT calculations, see the Supplementary Information and Supplementary Data 1). As proposed by Fig. 6a, radical intermediate **Int1** could be formed from oxalate **1B**. Mulliken atomic spin density analysis of **Int1** suggests that the single electron distributes on C^1^ and C^2^ with a similar spin density (0.64 and 0.47, Fig. 6b), indicating **Int1** is a combination of resonance forms of allenyl radical and propargylic radical. As an allenyl radical, **Int1** reacts with **L4**Cu^II^(CN)_2_ via a singlet diradical transition structure **TS1_a** with a free energy barrier of 10.1 kcal/mol, providing a closed-shell propargyl-Cu(III) complex **Int2_a** reversibly. Subsequent reductive elimination produces the final allenenitrile product **2B** with a very low barrier of 1.0 kcal/mol (**TS2_a**). Furthermore, the concerted radical cyanation process is also investigated. A triplet transition structure **TS_a** was obtained with a much higher free energy barrier of 30.3 kcal/mol, which indicates that the stepwise pathway via a Cu(III) intermediate is more favorable. On the other hand, the possibility of **Int1** acting as a propargyl radical has also been considered. A similar oxidation/reductive elimination process is obtained, but more energy demanding, due to the steric effect caused by the cyclohexyl group with the ligand. Thus, allenenitriles **2B** were generated as the only products.

These above results definitely confirmed that the reductive quenching cycle in Fig. 6a was the dominant pathway in the current transformation, which is different from the well-established oxidative quenching mechanism^61,75,76^.

In conclusion, we have developed a general and efficient method for the highly selective synthesis of di-, tri-, and tetra-substituted allenenitriles from readily available propargylic oxalates and TMSCN under photoredox conditions. This reaction featured mild conditions and a broad functional group compatibility. Excellent regioselectivities were achieved in both terminal and internal propargylic oxalates. Even for secondary substrates, allenenitriles were still the predominant products. The current method was further extended to the synthesis of cholorallenes or bromoallenes by using MgCl_2_ or MgBr_2_•6H_2_O as the nucleophile. Stern-Volmer quenching experiments, cyclic voltammetric measurements, radical trapping experiments, control experiments with different photocatalysts, and DFT calculation studies indicated that propargylic radical and allenyl radical generated via light-induced one-electron process were involved via the reductive quenching cycle. This protocol for allenenitrile syntheses involving one-electron mechanistic pathway is very different from the traditional transition metal-catalyzed two-electron coupling reactions and will surely overcome the scope limitation of the known protocols and enjoy scopes for the efficient syntheses of differently functionalized allenes due to the powerful catalytic activity of copper^80,81^. Further studies on highly selective allene synthesis via such one-electron process and other photocatalysts are being actively pursued in this laboratory.

## Methods

### General procedure for the copper-catalyzed cyanation of propargylic oxalates

To a flame-dried 10 mL Schlenk tube were added *fac*-Ir(ppy)_3_ (3.3 mg, 5 μmol), CuBr (7.3 mg, 0.05 mmol), 4,4′-di-*tert*-butyl-2,2′-bipyridine **L5** (16.4 mg, 0.06 mmol), **1a** (105.4 mg, 0.5 mmol)/CH_3_CN(2.5 mL), and TMSCN (157.2 mg, 1.5 mmol)/CH_3_CN(2.5 mL) sequentially under Ar atmosphere. The resulting mixture was irradiated with a 50 W blue LED lamp (2-3 cm away, with cooling fan to keep the reaction temperature at 35–40 °C) for 24 h with stirring and monitored by TLC. The resulting mixture was filtrated through a short pad of silica gel eluted with ethyl ether (30 mL). After evaporation, the residue was purified by chromatography on silica gel to afford the pure product **2a**.

## Supplementary information

Supplementary_Information

Description of Additional Supplementary Files

Supplementary Dataset 1

## Data Availability

The X-ray crystallographic coordinates for structures of **2w’** and *endo*-**5** reported in this study have been deposited in the Cambridge Crystallographic Data Centre (CCDC) under deposition numbers CCDC 2047907 (**2w’**), and CCDC-2047908 (*endo*-**5**). These data can be obtained free of charge from http://www.ccdc.cam.ac.uk/data_request/cif. The experimental procedures and characterization of the new compounds in this study are provided in the Supplementary Information. All other data are available from the authors upon request.
