# Supplementary material for: Photo and copper dual catalysis for allene syntheses from propargylic derivatives via one-electron process
Source: Nat Commun. 2022 Jun 8;13:3302. doi: 10.1038/s41467-022-30655-3 (PMC9177964; doi:10.1038/s41467-022-30655-3)
Supplement: Supplementary file 1 — Supplementary_Information [file 41467_2022_30655_MOESM1_ESM.pdf]

Supplementary Information: Photo and Copper Dual Catalysis for Allene Syntheses from Propargylic Derivatives via One-Electron Process

S. Ma et al.

Table of Contents

Supplementary Notes	2
Supplementary Methods	2-224
Supplementary References	225-228

Supplementary Notes

All reactions were carried out under Ar in dried Schlenk tubes or flasks. CuBr (98%) was purchased from Shanghai Aladdin Biochemical Technology Co., Ltd; *fac*-Ir(ppy)$_3$ (99%) was purchased from Sinocompound Catalysts Co., Ltd; 4,4'-di-*tert*-butyl-2,2'-bipyridine (98%) was purchased from Meryer Chemical Technology Co., Ltd; DMAP (99%) and methyl oxalyl chloride (98%) were purchased from Shanghai Adamas Reagent Co., Ltd; Et_3N (99%) was purchased from Sinopharm Chemical Reagent Co., Ltd. Acetonitrile and dichloromethane were dried over CaH_2 and distilled freshly before use. Other reagents were used without further treatment. Petroleum ether (60 ~ 90 °C) was used for chromatography. $CFCl_3$ was used as the internal standard for the ^{19}F NMR analysis.

Supplementary Methods

Supplementary table for solvent screening

Supplementary Table 1. Details for solvent screening

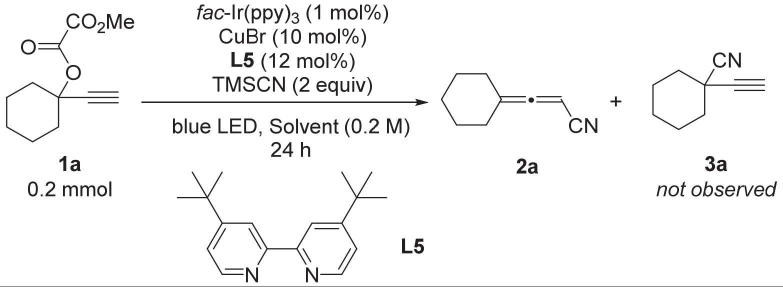

Entry	Solvent	Yield of **2a**[a]	Recovery of **1a**[a]
1	DMAC	72	27
2	NMP	56	35
3	DMPU	43	28
4	THF	22	79
5	DCM	30	68

[a] Determined by ^1H NMR analysis with CH_2Br_2 as the internal standard.

Synthesis of propargylic alcohols

Propargylic alcohols **S-1a**, **S-1b**, **S-1c**, **S-1i**, **S-1y**, and **S-1B** were commercial available and used as received without further treatment. Propargylic alcohols **S-1d**,[1] **S-1e**,[2] **S-1f**,[3] **S-1g**,[4] **S-1h**,[4] **S-1j**,[5] **S-1k**,[6] **S-1m**,[1] **S-1w**,[7] **S-1z**,[8] **S-1A**,[8] **S-1C**,[2] **S-1D**,[9] and **S-1E**[10] were prepared according to literature methods.

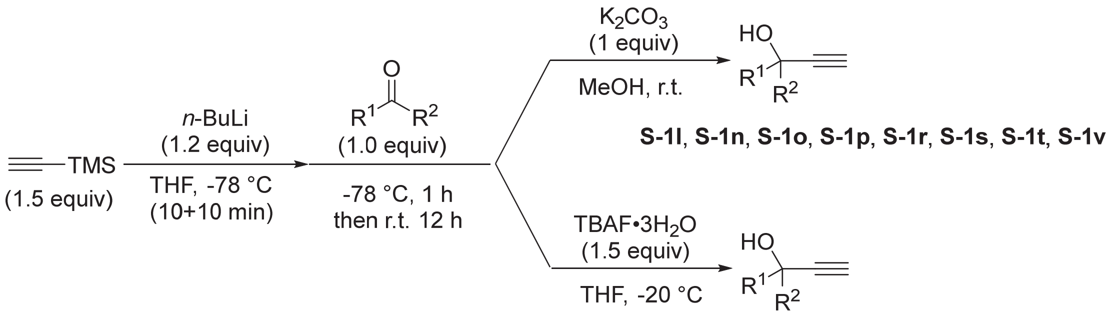

General Procedure: To a flame-dried round-bottomed flask were added trimethylsilylacetylene (1.5 equiv) and THF (0.6 mol/L). Then a solution of *n*-BuLi (1.2 equiv) was added dropwise within 10 min at -78 ºC under Ar atmosphere. After being stirred at -78 ºC for another 10 min, a solution of ketone (1.0 equiv) in THF (2.0 mol/L) was added dropwise. The resulting mixture was stirred at -78 ºC for 1 h, naturally warmed up to room temperature, and stirred for 12 h as monitored by TLC. After being quenched by a saturated aqueous solution of NH_4Cl (10 mL) and extracted with ethyl acetate (3 x 10 mL), the combined organic layer was dried over anhydrous Na_2SO_4. After filtration and evaporation, the residue was purified by chromatography on silica gel to afford the crude product.

For **S-1l**, **S-1n**, **S-1o**, **S-1p**, **S-1r**, **S-1s**, **S-1t**, and **S-1v**: To a flask were added the crude product obtained above, K_2CO_3 (1 equiv), and MeOH (0.33 mol/L). The resulting mixture was stirred for 24 h at room temperature as monitored by TLC and concentrated under reduced pressure directly. Then H_2O (10 mL) was added, the resulting solution was extracted with CH_2Cl_2 (3 x 10 mL). The combined organic layer was dried over anhydrous Na_2SO_4, filtrated, and concentrated under reduced pressure. The residue was purified by chromatography on silica gel to afford the desired propargylic alcohol **S-1**, which was used in the next step without further characterization.

For **S-1x**: The crude product obtained above was dissolved in THF (0.2 mol/L) and treated with TBAF•3H_2O (1.5 equiv) at -20 ºC. After 12 h, the reaction was complete as monitored by TLC, H_2O (10 mL) was added. The organic layer was separated, and the aqueous layer was extracted with ethyl acetate (3 x 10 mL). The combined organic layer was dried over anhydrous Na_2SO_4. After filtration and evaporation, the residue was purified by chromatography on silica gel to afford the desired propargylic alcohol **S-1x**, which was used in the next step without further characterization.

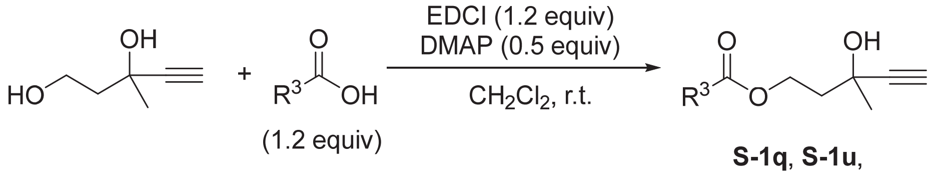

S-1q, **S-1u**,

For **S-1q** and **S-1u**: To an oven-dried round-bottomed flask were added acid (1.2 equiv), *N*-(3-dimethylaminopropyl)-*N*'-ethylcarbodiimide hydrochloride (EDCI, 1.2 equiv), 4-dimethylaminopyridine (DMAP, 0.5 equiv), 3-methylpent-4-yne-1,3-diol (1 equiv), and DCM (0.2 mol/L) sequentially under Ar atmosphere. The resulting mixture was stirred at room temperature for 17 h as monitored by TLC, and then washed sequentially with a saturated solution of NH_4Cl (45 mL), H_2O (45 mL), and brine (45 mL). The organic layer was dried over anhydrous Na_2SO_4. After filtration and evaporation, the residue was purified by chromatography on silica gel to afford the desired propargylic alcohol **S-1q** or **S-1u**, which was used in the next step without further characterization.

Synthesis of propargylic oxalates

(1) 1-Ethynylcyclooctyl methyl oxalate **1c** (lq-6-137)

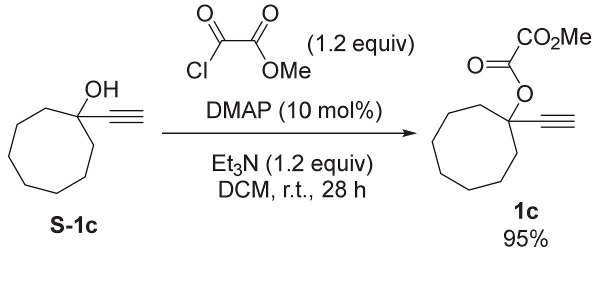

S-1c

1c
95%

Typical Procedure I: To a round-bottomed flask were added alkynol **S-1c** (528.0 mg, 3.47 mmol), DCM (10.4 mL), 4-dimethylaminopyridine (DMAP, 44.6 mg, 0.361 mmol), and Et_3N (0.59 mL, d = 0.726 g/mL, 428.3 mg, 4.19 mmol) sequentially. Then methyl oxalyl chloride (0.39 mL, d = 1.332 g/mL, 519.5 mg, 4.16 mmol) was added dropwise. The resulting mixture was stirred at room temperature for 28 h as monitored by TLC, then approximately half of the solvent was evaporated through rotary evaporation followed by the addition of ethyl ether (25 mL). The resulting mixture was filtered and eluted with ethyl ether (25 mL). The filtrate was concentrated under reduced pressure and the residue was purified by chromatography on silica gel (eluent: petroleum ether/ethyl acetate = 20:1) to afford **1c** (781.1 mg, 95%) as a liquid: **^{1}H NMR** (400 MHz, $CDCl_3$) δ 3.88 (s, 3 H, OCH_3), 2.68 (s, 1 H, C≡CH), 2.39-2.27 (m, 2 H), 2.26-2.14 (m, 2 H), 1.77-1.41 (m, 10 H); **^{13}C NMR** (100 MHz, $CDCl_3$) δ 158.4, 155.6, 82.5, 82.4, 75.4, 53.3, 34.6, 27.6, 24.4, 21.5; **MS** (ESI) *m/z* 261 ($[M+Na]^+$), 277 ($[M+K]^+$); **IR** (neat): ν = 3278, 2925, 2855, 1770, 1743, 1475, 1446, 1317, 1197, 1153, 1062, 1011 cm^{-1}; **HRMS** (ESI) calcd for $C_{13}H_{18}O_4Na$ ($[M+Na]^+$): 261.1097, Found: 261.1096.

(2) 1-Ethynylcyclododecyl methyl oxalate **1d** (lq-6-145)

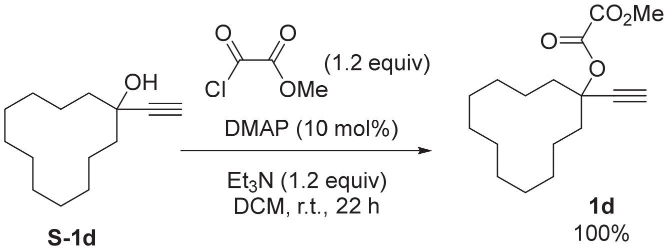

Following **Typical Procedure I**, the reaction of alkynol **S-1d** (757.1 mg, 3.63 mmol)/DCM (10.9 mL), methyl oxalyl chloride (0.41 mL, d = 1.332 g/mL, 546.1 mg, 4.37 mmol), DMAP (44.8 mg, 0.363 mmol), and NEt_3 (0.61 mL, d = 0.726 g/mL, 442.9 mg, 4.33 mmol) afforded **1d** (1.0806 g, 100%) (eluent: petroleum ether/ethyl ether = 20:1) as a white solid (m.p. 82.4-83.5 °C, *n*-hexane): **^{1}H NMR** (400 MHz, $CDCl_3$) δ 3.88 (s, 3 H, OCH_3), 2.66 (s, 1 H, C≡CH), 2.30-2.18 (m, 2 H), 1.98-1.86 (m, 2 H), 1.66-1.52 (m, 2 H), 1.45-1.25 (m, 16 H); **^{13}C NMR** (100 MHz, $CDCl_3$) δ 158.3, 155.5, 82.0,

81.5, 75.5, 53.4, 32.6, 25.9, 25.6, 22.2, 21.9, 19.1; **MS** (ESI) *m/z* 317 ([M+Na]$^+$), 333 ([M+K]$^+$); **IR** (neat): ν = 3267, 2960, 2934, 2853, 1760, 1738, 1469, 1450, 1440, 1334, 1206, 1174, 1152, 1053 cm^{-1}; Anal. Calcd. for $C_{17}H_{26}O_4$: C 69.36, H 8.90; Found: C 69.67, H 8.91.

(3) 4-Ethynyltetrahydro-2*H*-thiopyran-4-yl methyl oxalate **1e** (lq-7-091)

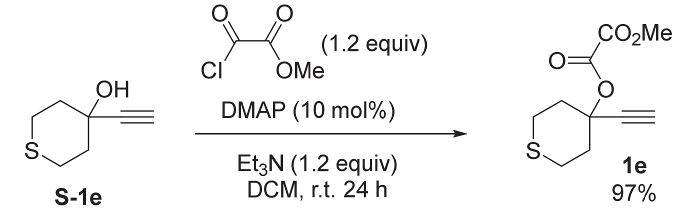

Following **Typical Procedure I**, the reaction of alkynol **S-1e** (674.4 mg, 4.74 mmol)/DCM (14.2 mL), methyl oxalyl chloride (0.53 mL, d = 1.332 g/mL, 706.0 mg, 5.65 mmol), DMAP (58.7 mg, 0.476 mmol), and NEt_3 (0.8 mL, d = 0.726 g/mL, 580.8 mg, 5.68 mmol) afforded **1e** (1.0533 g, 97%) (eluent: petroleum ether/ethyl acetate = 10:1) as a liquid: **^{1}H NMR** (400 MHz, $CDCl_3$) δ 3.91 (s, 3 H, OCH_3), 2.93-2.83 (m, 2 H, 2 × one proton of SCH_2), 2.81 (s, 1 H, C≡CH), 2.78-2.67 (m, 2 H, 2 × one proton of SCH_2), 2.57-2.46 (m, 2 H, 2 × one proton of CH_2), 2.35-2.24 (m, 2 H, 2 × one proton of CH_2); **^{13}C NMR** (100 MHz, $CDCl_3$) δ 157.9, 155.2, 80.4, 77.8, 77.6, 53.5, 37.4, 24.9; **MS** (ESI) *m/z* 246 ([M+NH_4]$^+$), 251 ([M+Na]$^+$); **IR** (neat): ν = 3214, 2910, 2108, 1774, 1759, 1737, 1634, 1439, 1351, 1316, 1276, 1261, 1223, 1198, 1160, 1117, 1078, 1019 cm^{-1}; **HRMS** (ESI) calcd for $C_{10}H_{12}O_4NaS$ ([M+Na]$^+$): 251.0349, Found: 251.0347.

(4) 1-Acetyl-4-ethynylpiperidin-4-yl methyl oxalate **1f** (lq-6-150)

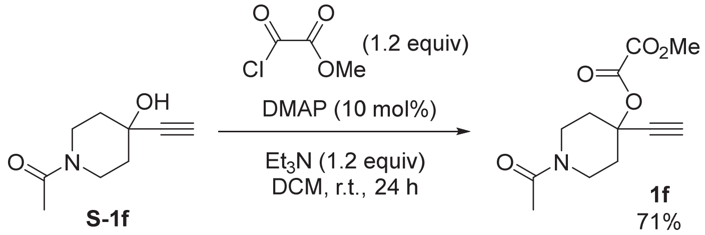

Following **Typical Procedure I**, the reaction of alkynol **S-1f** (553.8 mg, 3.3 mmol)/DCM (10 mL), methyl oxalyl chloride (0.37 mL, d = 1.332 g/mL, 492.8 mg, 3.94 mmol), DMAP (40.9 mg, 0.33 mmol), and NEt_3 (0.56 mL, d = 0.726 g/mL, 406.6

mg, 3.98 mmol) afforded **1f** (596.5 mg, 71%) (eluent: petroleum ether/ethyl acetate = 1:1 (500 mL) to dichloromethane/MeOH = 20:1 (~525 mL)) as a liquid: **^{1}H NMR** (400 MHz, $CDCl_3$) δ 4.13-4.00 (m, 1 H, one proton of NCH_2), 3.91 (s, 3 H, OCH_3), 3.74-3.63 (m, 1 H, one proton of NCH_2), 3.57-3.38 (m, 2 H, two proton of NCH_2), 2.79 (s, 1 H, C≡CH), 2.40-2.21 (m, 2 H, two protons of 2 × CH_2), 2.18-2.00 (m, 2 H, two protons of 2 × CH_2), 2.12 (s, 3 H, $COCH_3$); **^{13}C NMR** (100 MHz, $CDCl_3$) δ 168.8, 157.6, 155.3, 79.7, 77.3, 76.2, 53.6, 42.6, 37.7, 36.3, 35.7, 21.2; **MS** (ESI) *m/z* 254 ($[M+H]^+$), 276 ($[M+Na]^+$); **IR** (neat): ν = 3187, 2876, 2113, 1767, 1741, 1624, 1474, 1432, 1373, 1315, 1275, 1249, 1226, 1193, 1158, 1136, 1089, 1055, 1025 cm^{-1}; **HRMS** (ESI) calcd for $C_{12}H_{15}NO_5Na$ ($[M+Na]^+$): 276.08424, Found: 276.08401.

(5) 8-Ethynyl-1,4-dioxaspiro[4.5]decan-8-yl methyl oxalate **1g** (lq-7-008)

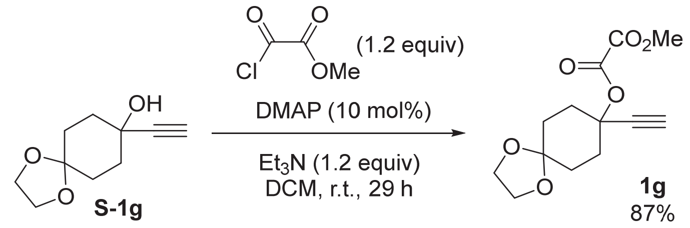

Following **Typical Procedure I**, the reaction of alkynol **S-1g** (298.7 mg, 1.64 mmol)/DCM (4.9 mL), methyl oxalyl chloride (0.18 mL, d = 1.332 g/mL, 239.8 mg, 1.918 mmol), DMAP (20.3 mg, 0.165 mmol), and NEt_3 (0.28 mL, d = 0.726 g/mL, 203.3 mg, 1.989 mmol) afforded **1g** (383.1 mg, 87%) (eluent: petroleum ether/ethyl ether = 5:1 (360 mL) to 3:1 (400 mL)) as a liquid: **^{1}H NMR** (400 MHz, $CDCl_3$) δ 3.96 (s, 4 H, 2 × CH_2), 3.89 (s, 3 H, OCH_3), 2.70 (s, 1 H, C≡CH), 2.29 (t, J = 6.2 Hz, 4 H, 2 × CH_2), 1.90-1.73 (m, 4 H, 2 × CH_2); **^{13}C NMR** (100 MHz, $CDCl_3$) δ 158.0, 155.6, 107.1, 80.9, 77.2, 75.9, 64.4, 64.3, 53.5, 33.9, 30.8; **MS** (ESI) *m/z* 286 ($[M+NH_4]^+$), 291 ($[M+Na]^+$); **IR** (neat): ν = 3276, 2959, 2889, 2117, 1769, 1745, 1656, 1440, 1375, 1319, 1272, 1201, 1153, 1102, 1033 cm^{-1}; **HRMS** (ESI) calcd for $C_{13}H_{16}O_6Na$ ($[M+Na]^+$): 291.0839, Found: 291.0838.

(6) 1-Ethynyl-4-oxocyclohexyl methyl oxalate **1h** (lq-7-010)

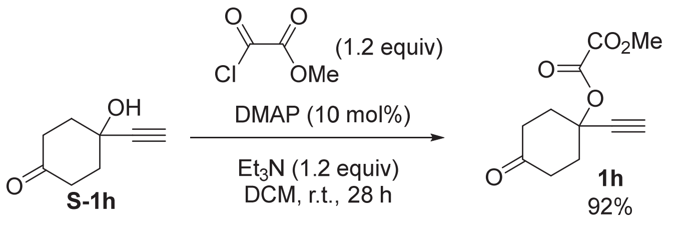

Following **Typical Procedure I**, the reaction of alkynol **S-1h** (350.4 mg, 2.54 mmol)/DCM (7.6 mL), methyl oxalyl chloride (0.29 mL, d = 1.332 g/mL, 386.3 mg, 3.09 mmol), DMAP (31.5 mg, 0.255 mmol), and NEt_3 (0.43 mL, d = 0.726 g/mL, 312.2 mg, 3.05 mmol) afforded **1h** (521.2 mg, 92%) (eluent: petroleum ether/ethyl acetate = 5:1 (360 mL) to 3:1 (400 mL)) as a light yellow solid (m.p. 102.1-103.2 ^{o}C, *n*-hexane): **^{1}H NMR** (400 MHz, $CDCl_3$) δ 3.92 (s, 3 H, OCH_3), 2.80 (s, 1 H, C≡CH), 2.66-2.40 (m, 8 H, 4 × CH_2); **^{13}C NMR** (100 MHz, $CDCl_3$) δ 207.4, 157.7, 155.4, 79.7, 77.0, 75.7, 53.6, 36.7, 35.6; **MS** (ESI) *m/z* 242 ($[M+NH_4]^+$), 247 ($[M+Na]^+$); **IR** (neat): ν = 3255, 2952, 2119, 1766, 1748, 1713, 1697, 1455, 1440, 1410, 1379, 1346, 1312, 1261, 1232, 1203, 1152, 1118, 1086, 1022 cm^{-1}; Anal. Calcd. for $C_{11}H_{12}O_5$: C 58.93, H 5.39; Found: C 58.88, H 5.55.

(7) 5-Ethynylnonan-5-yl methyl oxalate **1j** (lq-7-197)

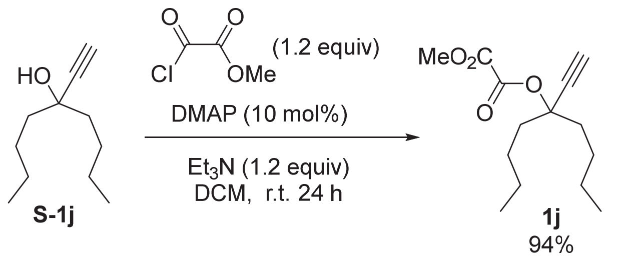

Following **Typical Procedure I**, the reaction of alkynol **S-1j** (2.8970 g, 17.2 mmol)/DCM (52 mL), methyl oxalyl chloride (1.94 mL, d = 1.332 g/mL, 2.5841 g, 20.67 mmol), DMAP (213.1 mg, 1.73 mmol), and NEt_3 (2.9 mL, d = 0.726 g/mL, 2.1054 g, 20.6 mmol) afforded **1j** (4.1304 g, 94%) (eluent: petroleum ether/ethyl ether = 20:1) as a liquid: **^{1}H NMR** (400 MHz, $CDCl_3$) δ 3.89 (s, 3 H, OCH_3), 2.66 (s, 1 H, C≡CH), 2.13-2.03 (m, 2 H), 2.01-1.91 (m, 2 H), 1.54-1.28 (m, 8 H), 0.93 (t, J = 7.0 Hz, 6 H, 2 × CH_3); **^{13}C NMR** (100 MHz, $CDCl_3$) δ 158.3, 155.7, 82.4, 81.5, 75.7, 53.4, 37.7, 25.9, 22.5, 13.8; **MS** (ESI) *m/z* 277 ($[M+Na]^+$); **IR** (neat): ν = 3280, 2958, 2934,

2873, 2119, 1770, 1745, 1459, 1438, 1381, 1319, 1199, 1162, 1122 cm^{-1}; **HRMS** (ESI) calcd for $C_{14}H_{22}O_4Na$ ($[M+Na]^+$): 277.1410, Found: 277.1404.

(8) 7-Ethoxy-3-methyl-7-oxohept-1-yn-3-yl methyl oxalate **1k** (lq-7-059)

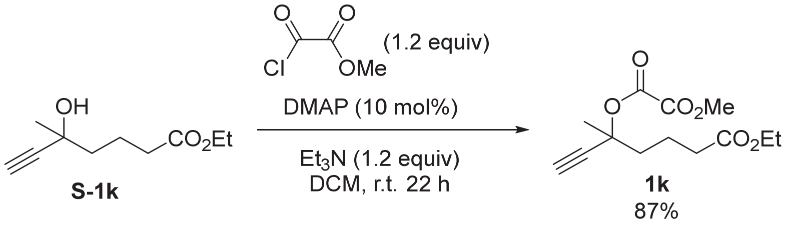

Following **Typical Procedure I**, the reaction of alkynol **S-1k** (1.9104 g, 10.37 mmol)/DCM (31 mL), methyl oxalyl chloride (1.2 mL, d = 1.332 g/mL, 1.5984 g, 12.79 mmol), DMAP (128.3 mg, 1.04 mmol), and NEt_3 (1.8 mL, d = 0.726 g/mL, 1.3068 mg, 12.79 mmol) afforded **1k** (2.4247 g, 87%) (eluent: petroleum ether/ethyl acetate = 5:1) as a liquid: **^{1}H NMR** (400 MHz, $CDCl_3$) δ 4.14 (q, J = 7.1 Hz, 2 H, CH_2), 3.89 (s, 3 H, OCH_3), 2.67 (s, 1 H, C≡CH), 2.47-2.29 (m, 2 H, CH_2), 2.15-2.02 (m, 1 H, one proton of CH_2), 1.99-1.82 (m, 3 H, protons of 2 × CH_2), 1.78 (s, 3 H, CH_3), 1.27 (t, J = 7.2 Hz, 3 H, CH_3); **^{13}C NMR** (100 MHz, $CDCl_3$) δ 172.9, 158.0, 155.5, 81.4, 78.1, 75.4, 60.3, 53.4, 40.3, 33.6, 25.9, 19.4, 14.1; **MS** (ESI) *m/z* 288 ($[M+NH_4]^+$), 293 ($[M+Na]^+$); **IR** (neat): ν = 3277, 2983, 2959, 2121, 1771, 1731, 1661, 1439, 1376, 1320, 1258, 1199, 1149, 1090, 1031 cm^{-1}; **HRMS** (ESI) calcd for $C_{13}H_{18}O_6Na$ ($[M+Na]^+$): 293.0996, Found: 293.0993.

(9) 2-(Adamantan-1-yl)but-3-yn-2-yl methyl oxalate **1l** (lq-7-199)

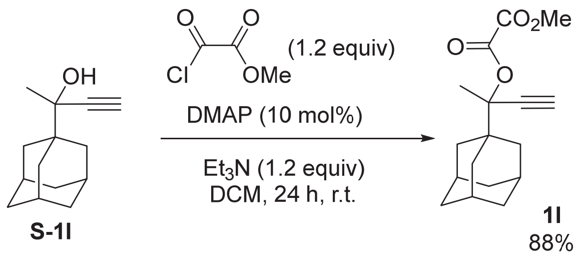

Following **Typical Procedure I**, the reaction of alkynol **S-1l** (4.0857 g, 20 mmol)/DCM (60 mL), methyl oxalyl chloride (2.25 mL, d = 1.332 g/mL, 2.9970 g, 23.98 mmol), DMAP (247.3 mg, 2 mmol), and NEt_3 (3.38 mL, d = 0.726 g/mL, 2.4539

g, 24 mmol) afforded **1l** (5.1246 g, 88%) (eluent: petroleum ether/ethyl ether = 20:1 (1000 mL) to petroleum ether/ethyl acetate = 10:1) as a liquid: **^{1}H NMR** (400 MHz, $CDCl_3$) δ 3.89 (s, 3 H, OCH_3), 2.64 (s, 1 H, C≡CH), 2.06 (s, 3 H, CH_3), 1.86-1.61 (m, 15 H); **^{13}C NMR** (100 MHz, $CDCl_3$) δ 158.4, 155.8, 84.2, 80.5, 76.2, 53.3, 40.0, 36.7, 35.8, 28.2, 19.1; **MS** (ESI) *m/z* 291 ($[M+H]^+$), 308 ($[M+NH_4]^+$), 313 ($[M+Na]^+$); **IR** (neat): ν = 3268, 2935, 2904, 2889, 2848, 1763, 1743, 1448, 1382, 1364, 1343, 1318, 1264, 1205, 1167, 1148, 1106, 1068, 1041 cm^{-1}; **HRMS** (ESI) calcd for $C_{17}H_{23}O_4$ ($[M+H]^+$): 291.1591, Found: 291.1591.

(10) Methyl (3-methyl-5-phenylpent-1-yn-3-yl) oxalate **1m (**lq-6-136)

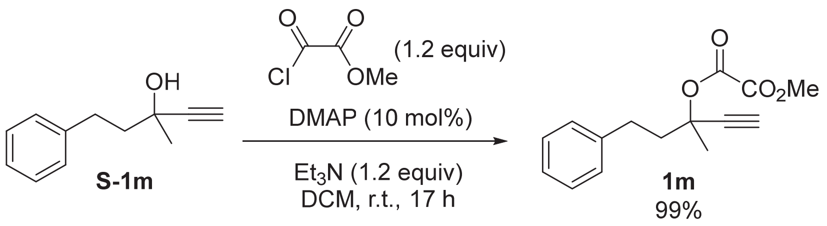

Following **Typical Procedure I**, the reaction of alkynol **S-1m** (622.9 mg, 3.57 mmol)/DCM (10.7 mL), methyl oxalyl chloride (0.4 mL, d = 1.332 g/mL, 532.8 mg, 4.26 mmol), DMAP (44.4 mg, 0.36 mmol), and NEt_3 (0.6 mL, d = 0.726 g/mL, 435.6 mg, 4.26 mmol) afforded **1m** (921.2 mg, 99%) (eluent: petroleum ether/ethyl acetate = 20:1) as a liquid: **^{1}H NMR** (400 MHz, $CDCl_3$) δ 7.35-7.14 (m, 5 H, ArH), 3.88 (s, 3 H, OCH_3), 2.95-2.79 (m, 2 H, CH_2), 2.72 (s, 1 H, C≡CH), 2.43-2.30 (m, 1 H, one proton of CH_2), 2.24-2.12 (m, 1 H, one proton of CH_2), 1.83 (s, 3 H, CH_3); **^{13}C NMR** (100 MHz, $CDCl_3$) δ 158.1, 155.6, 140.8, 128.43, 128.35, 126.1, 81.6, 78.2, 75.5, 53.4, 42.9, 30.4, 26.1; **MS** (ESI) *m/z* 283 ($[M+Na]^+$), 299 ($[M+K]^+$); **IR** (neat): ν = 3267, 3026, 2957, 2117, 1758, 1601, 1498, 1457, 1433, 1377, 1324, 1207, 1155, 1090, 1059, 1035 cm^{-1}; **HRMS** (ESI) calcd for $C_{15}H_{16}O_4Na$ ($[M+Na]^+$): 283.09408, Found: 283.09307.

(11) 1-(4-Fluorophenyl)-2-methylbut-3-yn-2-yl methyl oxalate **1n** (lq-7-021)

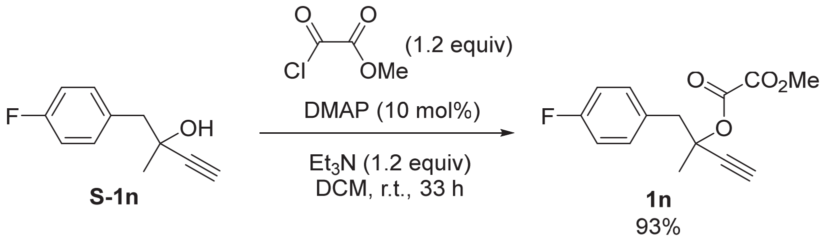

Following **Typical Procedure I**, the reaction of alkynol **S-1n** (837.8 mg, 4.7 mmol)/DCM (14 mL), methyl oxalyl chloride (0.53 mL, d = 1.332 g/mL, 706.0 mg, 5.65 mmol), DMAP (58.2 mg, 0.47 mmol), and NEt_3 (0.79 mL, d = 0.726 g/mL, 573.5 mg, 5.61 mmol) afforded **1n** (1.1525 g, 93%) (eluent: petroleum ether/ethyl ether = 20:1 (~525 mL) to 10:1 (550 mL)) as a liquid: **^{1}H NMR** (400 MHz, $CDCl_3$) δ 7.34-7.25 (m, 2 H, ArH), 7.00 (t, *J* = 8.8 Hz, 2 H, ArH), 3.89 (s, 3 H, OCH_3), 3.30 (d, *J* = 13.6 Hz, 1 H, one proton of CH_2), 3.18 (d, *J* = 13.6 Hz, 1 H, one proton of CH_2), 2.68 (s, 1 H, C≡CH), 1.73 (s, 3 H, CH_3); **^{13}C NMR** (100 MHz, $CDCl_3$) δ 162.2 (d, *J* = 244.1 Hz), 157.9, 155.4, 132.4 (d, *J* = 8.1 Hz), 130.2 (d, *J* = 3.2 Hz), 114.9 (d, *J* = 21.0 Hz), 81.4, 77.8 (d, *J* = 1.2 Hz), 76.4, 53.5, 45.9, 25.6; **^{19}F NMR** (376 MHz, $CDCl_3$) δ -115.9; **MS** (ESI) *m/z* 282 ($[M+NH_4]^+$), 287 ($[M+Na]^+$); **IR** (neat): ν = 3283, 2992, 2958, 2122, 1770, 1746, 1605, 1510, 1439, 1418, 1377, 1321, 1222, 1201, 1154, 1122, 1098, 1059, 1017; **HRMS** (ESI) calcd for $C_{14}H_{17}O_4NF$ ($[M+NH_4]^+$): 282.1136, Found: 282.1136.

(12) 1-(4-Chlorophenyl)-2-methylbut-3-yn-2-yl methyl oxalate **1o** (lq-6-160)

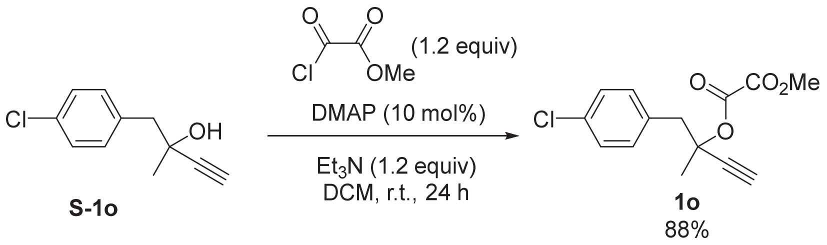

Following **Typical Procedure I**, the reaction of alkynol **S-1o** (581.5 mg, 3 mmol)/DCM (9 mL), methyl oxalyl chloride (0.34 mL, d = 1.332 g/mL, 452.9 mg, 3.62 mmol), DMAP (37.2 mg, 0.3 mmol), and NEt_3 (0.51 mL, d = 0.726 g/mL, 370.3 mg, 3.62 mmol) afforded **1o** (734.3 mg, 88%) (eluent: petroleum ether/ethyl acetate = 20:1) as a liquid: **^{1}H NMR** (400 MHz, $CDCl_3$) δ 7.33-7.20 (m, 4 H, ArH), 3.89 (s, 3 H, OCH_3), 3.30 (d, *J* = 14.0 Hz, 1 H, one proton of CH_2), 3.17 (d, *J* = 13.6 Hz, 1 H, one proton of CH_2), 2.68 (s, 1 H, C≡CH), 1.73 (s, 3 H, CH_3); **^{13}C NMR** (100 MHz, $CDCl_3$) δ 157.9,

155.4, 133.3, 133.0, 132.2, 128.2, 81.3, 77.7, 76.4, 53.5, 46.1, 25.7; **MS** (ESI) *m/z* 298 ([M(^{35}Cl)+NH_4]$^+$), 300 ([M(^{37}Cl)+NH_4]$^+$), 303 ([M(^{35}Cl)+Na]$^+$), 305 ([M(^{37}Cl)+Na]$^+$); **IR** (neat): ν = 3287, 2994, 2956, 2122, 1769, 1745, 1598, 1492, 1438, 1409, 1376, 1320, 1259, 1200, 1150, 1106, 1088, 1059, 1016 cm^{-1}; **HRMS** (ESI) calcd for $C_{14}H_{13}O_4Na^{35}Cl$ ([M(^{35}Cl)+Na]$^+$): 303.03946, Found: 303.03931.

(13) 1-(4-Bromophenyl)-2-methylbut-3-yn-2-yl methyl oxalate **1p** (lq-7-026)

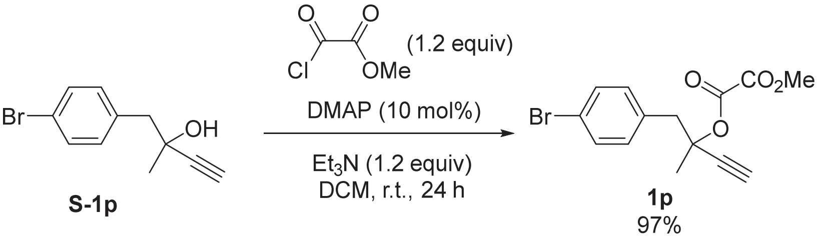

Following **Typical Procedure I**, the reaction of alkynol **S-1p** (1.4111 g, 5.9 mmol)/DCM (17.7 mL), methyl oxalyl chloride (0.66 mL, d = 1.332 g/mL, 879.1 mg, 7.03 mmol), DMAP (72.8 mg, 0.59 mmol), and NEt_3 (1 mL, d = 0.726 g/mL, 726.0 mg, 7.10 mmol) afforded **1p** (1.8570 g, 97%) (eluent: petroleum ether/ethyl ether = 20:1 (~525 mL) to 10:1 (550 mL)) as a liquid: **^{1}H NMR** (400 MHz, $CDCl_3$) δ 7.44 (d, *J* = 8.4 Hz, 2 H, ArH), 7.22 (d, *J* = 8.4 Hz, 2 H, ArH), 3.89 (s, 3 H, OCH_3), 3.28 (d, *J* = 13.6 Hz, 1 H, one proton of CH_2), 3.15 (d, *J* = 13.6 Hz, 1 H, one proton of CH_2), 2.68 (s, 1 H, C≡CH), 1.74 (s, 3 H, CH_3); **^{13}C NMR** (100 MHz, $CDCl_3$) δ 157.8, 155.4, 133.5, 132.6, 131.2, 121.5, 81.3, 77.6, 76.5, 53.5, 46.1, 25.7; **MS** (ESI) *m/z* 342 ([M(^{79}Br)+NH_4]$^+$), 344 ([M(^{81}Br)+NH_4]$^+$), 347 ([M(^{79}Br)+Na]$^+$), 349 ([M(^{81}Br)+Na]$^+$), 363 ([M(^{79}Br)+K]$^+$), 365 ([M(^{81}Br)+Na]$^+$); **IR** (neat): ν = 3286, 2989, 2955, 2121, 1769, 1745, 1592, 1489, 1438, 1406, 1375, 1320, 1244, 1200, 1152, 1103, 1059, 1012 cm^{-1}; **HRMS** (ESI) calcd for $C_{14}H_{13}O_4Na^{79}Br$ ([M(^{79}Br)+Na]$^+$): 346.9889, Found: 346.9892.

(14) 5-((4-Ethynylbenzoyl)oxy)-3-methylpent-1-yn-3-yl methyl oxalate **1q** (lq-8-043)

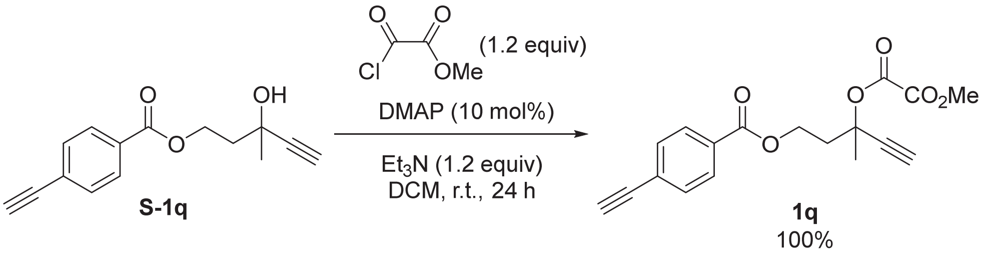

Following **Typical Procedure I**, the reaction of alkynol **S-1q** (1.7702 g, 7.3 mmol)/DCM (22 mL), methyl oxalyl chloride (0.82 mL, d = 1.332 g/mL, 1.0992 g, 8.74 mmol), DMAP (90.3 mg, 0.73 mmol), and NEt_3 (1.2 mL, d = 0.726 g/mL, 871.2 mg, 8.52 mmol) afforded **1q** (2.3903 g, 100%) (using Biotage Isolera One purification system on flash silica gel column (Santai Tech. Inc., 80 g), flowrate: 50 mL/min, eluent: petroleum ether/ethyl acetate = 98:2 (200 mL), 98:2 to 80:20 (linear gradient, 1000 mL), 80:20 (200 mL)) as a liquid: **^{1}H NMR** (400 MHz, $CDCl_3$) δ 7.99 (d, J = 8.4 Hz, 2 H, ArH), 7.55 (d, J = 8.4 Hz, 2 H, ArH), 4.60 (t, J = 6.6 Hz, 2 H, OCH_2), 3.82 (s, 3 H, OCH_3), 3.25 (s, 1 H, C≡CH), 2.72 (s, 1 H, C≡CH), 2.58 (dt, J_1 = 14.4 Hz, J_2 = 6.8 Hz, 1 H, one proton of CH_2), 2.43 (dt, J_1 = 14.4 Hz, J_2 = 7.0 Hz, 1 H, one proton of CH_2), 1.88 (s, 3 H, CH_3); **^{13}C NMR** (100 MHz, $CDCl_3$) δ 165.6, 157.8, 155.5, 132.0, 129.9, 129.5, 126.9, 82.7, 81.0, 80.2, 76.6, 76.1, 60.8, 53.4, 39.6, 26.5; **MS** (ESI) *m/z* 346 ($[M+NH_4]^+$), 351 ($[M+Na]^+$); **IR** (neat): ν = 3286, 3268, 3012, 2968, 2120, 2108, 1749, 1605, 1561, 1504, 1458, 1444, 1404, 1375, 1326, 1304, 1179, 1155, 1113, 1105, 1095, 1039, 1021 cm^{-1}; **HRMS** (ESI) calcd for $C_{18}H_{16}O_6Na$ ($[M+Na]^+$): 351.0839, Found: 351.0833.

(15) 5-(4-(Allyloxy)phenyl)-3-methylpent-1-yn-3-yl methyl oxalate **1r** (lq-8-085)

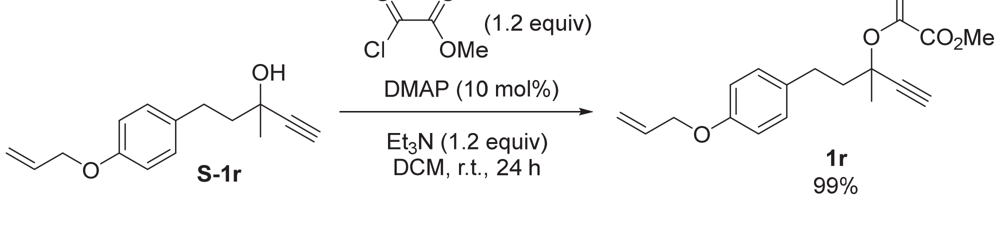

Following **Typical Procedure I**, the reaction of alkynol **S-1r** (3.3829 g, 14.7 mmol)/DCM (44 mL), methyl oxalyl chloride (1.66 mL, d = 1.332 g/mL, 2.2111 g, 17.69 mmol), DMAP (181.9 mg, 1.47 mmol), and NEt_3 (2.48 mL, d = 0.726 g/mL,

1.8005 g, 17.62 mmol) afforded **1r** (4.5901 g, 99%) (using Biotage Isolera One purification system on flash silica gel column (Santai Tech. Inc., 80 g), flowrate: 50 mL/min, eluent: petroleum ether/ethyl acetate = 98:2 (200 mL), 98:2 to 82:18 (linear gradient, 1000 mL), 82:18 (300 mL)) as a liquid: **^{1}H NMR** (400 MHz, $CDCl_3$) δ 7.12 (d, J = 8.8 Hz, 2 H, ArH), 6.84 (d, J = 8.8 Hz, 2 H, ArH), 6.12-5.98 (m, 1 H, =CH), 5.40 (dd, J_1 = 17.2 Hz, J_2 = 1.6 Hz, 1 H, one proton of =CH_2), 5.27 (dd, J_1 = 10.4 Hz, J_2 = 1.6 Hz, 1 H, one proton of =CH_2), 4.51 (d, J = 5.2 Hz, 2 H, OCH_2), 3.88 (s, 3 H, OCH_3), 2.88-2.73 (m, 2 H, CH_2), 2.71 (s, 1 H, C≡CH), 2.38-2.26 (m, 1 H, one proton of CH_2), 2.21-2.09 (m, 1 H, one proton of CH_2), 1.81 (s, 3 H, CH_3); **^{13}C NMR** (100 MHz, $CDCl_3$) δ 158.1, 156.9, 155.6, 133.3, 133.0, 129.2, 117.5, 114.7, 81.6, 78.2, 75.4, 68.7, 53.4, 43.0, 29.4, 26.1; **MS** (ESI) *m/z* 334 ($[M+NH_4]^+$), 339 ($[M+Na]^+$), 355 ($[M+K]^+$); **IR** (neat): ν = 3278, 2942, 2867, 2121, 1770, 1746, 1611, 1583, 1510, 1457, 1377, 1320, 1240, 1202, 1152, 1104, 1070, 1023 cm^{-1}; **HRMS** (ESI) calcd for $C_{18}H_{20}O_5Na$ ($[M+Na]^+$): 339.1203, Found: 339.1203.

(16) 5-(Benzo[d][1,3]dioxol-5-yl)-3-methylpent-1-yn-3-yl methyl oxalate **1s** (lq-7-037)

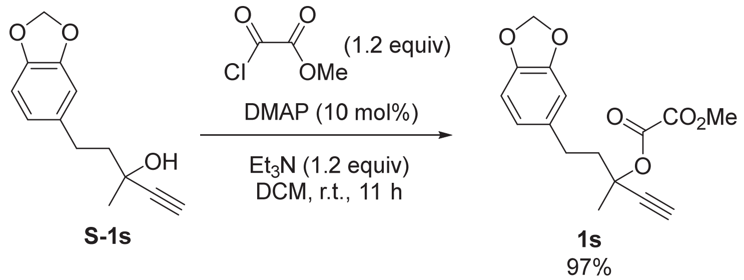

Following **Typical Procedure I**, the reaction of alkynol **S-1s** (1.8003 g, 8.25 mmol)/DCM (25 mL), methyl oxalyl chloride (0.94 mL, d = 1.332 g/mL, 1.2521 g, 10 mmol), DMAP (103.2 mg, 0.836 mmol), and NEt_3 (1.4 mL, d = 0.726 g/mL, 1.0164 g, 9.94 mmol) afforded **1s** (2.4269 g, 97%) (eluent: petroleum ether/ethyl ether = 10:1 (550 mL) to petroleum ether/ethyl acetate = 10:1 (1100 mL)) as a liquid: **^{1}H NMR** (400 MHz, $CDCl_3$) δ 6.73 (d, J = 7.6 Hz, 1 H, ArH), 6.70 (d, J = 1.2 Hz, 1 H, ArH), 6.66 (dd, J_1 = 8.2 Hz, J_2 = 1.4 Hz, 1 H, ArH), 5.92 (s, 2 H, OCH_2O), 3.89 (s, 3 H, OCH_3), 2.86-2.73 (m, 2 H, CH_2), 2.71 (s, 1 H, C≡CH), 2.37-2.25 (m, 1 H, one proton of CH_2), 2.19-

2.08 (m, 1 H, one proton of CH_2), 1.81 (s, 3 H, CH_3); **^{13}C NMR** (100 MHz, $CDCl_3$) δ 158.1, 155.6, 147.6, 145.8, 134.6, 121.1, 108.9, 108.2, 100.8, 81.6, 78.1, 75.5, 53.5, 43.2, 30.1, 26.1; **MS** (ESI) *m/z* 322 ($[M+NH_4]^+$), 327 ($[M+Na]^+$), 343 ($[M+K]^+$); **IR** (neat): ν = 3271, 2955, 2900, 2118, 1766, 1744, 1610, 1504, 1486, 1458, 1438, 1380, 1306, 1268, 1249, 1203, 1156, 1100, 1072, 1033 cm^{-1}; **HRMS** (ESI) calcd for $C_{16}H_{16}O_6Na$ ($[M+Na]^+$): 327.0839, Found: 327.0842.

(17) Methyl (2-methyl-1-(thien-3-yl)but-3-yn-2-yl) oxalate **1t** (lq-7-087)

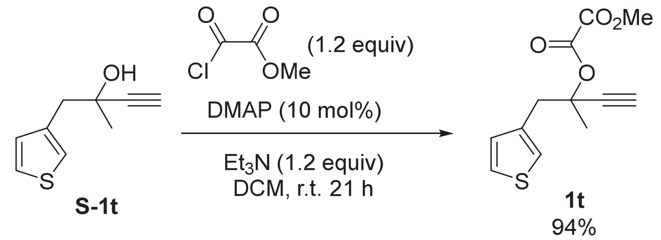

Following **Typical Procedure I**, the reaction of alkynol **S-1t** (457.4 mg, 2.75 mmol)/DCM (8.3 mL), methyl oxalyl chloride (0.31 mL, d = 1.332 g/mL, 412.9 mg, 3.3 mmol), DMAP (34.0 mg, 0.276 mmol), and NEt_3 (0.46 mL, d = 0.726 g/mL, 334.0 mg, 3.27 mmol) afforded **1t** (655.4 mg, 94%) (eluent: petroleum ether/ethyl ether = 20:1 (~210 mL) to 10:1 (440 mL)) as a liquid: **^{1}H NMR** (400 MHz, $CDCl_3$) δ 7.26 (dd, J_1 = 4.8 Hz, J_2 = 3.2 Hz, 1 H, ArH), 7.18 (d, J = 2.0 Hz, 1 H, ArH), 7.10 (d, J = 4.8 Hz, 1 H, ArH), 3.89 (s, 3 H, OCH_3), 3.35 (d, J = 14.4 Hz, 1 H, one proton of CH_2), 3.30 (d, J = 14.0 Hz, 1 H, one proton of CH_2), 2.67 (s, 1 H, C≡CH), 1.72 (s, 3 H, CH_3); **^{13}C NMR** (100 MHz, $CDCl_3$) δ 158.0, 155.5, 134.6, 129.6, 125.0, 124.4, 81.8, 77.7, 75.8, 53.5, 41.3, 25.6; **MS** (EI) *m/z* (%) 252 (M^+, 2.4), 97 (100); **IR** (neat): ν = 3279, 3107, 2991, 2955, 2121, 1768, 1744, 1438, 1413, 1376, 1321, 1281, 1201, 1150, 1112, 1083, 1057 cm^{-1}; **HRMS** (EI) calcd for $C_{12}H_{12}O_4S$ (M^+): 252.0451, Found: 252.0451.

(18) **1u** (lq-7-110)

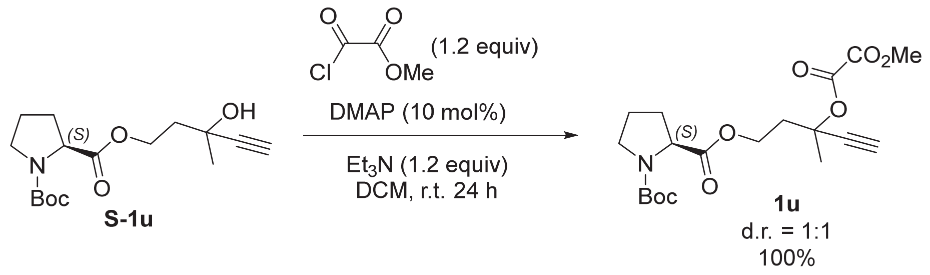

Following **Typical Procedure I**, the reaction of alkynol **S-1u** (858.6 mg, 2.76 mmol)/DCM (8.3 mL), methyl oxalyl chloride (0.31 mL, d = 1.332 g/mL, 412.9 mg, 3.30 mmol), DMAP (34.5 mg, 0.28 mmol), and NEt_3 (0.47 mL, d = 0.726 g/mL, 341.2 mg, 3.34 mmol) afforded **1u** (1.0952 g, 100%, d.r. = 1:1) (eluent: petroleum ether/ethyl acetate = 5:1 (~240 mL) to 3:1 (600 mL)) as a liquid: The d.r. ratio of **1u** was determined to 1:1 by HPLC (HPLC conditions: Chiralpak OD-H column, hexane/*i*-PrOH = 90/10, 1.0 mL/min, λ = 214 nm, t_R = 28.6 min, t_R = 30.9 min); **^{1}H NMR** (400 MHz, $CDCl_3$, a mixture of rotamers) δ 4.46-4.34 (m, 2 H, OCH_2), [4.32 (dd, J_1 = 8.4 Hz, J_2 = 3.2 Hz, 0.48 H), 4.22 (dd, J_1 = 8.6 Hz, J_2 = 3.8 Hz, 0.56 H), 1 H, NCH], 3.89 (s, 3 H, OCH_3), 3.60-3.34 (m, 2 H, NCH_2), [2.72 (s, 0.58 H), 2.71 (s, 0.45 H), 1 H, C≡CH], 2.50-2.37 (m, 1 H), 2.34-2.11 (m, 2 H), 2.03-1.84 (m, 3 H), [1.83 (s, 1.67 H), 1.82 (s, 1.34 H), 3 H, CH_3], [1.46 (s, 3.92 H), 1.42 (s, 4.97 H), 9 H, $OC(CH_3)_3$]; **MS** (ESI) *m/z* 398 ($[M+H]^+$), 415 ($[M+NH_4]^+$); **IR** (neat): ν = 3270, 2976, 2882, 2119, 1771, 1744, 1693, 1478, 1393, 1366, 1320, 1279, 1257, 1198, 1153, 1119, 1087 cm^{-1}; **HRMS** (ESI) calcd for $C_{19}H_{28}O_8N$ ($[M+H]^+$): 398.1809, Found: 398.1801.

(19) **1v** (lq-7-116)

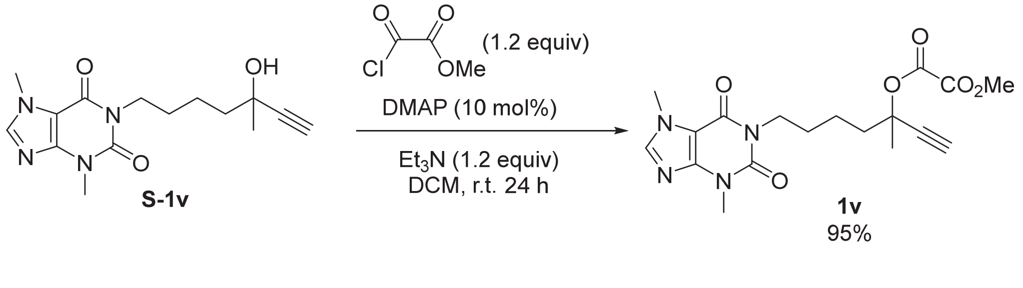

Following **Typical Procedure I**, the reaction of alkynol **S-1v** (681.7 mg, 2.24 mmol)/DCM (6.7 mL), methyl oxalyl chloride (0.25 mL, d = 1.332 g/mL, 333.0 mg, 2.66 mmol), DMAP (27.9 mg, 0.226 mmol), and NEt_3 (0.38 mL, d = 0.726 g/mL, 275.9

mg, 2.70 mmol) afforded **1v** (829.2 mg, 95%) (eluent: dichloromethane (200 mL) to dichloromethane/MeOH = 100:1 (~500 mL)) as a liquid: **^{1}H NMR** (400 MHz, $CDCl_3$) δ 7.56 (s, 1 H, CH), 4.02 (t, *J* = 7.2 Hz, 2 H, NCH_2), 4.01 (s, 3 H, OCH_3), 3.89 (s, 3 H, NCH_3), 3.58 (s, 3 H, NCH_3), 2.68 (s, 1 H, C≡CH), 2.15-2.04 (m, 1 H, one proton of CH_2), 2.01-1.90 (m, 1 H, one proton of CH_2), 1.77 (s, 3 H, CH_3), 1.75-1.51 (m, 4 H, 2 × CH_2); **^{13}C NMR** (100 MHz, $CDCl_3$) δ 158.0, 155.5, 155.0, 151.2, 148.6, 141.4, 107.5, 81.7, 78.3, 75.2, 53.3, 40.8, 40.6, 33.4, 29.5, 27.5, 25.9, 21.3; **MS** (ESI) *m/z* 391 ($[M+H]^+$), 413 ($[M+Na]^+$), 429 ($[M+K]^+$); **IR** (neat): ν = 3248, 3118, 2952, 2871, 2117, 1768, 1746, 1699, 1651, 1604, 1548, 1486, 1454, 1436, 1415, 1358, 1321, 1287, 1233, 1202, 1151, 1091, 1046 cm^{-1}; **HRMS** (ESI) calcd for $C_{18}H_{23}O_6N_4$ ($[M+H]^+$): 391.1612, Found: 391.1611.

(20) **1w** (lq-7-048)

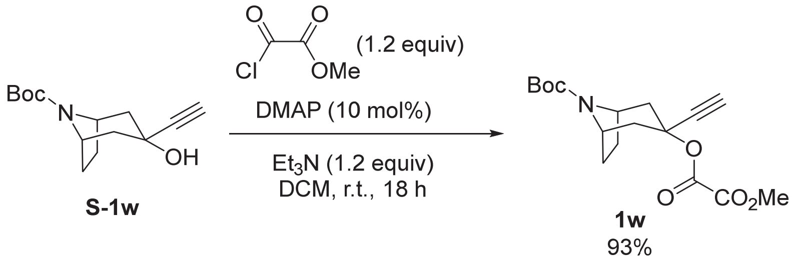

Following **Typical Procedure I**, the reaction of alkynol **S-1w** (754.0 mg, 3.00 mmol)/DCM (9 mL), methyl oxalyl chloride (0.34 mL, d = 1.332 g/mL, 452.9 mg, 3.62 mmol), DMAP (38.1 mg, 0.31 mmol), and NEt_3 (0.51 mL, d = 0.726 g/mL, 370.3 mg, 3.62 mmol) afforded **1w** (941.7 mg, 93%) (eluent: petroleum ether/ethyl acetate/dichloromethane = 10:1:1 (480 mL)) as a white solid (m.p. 118.6-119.3 °C, *n*-hexane): **^{1}H NMR** (400 MHz, $CDCl_3$) δ 4.34-4.11 (m, 2 H, 2 × NCH), 3.91 (s, 3 H, OCH_3), 2.67 (s, 1 H, C≡CH), 2.60-2.46 (m, 2 H, two protons of 2 × CH_2), 2.45-2.19 (m, 2 H, two protons of 2 × CH_2), 2.03-1.88 (m, 4 H, 2 × CH_2), 1.48 (s, 9 H, 3 × CH_3); **^{13}C NMR** (100 MHz, $CDCl_3$) δ 158.0, 155.4, 153.1, 82.1, 79.8, 75.9, 74.8, 53.6, 52.4, 51.6, 41.0, 40.4, 28.4, 27.8, 27.1; **MS** (ESI) *m/z* 360 ($[M+Na]^+$); **IR** (neat): ν = 3262, 2974, 2959, 2930, 2119, 1770, 1741, 1687, 1474, 1445, 1431, 1396, 1365, 1324, 1270, 1243, 1228, 1197, 1157, 1124, 1098, 1067, 1045, 1006 cm^{-1}; Anal. Calcd. for $C_{17}H_{23}NO_6$: C

60.52, H 6.87, N 4.15; Found: C 60.47, H 7.01, N 3.81.

(21) **1x** (lq-7-191)

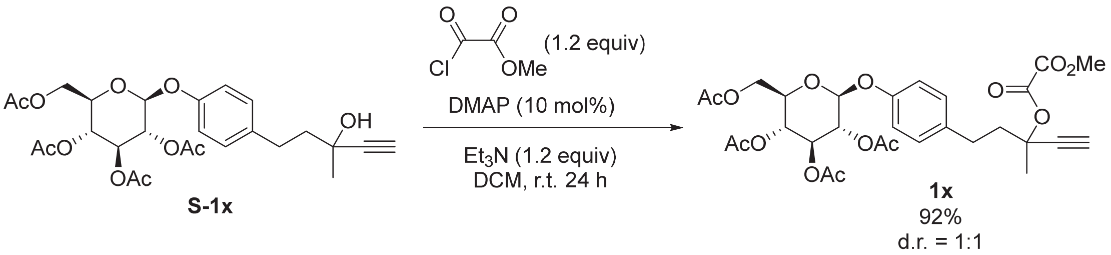

Following **Typical Procedure I**, the reaction of alkynol **S-1x** (774.1 mg, 1.49 mmol)/DCM (4.5 mL), methyl oxalyl chloride (0.17 mL, d = 1.332 g/mL, 226.4 mg, 1.81 mmol), DMAP (18.4 mg, 0.149 mmol), and NEt_3 (0.25 mL, d = 0.726 g/mL, 181.5 mg, 1.78 mmol) afforded **1x** (832.6 mg, 92%, d.r. = 1:1) (eluent: petroleum ether/ethyl acetate = 3:1 (400 mL) to 2:1 (900 mL)) as a liquid: The d.r. ratio of **1x** was determined to 1:1 by HPLC (HPLC conditions: Chiralpak IC column, hexane/*i*-PrOH = 60/40, 1.0 mL/min, λ = 214 nm, t_R = 23.1 min, t_R = 26.8 min); **^{1}H NMR** (400 MHz, $CDCl_3$) δ 7.14 (d, *J* = 8.4 Hz, 2 H, ArH), 6.92 (d, *J* = 8.8 Hz, 2 H, ArH), 5.34-5.22 (m, 2 H, 2 × OCH), 5.17 (t, *J* = 9.4 Hz, 1 H, OCH), 5.05 (d, *J* = 7.2 Hz, 1 H, OCH), 4.29 (dd, J_1 = 12.4 Hz, J_2 = 5.6 Hz, 1 H, one proton of OCH_2), 4.17 (dd, J_1 = 12.4 Hz, J_2 = 2.4 Hz, 1 H, one proton of OCH_2), 3.87 (s, 3 H, OCH_3), 3.90-3.82 (m, 1 H, OCH), 2.90-2.75 (m, 2 H, CH_2), 2.72 (s, 1 H, C≡CH), 2.38-2.27 (m, 1 H, one proton of CH_2), 2.20-2.10 (m, 1 H, one proton of CH_2), 2.09 (s, 3 H, OCH_3), 2.06 (s, 3 H, OCH_3), 2.05 (s, 3 H, OCH_3), 2.04 (s, 3 H, OCH_3), 1.82 (s, 3 H, CH_3); **^{13}C NMR** (100 MHz, $CDCl_3$) δ 170.6, 170.2, 169.4, 169.3, 158.0, 155.6, 155.2, 135.8, 129.4, 117.0, 99.2, 81.5, 78.1, 75.5, 72.6, 71.9, 71.1, 68.2, 61.9, 53.5, 42.9, 29.6, 26.1, 20.64, 20.56, 20.54, 20.51; **MS** (ESI) *m/z* 624 ($[M+NH_4]^+$)); **IR** (neat): ν = 3271, 2958, 2115, 1743, 1611, 1510, 1437, 1368, 1323, 1209, 1156, 1066, 1034 cm^{-1}; Anal. Calcd. for $C_{29}H_{34}O_{14}$: C 57.42, H 5.65; Found: C 56.81, H 5.49.

(22) 1-Cyclohexylprop-2-ynyl methyl oxalate **1z** (lq-6-151)

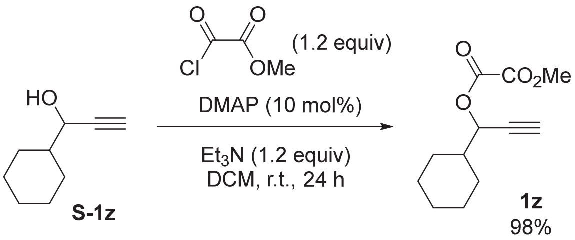

Following **Typical Procedure I**, the reaction of alkynol **S-1z** (474.1 mg, 3.4 mmol)/DCM (10 mL), methyl oxalyl chloride (0.38 mL, d = 1.332 g/mL, 506.1 mg, 4.05 mmol), DMAP (42.3 mg, 0.34 mmol), and NEt_3 (0.58 mL, d = 0.726 g/mL, 421.1 mg, 4.12 mmol) afforded **1z** (757.2 mg, 98%) (eluent: petroleum ether/ethyl ether = 20:1) as a liquid: **^{1}H NMR** (400 MHz, $CDCl_3$) δ 5.30 (dd, J_1 = 6.0 Hz, J_2 = 2.0 Hz, 1 H, OCH), 3.92 (s, 3 H, OCH_3), 2.55 (d, J = 2.0 Hz, 1 H, CH), 1.96-1.63 (m, 6 H), 1.35-1.05 (m, 5 H); **^{13}C NMR** (100 MHz, $CDCl_3$) δ 157.8, 156.6, 78.4, 75.7, 70.9, 53.6, 41.3, 28.2, 27.8, 25.9, 25.5, 25.4; **MS** (ESI) *m/z* 242 ($[M+NH_4]^+$), 247 ($[M+Na]^+$); **IR** (neat): ν = 3283, 2930, 2855, 2125, 1771, 1745, 1451, 1344, 1308, 1199, 1157, 1083, 1066, 1053, 1006 cm^{-1}; **HRMS** (ESI) calcd for $C_{12}H_{16}O_4Na$ ($[M+Na]^+$): 247.09408, Found: 247.09414.

(23) Methyl oct-1-yn-3-yl oxalate **1A** (lq-6-120)

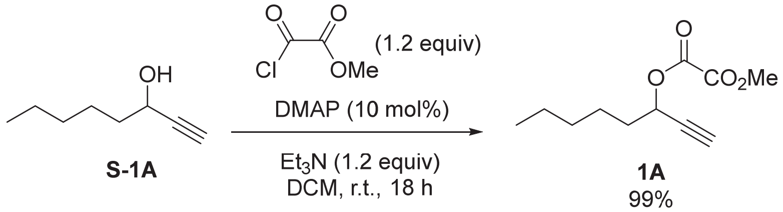

Following **Typical Procedure I**, the reaction of alkynol **S-1A** (631.2 mg, 5 mmol)/DCM (15 mL), methyl oxalyl chloride (0.56 mL, d = 1.332 g/mL, 745.9 mg, 5.97 mmol), DMAP (61.8 mg, 0.5 mmol), and NEt_3 (0.84 mL, d = 0.726 g/mL, 609.8 mg, 5.97 mmol) afforded **1A** (1.0485 g, 99%) (eluent: petroleum ether/ethyl ether = 20:1) as a liquid: **^{1}H NMR** (400 MHz, $CDCl_3$) δ 5.46 (t, J = 6.6 Hz, 1 H, OCH), 3.92 (s, 3 H, OCH_3), 2.56 (d, J = 2.0 Hz, 1 H, C≡CH), 1.96-1.82 (m, 2 H, CH_2), 1.56-1.42 (m, 2 H, CH_2), 1.39-1.26 (m, 4 H, 2 × CH_2), 0.90 (t, J = 6.6 Hz, 3 H, CH_3); **^{13}C NMR** (100 MHz, $CDCl_3$) δ 157.7, 156.5, 79.4, 75.1, 66.8, 53.6, 34.2, 31.0, 24.4, 22.3, 13.8; **MS** (ESI) *m/z* 230 ($[M+NH_4]^+$), 235 ($[M+Na]^+$), 251 ($[M+K]^+$); **IR** (neat): ν = 3287,

2961, 2933, 2862, 1773, 1747, 1456, 1439, 1307, 1259, 1199, 1158, 1115, 1081, 1013 cm^{-1}; **HRMS** (ESI) calcd for $C_{11}H_{16}O_4Na$ ([M+Na]$^+$): 235.09408, Found: 235.09398.

(24) 1-Phenylprop-2-ynyl methyl oxalate **1J** (lq-9-146)

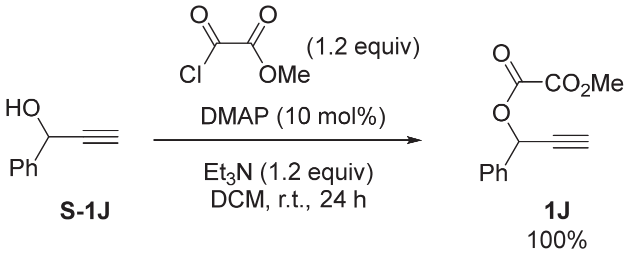

Following **Typical Procedure I**, the reaction of alkynol **S-1J** (1.3484 g, 10 mmol)/DCM (30 mL), methyl oxalyl chloride (1.1 mL, d = 1.332 g/mL, 1.4652 g, 12 mmol), DMAP (123.5 mg, 1 mmol), and NEt_3 (1.7 mL, d = 0.726 g/mL, 1.2342 g, 12 mmol) afforded **1J** (2.1806 g, 100%) (using Biotage Isolera One purification system on flash silica gel column (Santai Tech. Inc., 40 g), flowrate: 40 mL/min, eluent: petroleum ether (100 mL), petroleum ether to petroleum ether/ethyl acetate = 90:10 (linear gradient, 250 mL), 90:10 (500 mL)) as a white solid (m.p. 33.5-34.1 ºC, petroleum ether/ethyl ether): **^{1}H NMR** (400 MHz, $CDCl_3$) δ 7.65-7.52 (m, 2 H, ArH), 7.45-7.32 (m, 3 H, ArH), 6.53 (d, *J* = 2.4 Hz, 1 H, OCH), 3.86 (s, 3 H, OCH_3), 2.78 (d, *J* = 2.0 Hz, 1 H, C≡CH); **^{13}C NMR** (100 MHz, $CDCl_3$) δ 157.4, 156.2, 134.7, 129.6, 128.7, 127.9, 78.4, 77.2, 68.0, 53.6; **MS** (ESI) *m/z* 241 ([M+Na]$^+$); **IR** (neat): ν = 3280, 3037, 3013, 2130, 1758, 1740, 1498, 1458, 1439, 1336, 1297, 1282, 1202, 1171, 1156, 1081, 1033, 1008 cm^{-1}; Anal. Calcd. for $C_{12}H_{10}O_4$: C 66.05, H 4.62; Found: C 65.78, H 4.52.

(25) Methyl (1-(prop-1-yn-1-yl)cyclohexyl) oxalate **1B** (lq-7-075)

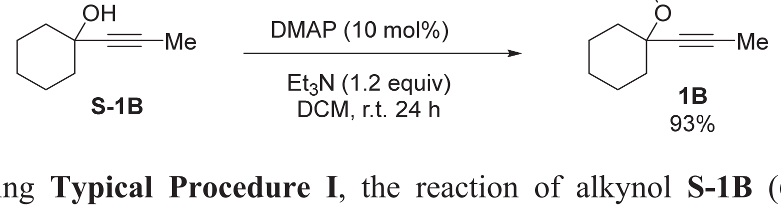

Following **Typical Procedure I**, the reaction of alkynol **S-1B** (691.5 mg, 5 mmol)/DCM (15 mL), methyl oxalyl chloride (0.56 mL, d = 1.332 g/mL, 745.9 mg,

5.97 mmol), DMAP (61.9 mg, 0.5 mmol), and NEt_3 (0.84 mL, d = 0.726 g/mL, 609.8 mg, 5.97 mmol) afforded **1B** (1.0197 g, 93%) (eluent: petroleum ether/ethyl ether = 20:1 (~210 mL) to 10:1 (~440 mL)) as a liquid: **^{1}H NMR** (400 MHz, $CDCl_3$) δ 3.89 (s, 3 H, OCH_3), 2.21-2.10 (m, 2 H), 1.99-1.90 (m, 2 H), 1.88 (s, 3 H, CH_3), 1.73-1.46 (m, 5 H), 1.41-1.27 (m, 1 H); **^{13}C NMR** (100 MHz, $CDCl_3$) δ 158.5, 155.6, 84.0, 80.1, 77.4, 53.3, 36.9, 24.9, 22.6, 3.6; **MS** (ESI) *m/z* 247 ($[M+Na]^+$), 471 ($[2M+Na]^+$); **IR** (neat): ν = 2938, 2862, 2247, 1770, 1745, 1447, 1309, 1259, 1200, 1158, 1123, 1082, 1012 cm^{-1}; **HRMS** (ESI) calcd for $C_{12}H_{16}O_4Na$ ($[M+Na]^+$): 247.0941, Found: 247.0938.

(26) Methyl (1-((trimethylsilyl)ethynyl)cyclohexyl) oxalate **1C** (lq-7-117)

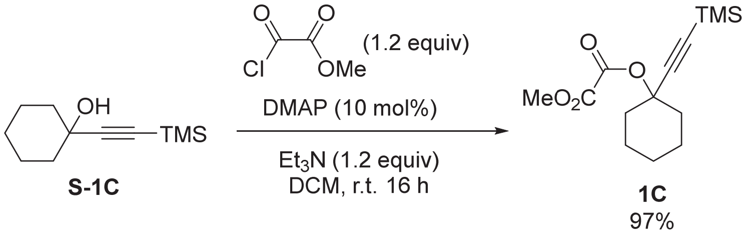

Following **Typical Procedure I**, the reaction of alkynol **S-1C** (1.8510 g, 9.43 mmol)/DCM (28 mL), methyl oxalyl chloride (1.06 mL, d = 1.332 g/mL, 1.4119 g, 11.3 mmol), DMAP (116.7 mg, 0.946 mmol), and NEt_3 (1.59 mL, d = 0.726 g/mL, 1.1543 g, 11.3 mmol) afforded **1C** (2.5817 g, 97%) (eluent: petroleum ether/ethyl ether = 20:1) as a liquid: **^{1}H NMR** (400 MHz, $CDCl_3$) δ 3.89 (s, 3 H, OCH_3), 2.26-2.11 (m, 2 H), 1.99-1.84 (m, 2 H), 1.74-1.48 (m, 5 H), 1.40-1.25 (m, 1 H), 0.17 (s, 9 H, $Si(CH_3)_3$); **^{13}C NMR** (100 MHz, $CDCl_3$) δ 158.4, 155.3, 103.0, 92.7, 79.5, 53.3, 36.7, 24.9, 22.6, -0.3; **MS** (ESI) *m/z* 305 ($[M+Na]^+$), 321 ($[M+K]^+$); **IR** (neat): ν = 2939, 2863, 2175, 1772, 1747, 1448, 1314, 1299, 1250, 1198, 1150, 1120, 1015 cm^{-1}; **HRMS** (ESI) calcd for $C_{14}H_{22}O_4NaSi$ ($[M+Na]^+$): 305.1180, Found: 305.1179.

(27) Methyl (1-(cyclohexylethynyl)cyclohexyl) oxalate **1K** (lq-9-158)

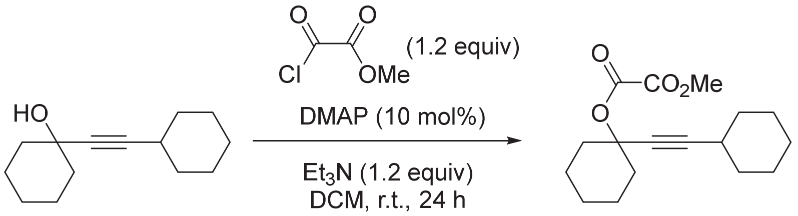

Following **Typical Procedure I**, the reaction of alkynol **S-1K** (1.6644 g, 8.1 mmol)/DCM (24 mL), methyl oxalyl chloride (0.9 mL, d = 1.332 g/mL, 1.1988 g, 9.59 mmol), DMAP (101.0 mg, 0.82 mmol), and NEt_3 (1.4 mL, d = 0.726 g/mL, 1.0164 g, 9.94 mmol) afforded **1K** (2.2794 g, 97%) (using Biotage Isolera One purification system on flash silica gel column (Santai Tech. Inc., 40 g), flowrate: 40 mL/min, eluent: petroleum ether (100 mL), petroleum ether to petroleum ether/ethyl acetate = 95:5 (linear gradient, 100 mL), 95:5 (500 mL)) as a liquid: **^{1}H NMR** (400 MHz, $CDCl_3$) δ 3.88 (s, 3 H, OCH_3), 2.50-2.39 (m, 1 H, CH), 2.23-2.11 (m, 2 H), 1.95-1.84 (m, 2 H), 1.81-1.24 (m, 16 H); **^{13}C NMR** (100 MHz, $CDCl_3$) δ 158.6, 155.5, 92.7, 80.3, 78.4, 53.3, 37.1, 32.3, 28.8, 25.8, 25.0, 24.5, 22.8; **MS** (ESI) *m/z* 315 ($[M+Na]^+$); **IR** (neat): ν = 2929, 2855, 2242, 1771, 1745, 1448, 1311, 1260, 1198, 1155, 1123, 1012 cm^{-1}; **HRMS** (ESI) calcd for $C_{17}H_{24}O_4Na$ ($[M+Na]^+$): 315.1567, Found: 315.1555.

(28) Methyl (1-(phenylethynyl)cyclohexyl) oxalate **1D** (lq-7-153)

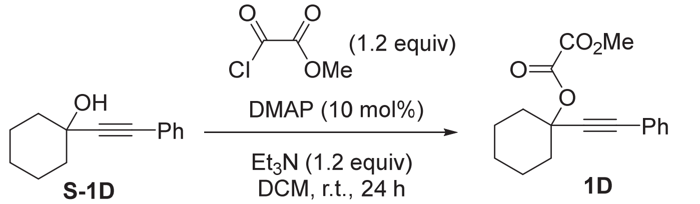

Following **Typical Procedure I**, the reaction of alkynol **S-1D** (2.0167 g, 10 mmol)/DCM (30 mL), methyl oxalyl chloride (1.1 mL, d = 1.332 g/mL, 1.4652 g, 11.7 mmol), DMAP (124.1 mg, 1 mmol), and NEt_3 (1.7 mL, d = 0.726 g/mL, 1.2342 g, 12.1 mmol) afforded **1D** (2.4109 g, 84%) (eluent: petroleum ether/ethyl acetate = 20:1) as a liquid: **^{1}H NMR** (400 MHz, $CDCl_3$) δ 7.48-7.40 (m, 2 H, ArH), 7.35-7.24 (m, 3 H, ArH), 3.89 (s, 3 H, OCH_3), 2.37-2.24 (m, 2 H), 2.09-1.96 (m, 2 H), 1.80-1.53 (m, 5 H),

1.45-1.31 (m, 1 H); **^{13}C NMR** (100 MHz, $CDCl_3$) δ 158.4, 155.5, 131.8, 128.5, 128.1, 122.2, 87.7, 87.1, 79.9, 53.4, 36.8, 24.9, 22.7; **MS** (ESI) *m/z* 309 ([M+Na]$^+$); **IR** (neat): ν = 2937, 2861, 2230, 1768, 1743, 1598, 1490, 1443, 1314, 1197, 1150, 1127, 1071, 1012 cm^{-1}; **HRMS** (ESI) calcd for $C_{17}H_{18}O_4Na$ ([M+Na]$^+$): 309.1097, Found: 309.1090.

(29) 1-(3-Methoxy-3-oxoprop-1-yn-1-yl)cyclohexyl methyl oxalate **1E** (lq-7-184)

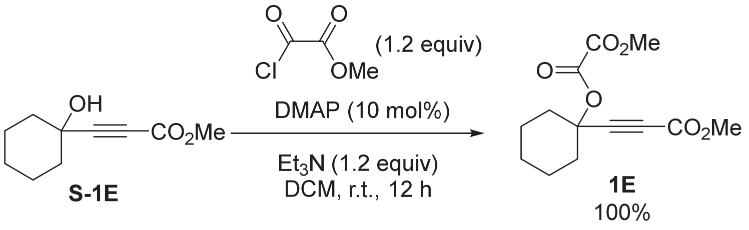

Following **Typical Procedure I**, the reaction of alkynol **S-1E** (1.7860 g, 9.8 mmol)/DCM (29.4 mL), methyl oxalyl chloride (1.1 mL, d = 1.332 g/mL, 1.4652 g, 11.72 mmol), DMAP (121.0 mg, 0.98 mmol), and NEt_3 (1.7 mL, d = 0.726 g/mL, 1.2342 g, 12.07 mmol) afforded **1E** (2.6256 g, 100%) (eluent: petroleum ether/ethyl acetate = 20:1 (~315 mL) to 10:1 (550 mL)) as a liquid: **^{1}H NMR** (400 MHz, $CDCl_3$) δ 3.90 (s, 3 H, OCH_3), 3.78 (s, 3 H, OCH_3), 2.32-2.16 (m, 2 H), 2.07-1.92 (m, 2 H), 1.80-1.51 (m, 5 H), 1.47-1.30 (m, 1 H); **^{13}C NMR** (100 MHz, $CDCl_3$) δ 157.8, 155.3, 153.4, 84.6, 78.9, 77.8, 53.5, 52.8, 35.9, 24.6, 22.2; **MS** (ESI) *m/z* 286 ([M+NH_4]$^+$), 291([M+Na]$^+$); **IR** (neat): ν = 2943, 2864, 2237, 1772, 1747, 1715, 1437, 1306, 1244, 1198, 1149, 1117, 1047, 1015 cm^{-1}; **HRMS** (ESI) calcd for $C_{13}H_{16}O_6Na$ ([M+Na]$^+$): 291.0839, Found: 291.0829.

Synthesis of allenenitriles

(1) 4,4-Pentamethylene-2,3-butadienenitrile **2a** (lq-6-119)

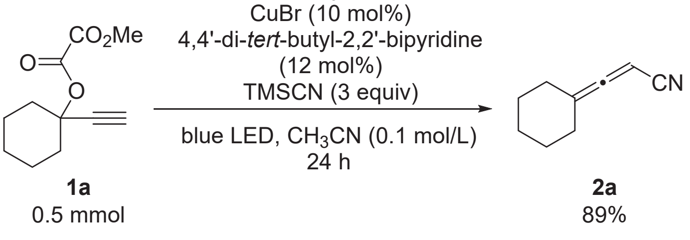

Typical Procedure II, to a flame-dried 10 mL Schlenk tube were added *fac*-Ir(ppy)$_3$

(3.3 mg, 5 μmol), CuBr (7.3 mg, 0.05 mmol), 4,4'-di-*tert*-butyl-2,2'-bipyridine (16.4 mg, 0.06 mmol), **1a** (105.4 mg, 0.5 mmol)/CH_3CN(2.5 mL), and TMSCN (157.2 mg, 1.5 mmol)/CH_3CN(2.5 mL) sequentially under Ar atmosphere. The resulting mixture was irradiated with a 50 W 460 nm blue LED lamp (2-3 cm away, with cooling fan to keep the reaction temperature at 35~40 °C) for 24 h with stirring and monitored by TLC. The resulting mixture was filtrated through a short pad of silica gel eluted with ethyl ether (30 mL). After evaporation, the residue was purified by chromatography on silica gel (eluent: petroleum ether/ethyl ether = 50:1) to afford **2a** (59.1 mg, 89%) as a liquid: **^{1}H NMR** (400 MHz, $CDCl_3$) δ 5.08-5.03 (m, 1 H, =CH), 2.30-2.12 (m, 4 H), 1.79-1.45 (m, 6 H); **^{13}C NMR** (100 MHz, $CDCl_3$) δ 210.4, 114.6, 108.2, 64.5, 29.7, 26.4, 25.3; **MS** (EI) *m/z* (%) 133 (M^+, 38.38), 132 ($[M-H]^+$, 95.07), 91 (100); **IR** (neat): ν = 2932, 2855, 2222, 1958, 1445, 1408, 1344, 1322, 1262, 1237 cm^{-1}; **HRMS** (EI) calcd for $C_9H_{11}N$ (M^+): 133.0891, Found: 133.0890.

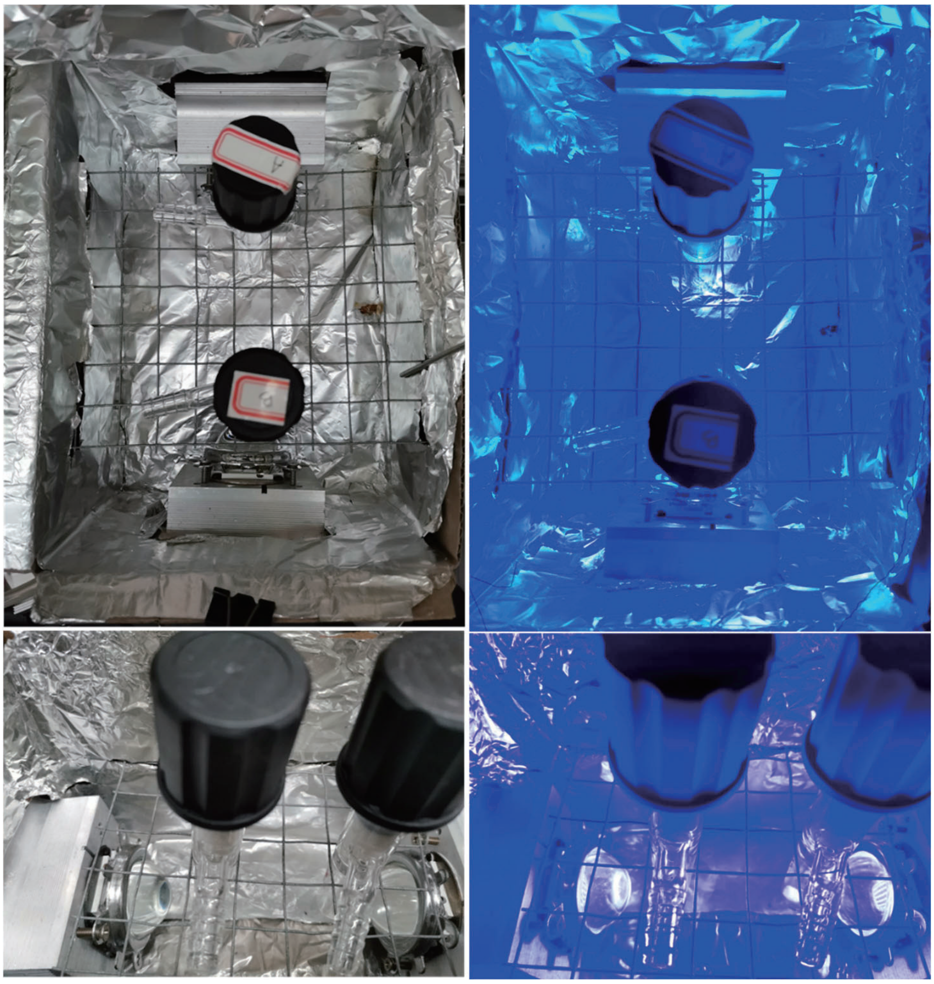

Supplementary Figure 1. The visible-light reaction apparatus for Typical Procedure II.

(2) 4,4-Hexamethylene-2,3-butadienenitrile **2b** (lq-6-135)

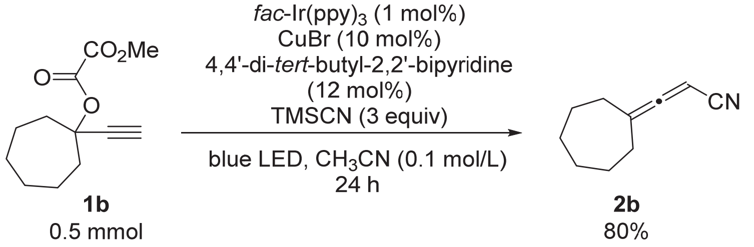

Following **Typical Procedure II**, the reaction of *fac*-Ir(ppy)3 (3.3 mg, 5 μmol), CuBr (7.3 mg, 0.05 mmol), 4,4'-di-*tert*-butyl-2,2'-bipyridine (16.4 mg, 0.06 mmol), **1b** (112.6 mg, 0.5 mmol)/CH_3CN(2.5 mL), and TMSCN (157.0 mg, 1.5 mmol)/CH_3CN(2.5 mL) afforded **2b** (59.3 mg, 80%) (eluent: petroleum ether/ethyl ether = 50:1) as a liquid: **^{1}H NMR** (400 MHz, $CDCl_3$) δ 5.10-4.97 (m, 1 H, =CH), 2.43-2.27 (m, 4 H), 1.77-1.47 (m, 8 H); **^{13}C NMR** (100 MHz, $CDCl_3$) δ 213.4, 114.6, 110.8, 64.4, 30.9, 29.2, 27.6; **MS** (EI) *m/z* (%) 147 (M^+, 17.52), 146 ($[M-H]^+$, 36.22), 81 (100); **IR** (neat): ν = 2924, 2853, 2221, 1952, 1638, 1443, 1351, 1326, 1267, 1217 cm^{-1}; **HRMS** (EI) calcd for $C_{10}H_{13}N$ (M^+): 147.1048, Found: 147.1052.

(3) 4,4-Heptamethylene-2,3-butadienenitrile **2c** (lq-6-143)

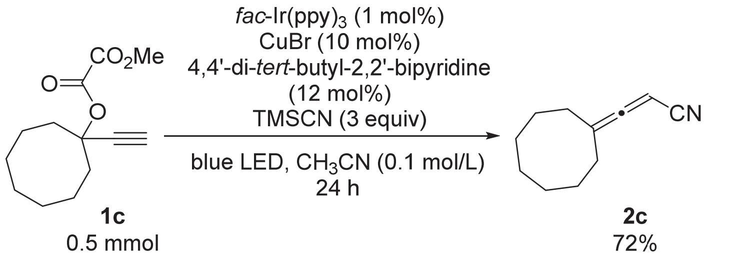

Following **Typical Procedure II**, the reaction of *fac*-Ir(ppy)3 (3.3 mg, 5 μmol), CuBr (7.3 mg, 0.05 mol), 4,4'-di-*tert*-butyl-2,2'-bipyridine (16.4 mg, 0.06 mmol), **1c** (119.2 mg, 0.5 mmol)/CH_3CN(2.5 mL), and TMSCN (157.0 mg, 1.5 mmol)/CH_3CN(2.5 mL) afforded **2c** (58.0 mg, 72%) (eluent: petroleum ether/ethyl ether = 50:1) as a liquid: **^{1}H NMR** (400 MHz, $CDCl_3$) δ 5.09-5.05 (m, 1 H, =CH), 2.34-2.21 (m, 4 H), 1.75-1.47 (m, 10 H); **^{13}C NMR** (100 MHz, $CDCl_3$) δ 213.4, 114.3, 111.3, 65.0, 30.2, 26.5, 26.2, 25.6; **MS** (EI) *m/z* (%) 161 (M^+, 5.16), 160 ($[M-H]^+$, 15.75), 67

(100); **IR** (neat): ν = 2922, 2852, 2221, 1950, 1636, 1467, 1445, 1359, 1328, 1227, 1156, 1018 cm^{-1}; **HRMS** (EI) calcd for $C_{11}H_{14}N$ ($[M-H]^+$): 160.1126, Found: 160.1131.

(4) 4,4-Undecamethylene-2,3-butadienenitrile **2d** (lq-6-148)

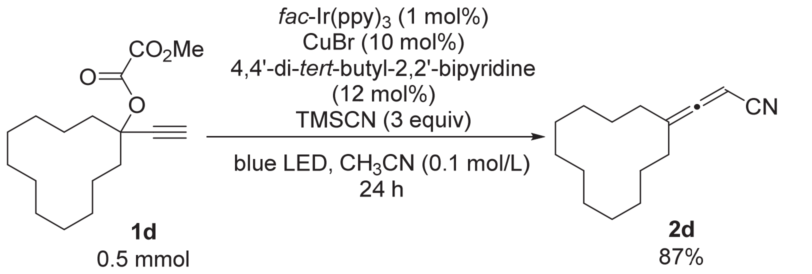

Following **Typical Procedure II**, the reaction of *fac*-Ir(ppy)$_3$ (3.3 mg, 5 μmol), CuBr (7.3 mg, 0.05 mmol), 4,4'-di-*tert*-butyl-2,2'-bipyridine (16.4 mg, 0.06 mmol), **1d** (147.2 mg, 0.5 mmol)/CH_3CN(2.5 mL), and TMSCN (156.8 mg, 1.5 mmol)/CH_3CN(2.5 mL) afforded **2d** (94.8 mg, 87%) (eluent: petroleum ether/ethyl ether = 50:1) as a liquid: **^{1}H NMR** (400 MHz, $CDCl_3$) δ 5.18-5.14 (m, 1 H, =CH), 2.20-2.09 (m, 4 H), 1.59-1.47 (m, 4 H), 1.45-1.20 (m, 14 H); **^{13}C NMR** (100 MHz, $CDCl_3$) δ 213.8, 114.2, 108.7, 66.8, 29.1, 24.3, 24.2, 23.7, 22.9, 21.9; **MS** (EI) *m/z* (%) 217 (M^+, 3.89), 216 ($[M-H]^+$, 19.24), 81 (100); **IR** (neat): ν = 2937, 2915, 2848, 2218, 1953, 1468, 1443, 1398, 1339, 1318, 1306, 1247 cm^{-1}; **HRMS** (EI) calcd for $C_{15}H_{23}N$ (M^+): 217.1830, Found: 217.1828.

(5) 3-(Tetrahydro-thiopyran-4-ylidene)acrylonitrile **2e** (lq-7-095)

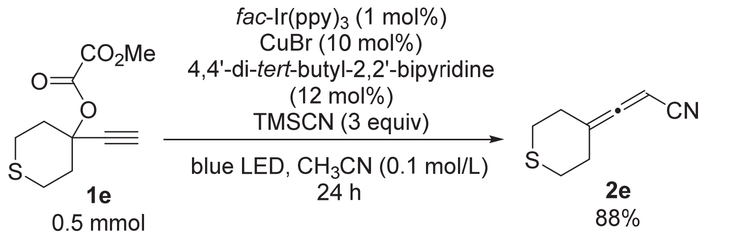

Following **Typical Procedure II**, the reaction of *fac*-Ir(ppy)$_3$ (3.3 mg, 5 μmol), CuBr (7.3 mg, 0.05 mmol), 4,4'-di-*tert*-butyl-2,2'-bipyridine (16.4 mg, 0.06 mmol), **1e** (114.3 mg, 0.5 mmol)/CH_3CN(2.5 mL), and TMSCN (156.8 mg, 1.5 mmol)/CH_3CN(2.5 mL) afforded **2e** (66.4 mg, 88%) (eluent: petroleum ether/ethyl ether = 20:1) as a white solid (m.p. 79.7-80.3 °C, *n*-hexane): **^{1}H NMR** (400 MHz,

$CDCl_3$) δ 5.18-5.12 (m, 1 H, =CH), 2.83-2.68 (m, 4 H, 2 × SCH_2), 2.62-2.48 (m, 4 H, 2 × CH_2); **^{13}C NMR** (100 MHz, $CDCl_3$) δ 210.9, 113.8, 106.5, 65.7, 30.9, 28.8; **MS** (EI) *m/z* (%) 151 (M^+, 100), 150 ($[M-H]^+$, 41.66); **IR** (neat): ν = 3010, 2951, 2919, 2220, 1963, 1425, 1327, 1308, 1271, 1242, 1194, 1116, 1018 cm^{-1}; Anal. Calcd. for C_8H_9NS: C 63.54, H 6.00, N, 9.26; Found: C 63.55, H 6.04, N, 9.29.

(6) 3-(1-Acetylpiperidin-4-ylidene)acrylonitrile **2f** (lq-6-161)

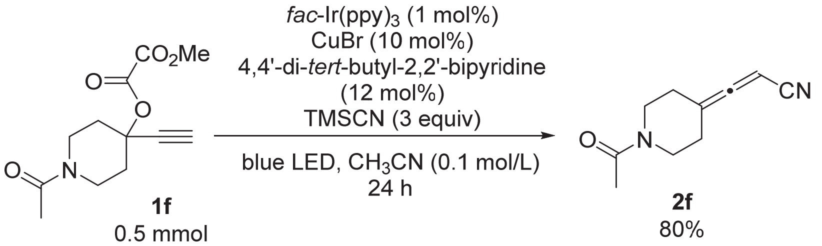

Following **Typical Procedure II**, the reaction of *fac*-Ir(ppy)$_3$ (3.3 mg, 5 μmol), CuBr (7.3 mg, 0.05 mmol), 4,4'-di-*tert*-butyl-2,2'-bipyridine (16.4 mg, 0.06 mmol), **1f** (126.8 mg, 0.5 mmol)/CH_3CN(2.5 mL), and TMSCN (156.8 mg, 1.5 mmol)/CH_3CN(2.5 mL) afforded **2f** (70.8 mg, 80%) (eluent: dichloromethane (5 mL) to dichloromethane/ethyl ether = 40:1 (~120 mL)) as a liquid: **^{1}H NMR** (400 MHz, $CDCl_3$) δ 5.24-5.18 (m, 1 H, =CH), 3.81-3.72 (m, 1 H, one proton of NCH_2), 3.71-3.63 (m, 1 H, one proton of NCH_2), 3.57 (t, *J* = 5.8 Hz, 2 H, two protons of 2 × NCH_2), 2.44-2.25 (m, 4 H, 2 × CH_2), 2.13 (s, 3 H, $COCH_3$); **^{13}C NMR** (100 MHz, $CDCl_3$) δ 210.5, 168.8, 113.6, 104.2, 66.5, 46.1, 41.3, 29.4, 28.6, 21.2; **MS** (EI) *m/z* (%) 176 (M^+, 6.31), 134 (100); **IR** (neat): ν = 3459, 3004, 2964, 2913, 2870, 2223, 1967, 1625, 1425, 1362, 1325, 1272, 1229, 1168, 1035 cm^{-1}; **HRMS** (EI) calcd for $C_{10}H_{12}N_2O$ (M^+): 176.0950, Found: 176.0955.

(7) 3-(1,4-Dioxaspiro[4.5]decan-8-ylidene)acrylonitrile **2g** (lq-7-018)

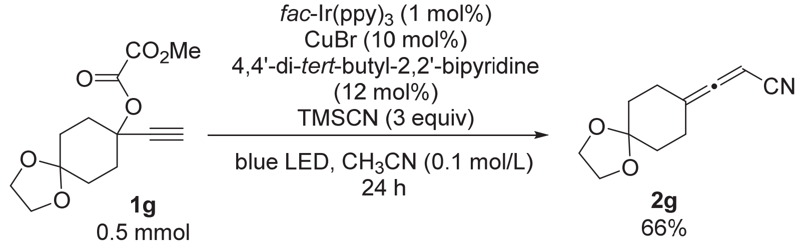

Following **Typical Procedure II**, the reaction of *fac*-Ir(ppy)$_3$ (3.3 mg, 5 μmol), CuBr (7.3 mg, 0.05 mmol), 4,4'-di-*tert*-butyl-2,2'-bipyridine (16.4 mg, 0.06 mmol), **1g** (134.3 mg, 0.5 mmol)/CH$_3$CN(2.5 mL), and TMSCN (157.1 mg, 1.5 mmol)/CH$_3$CN(2.5 mL) afforded **2g** (62.8 mg, 66%) (eluent: petroleum ether/ethyl ether = 20:1 (~42 mL) to 10:1 (55 mL)) as a white solid (m.p. 91.2-92.5 ^{o}C, *n*-hexane): **^{1}H NMR** (400 MHz, CDCl$_3$) δ 5.18-5.09 (m, 1 H, =CH), 3.97 (s, 4 H, 2 × OCH$_2$), 2.50-2.33 (m, 4 H, 2 × CH$_2$), 1.87-1.69 (m, 4 H, 2 × CH$_2$); **^{13}C NMR** (100 MHz, CDCl$_3$) δ 210.1, 114.3, 107.2, 106.0, 65.2, 64.4, 34.3, 27.1; **MS** (EI) *m/z* (%) 191 (M$^+$, 5.58), 86 (100); **IR** (neat): ν = 3026, 2999, 2960, 2886, 2220, 1960, 1477, 1440, 1407, 1360, 1336, 1318, 1275, 1237, 1202, 1166, 1112, 1074, 1031 cm^{-1}; Anal. Calcd. for $C_{11}H_{13}NO_2$: C 69.09, H 6.85, N 7.32; Found: C 69.10, H 6.98, N 7.10.

(8) 3-(4-((trimethylsilyl)oxy)cyclohex-3-enylidene)acrylonitrile **2h** (lq-7-019)

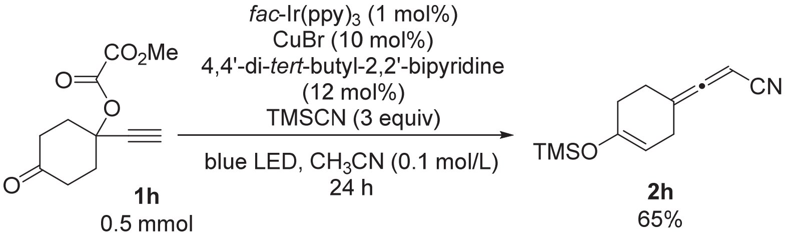

Following **Typical Procedure II**, the reaction of *fac*-Ir(ppy)$_3$ (3.3 mg, 5 μmol), CuBr (7.3 mg, 0.05 mmol), 4,4'-di-*tert*-butyl-2,2'-bipyridine (16.4 mg, 0.06 mmol), **1h** (112.4 mg, 0.5 mmol)/CH$_3$CN(2.5 mL), and TMSCN (156.8 mg, 1.5 mmol)/CH$_3$CN(2.5 mL) afforded **2g** (71.6 mg, 65%) (eluent: petroleum ether/ethyl ether = 20:1, it should be noted that the column packed with silica gel was eluted with a mixture of petroleum ether (10 mL) and two drops of Et$_3$N before loading the sample) as a liquid: **^{1}H NMR** (400 MHz, CDCl$_3$) δ 5.19-5.12 (m, 1 H, =CH), 4.87-4.80 (m, 1 H, =CH), 2.99-2.80 (m, 2 H, CH$_2$), 2.51-2.38 (m, 2 H, two protons of 2 × CH$_2$), 2.30-

2.14 (m, 2 H, two protons of 2 × CH_2), 0.20 (s, 9 H, 3 × CH_3); **^{13}C NMR** (100 MHz, $CDCl_3$) δ 210.7, 150.1, 114.3, 104.1, 100.5, 65.9, 29.7, 26.8, 26.5, 0.3; **MS** (EI) *m/z* (%) 219 (M^+, 18.46), 218 ($[M-H]^+$, 2.49), 91 (100); **IR** (neat): ν = 2959, 2925, 2841, 2223, 1966, 1667, 1433, 1410, 1365, 1315, 1252, 1218, 1194, 1175, 1044, 1016 cm^{-1}; **HRMS** (EI) calcd for $C_{12}H_{17}NOSi$ (M^+): 219.1079, Found: 219.1077.

(9) 4,6-Dimethylhepta-2,3-dienenitrile **2i** (lq-6-138)

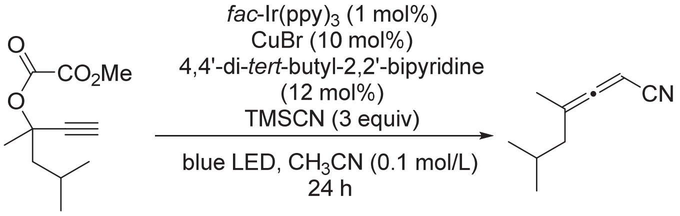

1i
0.5 mmol

2i
87%

Following **Typical Procedure II**, the reaction of *fac*-Ir(ppy)$_3$ (3.3 mg, 5 μmol), CuBr (7.3 mg, 0.05 mmol), 4,4'-di-*tert*-butyl-2,2'-bipyridine (16.4 mg, 0.06 mmol), **1i** (106.3 mg, 0.5 mmol)/CH_3CN(2.5 mL), and TMSCN (156.8 mg, 1.5 mmol)/CH_3CN(2.5 mL) afforded **2i**[11] (58.7 mg, 87%) (eluent: petroleum ether/ethyl ether = 50:1) as a liquid: **^{1}H NMR** (400 MHz, $CDCl_3$) δ 5.16-5.03 (m, 1 H, =CH), 1.95 (dd, J_1 = 7.0 Hz, J_2 = 2.6 Hz, 2 H, CH_2), 1.84-1.71 (m, 1 H, CH), 1.78 (d, *J* = 2.8 Hz, 3 H, CH_3), 0.93 (d, *J* = 6.8 Hz, 6 H, 2 × CH_3); **^{13}C NMR** (100 MHz, $CDCl_3$) δ 213.7, 114.3, 105.2, 65.6, 42.3, 26.1, 22.29, 22.27, 17.7; **MS** (EI) *m/z* (%) 135 (M^+, 100), 134 ($[M-H]^+$, 19.76); **IR** (neat): ν = 2957, 2929, 2899, 2872, 2223, 1961, 1466, 1386, 1369, 1285, 1251, 1205, 1168, 1085, 1005 cm^{-1}.

(10)4-Butylocta-2,3-dienenitrile **2j** (lq-8-028)

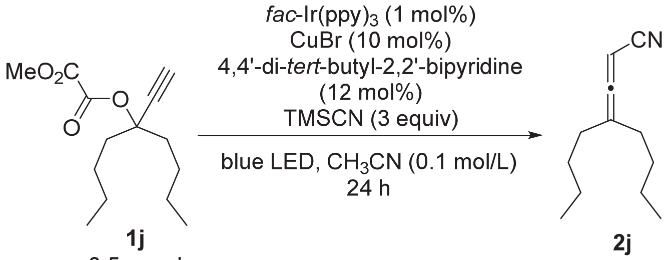

1j
0.5 mmol

2j
85%

Following **Typical Procedure II**, the reaction of *fac*-Ir(ppy)$_3$ (3.3 mg, 5 μmol),

CuBr (7.3 mg, 0.05 mmol), 4,4'-di-*tert*-butyl-2,2'-bipyridine (16.4 mg, 0.06 mmol), **1j** (127.6 mg, 0.5 mmol)/CH_3CN(2.5 mL), and TMSCN (157.0 mg, 1.5 mmol)/CH_3CN(2.5 mL) afforded **2j** (75.5 mg, 85%) (eluent: petroleum ether (100 mL) to petroleum ether/ethyl ether = 50:1 (~300 mL)) as a liquid: **^{1}H NMR** (400 MHz, $CDCl_3$) δ 5.21-5.12 (m, 1 H, =CH), 2.14-1.98 (m, 4 H, 2 × CH_2), 1.50-1.27 (m, 8 H, 4 × CH_2), 0.92 (t, *J* = 7.2 Hz, 6 H, 2 × CH_3); **^{13}C NMR** (100 MHz, $CDCl_3$) δ 213.2, 114.5, 111.4, 67.4, 31.3, 29.1, 22.2, 13.7; **MS** (EI) *m/z* (%) 177 (M^+, 0.81), 176 ($[M-H]^+$, 3.61), 93 (100); **IR** (neat): ν = 2958, 2929, 2861, 2223, 1956, 1465, 1398, 1379, 1330, 1300, 1257, 1230, 1152, 1107 cm^{-1}; **HRMS** (EI) calcd for $C_{12}H_{19}N$ (M^+): 177.1517, Found: 177.1516.

(11) Ethyl 7-cyano-5-methylhepta-5,6-dienoate **2k** (lq-7-077)

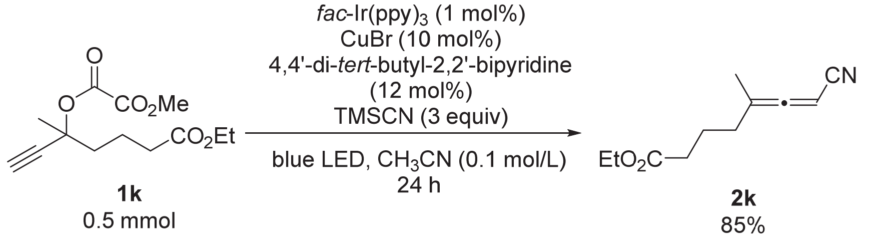

1k 0.5 mmol

2k 85%

Following **Typical Procedure II**, the reaction of *fac*-Ir(ppy)$_3$ (3.3 mg, 5 μmol), CuBr (7.3 mg, 0.05 mmol), 4,4'-di-*tert*-butyl-2,2'-bipyridine (16.4 mg, 0.06 mmol), **1k** (135.7 mg, 0.5 mmol)/CH_3CN(2.5 mL), and TMSCN (156.9 mg, 1.5 mmol)/CH_3CN(2.5 mL) afforded **2k** (82.1 mg, 85%) (eluent: petroleum ether/ethyl acetate = 50:1(~25 mL) to 20:1 (~84 mL)) as a liquid: **^{1}H NMR** (400 MHz, $CDCl_3$) δ 5.20-5.13 (m, 1 H, =CH), 4.14 (q, *J* = 7.1 Hz, 2 H, OCH_2), 2.34 (t, *J* = 7.4 Hz, 2 H, CH_2C=O), 2.19-2.06 (m, 2 H, CH_2), 1.86-1.72 (m, 2 H, CH_2 and CH_3), 1.27 (t, *J* = 7.2 Hz, 3 H, CH_3); **^{13}C NMR** (100 MHz, $CDCl_3$) δ 213.0, 172.9, 114.1, 105.8, 66.6, 60.3, 33.2, 32.1, 22.0, 17.5, 14.1; **MS** (EI) *m/z* (%) 193 (M^+, 2.07), 79 (100); **IR** (neat): ν = 2983, 2938, 2223, 1961, 1729, 1447, 1420, 1393, 1373, 1337, 1313, 1181, 1096, 1028 cm^{-1}; **HRMS** (EI) calcd for $C_{11}H_{15}NO_2$ (M^+): 193.1103, Found: 193.1099.

(12) 4-(Adamantan-1-yl)penta-2,3-dienenitrile **2l** (lq-8-087)

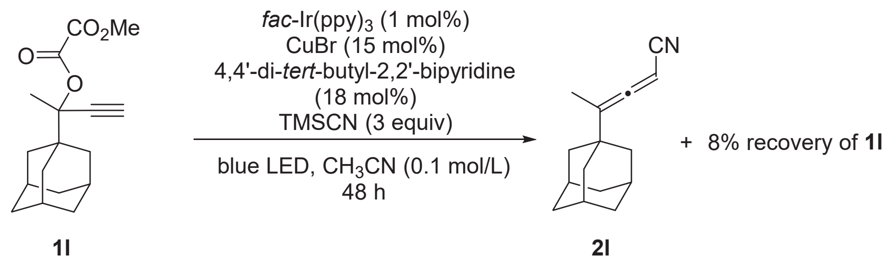

Following **Typical Procedure II**, the reaction of *fac*-Ir(ppy)$_3$ (3.3 mg, 5 μmol), CuBr (11.0 mg, 0.075 mmol), 4,4'-di-*tert*-butyl-2,2'-bipyridine (24.6 mg, 0.09 mmol), **1l** (145.3 mg, 0.5 mmol)/CH_3CN(2.5 mL), and TMSCN (157.2 mg, 1.5 mmol)/CH_3CN(2.5 mL) afforded **2l**. The recovery of **1l** (8%) was determined by ^{1}H NMR analysis of the crude product using CH_3NO_2 as the internal standard. **2l** (97.2 mg, 91%) (eluent: petroleum ether (100 mL) to petroleum ether/ethyl ether = 50:1 (~300 mL)) as a liquid: **^{1}H NMR** (400 MHz, $CDCl_3$) δ 5.13 (q, *J* = 2.9 Hz, 1 H, =CH), 2.10-1.96 (m, 3 H), 1.80-1.60 (m, 15 H); **^{13}C NMR** (100 MHz, $CDCl_3$) δ 213.4, 115.4, 114.8, 66.6, 40.6, 36.5, 35.7, 28.4, 12.5; **MS** (EI) *m/z* (%) 213 (M^+, 1.7), 135 (100); **IR** (neat): ν = 2996, 2911, 2848, 2216, 1949, 1454, 1358, 1343, 1312, 1263, 1212, 1106, 1055 cm^{-1}; **HRMS** (EI) calcd for $C_{15}H_{19}N$ (M^+): 213.1517, Found: 213.1516.

(13) 4-Methyl-6-phenylhexa-2,3-dienenitrile **2m** (lq-6-142)

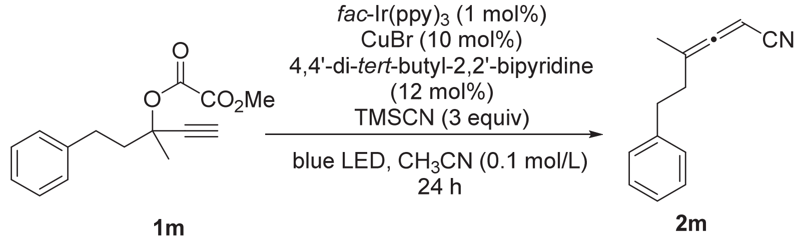

Following **Typical Procedure II**, the reaction of *fac*-Ir(ppy)$_3$ (3.3 mg, 5 μmol), CuBr (7.3 mg, 0.05 mmol), 4,4'-di-*tert*-butyl-2,2'-bipyridine (16.4 mg, 0.06 mmol), **1m** (130.3 mg, 0.5 mmol)/CH_3CN(2.5 mL), and TMSCN (156.8 mg, 1.5 mmol)/CH_3CN(2.5 mL) afforded **2m** (79.8 mg, 87%) (eluent: petroleum ether/ethyl ether = 50:1) as a liquid: **^{1}H NMR** (400 MHz, $CDCl_3$) δ 7.30 (t, *J* = 7.4 Hz, 2 H, ArH), 7.24-7.13 (m, 3 H, ArH), 5.13-5.04 (m, 1 H, =CH), 2.76 (t, *J* = 8.0 Hz, 2 H, CH_2), 2.45-2.27 (m, 2 H, CH_2), 1.81 (d, *J* = 2.8 Hz, 3 H, CH_3); **^{13}C NMR** (100 MHz, $CDCl_3$) δ

213.4, 140.5, 128.5, 128.2, 126.2, 114.1, 106.0, 66.7, 34.5, 33.2, 17.8; **MS** (EI) *m/z* (%) 183 (M^+, 16.42), 182 ($[M-H]^+$, 60.35), 91 (100); **IR** (neat): ν = 3027, 2921, 2859, 2222, 1960, 1603, 1496, 1453, 1391, 1371, 1216, 1077, 1030 cm^{-1}; **HRMS** (EI) calcd for $C_{13}H_{13}N$ (M^+): 183.1048, Found: 183.1048.

(14) 5-(4-Fluorophenyl)-4-methylpenta-2,3-dienenitrile **2n** (lq-7-025)

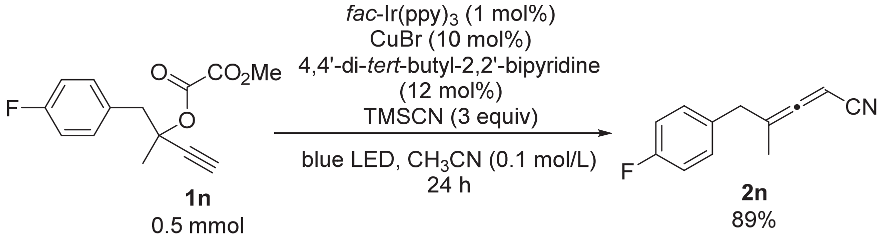

Following **Typical Procedure II**, the reaction of *fac*-Ir(ppy)$_3$ (3.3 mg, 5 μmol), CuBr (7.3 mg, 0.05 mmol), 4,4'-di-*tert*-butyl-2,2'-bipyridine (16.4 mg, 0.06 mmol), **1n** (132.4 mg, 0.5 mmol)/CH_3CN(2.5 mL), and TMSCN (156.9 mg, 1.5 mmol)/CH_3CN(2.5 mL) afforded **2n** (83.2 mg, 89%) (eluent: petroleum ether/ethyl ether = 50:1 (~50 mL) to 20:1 (~50 mL)) as a liquid: **^{1}H NMR** (400 MHz, $CDCl_3$) δ 7.15 (dd, J_1 = 8.6 Hz, J_2 = 5.4 Hz, 2 H, ArH), 7.01 (t, J = 8.6 Hz, 2 H, ArH), 5.12-5.06 (m, 1 H, =CH), 3.40-3.29 (m, 2 H, CH_2), 1.77 (d, J = 3.2 Hz, 3 H, CH_3); **^{13}C NMR** (100 MHz, $CDCl_3$) δ 213.7, 161.8 (d, J = 243.7 Hz), 132.5 (d, J = 3.2 Hz), 130.3 (d, J = 8.1 Hz), 115.4 (d, J = 21.0 Hz), 113.9, 106.3, 66.7, 38.9, 17.2; **^{19}F NMR** (376 MHz, $CDCl_3$) δ -116.1; **MS** (EI) *m/z* (%) 187 (M^+, 24.56), 186 ($[M-H]^+$, 37.55), 109 (100); **IR** (neat): ν = 3013, 2954, 2922, 2223, 1964, 1602, 1508, 1443, 1416, 1393, 1372, 1220, 1157, 1093, 1015 cm^{-1}; **HRMS** (EI) calcd for $C_{12}H_{10}NF$ (M^+): 187.0797, Found: 187.0799.

(15) 5-(4-Chlorophenyl)-4-methylpenta-2,3-dienenitrile **2o** (lq-6-178)

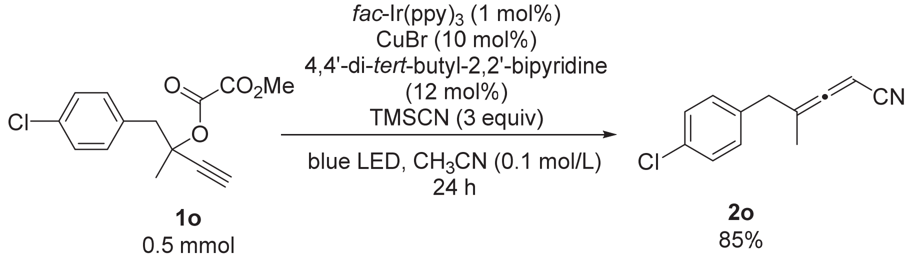

Following **Typical Procedure II**, the reaction of *fac*-Ir(ppy)$_3$ (3.3 mg, 5 μmol),

CuBr (7.3 mg, 0.05 mmol), 4,4'-di-*tert*-butyl-2,2'-bipyridine (16.4 mg, 0.06 mmol), **1o** (141.0 mg, 0.5 mmol)/CH_3CN(2.5 mL), and TMSCN (156.7 mg, 1.5 mmol)/CH_3CN(2.5 mL) afforded **2o** (87.3 mg, 85%) (eluent: petroleum ether/ethyl ether = 50:1 (~50 mL) to 20:1 (~50 mL)) as a liquid: **^{1}H NMR** (400 MHz, $CDCl_3$) δ 7.29 (d, J = 8.4 Hz, 2 H, ArH), 7.11 (d, J = 8.4 Hz, 2 H, ArH), 5.18-5.04 (m, 1 H, =CH), 3.41-3.27 (m, 2 H, CH_2), 1.77 (d, J = 3.2 Hz, 3 H, CH_3); **^{13}C NMR** (100 MHz, $CDCl_3$) δ 213.7, 135.3, 132.8, 130.2, 128.7, 113.8, 106.0, 66.7, 39.0, 17.2; **MS** (EI) *m/z* (%) 205 (M(^{37}Cl)$^+$, 2.02), 203 (M(^{35}Cl)$^+$, 4.49), 173 (100); **IR** (neat): ν = 3012, 2952, 2919, 2223, 1964, 1597, 1491, 1440, 1406, 1393, 1371, 1226, 1198, 1178, 1089, 1015 cm^{-1}; **HRMS** (EI) calcd for $C_{12}H_{10}NCl$ (M(^{35}Cl)$^+$): 203.0502, Found: 203.0505.

(16) 5-(4-Bromophenyl)-4-methylpenta-2,3-dienenitrile **2p** (lq-7-033)

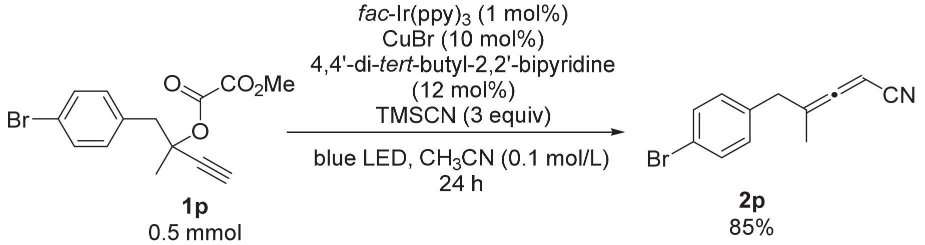

Following **Typical Procedure II**, the reaction of *fac*-Ir(ppy)$_3$ (3.3 mg, 5 μmol), CuBr (7.3 mg, 0.05 mmol), 4,4'-di-*tert*-butyl-2,2'-bipyridine (16.4 mg, 0.06 mmol), **1p** (162.8 mg, 0.5 mmol)/CH_3CN (2.5 mL), and TMSCN (156.8 mg, 1.5 mmol)/CH_3CN(2.5 mL) afforded **2p** (105.5 mg, 85%) (eluent: petroleum ether/ethyl ether = 20:1) as a liquid: **^{1}H NMR** (400 MHz, $CDCl_3$) δ 7.44 (d, J = 8.4 Hz, 2 H, ArH), 7.06 (d, J = 8.4 Hz, 2 H, ArH), 5.13-5.08 (m, 1 H, =CH), 3.39-3.27 (m, 2 H, CH_2), 1.77 (d, J = 3.2 Hz, 3 H, CH_3); **^{13}C NMR** (100 MHz, $CDCl_3$) δ 213.7, 135.8, 131.7, 130.5, 120.9, 113.8, 105.9, 66.8, 39.1, 17.2; **MS** (EI) *m/z* (%) 249 (M(^{81}Br)$^+$, 1.04), 247 (M(^{79}Br)$^+$, 1.05), 168 (100); **IR** (neat): ν = 3011, 2951, 2917, 2222, 1964, 1591, 1487, 1440, 1403, 1371, 1309, 1226, 1197, 1179, 1144, 1100, 1070, 1011 cm^{-1}; **HRMS** (EI) calcd for $C_{12}H_{10}N^{79}Br$ (M(^{79}Br)$^+$): 246.9997, Found: 247.0001.

(17) 5-Cyano-3-methylpenta-3,4-dienyl 4-ethynylbenzoate **2q** (lq-8-048)

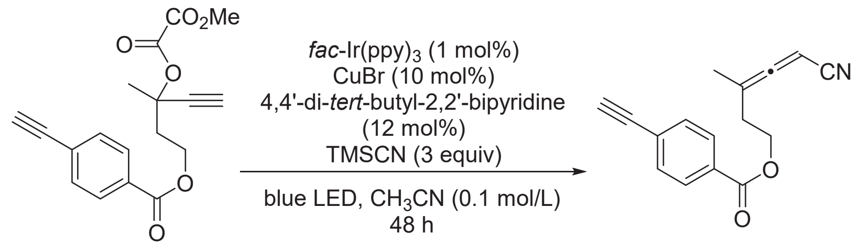

1q 0.5 mmol

2q 91%

Following **Typical Procedure II**, the reaction of *fac*-Ir(ppy)3 (3.3 mg, 5 μmol), CuBr (7.3 mg, 0.05 mmol), 4,4'-di-*tert*-butyl-2,2'-bipyridine (16.4 mg, 0.06 mmol), **1q** (164.5 mg, 0.5 mmol)/CH_3CN(2.5 mL), and TMSCN (157.1 mg, 1.5 mmol)/CH_3CN(2.5 mL) afforded **2q** (114.0 mg, 91%) (using Biotage Isolera One purification system on flash silica gel column (Santai Tech. Inc., 12 g), flowrate: 25 mL/min, eluent: petroleum ether (40 mL), petroleum ether to petroleum ether/ethyl acetate = 95:5 (linear gradient, 200 mL), 95:5 (100 mL), 95:5 to 91:9 (linear gradient, 200 mL), 91:9 (40 mL)) as a liquid: **^{1}H NMR** (400 MHz, $CDCl_3$) δ 8.00 (d, *J* = 8.4 Hz, 2 H, ArH), 7.56 (d, *J* = 8.0 Hz, 2 H, ArH), 5.24-5.14 (m, 1 H, =CH), 4.51-4.37 (m, 2 H, OCH_2), 3.24 (s, 1 H, C≡CH), 2.63-2.47 (m, 2 H, CH_2), 1.89 (d, *J* = 2.8 Hz, 3 H, CH_3); **^{13}C NMR** (100 MHz, $CDCl_3$) δ 213.2, 165.6, 132.1, 129.7, 129.5, 127.0, 113.7, 103.0, 82.7, 80.2, 67.1, 61.8, 32.3, 17.8; **MS** (EI) *m/z* (%) 251 (M^+, 4.32), 129 (100); **IR** (neat): ν = 3283, 2960, 2359, 2223, 1963, 1607, 1562, 1450, 1404, 1378, 1307, 1173, 1018, 859, 767, 695 cm^{-1}; **HRMS** (EI) calcd for $C_{16}H_{13}NO_2$ (M^+): 251.0941, Found: 251.0937.

Gram-scale synthesis of 5-cyano-3-methylpenta-3,4-dienyl 4-ethynylbenzoate **2q** (lq-8-049)

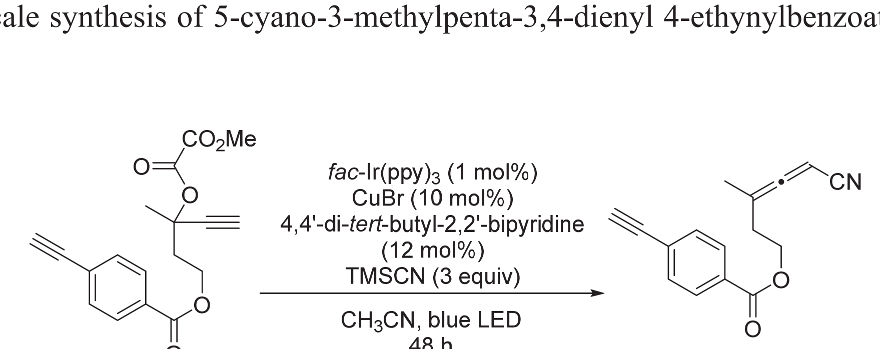

1q 5 mmol

2q 84% (1.0530 g)

To a flame-dried 100 mL Schlenk tube were added *fac*-Ir(ppy)3 (33.2 mg, 0.05 mmol), CuBr (73.5 mg, 0.5 mmol), 4,4'-di-*tert*-butyl-2,2'-bipyridine (164.4 mg, 0.6

mmol), **1q** (1.6418 g, 5 mmol)/CH_3CN(25 mL), and TMSCN (1.5680 g, 15 mmol)/CH_3CN(25 mL) sequentially under Ar atmosphere. The resulting mixture was irradiated with three 50 W 460 nm blue LED lamps (3-4 cm away, with a cooling fan to keep the reaction temperature at 35~45 ºC) for 48 h with stirring in a water bath without heating and monitored by TLC. The resulting mixture was filtrated through a short pad of silica gel eluted with ethyl ether (100 mL). After evaporation, the residue was purified by using Biotage Isolera One purification system on flash silica gel column (Santai Tech. Inc., 25 g), flowrate: 30 mL/min, eluent: petroleum ether (64 mL), petroleum ether to petroleum ether/ethyl acetate = 95:5 (linear gradient, 320 mL), 95:5 (320 mL), 95:5 to 91:9 (linear gradient, 320 mL)) to afford **2q** (1.0530 g, 84%) as a liquid: **^{1}H NMR** (400 MHz, $CDCl_3$) δ 8.00 (d, J = 8.4 Hz, 2 H, ArH), 7.56 (d, J = 8.0 Hz, 2 H, ArH), 5.22-5.14 (m, 1 H, =CH), 4.51-4.38 (m, 2 H, OCH_2), 3.25 (s, 1 H, C≡CH), 2.62-2.46 (m, 2 H, CH_2), 1.88 (d, J = 2.8 Hz, 3 H, CH_3); **^{13}C NMR** (100 MHz, $CDCl_3$) δ 213.2, 165.6, 132.1, 129.7, 129.4, 127.0, 113.7, 103.0, 82.7, 80.2, 67.1, 61.7, 32.3, 17.7.

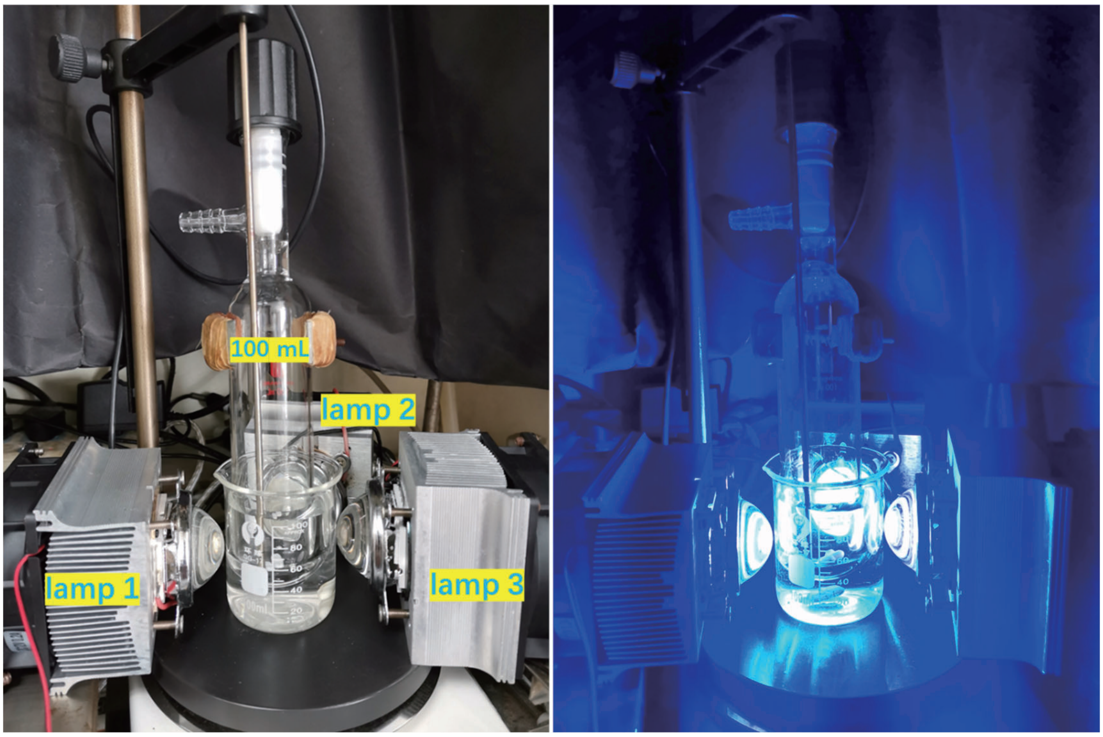

Supplementary Figure 2. The visible-light reaction apparatus used for large scale reactions (three lamps were used.).

(18) 6-(4-(Allyloxy)phenyl)-4-methylhexa-2,3-dienenitrile **2r** (lq-8-086)

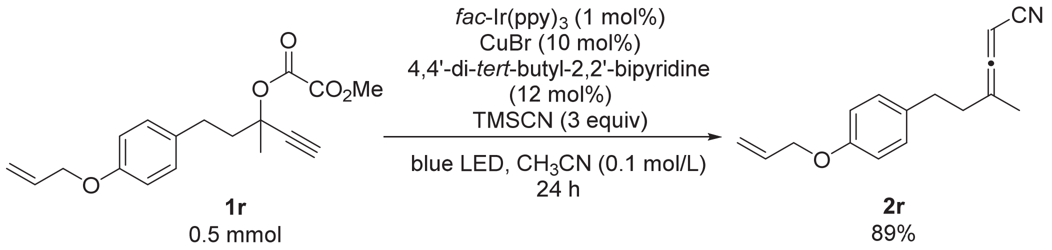

Following **Typical Procedure II**, the reaction of *fac*-Ir(ppy)$_3$ (3.3 mg, 5 μmol), CuBr (7.3 mg, 0.05 mmol), 4,4'-di-*tert*-butyl-2,2'-bipyridine (16.4 mg, 0.06 mmol), **1r** (158.0 mg, 0.5 mmol)/CH_3CN(2.5 mL), and TMSCN (156.8 mg, 1.5 mmol)/CH_3CN(2.5 mL) afforded **2r** (106.8 mg, 89%) (using Biotage Isolera One purification system on flash silica gel column (Santai Tech. Inc., 12 g), flowrate: 25 mL/min, eluent: petroleum ether (40 mL), petroleum ether to petroleum ether/ethyl acetate = 90:10 (linear gradient, 200 mL), 90:10 (120 mL)) as a liquid: **^{1}H NMR** (400 MHz, $CDCl_3$) δ 7.08 (d, J = 8.8 Hz, 2 H, ArH), 6.85 (d, J = 8.4 Hz, 2 H, ArH), 6.14-5.97 (m, 1 H, =CH), 5.40 (dd, J_1 = 17.2 Hz, J_2 = 1.6 Hz, 1 H, =CH), 5.33-5.21 (m, 1 H, =CH), 5.14-.5.02 (m, 1 H, =CH), 4.51 (d, J = 5.2 Hz, 2 H, OCH_2), 2.69 (t, J = 7.8 Hz, 2 H, CH_2), 2.44-2.23 (m, 2 H, CH_2), 1.80 (d, J = 2.8 Hz, 3 H, CH_3); **^{13}C NMR** (100 MHz, $CDCl_3$) δ 213.4, 157.0, 133.3, 132.8, 129.1, 117.5, 114.7, 114.2, 105.9, 68.8, 66.6, 34.7, 32.3, 17.8; **MS** (EI) *m/z* (%) 239 (M^+, 37.78), 238 ($[M-H]^+$, 36.87), 224 ($[M-CH_3]^+$, 17.59), 198 (100); **IR** (neat): ν = 3007, 2923, 2858, 2222, 1961, 1648, 1611, 1583, 1509, 1455, 1426, 1409, 1393, 1370, 1299, 1239, 1221, 1177, 1154, 1108, 1021 cm^{-1}; **HRMS** (EI) calcd for $C_{16}H_{17}NO$ (M^+): 239.1305, Found: 239.1301.

(19) 6-(Benzo[d][1,3]dioxol-5-yl)-4-methylhexa-2,3-dienenitrile **2s** (lq-7-051)

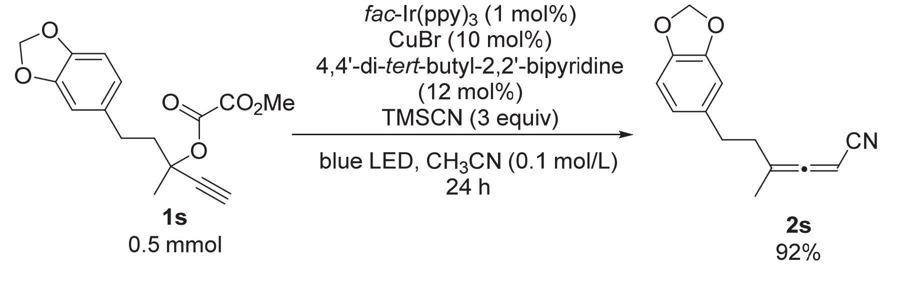

Following **Typical Procedure II**, the reaction of *fac*-Ir(ppy)$_3$ (3.3 mg, 5 μmol), CuBr (7.3 mg, 0.05 mmol), 4,4'-di-*tert*-butyl-2,2'-bipyridine (16.4 mg, 0.06 mmol), **1s** (152.2 mg, 0.5 mmol)/CH_3CN(2.5 mL), and TMSCN (157.2 mg, 1.5 mmol)/CH_3CN(2.5 mL) afforded **2s** (104.8 mg, 92%) (eluent: petroleum ether/ethyl acetate = 20:1) as a liquid: **^{1}H NMR** (400 MHz, $CDCl_3$) δ 6.73 (d, J = 7.6 Hz, 1 H, ArH), 6.66 (d, J = 1.2 Hz, 1 H, ArH), 6.62 (dd, J_1 = 8.0 Hz, J_2 = 1.6 Hz, 1 H, ArH), 5.92 (s, 2 H, OCH_2O), 5.13-5.08 (m, 1 H, =CH), 2.72-2.63 (m, 2 H, CH_2), 2.32-2.27 (m, 2 H, CH_2), 1.81 (d, J = 3.2 Hz, 3 H, CH_3); **^{13}C NMR** (100 MHz, $CDCl_3$) δ 213.3, 147.6, 145.9, 134.3, 121.0, 114.1, 108.6, 108.2, 105.8, 100.8, 66.7, 34.7, 32.9, 17.8; **MS** (EI) *m/z* (%) 227 (M^+, 23.93), 226 ($[M-H]^+$, 8.83), 135 (100); **IR** (neat): ν = 3012, 2896, 2777, 2222, 1961, 1608, 1503, 1488, 1441, 1393, 1366, 1243, 1188, 1121, 1097, 1036 cm^{-1}; **HRMS** (EI) calcd for $C_{14}H_{13}NO_2$ (M^+): 227.0946, Found: 227.0948.

(20) 4-Methyl-5-(thiophen-3-yl)penta-2,3-dienenitrile **2t** (lq-7-092)

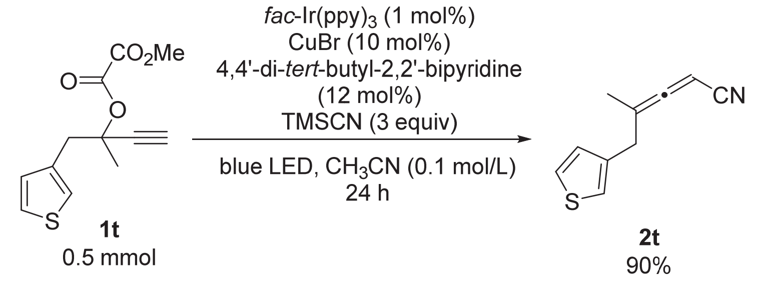

Following **Typical Procedure II**, the reaction of *fac*-Ir(ppy)$_3$ (3.3 mg, 5 μmol), CuBr (7.3 mg, 0.05 mmol), 4,4'-di-*tert*-butyl-2,2'-bipyridine (16.4 mg, 0.06 mmol), **1t** (126.4 mg, 0.5 mmol)/CH_3CN(2.5 mL), and TMSCN (157.2 mg, 1.5 mmol)/CH_3CN(2.5 mL) afforded **2t** (79.2 mg, 90%) (eluent: petroleum ether/ethyl ether = 50:1) as a liquid: **^{1}H NMR** (400 MHz, $CDCl_3$) δ 7.28 (dd, J_1 = 5.0 Hz, J_2 = 3.0 Hz, 1 H, ArH), 7.06-7.01 (m, 1 H, ArH), 6.93 (dd, J_1 = 4.8 Hz, J_2 = 1.2 Hz, 1 H, ArH), 5.13-5.07 (m, 1 H, =CH), 3.40 (d, J = 2.0 Hz, 2 H, CH_2), 1.78 (d, J = 3.2 Hz, 3 H, CH_3); **^{13}C NMR** (100 MHz, $CDCl_3$) δ 213.7, 137.0, 128.1, 126.0, 122.3, 114.0, 105.9, 66.5, 34.1, 17.2; **MS** (EI) *m/z* (%) 175 (M^+, 21.87), 174 ($[M-H]^+$, 16.07), 160 ($[M-Me]^+$, 28.44), 97 (100); **IR** (neat): ν = 3101, 3008, 2951, 2919, 2222, 1964, 1569, 1536, 1439, 1413, 1393, 1370, 1295, 1247, 1216, 1152, 1080, 1009, 759, 692, 637, 589 cm^{-1};

HRMS (EI) calcd for $C_{10}H_9NS$ (M^+): 175.0450, Found: 175.0448.

(21) **2u** (lq-7-119)

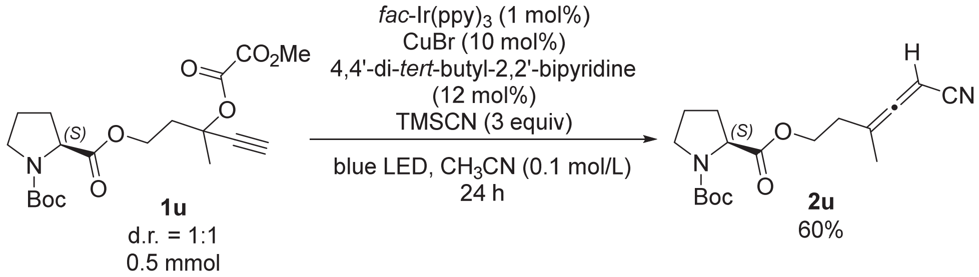

Following **Typical Procedure II**, the reaction of *fac*-Ir(ppy)$_3$ (3.3 mg, 5 μmol), CuBr (7.3 mg, 0.05 mmol), 4,4'-di-*tert*-butyl-2,2'-bipyridine (16.4 mg, 0.06 mmol), **1u** (198.8 mg, 0.5 mmol, d.r. = 1:1)/CH_3CN(2.5 mL), and TMSCN (156.7 mg, 1.5 mmol)/CH_3CN(2.5 mL) afforded **2u** (96.8 mg, 60%) (eluent: dichloromethane:ethyl ether = 40:1) as a liquid: **^{1}H NMR** (400 MHz, $CDCl_3$, a mixture of rotamers) δ 5.32-5.18 (m, 1 H, =CH), 4.35-4.14 (m, 3 H, NCH and OCH_2), 3.61-3.32 (m, 2 H, NCH_2), 2.52-2.35 (m, 2 H), 2.34-2.15 (m, 1 H), 2.04-1.88 (m, 3 H), 1.85 (s, 3 H, CH_3), [1.46 (s, 4.2 H), 1.42 (s, 4.98 H), 9 H, $OC(CH_3)_3$]; **MS** (ESI) *m/z* 343 ([M+Na]$^+$), 359 ([M+K]$^+$); **IR** (neat): ν = 2976, 2881, 2223, 1963, 1745, 1692, 1478, 1453, 1392, 1365, 1277, 1257, 1156, 1120, 1087, 1036 cm^{-1}; Anal. Calcd. for $C_{17}H_{24}N_2O_4$: C 63.73, H 7.55, N, 8.74; Found: C 63.27, H 7.60, N, 8.58.

Synthesis of **2u** on 1.1 mmol scale (lq-7-155)

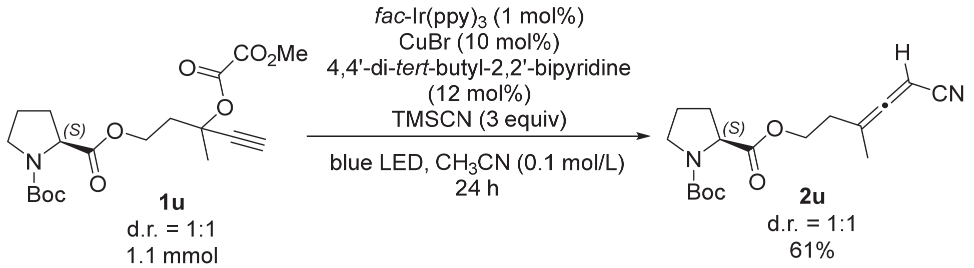

Following **Typical Procedure II**, the reaction of *fac*-Ir(ppy)$_3$ (7.3 mg, 11 μmol), CuBr (16.1 mg, 0.11 mmol), 4,4'-di-*tert*-butyl-2,2'-bipyridine (36.2 mg, 0.132 mmol), **1u** (438.2 mg, 1.1 mmol, d.r. = 1:1)/CH_3CN(5 mL), and TMSCN (345.0 mg, 3.3 mmol)/CH_3CN(5 mL) afforded **2u** (215.0 mg, 61%, d.r. = 1:1) (eluent: dichloromethane

(100 mL) to dichloromethane:ethyl ether = 40:1 (~500 mL)) as a liquid: The d.r. ratio of **2u** was determined to 1:1 by HPLC (HPLC conditions: Chiralpak AD-H column, hexane/*i*-PrOH = 90/10, 1.0 mL/min, λ = 214 nm, t_{R1} = 10.6 min, t_{R2} = 12.1 min); **^{1}H NMR** (400 MHz, $CDCl_3$, a mixture of rotamers) δ 5.28-5.17 (m, 1 H, =CH), 4.35-4.16 (m, 3 H, NCH and OCH_2), 3.60-3.32 (m, 2 H, NCH_2), 2.51-2.35 (m, 2 H), 2.33-2.15 (m, 1 H), 2.02-1.87 (m, 3 H), 1.85 (s, 3 H, CH_3), [1.46 (s, 4.3 H), 1.42 (s, 5.03 H), 9 H, $OC(CH_3)_3$].

(22) **2v** (lq-7-144)

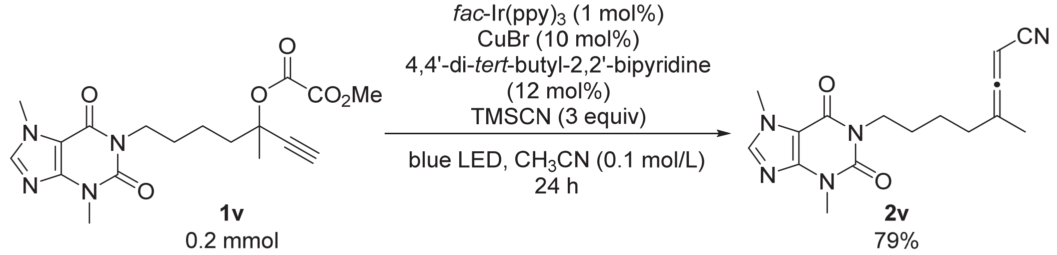

Following **Typical Procedure II**, the reaction of *fac*-Ir(ppy)$_3$ (1.3 mg, 2 μmol), CuBr (2.9 mg, 0.02 mmol), 4,4'-di-*tert*-butyl-2,2'-bipyridine (6.6 mg, 0.024 mmol), **1v** (78.0 mg, 0.2 mmol)/CH_3CN(2.5 mL), and TMSCN (77 μL, d = 0.793 g/mL, 61.1 mg, 0.6 mmol)/CH_3CN(2.5 mL) afforded **2v** (49.2 mg, 79%) (eluent: petroleum ether (50 mL) to petroleum ether/ethyl acetate = 2:1 (90 mL) to 1:1 (200 mL) to dichlromethane:MeOH = 50:1 (~200 mL)) as a liquid: **^{1}H NMR** (400 MHz, $CDCl_3$) δ 7.51 (s, 1 H, CH), 5.17-5.11 (m, 1 H, =CH), 4.02 (t, J = 7.4 Hz, 2 H, NCH_2), 3.99 (s, 3 H, NCH_3), 3.68 (s, 3 H, NCH_3), 2.18-2.07 (m, 2 H, CH_2), 1.79 (d, J = 2.8 Hz, 3 H, CH_3), 1.76-1.63 (m, 2 H, CH_2), 1.59-1.47 (m, 2 H, CH_2); **^{13}C NMR** (100 MHz, $CDCl_3$) δ 213.2, 155.1, 151.3, 148.6, 141.4, 114.2, 107.5, 106.1, 66.3, 40.7, 33.4, 32.4, 29.5, 27.3, 24.1, 17.5; **MS** (ESI) *m/z* 314 ($[M+H]^+$), 336 ($[M+Na]^+$); **IR** (neat): ν = 3516, 3114, 2946, 2222, 1960, 1699, 1650, 1605, 1548, 1484, 1453, 1357, 1323, 1286, 1232, 1187, 1122, 1046 cm^{-1}; **HRMS** (ESI) calcd for $C_{16}H_{20}O_2N_5$ ($[M+H]^+$): 314.1612, Found: 314.1603.

(23) **2w** and **2w'** (lq-8-110)

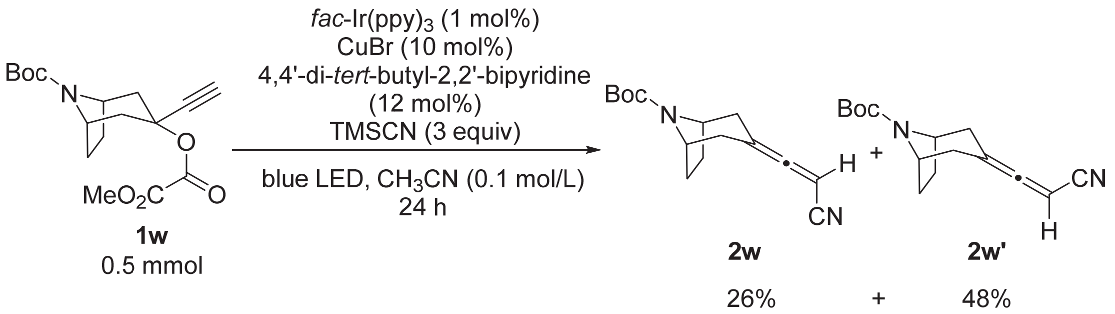

Following **Typical Procedure II**, the reaction of *fac*-Ir(ppy)3 (3.3 mg, 5 μmol), CuBr (7.3 mg, 0.05 mmol), 4,4'-di-*tert*-butyl-2,2'-bipyridine (16.4 mg, 0.06 mmol), **1w** (168.8 mg, 0.5 mmol)/CH_3CN(2.5 mL), and TMSCN (156.9 mg, 1.5 mmol)/CH_3CN(2.5 mL) afforded **2w** (34.2 mg, 26%, less polar) and **2w'** (62.0 mg, 48%, more polar) (using Biotage Isolera One purification system on flash silica gel column (Santai Tech. Inc., 25 g), flowrate: 30 mL/min, eluent: petroleum ether (96 mL), petroleum ether to petroleum ether/ethyl acetate = 92:8 (linear gradient, 480 mL), 92:8 (344 mL)).

2w: a white solid (m.p. 99.7-101.4 ºC, *n*-hexane): **^{1}H NMR** (400 MHz, $CDCl_3$) δ 5.13 (t, *J* = 4.8 Hz, 1 H, =CH), 4.51-4.20 (m, 2 H, 2 × NCH), 2.69-2.38 (m, 2 H), 2.25-2.13 (m, 2 H), 2.10-1.91 (m, 2 H), 1.85-1.69 (m, 2 H), 1.48 (s, 9 H, $OC(CH_3)_3$); **^{13}C NMR** (100 MHz, $CDCl_3$) δ 213.0, 153.2, 114.0, 102.5, 79.8, 65.5, 53.6, 53.0, 35.2, 34.5, 28.6, 28.4, 27.9; **MS** (ESI) *m/z* 283 ($[M+Na]^+$); **IR** (neat): ν = 2973, 2949, 2923, 2221, 1966, 1685, 1481, 1471, 1456, 1409, 1397, 1368, 1343, 1323, 1291, 1250, 1211, 1177, 1151, 1118, 1101, 1055, 1044, 1006 cm^{-1}; **HRMS** (ESI) calcd for $C_{15}H_{20}O_2N_2Na$ ($[M+Na]^+$): 283.1417, Found: 283.1416.

2w': a white solid (m.p. 126.7-127.2 ºC, *n*-hexane): **^{1}H NMR** (400 MHz, $CDCl_3$) δ **^{1}H NMR** (400 MHz, $CDCl_3$) δ 5.20 (t, *J* = 4.4 Hz, 1 H, =CH), 4.53-4.16 (m, 2 H, 2 × NCH), 2.75-2.40 (m, 2 H), 2.22-2.10 (m, 2 H), 2.07-1.90 (m, 2 H), 1.71-1.58 (m, 2 H), 1.49 (s, 9 H, $OC(CH_3)_3$); **^{13}C NMR** (100 MHz, $CDCl_3$) δ 212.8, 153.1, 113.7, 102.5, 79.9, 65.7, 53.4, 52.8, 35.0, 34.3, 28.6, 28.4, 27.8; **MS** (ESI) *m/z* 283 ($[M+Na]^+$); **IR** (neat): ν = 3011, 2977, 2962, 2918, 2899, 2223, 1965, 1687, 1475, 1456, 1433, 1392, 1363, 1341, 1321, 1303, 1283, 1250, 1236, 1217, 1181, 1153, 1146, 1121, 1102, 1059, 1036 cm^{-1}; **HRMS** (ESI) calcd for $C_{15}H_{20}O_2N_2Na$ ($[M+Na]^+$): 283.1417, Found:

283.1417.

(24) **2x** (lq-7-195)

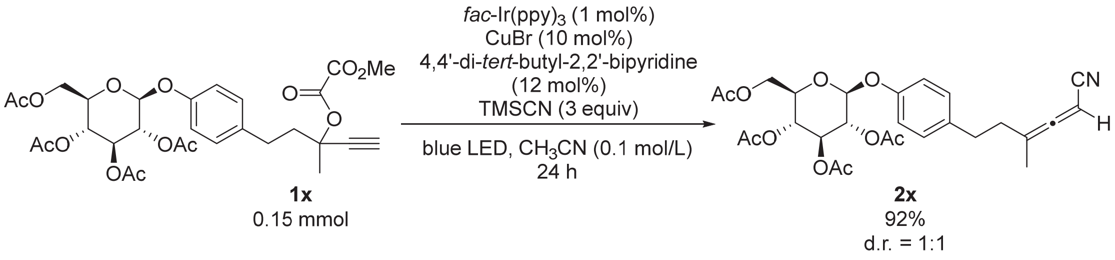

Following **Typical Procedure II**, the reaction of *fac*-Ir(ppy)$_3$ (1.0 mg, 1.5 μmol), CuBr (2.2 mg, 1.5 μmol), 4,4'-di-*tert*-butyl-2,2'-bipyridine (4.9 mg, 1.8 μmol), **1x** (91.2 mg, 0.15 mmol)/CH_3CN(1.5 mL), and TMSCN (59 μL, d = 0.793 g/mL, 46.8 mg, 0.45 mmol) afforded **2x** (73.4 mg, 92%, d.r. = 1:1) (eluent: petroleum ether/ethyl acetate = 3:1 (200 mL) to 2:1 (300 mL)) as a liquid: The d.r. ratio of **2x** was determined to 1:1 by HPLC (HPLC conditions: Chiralpak IF column, hexane/*i*-PrOH = 80/20, 1.0 mL/min, λ = 214 nm, t_{R1} = 21.1 min, t_{R2} = 22.7 min); **^{1}H NMR** (400 MHz, $CDCl_3$) δ 7.10 (d, J = 8.4 Hz, 2 H, ArH), 6.94 (d, J = 8.4 Hz, 2 H, ArH), 5.35-5.22 (m, 2 H, 2 × OCH), 5.17 (t, J = 9.4 Hz, 1 H, OCH), 5.14-5.09 (m, 1 H, =CH), 5.07 (d, J = 7.2 Hz, 1 H, OCH), 4.30 (dd, J_1 = 12.2 Hz, J_2 = 5.0 Hz, 1 H, one proton of OCH_2), 4.17 (dd, J_1 = 12.0 Hz, J_2 = 1.6 Hz, 1 H, one proton of OCH_2), 3.92-3.81 (m, 1 H, OCH), 2.72 (t, J = 7.4 Hz, 2 H, CH_2), 2.42-2.29 (m, 2 H, CH_2), 2.08 (s, 3 H, OCH_3), 2.07 (s, 3 H, OCH_3), 2.05 (s, 3 H, OCH_3), 2.04 (s, 3 H, OCH_3), 1.82 (d, J = 2.8 Hz, 3 H, CH_3); **^{13}C NMR** (100 MHz, $CDCl_3$) δ 213.3, 170.5, 170.1, 169.3, 169.2, 155.3, 135.5, 129.2, 117.1, 114.0, 105.7, 99.2, 72.6, 71.8, 71.0, 68.1, 66.7, 61.8, 34.5, 32.2, 20.6, 20.51, 20.48, 20.45, 17.7; the following signal was discernible for diastereoisomer: δ 105.68; **MS** (ESI) *m/z* 547 ([M+NH$_4$]$^+$), 552 ([M+Na]$^+$); **IR** (neat): ν = 2948, 2223, 1961, 1745, 1610, 1510, 1434, 1367, 1210, 1034 cm^{-1}; **HRMS** (ESI) calcd for $C_{27}H_{31}O_{10}NNa$ ([M+Na]$^+$): 552.1840, Found: 552.1821.

(25) **2y** (lq-6-162)

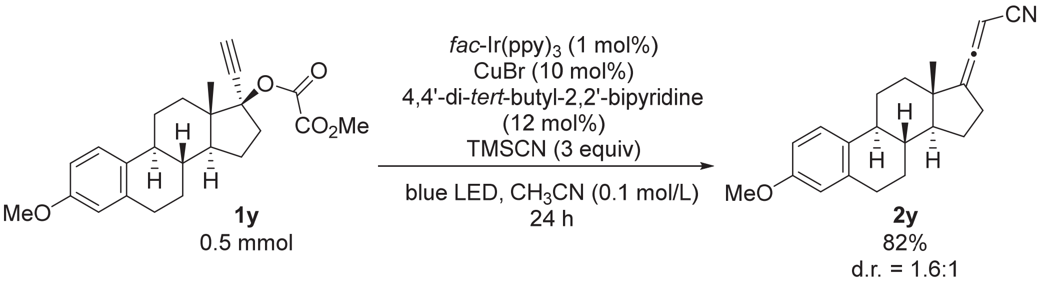

Following **Typical Procedure II**, the reaction of *fac*-Ir(ppy)$_3$ (3.3 mg, 5 μmol), CuBr (7.3 mg, 0.05 mmol), 4,4'-di-*tert*-butyl-2,2'-bipyridine (16.4 mg, 0.06 mmol), **1y** (199.1 mg, 0.5 mmol)/CH_3CN(2.5 mL), and TMSCN (157.0 mg, 1.5 mmol)/CH_3CN(2.5 mL) afforded **2y** (132.3 mg, 82%, d.r. = 1.6:1) (eluent: petroleum ether (5 mL) to petroleum ether/ethyl ether = 20:1 (~42 mL) to 10:1 (~55 mL)) as a white foam (we were not able to obtain the crystal from all the solvent tested, the m.p. value was determined by using the solid right after evaporation of the solvent. When the white solid was heated up to 71.4 °C, it started melting. At 150 °C, the sample started to decompose.); **^{1}H NMR** (400 MHz, $CDCl_3$) δ 7.20 (d, J = 8.4 Hz, 1 H, ArH), 6.71 (dd, J_1 = 8.4 Hz, J_2 = 2.4 Hz, 1 H, ArH), 6.63 (d, J = 2.4 Hz, 1 H, ArH), [5.23 (t, J = 3.8 Hz, 0.38 H), 5.16 (t, J = 4.2 Hz, 0.60 H), 1 H, =CH], 3.77 (s, 3 H, OCH_3), 2.99-2.49 (m, 4 H), 2.45-2.17 (m, 2 H), 2.05-1.81 (m, 3 H), 1.75-1.35 (m, 6 H), [1.00 (s, 1.25 H), 0.95 (s, 1.86 H), 3 H, CH_3]; **MS** (EI) *m/z* (%) 319 (M^+, 100), 318 ($[M-H]^+$, 5.64); **IR** (neat): ν = 2926, 2863, 2218, 1956, 1608, 1575, 1498, 1453, 1430, 1375, 1356, 1312, 1280, 1253, 1236, 1179, 1154, 1137, 1119, 1101, 1035 cm^{-1}; **HRMS** (EI) calcd for $C_{22}H_{25}NO$ (M^+): 319.1936, Found: 319.1942.

Gram-scale synthesis of **2y** (lq-7-166)

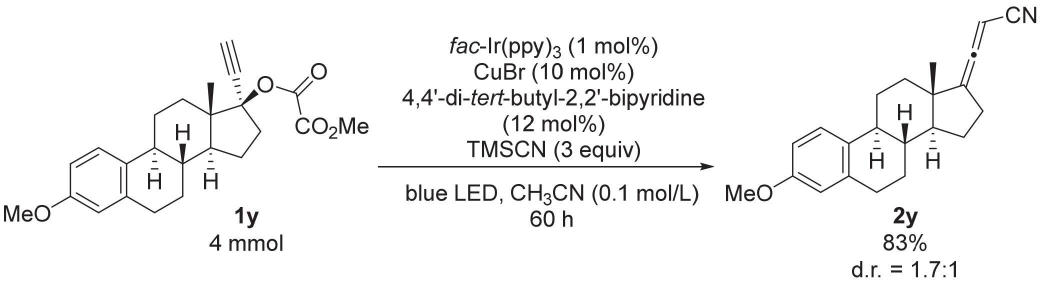

Following **Typical Procedure II**, the reaction of *fac*-Ir(ppy)$_3$ (26.5 mg, 0.04 mmol), CuBr (58.7 mg, 0.4 mmol), 4,4'-di-*tert*-butyl-2,2'-bipyridine (131.7 mg, 0.48

mmol), **1y** (1.5863 g, 4 mmol)/CH_3CN(20 mL), and TMSCN (1.2541 mg, 12 mmol)/CH_3CN(20 mL) afforded **2y** (1.0582 g, 83%, d.r. = 1.7:1) (eluent: petroleum ether (50 mL) to petroleum ether/ethyl ether = 20:1 (~525 mL) to 10:1 (550 mL)) as a white solid. **^{1}H NMR** (400 MHz, $CDCl_3$) δ 7.20 (d, J = 8.8 Hz, 1 H, ArH), 6.71 (dd, J_1 = 8.6 Hz, J_2 = 2.2 Hz, 1 H, ArH), 6.63 (d, J = 2.0 Hz, 1 H, ArH), [5.23 (t, J = 4.0 Hz, 0.35 H), 5.16 (t, J = 4.0 Hz, 0.60 H), 1 H, =CH], 3.77 (s, 3 H, OCH_3), 2.96-2.49 (m, 4 H), 2.44-2.18 (m, 2 H), 2.00-1.83 (m, 3 H), 1.71-1.35 (m, 6 H), [1.00 (s, 1.10 H), 0.95 (s, 1.93 H), 3 H, CH_3].

(26) 4-Cyclohexylbuta-2,3-dienenitrile **2z** (lq-6-191)

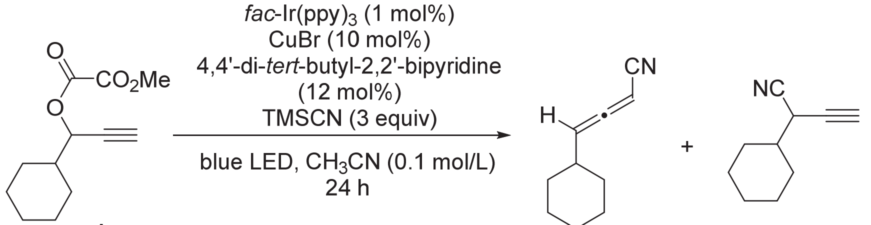

1z
0.5 mmol

2z **3z**

25:1
89%

Following **Typical Procedure II**, the reaction of *fac*-Ir(ppy)$_3$ (3.3 mg, 5 μmol), CuBr (7.3 mg, 0.05 mmol), 4,4'-di-*tert*-butyl-2,2'-bipyridine (16.4 mg, 0.06 mmol), **1z** (112.3 mg, 0.5 mmol)/CH_3CN(2.5 mL), and TMSCN (156.9 mg, 1.5 mmol)/CH_3CN(2.5 mL) afforded a mixture of **2z** and **3z** (65.8 mg, 89%, **2z**/**3z** = 25/1 as determined by ^{1}H NMR analysis)(eluent: petroleum ether/ethyl ether = 100:1) as a liquid: **^{1}H NMR** (400 MHz, $CDCl_3$) δ 5.70 (t, J = 6.4 Hz, 1 H, =CH), 5.22 (dd, J_1 = 6.4 Hz, J_2 = 3.2 Hz, 1 H, =CH), 2.26-2.09 (m, 1 H, CH), 2.00-1.56 (m, 5 H), 1.41-1.04 (m, 5 H); the following signal was discernible for **3z**: δ 3.39 (dd, J_1 = 6.0 Hz, J_2 = 2.4 Hz, 1 H, CH), 2.32 (d, J = 2.4 Hz, 1 H, ≡CH); **^{13}C NMR** (100 MHz, $CDCl_3$) δ 214.6, 113.9, 102.4, 67.9, 36.5, 32.4, 32.3, 25.7, 25.6; **MS** (EI) *m/z* (%) 147 (M^+, 9.66), 146 ($[M-H]^+$, 42.68), 55 (100); **IR** (neat): ν = 2925, 2852, 2225, 1955, 1448, 1349, 1303, 1289, 1258, 1229 cm^{-1}; Anal. Calcd. for $C_{10}H_{13}N$: C 81.58, H 8.90, N 9.51; Found: C 81.21, H 8.91, N 9.54.

(27) Nona-2,3-dienenitrile **2A** (lq-7-038)

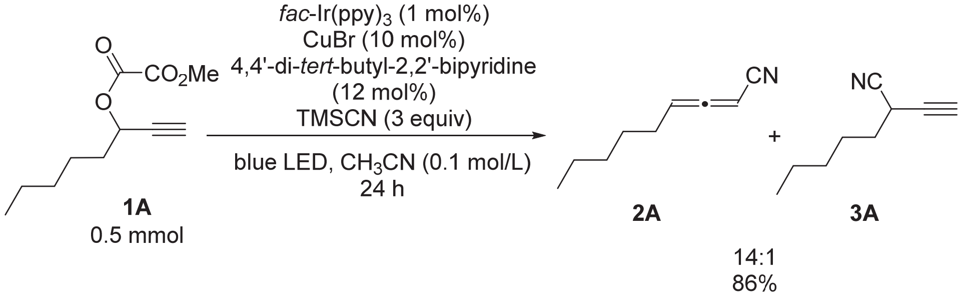

Following **Typical Procedure II**, the reaction of *fac*-Ir(ppy)$_3$ (3.3 mg, 5 μmol), CuBr (7.3 mg, 0.05 mmol), 4,4'-di-*tert*-butyl-2,2'-bipyridine (16.4 mg, 0.06 mmol), **1A** (106.3 mg, 0.5 mmol)/CH_3CN(2.5 mL), and TMSCN (157.1 mg, 1.5 mmol)/CH_3CN(2.5 mL) afforded a mixture of **2A** and **3A** (58.0 mg, 86%, **2A**/**3A** = 14/1 as determined by ^{1}H NMR analysis) (eluent: petroleum ether/ethyl ether = 100:1) as a liquid: **^{1}H NMR** (400 MHz, $CDCl_3$) δ 5.72 (q, J = 6.8 Hz, 1 H, =CH), 5.21 (dt, J_1 = 6.4 Hz, J_2 = 3.2 Hz, 1 H, =CH), 2.14 (qd, J_1 = 7.3 Hz, J_2 = 3.1 Hz, 2 H, CH_2), 1.53-1.41 (m, 2 H, CH_2), 1.40-1.24 (m, 4 H, 2 × CH_2), 0.91 (t, J = 7.0 Hz, 3 H, CH_3); the following signal was discernible for **3A**: δ 3.52 (td, J_1 = 6.9 Hz, J_2 = 2.5 Hz, 1 H, CH), 2.32 (d, J = 2.8 Hz, 1 H, ≡CH), 1.91-1.82 (m, 2 H, CH_2); **^{13}C NMR** (100 MHz, $CDCl_3$) δ 215.2, 113.8, 96.9, 67.2, 31.0, 28.0, 27.2, 22.3, 13.9; the following signal was discernible for **3A**: δ 76.0, 72.4, 32.9, 30.7, 26.1, 22.9, 13.8; **GC-MS** (GC condition: injector: 250 ºC; column: DB5 column 30 m × 0.25 mm, temperature programming: 40 ºC (2 min), 20 ºC/min to 260 ºC, 260 ºC (6 min); detector: 250 ºC) (70 ev, EI) *m/z* (%) for **2A**: t_R (major) = 8.09 min: 135 (M^+, 0.71), 134 ($[M-H]^+$, 6.27), 80 (100); **HRMS** (EI) calcd for $C_9H_{13}N$ [M^+]: 135.1048, found: 135.1044; for **3A**: t_R (minor) = 7.04 min: 135 (M^+, 0.24), 134 ($[M-H]^+$, 2.33), 43 (100); **HRMS** (EI) calcd for $C_9H_{13}N$ [M^+]: 135.1048, found: 135.1046; **IR** (neat): ν = 3018, 2957, 2928, 2859, 2225, 1959, 1465, 1379, 1261, 1104 cm^{-1}.

(28) 4-Phenyl-2,3-butadienenitrile **2J** (lq-9-159-b)

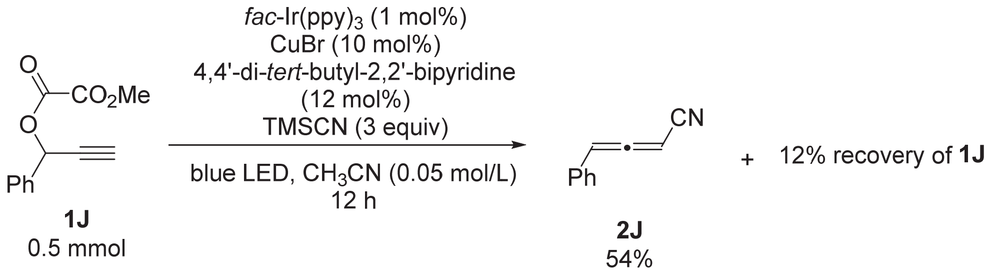

Following **Typical Procedure II**, the reaction of *fac*-Ir(ppy)$_3$ (3.3 mg, 5 μmol), CuBr (7.3 mg, 0.05 mmol), 4,4'-di-*tert*-butyl-2,2'-bipyridine (16.4 mg, 0.06 mmol), **1J** (109.3 mg, 0.5 mmol)/CH_3CN(5 mL), and TMSCN (156.7 mg, 1.5 mmol)/CH_3CN(5 mL) afforded **2**.[12] The recovery of **1J** (12%) was determined by ^{1}H NMR analysis of the crude product using CH_2Br_2 as the internal standard. **2J** was obtained (38.3 mg, 54%) using Biotage Isolera One purification system on flash silica gel column (Santai Tech. Inc., 12 g, flowrate: 25 mL/min, eluent: petroleum ether (40 mL), petroleum ether to petroleum ether/ethyl acetate = 95:5 (linear gradient, 60 mL), 95:5 (200 mL)) as a liquid (it should be noted that this compound is very unstable when concentrated, thus, must handle it as soon as possible): **^{1}H NMR** (400 MHz, $CDCl_3$) δ 7.41-7.28 (m, 5 H, ArH), 6.71 (d, *J* = 6.8 Hz, 1 H, =CH) , 5.67 (d, *J* = 6.8 Hz, 1 H, =CH); **^{13}C NMR** (100 MHz, $CDCl_3$) δ 217.8, 129.3, 129.1, 127.9, 112.5, 99.9, 71.3; **MS** (EI) *m/z* (%) 141 (M^+, 95.56), 140 (100); **IR** (neat): ν = 3059, 3032, 3005, 2992, 2222, 1947, 1602, 1494, 1455, 1259, 1073, 1028 cm^{-1}.

(29) 4,4-Pentamethylene-2-methyl-2,3-butadienenitrile **2B** (lq-7-176)

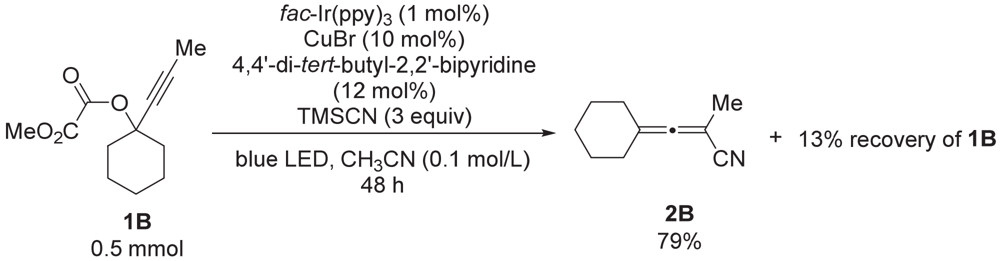

Following **Typical Procedure II**, the reaction of *fac*-Ir(ppy)$_3$ (3.3 mg, 5 μmol), CuBr (7.3 mg, 0.05 mmol), 4,4'-di-*tert*-butyl-2,2'-bipyridine (16.4 mg, 0.06 mmol), **1B** (112.3 mg, 0.5 mmol)/CH_3CN(2.5 mL), and TMSCN (156.8 mg, 1.5

mmol)/CH_3CN(2.5 mL) afforded **2B**. The recovery of **1B** (13%) was determined by ^1H NMR analysis of the crude product using CH_2Br_2 as the internal standard. **2B** (58.5 mg, 79%) (eluent: petroleum ether/ethyl ether = 50:1) as a liquid: **^{1}H NMR** (400 MHz, $CDCl_3$) δ 2.25-2.10 (m, 4 H, 2 × CH_2), 1.88 (s, 3 H, CH_3), 1.74-1.48 (m, 6 H, 3 × CH_2); **^{13}C NMR** (100 MHz, $CDCl_3$) δ 206.6, 117.4, 107.7, 73.9, 30.2, 26.7, 25.5, 18.0; **MS** (EI) *m/z* (%) 147 (M^+, 74.04), 146 ($[M-H]^+$, 97.25), 91 (100); **IR** (neat): ν = 2930, 2855, 2216, 1957, 1440, 1402, 1374, 1341, 1318, 1264, 1247, 1217, 1164, 1128, 1101, 1005 cm^{-1}; **HRMS** (EI) calcd for $C_{10}H_{12}N$ ($[M-H]^+$): 146.0964, Found: 146.0964.

(30) 4,4-Pentamethylene-2-trimethylsilyl-2,3-butadienenitrile **2C** (lq-7-165)

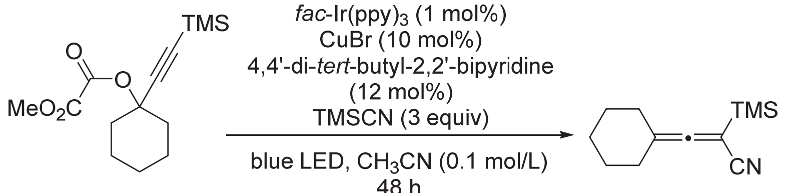

1C
0.5 mmol

2C
88%

Following **Typical Procedure II**, the reaction of *fac*-Ir(ppy)$_3$ (3.3 mg, 5 μmol), CuBr (7.3 mg, 0.05 mmol), 4,4'-di-*tert*-butyl-2,2'-bipyridine (16.4 mg, 0.06 mmol), **1C** (141.3 mg, 0.5 mmol)/CH_3CN(2.5 mL), and TMSCN (156.8 mg, 1.5 mmol)/CH_3CN(2.5 mL) afforded **2C** (90.8 mg, 88%) (eluent: petroleum ether (50 mL) to petroleum ether/ethyl ether = 50:1 (~200 mL)) as a liquid: **^{1}H NMR** (400 MHz, $CDCl_3$) δ 2.25-2.10 (m, 4 H, 2 × CH_2), 1.77-1.41 (m, 6 H, 3 × CH_2), 0.22 (s, 9 H, $Si(CH_3)_3$); **^{13}C NMR** (100 MHz, $CDCl_3$) δ 213.7, 116.0, 100.9, 73.1, 29.3, 26.7, 25.5, -1.8; **MS** (EI) *m/z* (%) 205 (M^+, 47.38), 190 ($[M-Me]^+$, 16.8), 73 (100); **IR** (neat): ν = 2931, 2855, 2204, 1936, 1447, 1410, 1385, 1338, 1313, 1250, 1017 cm^{-1}; **HRMS** (EI) calcd for $C_{12}H_{19}NSi$ (M^+): 205.1281, Found: 205.1278.

(31) 2-Cyclohexyl-4,4-pentamethylene-2,3-butadienenitrile **2K** (lq-9-160)

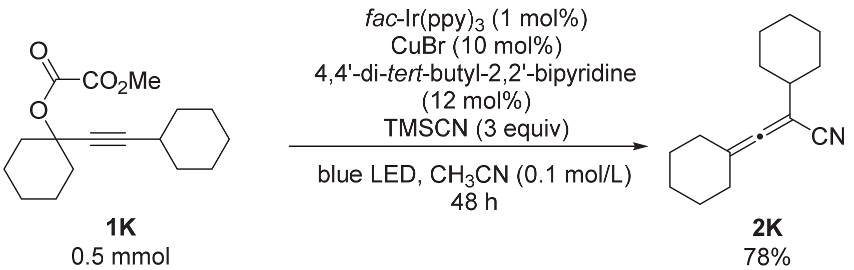

Following **Typical Procedure II**, the reaction of *fac*-Ir(ppy)$_3$ (3.3 mg, 5 μmol), CuBr (7.3 mg, 0.05 mmol), 4,4'-di-*tert*-butyl-2,2'-bipyridine (16.4 mg, 0.06 mmol), **1K** (146.5 mg, 0.5 mmol)/CH_3CN(2.5 mL), and TMSCN (157.0 mg, 1.5 mol)/CH_3CN(2.5 mL) afforded **2K** (84.6 mg, 76%, purity 97%) (using Biotage Isolera One purification system on flash silica gel column (Santai Tech. Inc., 12 g), flowrate: 25 mL/min, eluent: petroleum ether (40 mL), petroleum ether/ethyl acetate = 95:5 (200 mL)) as a liquid: **^{1}H NMR** (400 MHz, $CDCl_3$) δ 2.18 (t, J = 5.6 Hz, 4 H, 2 × CH_2), 2.08 (tt, J_1 = 11.2 Hz, J_2 = 3.5 Hz, 1 H, CH), 1.93-1.49 (m, 11 H), 1.36-1.09 (m, 5 H); **^{13}C NMR** (100 MHz, $CDCl_3$) δ 204.9, 116.4, 109.1, 85.0, 39.3, 31.6, 30.5, 26.8, 25.7, 25.6, 25.5; **MS** (EI) *m/z* (%) 215 (M^+, 11.16), 186 (100); **IR** (neat): ν = 2926, 2853, 2213, 1954, 1448, 1341, 1302, 1237, 1004 cm^{-1}; **HRMS** (EI) calcd for $C_{15}H_{21}N$ (M^+): 215.1669, Found: 215.1673.

The reaction of 1D and 1E affording dinitrile products

1. 2-Cyclohexylidene-3-phenylbutanedinitrile **4a** (lq-7-175)

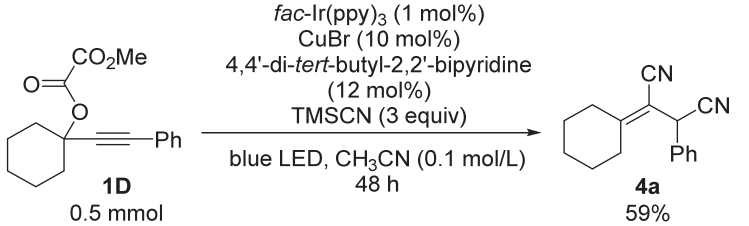

Following **Typical Procedure II**, the reaction of *fac*-Ir(ppy)$_3$ (3.3 mg, 5 μmol), CuBr (7.3 mg, 0.05 mmol), 4,4'-di-*tert*-butyl-2,2'-bipyridine (16.4 mg, 0.06 mmol), **1D** (143.5 mg, 0.5 mmol)/CH_3CN(2.5 mL), and TMSCN (157.2 mg, 1.5 mmol)/CH_3CN(2.5 mL) afforded **4a** (70.0 mg, 59%) (eluent: petroleum ether/ethyl acetate = 20:1 (~100 mL) to 10:1 (330 mL)) as a liquid: **^{1}H NMR** (400 MHz, $CDCl_3$) δ 7.53-7.32 (m, 5 H, ArH), 5.05 (s, 1 H, CH), 2.66-2.52 (m, 2 H), 2.51-2.39 (m, 2 H),

1.83-1.54 (m, 6 H); **^{13}C NMR** (100 MHz, $CDCl_3$) δ 164.1, 132.4, 129.3, 128.8, 127.0, 117.2, 115.8, 103.1, 36.1, 35.6, 31.2, 27.8, 27.4, 25.5; **MS** (EI) *m/z* (%) 236 (M^+, 100), 235 ($[M-H]^+$, 40.14); **IR** (neat): ν = 3033, 2936, 2859, 2244, 2214, 1783, 1620, 1494, 1450, 1353, 1319, 1255, 1155, 1079, 1030, 1001, 726, 697 cm^{-1}; **HRMS** (EI) calcd for $C_{16}H_{16}N_2$ (M^+): 236.1313, Found: 236.1316.

2. Methyl 2,3-dicyano-3-cyclohexylidenepropanoate **4b** (lq-8-023)

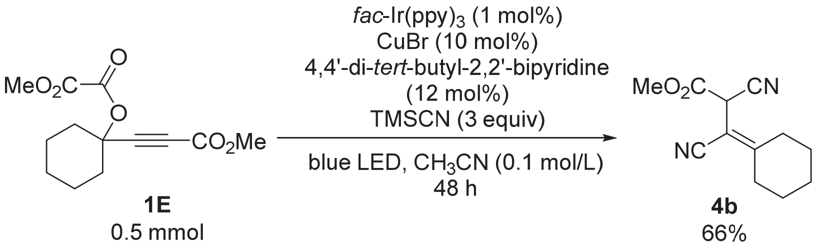

Following **Typical Procedure II**, the reaction of *fac*-Ir(ppy)$_3$ (3.3 mg, 5 μmol), CuBr (7.3 mg, 0.05 mmol), 4,4'-di-*tert*-butyl-2,2'-bipyridine (16.4 mg, 0.06 mmol), **1E** (134.6 mg, 0.5 mmol)/CH_3CN(2.5 mL), and TMSCN (157.1 mg, 1.5 mmol)/CH_3CN(2.5 mL) afforded **4b** (72.5 mg, 66%) (eluent: petroleum ether/ethyl acetate = 10:1 (~110 mL) to 5:1 (360 mL)) as a liquid: **^{1}H NMR** (400 MHz, $CDCl_3$) δ 4.61 (s, 1 H, CH), 3.90 (s, 3 H, OCH_3), 2.71-2.52 (m, 2 H), 2.46-2.28 (m, 2 H), 1.84-1.55 (m, 6 H); **^{13}C NMR** (100 MHz, $CDCl_3$) δ 168.2, 163.5, 115.5, 113.5, 96.8, 54.4, 37.3, 35.6, 31.5, 27.7, 27.2, 25.3; **MS** (EI) *m/z* (%) 218 (M^+, 29.7), 217 ($[M-H]^+$, 3.9), 203 ($[M-CH_3]^+$, 86.59), 159 (100); **IR** (neat): ν = 2939, 2861, 2217, 1751, 1622, 1436, 1354, 1260, 1206, 1173, 1110, 1027 cm^{-1}; **HRMS** (EI) calcd for $C_{12}H_{14}N_2O_2$ (M^+): 218.1055, Found: 218.1053.

Synthesis of haloallenes

(1) 3-(Adamantan-1-yl)-1-chlorobuta-1,2-diene **12l** (lq-9-175)

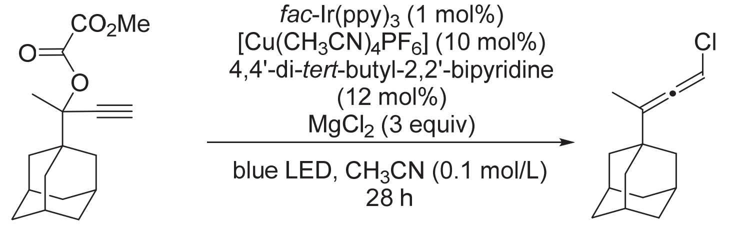

1l
0.2 mmol

12l
73%

Typical Procedure III, to a flame-dried 10 mL Schlenk tube were added *fac*-Ir(ppy)$_3$ (1.3 mg, 2 μmol), [Cu(CH$_3$CN)$_4$PF$_6$] (7.6 mg, 0.02 mmol), 4,4'-di-*tert*-butyl-2,2'-bipyridine (6.6 mg, 0.024 mmol), **1l** (58.2 mg, 0.2 mmol)/CH$_3$CN(1 mL), and MgCl$_2$ (59.0 mg, 0.6 mmol)/CH$_3$CN(1 mL) sequentially under Ar atmosphere. The resulting mixture was irradiated with a 50 W 460 nm blue LED lamp (2-3 cm away, with cooling fan to keep the reaction temperature at 35~40 °C) for 28 h with stirring. The resulting mixture was filtrated through a short pad of silica gel eluted with ethyl ether (30 mL). After evaporation, the residue was purified by chromatography on silica gel (eluent: petroleum ether) to afford **12l** (32.6 mg, 73%) as a liquid: **^{1}H NMR** (400 MHz, CDCl$_3$) δ 5.96 (q, *J* = 2.0 Hz, 1 H, =CH), 2.07-1.97 (m, 3 H), 1.77 (d, *J* = 2.0 Hz, 3 H, CH$_3$), 1.75-1.61 (m, 12 H); **^{13}C NMR** (100 MHz, CDCl$_3$) δ 198.7, 121.4, 87.5, 40.8, 36.7, 36.3, 28.5, 14.0; **MS** (EI) *m/z* (%) 224 (M(^{37}Cl)$^+$, 0.65), 222 (M(^{35}Cl)$^+$, 1.97), 135 (100); **IR** (neat): ν = 2943, 2928, 2907, 2902, 2849, 1954, 1452, 1445, 1336, 1205, 1057 cm^{-1}; **HRMS** (EI) calcd for $C_{14}H_{19}{}^{35}Cl$ (M(^{35}Cl)$^+$): 222.1170, Found: 222.1172.

(2) 5-(Benzo[d][1,3]dioxol-5-yl)-1-chloro-3-methylpenta-1,2-diene **12s** (lq-9-171)

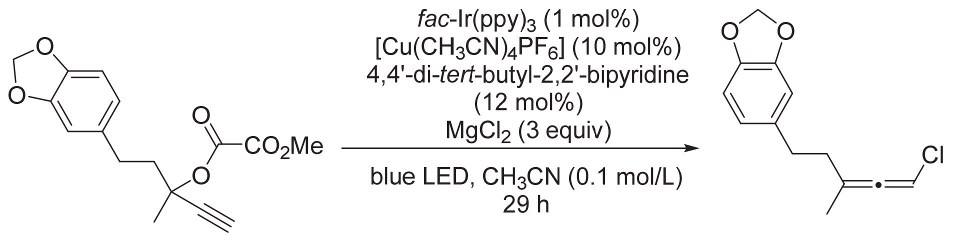

1s
0.2 mmol

12s
64%

Following **Typical Procedure III**, the reaction of *fac*-Ir(ppy)$_3$ (1.3 mg, 2 μmol), [Cu(CH$_3$CN)$_4$PF$_6$] (7.6 mg, 0.02 mmol), 4,4’-di-*tert*-butyl-2,2’-bipyridine (6.6 mg, 0.024 mmol), **1s** (61.2 mg, 0.2 mmol)/CH$_3$CN(1 mL), and MgCl$_2$ (59.0 mg, 0.6

mmol)/CH_3CN(1 mL) afforded **12s** (30.5 mg, 64%) (eluent: petroleum ether) as a liquid: **^{1}H NMR** (400 MHz, $CDCl_3$) δ 6.73 (d, J = 8.0 Hz, 1 H, ArH), 6.01-5.96 (m, 1 H, ArH), 6.64 (dd, J_1 = 7.8 Hz, J_2 = 1.40 Hz, 1 H, ArH), 5.98 (q, J = 2.1 Hz, 1 H, =CH), 5.92 (s, 2 H, OCH_2O), 2.68 (t, J = 7.8 Hz, 2 H, CH_2), 2.37-2.28 (m, 2 H, CH_2), 1.82 (d, J = 2.0 Hz, 3 H, CH_3); **^{13}C NMR** (100 MHz, $CDCl_3$) δ 199.1, 147.5, 145.7, 135.2, 121.1, 111.8, 108.8, 108.1, 100.8, 87.8, 36.2, 33.2, 19.7; **MS** (EI) *m/z* (%) 238 ($M(^{37}Cl)^+$, 5.45), 236 ($M(^{35}Cl)^+$, 17.5), 135 (100); **IR** (neat): ν = 2875, 1960, 1609, 1503, 1489, 1443, 1362, 1244, 1202, 1189, 1097, 1068, 1040 cm^{-1}; **HRMS** (EI) calcd for $C_{13}H_{13}{}^{35}ClO_2$ ($M(^{35}Cl)^+$): 236.0599, Found: 236.0598.

(3) 3-(Adamantan-1-yl)-1-bromo-buta-1,2-diene **13l** (lq-9-170)

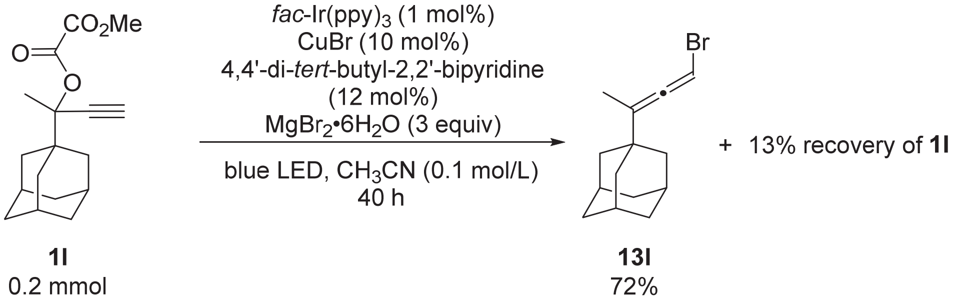

Following **Typical Procedure III**, the reaction of *fac*-Ir(ppy)$_3$ (1.3 mg, 2 μmol), CuBr (2.9 mg, 0.02 mmol), 4,4'-di-*tert*-butyl-2,2'-bipyridine (6.6 mg, 0.024 mmol), **1l** (58.7 mg, 0.2 mmol)/CH_3CN(1 mL), and $MgBr_2$•$6H_2O$ (179.0 mg, 0.6 mmol)/CH_3CN(1 mL) afforded **13l**. The recovery of **1l** (13%) was determined by ^{1}H NMR analysis of the crude product using CH_2Br_2 as the internal standard. **13l** (39.0 mg, 72%) as a liquid: **^{1}H NMR** (400 MHz, $CDCl_3$) δ 5.89 (q, J = 2.0 Hz, 1 H, =CH), 2.07-1.96 (m, 3 H), 1.78 (d, J = 2.0 Hz, 3 H, CH_3), 1.75-1.61 (m, 12 H); **^{13}C NMR** (100 MHz, $CDCl_3$) δ 199.0, 120.5, 71.8, 40.7, 36.7, 35.9, 28.5, 13.6; **MS** (EI) *m/z* (%) 268 ($M(^{81}Br)^+$, 1.24), 266 ($M(^{79}Br)^+$, 1.23), 135 (100); **IR** (neat): ν = 2983, 2900, 2848, 1947, 1450, 1365, 1357, 1338, 1313, 1257, 1177, 1153, 1102, 1056 cm^{-1}; **HRMS** (EI) calcd for $C_{14}H_{19}{}^{79}Br$ ($M(^{79}Br)^+$): 266.0665, Found: 266.0670.

(4) 5-Bromo-3-methylpenta-3,4-dienyl 4-ethynylbenzoate **13q** (lq-9-186)

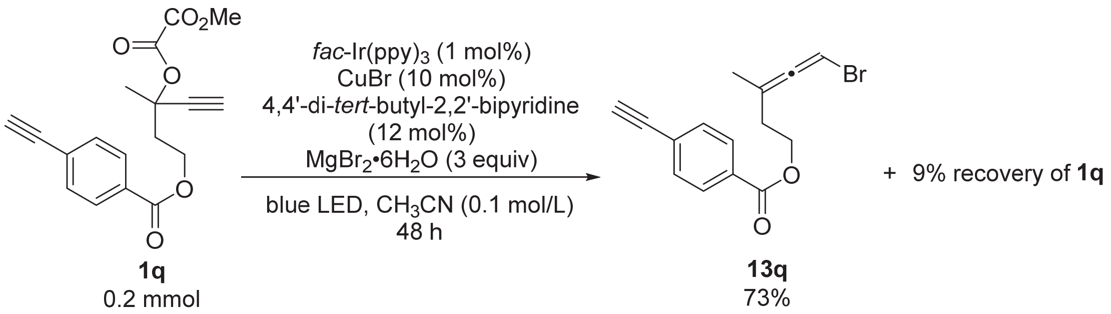

Following **Typical Procedure III**, the reaction of *fac*-Ir(ppy)$_3$ (1.3 mg, 2 μmol), CuBr (2.9 mg, 0.02 mmol), 4,4'-di-*tert*-butyl-2,2'-bipyridine (6.6 mg, 0.024 mmol), **1q** (65.8 mg, 0.2 mmol)/CH_3CN(1 mL), and $MgBr_2$•$6H_2O$ (179.5 mg, 0.6 mmol)/CH_3CN(1 mL) afforded **13q**. The recovery of **1q** (9%) was determined by ^{1}H NMR analysis of the crude product using CH_2Br_2 as the internal standard. **13q** (44.8 mg, 73%) as a liquid: **^{1}H NMR** (400 MHz, $CDCl_3$) δ 8.01 (d, *J* = 8.4 Hz, 2 H, ArH), 7.55 (d, *J* = 8.4 Hz, 2 H, ArH), 6.00-5.87 (m, 1 H, =CH), 4.52-4.37 (m, 2 H, OCH_2), 3.24 (s, 1 H, C≡CH), 2.56 (t, *J* = 6.2 Hz, 2 H, CH_2), 1.91 (d, *J* = 1.2 Hz, 3 H, CH_3); **^{13}C NMR** (100 MHz, $CDCl_3$) δ 199.6, 165.8, 132.0, 130.0, 129.6, 126.7, 107.3, 82.8, 80.1, 72.1, 62.4, 32.9, 19.0; **MS** (ESI) *m/z* 305 ([M(^{79}Br)+H]$^+$), 307 ([M(^{81}Br)+H]$^+$), 322 ([M(^{79}Br)+NH_4]$^+$), 324 ([M(^{81}Br)+NH_4]$^+$); **IR** (neat): ν = 3288, 2965, 2916, 1956, 1716, 1607, 1449, 1405, 1365, 1307, 1269, 1173, 1106, 1018 cm^{-1}; **HRMS** (ESI) calcd for $C_{15}H_{14}O_2{}^{79}Br$ ([M(^{79}Br)+H]$^+$): 305.0172, Found: 305.0171.

Synthetic transformations

1. Synthesis of *endo*-**5** and *exo*-**5** (lq-8-004-b)

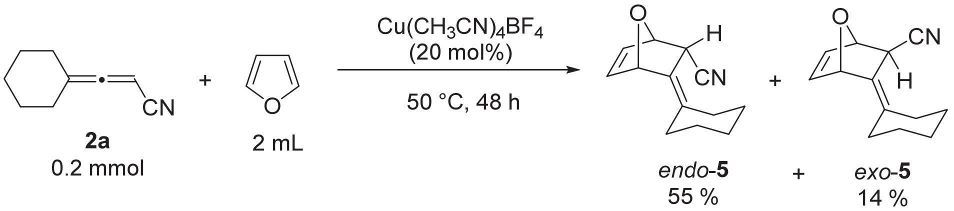

To a dried Schlenk tube were added $Cu(CH_3CN)_4BF_4$ (12.7 mg, 0.04 mmol), **2a** (26.7 mg, 0.2 mmol), and freshly distilled furan (2 mL). The Schlenk tube was then placed in an oil bath pre-heated at 50 ºC for 48 h. After cooling to room temperature,

the crude reaction mixture was filtered through a short pad of celite eluted with ethyl ether (30 mL). After evaporation, the residue was purified by chromatography on silica gel (eluent: petroleum ether/ethyl ether = 20:1 (~50 mL) to 10:1 (220 mL) to petroleum ether/ethyl acetate = 10:1 (220 mL)) to afford *endo*-**5** (22.0 mg, 55%, less polar) and *exo*-**5** (5.6 mg, 14%, more polar).

endo-**5**: a white solid, m.p. 136.0-136.5 °C (ethyl acetate/*n*-hexane); **^{1}H NMR** (400 MHz, $CDCl_3$) δ 6.60 (d, J = 5.6 Hz, 1 H, =CH), 6.49 (d, J = 5.6 Hz, 1 H, =CH), 5.40 (s, 1 H, OCH), 5.29 (d, J = 4.0 Hz, 1 H, OCH), 3.49 (d, J = 4.0 Hz, 1 H, CH), 2.39-2.04 (m, 4 H), 1.77-1.38 (m, 6 H); **^{13}C NMR** (100 MHz, $CDCl_3$) δ 136.3, 135.8, 132.7, 122.8, 119.3, 79.8, 79.6, 33.0, 32.1, 31.1, 27.7, 26.8, 26.1; **MS** (EI) *m/z* (%) 201 (M^+, 25), 172 (100); **IR** (neat): ν = 2970, 2950, 2927, 2851, 2237, 1441, 1349, 1319, 1301, 1221, 1156, 1133, 1079, 1059, 1020 cm^{-1}; **HRMS** (EI) calcd for $C_{13}H_{15}NO$ (M^+): 201.1154, Found: 201.1157.

exo-**5**: a white solid, m.p. 95.7-96.4 °C (*n*-hexane); **^{1}H NMR** (400 MHz, $CDCl_3$) δ 6.49 (dd, J_1 = 5.8 Hz, J_2 = 1.4 Hz, 1 H, =CH), 6.27 (dd, J_1 = 5.6 Hz, J_2 = 1.6 Hz, 1 H, =CH), 5.43 (s, 1 H, OCH), 5.31 (s, 1 H, OCH), 2.90 (s, 1 H, CH), 2.38-1.97 (m, 4 H), 1.78-1.46 (m, 6 H); **^{13}C NMR** (100 MHz, $CDCl_3$) δ 136.8, 136.2, 132.3, 122.9, 119.9, 82.1, 78.6, 33.2, 32.1, 32.0, 27.6, 26.9, 26.1; **MS** (EI) *m/z* (%) 201 (M^+, 11.5), 172 (100); **IR** (neat): ν = 2956, 2928, 2850, 2232, 1442, 1346, 1314, 1299, 1239, 1158, 1139, 1078, 1062, 1019 cm^{-1}; **HRMS** (EI) calcd for $C_{13}H_{15}NO$ (M^+): 201.1154, Found: 201.1156.

2. Synthesis of 4,4-Pentamethylene-3-[(4-methylphenyl)thio]-3-butenenitrile **6** (lq-7-065)

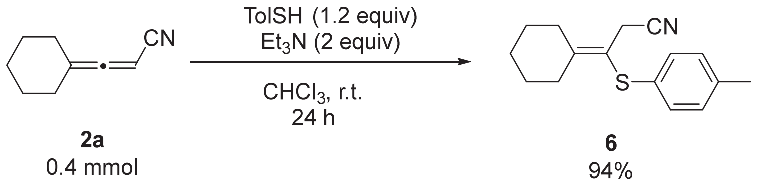

To a round-bottomed flask were added **2a** (53.5 mg, 0.4 mmol)/$CHCl_3$ (1 mL), 4-methylbenzenethiol (60.8 mg, 0.48 mmol), and NEt_3 (0.11 mL, d = 0.726 g/mL, 79.9 mg, 0.8 mmol) sequentially. After being stirred at room temperature for 24 h as

monitored by TLC, the crude resulting mixture was concentrated under reduced pressure. The residue was purified by chromatography on silica gel (eluent: petroleum ether/ethyl ether = 50:1) to afford **6** (97.0 mg, 94%) as a white solid (m.p. 93.1-94.2 °C, *n*-hexane): **^{1}H NMR** (400 MHz, $CDCl_3$) δ 7.17 (d, J = 8.0 Hz, 2 H, ArH), 7.11 (d, J = 7.6 Hz, 2 H, ArH), 3.26 (s, 2 H, CH_2), 2.71-2.60 (m, 2 H, two protons of 2 × CH_2), 2.37 (t, J = 5.8 Hz, 2 H, two protons of 2 × CH_2), 2.32 (s, 3 H, CH_3), 1.75-1.46 (m, 6 H); **^{13}C NMR** (100 MHz, $CDCl_3$) δ 152.5, 136.7, 130.9, 130.0, 129.7, 117.7, 113.0, 33.2, 32.2, 28.0, 27.6, 26.1, 22.0, 21.0; **MS** (EI) *m/z* (%) 257 (M^+, 100); **IR** (neat): ν = 2929, 2853, 2247, 1616, 1490, 1439, 1403, 1324, 1305, 1255, 1235, 1209, 1121, 1086, 1050, 1015 cm^{-1}; Anal. Calcd. for $C_{16}H_{19}NS$: C 74.66, H 7.44, N, 5.44; Found: C 74.67, H 7.46, N, 5.39.

3. Synthesis of 4-methyl-6-phenylhexa-2,3-dienenitrile-2-*d* *d*-**2m** (lq-7-069)

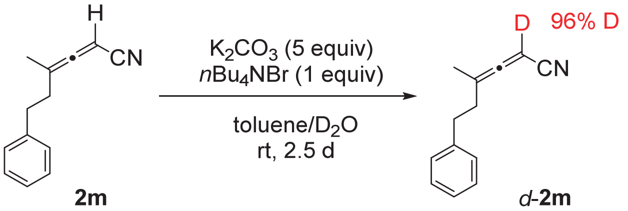

2m 0.27 mmol; *d*-**2m** 96%

To a round-bottomed flask were added **2m** (49.6 mg, 0.27 mmol), *n*Bu_4NBr (87.9 mg, 0.27 mmol), toluene (0.45 mL), and D_2O (0.55 mL, D-incorporation: 99.8%) sequentially. After being stirred at room temperature for 2.5 d, the crude resulting mixture was extracted with ethyl ether (5 × 2 mL). The combined organic layer was dried over anhydrous Na_2SO_4. After filtration and evaporation, the residue was purified by chromatography on silica gel (eluent: petroleum ether/ethyl ether = 50:1) to afford *d*-**2m** (47.4 mg, 96%, D-incorporation: 96%) as a liquid: **^{1}H NMR** (400 MHz, $CDCl_3$) δ 7.30 (t, J = 7.6 Hz, 2 H, ArH), 7.23-7.14 (m, 3 H, ArH), 2.75 (t, J = 7.8 Hz, 2 H, CH_2), 2.36 (dd, J_1 = 8.6 Hz, J_2 = 7.0 Hz, 2 H, CH_2), 1.81 (s, 3 H, CH_3); **^{13}C NMR** (100 MHz, $CDCl_3$) δ 213.3, 140.5, 128.4, 128.2, 126.2, 114.1, 106.0, 66.5 (t, $J_{C\text{-}D}$ = 28.0 Hz), 34.4, 33.2, 17.8; **MS** (EI) *m/z* (%) 184 (M^+, 12.91), 182 ($[M\text{-}D]^+$, 4.14), 91 (100); **IR** (neat): ν = 3062, 3027, 2987, 2921, 2859, 2217, 1950, 1602, 1496, 1453, 1373, 1334, 1261, 1154, 1079, 1046, 1030 cm^{-1}; **HRMS** (EI) calcd for $C_{13}DH_{12}N$ (M^+): 184.1111, Found:

184.1110.

4. Synthesis of 4-(adamantan-1-yl)penta-2,3-dienamide **7** (lq-8-025)

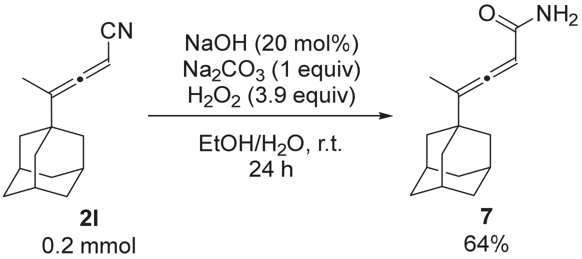

To a round-bottomed flask were added **2l** (42.5 mg, 0.2 mmol)/EtOH (0.5 mL), H_2O (0.1 mL), NaOH (1.8 mg, 0.04 mmol), and Na_2CO_3 (21.3 mg, 0.2 mmol) sequentially. Then H_2O_2 (80 μL, 30 % aqueous solution, d = 1.11 g/mL, 0.78 mmol) was added dropwise. The resulting mixture was stirred at room temperature for 24 h as monitored by TLC. The crude resulting mixture was filtrated through a short pad of basic aluminum oxide (200-300 mesh) eluted with dichloromethane/MeOH (20:1, 21 mL). After evaporation, the residue was purified by chromatography on silica gel (eluent: dichloromethane (50 mL) to dichloromethane/MeOH = 20:1 (~210 mL)) to afford **7** (29.6 mg, 64%) as a white solid (m.p. 155.0-155.7 °C, dichloromethane/petroleum ether): **^{1}H NMR** (400 MHz, $CDCl_3$) δ 5.98-5.66 (br, 2 H, $CONH_2$), 5.54-5.43 (m, 1 H, =CH), 2.07-1.99 (m, 3 H), 1.77 (d, *J* = 2.8 Hz, 3 H, CH_3), 1.76-1.62 (m, 12 H); **^{13}C NMR** (100 MHz, $CDCl_3$) δ 205.7, 168.9, 115.1, 90.7, 41.0, 36.6, 35.8, 28.5, 12.9; **MS** (EI) *m/z* (%) 231 (M^+, 6.85), 135 (100); **IR** (neat): ν = 3393, 3169, 2899, 2846, 1953, 1650, 1614, 1446, 1398, 1359, 1342, 1263, 1205, 1158, 1101, 1058 cm^{-1}; **HRMS** (EI) calcd for $C_{15}H_{21}NO$ (M^+): 231.1623, Found: 231.1621.

5. Synthesis of **8** (lq-8-068)

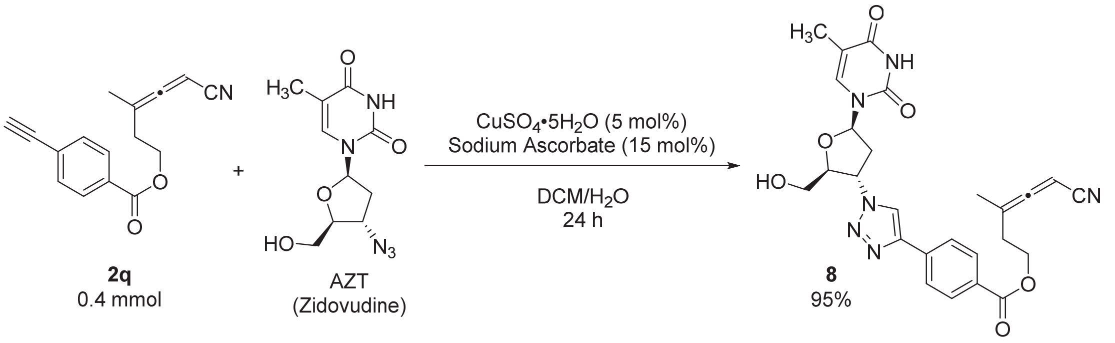

To a round-bottomed flask were added AZT (109.4 mg, 0.4 mmol), **2q** (100.7 mg, 0.4 mmol)/dichloromethane (2 mL), H_2O (2 mL), $CuSO_4•5H_2O$ (5.1 mg, 0.02 mmol), and sodium ascorbate (12.0 mg, 0.06 mmol). The resulting solution was stirred at room temperature for 24 h as monitored by TLC, diluted with H_2O (5 mL), and extracted with dichloromethane (3 × 5 mL). The combined organic layer was dried over anhydrous Na_2SO_4. After filtration and evaporation, the residue was purified by using Biotage Isolera One purification system on flash silica gel column (Santai Tech. Inc., 12 g), flowrate: 25 mL/min, eluent: dichloromethane (40 mL), dichloromethane to dichloromethane/MeOH = 92:8 (linear gradient, 200 mL), 92:8 (110 mL) to afford **8** (197.6 mg, 95%) as a white solid (m.p. 155.1-157.0 °C, $CHCl_3$): **^{1}H NMR** (400 MHz, d_6-DMSO) δ 11.38 (s, 1 H, NH), 8.96 (s, 1 H, =CH), 8.07 (d, J = 8.4 Hz, 2 H, ArH), 8.01 (d, J = 8.4 Hz, 2 H, ArH), 7.85 (s, 1 H, =CH), 6.47 (t, J = 6.6 Hz, 1 H, OCH), 5.86-5.73 (m, 1 H, C=CH), 5.51-5.39 (m, 1 H, NCH), 5.32 (t, J = 5.2 Hz, 1 H, OH), 4.48-4.34 (m, 2 H, OCH_2), 4.34-4.26 (m, 1 H, OCH), 3.82-3.62 (m, 2 H, OCH_2), 2.89-2.78 (m, 1 H, one proton of CH_2), 2.77-2.67 (m, 1 H, one proton of CH_2), 2.63-2.54 (m, 2 H, CH_2), 1.88 (d, J = 2.8 Hz, 3 H, CH_3), 1.83 (s, 3 H, CH_3); **^{13}C NMR** (100 MHz, d_6-DMSO) δ 212.7, 165.3, 163.8, 150.5, 145.5, 136.3, 135.2, 130.0, 128.7, 125.2, 122.3, 114.4, 109.7, 102.9, 84.4, 83.9, 66.9, 61.7, 60.8, 59.5, 37.1, 31.7, 17.3, 12.3; **MS** (ESI) m/z 519 ([M+H]$^+$); **IR** (neat): ν = 3448, 2922, 2223, 1962, 1686, 1614, 1466, 1413, 1376, 1270, 1178, 1097, 1016 cm^{-1}; **HRMS** (ESI) calcd for $C_{26}H_{27}O_6N_6$ ([M+H]$^+$): 519.1987, Found: 519.1981.

Mechanistic studies

Cyclic voltammetry experiments

Cyclic voltammograms were recorded with a CHI660E potentiostat at room temperature in CH_3CN. *n*Bu_4NPF_6 (0.1 M) was used as the supporting electrolyte, a glassy carbon was used as the working electrode, a Pt sheet was used as auxiliary electrode, and a saturated calomel electrode was used as the reference electrode. The scan rate was 100 mV/s.

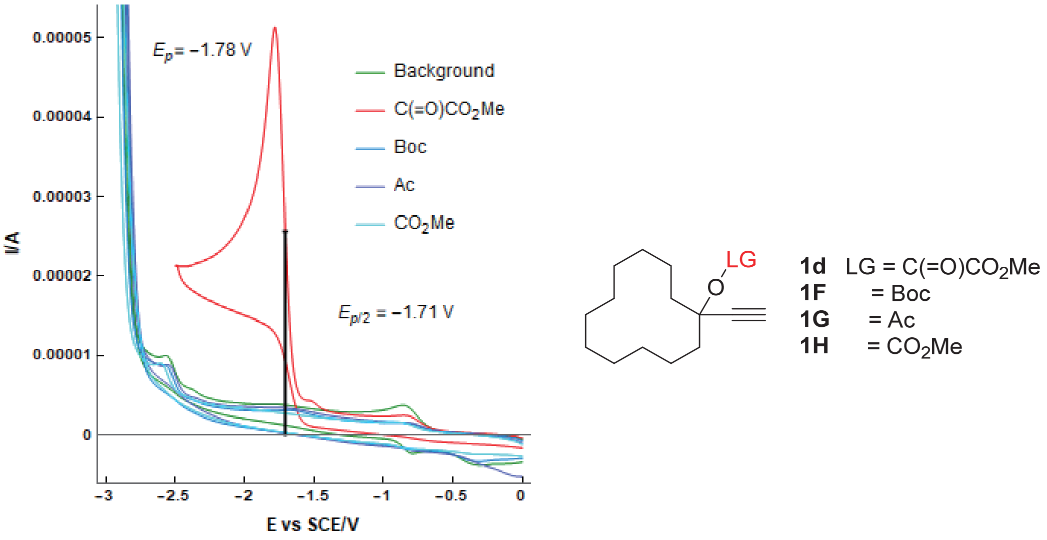

Supplementary Figure 3. Cyclic voltammogram of different propargylic compounds

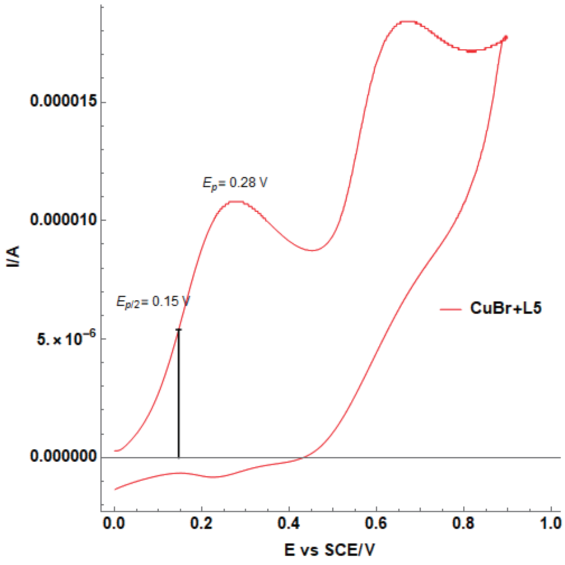

Supplementary Figure 4. Cyclic voltammogram of CuBr/**L5**

Proposed an oxidative quenching mechanism

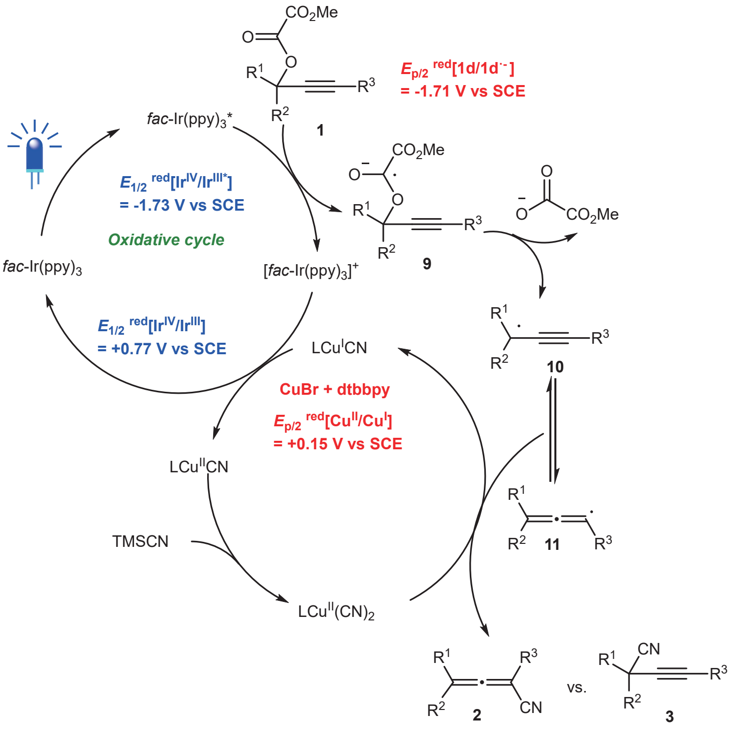

Supplementary Figure 5. Proposed Mechanism via Oxidative Quenching Cycle

Stern-Volmer quenching experiments

Stern–Volmer quenching experiments were conducted following a known report.[13] Fluorescence spectra was collected on Agilent Fluorescence Spectrophotometer G9800A for all experiments. All *fac*-Ir(ppy)3 solutions were excited at 450 nm and the emission intensity was collected at around 520 nm. In a typical experiment, the emission spectrum of a 2×10^{-3} M solution of *fac*-Ir(ppy)3 in MeCN was collected.

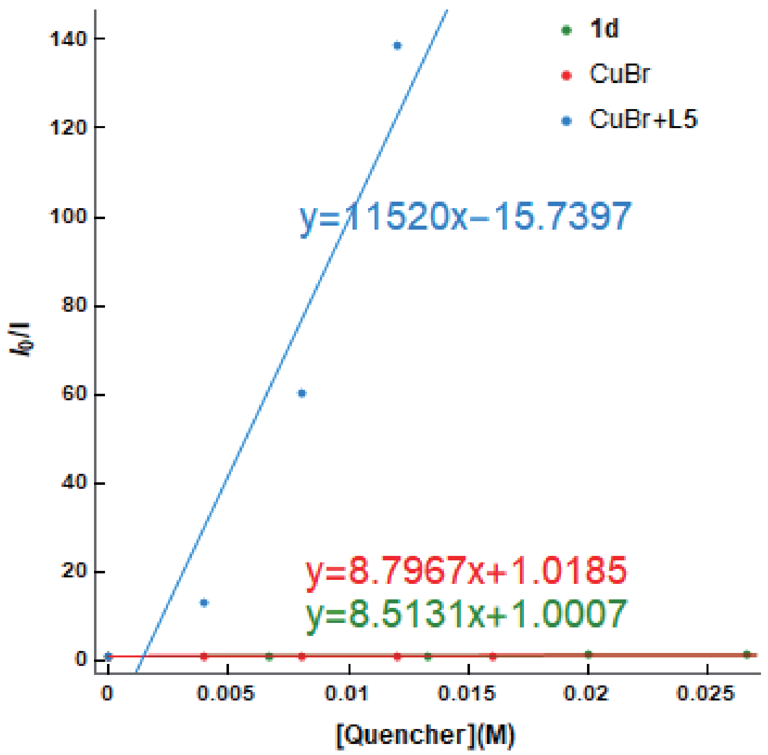

Supplementary Figure 6. Stern–Volmer Quenching Experiments for the Photocatalyst *fac*-Ir(ppy)3

Reaction with Ph-PTZ photocatalyst

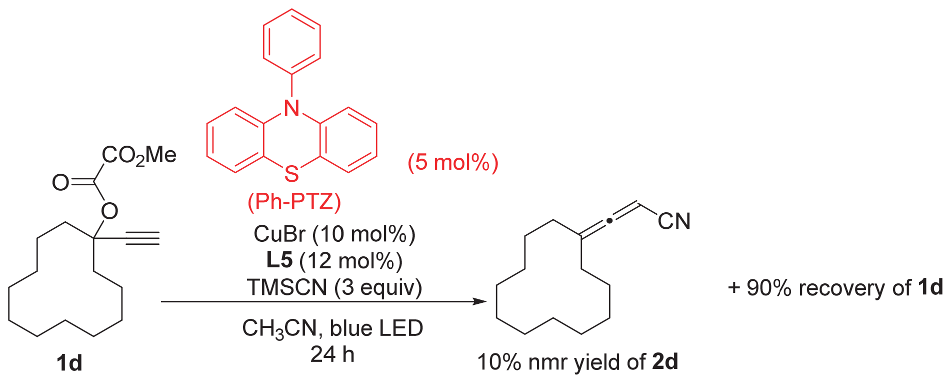

To a flame-dried Schlenk tube were added Ph-PTZ (2.8 mg, 0.01 mmol), CuBr (2.9 mg, 0.02 mmol), 4,4'-di-*tert*-butyl-2,2'-bipyridine (6.6 mg, 0.024 mmol), **1d** (58.9 mg, 0.2 mmol)/CH_3CN(2 mL), and TMSCN (77 μL, d = 0.793 g/mL, 60.7 mg, 0.6 mmol) sequentially under Ar atmosphere. The resulting mixture was irradiated with a 50 W 460 nm blue LED lamp (2-3 cm away, with cooling fan to keep the reaction temperature at 35~40 °C) for 24 h with stirring. The resulting mixture was filtrated through a short pad of silica gel eluted with ethyl ether (30 mL). After evaporation, 7

μL of CH_2Br_2 was added as the internal standard for the ^{1}H NMR analysis (10% of **2d** and 90% recovery of **1d**).

Radical trapping experiments

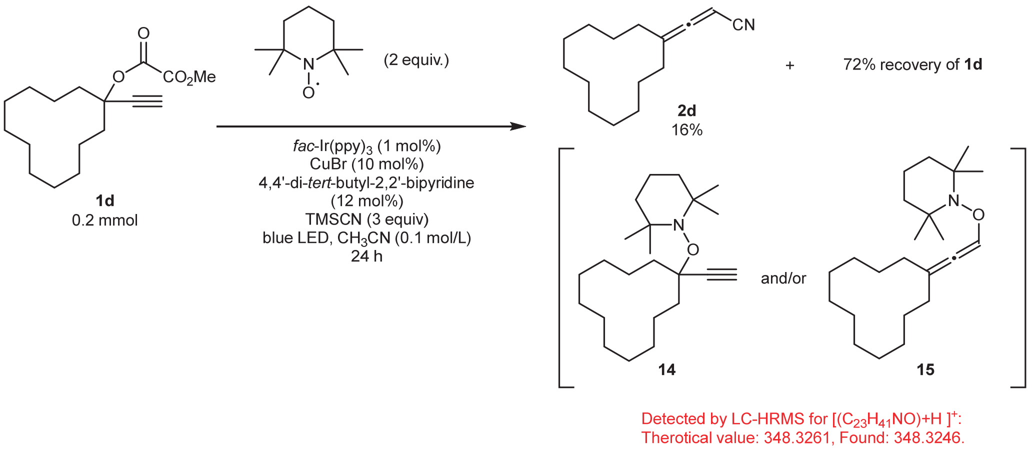

To a flame-dried Schlenk tube were added *fac*-Ir(ppy)₃ (1.3 mg, 2 μmol), CuBr (2.9 mg, 0.02 mmol), 4,4'-di-*tert*-butyl-2,2'-bipyridine (6.6 mg, 0.024 mmol), TEMPO (63.8 mg, 0.4 mmol), **1d** (59.2 mg, 0.2 mmol)/CH_3CN(1 mL), and TMSCN (61.0 mg, 0.6 mmol)/CH_3CN(1 mL) sequentially under Ar atmosphere. The resulting mixture was irradiated with a 50 W 460 nm blue LED lamp (2-3 cm away, with cooling fan to keep the reaction temperature at 35~40 °C) for 24 h with stirring. The resulting mixture was filtrated through a short pad of silica gel eluted with ethyl ether (30 mL). After evaporation, 7 μL of CH_2Br_2 was added as the internal standard for the ^{1}H NMR analysis (16% of **2d** and 72% recovery of **1d**).

For LC-HRMS analysis:

To a flame-dried Schlenk tube were added *fac*-Ir(ppy)$_3$ (1.3 mg, 2 μmol), CuBr (2.9 mg, 0.02 mmol), 4,4'-di-*tert*-butyl-2,2'-bipyridine (6.6 mg, 0.024 mmol), TEMPO (63.9 mg, 0.4 mmol), **1d** (59.0 mg, 0.2 mmol)/CH_3CN(1 mL), and TMSCN (61.5 mg, 0.6 mmol)/CH_3CN(1 mL) sequentially under Ar atmosphere. The resulting mixture was irradiated with a 50 W 460 nm blue LED lamp (2-3 cm away, with cooling fan to keep the reaction temperature at 35~40 °C) for 24 h with stirring. The resulting mixture was

analyzed by LC-HRMS directly, and the TEMPO-trappling product **14** and/or **15** could be detected. **HRMS** (ESI) calcd for $C_{23}H_{42}NO$ ([M+H]$^+$): 348.3261, Found: 348.3246.

Qualitative Analysis Report

**Data Filename**	LQ-9-184.d	**Sample Name**	LQ-9-184
**Sample Type**	Sample	**Position**	Vial 12
**Instrument Name**	Instrument 1	**User Name**	hp-PC\hp
**Acq Method**	pub-LOWP-2_2.m	**Acquired Time**	2/21/2022 4:47:07 PM
**IRM Calibration Status**	Success	**DA Method**	Default.m
**Comment**			

**Sample Group**		**Info.**
**Acquisition SW Version**	6200 series TOF/6500 series Q-TOF B.05.01 (B5125.3)	

User Spectra

Fragmentor Voltage	Collision Energy	Ionization Mode
75	0	ESI

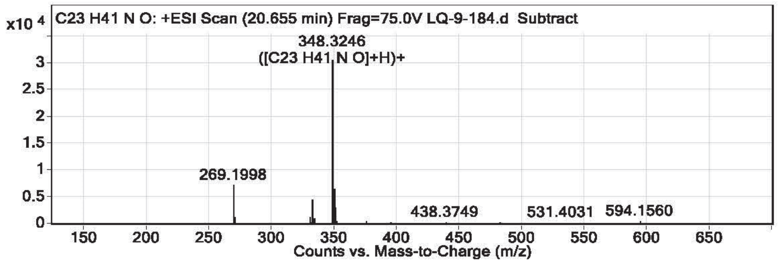

Peak List

m/z	z	Abund	Formula	Ion
348.3246	1	30598.65	C23 H41 N O	(M+H)+
349.3263	1	6621.79	C23 H41 N O	(M+H)+
350.337	1	3050.36	C23 H41 N O	(M+H)+
351.3418	1	544.62	C23 H41 N O	(M+H)+

Formula Calculator Element Limits

Element	Min	Max
C	3	60
H	0	120
O	1	10
N	1	5

Formula Calculator Results

Ion Formula	m/z	m/z (Calc)	DBE	Diff (ppm)	Score (MFG)
C23 H42 N O	348.3246	348.3261	4	4.29	93.66

--- End Of Report ---

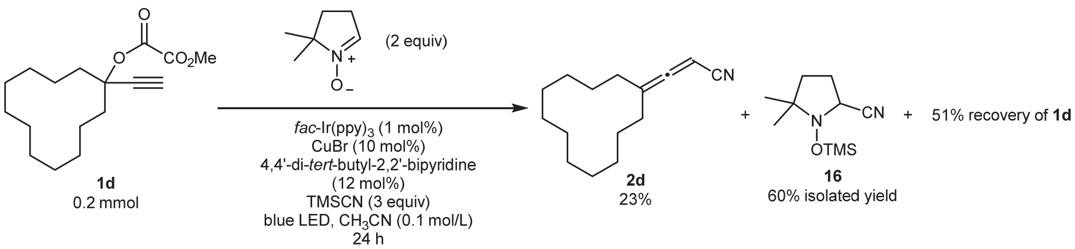

To a flame-dried Schlenk tube were added *fac*-Ir(ppy)$_3$ (1.3 mg, 2 μmol), CuBr (2.9 mg, 0.02 mmol), 4,4'-di-*tert*-butyl-2,2'-bipyridine (6.6 mg, 0.024 mmol), **1d** (59.1 mg, 0.2 mmol)/CH_3CN(1 mL), DMPO (47.2 mg, 0.4 mmol), and TMSCN (64.1 mg, 0.6 mmol)/CH_3CN(1 mL) sequentially under Ar atmosphere. The resulting mixture was irradiated with a 50 W 460 nm blue LED lamp (2-3 cm away, with cooling fan to keep the reaction temperature at 35~40 ºC) for 24 h with stirring. The resulting mixture was filtrated through a short pad of silica gel eluted with ethyl ether (30 mL). After evaporation, 7 μL of CH_2Br_2 was added as the internal standard for the ^{1}H NMR analysis (23% of **2d**, 60% of **16**, and 51% recovery of **1d**). **16** (25.5 mg, 60%) (using Biotage Isolera One purification system on flash silica gel column (Santai Tech. Inc., 12 g), flowrate: 25 mL/min, eluent: petroleum ether (80 mL), petroleum ether/ethyl ether = 20:1 (300 mL)) as a liquid: **^{1}H NMR** (400 MHz, $CDCl_3$) δ 4.14-3.51 (m, 1 H, NCH), 2.43-1.95 (m, 2 H, 2 × one proton CH_2), 1.83-1.60 (m, 2 H, 2 × one proton CH_2), 1.17 (s, 3 H, CH_3), 1.05 (s, 3 H, CH_3), 0.21 (s, 9 H, $Si(CH_3)_3$); **^{13}C NMR** (100 MHz, $CDCl_3$) δ 120.6, 65.5, 56.2, 34.5, 26.9, 25.1, 18.6, 0.1; **MS** (EI) *m/z* (%) 212 (M^+, 12.75), 197 (100); **IR** (neat): ν = 2968, 2877, 2247, 1464, 1381, 1368, 1319, 1250, 1170, 1008 cm^{-1}; **HRMS** (EI) calcd for $C_{10}H_{20}N_2OSi$ (M^+): 212.1339, Found: 212.1339.

Supplementary table for photocatalyst screening

Supplementary Table 2. Details for photocatalyst screening

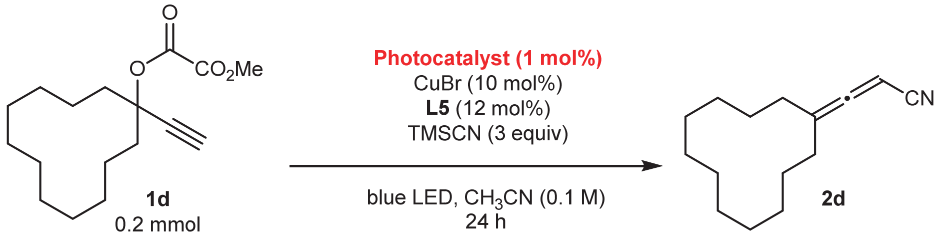

Entry	Photocatalyst	Wavelength of light (nm)	E_T (kcal/mol)[a]	Yield of **2d**[b]	Recovery of **1d**[c]
1	$Ru(bpy)_3Cl_2{\bullet}6H_2O$	460	46.5	0	100
2	$Ru(bpz)_3(PF_6)_2$	460	48.4	0	100
3	$Ir(dtbbpy)(ppy)_2PF_6$	460	49.2	0	100
4	*fac*-$Ir(ppy)_3$	460	57.8	87[b]	0
5	$Ir[dF(CF_3)ppy]_2(dtbbpy)PF_6$	460	60.8	0	100
6	Michler's ketone (5 mol%)	460	61.0	0	100
7	Michler's ketone (5 mol%)	395	61.0	33	38
8	Thioxanthone (5 mol%)	460	65.4	0	100
9	Thioxanthone (5 mol%)	395	65.4	13	83

[a] Data were taken from Ref 14 and 15.

[b] Determined by ^1H NMR analysis with CH_2Br_2 as the internal standard.

[c] Isolated yield in 0.5 mmol scale reaction.

For Michler's ketone (entry 7), 33% of **2d** was produced under 390 nm wavelength irradiation, which could be attributed to the high reducing ability of the excited photocatalyst ($E_{1/2}^{red}$ [MK^+/MK] = -2.12 V vs SCE in CH_3CN, MK represents Michler's ketone).[16]

Computational studies

Computational method

All calculations were performed with the Gaussian 09 program.[17] Geometries have been fully optimized with the density functional theory of (U)B3LYP method,[18] including Grimme's D3 dispersion corrections ((U)B3LYP-D3 with Becke-Johnson damping).[19,20] The standard 6-31G(d,p)[21-23] basis set (LANL2DZ[24,25] basis set for Cu) was used for the geometry optimizations. Harmonic vibration frequency calculations were conducted at the same level of theory to verify the stationary points to be minima (no imaginary frequency) or saddle points (one imaginary frequency). Intrinsic reaction coordinate (IRC)[26-28] calculations were performed to confirm the connection of the transition structures with their corresponding reactants and products. The solvent effects were determined by single-point calculations of the gas-phase stationary points at (U)M06[29,30]/SDD[31]-6-311++G(d,p) level by using SMD[32] solvation model. The reported energies are the solution-phase Gibbs free energies (ΔG_{sol}) in acetonitrile (ε = 35.688).

Energies of intermediates and transition states

Supplementary Table 3**.** Electronic energies (E_{elec}), Gibbs free energies (G_{298}), thermal correction to Gibbs free energy ($cor\ G_{gas}$), solvation energies (E_{sol}), solvation free energies (G_{sol}) in acetonitrile (ε = 35.688) for all stationary points of this process.

species	Eelec (a.u.)	G298 (a.u.)	cor Ggas (a.u.)	Esol (a.u.)	Gsol (a.u.)
**Int1**	-350.755721	-350.596262	0.159459	-350.515384	-350.355925
**$LCu^{II}(CN)2$**	-877.302846	-877.171941	0.130905	-878.197561	-878.066656
**TS1_a**	-1228.074897	-1227.761174	0.313724	-1228.720197	-1228.406473
**Int2_a**	-1228.081287	-1227.766843	0.314444	-1228.729941	-1228.415497
**TS2_a**	-1228.081268	-1227.765827	0.315441	-1228.729385	-1228.413944
**2B**	-443.651446	-443.481839	0.169607	-443.370224	-443.200617
**$LCu^{I}CN$**	-784.49249	-784.368961	0.123529	-785.419892	-785.296363
**TS_a**	-1228.04555	-1227.733197	0.312353	-1228.686702	-1228.374349
**TS1_b**	-1228.065938	-1227.748957	0.316981	-1228.71577	-1228.398789
**Int2_b**	-1228.066443	-1227.750151	0.316292	-1228.717786	-1228.401494
**TS2_b**	-1228.065873	-1227.749808	0.316065	-1228.714473	-1228.398408
**3B**	-443.638513	-443.470252	0.168262	-443.366302	-443.19804
**TS_b**	-1228.046329	-1227.731806	0.314523	-1228.685554	-1228.371031

NMR and HPLC spectra

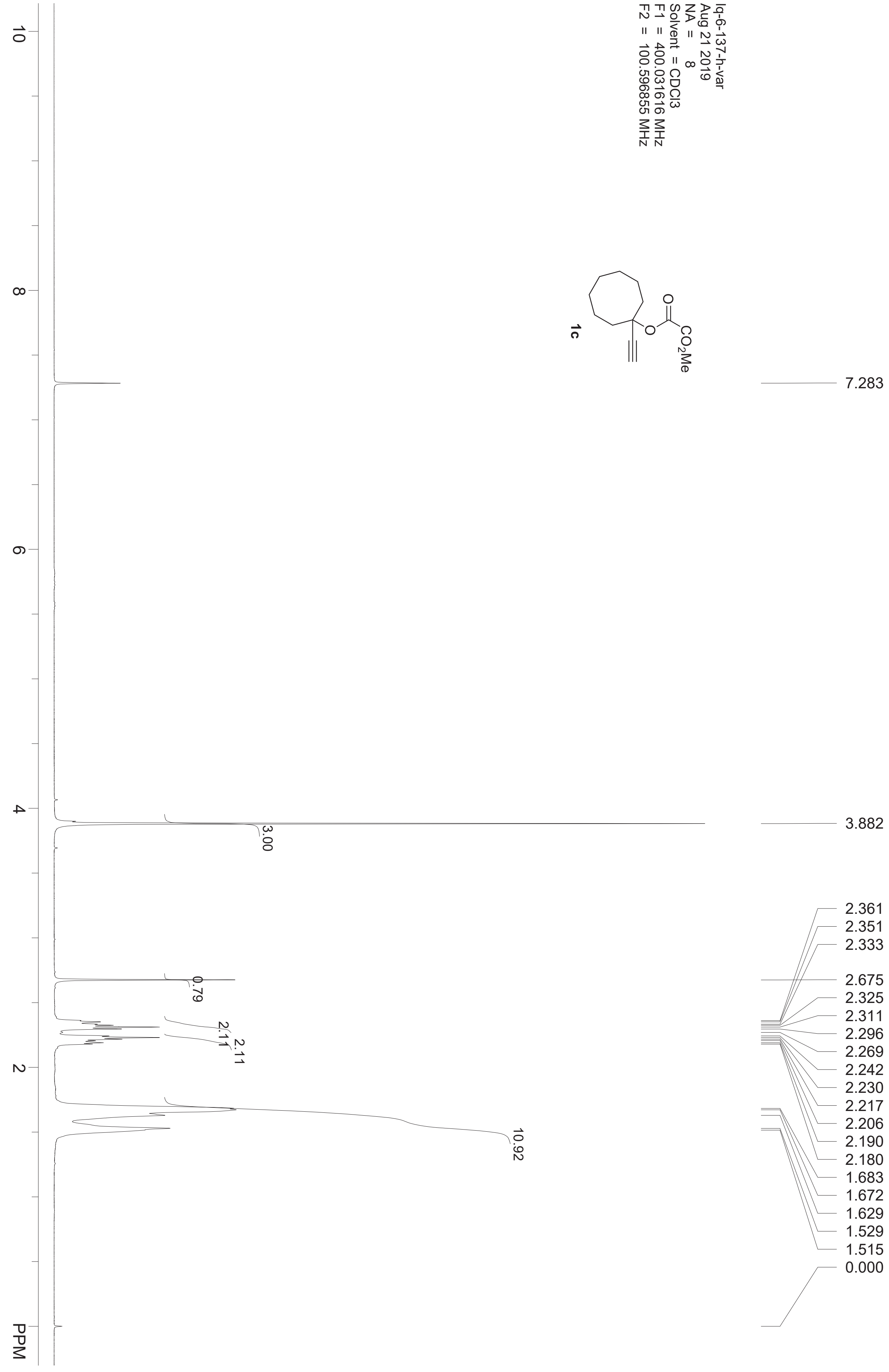

Supplementary Figure 7. ^{1}H NMR (400 MHz, $CDCl_3$) spectrum for **1c**

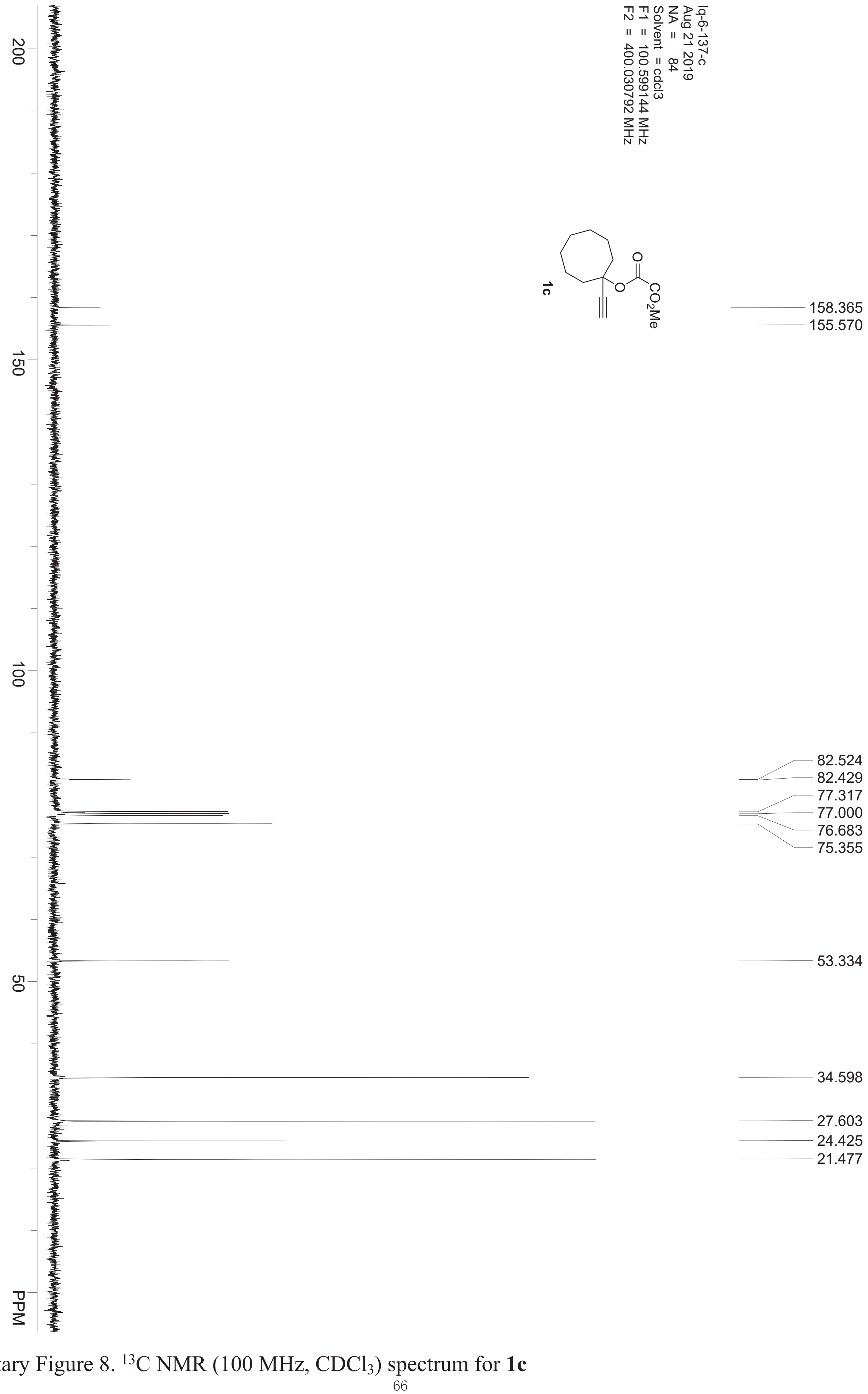

Supplementary Figure 8. ^{13}C NMR (100 MHz, $CDCl_3$) spectrum for **1c**

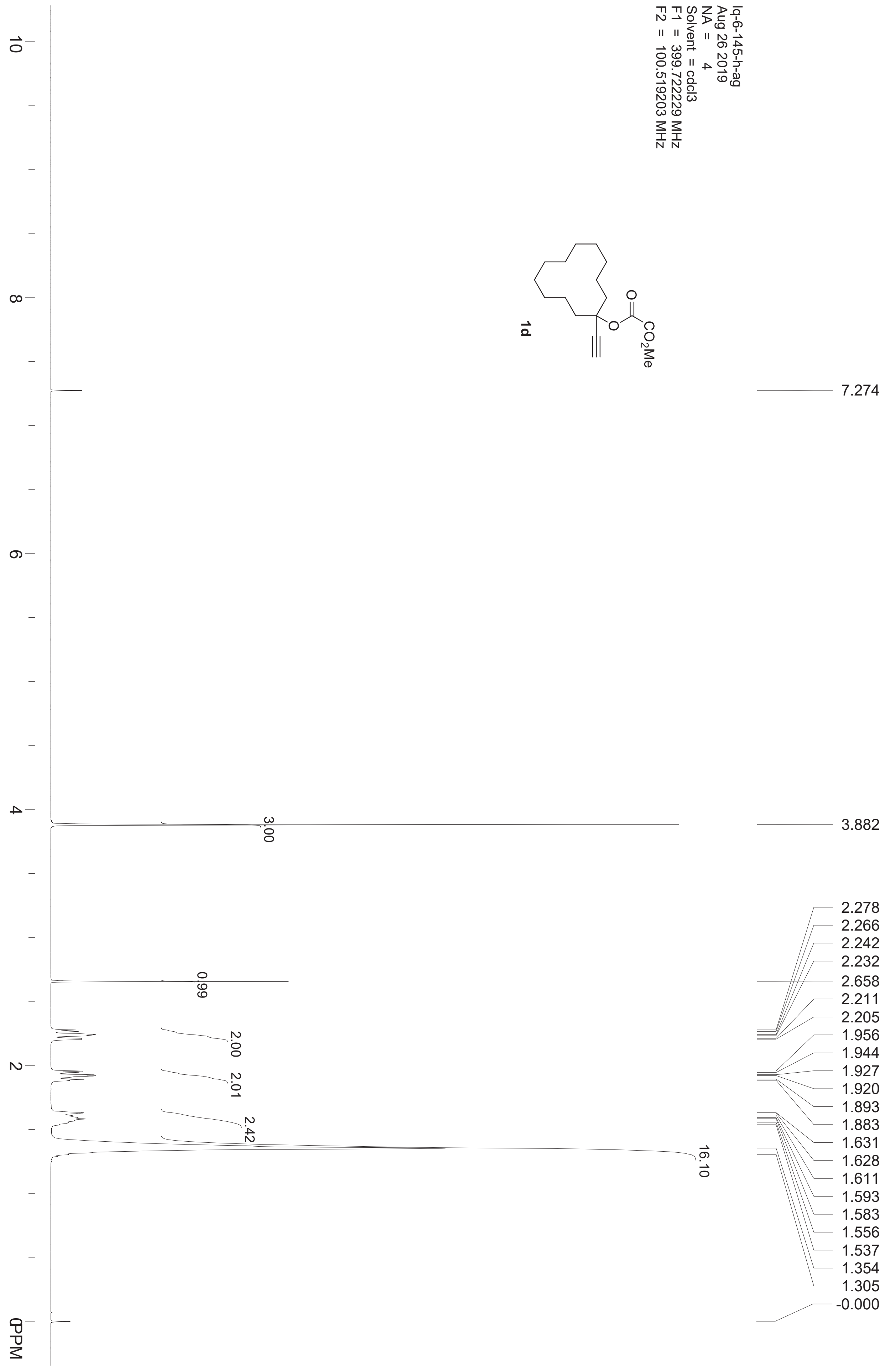

Supplementary Figure 9. ^{1}H NMR (400 MHz, $CDCl_3$) spectrum for **1d**

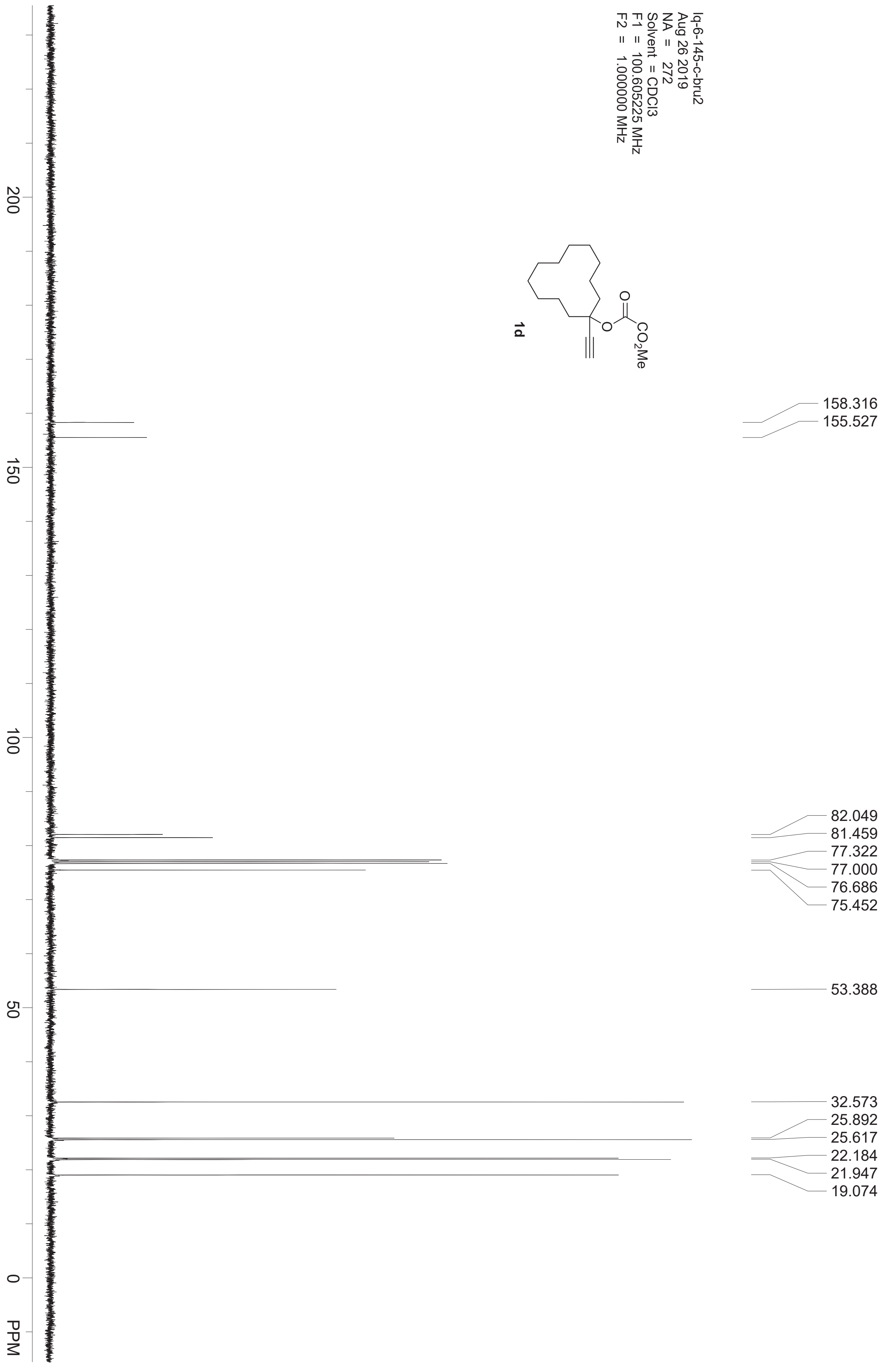

Supplementary Figure 10. ^{13}C NMR (100 MHz, $CDCl_3$) spectrum for **1d**

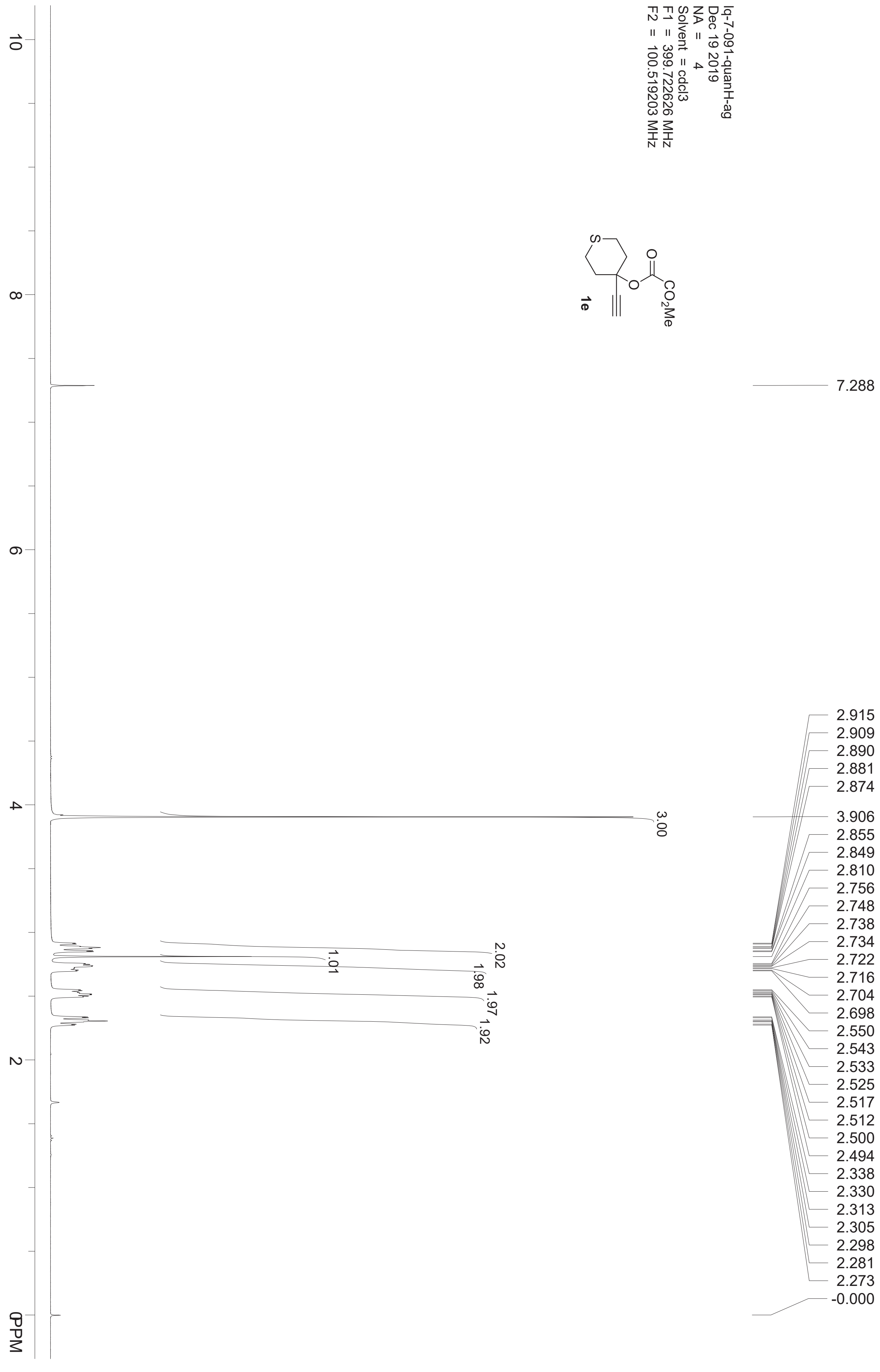

Supplementary Figure 11. ^{1}H NMR (400 MHz, $CDCl_3$) spectrum for **1e**

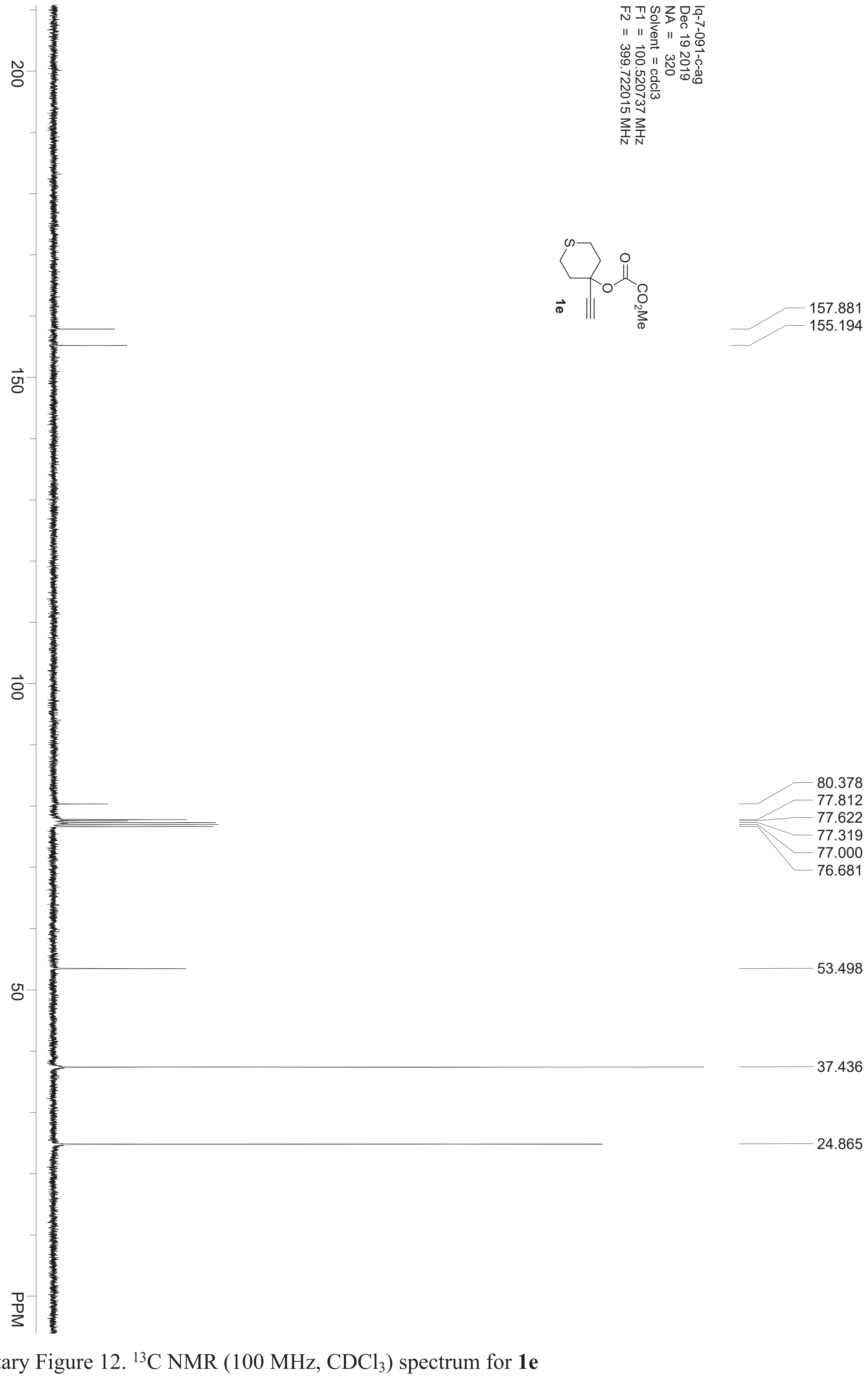

Supplementary Figure 12. ^{13}C NMR (100 MHz, $CDCl_3$) spectrum for **1e**

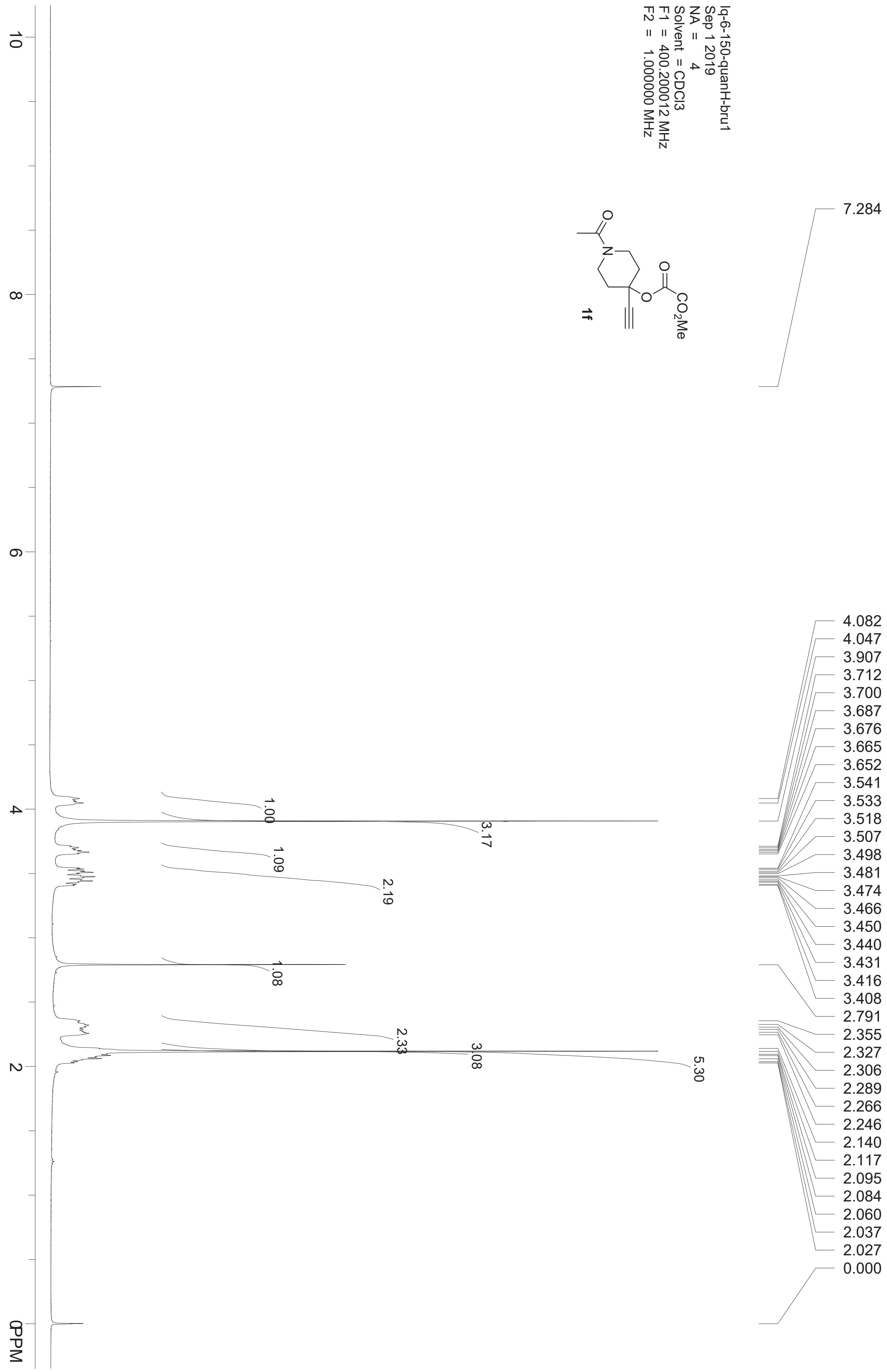

Supplementary Figure 13. ^{1}H NMR (400 MHz, $CDCl_3$) spectrum for **1f**

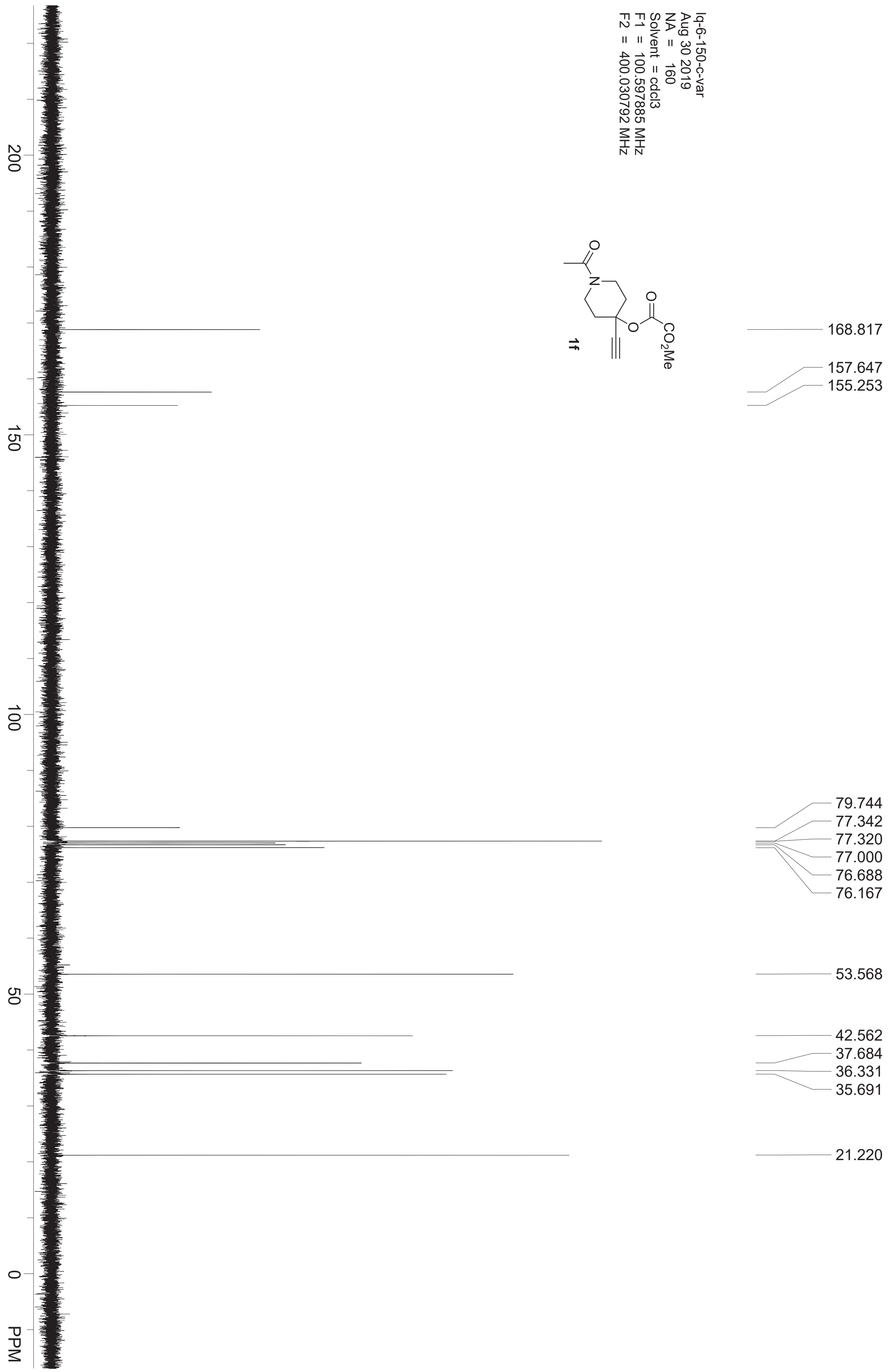

Supplementary Figure 14. ^{13}C NMR (100 MHz, $CDCl_3$) spectrum for **1f**

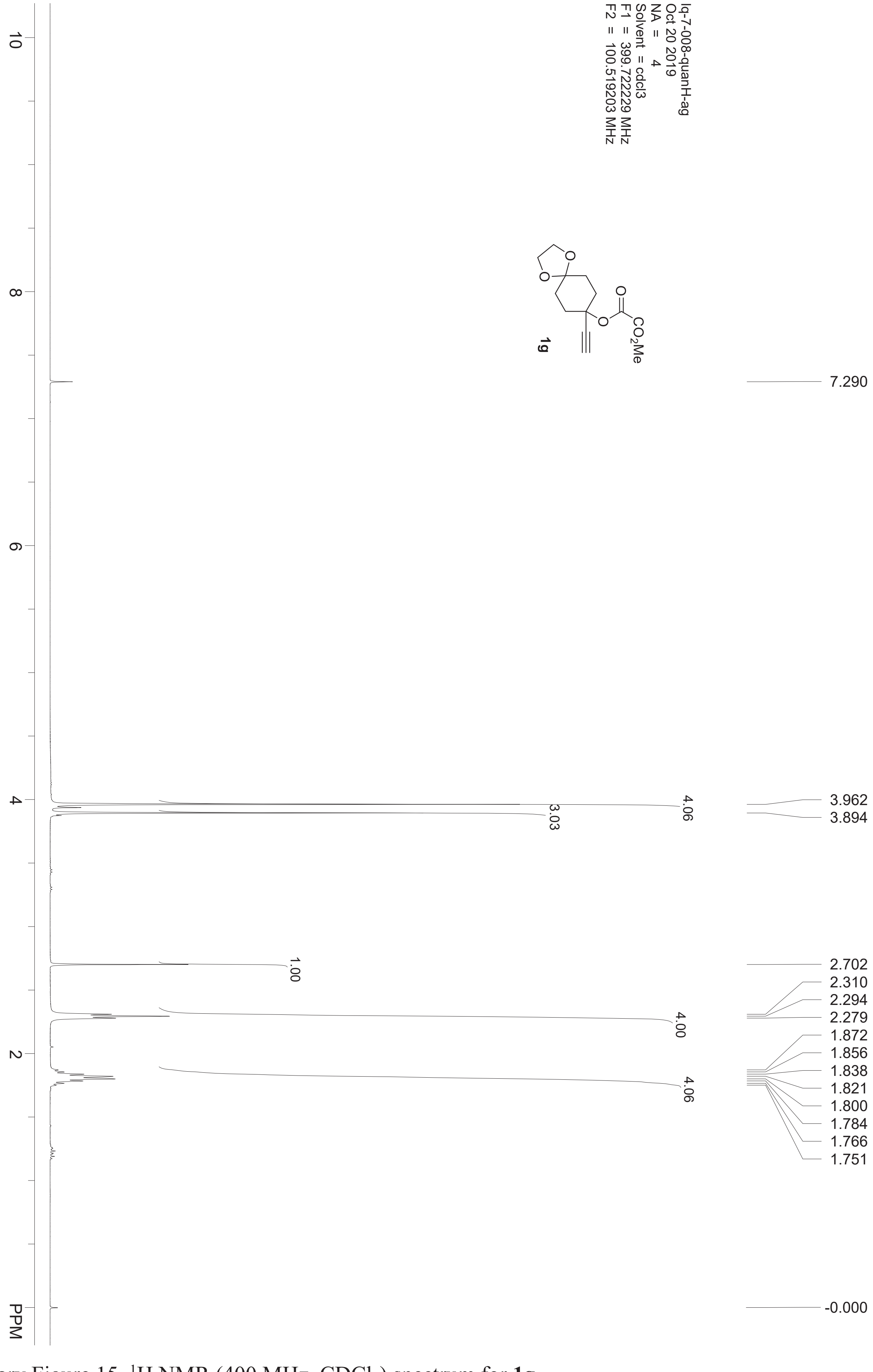

Supplementary Figure 15. ^{1}H NMR (400 MHz, $CDCl_3$) spectrum for **1g**

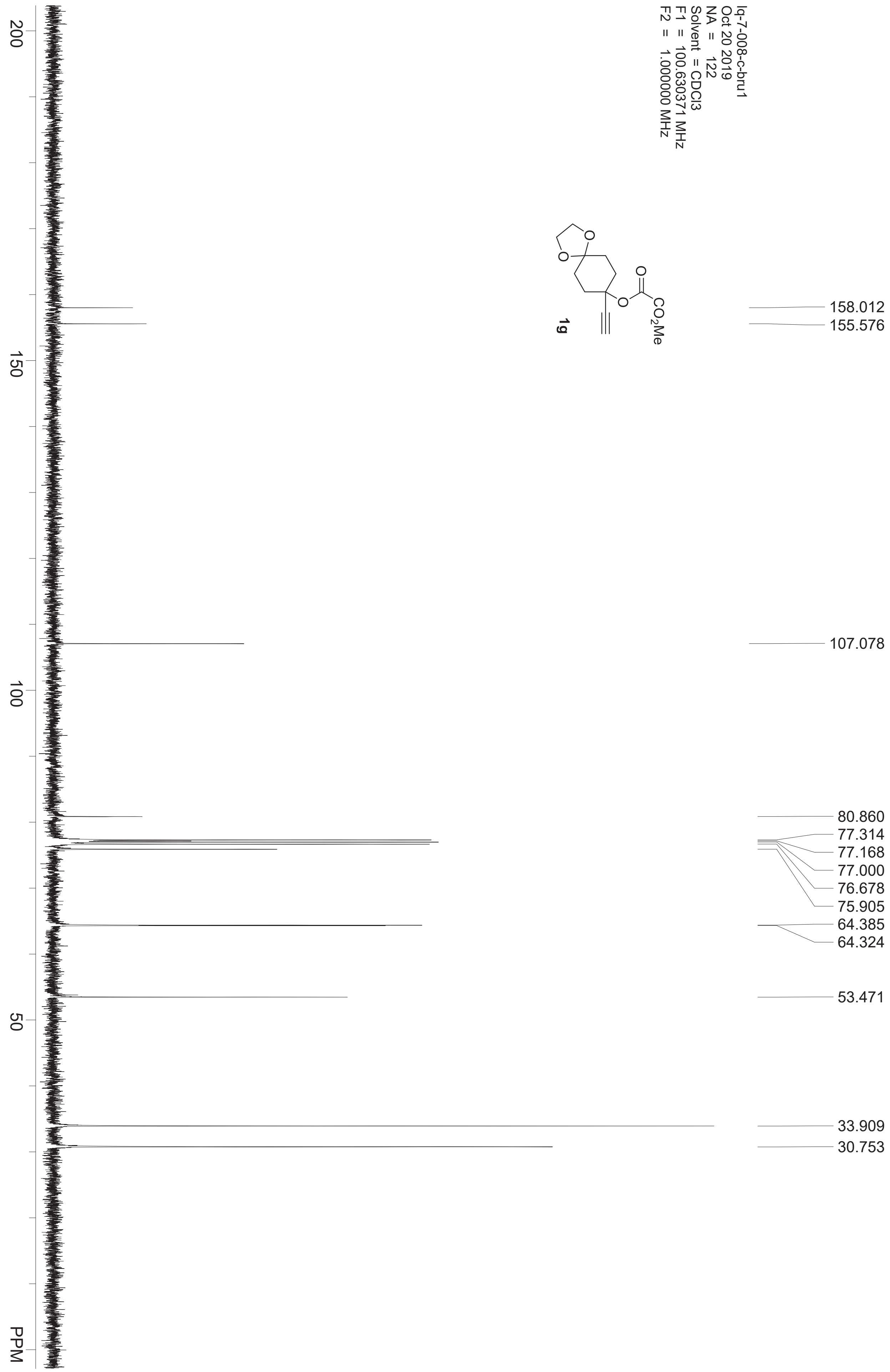

Supplementary Figure 16. ^{13}C NMR (100 MHz, CDCl3) spectrum for **1g**

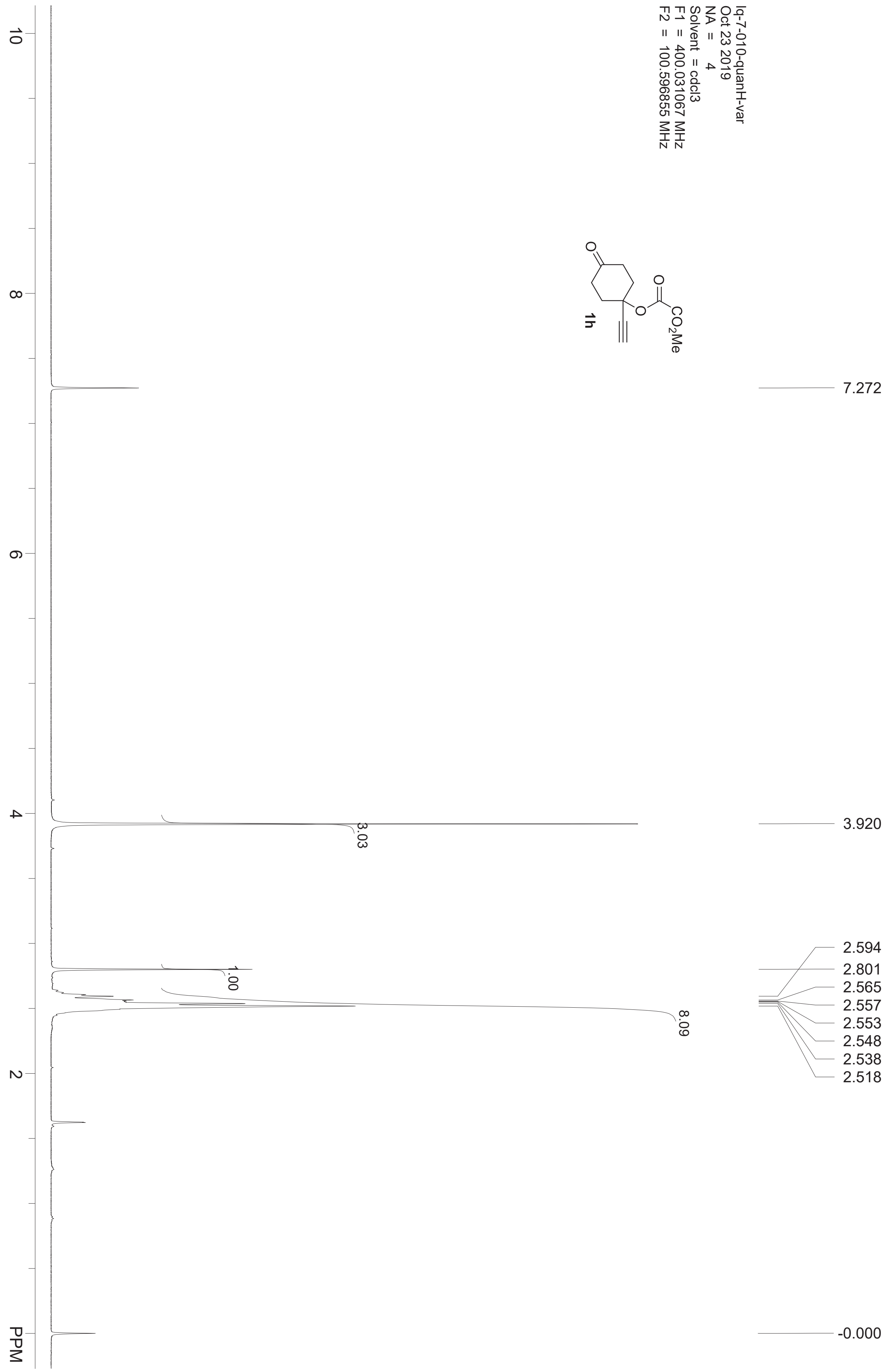

Supplementary Figure 17. ^{1}H NMR (400 MHz, $CDCl_3$) spectrum for **1h**

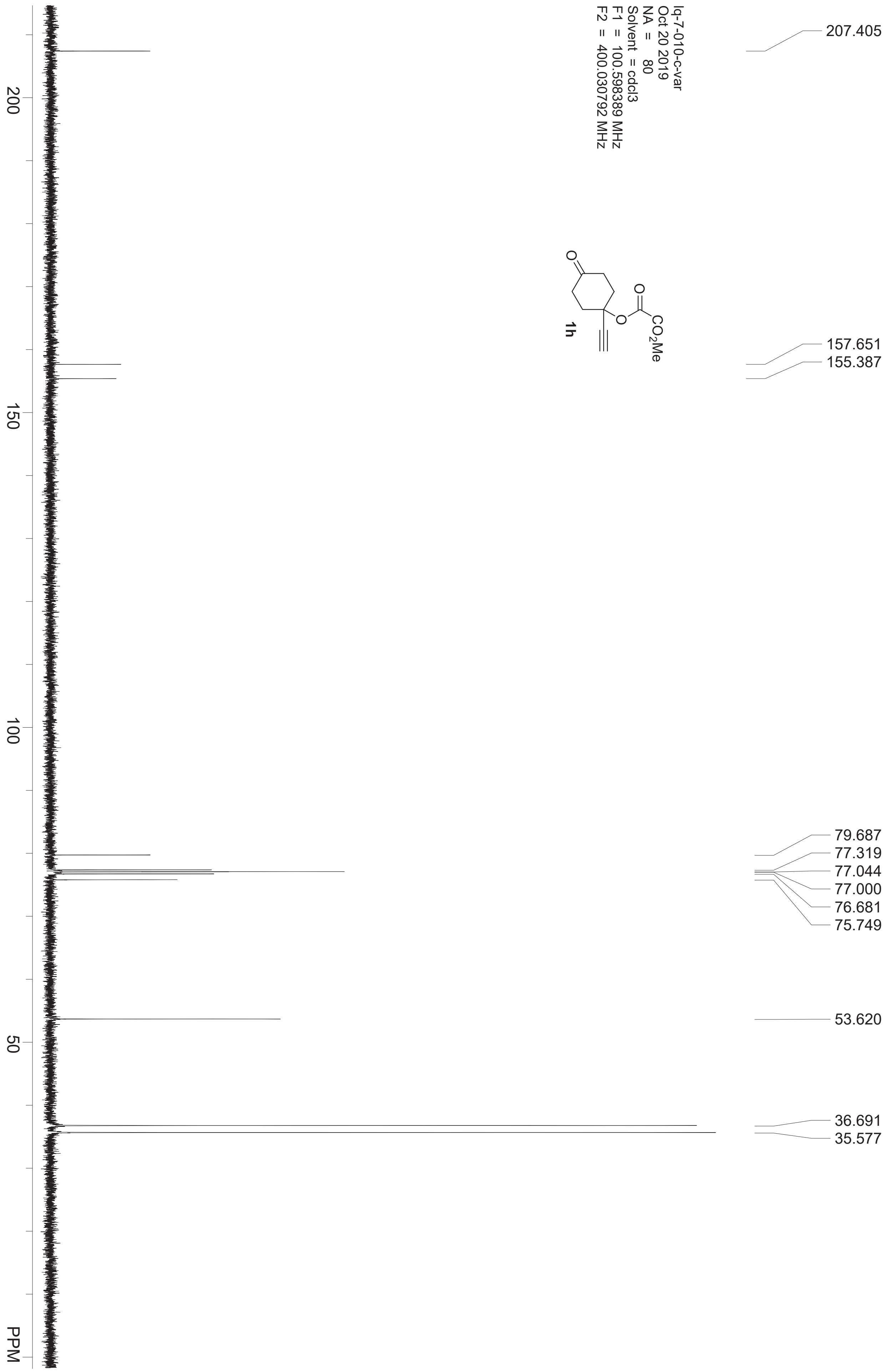

Supplementary Figure 18. ^{13}C NMR (100 MHz, $CDCl_3$) spectrum for **1h**

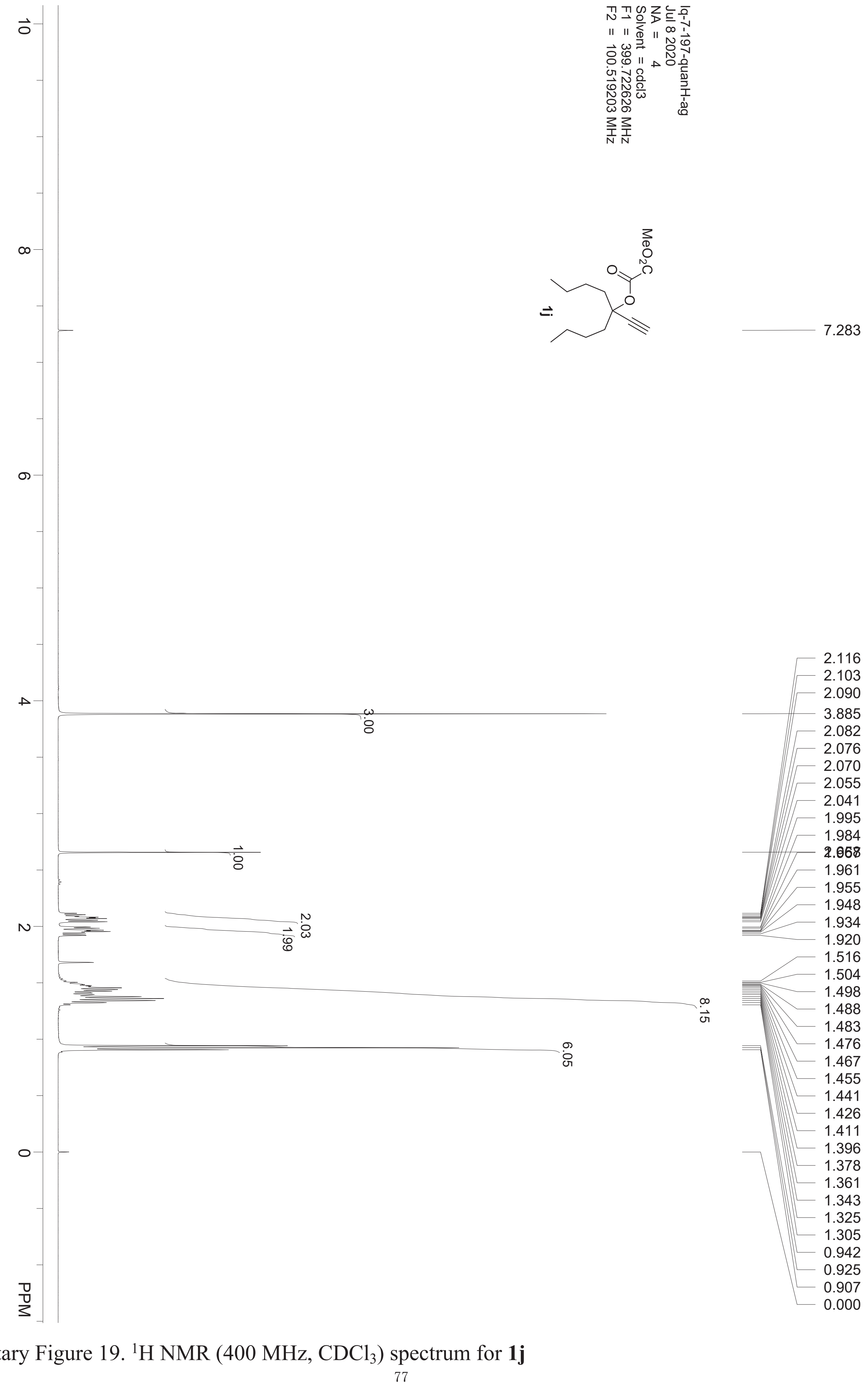

Supplementary Figure 19. ^{1}H NMR (400 MHz, $CDCl_3$) spectrum for **1j**

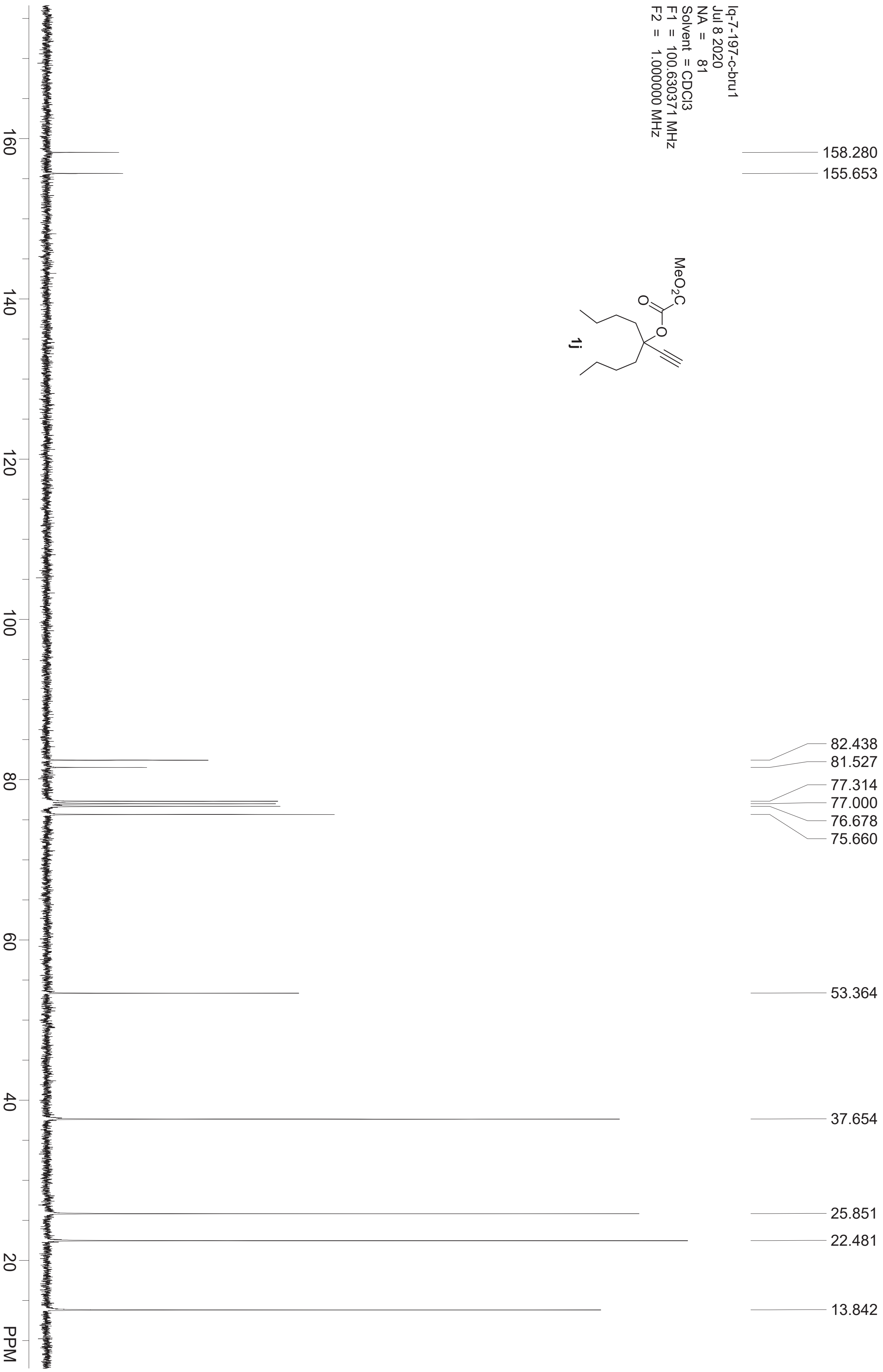

Supplementary Figure 20. ^{13}C NMR (100 MHz, CDCl3) spectrum for **1j**

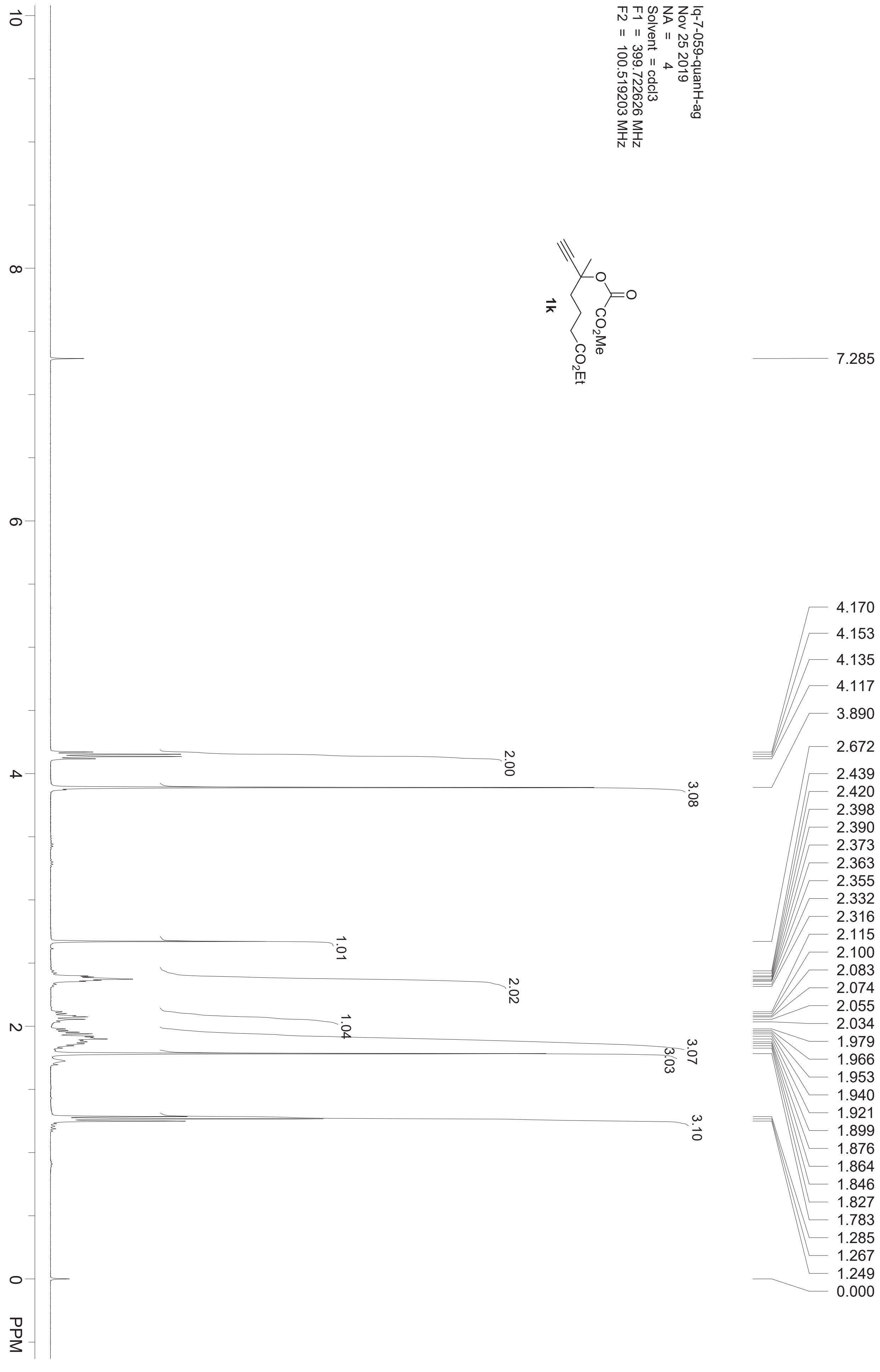

Supplementary Figure 21. ^{1}H NMR (400 MHz, $CDCl_3$) spectrum for **1k**

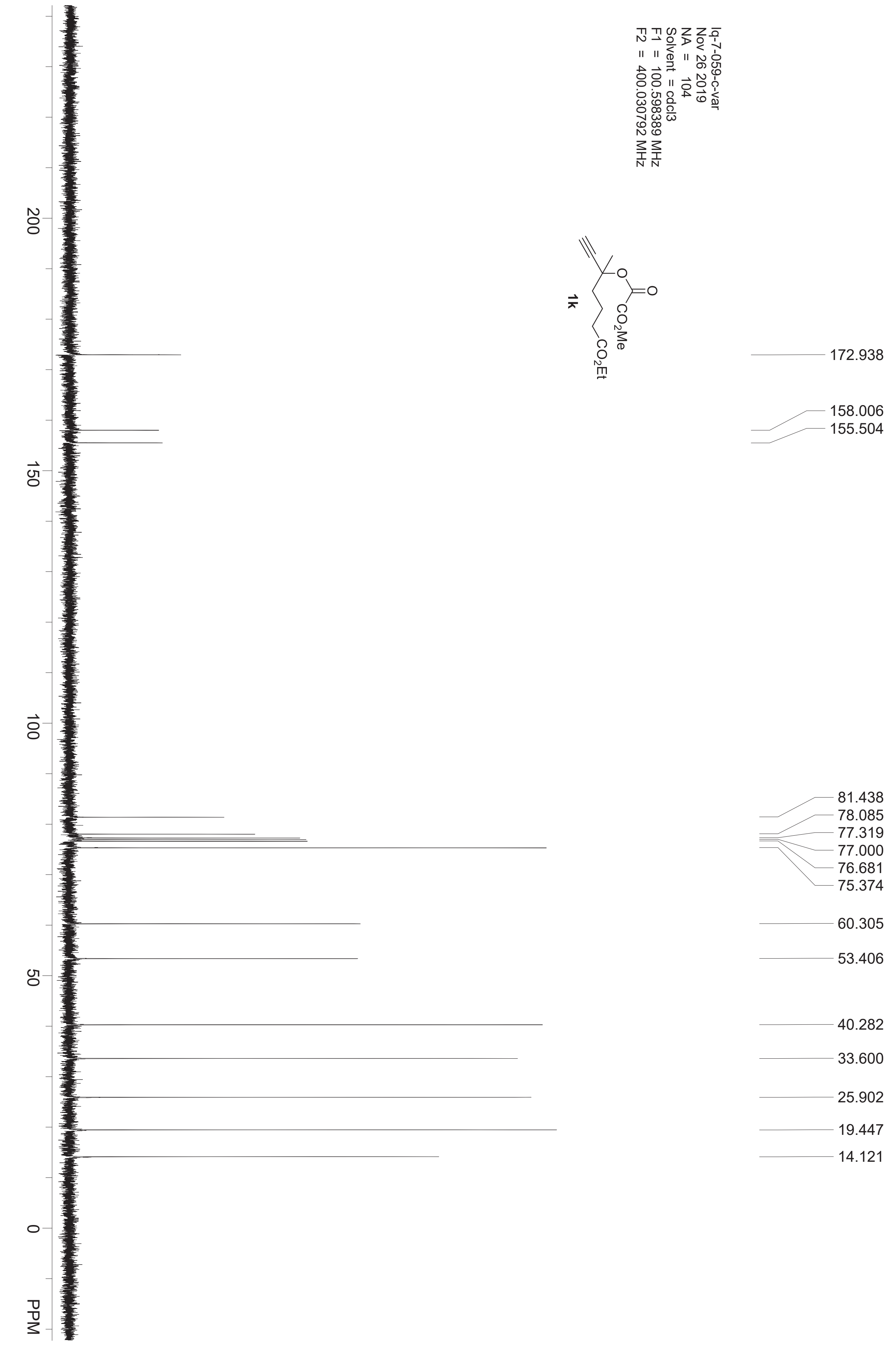

Supplementary Figure 22. ^{13}C NMR (100 MHz, $CDCl_3$) spectrum for **1k**

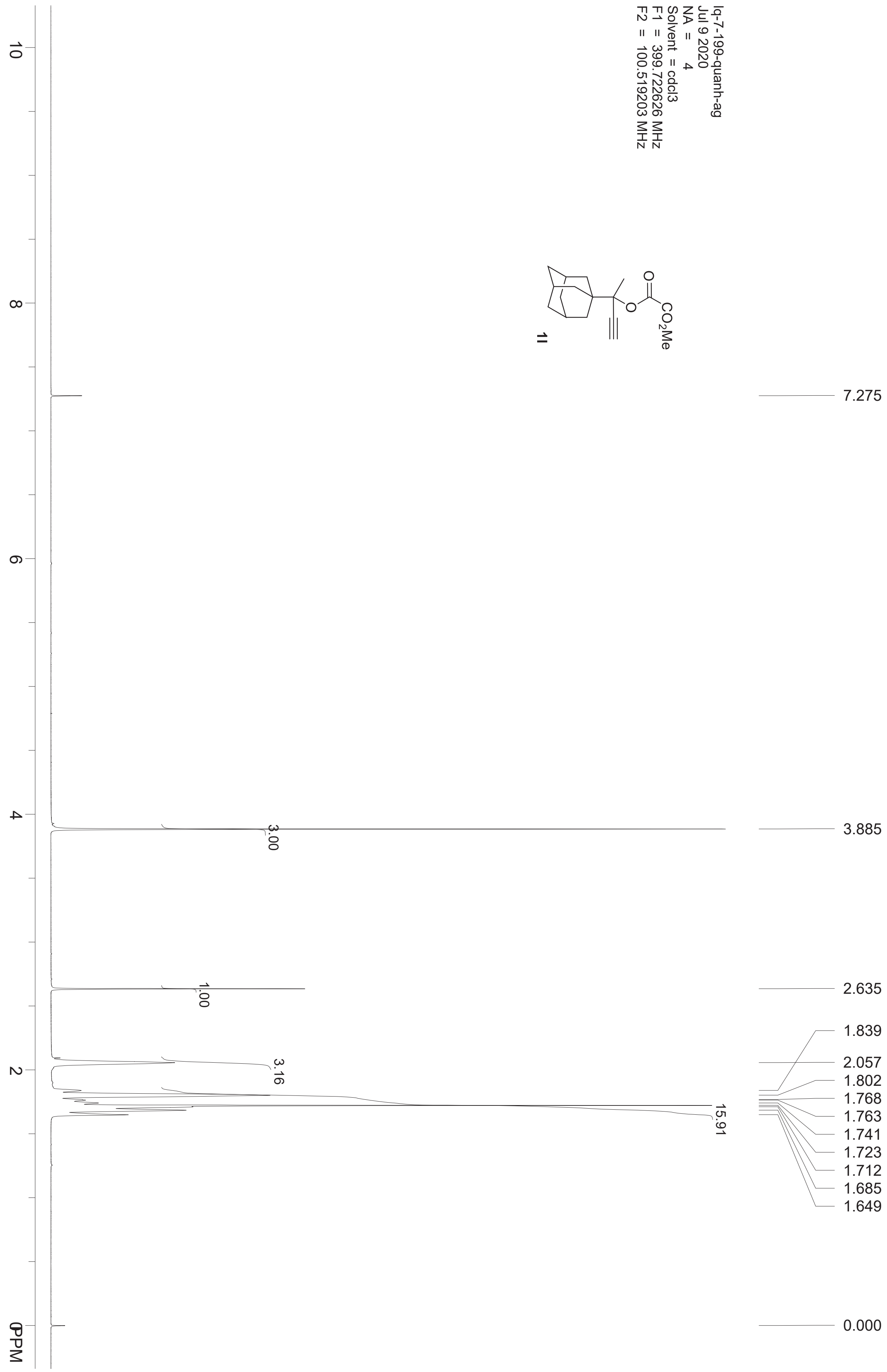

Supplementary Figure 23. ^{1}H NMR (400 MHz, $CDCl_3$) spectrum for **1l**

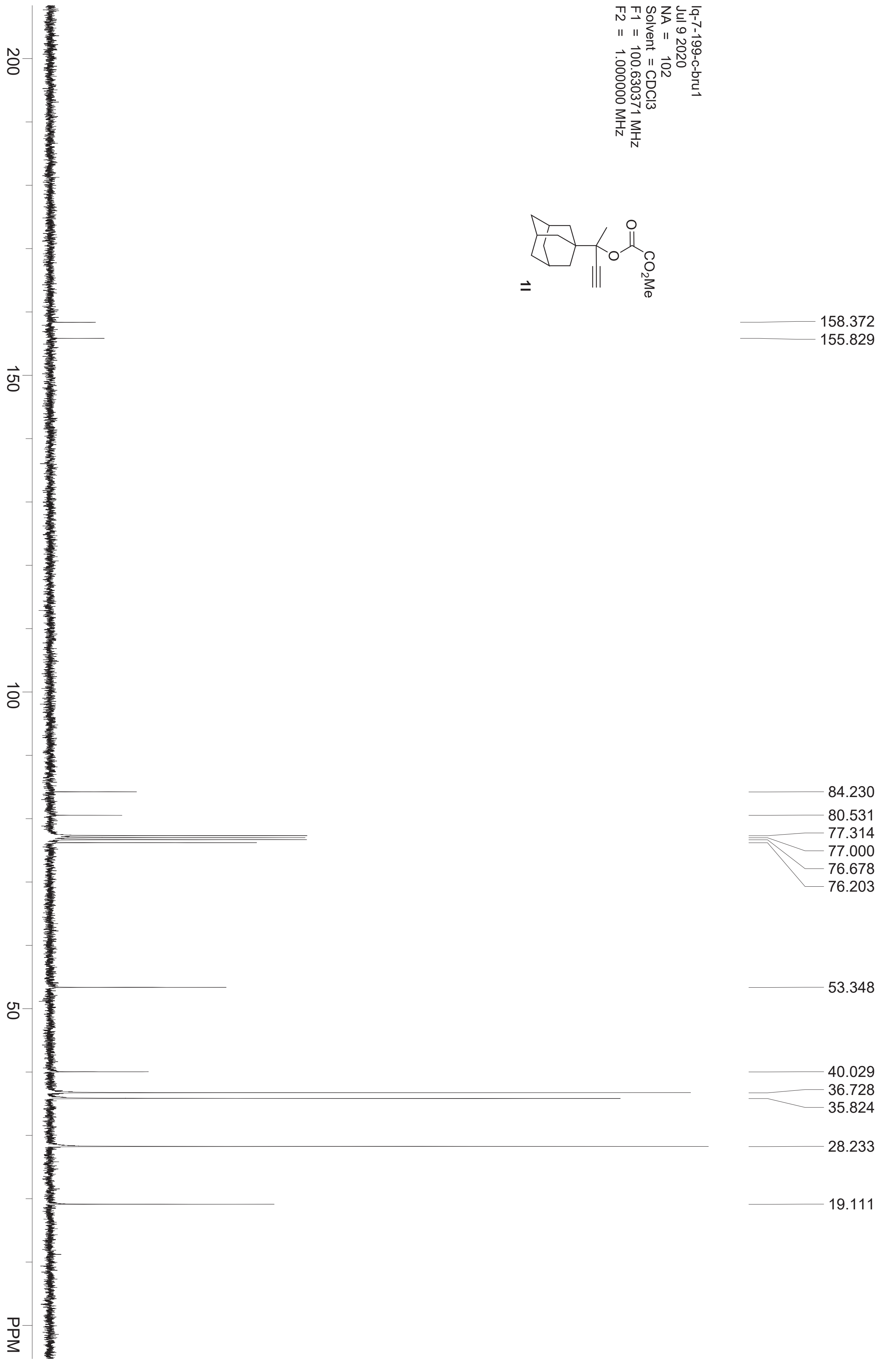

Supplementary Figure 24. ^{13}C NMR (100 MHz, $CDCl_3$) spectrum for **1l**

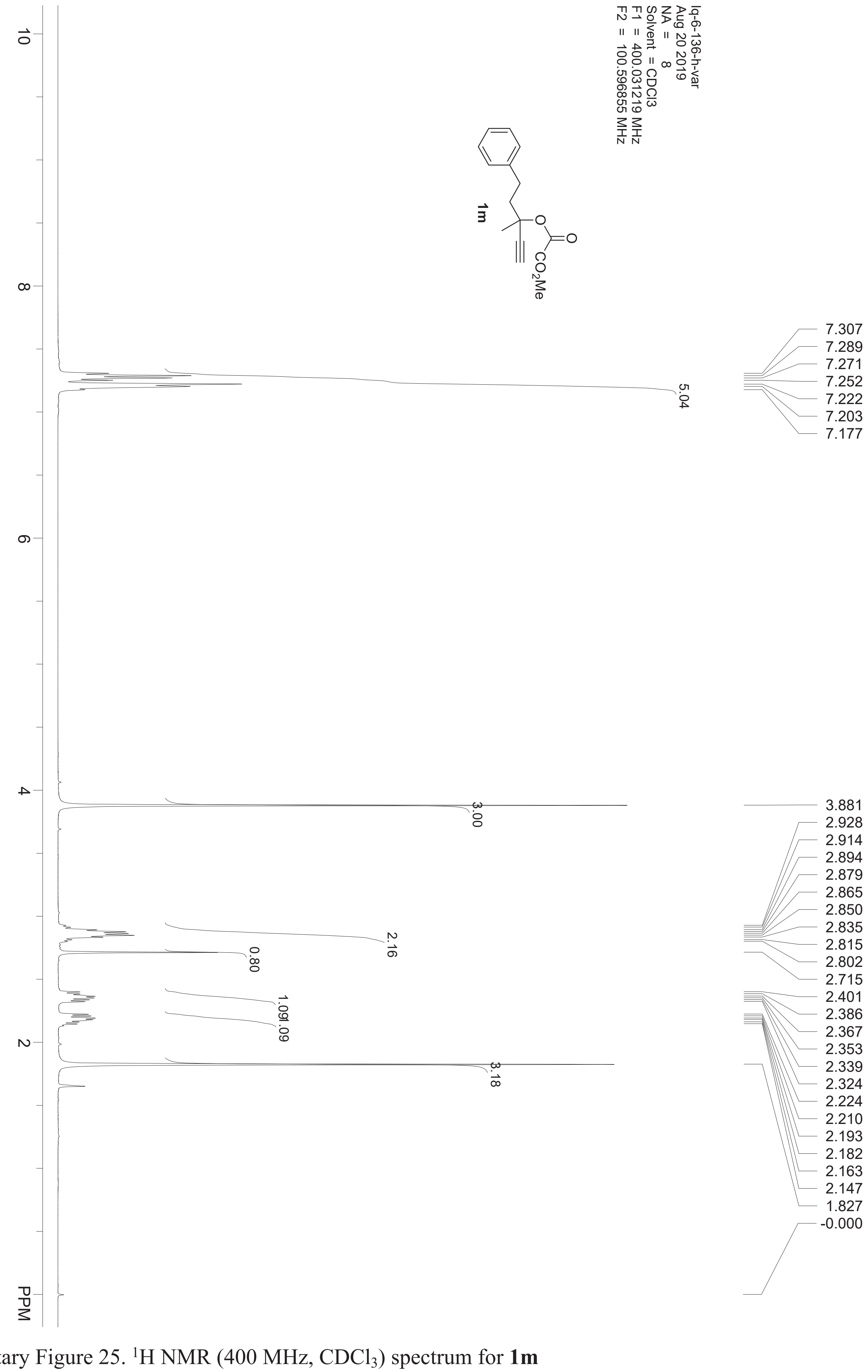

Supplementary Figure 25. ^{1}H NMR (400 MHz, $CDCl_3$) spectrum for **1m**

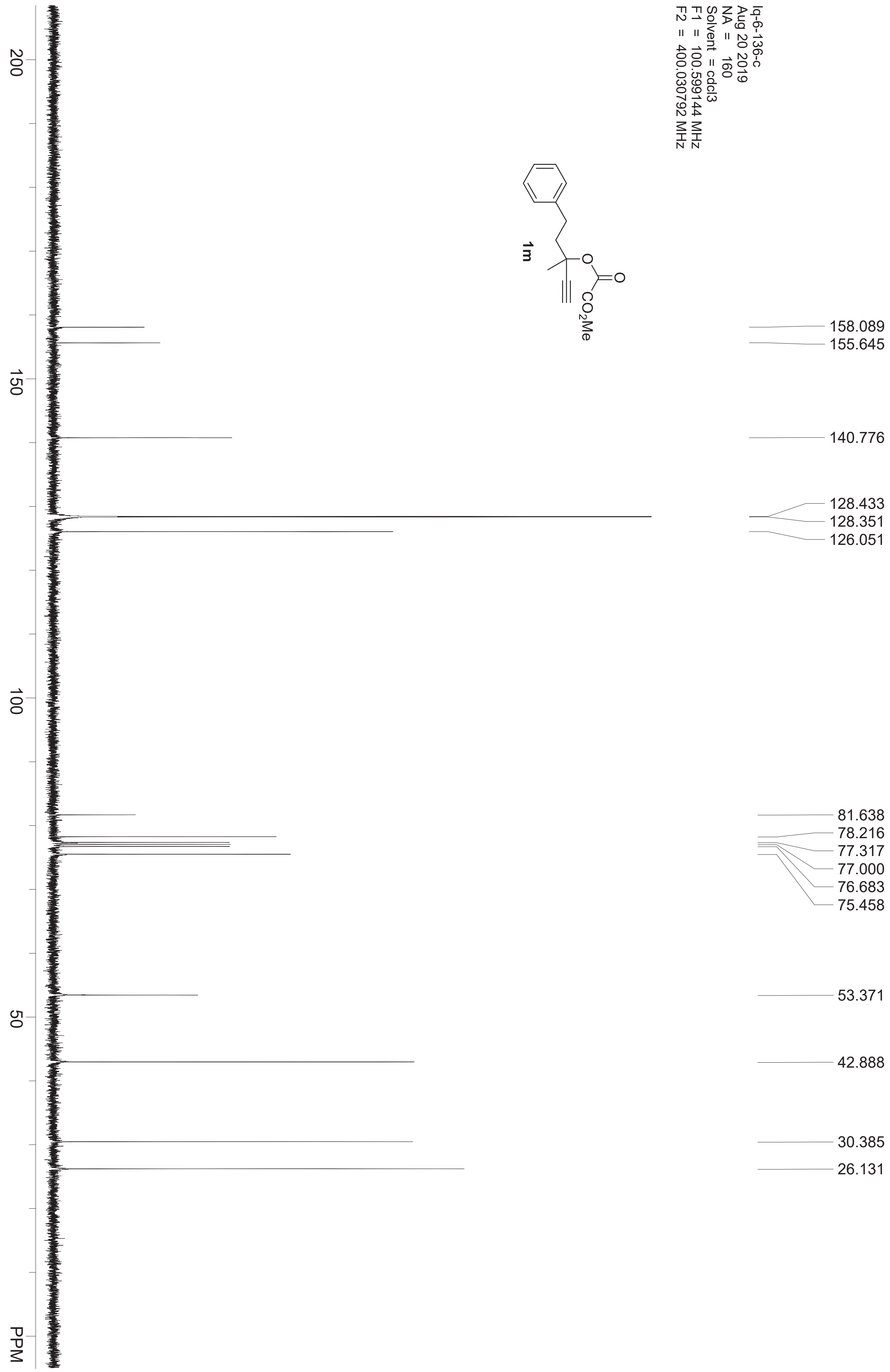

Supplementary Figure 26. ^{13}C NMR (100 MHz, $CDCl_3$) spectrum for **1m**

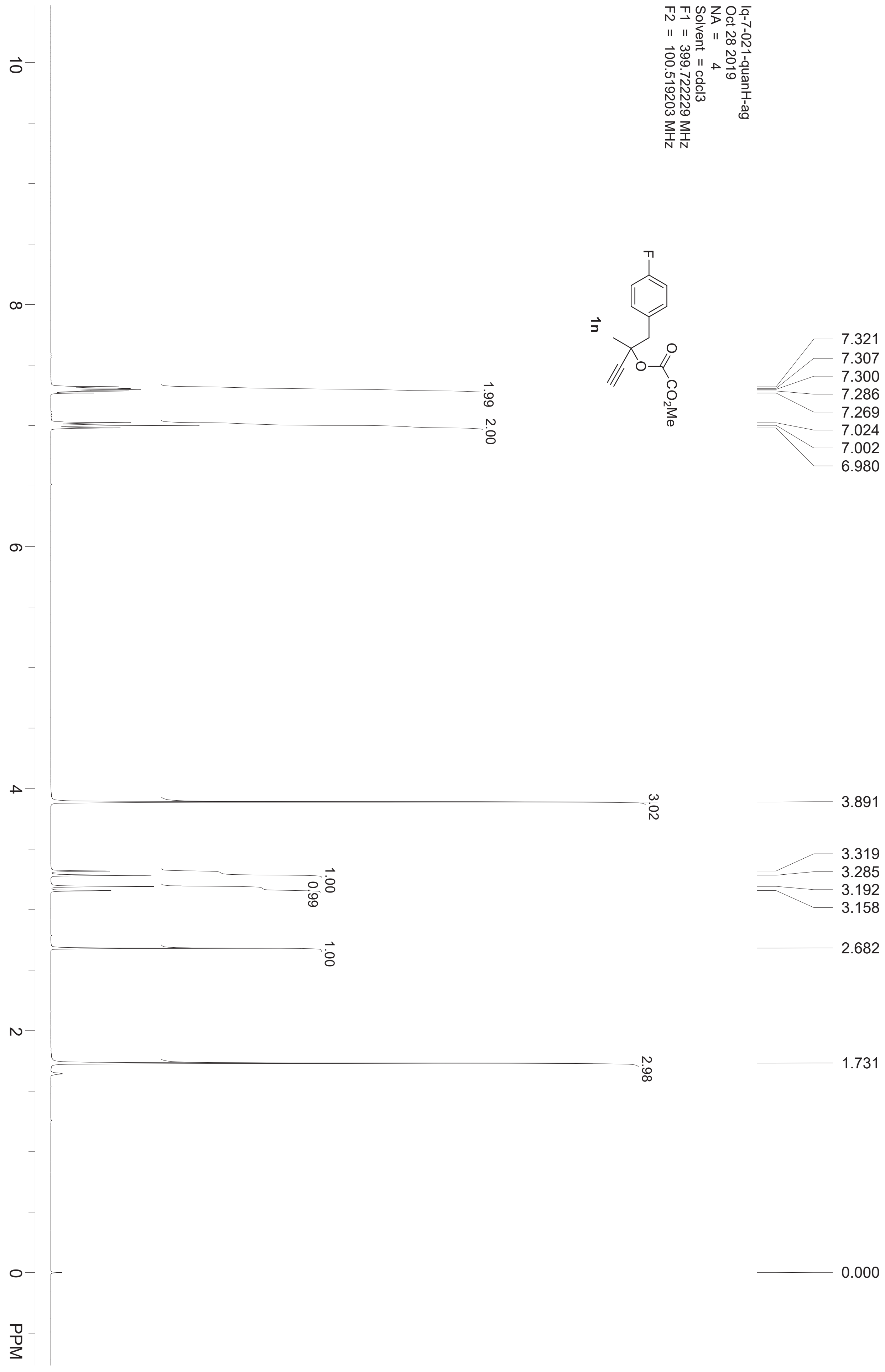

Supplementary Figure 27. ^{1}H NMR (400 MHz, $CDCl_3$) spectrum for **1n**

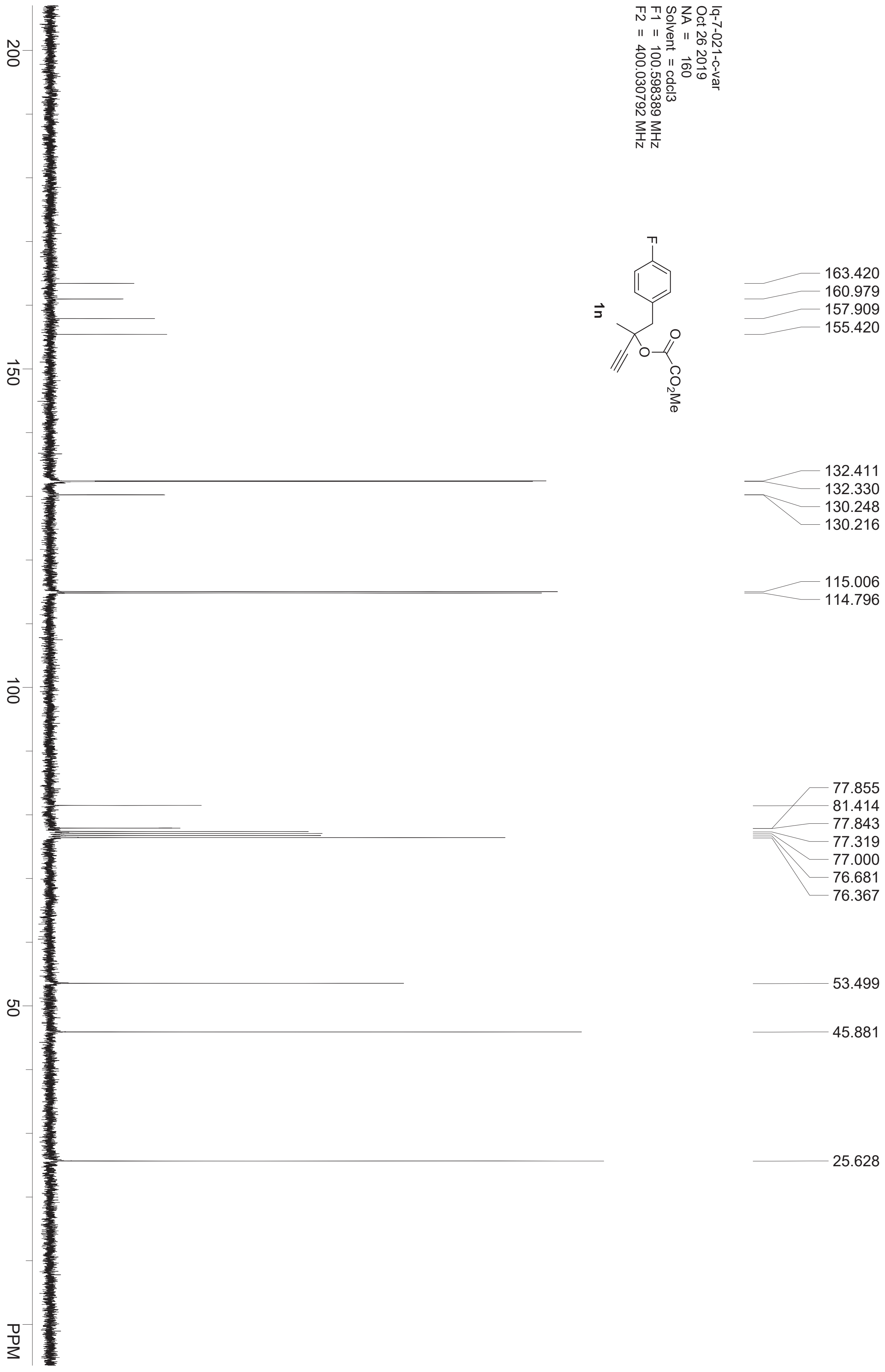

Supplementary Figure 28. ^{13}C NMR (100 MHz, $CDCl_3$) spectrum for **1n**

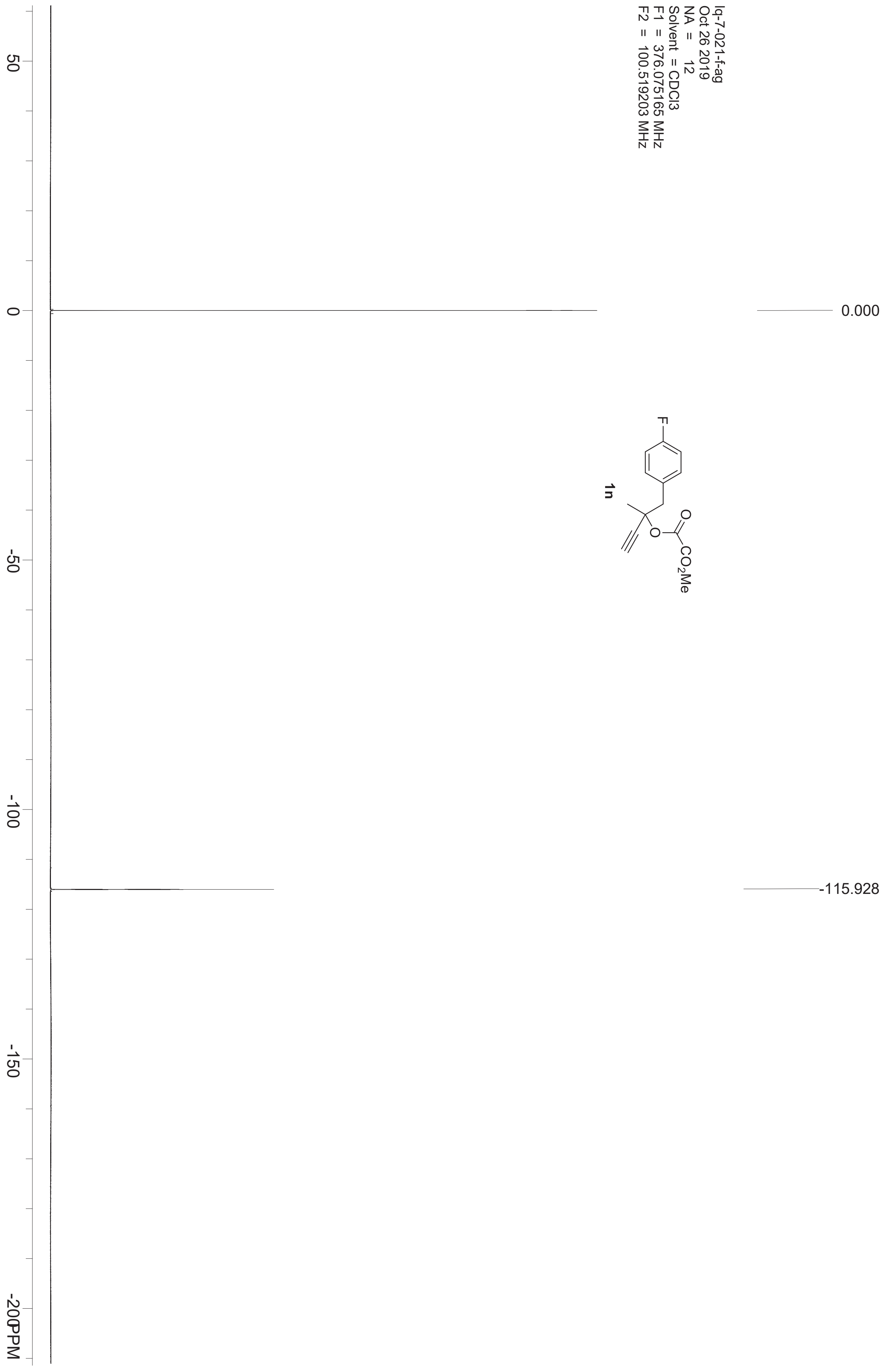

Supplementary Figure 29. ^{19}F NMR (376 MHz, $CDCl_3$) spectrum for **1n**

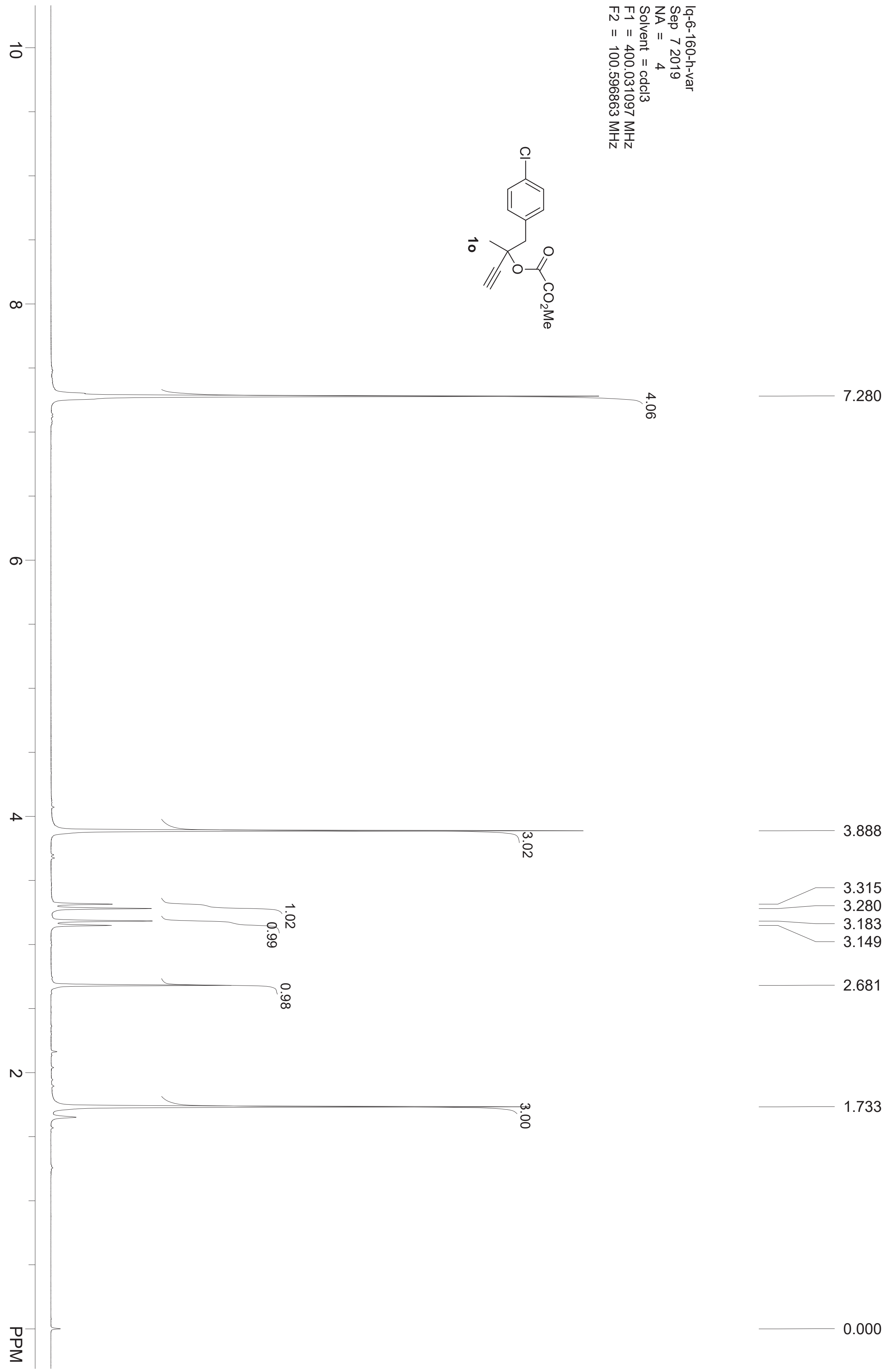

Supplementary Figure 30. ^{1}H NMR (400 MHz, $CDCl_3$) spectrum for **1o**

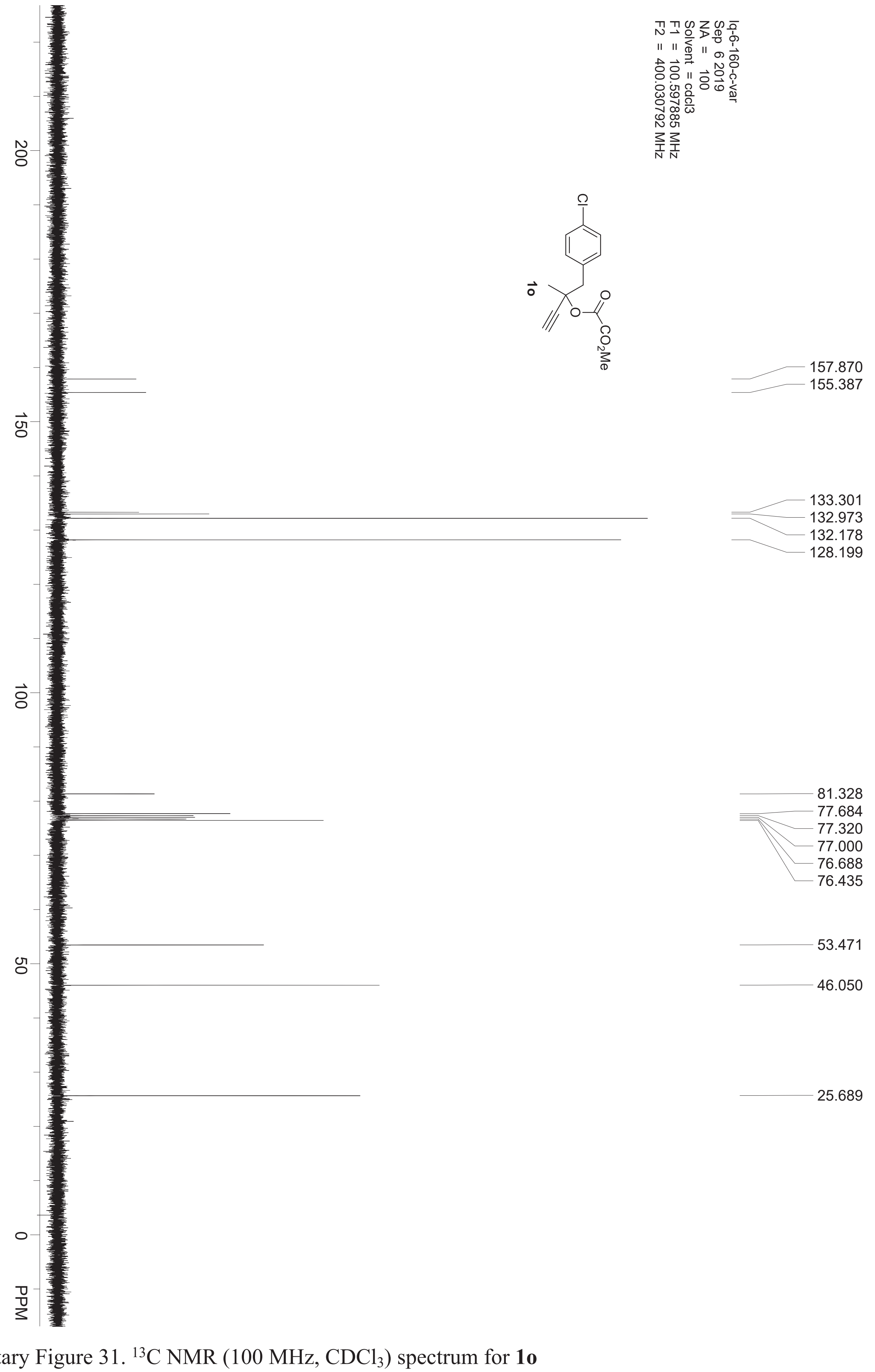

Supplementary Figure 31. ^{13}C NMR (100 MHz, $CDCl_3$) spectrum for **1o**

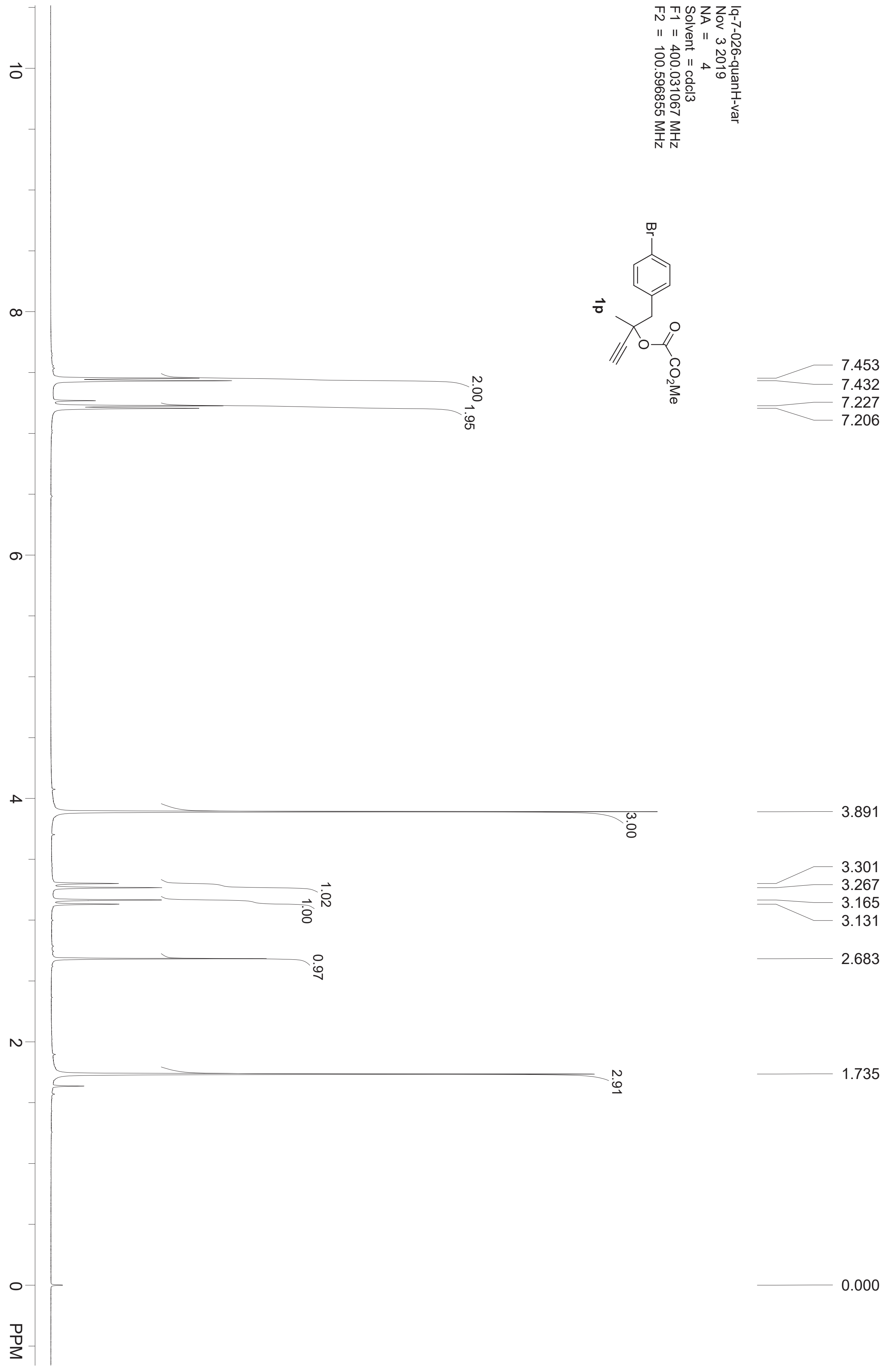

Supplementary Figure 32. ^{1}H NMR (400 MHz, $CDCl_3$) spectrum for **1p**

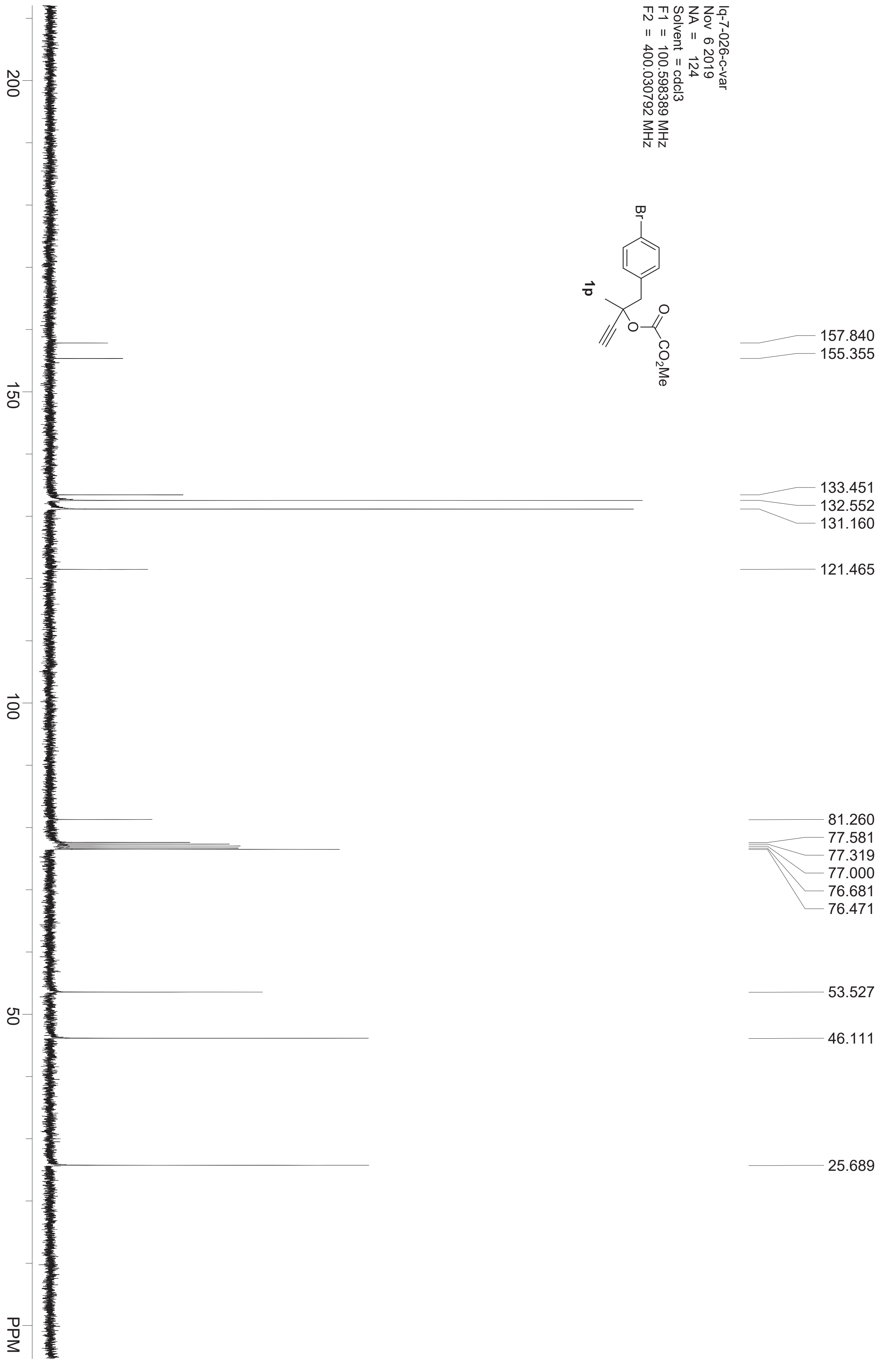

Supplementary Figure 33. ^{13}C NMR (100 MHz, $CDCl_3$) spectrum for **1p**

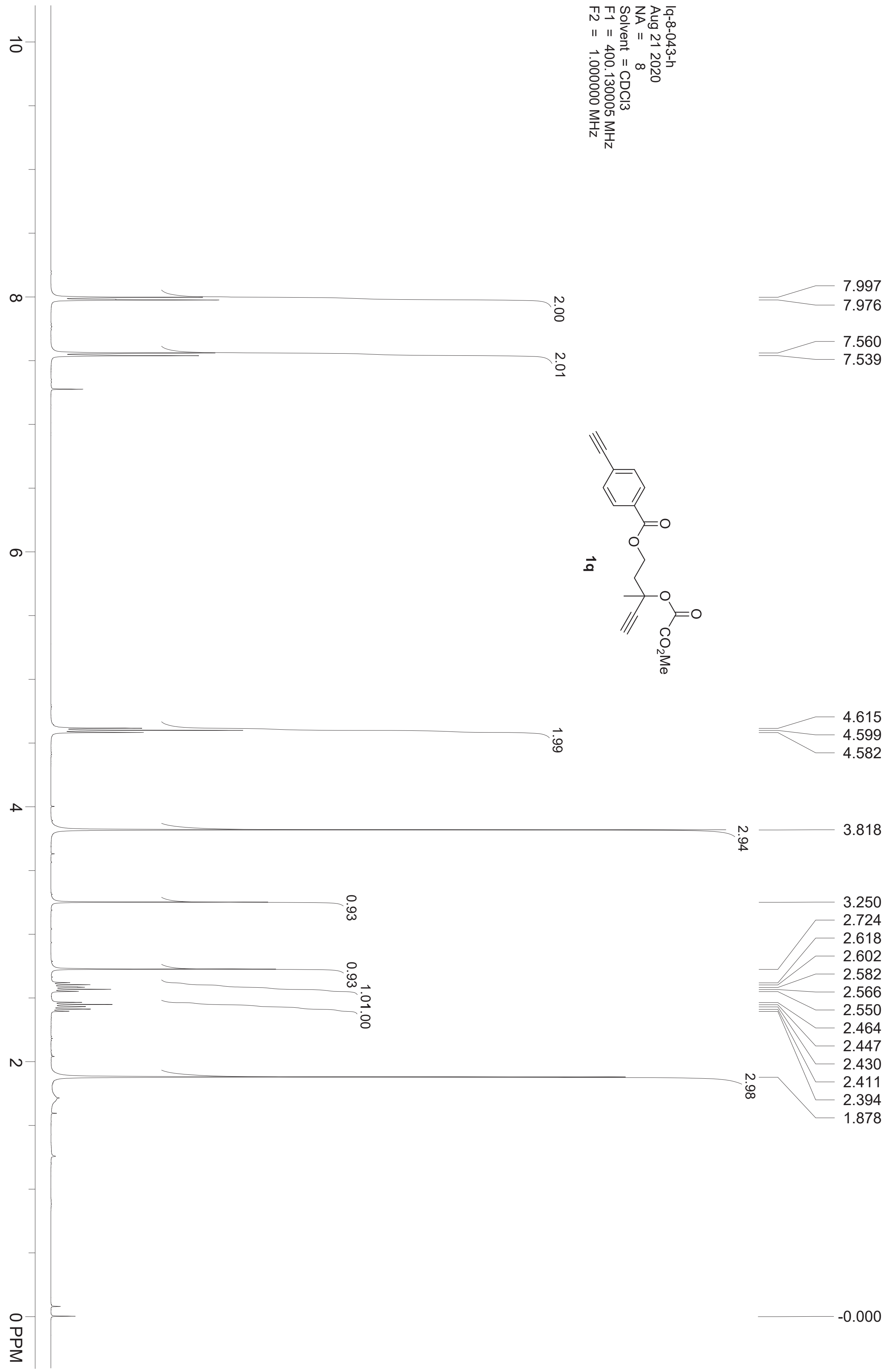

Supplementary Figure 34. ^{1}H NMR (400 MHz, $CDCl_3$) spectrum for **1q**

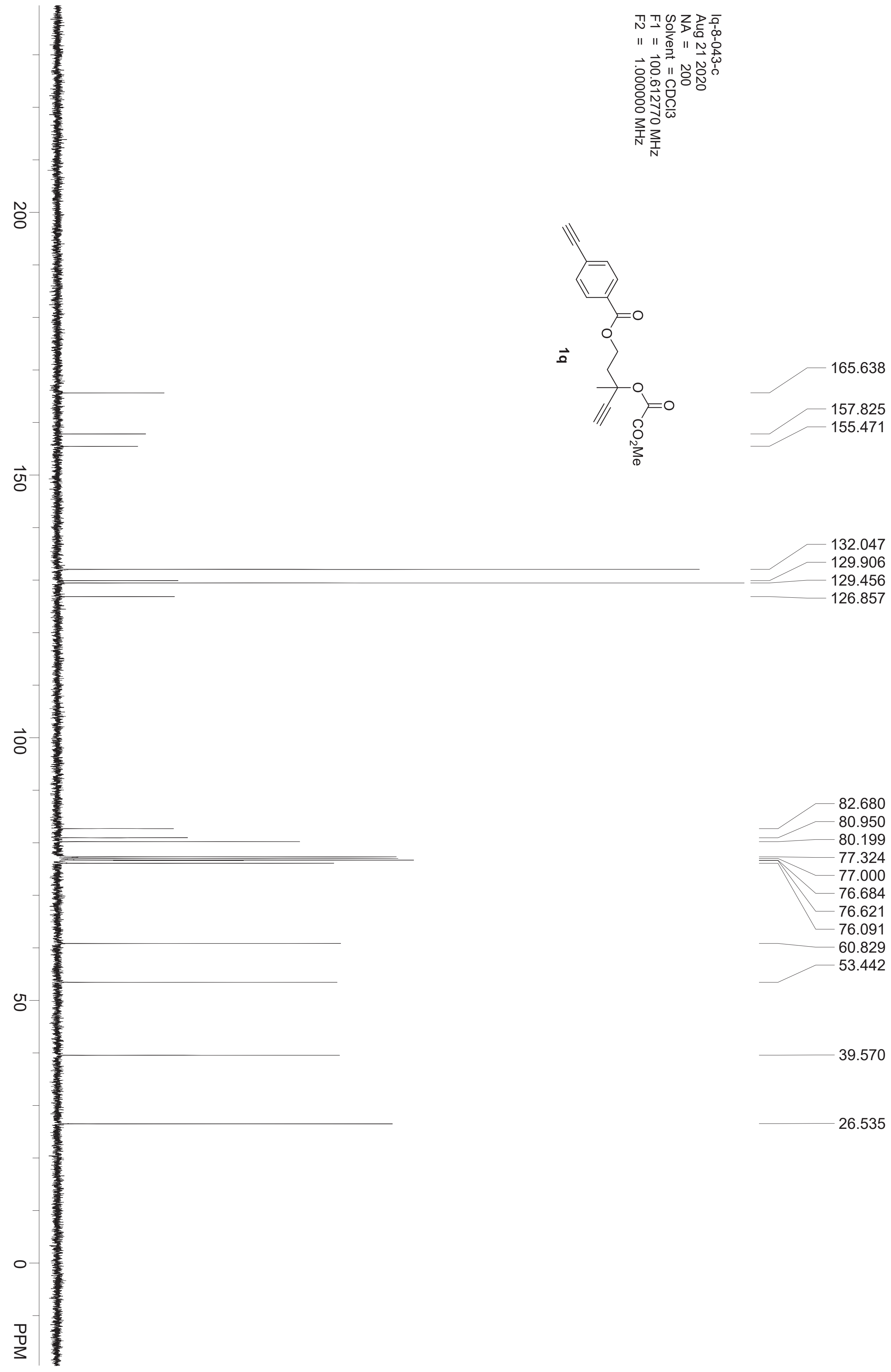

Supplementary Figure 35. ^{13}C NMR (100 MHz, $CDCl_3$) spectrum for **1q**

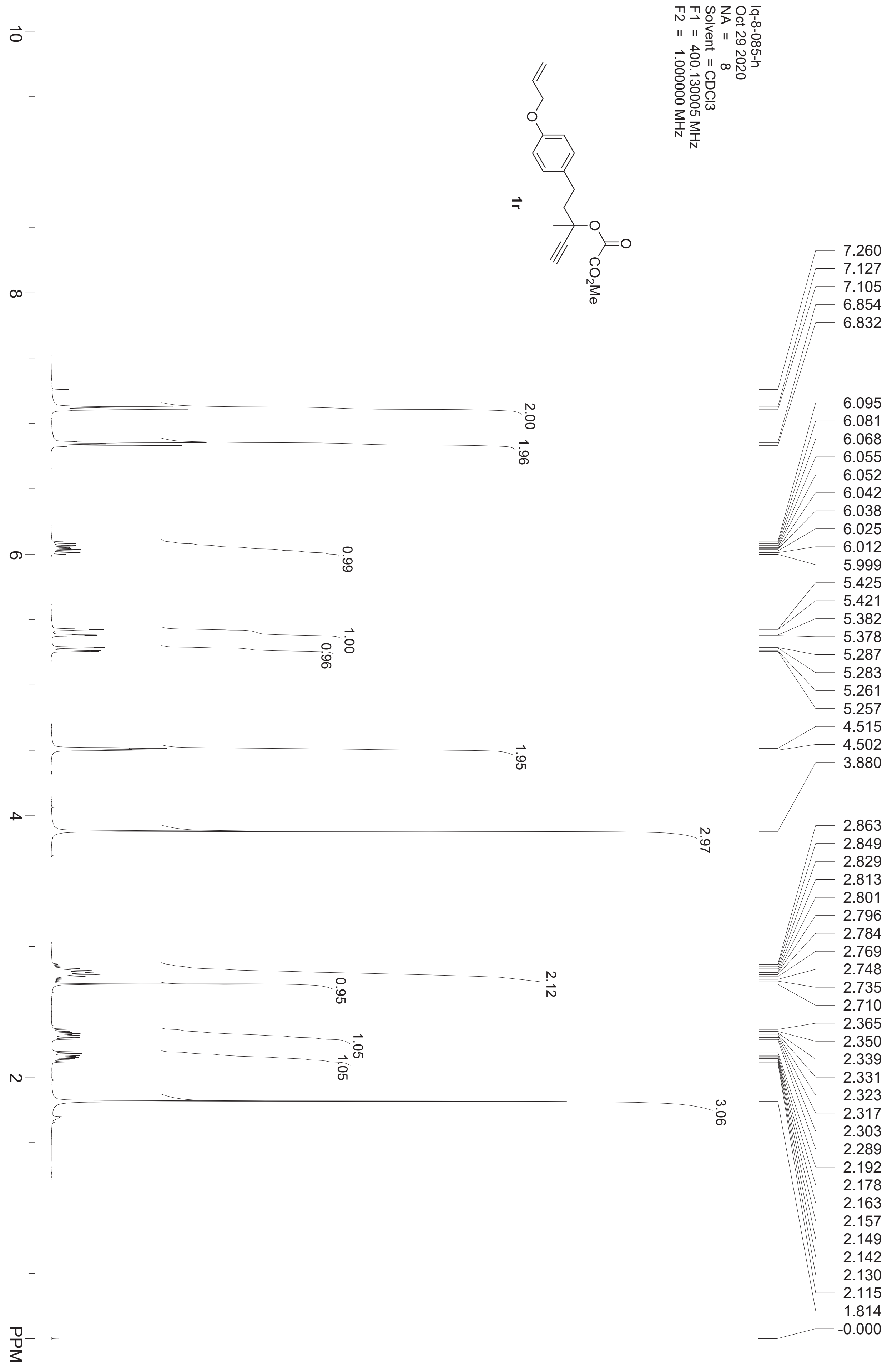

Supplementary Figure 36. ^{1}H NMR (400 MHz, $CDCl_3$) spectrum for **1r**

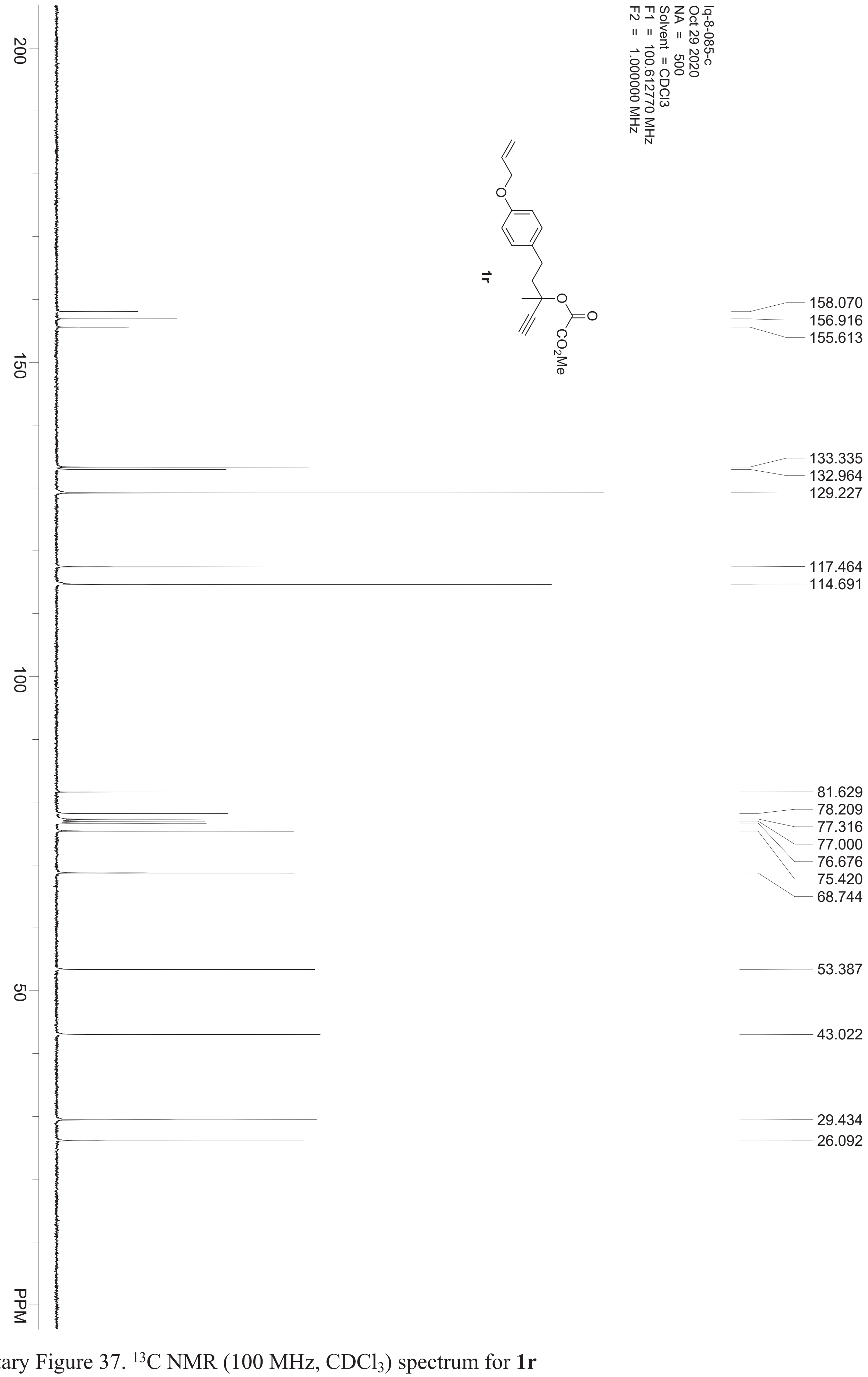

Supplementary Figure 37. ^{13}C NMR (100 MHz, $CDCl_3$) spectrum for **1r**

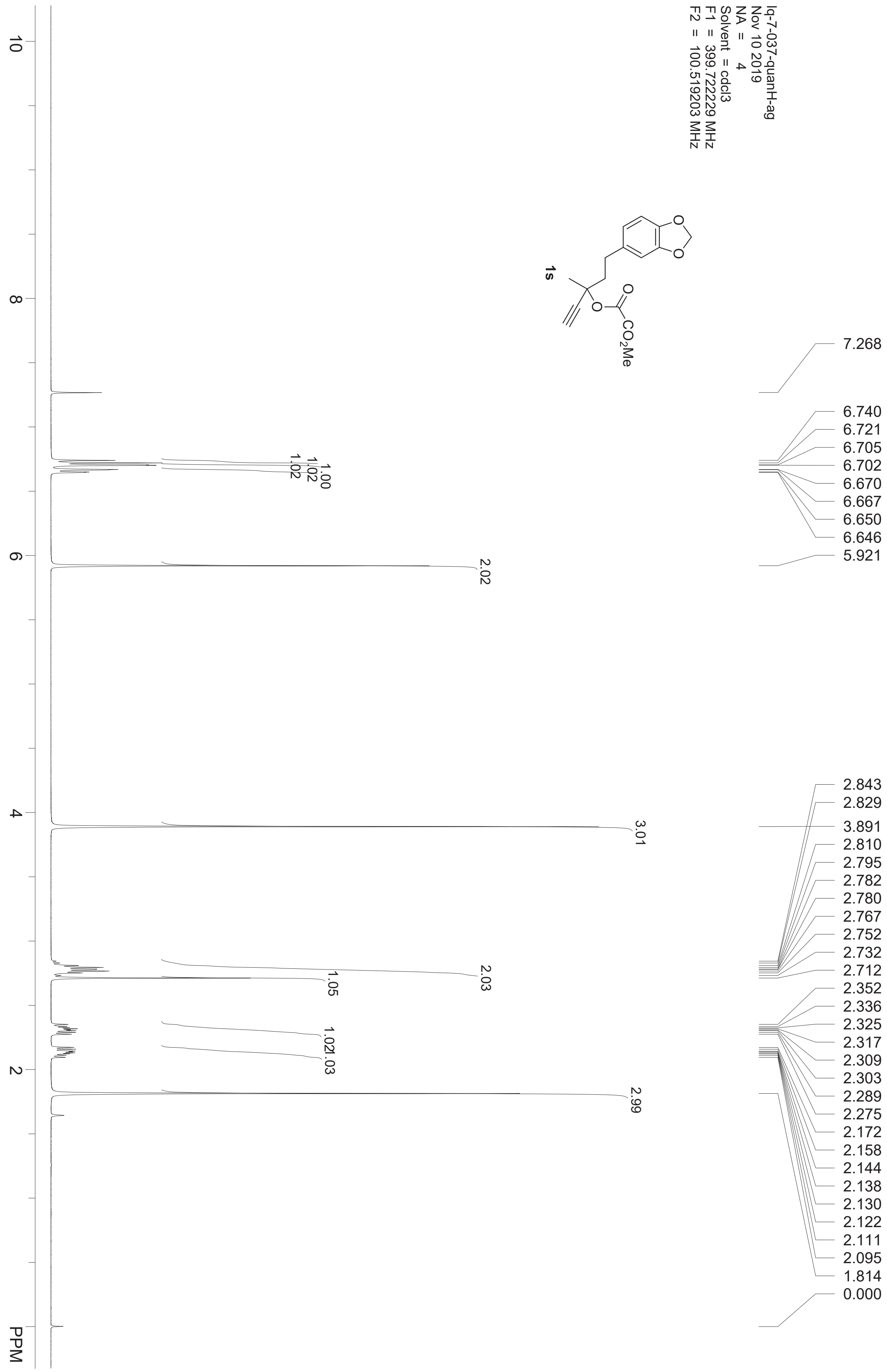

Supplementary Figure 38. ^{1}H NMR (400 MHz, $CDCl_3$) spectrum for **1s**

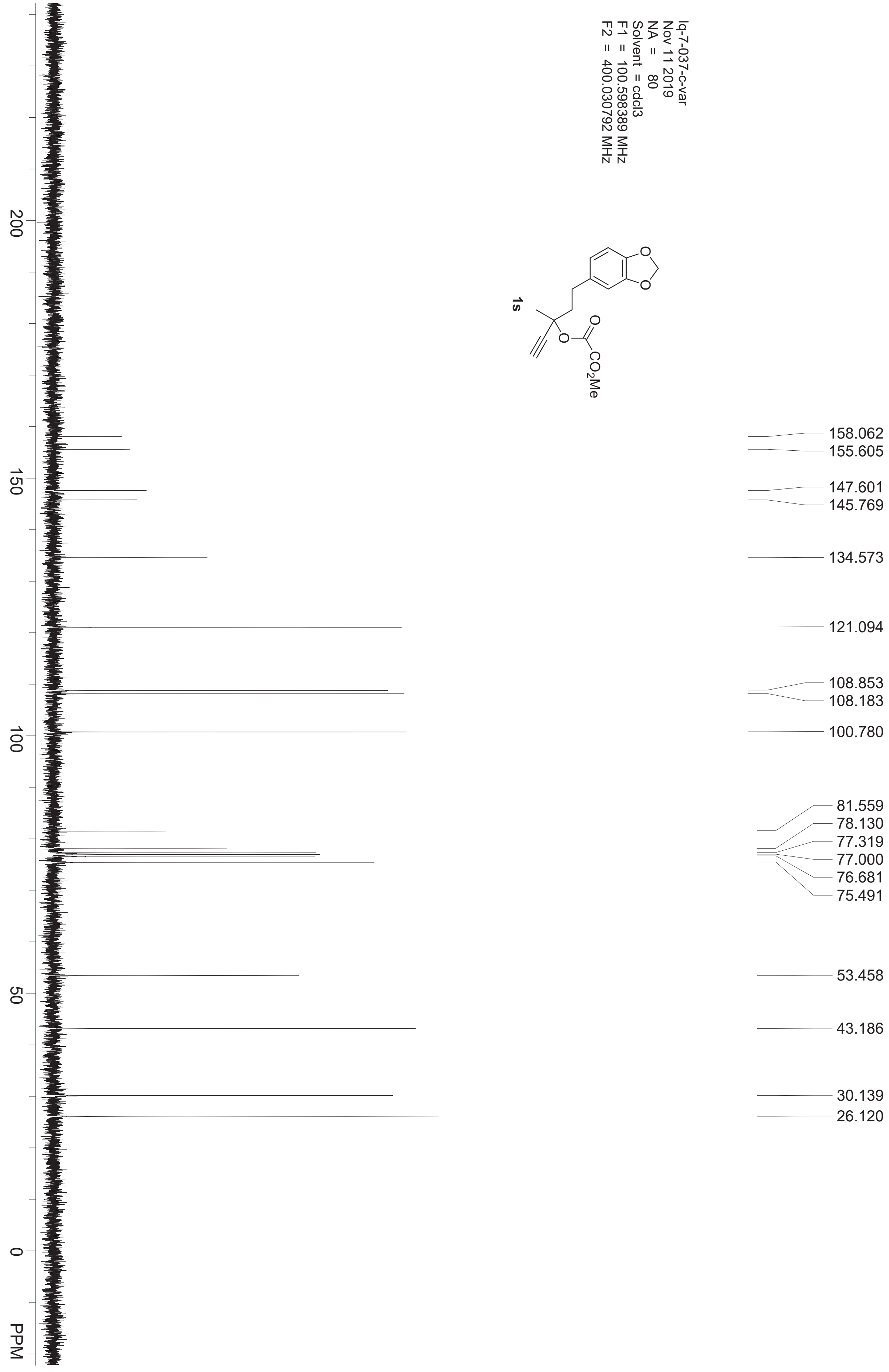

Supplementary Figure 39. ^{13}C NMR (100 MHz, $CDCl_3$) spectrum for **1s**

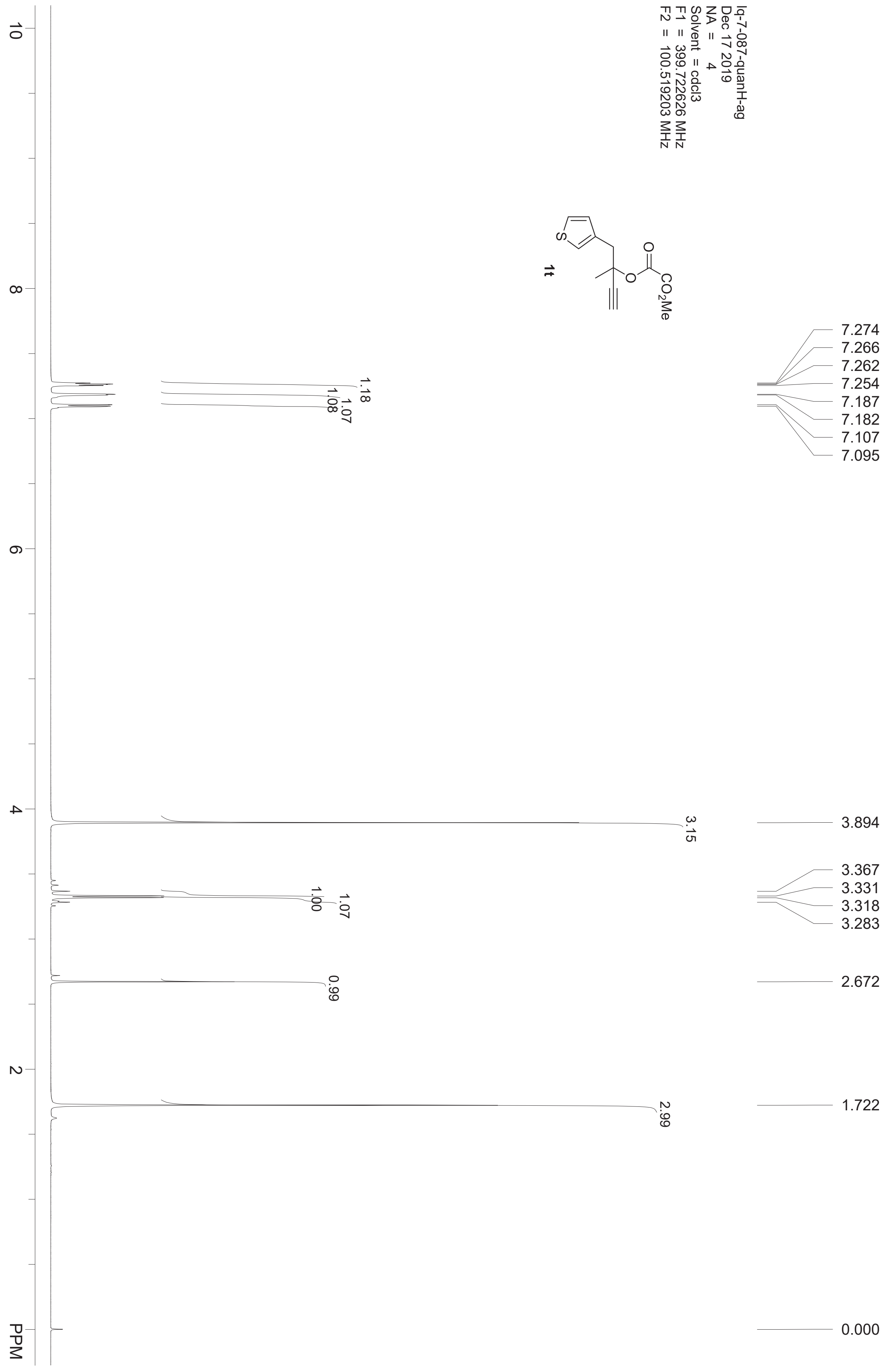

Supplementary Figure 40. ^{1}H NMR (400 MHz, $CDCl_3$) spectrum for **1t**

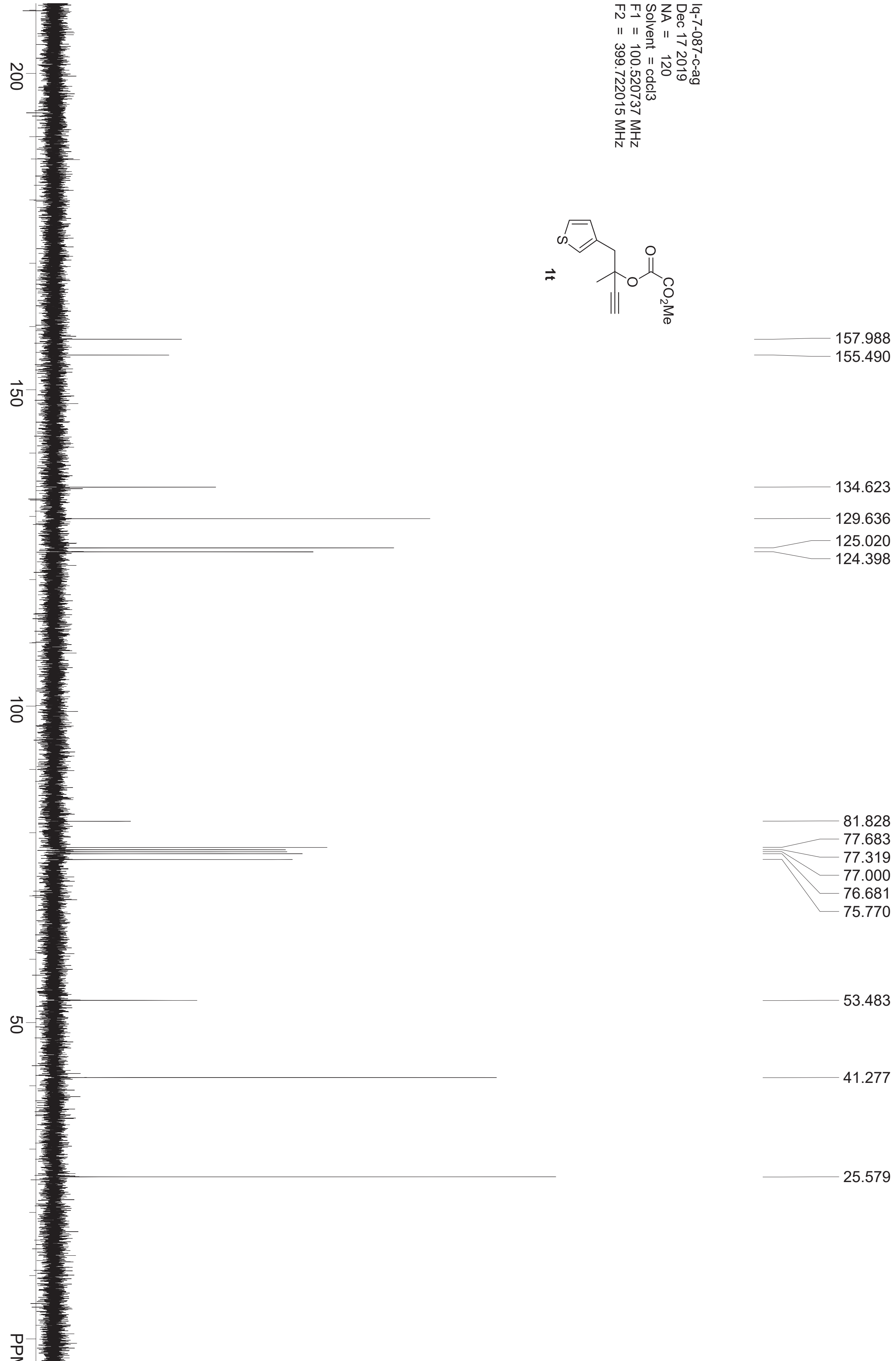

Supplementary Figure 41. ^{13}C NMR (100 MHz, $CDCl_3$) spectrum for **1t**

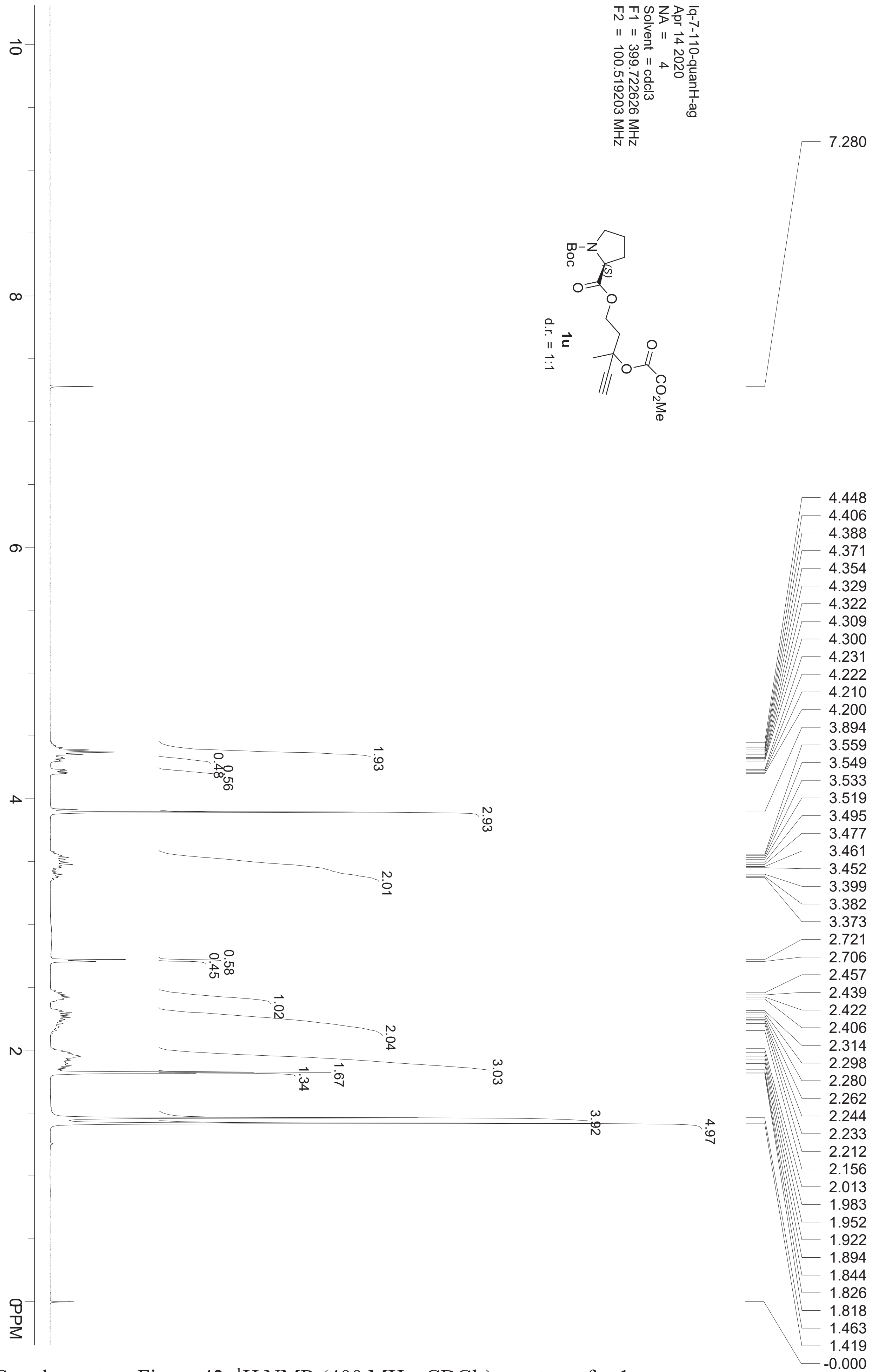

Supplementary Figure 42. ^{1}H NMR (400 MHz, $CDCl_3$) spectrum for **1u**

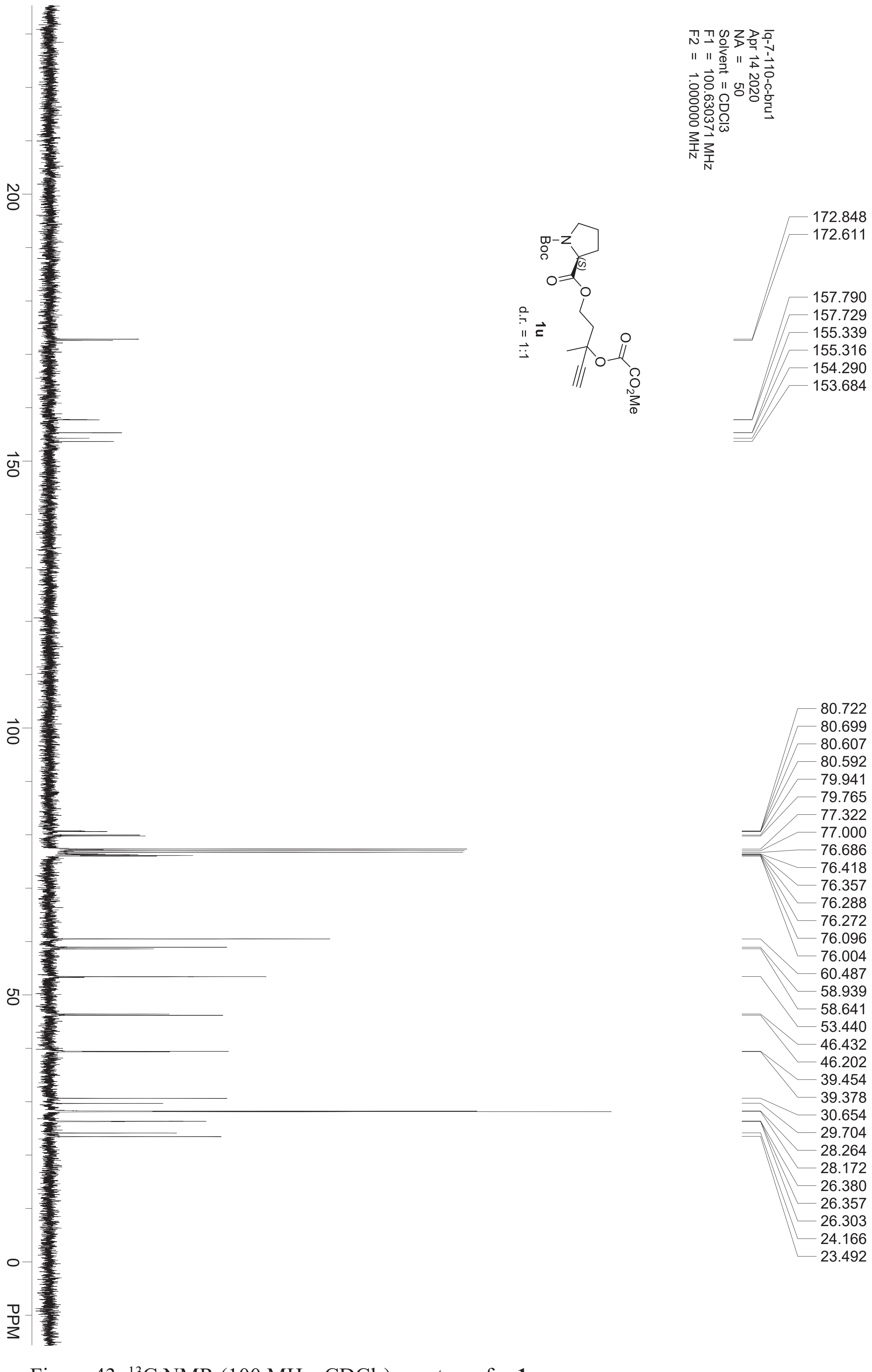

Supplementary Figure 43. ^{13}C NMR (100 MHz, $CDCl_3$) spectrum for **1u**

Area Percent Report

Agilent Technologies

sample LQ-7-110-OD-H-90-10-0.5-214

Data file: C:\Users\Public\Documents\ChemStation\1\Data\YuanYuan 2021-01-28 14-08-30\002-P2-F4-lq-7-110.D

Acquisition Data:

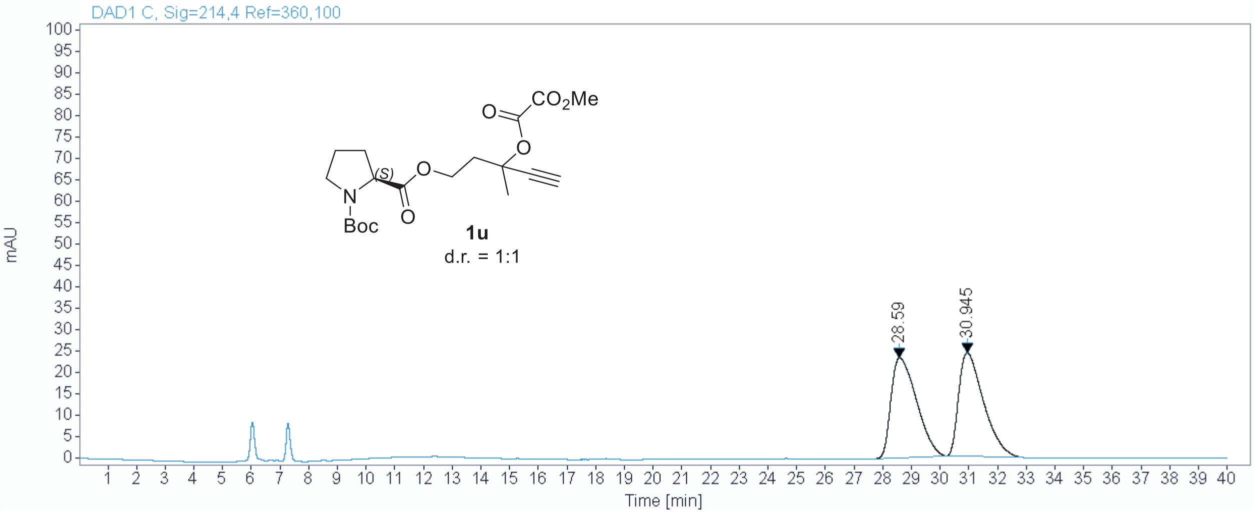

Signal: DAD1 C, Sig=214,4 Ref=360,100

RT [min]	Width [min]	Height	Area	Area%
28.590	1.0256	23.3921	1439.4119	49.8243
30.945	0.9998	24.1639	1449.5665	50.1757
		Sum	2888.9784	100.0000

Supplementary Figure 44. HPLC spectrum for **1u**

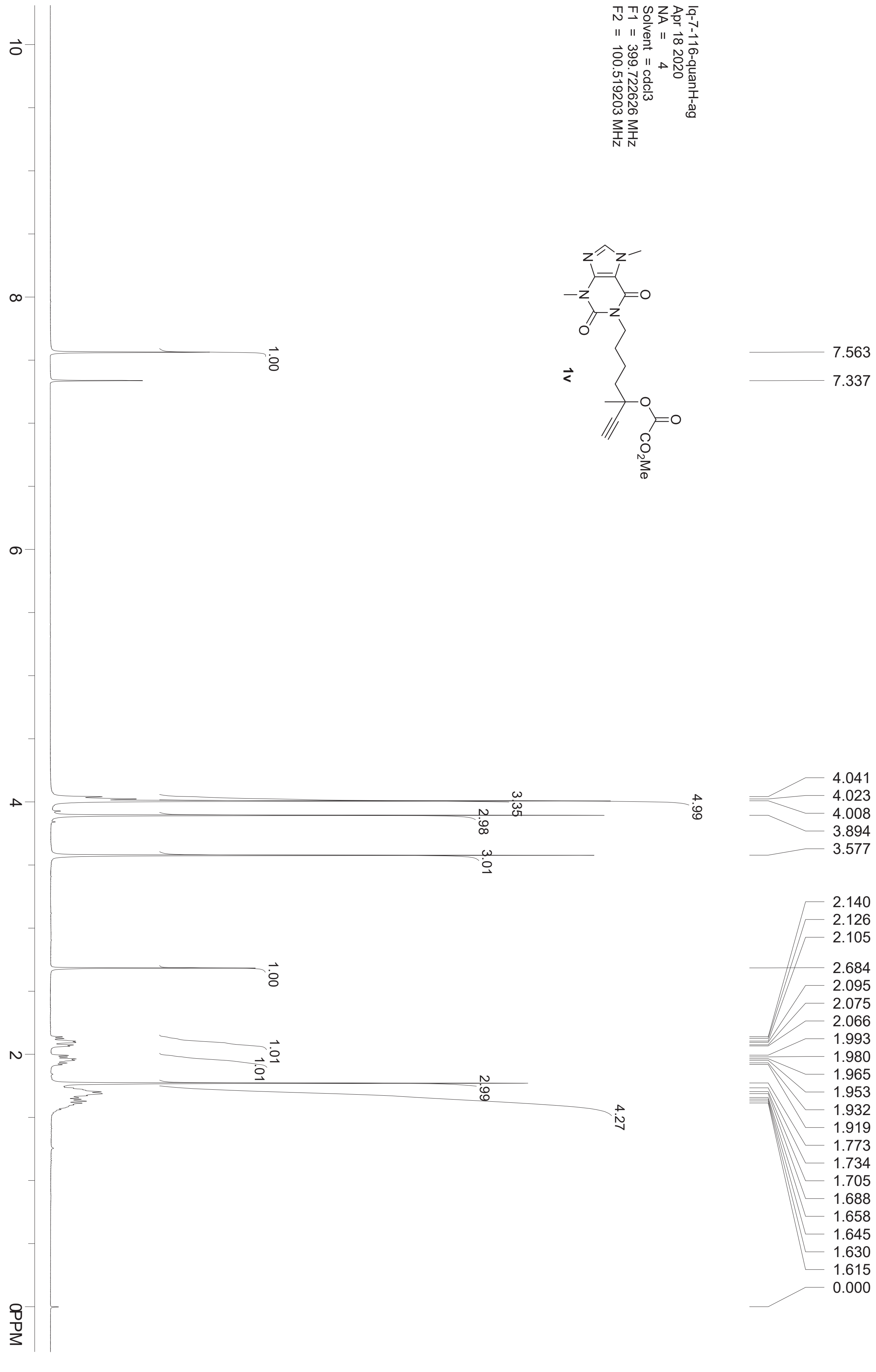

Supplementary Figure 45. ^{1}H NMR (400 MHz, $CDCl_3$) spectrum for **1v**

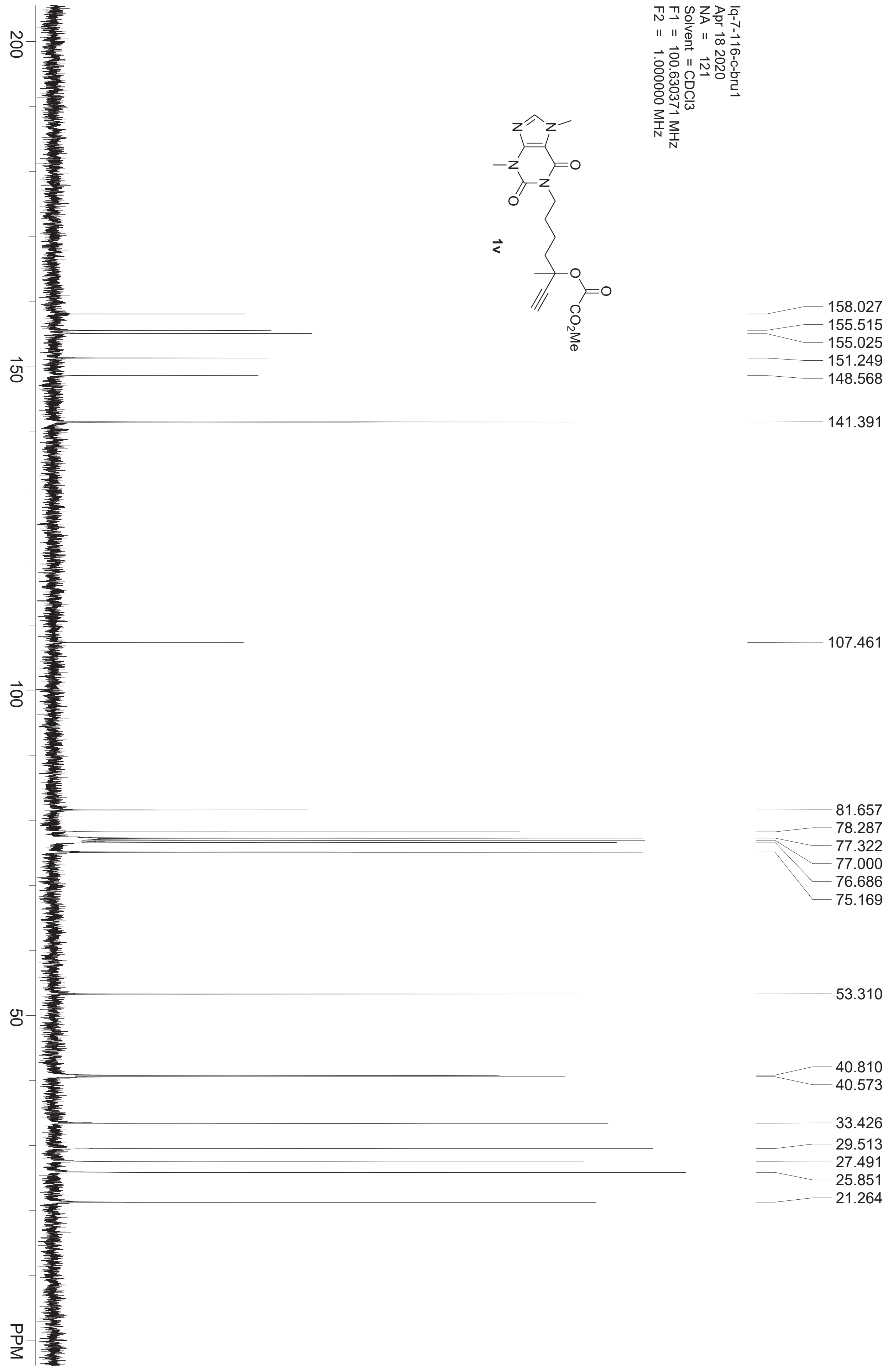

Supplementary Figure 46. ^{13}C NMR (100 MHz, $CDCl_3$) spectrum for **1v**

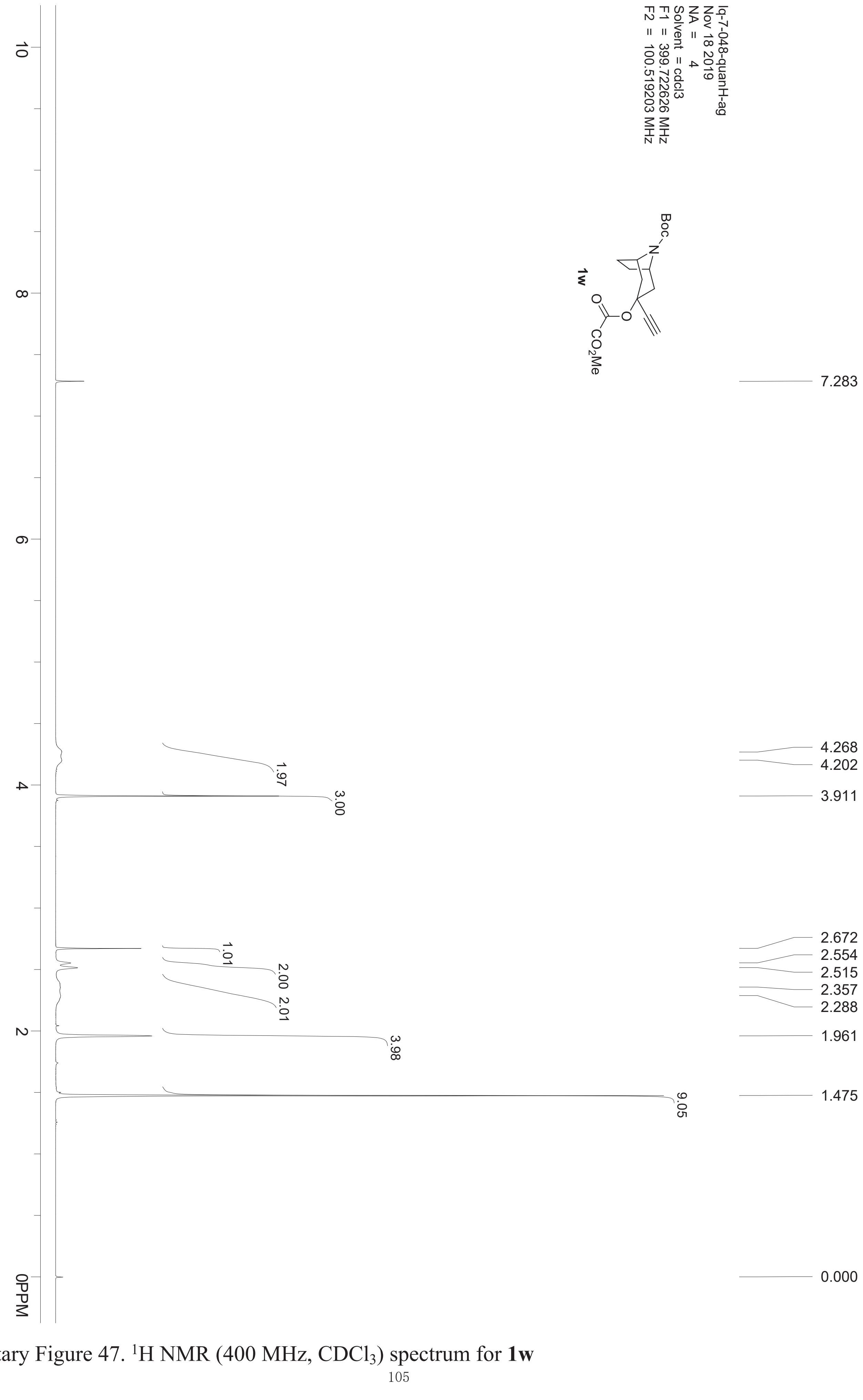

Supplementary Figure 47. ^{1}H NMR (400 MHz, $CDCl_3$) spectrum for **1w**

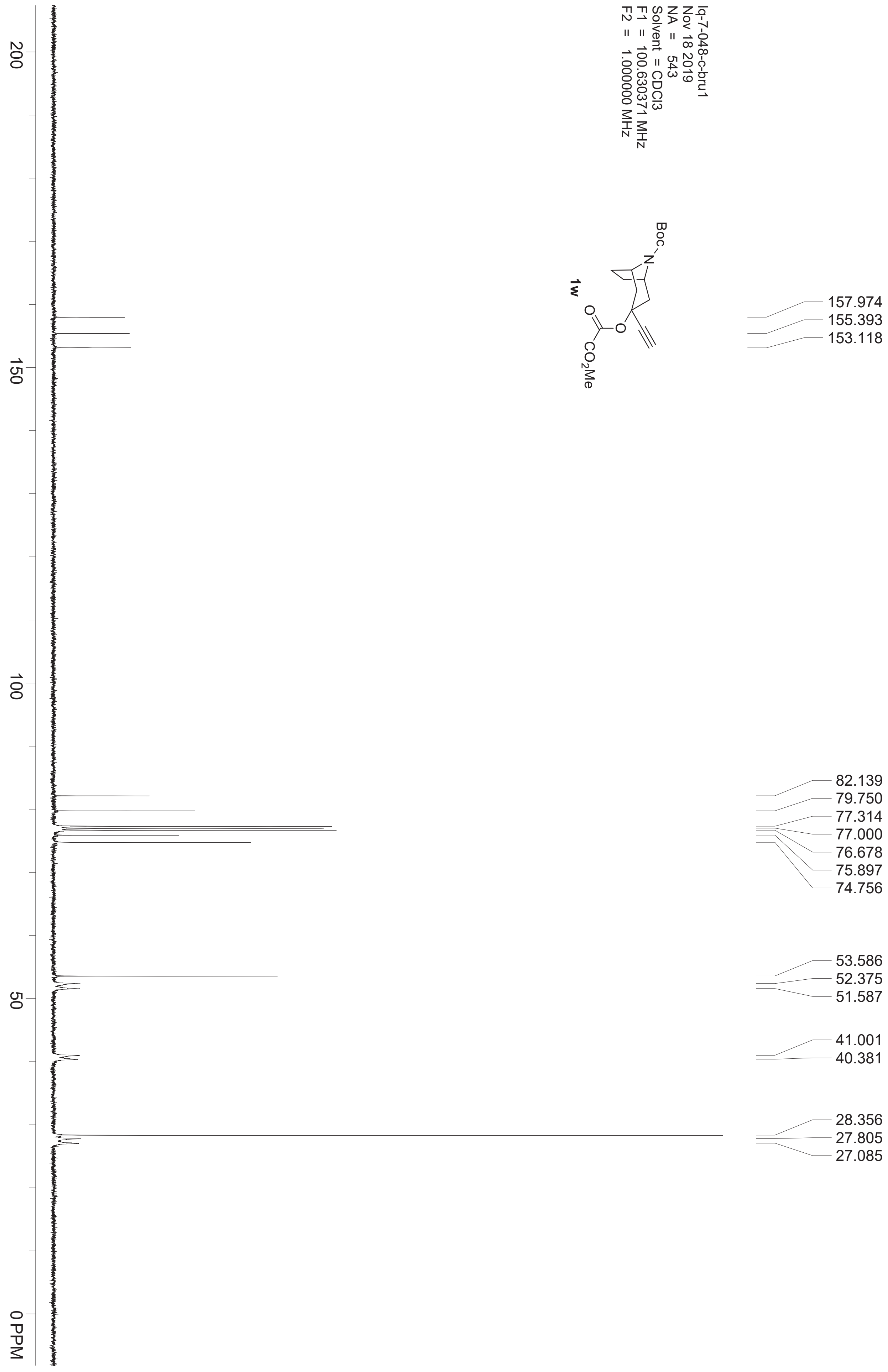

Supplementary Figure 48. ^{13}C NMR (100 MHz, $CDCl_3$) spectrum for **1w**

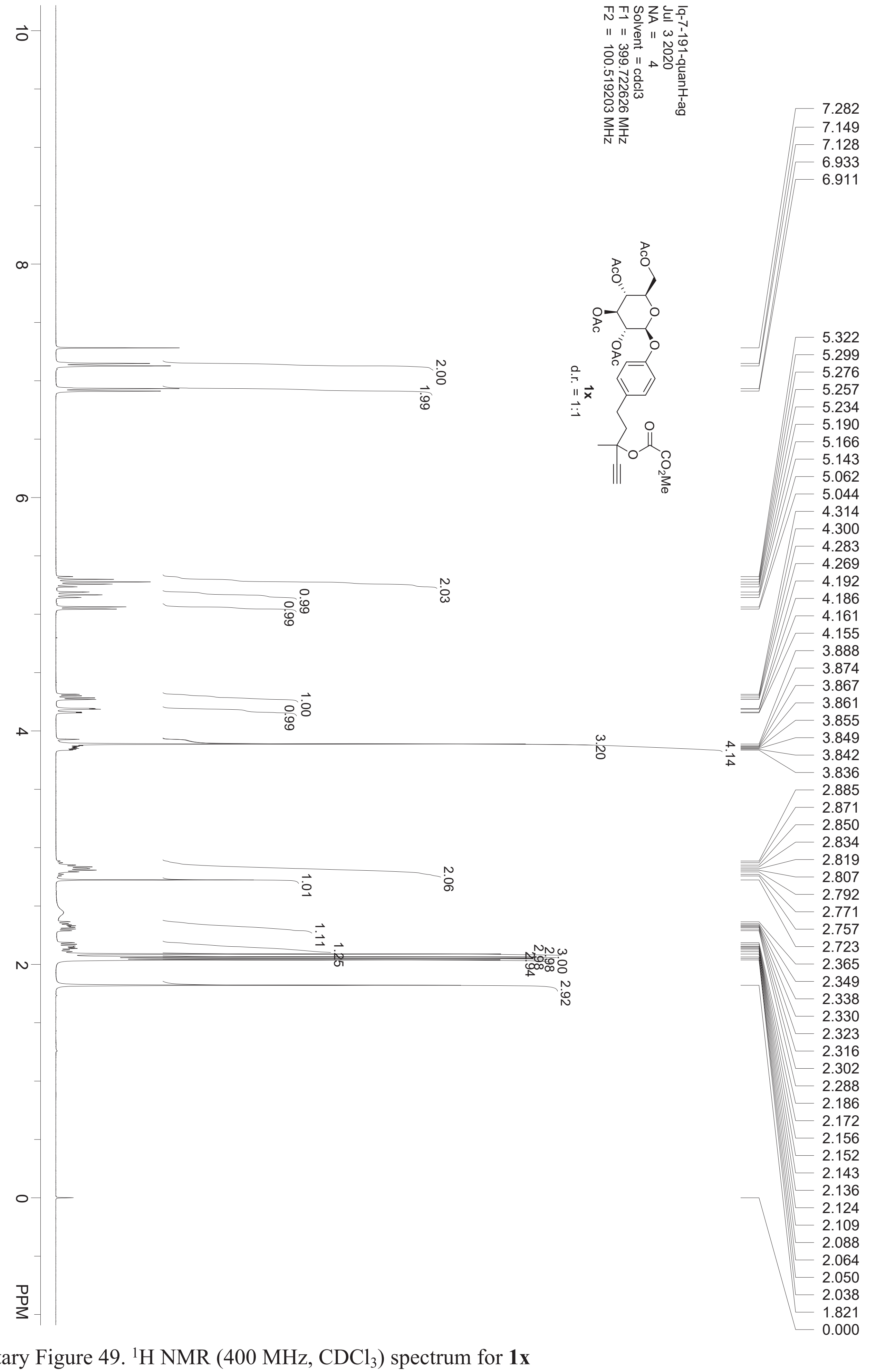

Supplementary Figure 49. ^{1}H NMR (400 MHz, $CDCl_3$) spectrum for **1x**

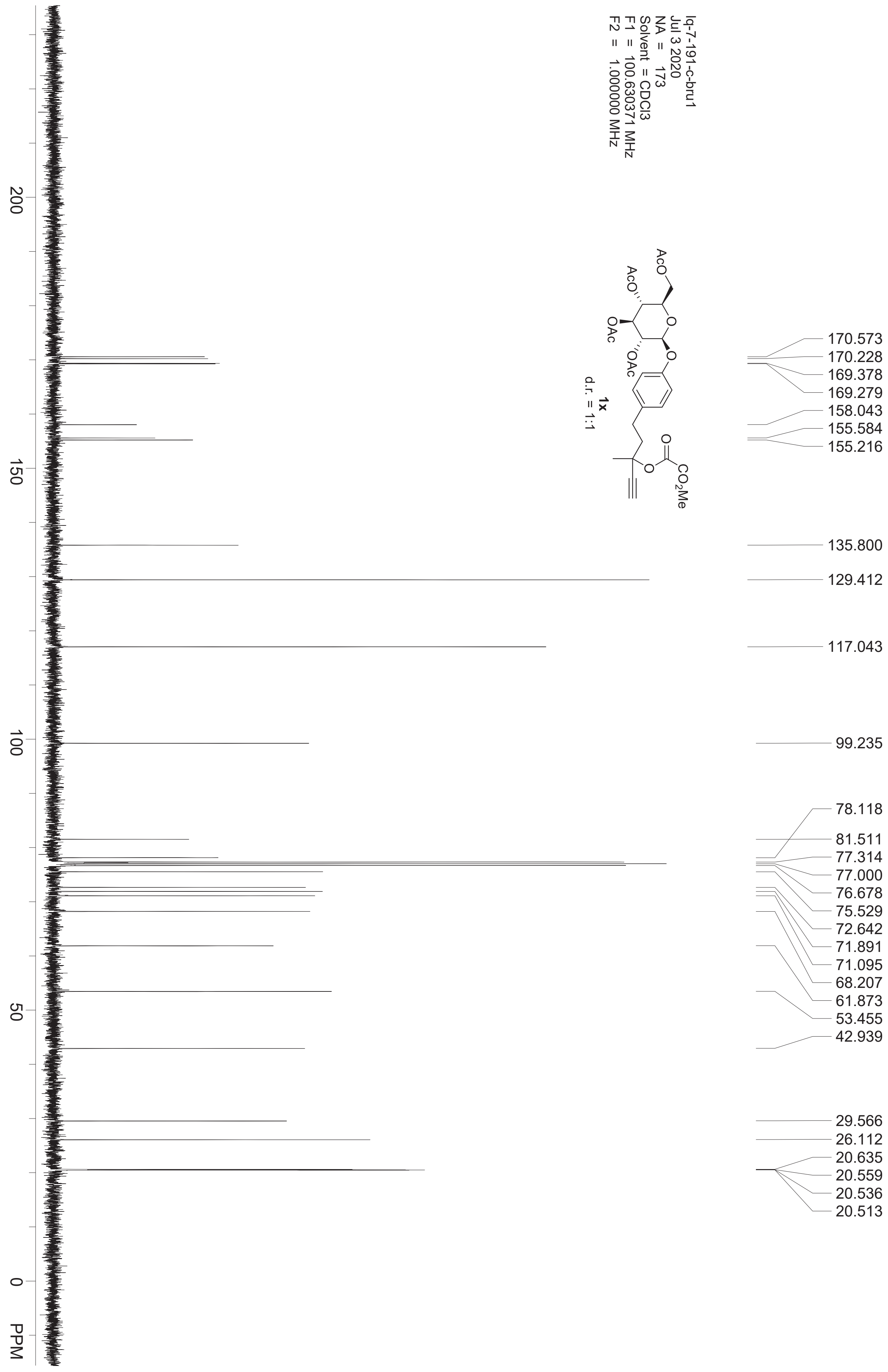

Supplementary Figure 50. ^{13}C NMR (100 MHz, $CDCl_3$) spectrum for **1x**

Empower 3 SOFTWARE

Default Individual Report

SAMPLE INFORMATION

Sample Name:	lq-7-191-ic-60-40-1-214	Acquired By:	System
Sample Type:	Unknown	Sample Set Name:	
Vial:	1	Acq. Method Set:	zgj60
Injection #:	89	Processing Method:	111
Injection Volume:	10.00 ul	Channel Name:	W2489 ChA
Run Time:	40.0 Minutes	Proc. Chnl. Descr.:	W2489 ChA 214nm
Date Acquired:	7/9/2020 6:05:39 PM EDT		
Date Processed:	7/9/2020 6:43:11 PM EDT		

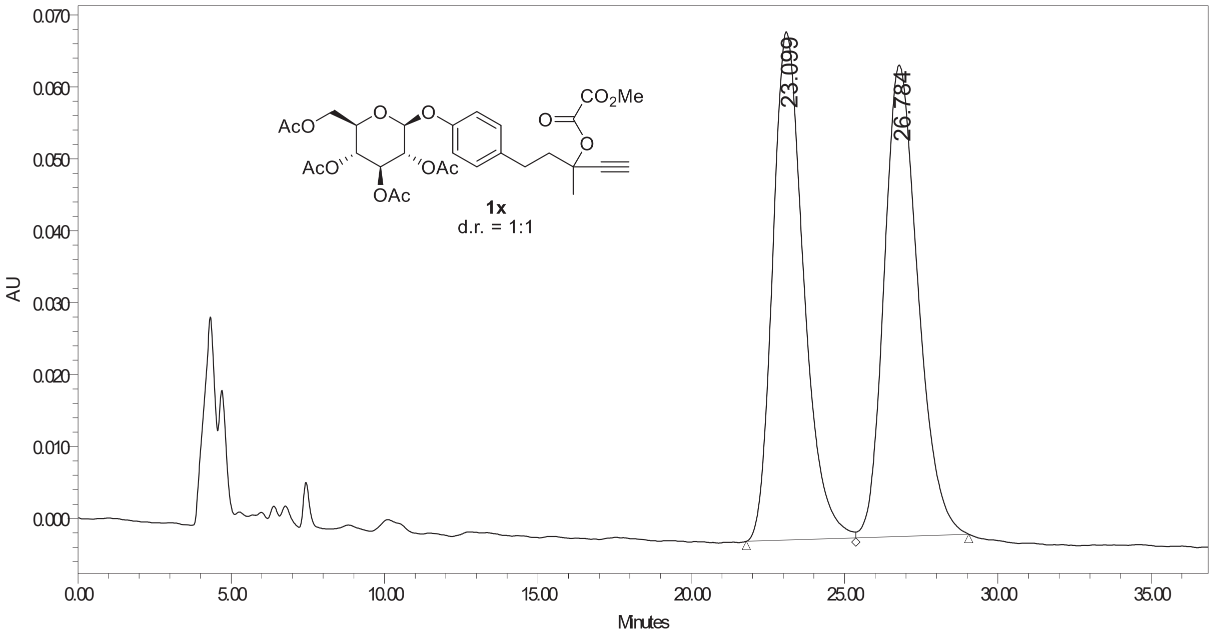

	RT	Area	%Area	Height
1	23.099	4963829	49.68	70661
2	26.784	5027349	50.32	65522

Reported by User: System
Report Method: Default Individual Report
Report Method ID: 1005 1005
Page: 1 of 1

Project Name: HPLC_1525
Date Printed:
7/9/2020
10:13:22 PM America/New_York

Supplementary Figure 51. HPLC spectrum for **1x**

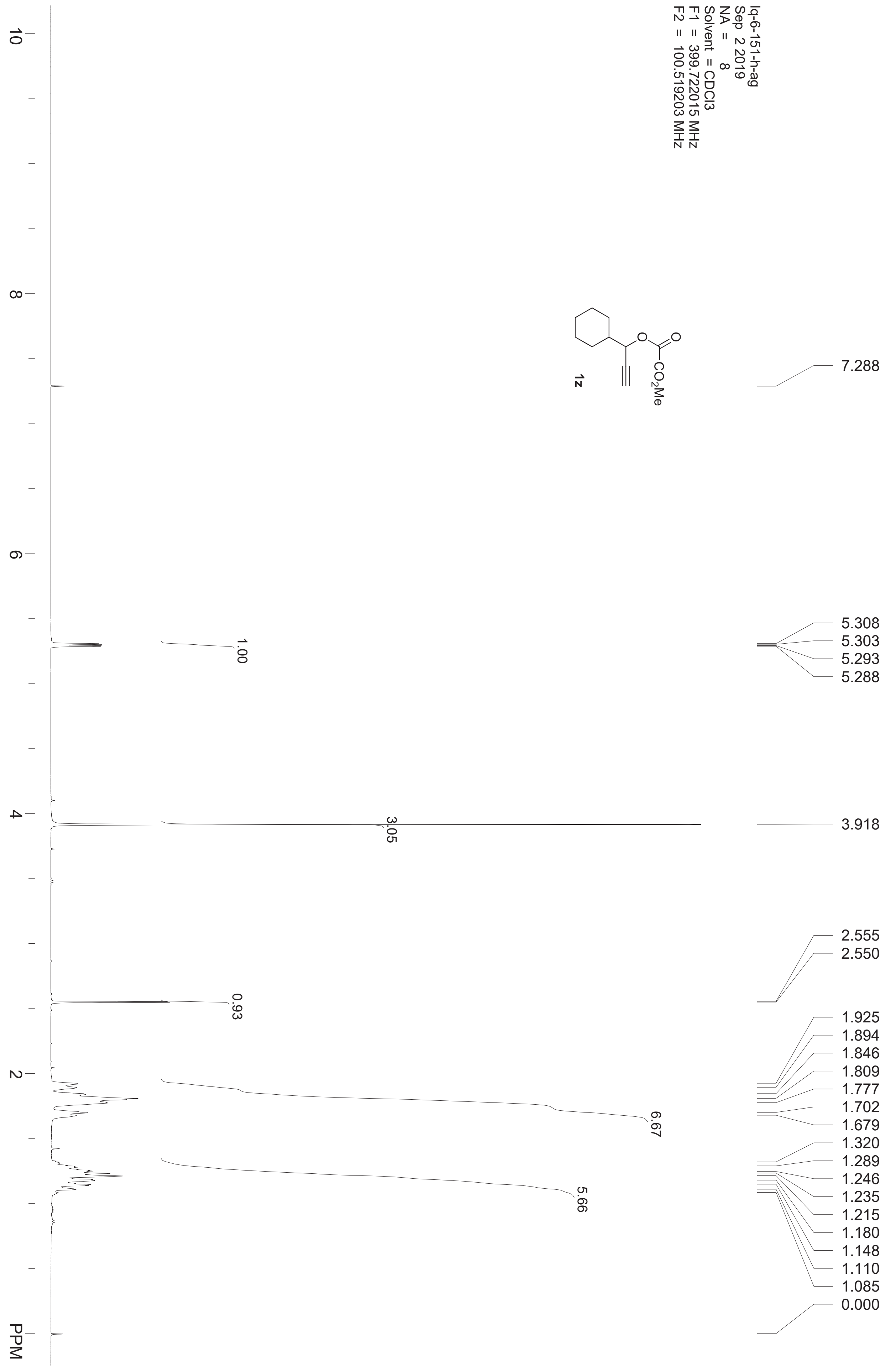

Supplementary Figure 52. ^{1}H NMR (400 MHz, $CDCl_3$) spectrum for **1z**

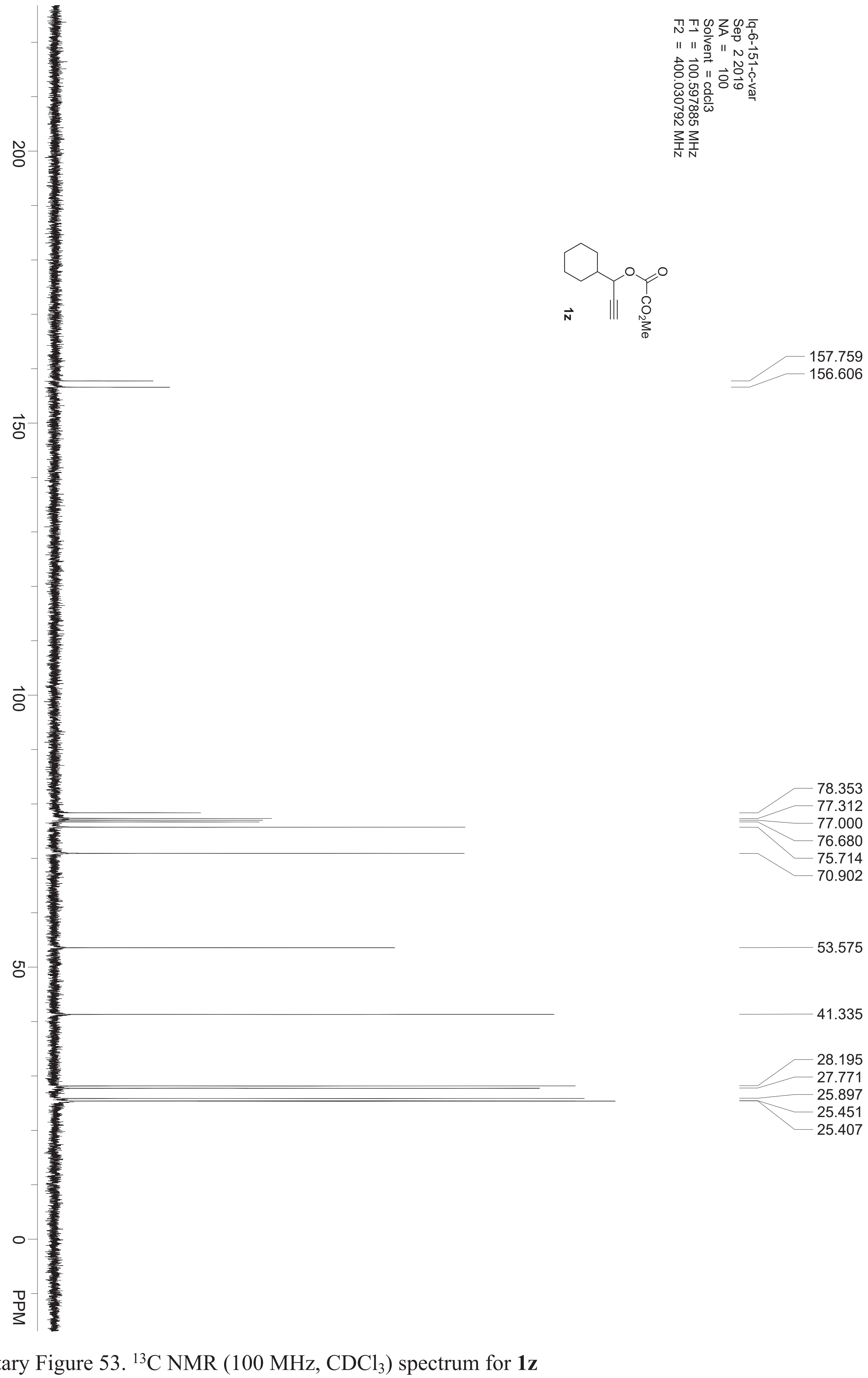

Supplementary Figure 53. ^{13}C NMR (100 MHz, $CDCl_3$) spectrum for **1z**

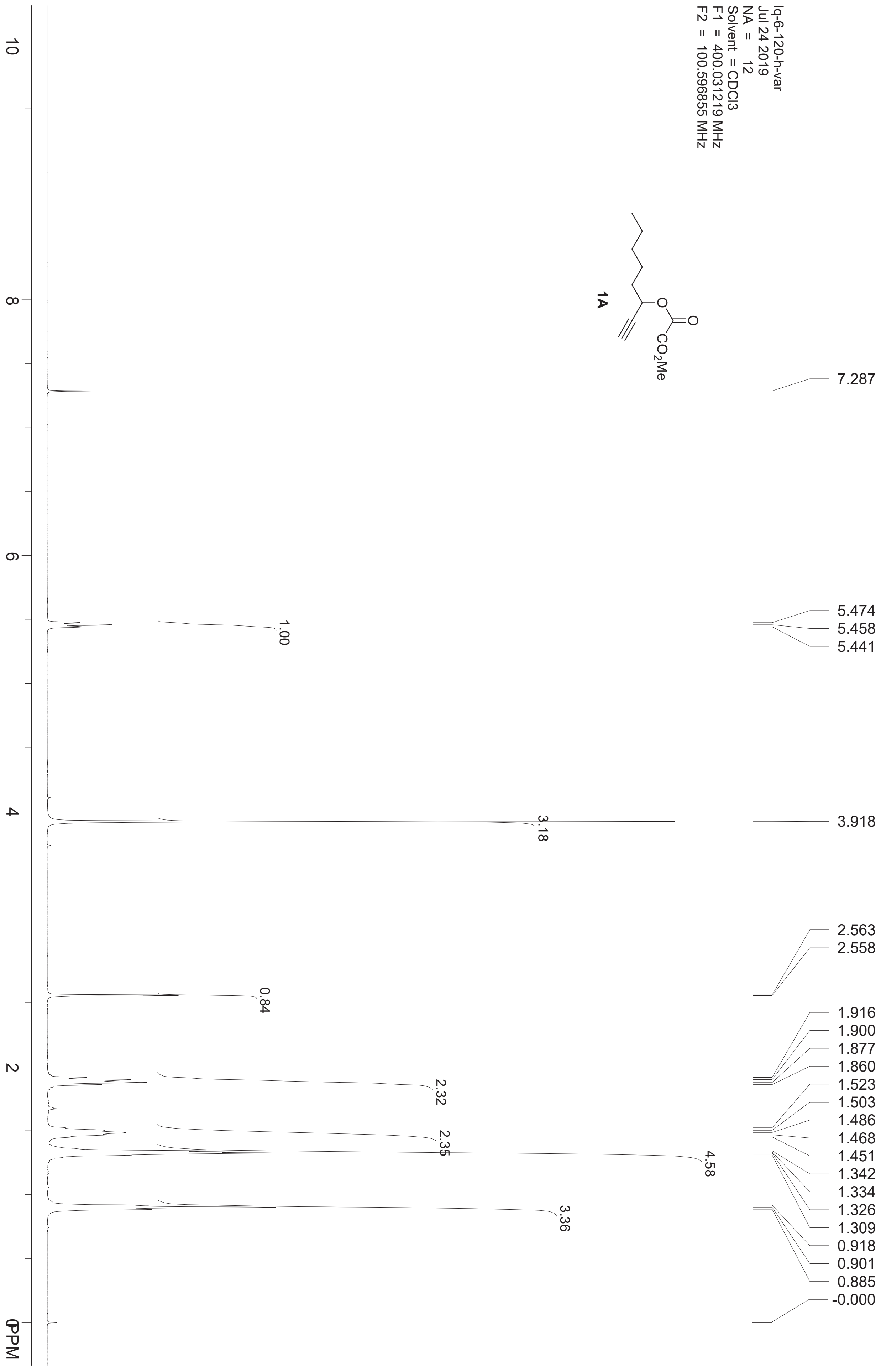

Supplementary Figure 54. ^{1}H NMR (400 MHz, $CDCl_3$) spectrum for **1A**

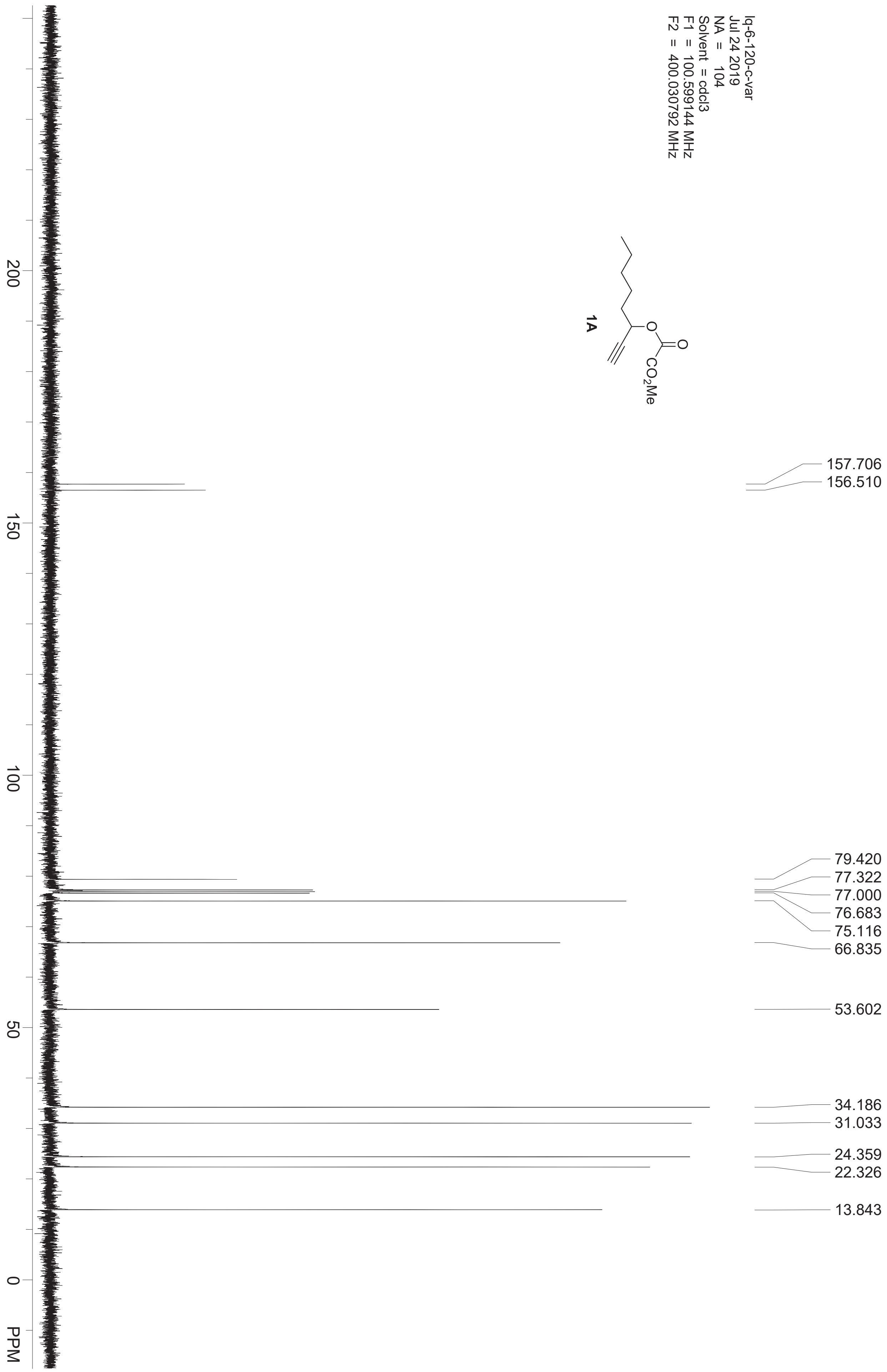

Supplementary Figure 55. ^{13}C NMR (100 MHz, CDCl$_3$) spectrum for **1A**

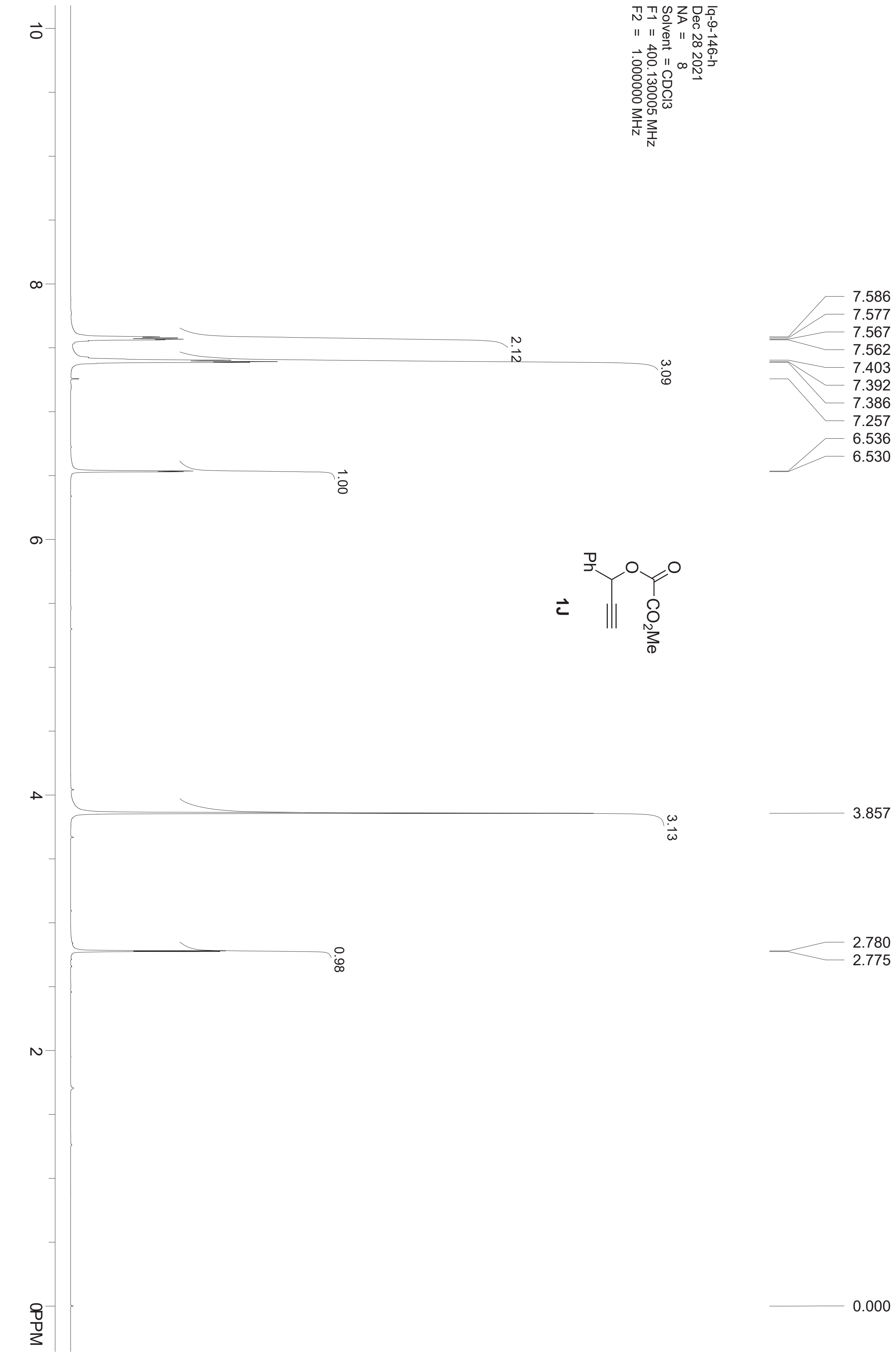

Supplementary Figure 56. ^{1}H NMR (400 MHz, $CDCl_3$) spectrum for **1J**

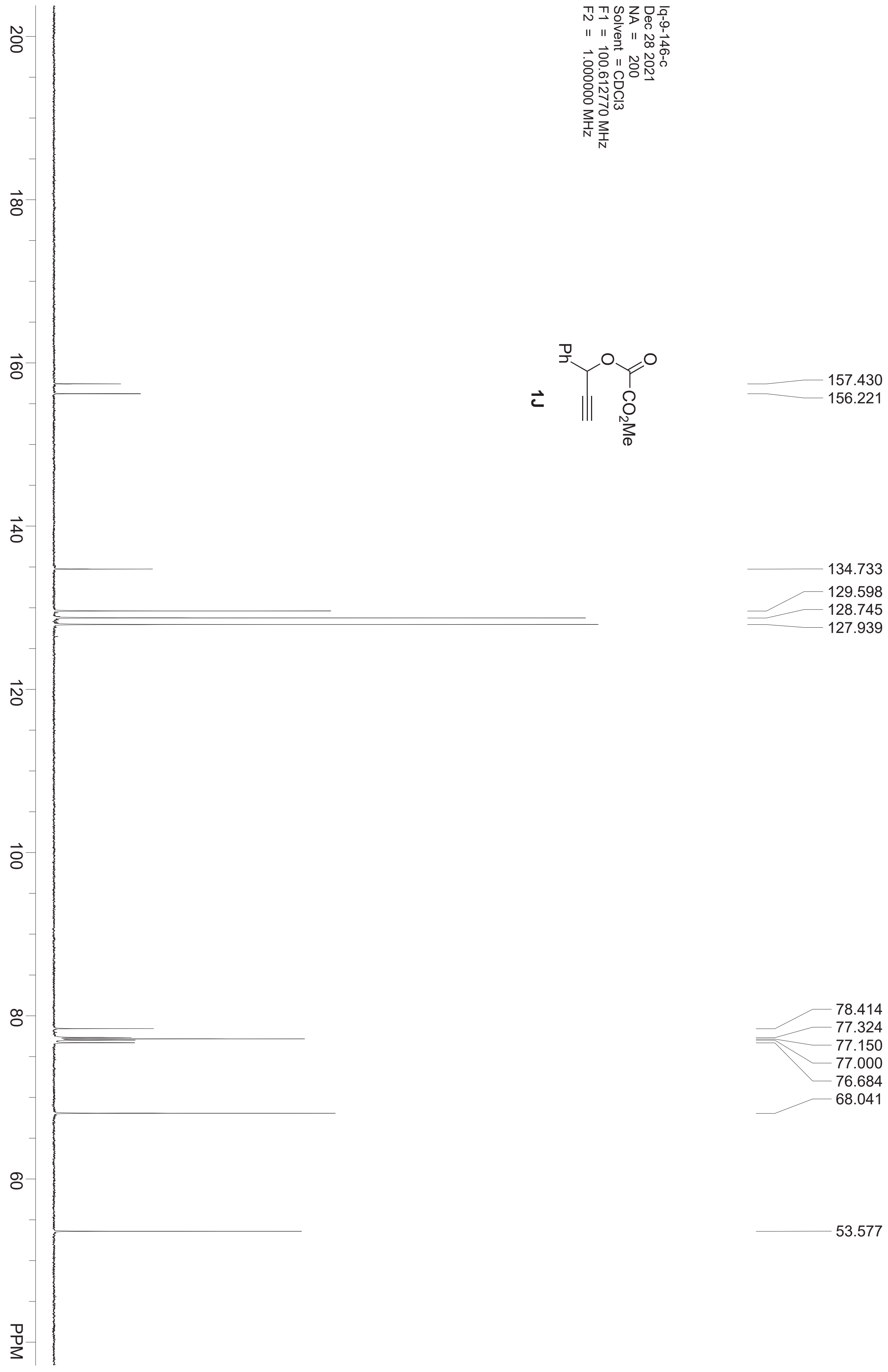

Supplementary Figure 57. ^{13}C NMR (100 MHz, $CDCl_3$) spectrum for **1J**

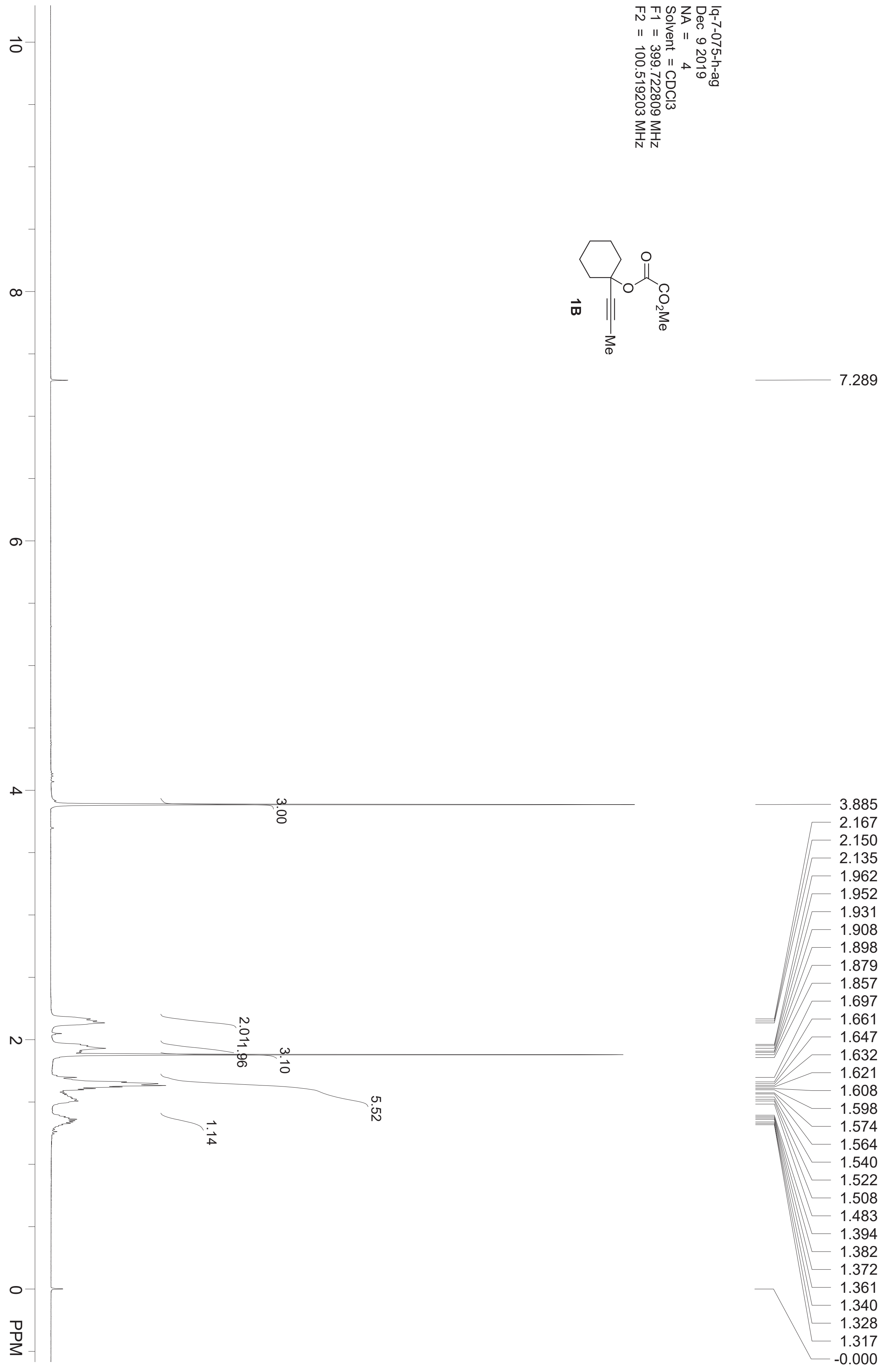

Supplementary Figure 58. ^{1}H NMR (400 MHz, $CDCl_3$) spectrum for **1B**

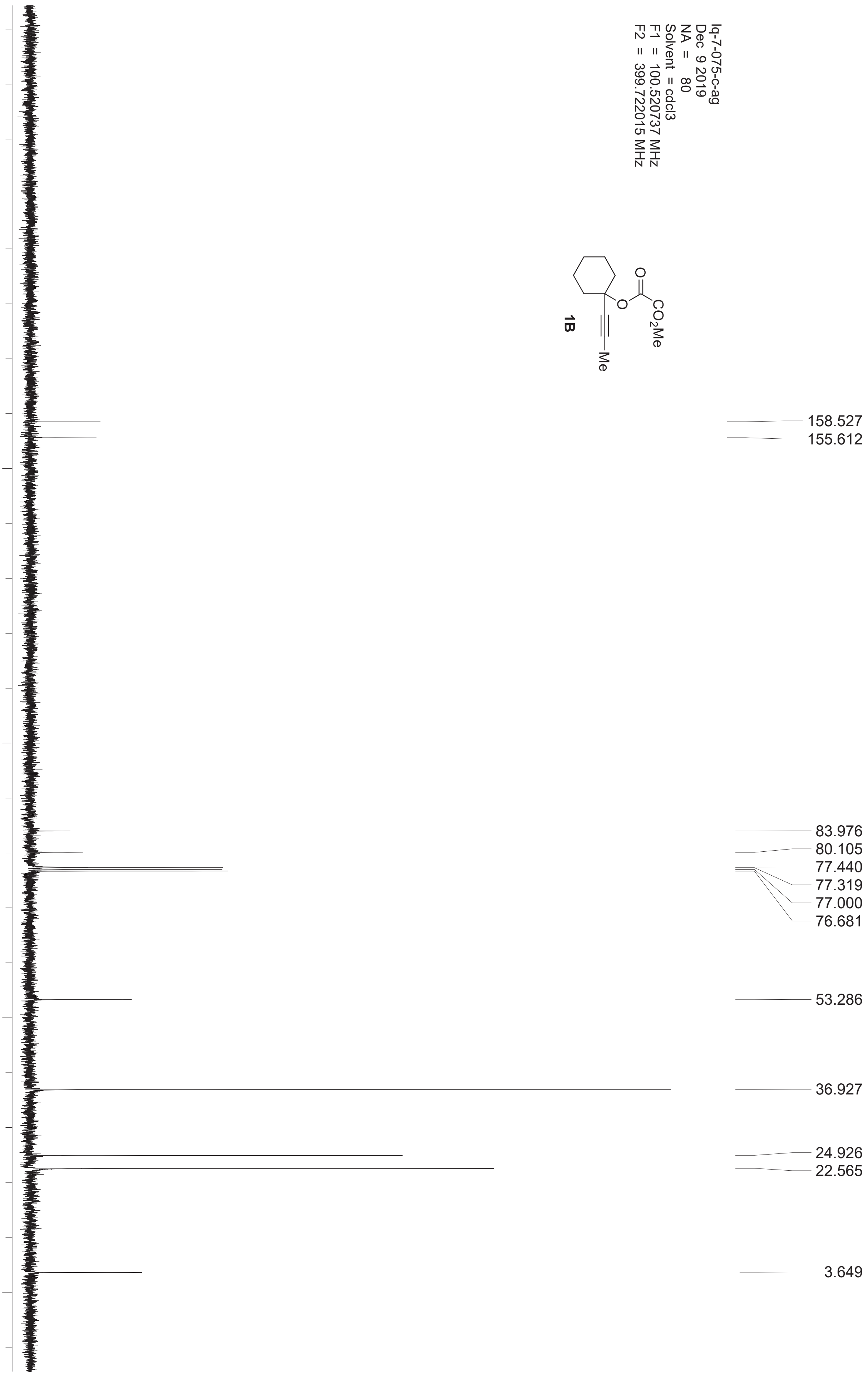

Supplementary Figure 59. ^{13}C NMR (100 MHz, $CDCl_3$) spectrum for **1B**

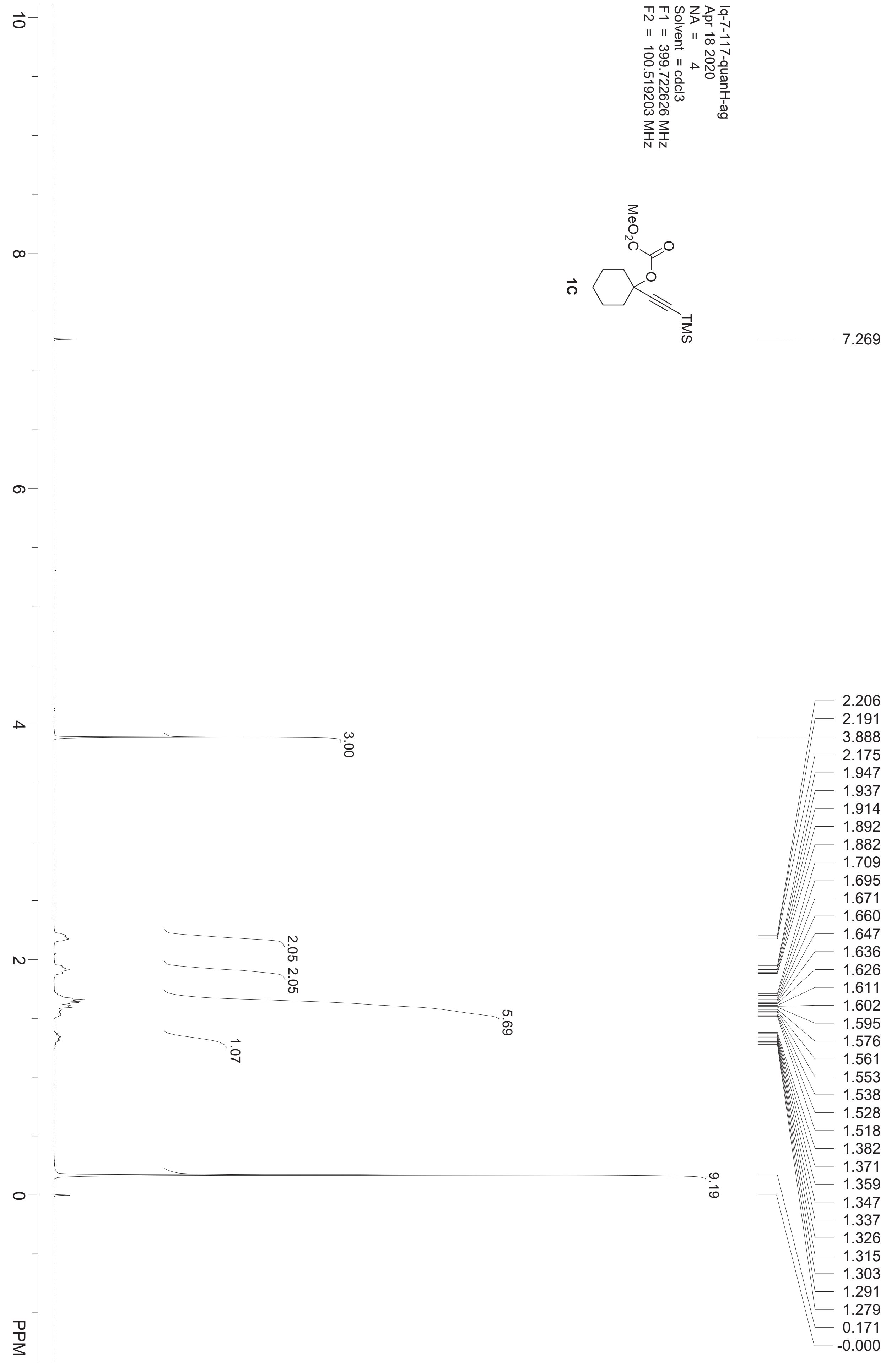

Supplementary Figure 60. ^{1}H NMR (400 MHz, $CDCl_3$) spectrum for **1C**

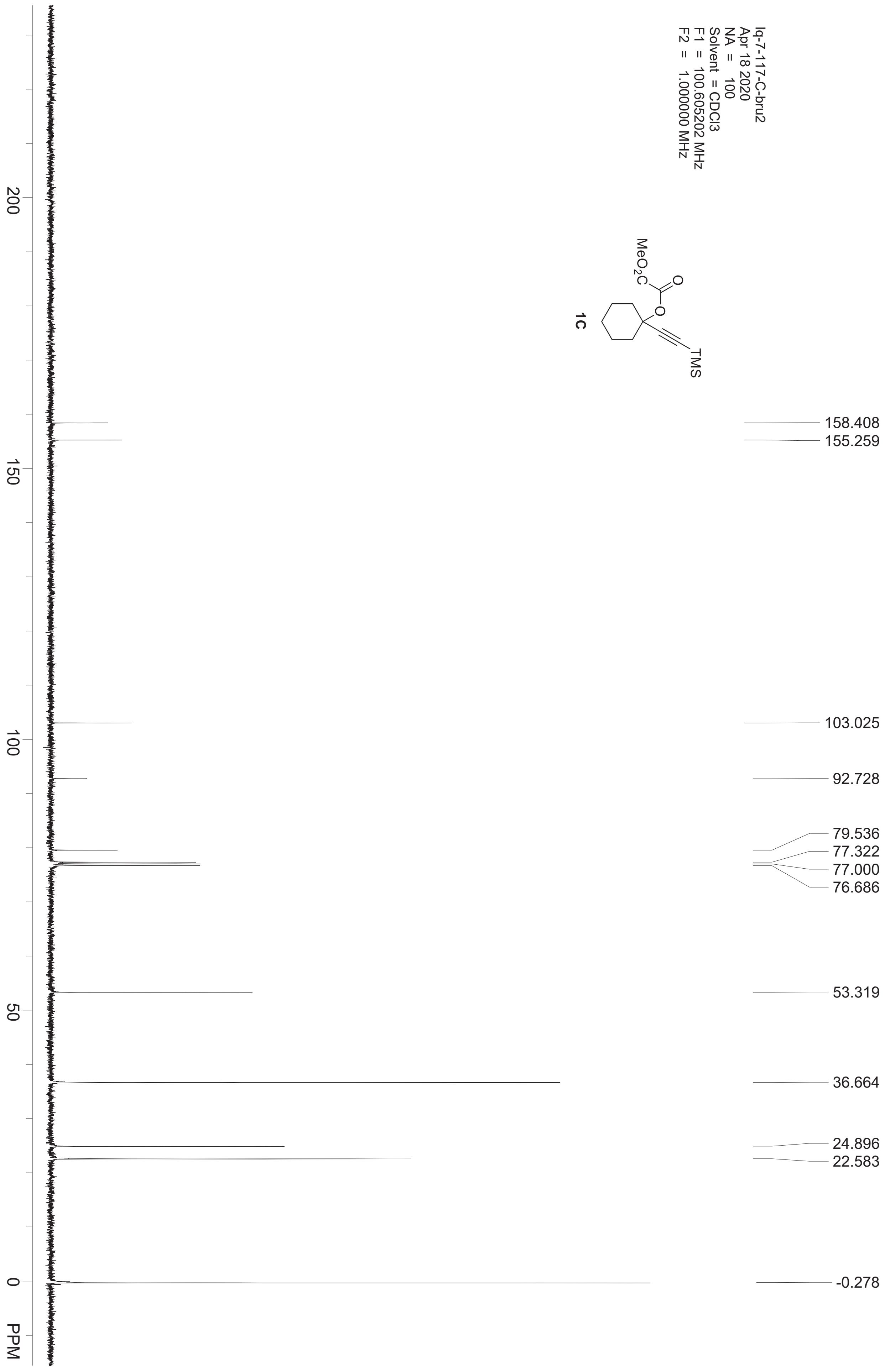

Supplementary Figure 61. ^{13}C NMR (100 MHz, $CDCl_3$) spectrum for **1C**

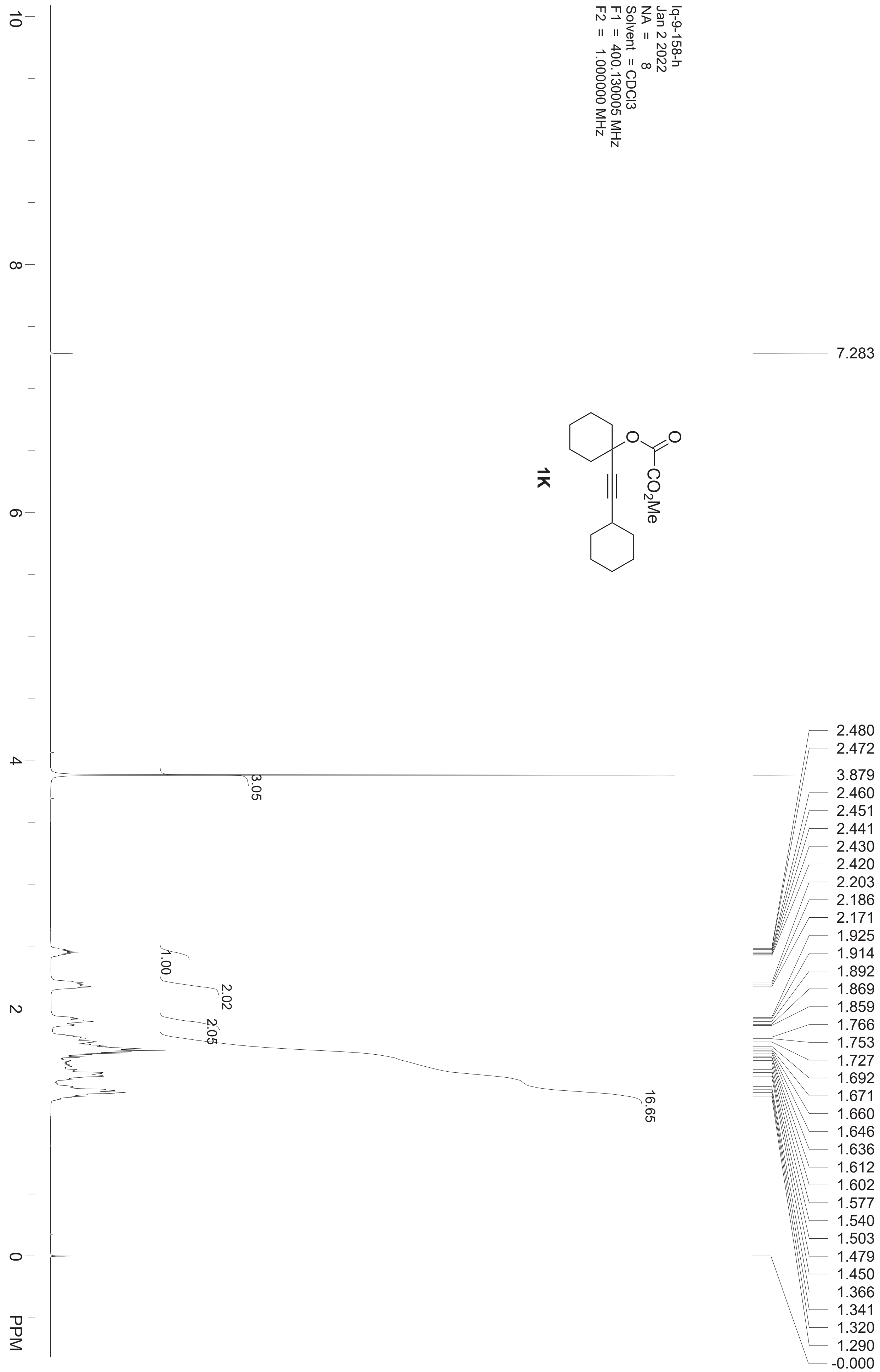

Supplementary Figure 62. ^{1}H NMR (400 MHz, $CDCl_3$) spectrum for **1K**

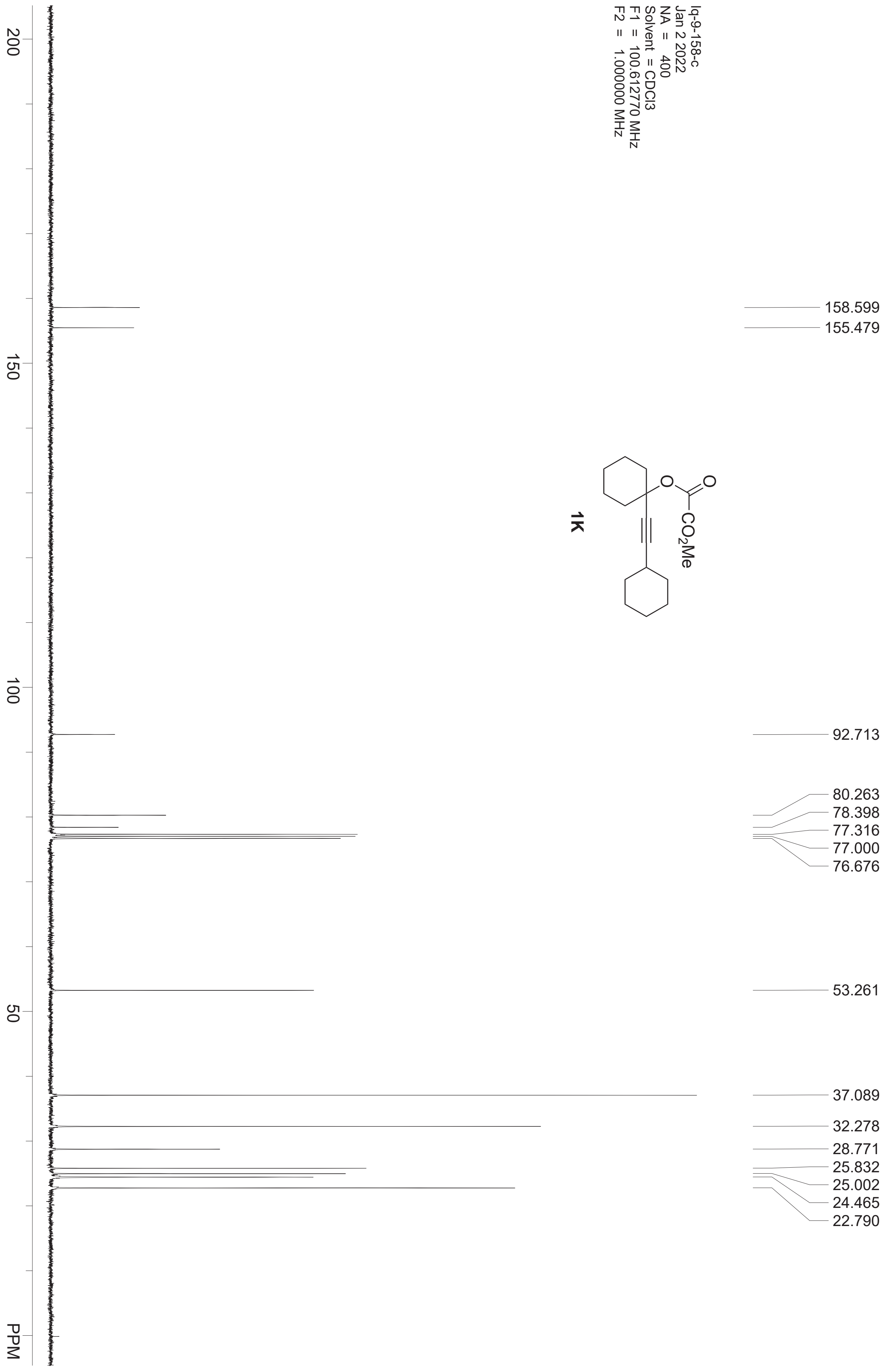

Supplementary Figure 63. ^{13}C NMR (100 MHz, $CDCl_3$) spectrum for **1K**

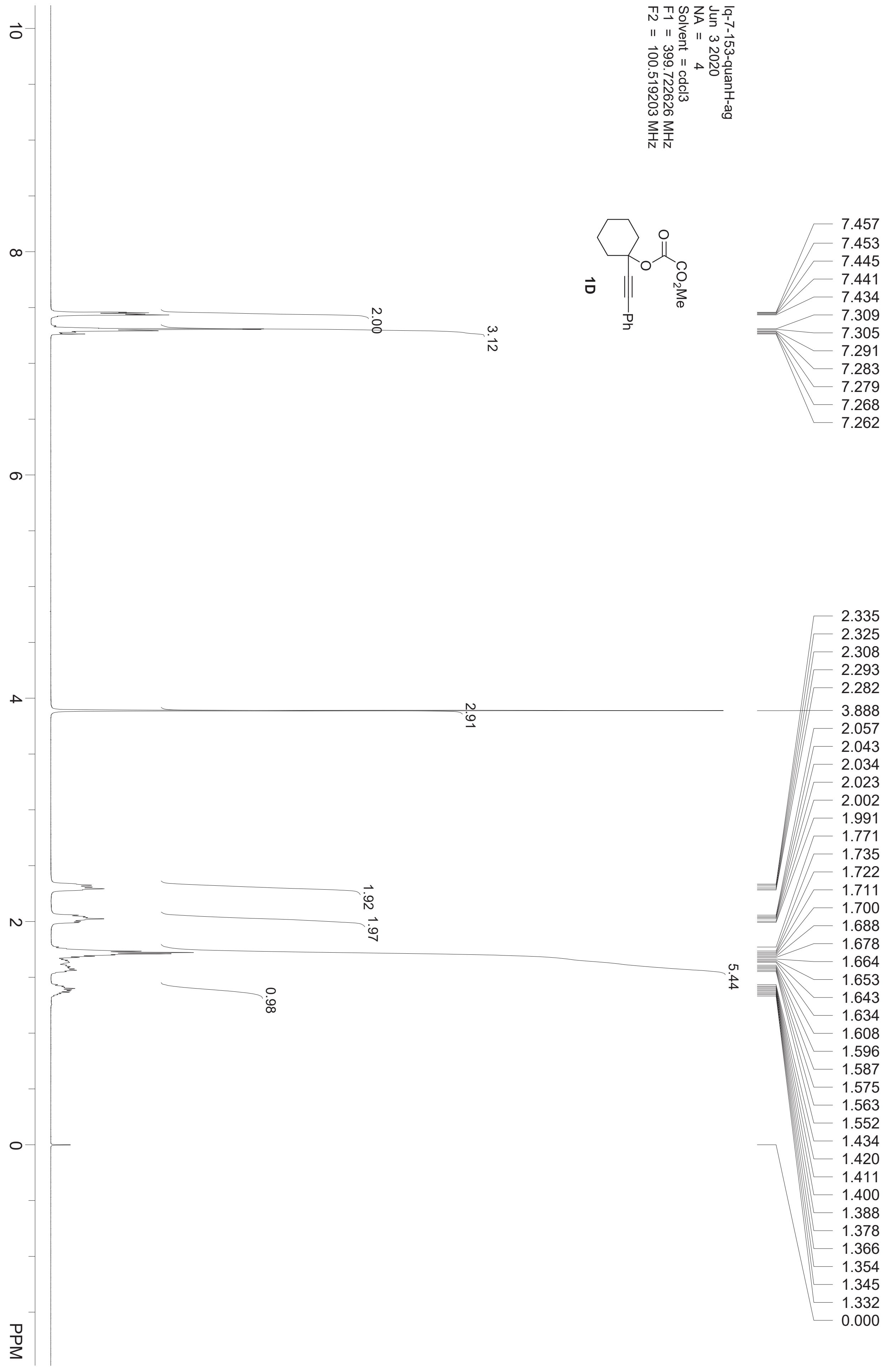

Supplementary Figure 64. ^{1}H NMR (400 MHz, $CDCl_3$) spectrum for **1D**

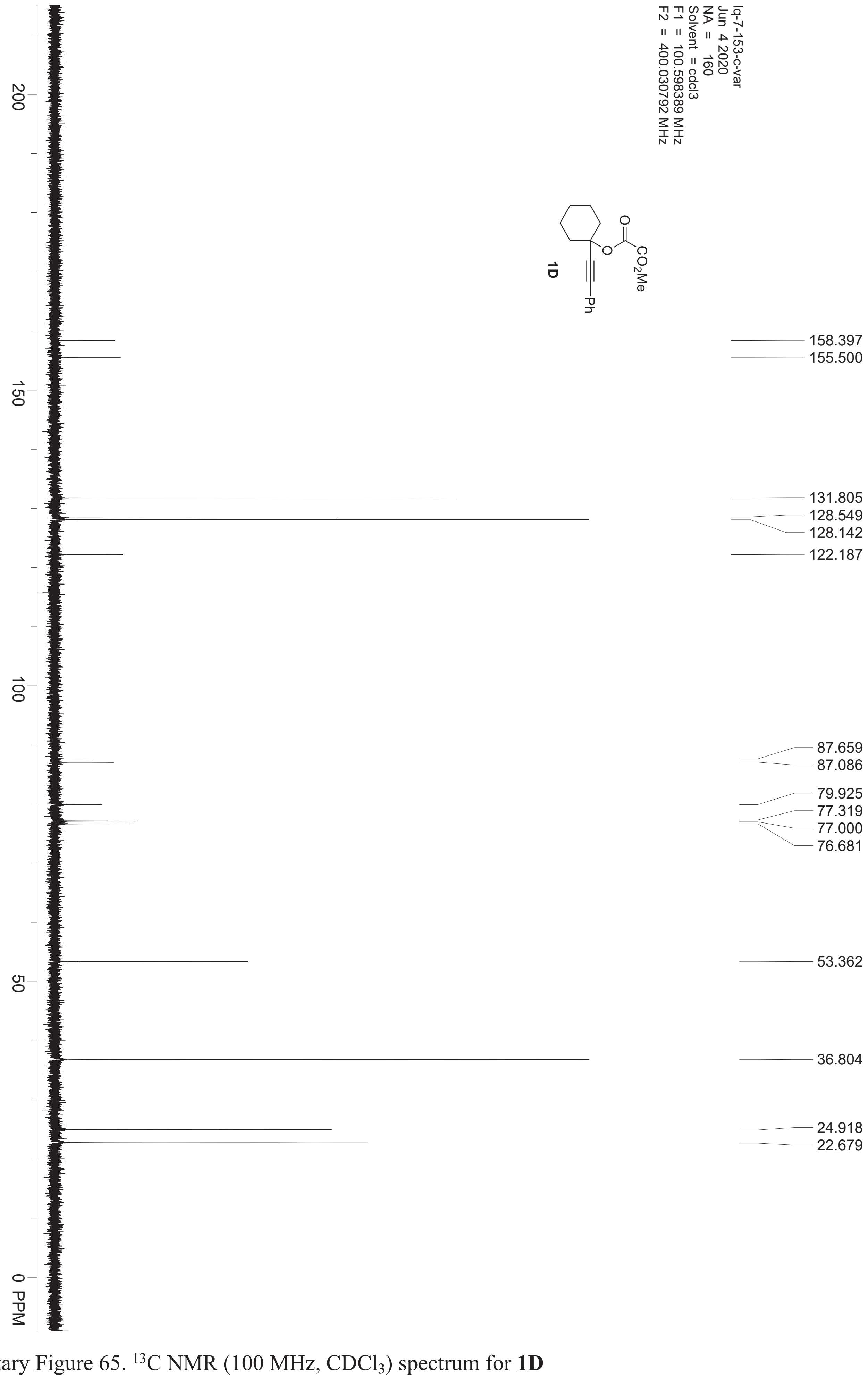

Supplementary Figure 65. ^{13}C NMR (100 MHz, $CDCl_3$) spectrum for **1D**

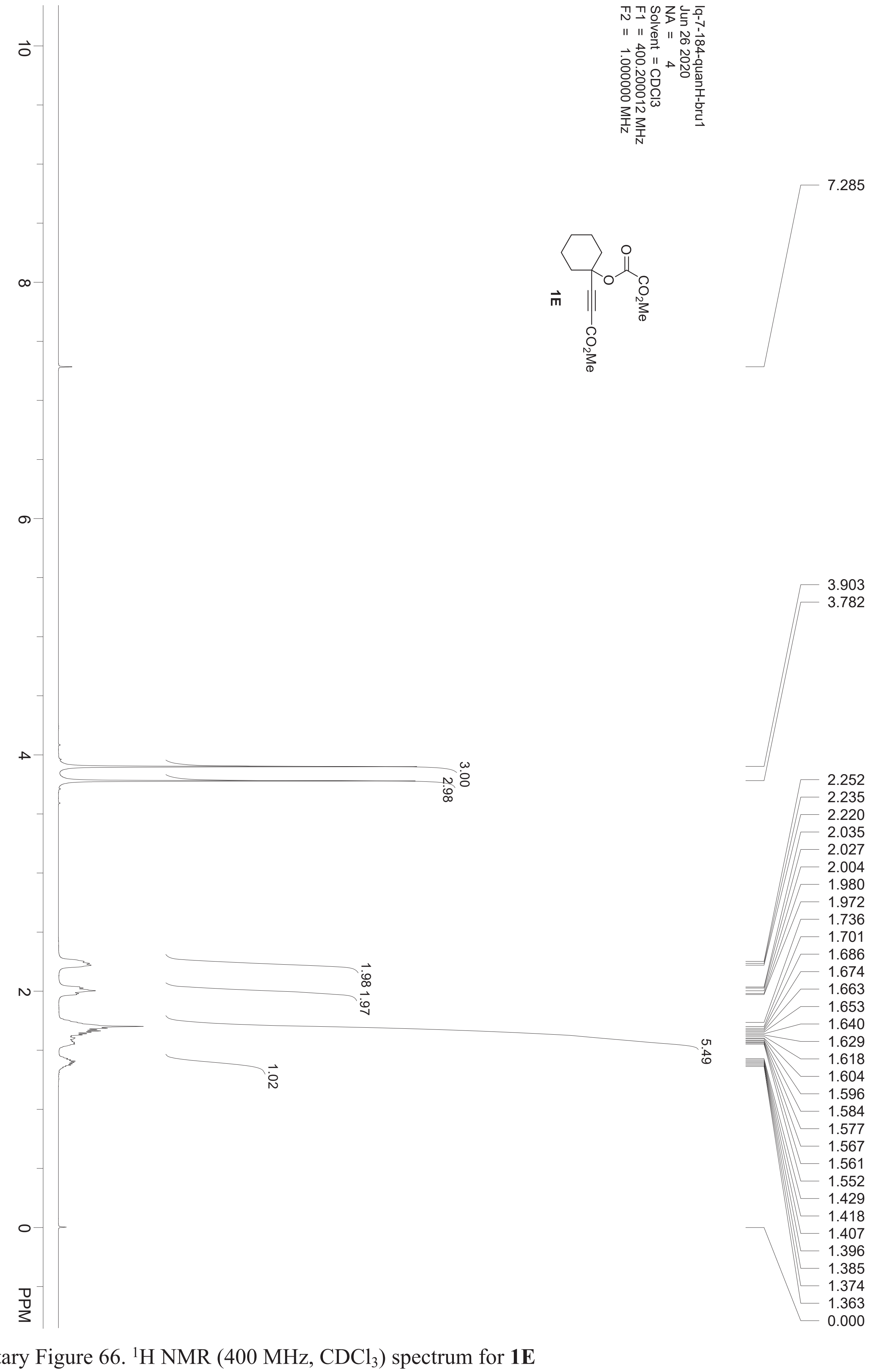

Supplementary Figure 66. ^{1}H NMR (400 MHz, $CDCl_3$) spectrum for **1E**

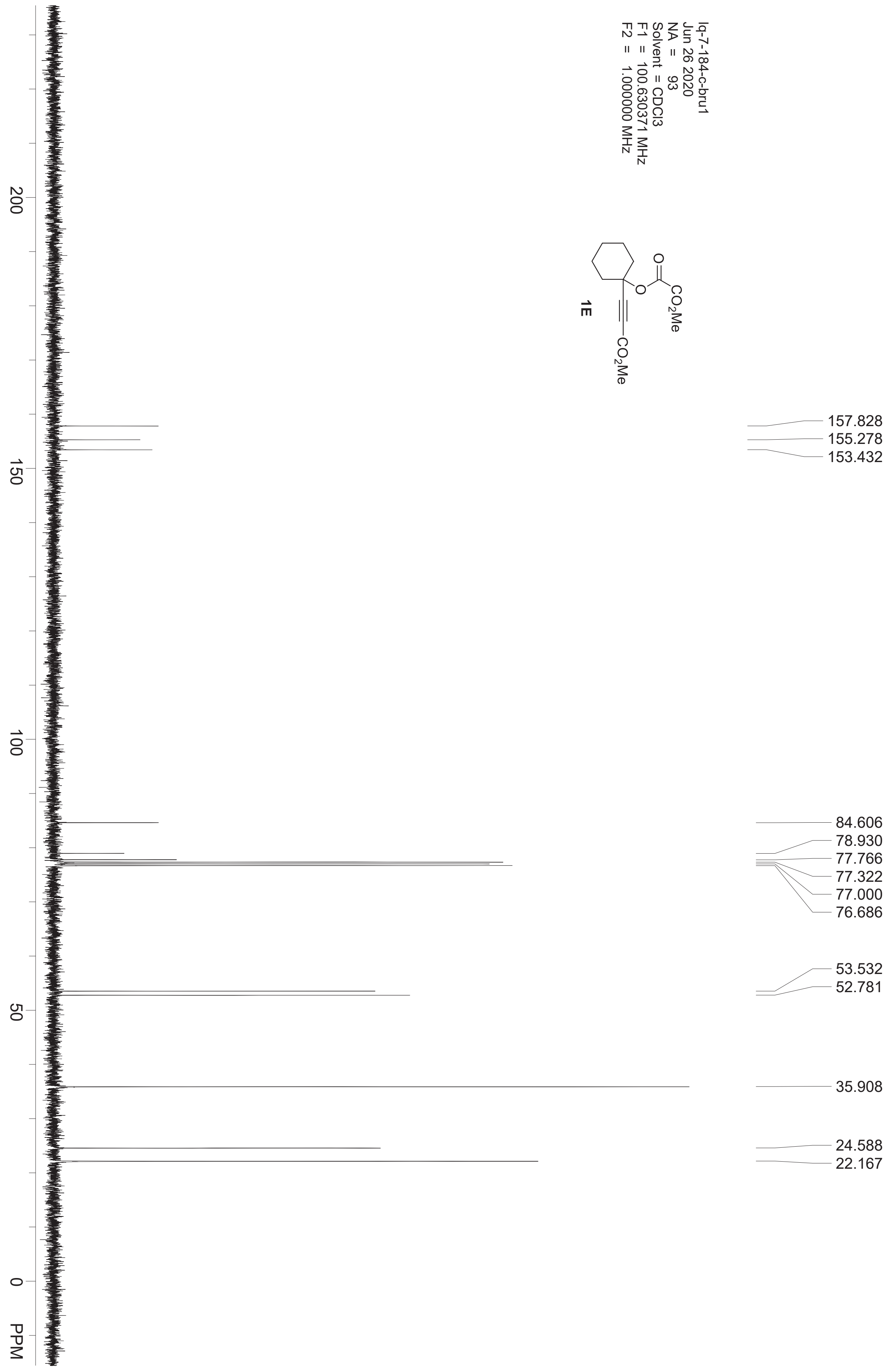

Supplementary Figure 67. ^{13}C NMR (100 MHz, $CDCl_3$) spectrum for **1E**

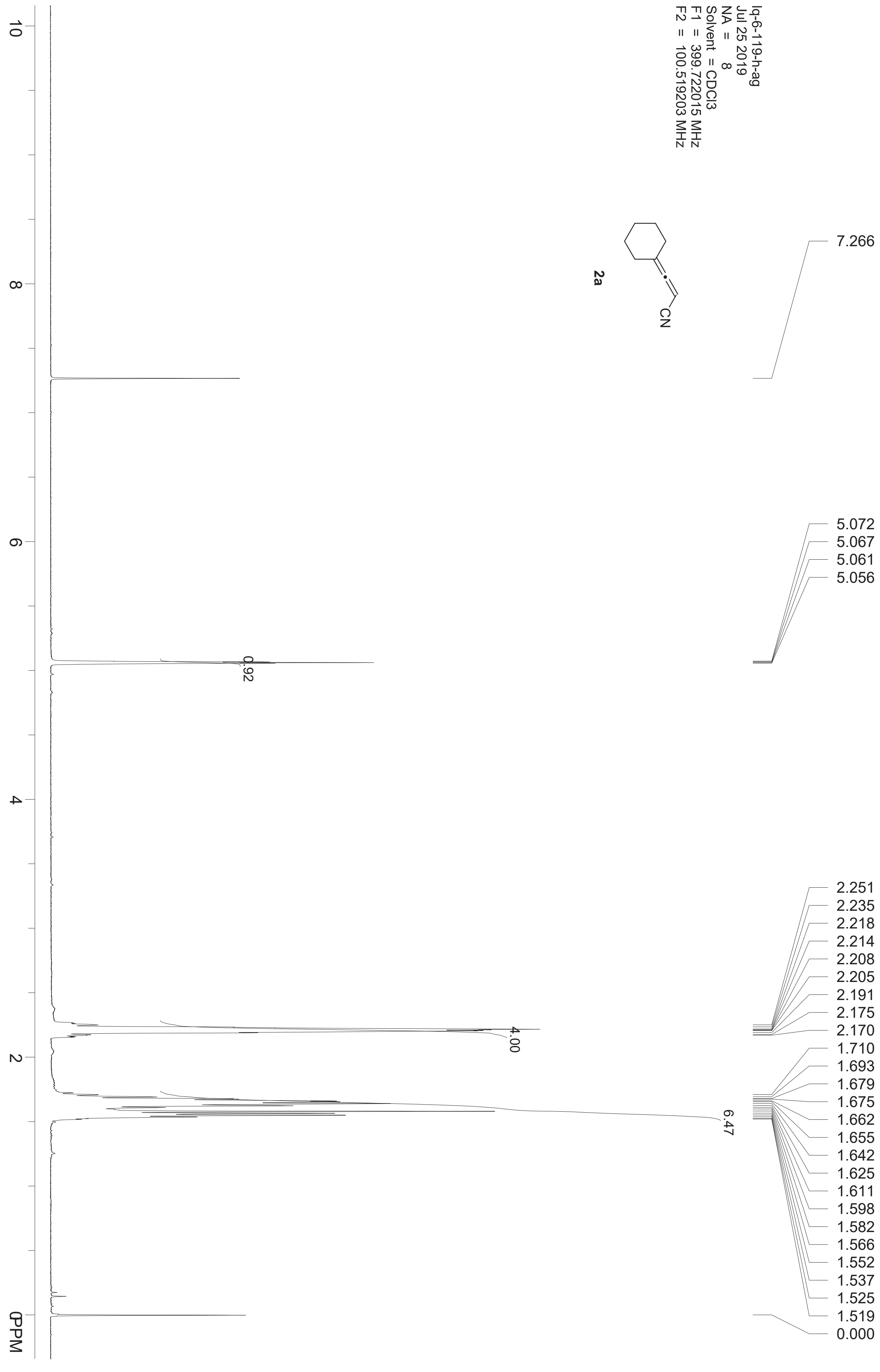

Supplementary Figure 68. ^{1}H NMR (400 MHz, $CDCl_3$) spectrum for **2a**

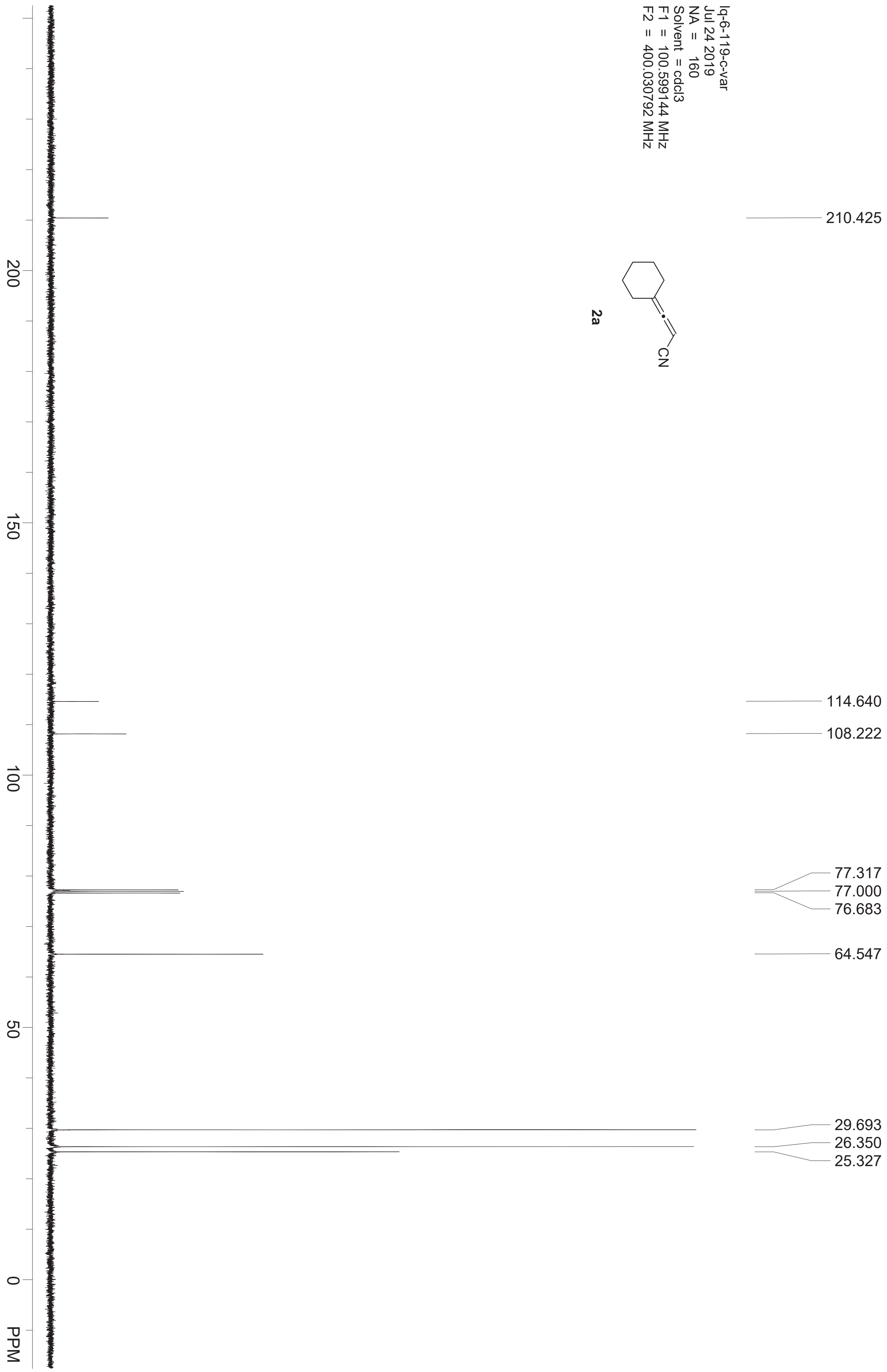

Supplementary Figure 69. ^{13}C NMR 100 MHz, $CDCl_3$) spectrum for **2a**

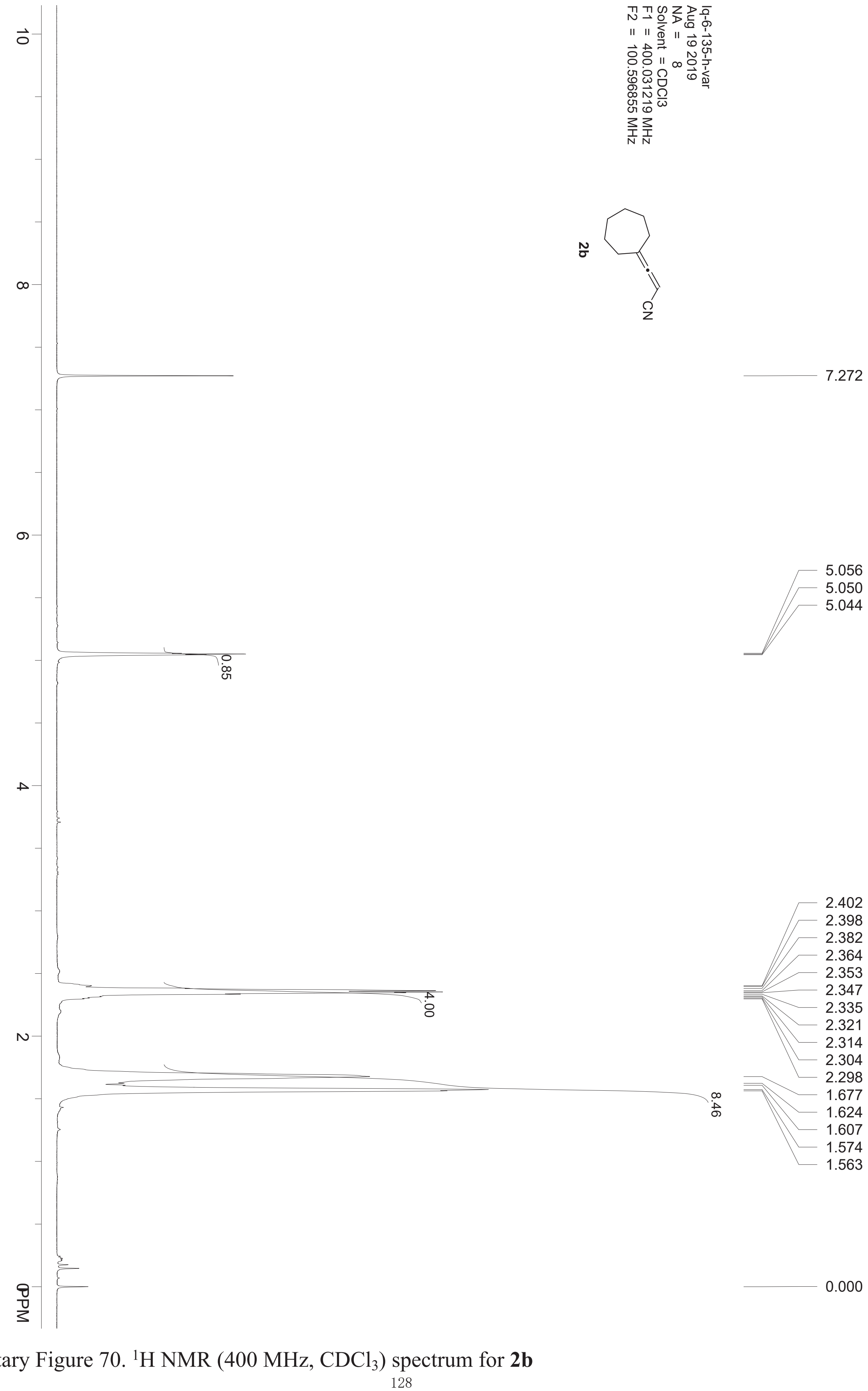

Supplementary Figure 70. ^{1}H NMR (400 MHz, $CDCl_3$) spectrum for **2b**

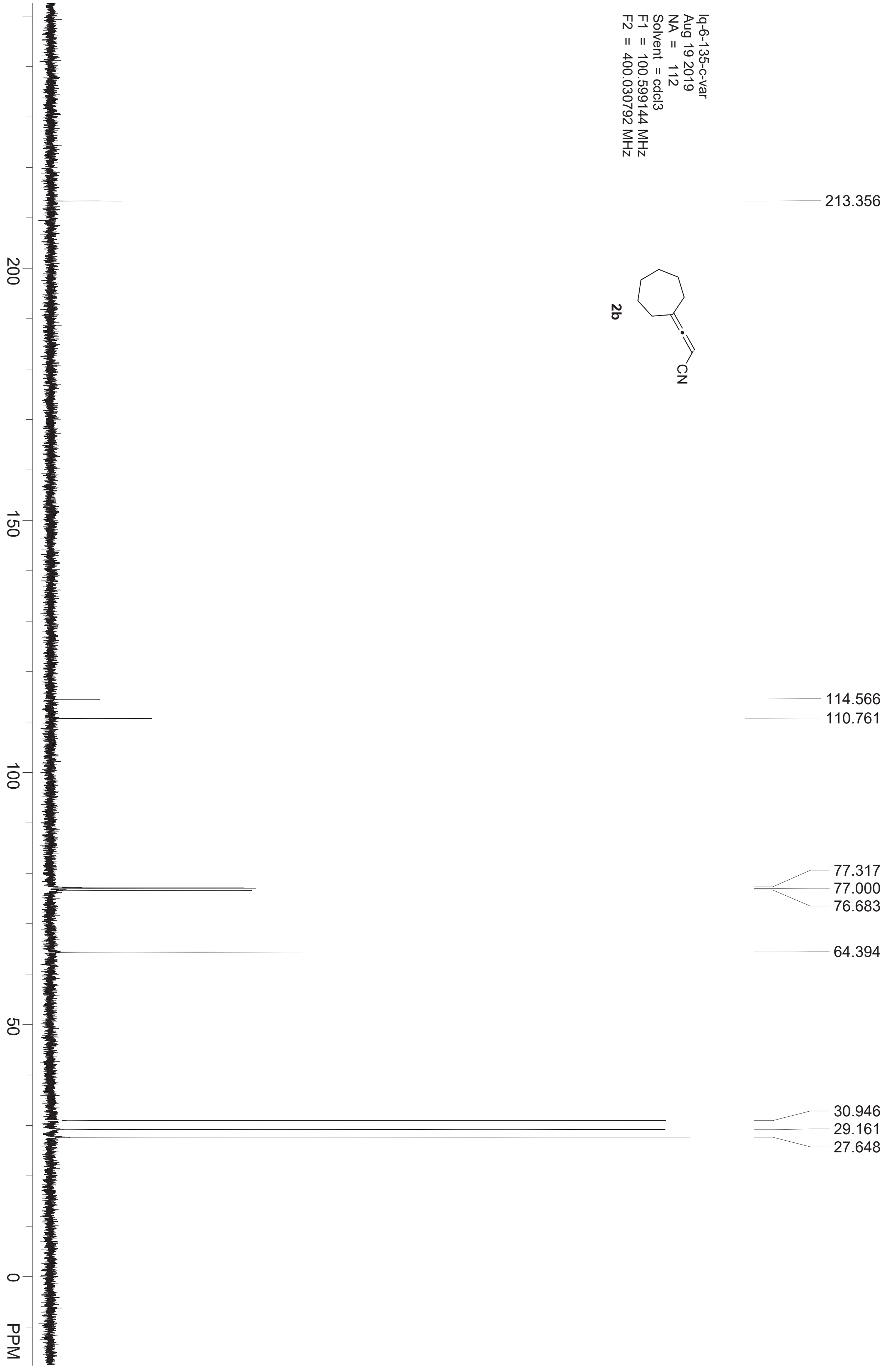

Supplementary Figure 71. ^{13}C NMR (100 MHz, $CDCl_3$) spectrum for **2b**

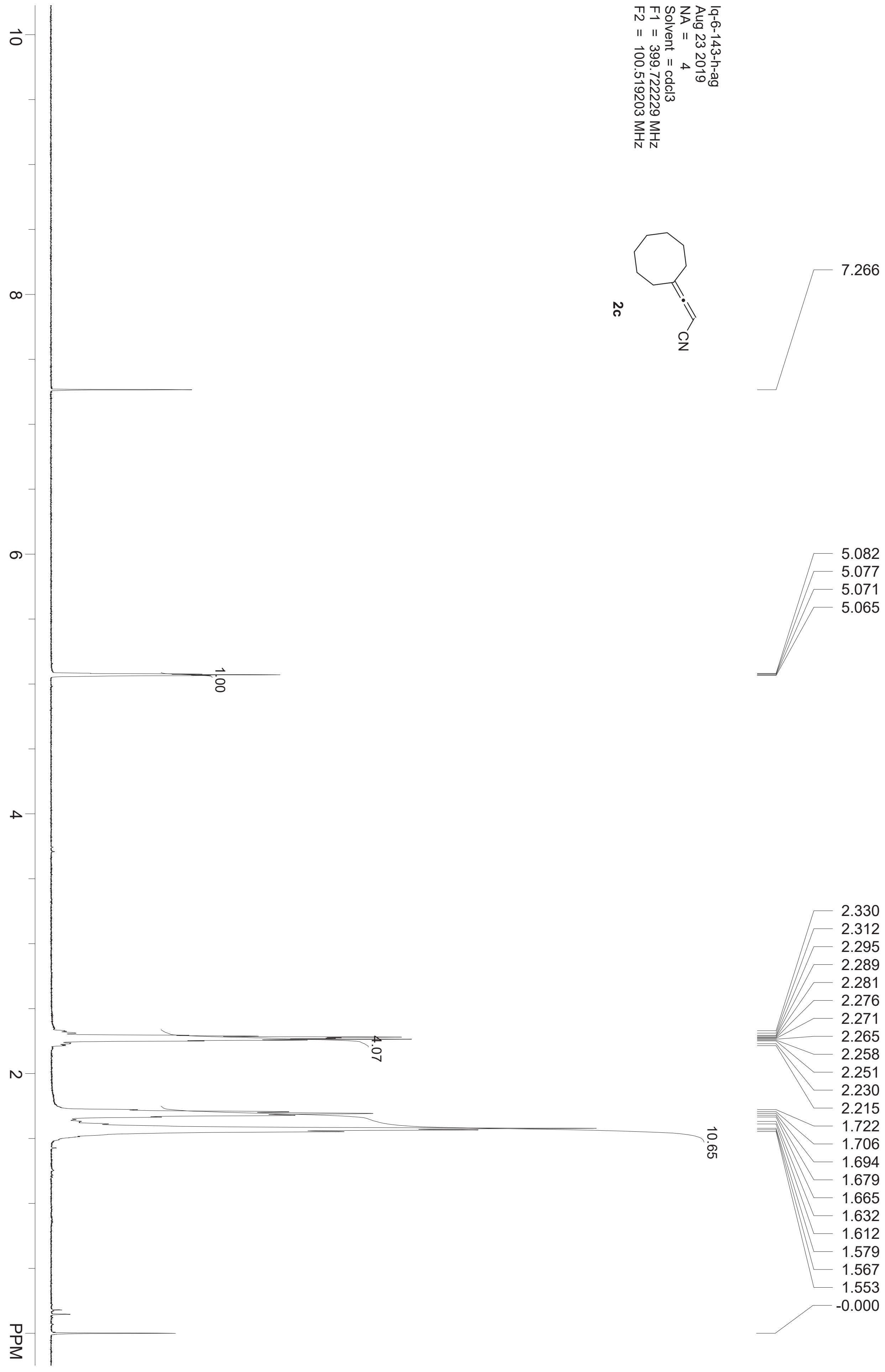

Supplementary Figure 72. ^{1}H NMR (400 MHz, $CDCl_3$) spectrum for **2c**

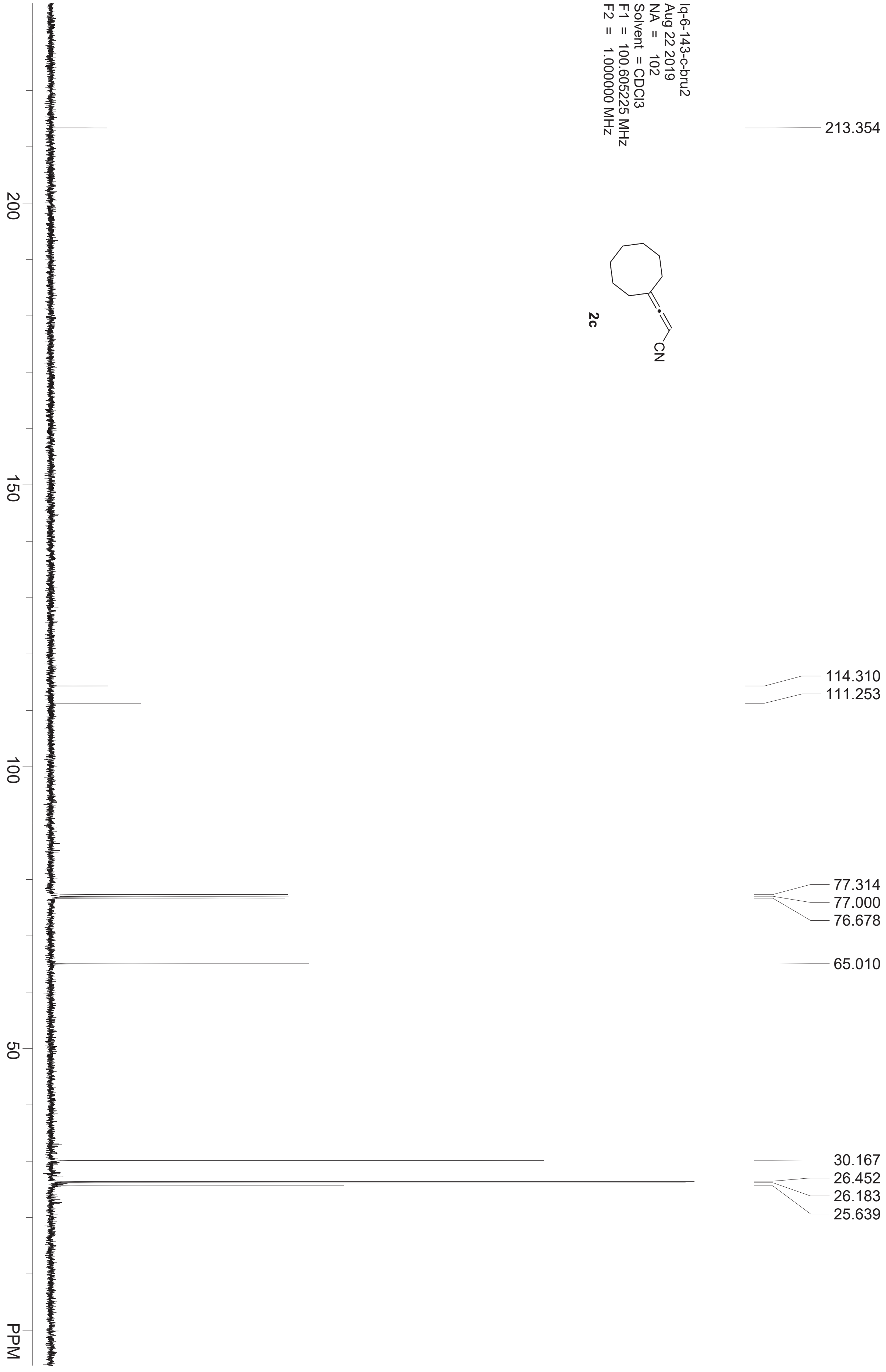

Supplementary Figure 73. ^{13}C NMR (100 MHz, $CDCl_3$) spectrum for **2c**

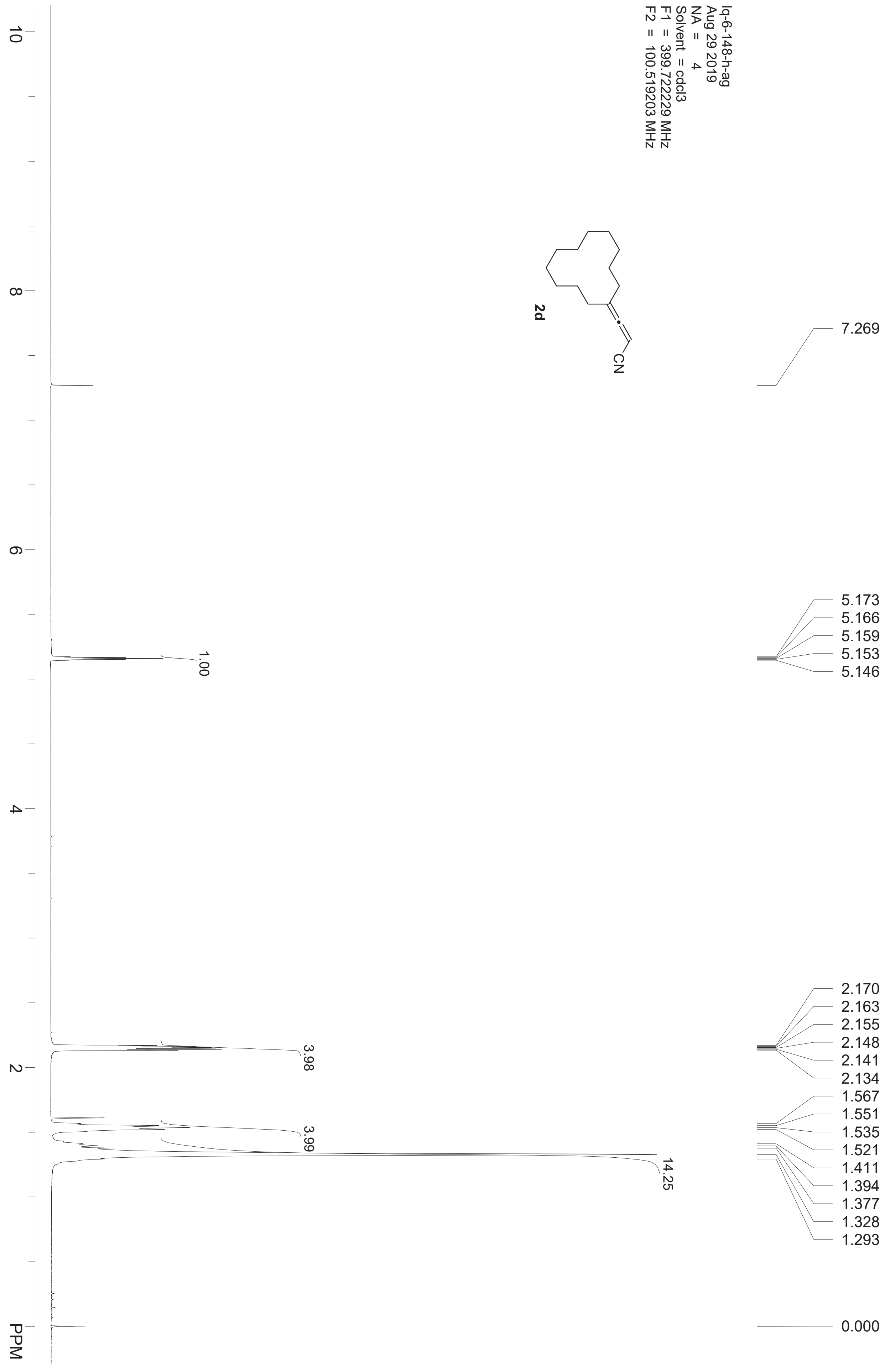

Supplementary Figure 74. ^{1}H NMR (400 MHz, $CDCl_3$) spectrum for **2d**

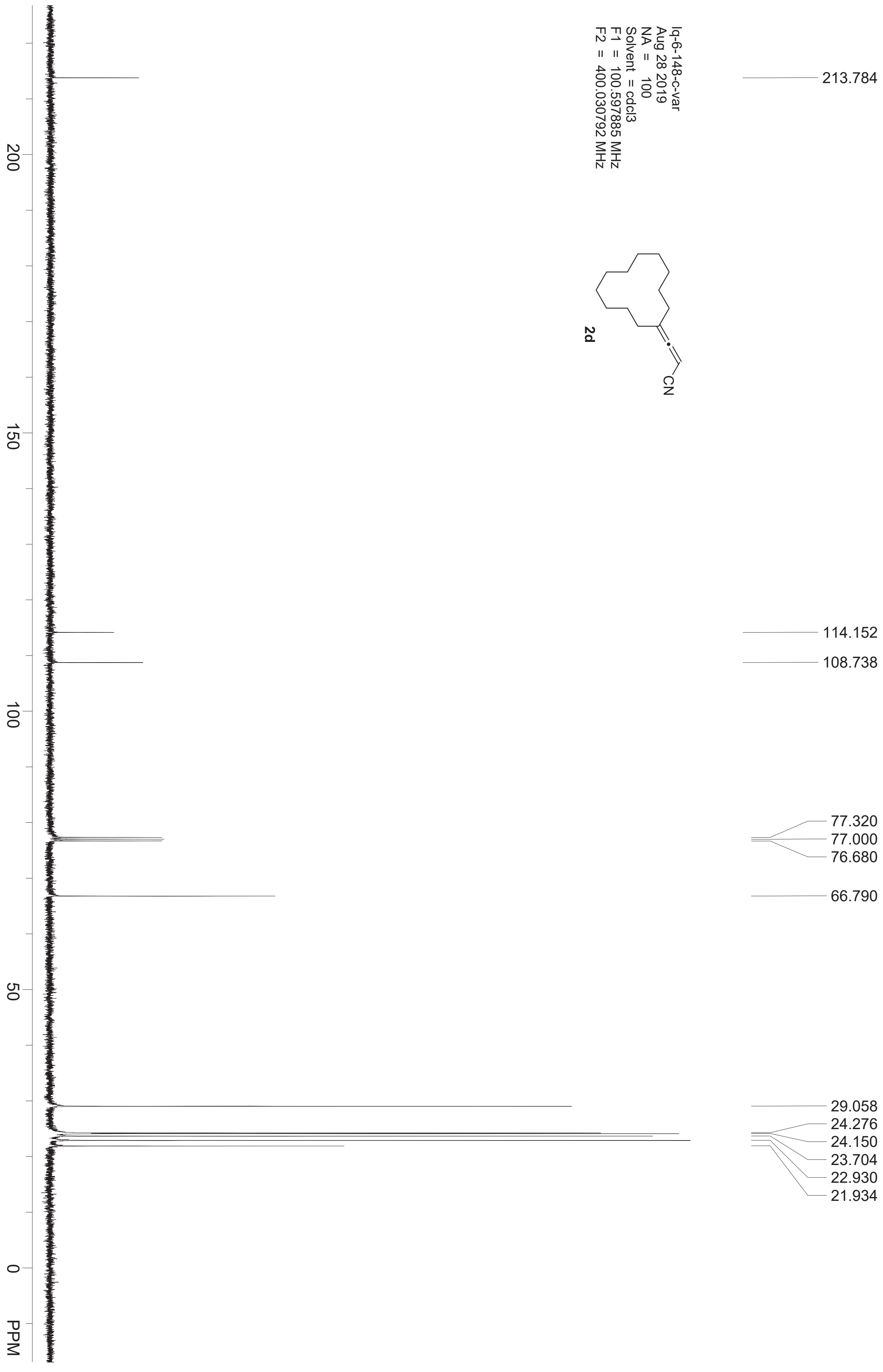

Supplementary Figure 75. ^{13}C NMR (100 MHz, $CDCl_3$) spectrum for **2d**

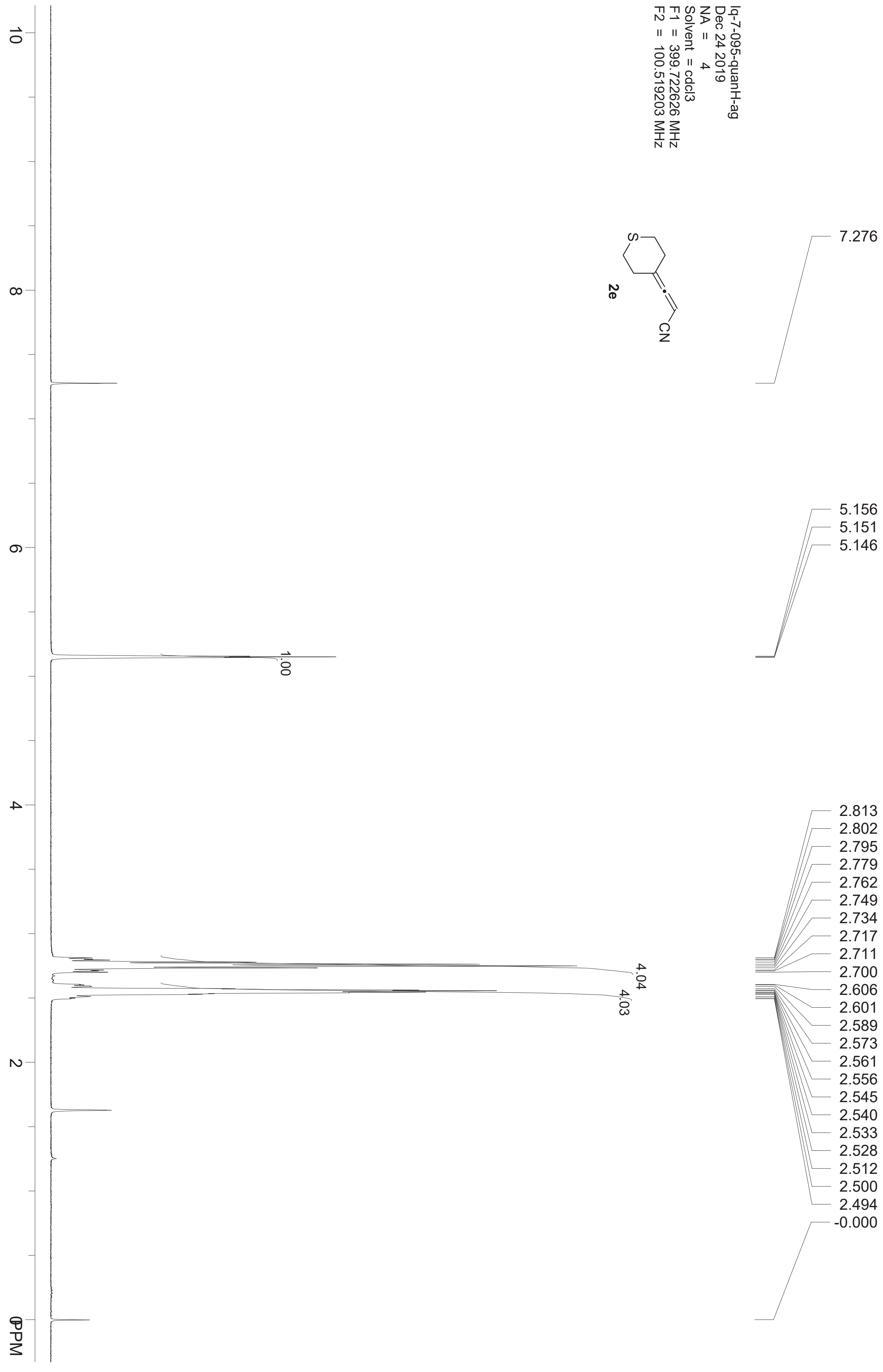

Supplementary Figure 76. ^{1}H NMR (400 MHz, $CDCl_3$) spectrum for **2e**

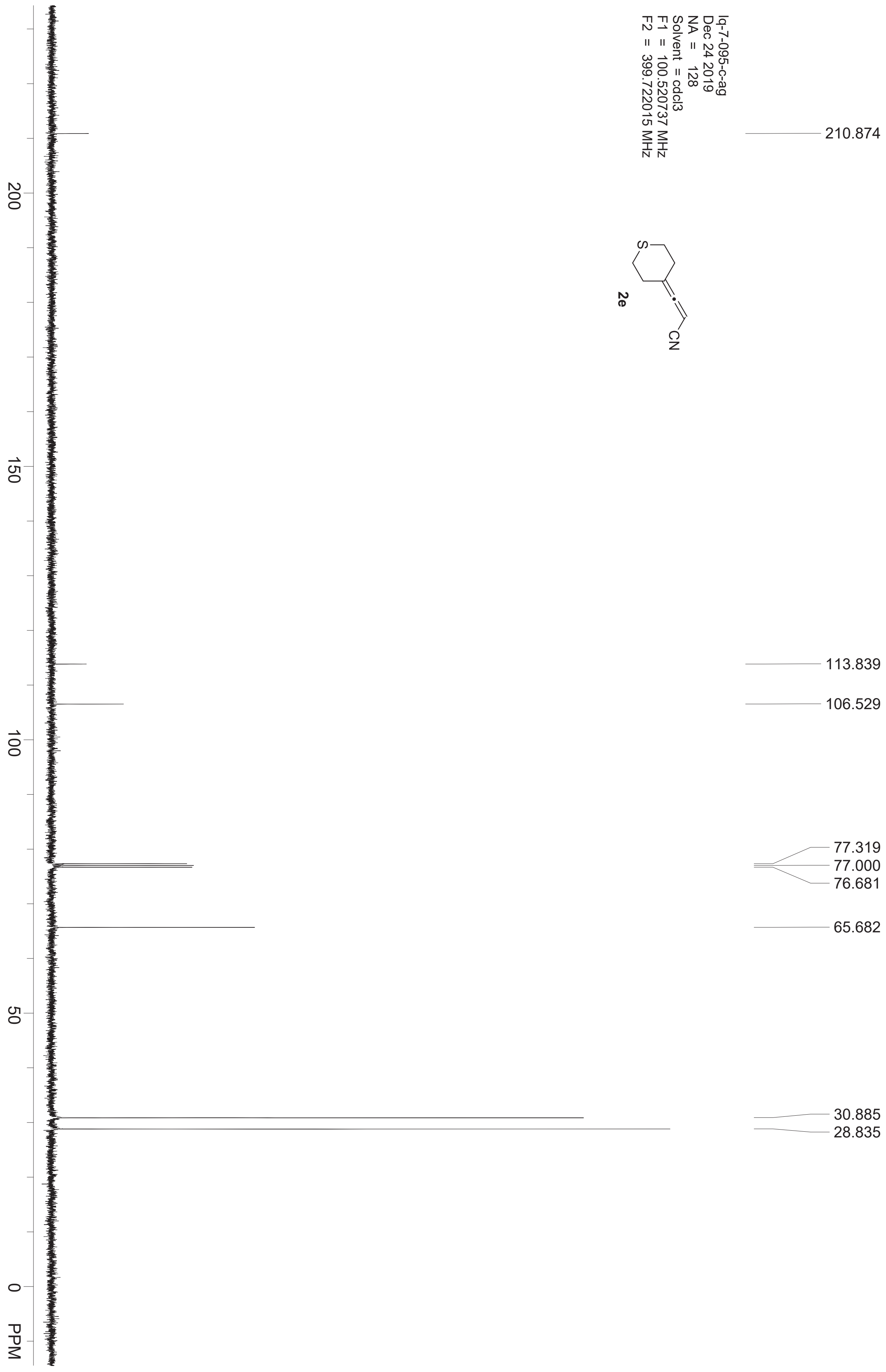

Supplementary Figure 77. ^{13}C NMR (100 MHz, $CDCl_3$) spectrum for **2e**

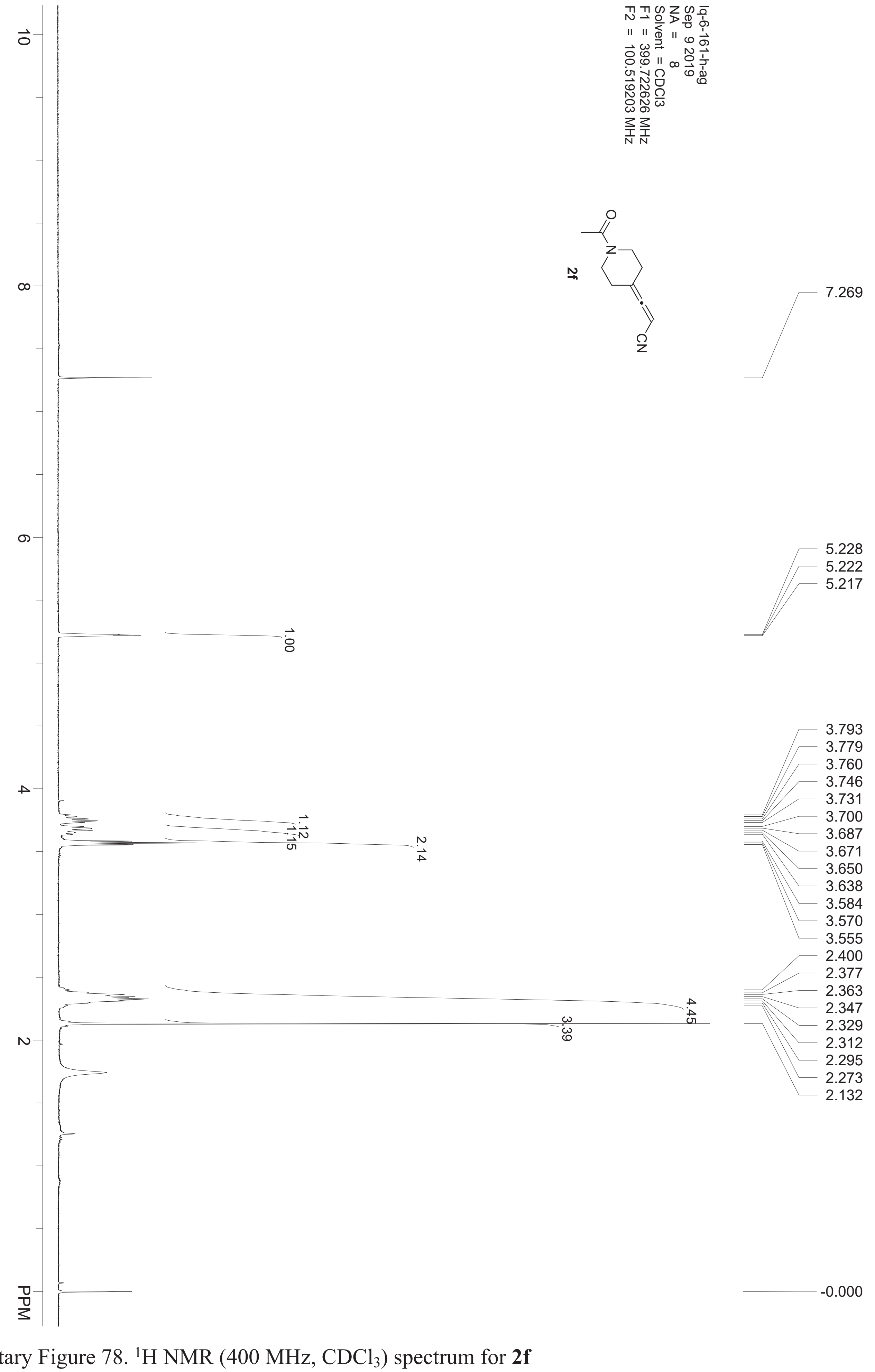

Supplementary Figure 78. ^{1}H NMR (400 MHz, $CDCl_3$) spectrum for **2f**

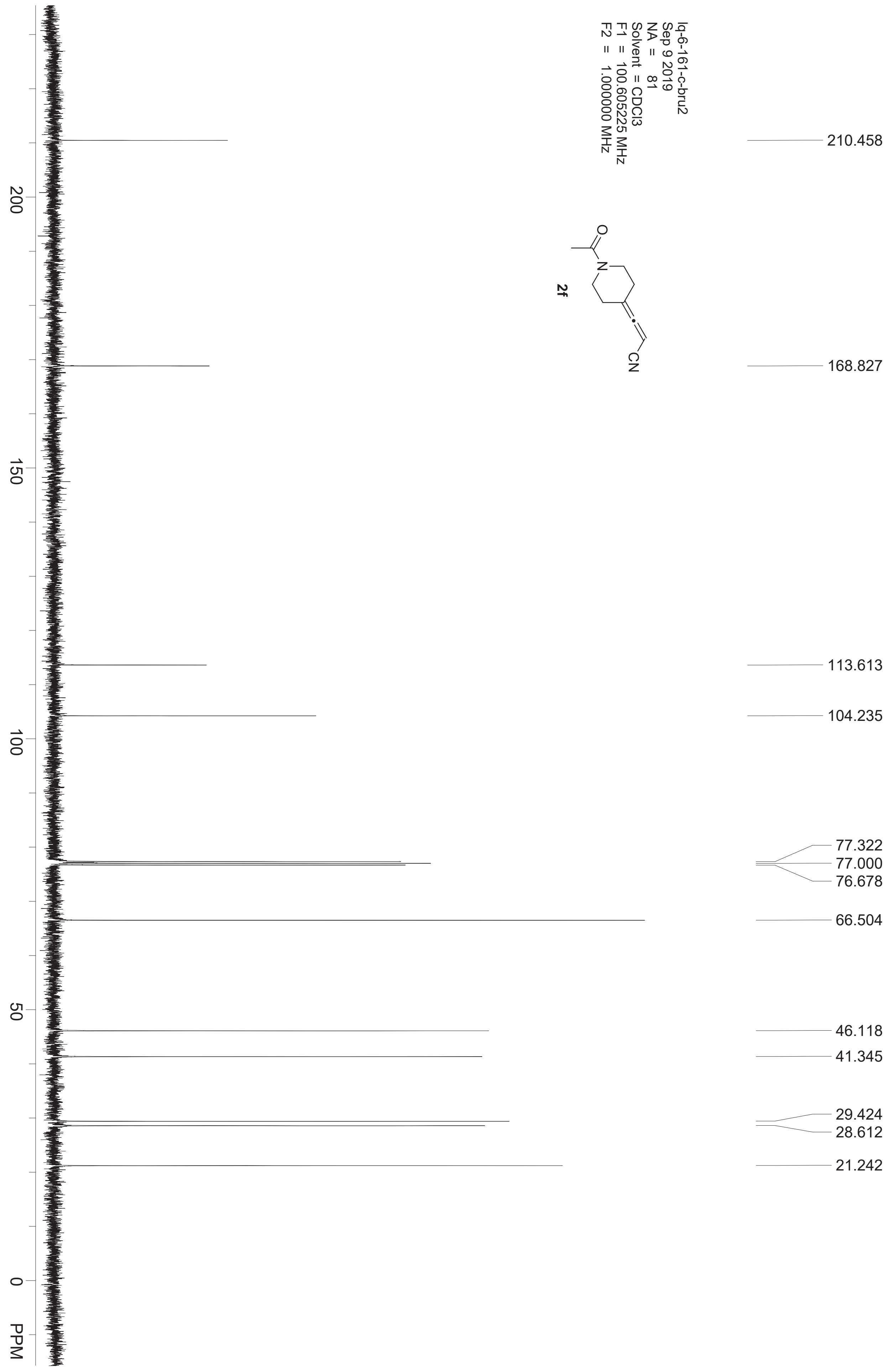

Supplementary Figure 79. ^{13}C NMR (100 MHz, $CDCl_3$) spectrum for **2f**

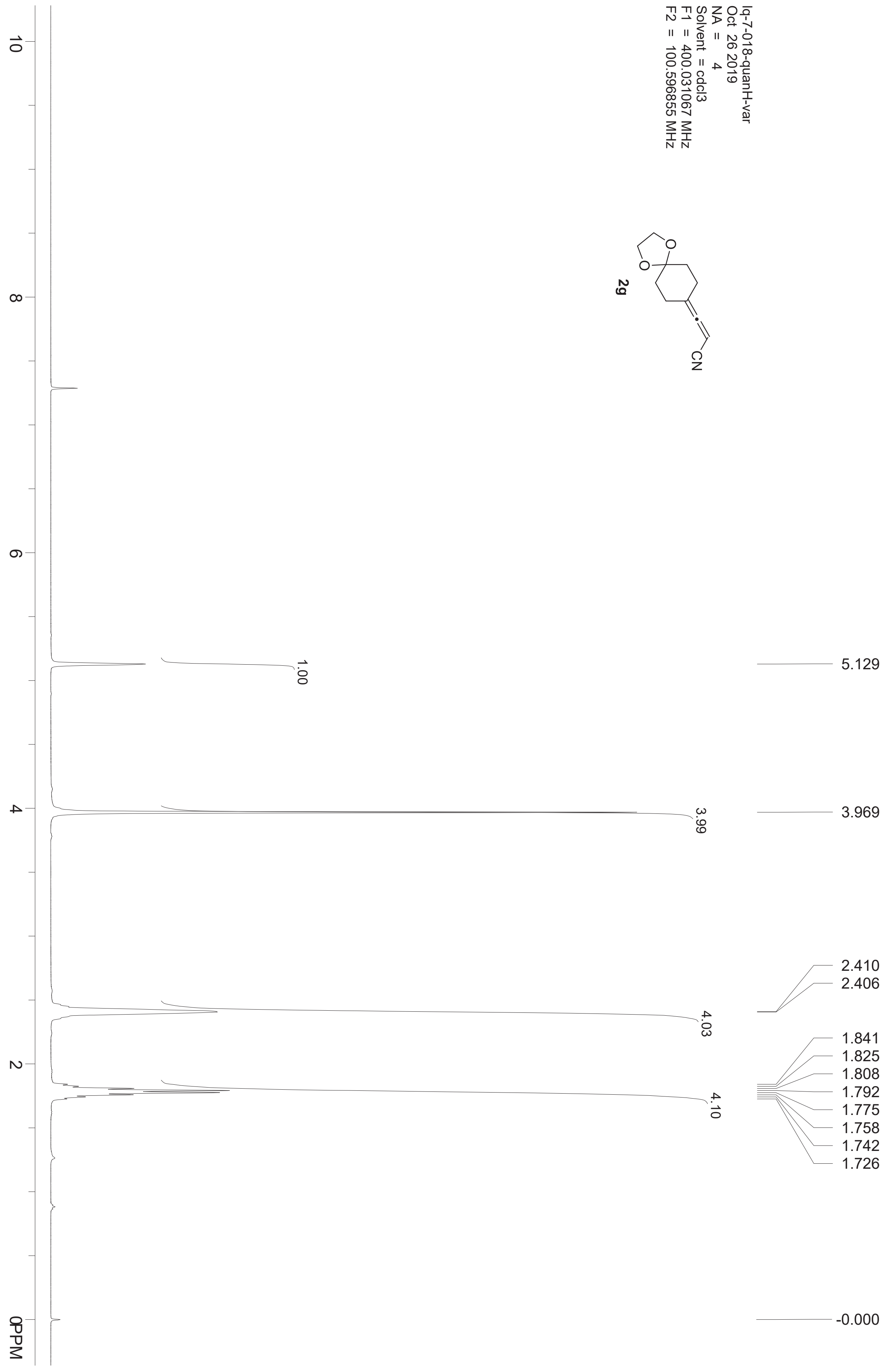

Supplementary Figure 80. ^{1}H NMR (400 MHz, $CDCl_3$) spectrum for **2g**

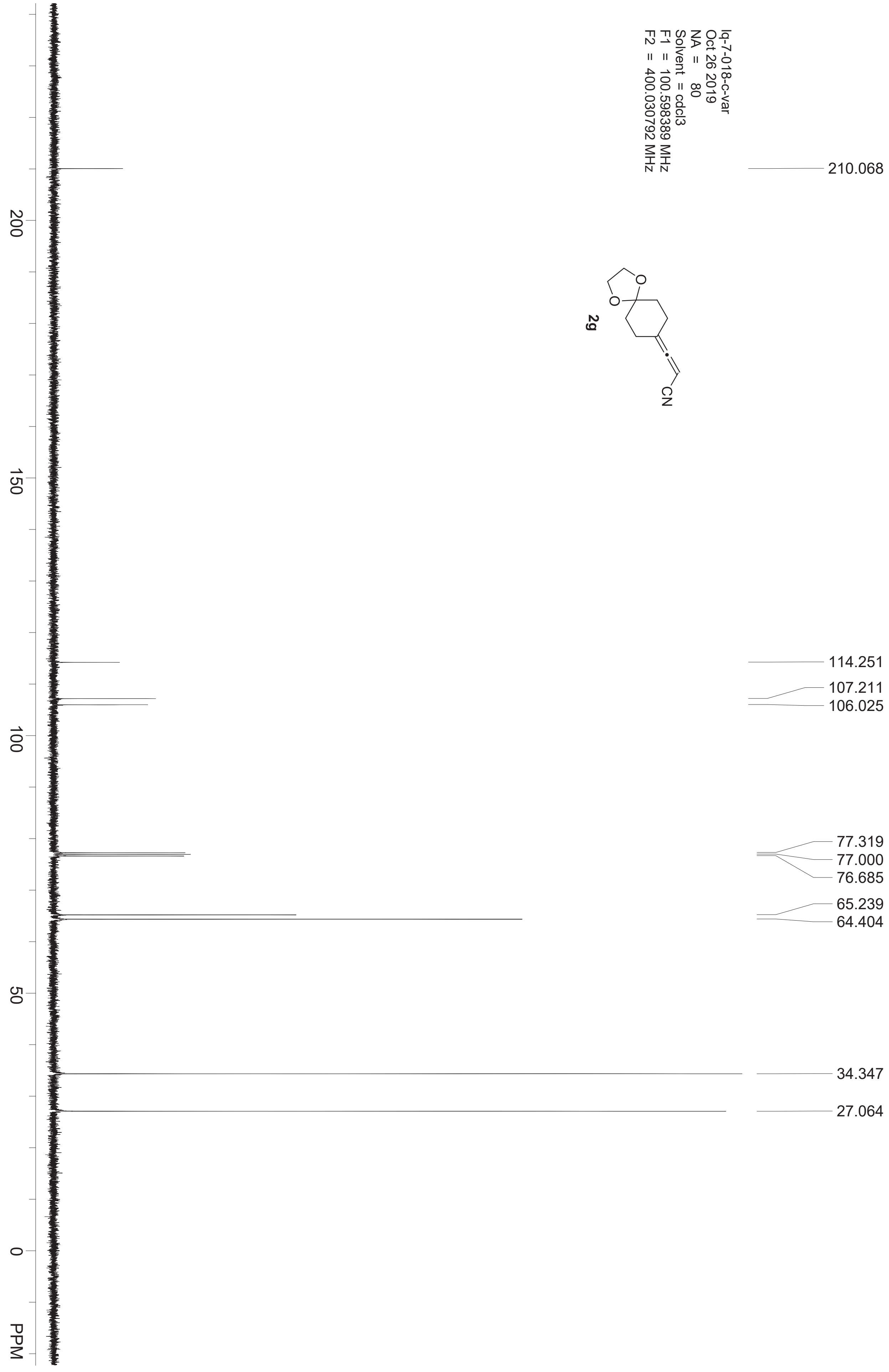

Supplementary Figure 81. ^{13}C NMR (100 MHz, $CDCl_3$) spectrum for **2g**

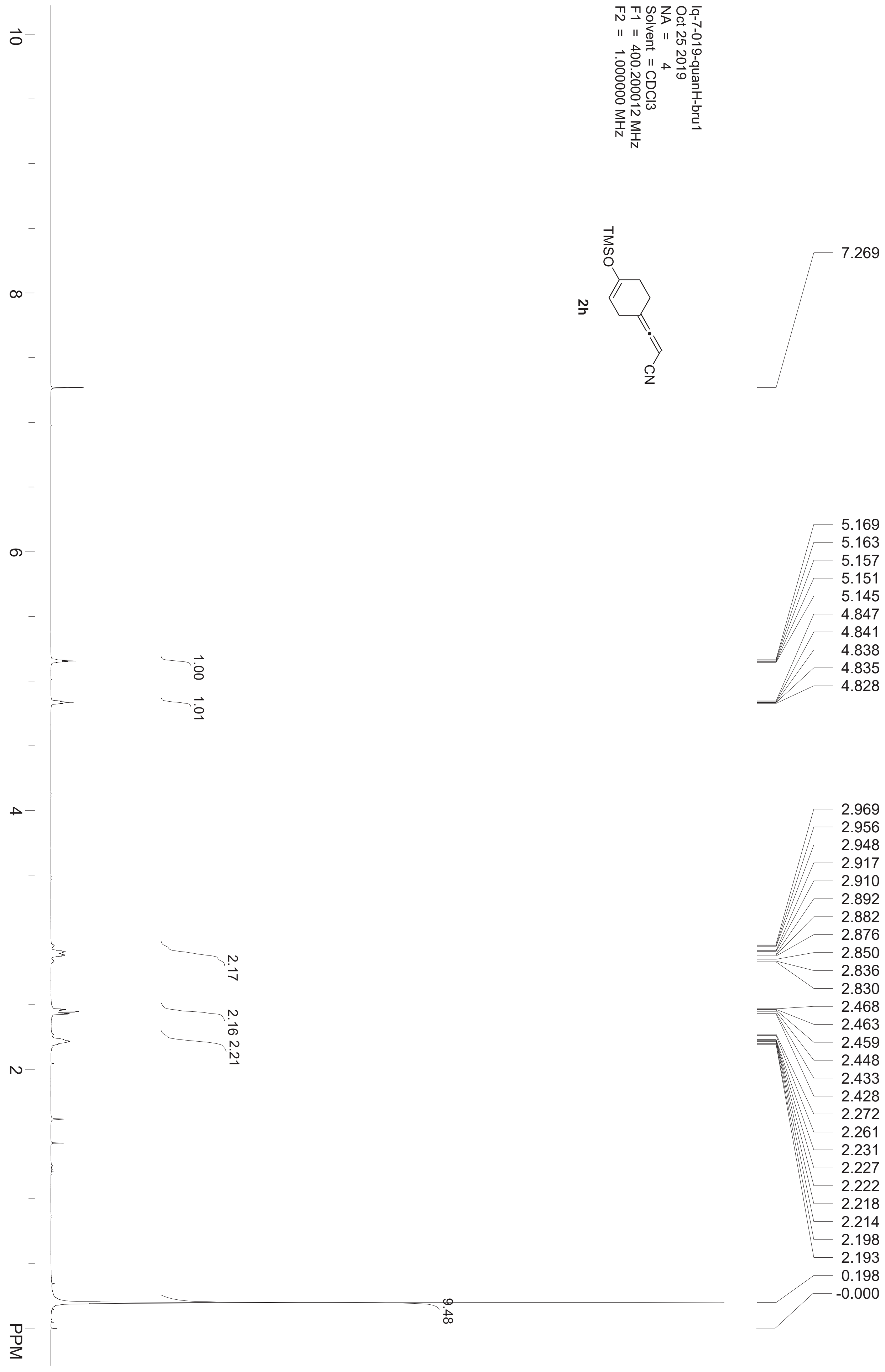

Supplementary Figure 82. ^{1}H NMR (400 MHz, $CDCl_3$) spectrum for **2h**

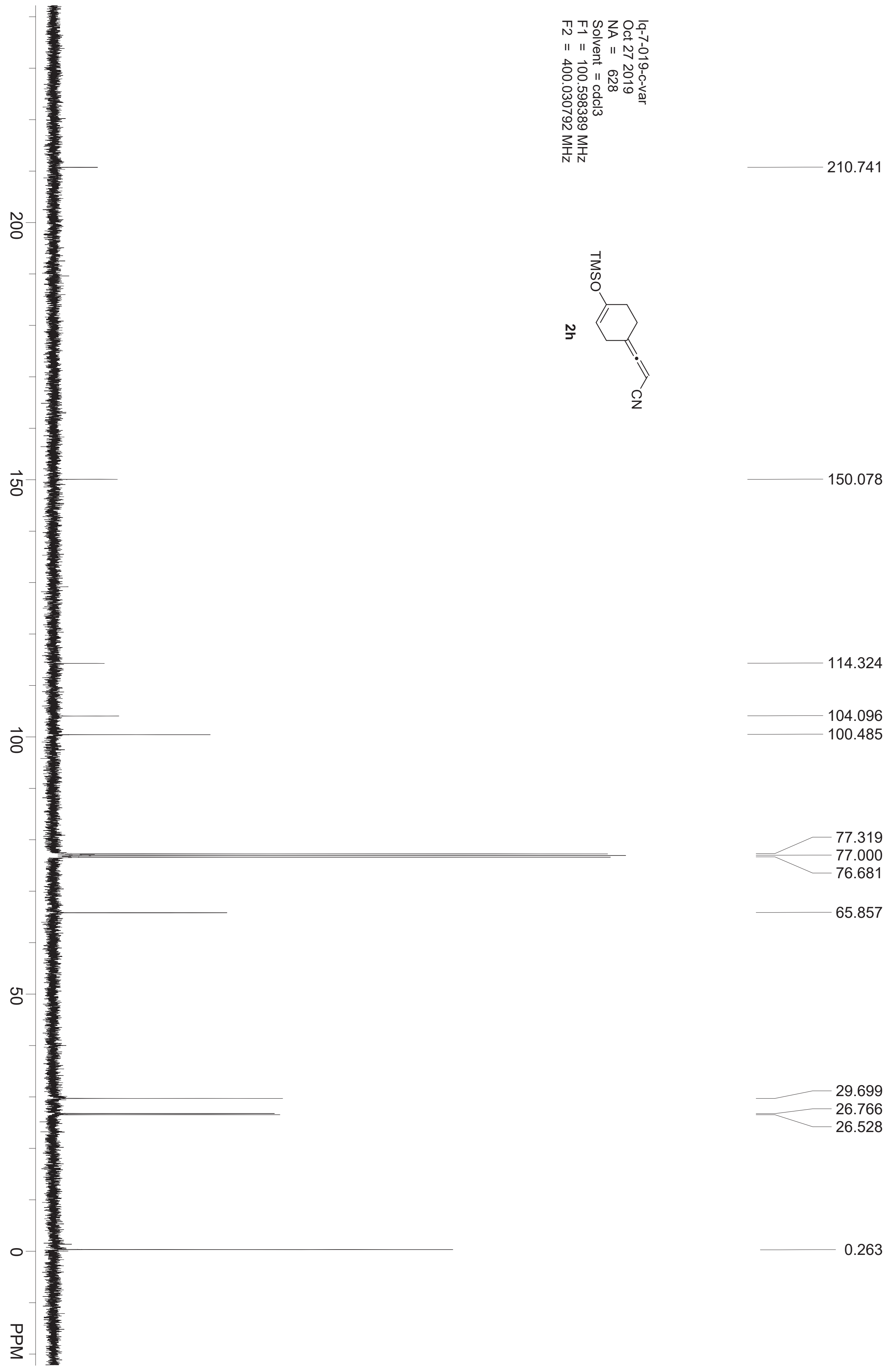

Supplementary Figure 83. ^{13}C NMR (100 MHz, $CDCl_3$) spectrum for **2h**

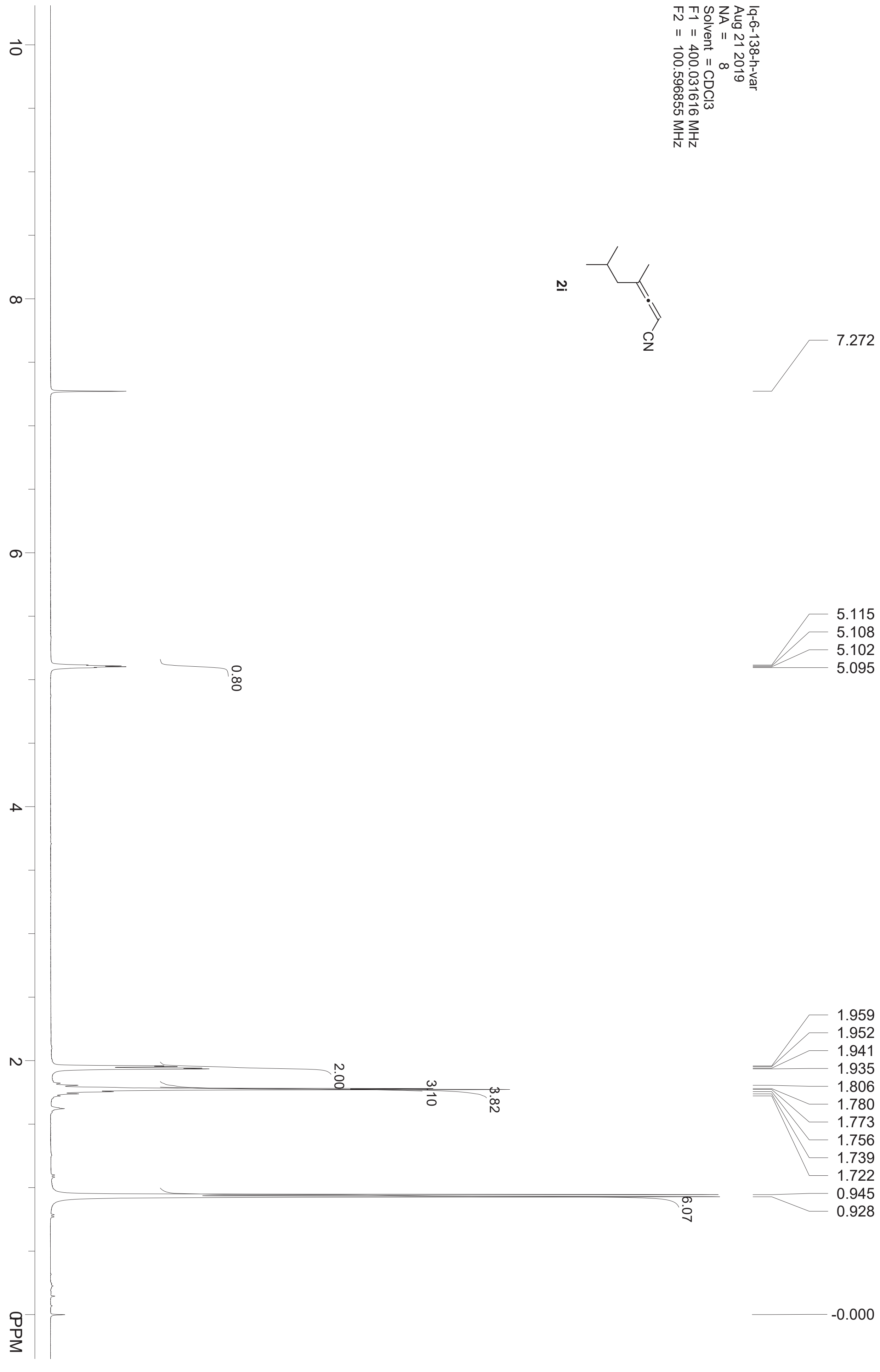

Supplementary Figure 84. ^{1}H NMR (400 MHz, $CDCl_3$) spectrum for **2i**

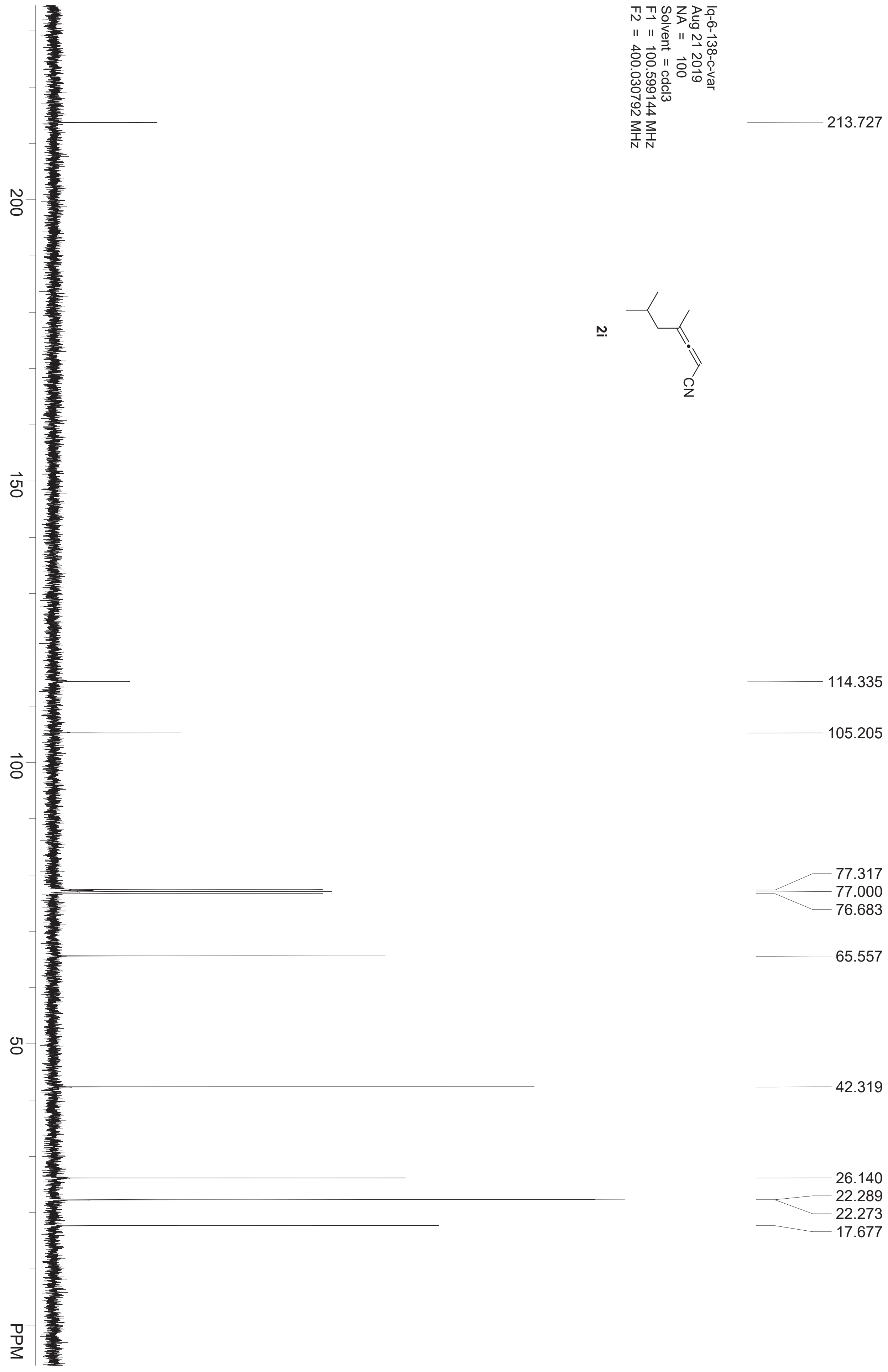

Supplementary Figure 85. ^{13}C NMR (100 MHz, $CDCl_3$) spectrum for **2i**

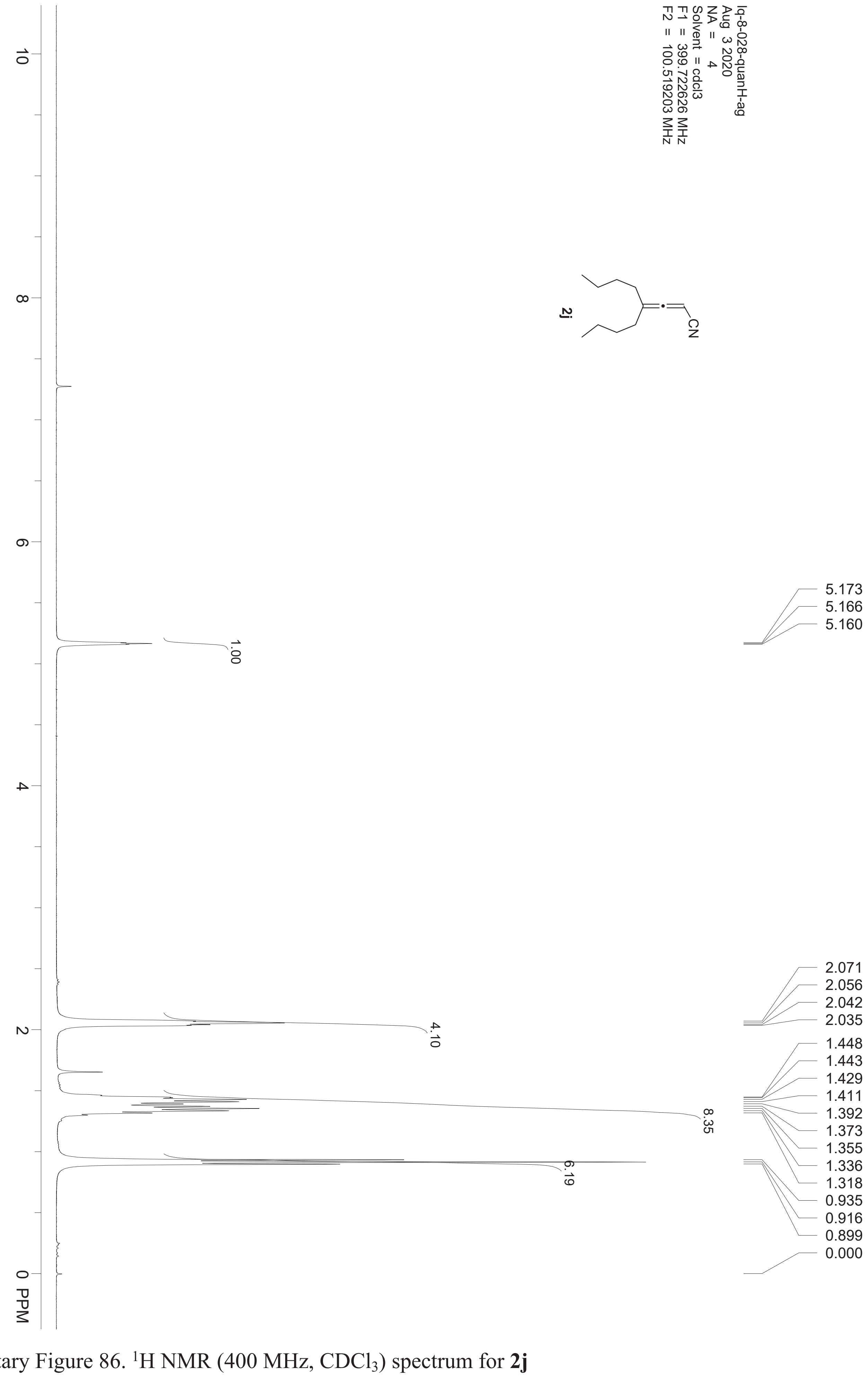

Supplementary Figure 86. ^{1}H NMR (400 MHz, $CDCl_3$) spectrum for **2j**

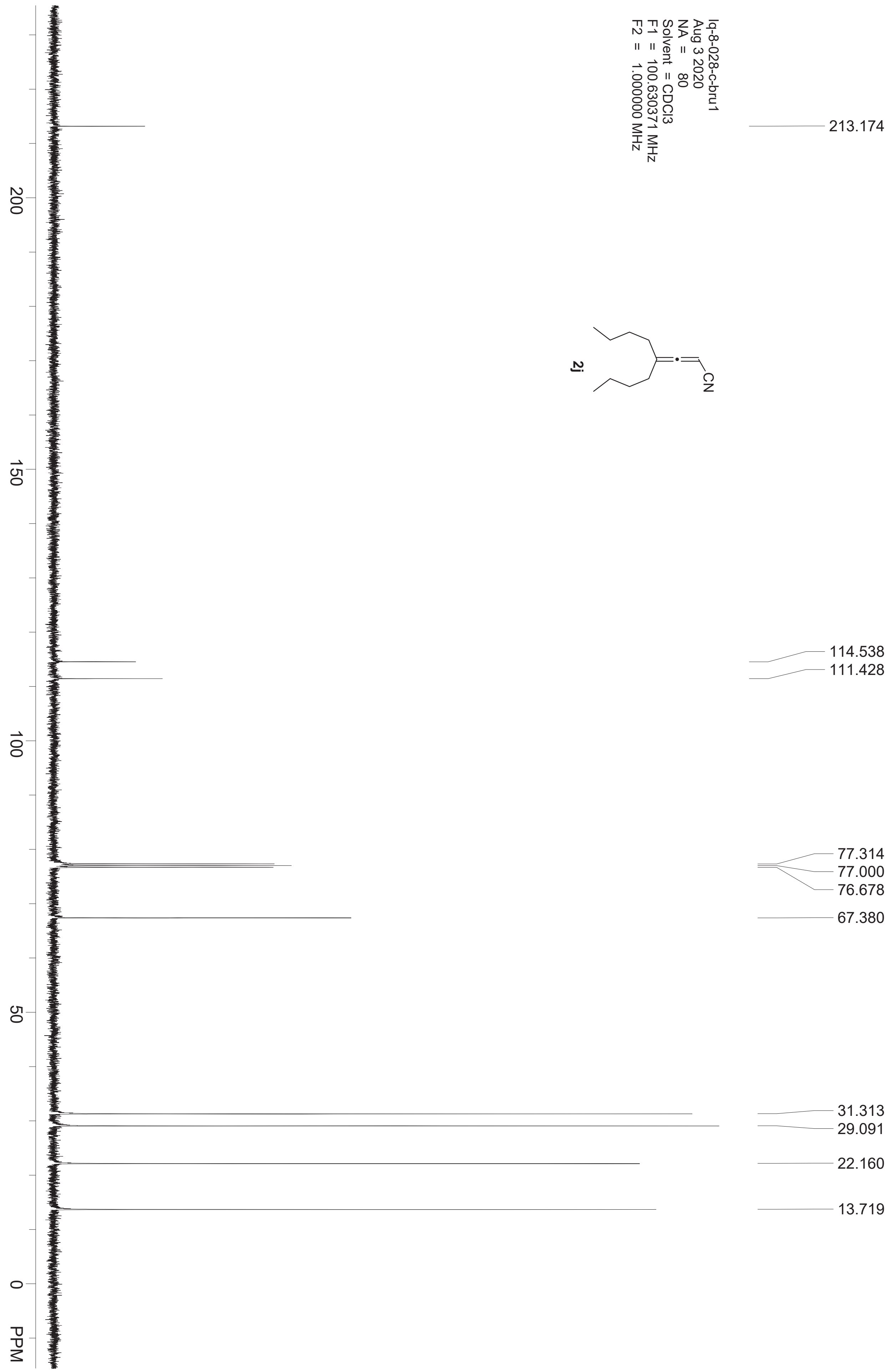

Supplementary Figure 87. ^{13}C NMR (100 MHz, $CDCl_3$) spectrum for **2j**

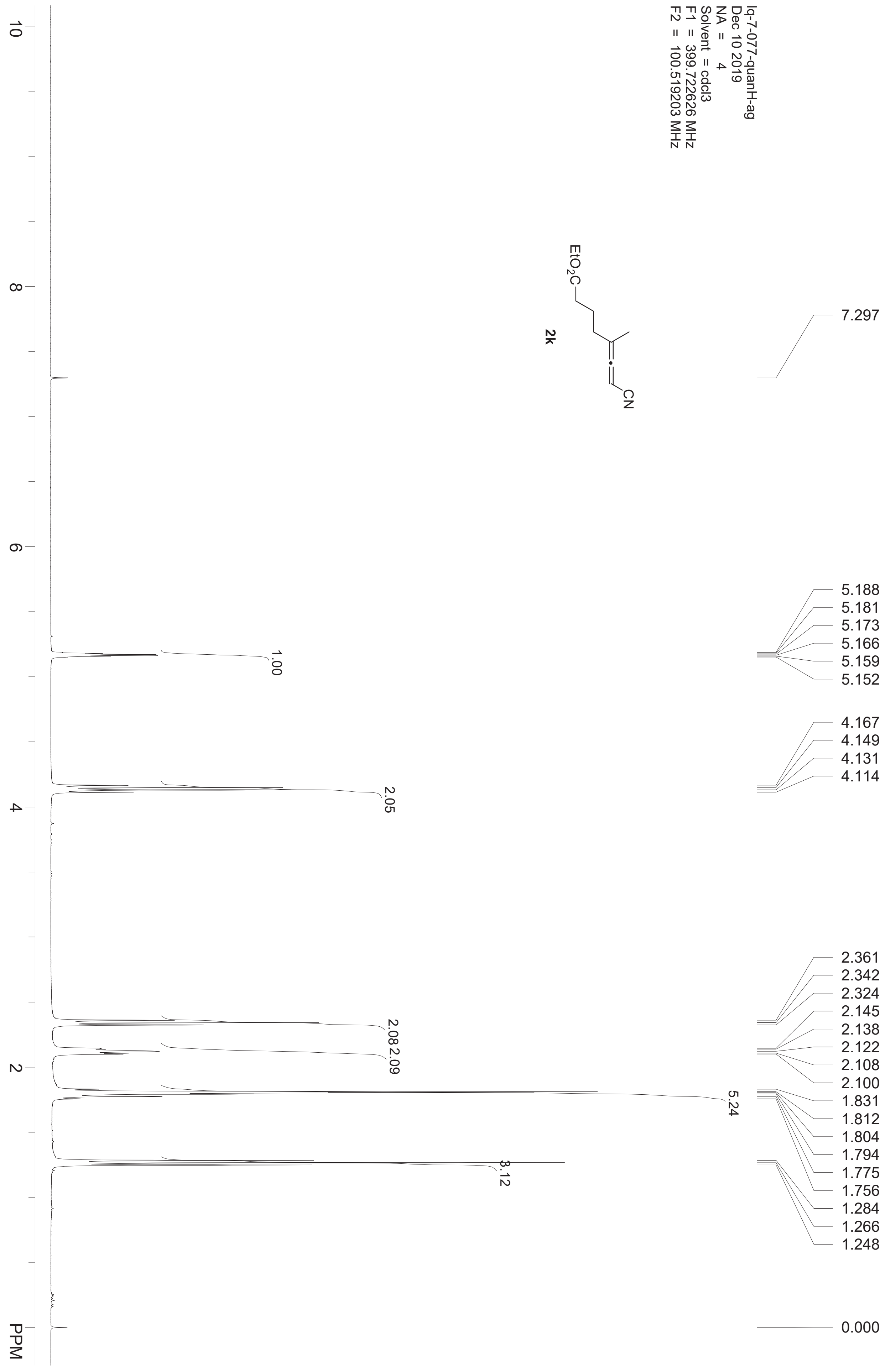

Supplementary Figure 88. ^{1}H NMR (400 MHz, $CDCl_3$) spectrum for **2k**

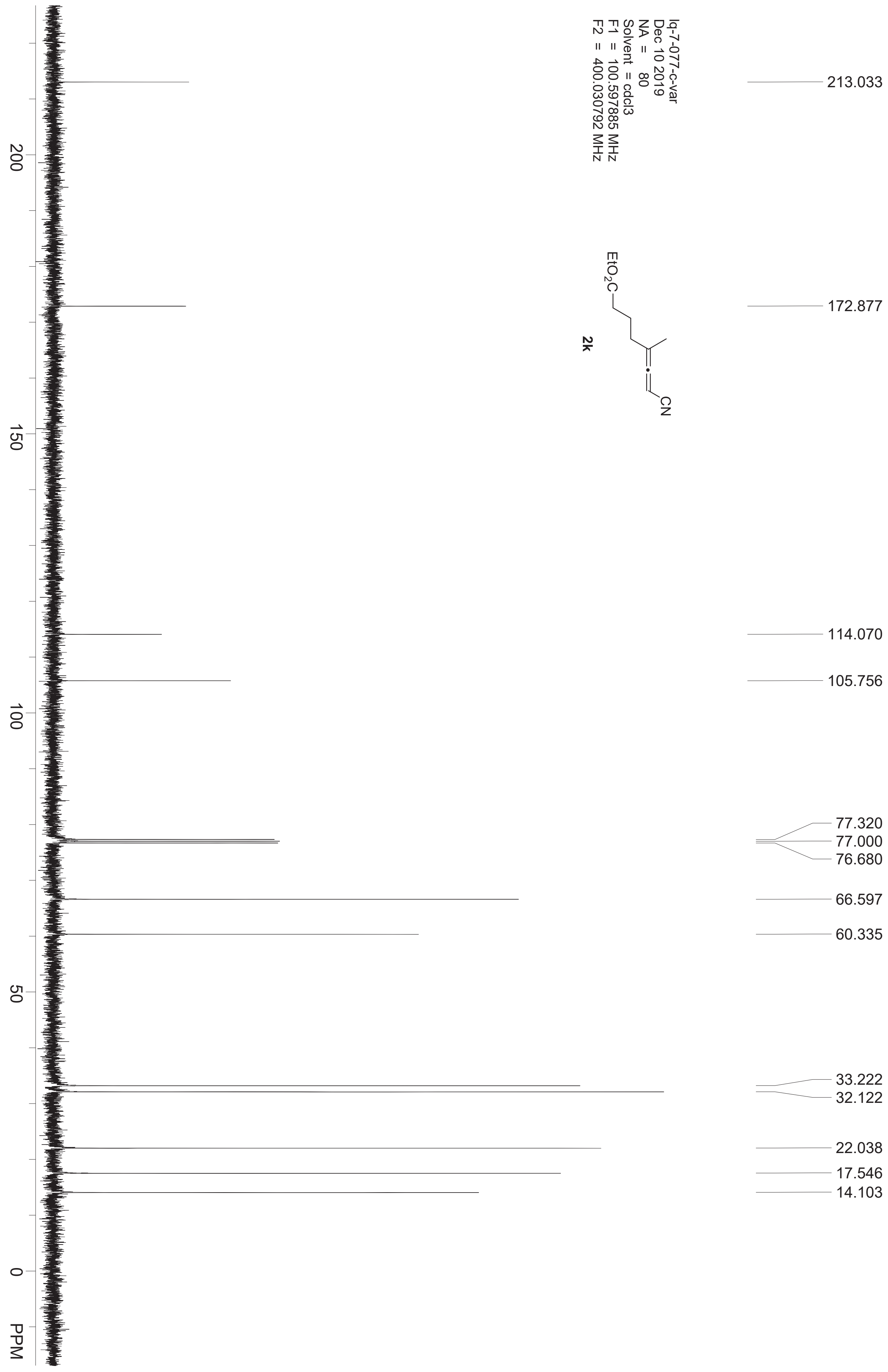

Supplementary Figure 89. ^{13}C NMR (100 MHz, $CDCl_3$) spectrum for **2k**

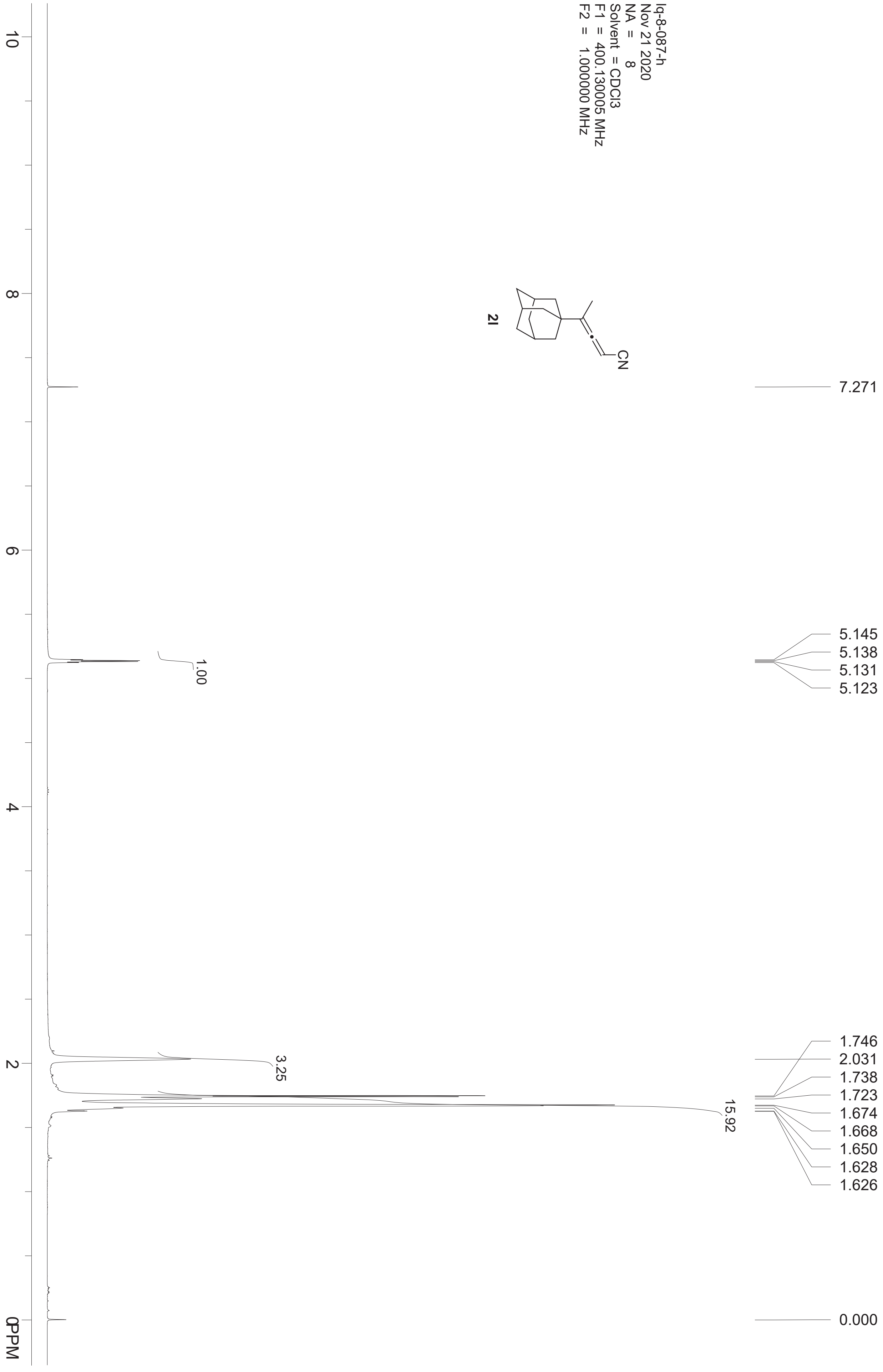

Supplementary Figure 90. ^{1}H NMR (400 MHz, $CDCl_3$) spectrum for **2l**

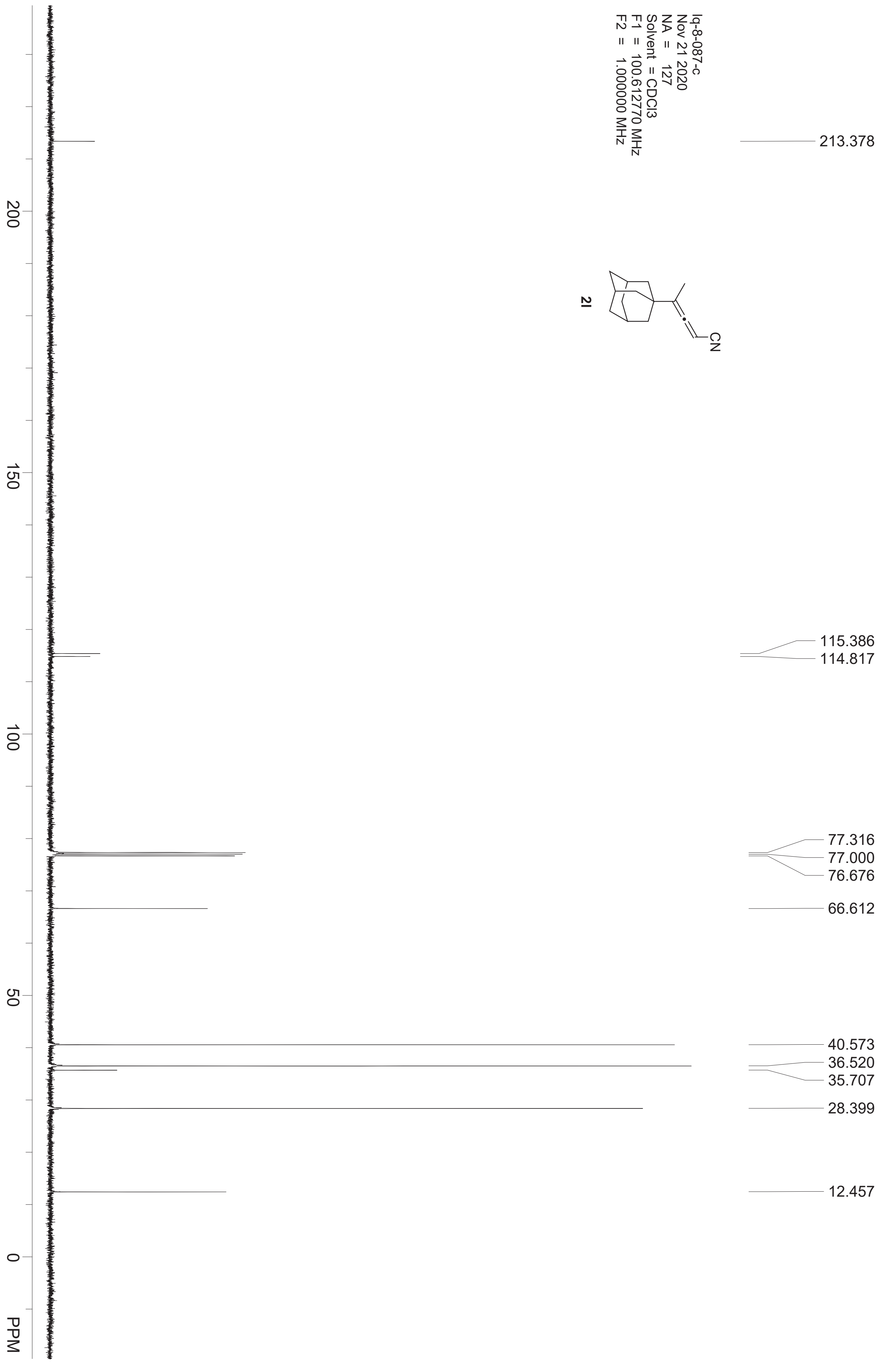

Supplementary Figure 91. ^{13}C NMR (100 MHz, $CDCl_3$) spectrum for **2l**

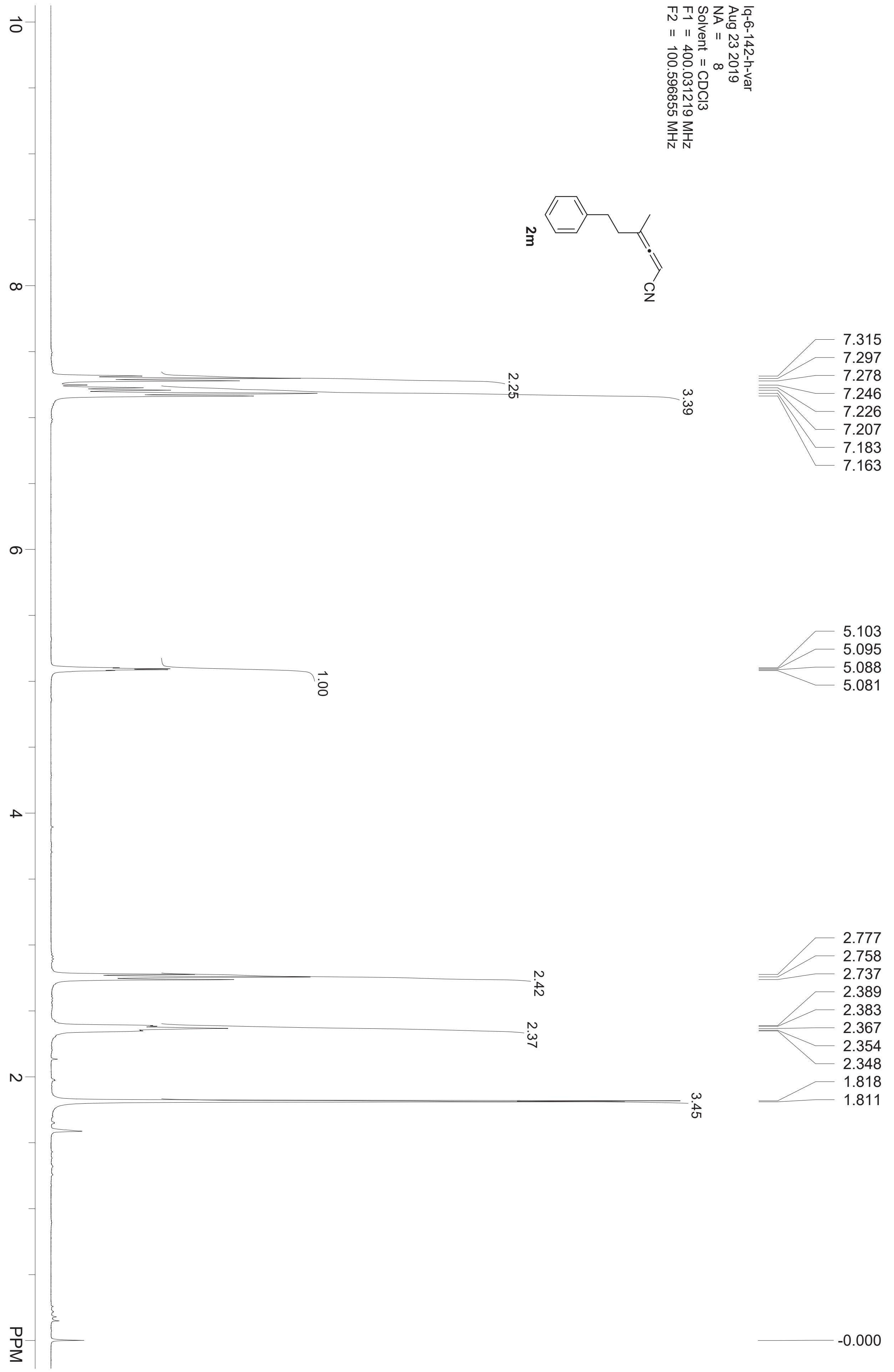

Supplementary Figure 92. ^{1}H NMR (400 MHz, $CDCl_3$) spectrum for **2m**

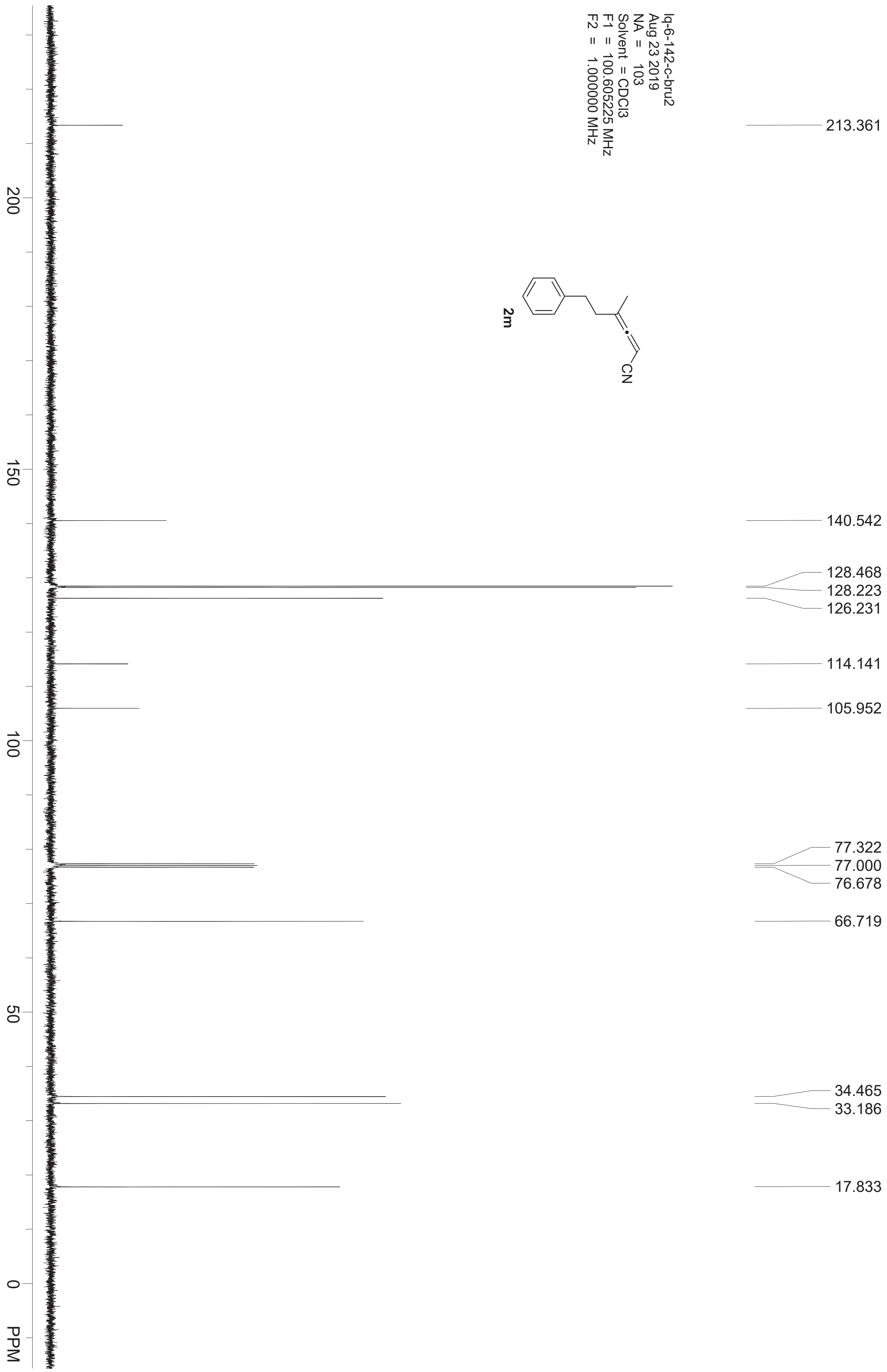

Supplementary Figure 93. ^{13}C NMR (100 MHz, $CDCl_3$) spectrum for **2m**

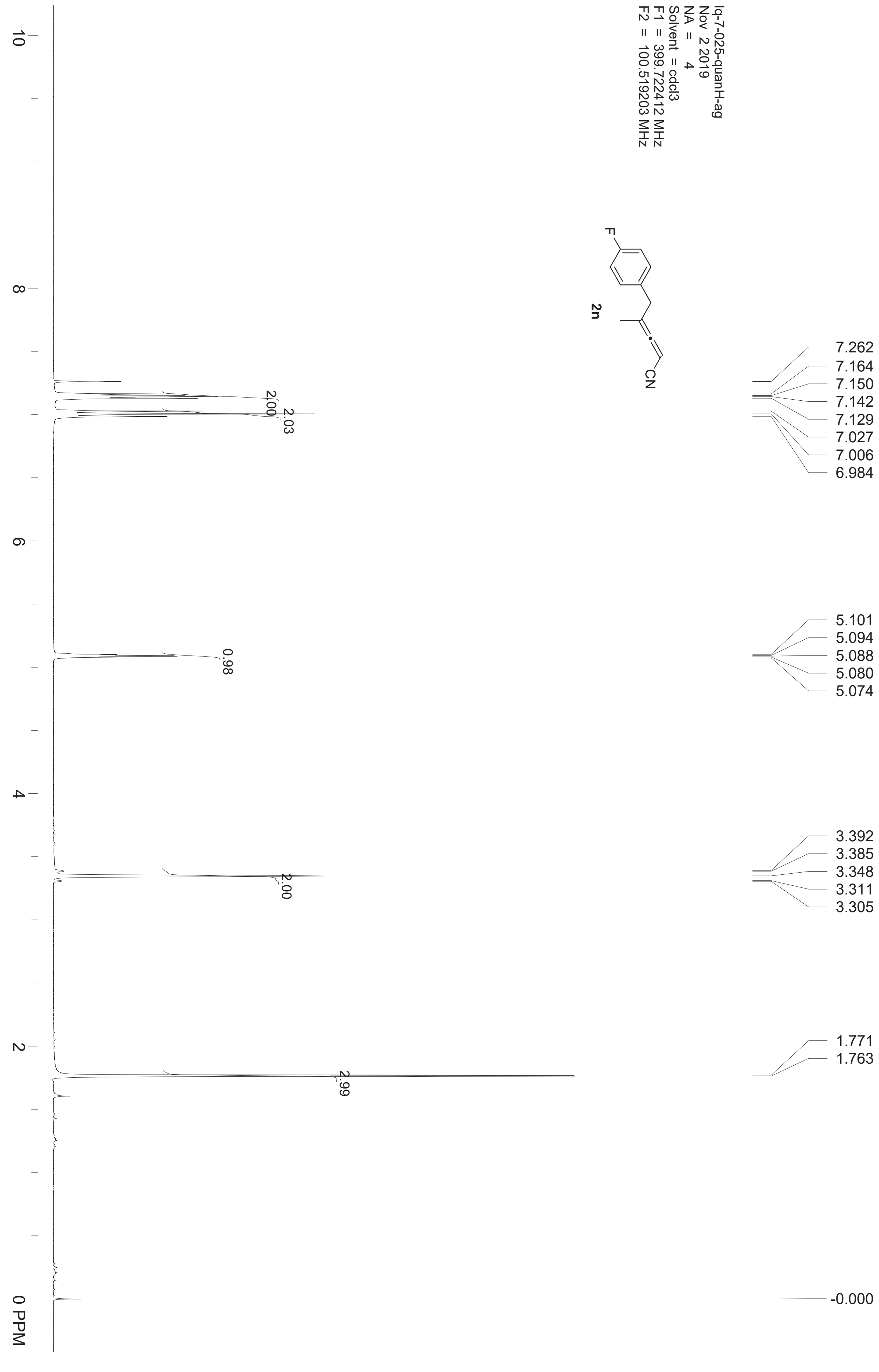

Supplementary Figure 94. ^{1}H NMR (400 MHz, $CDCl_3$) spectrum for **2n**

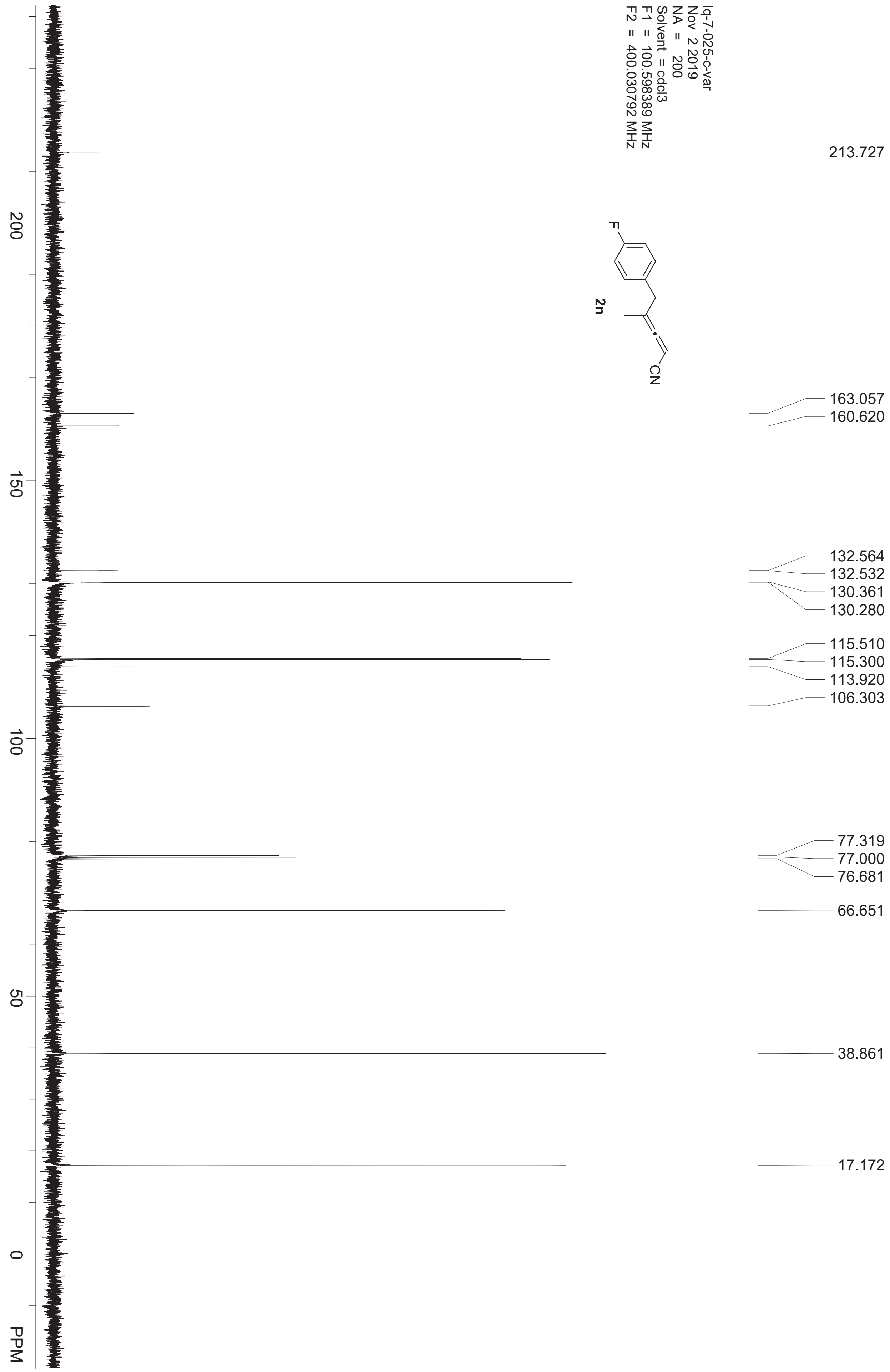

Supplementary Figure 95. ^{13}C NMR (100 MHz, CDCl$_3$) spectrum for **2n**

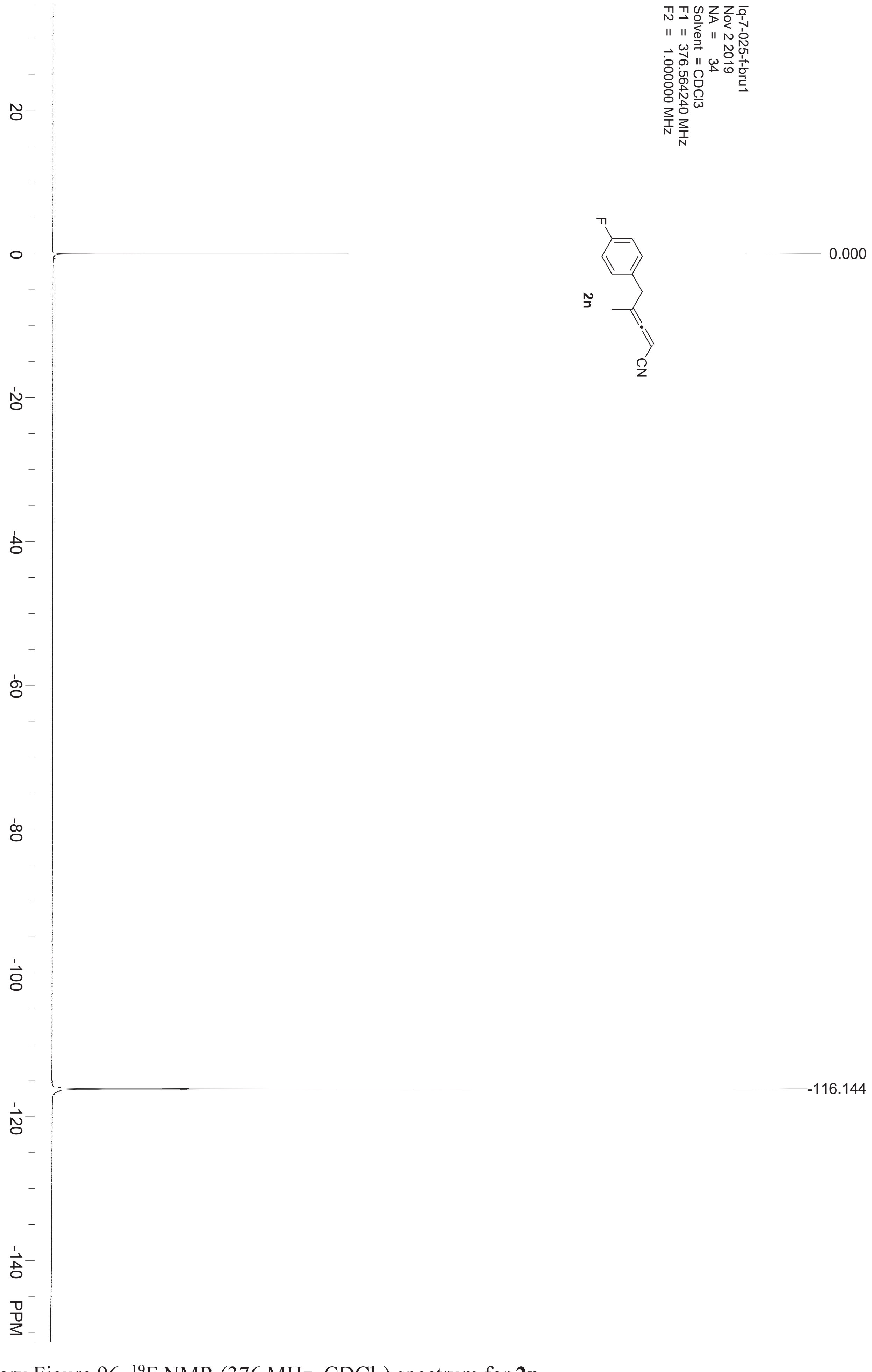

Supplementary Figure 96. ^{19}F NMR (376 MHz, $CDCl_3$) spectrum for **2n**

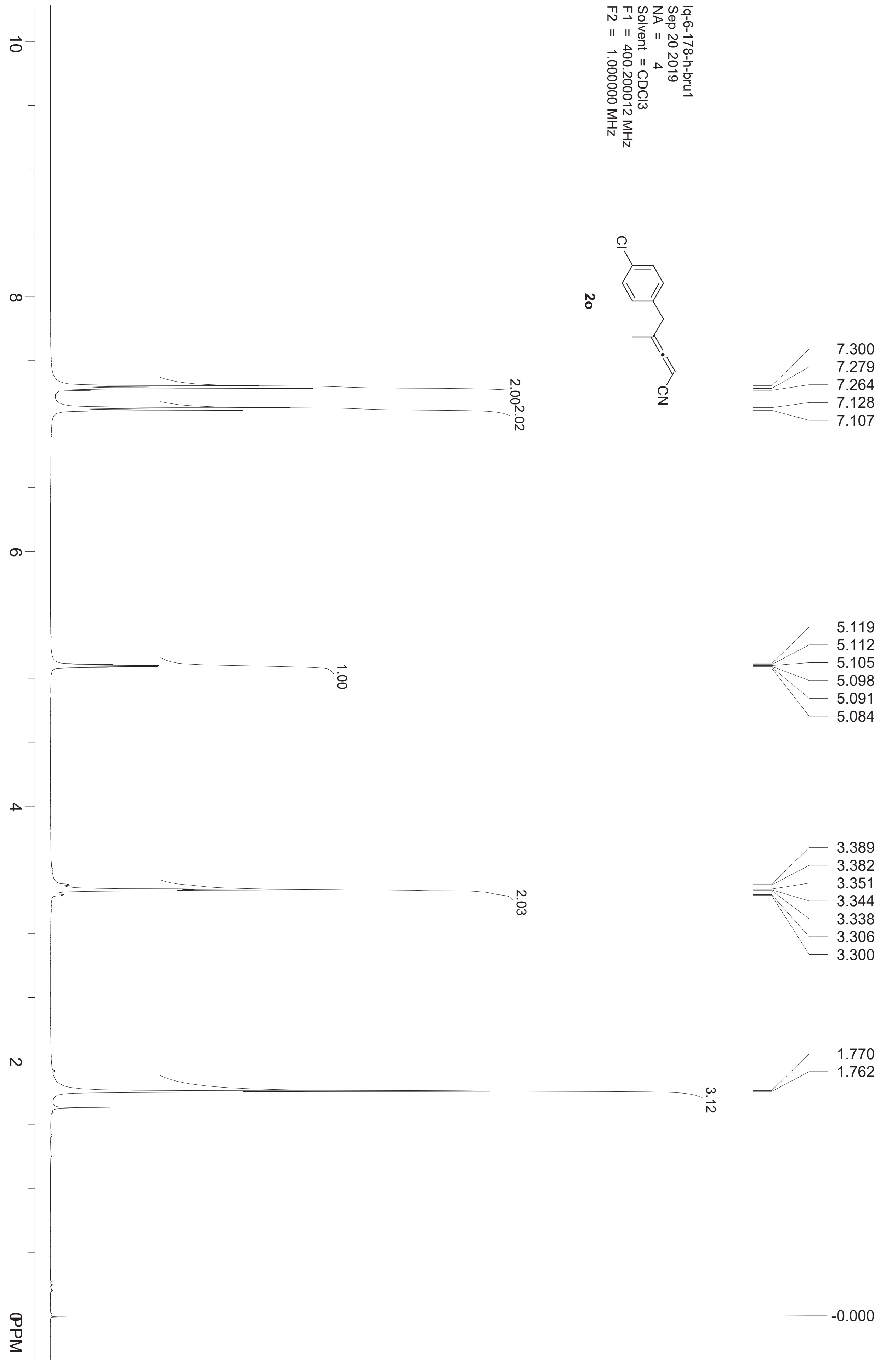

Supplementary Figure 97. ^{1}H NMR (400 MHz, $CDCl_3$) spectrum for **2o**

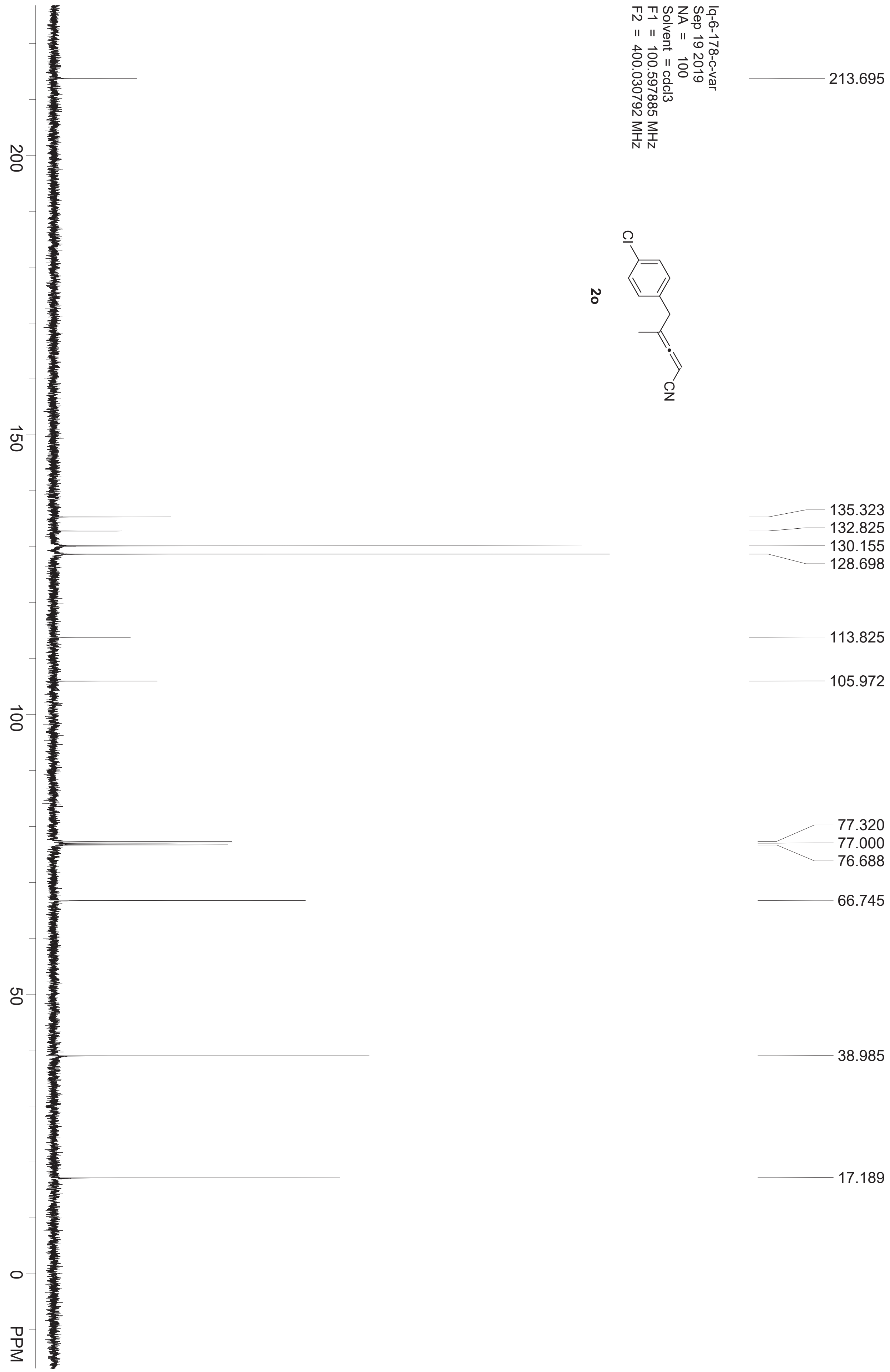

Supplementary Figure 98. ^{13}C NMR (100 MHz, $CDCl_3$) spectrum for **2o**

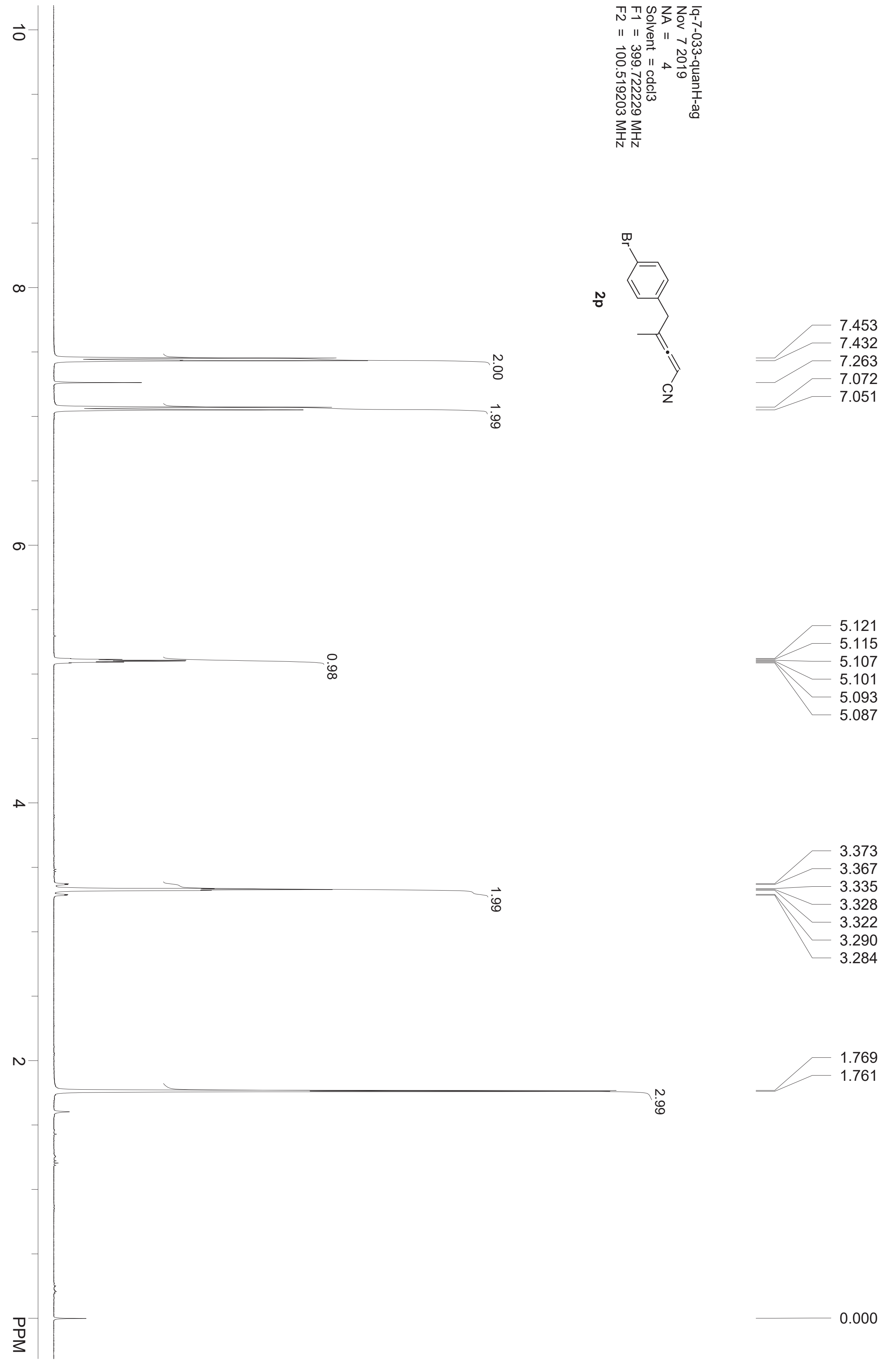

Supplementary Figure 99. ^{1}H NMR (400 MHz, $CDCl_3$) spectrum for **2p**

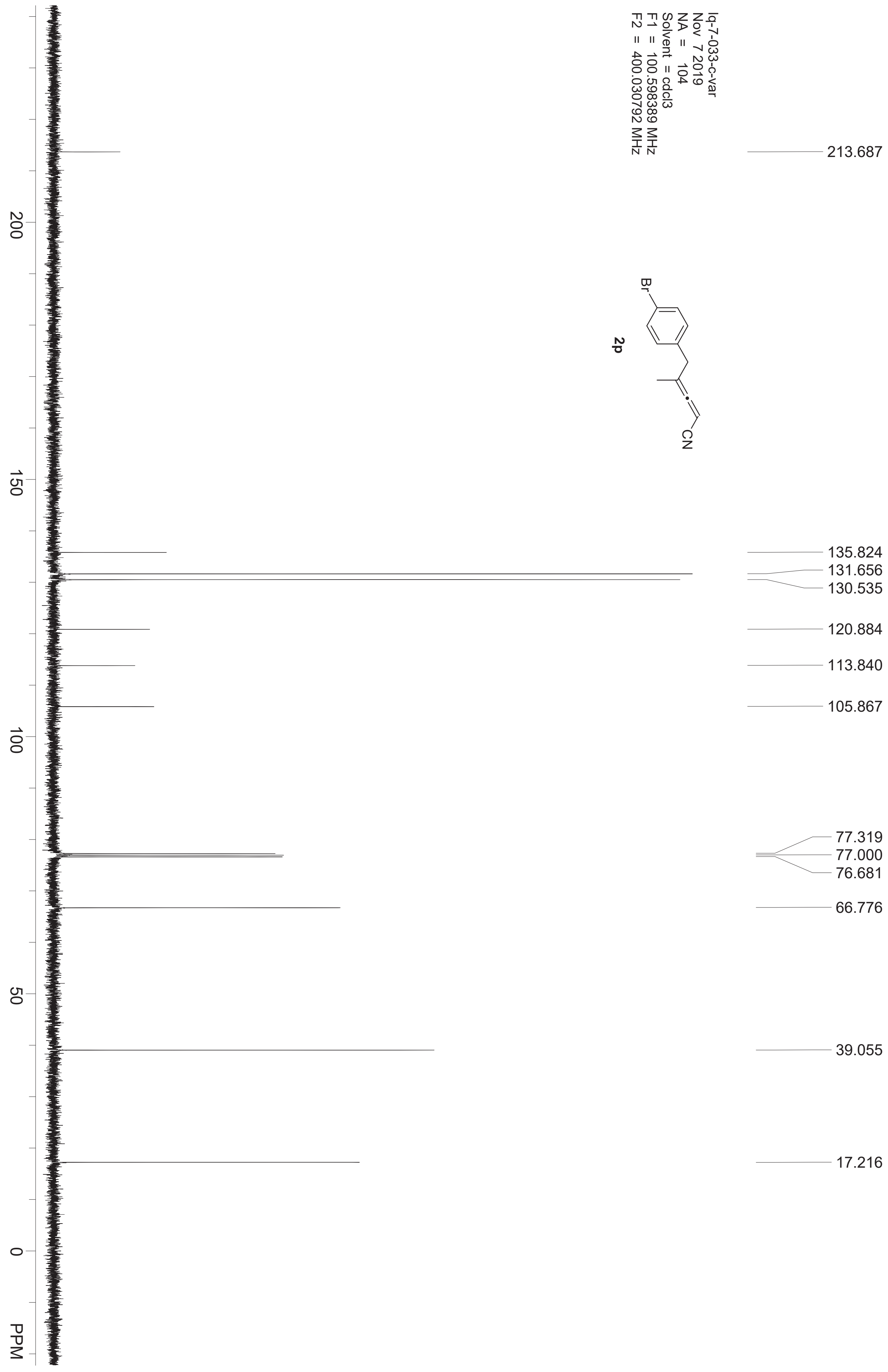

Supplementary Figure 100. ^{13}C NMR (100 MHz, $CDCl_3$) spectrum for **2p**

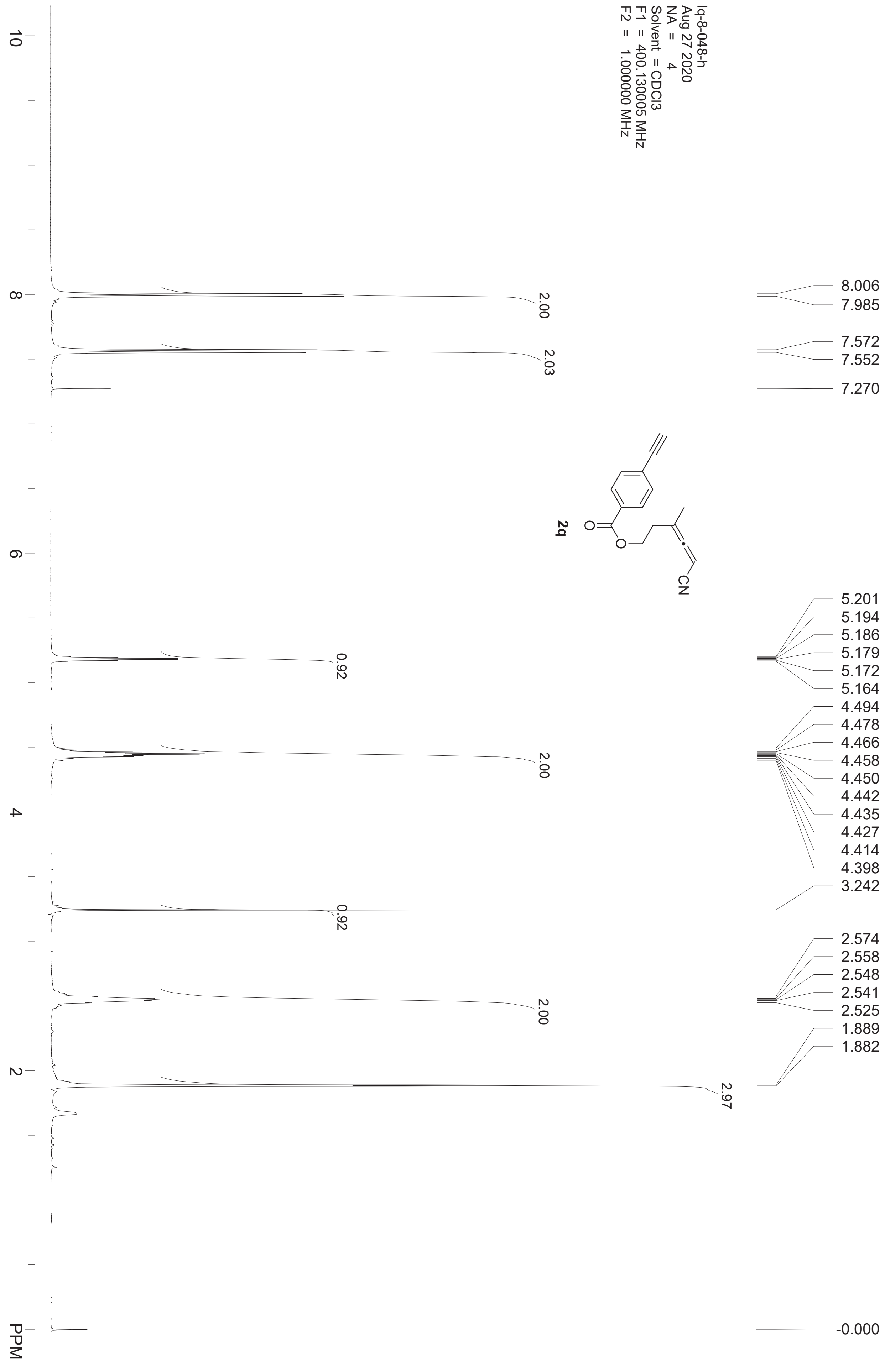

Supplementary Figure 101. ^{1}H NMR (400 MHz, $CDCl_3$) spectrum for **2q**

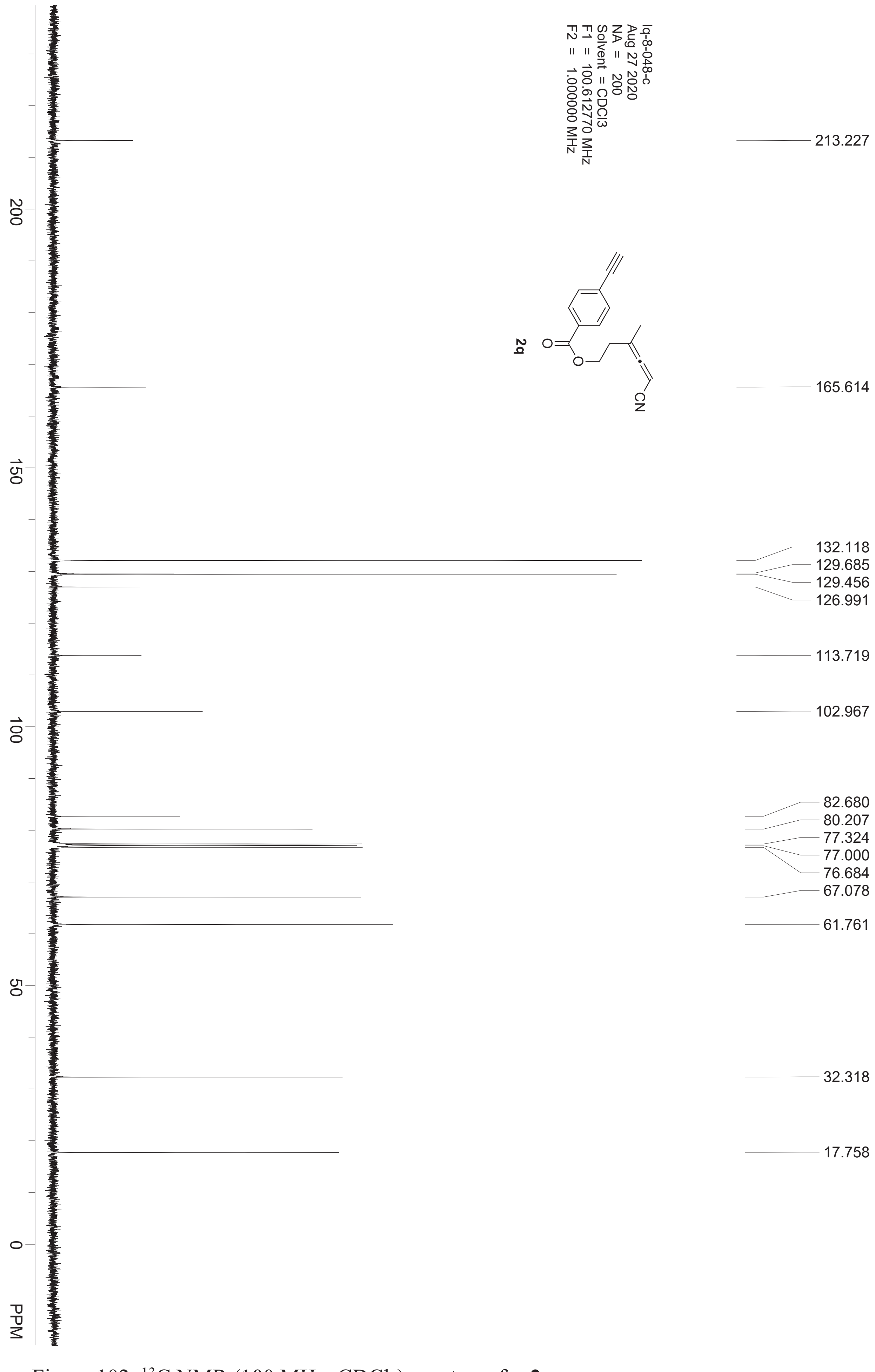

Supplementary Figure 102. ^{13}C NMR (100 MHz, $CDCl_3$) spectrum for **2q**

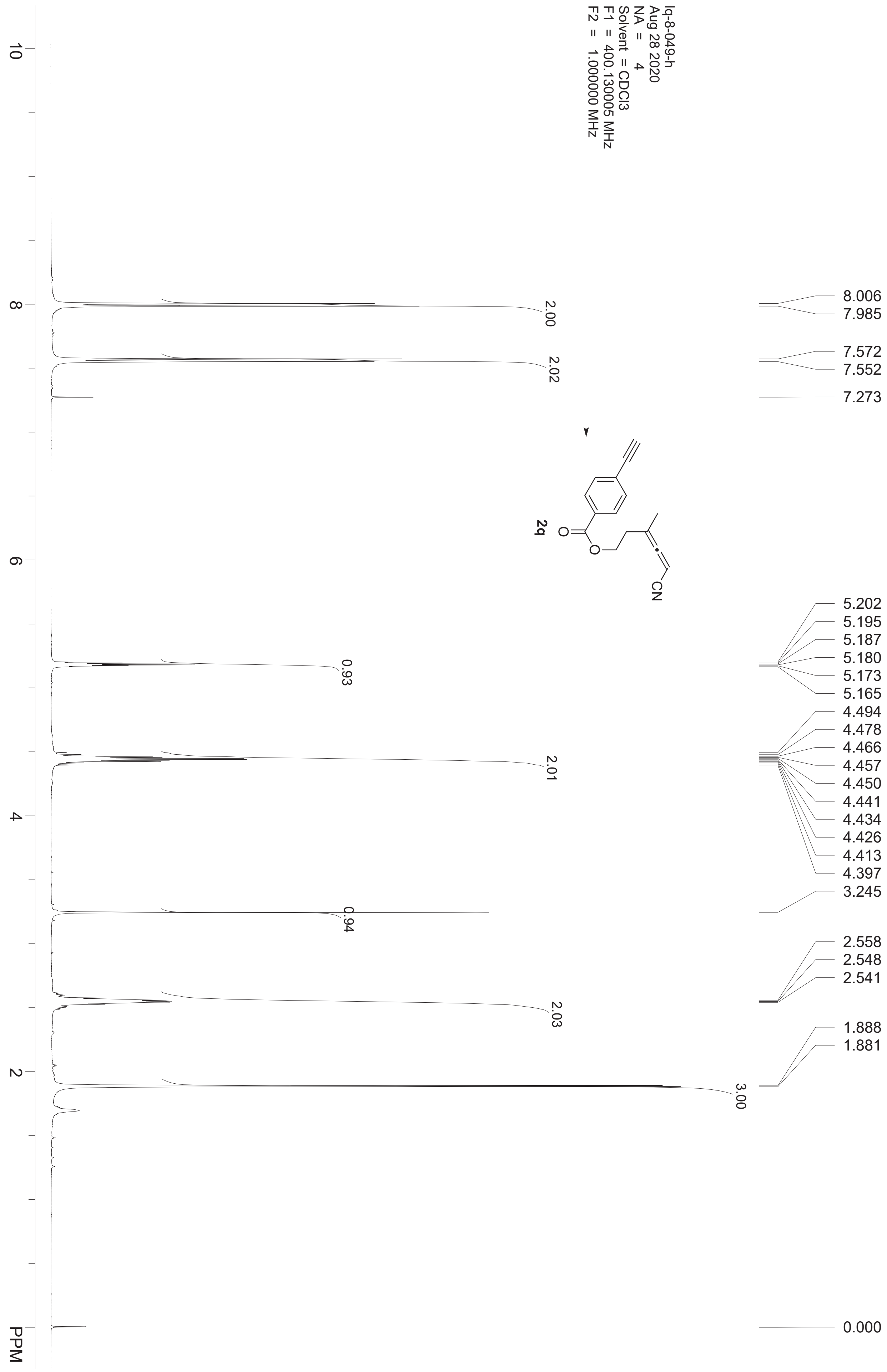

Supplementary Figure 103. ^{1}H NMR (400 MHz, $CDCl_3$) spectrum for **2q**

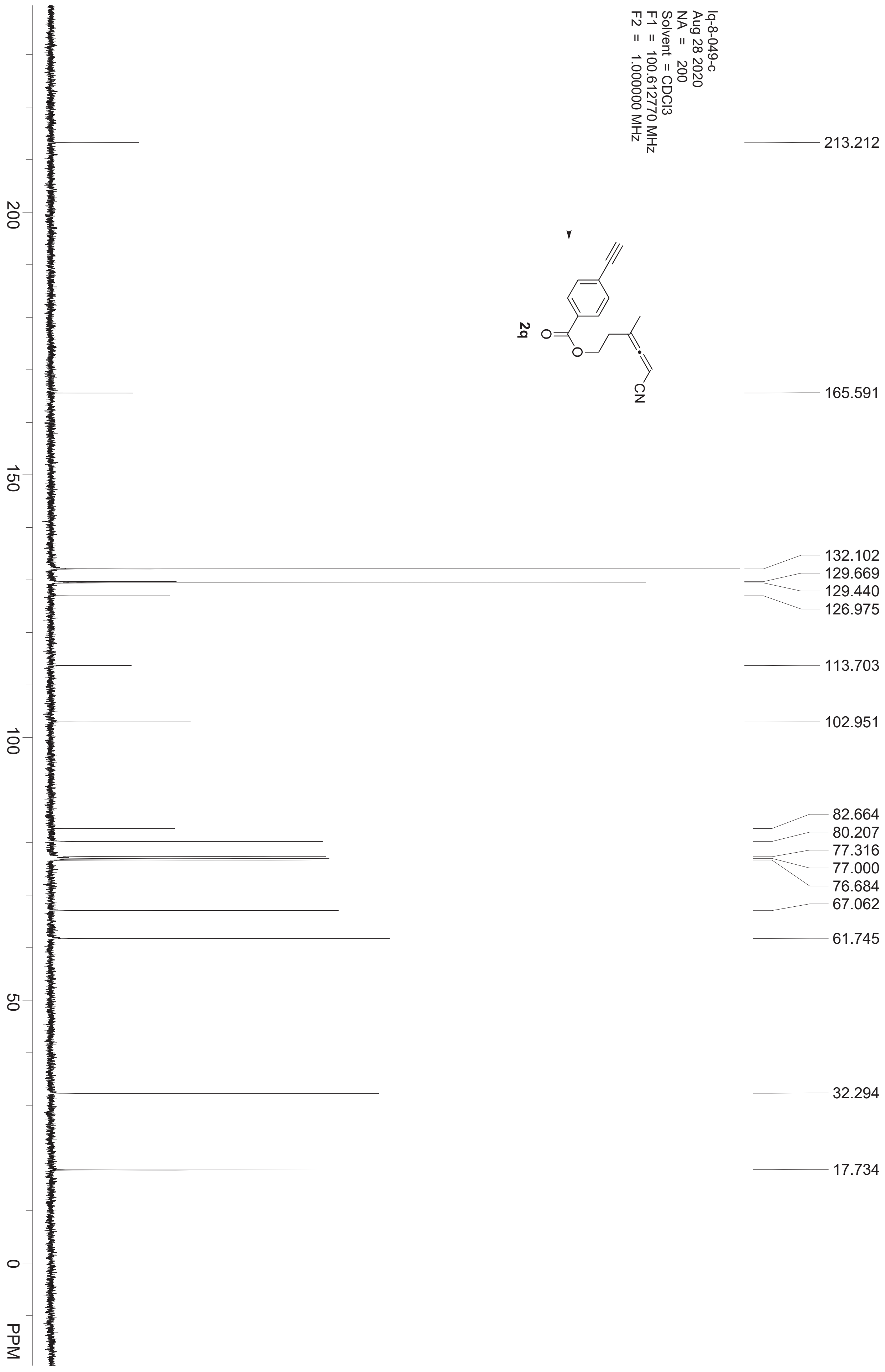

Supplementary Figure 104. ^{13}C NMR (100 MHz, $CDCl_3$) spectrum for **2q**

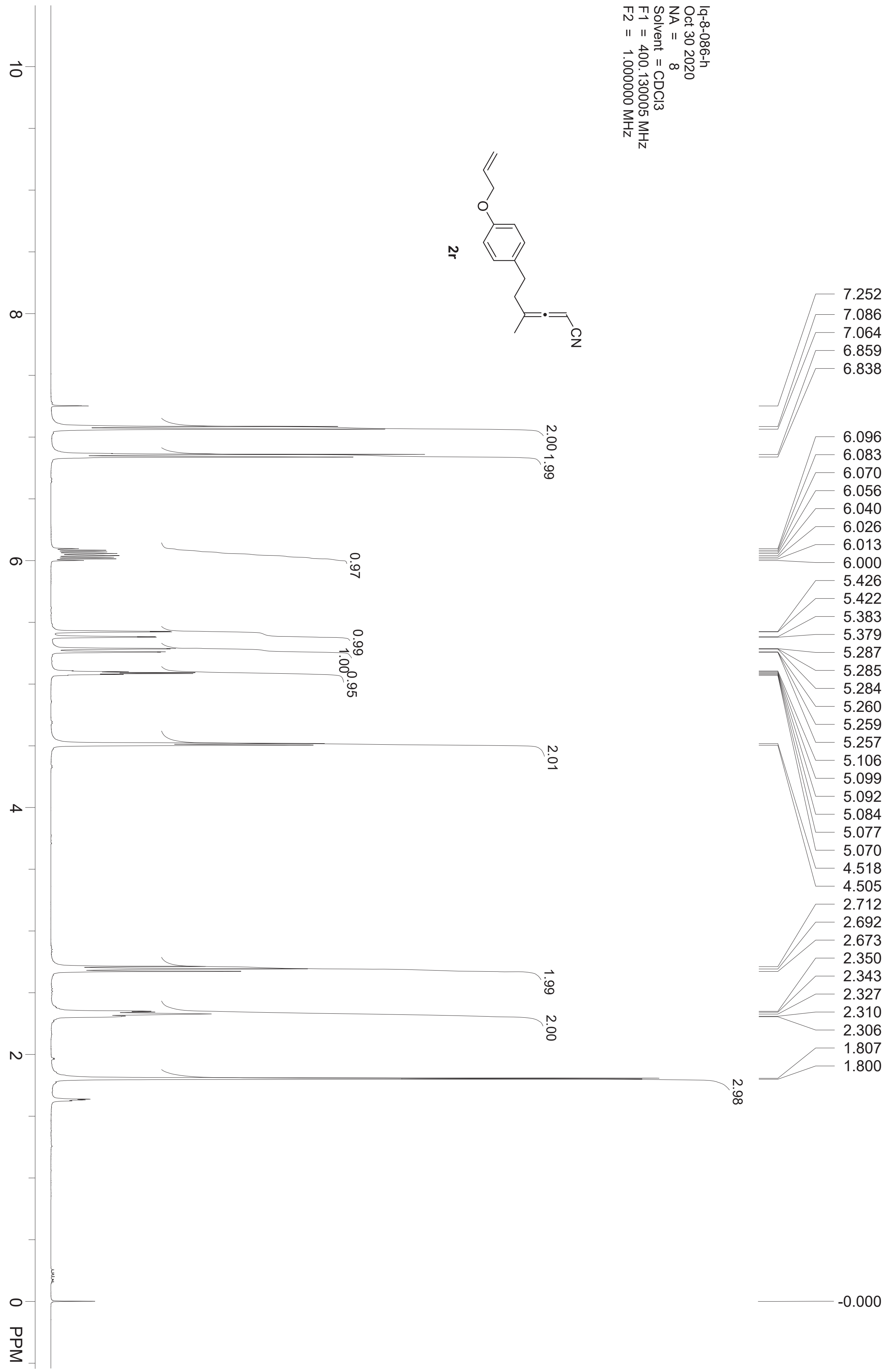

Supplementary Figure 105. ^{1}H NMR (400 MHz, $CDCl_3$) spectrum for **2r**

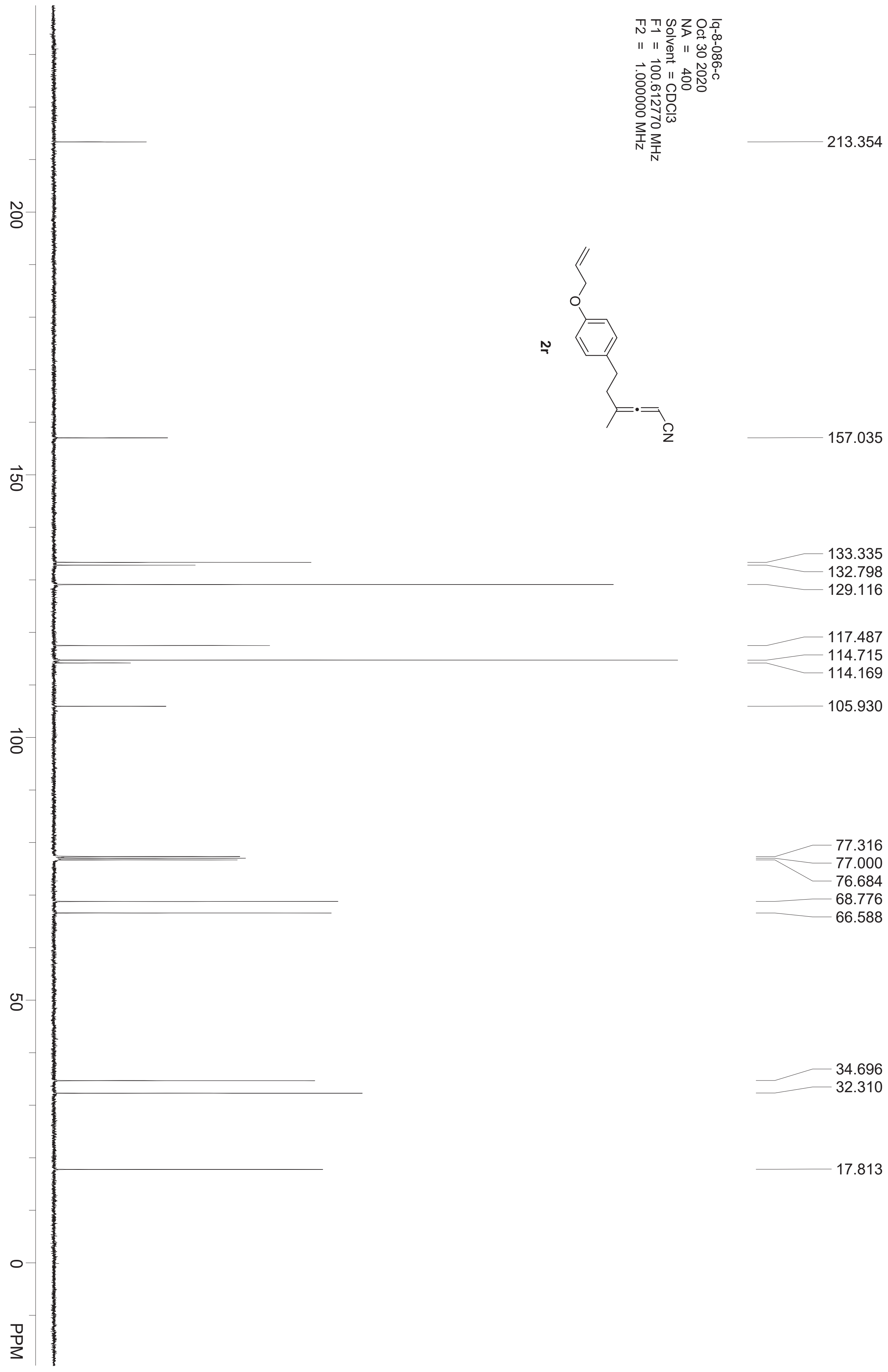

Supplementary Figure 106. ^{13}C NMR (100 MHz, $CDCl_3$) spectrum for **2r**

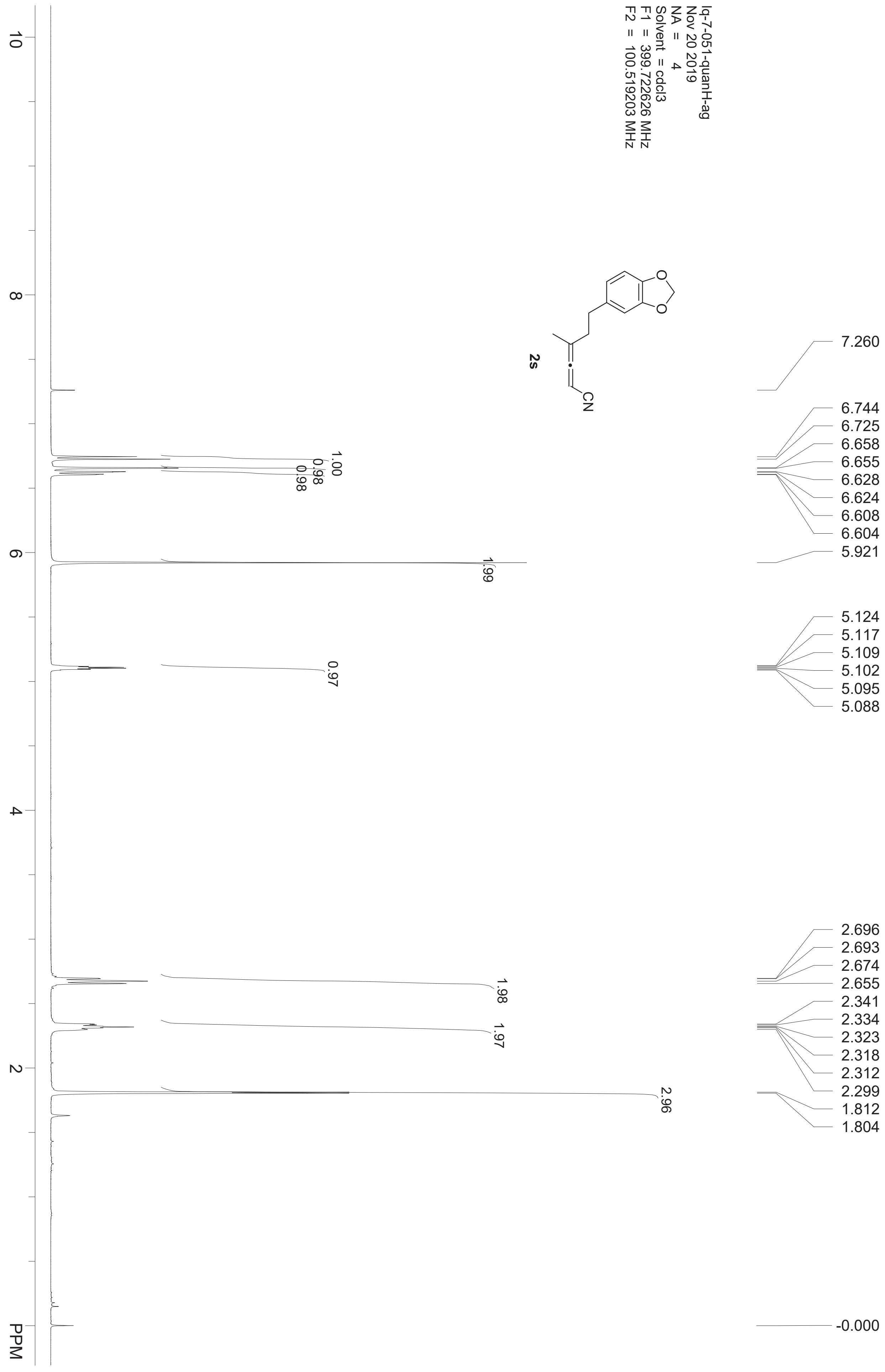

Supplementary Figure 107. ^{1}H NMR (400 MHz, $CDCl_3$) spectrum for **2s**

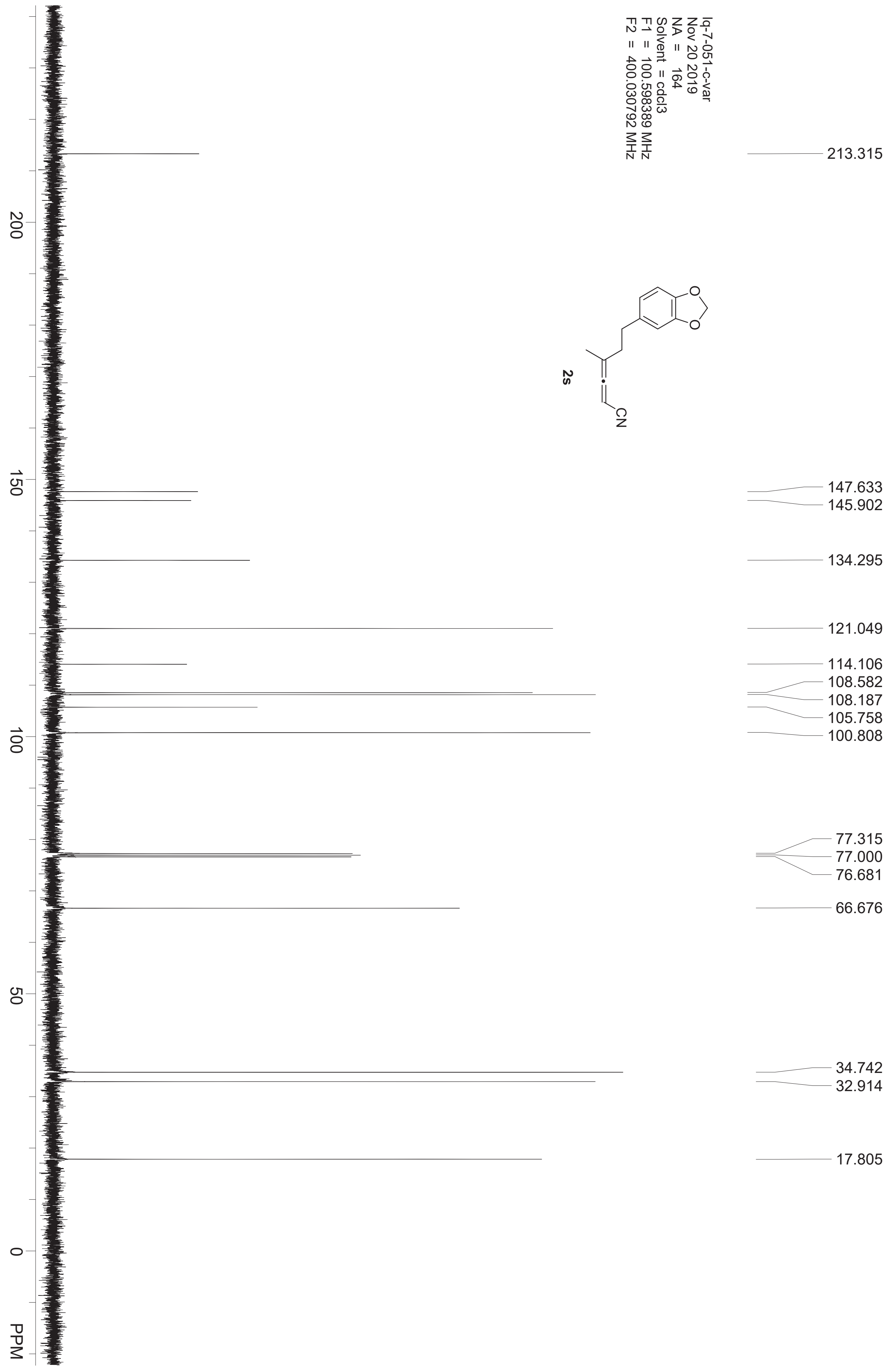

Supplementary Figure 108. ^{13}C NMR (100 MHz, $CDCl_3$) spectrum for **2s**

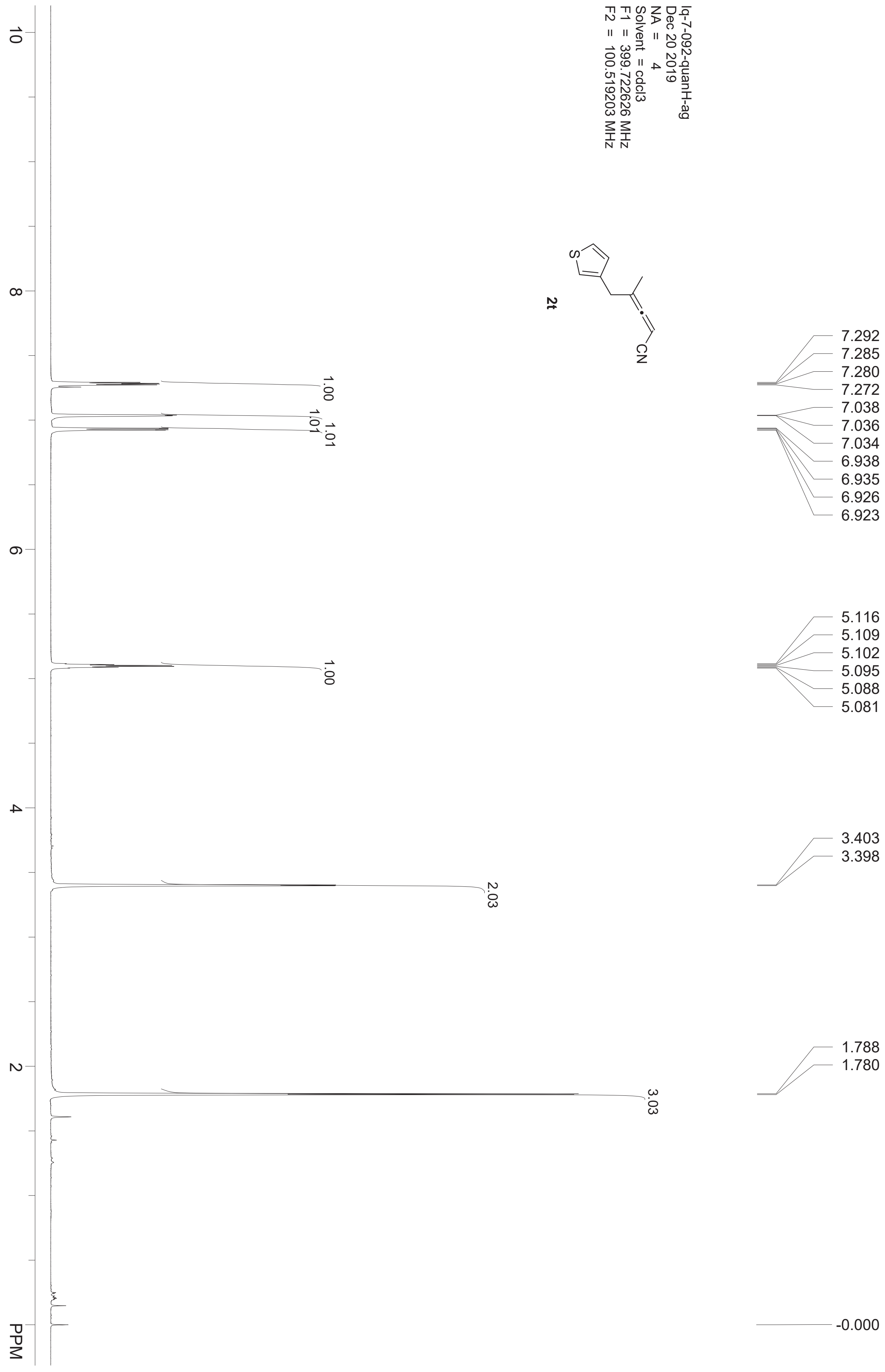

Supplementary Figure 109. ^{1}H NMR (400 MHz, $CDCl_3$) spectrum for **2t**

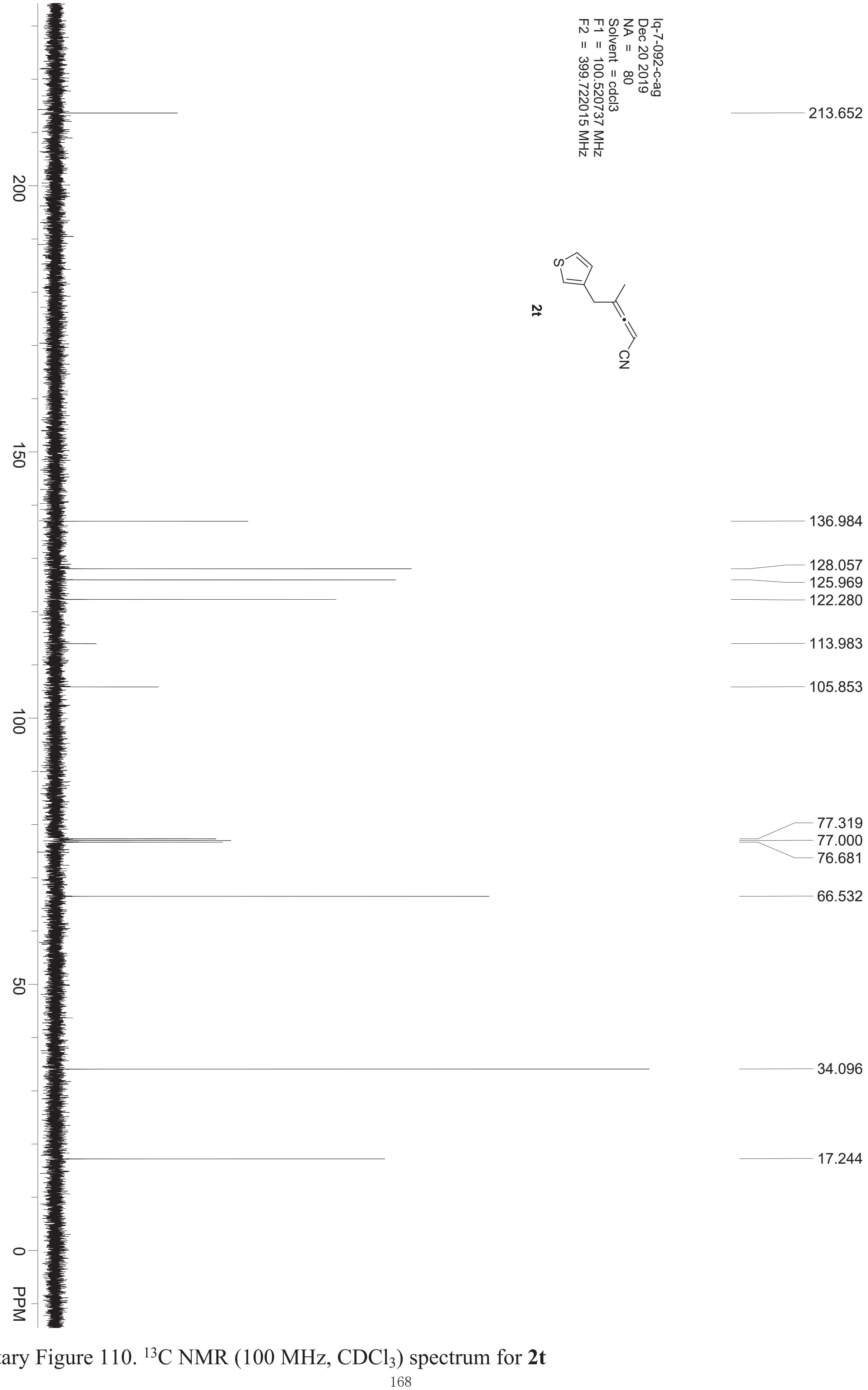

Supplementary Figure 110. ^{13}C NMR (100 MHz, $CDCl_3$) spectrum for **2t**

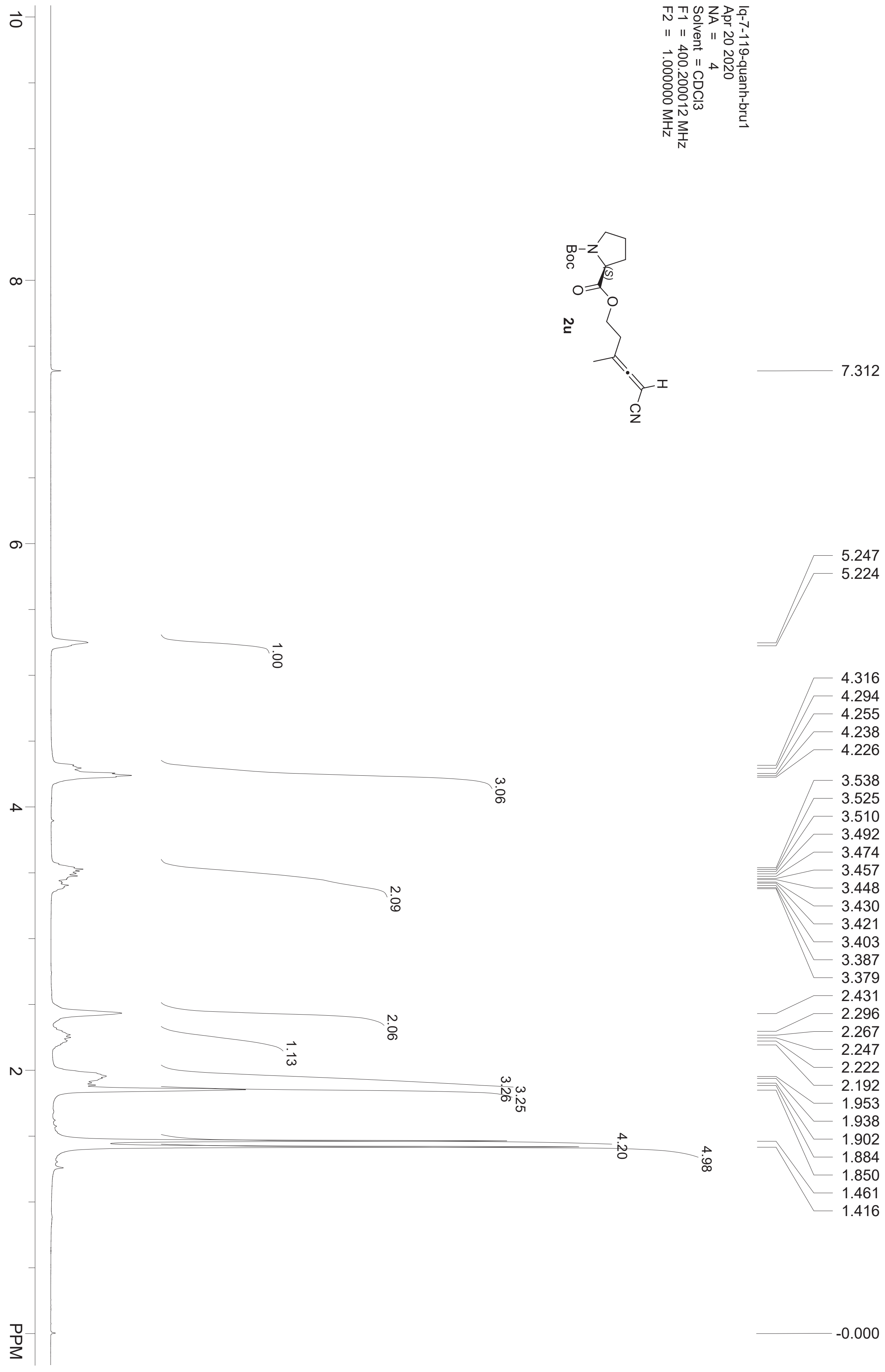

Supplementary Figure 111. ^{1}H NMR (400 MHz, $CDCl_3$) spectrum for **2u**

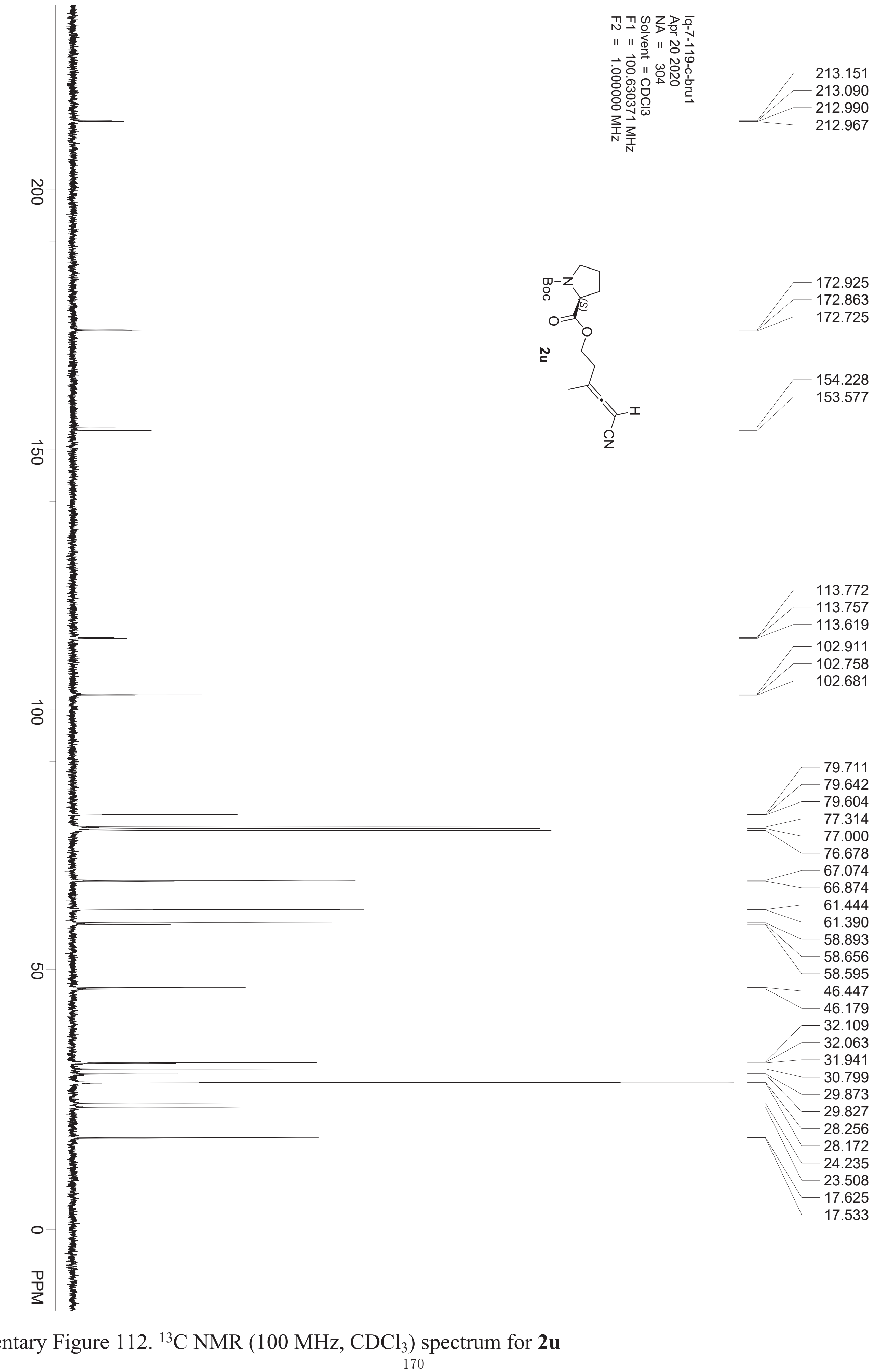

Supplementary Figure 112. ^{13}C NMR (100 MHz, $CDCl_3$) spectrum for **2u**

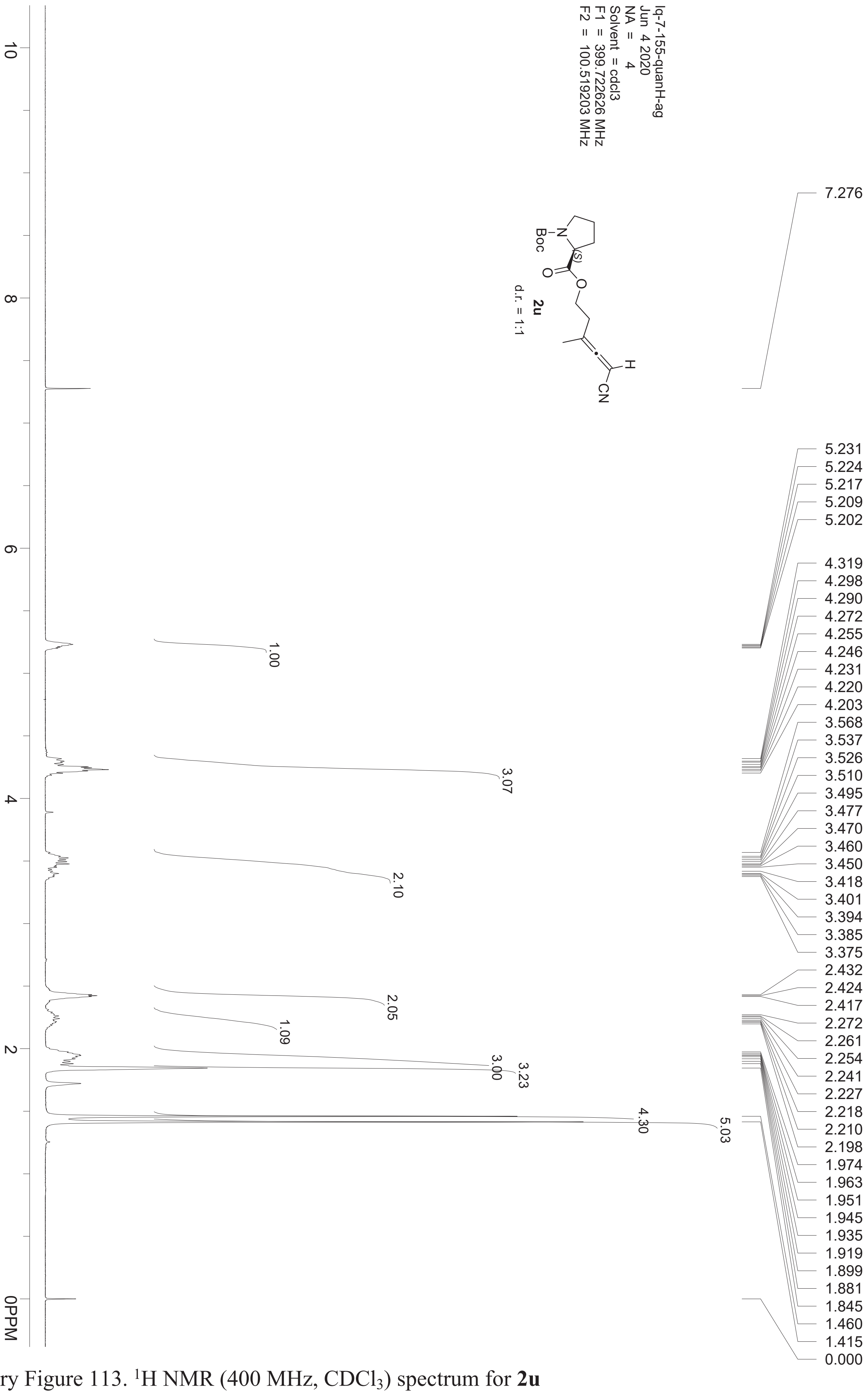

Supplementary Figure 113. ^{1}H NMR (400 MHz, $CDCl_3$) spectrum for **2u**

Area Percent Report

Agilent Technologies

sample LQ-7-155-AD-H-90-10-1.0-214

Data file: C:\Users\Public\Documents\ChemStation\1\Data\YuanYuan 2021-01-27 15-52-31\045-P2-F5-lq-7-155.D

Acquisition Data:

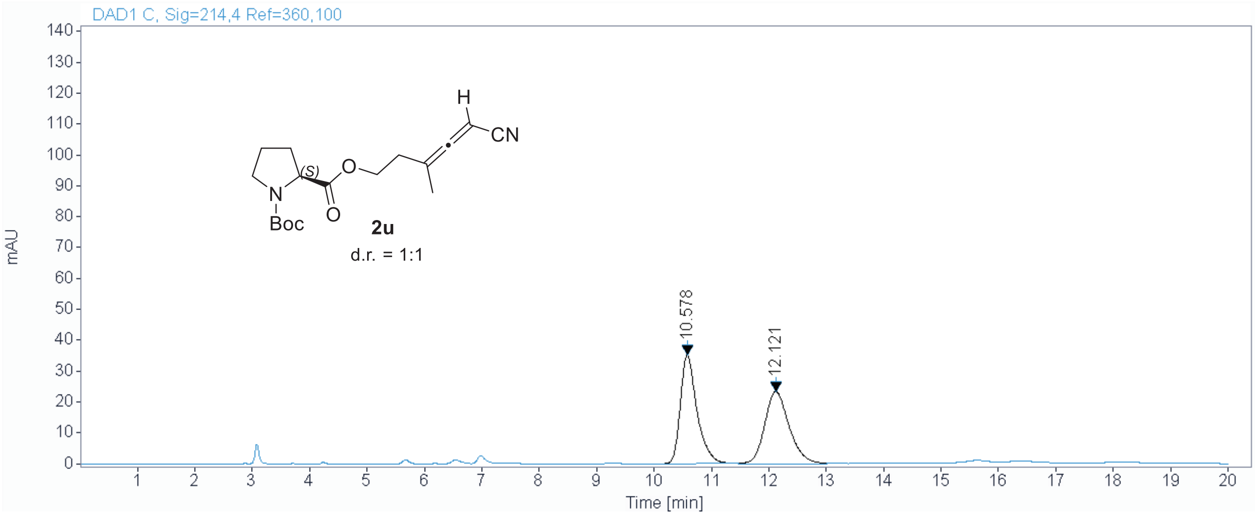

Signal: DAD1 C, Sig=214,4 Ref=360,100

RT [min]	Width [min]	Height	Area	Area%
10.578	0.3303	35.1841	697.2301	50.2261
12.121	0.4923	23.3925	690.9521	49.7739
		Sum	1388.1822	100.0000

Supplementary Figure 114. HPLC spectrum for **2u**

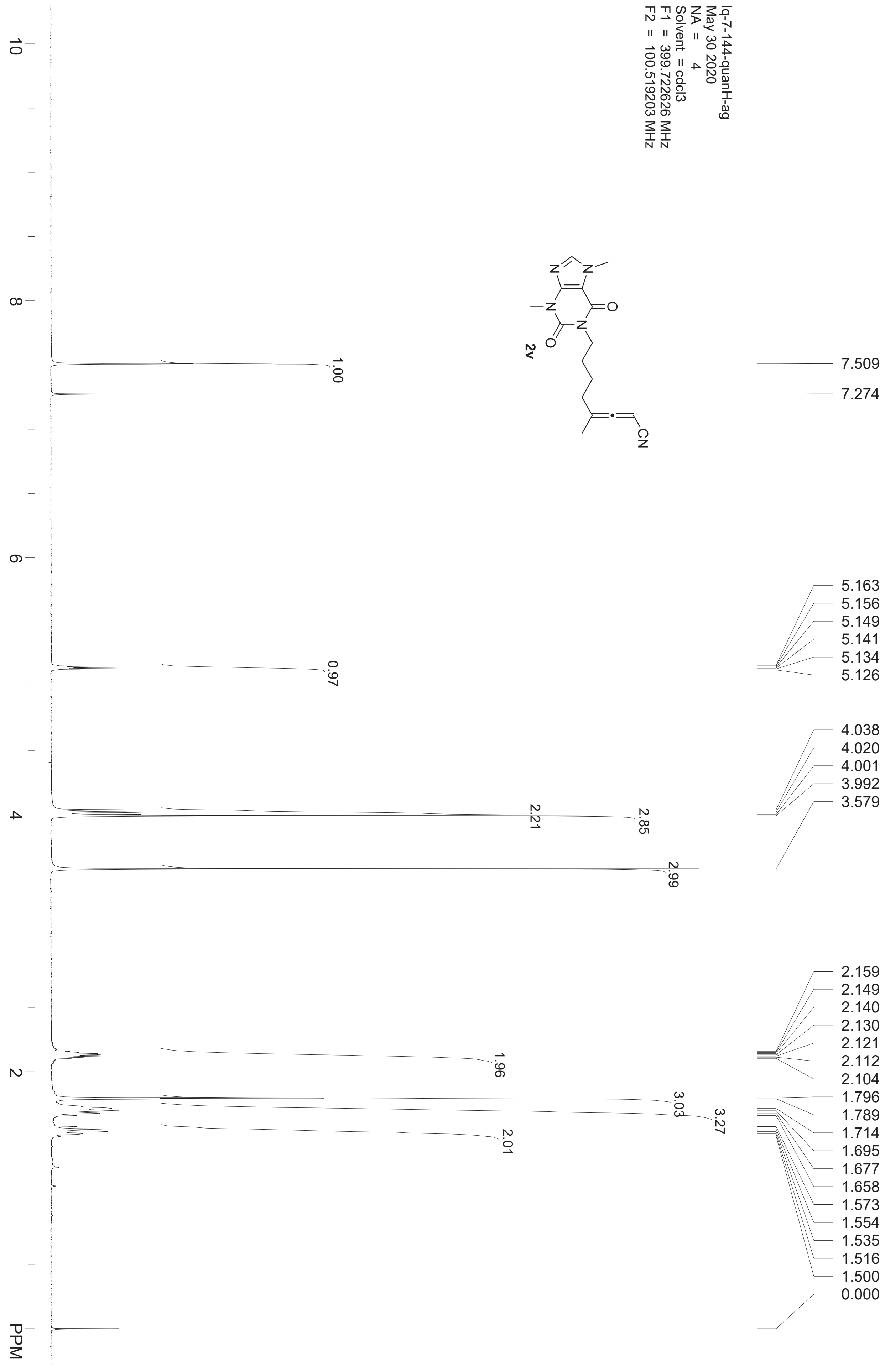

Supplementary Figure 115. ^{1}H NMR (400 MHz, $CDCl_3$) spectrum for **2v**

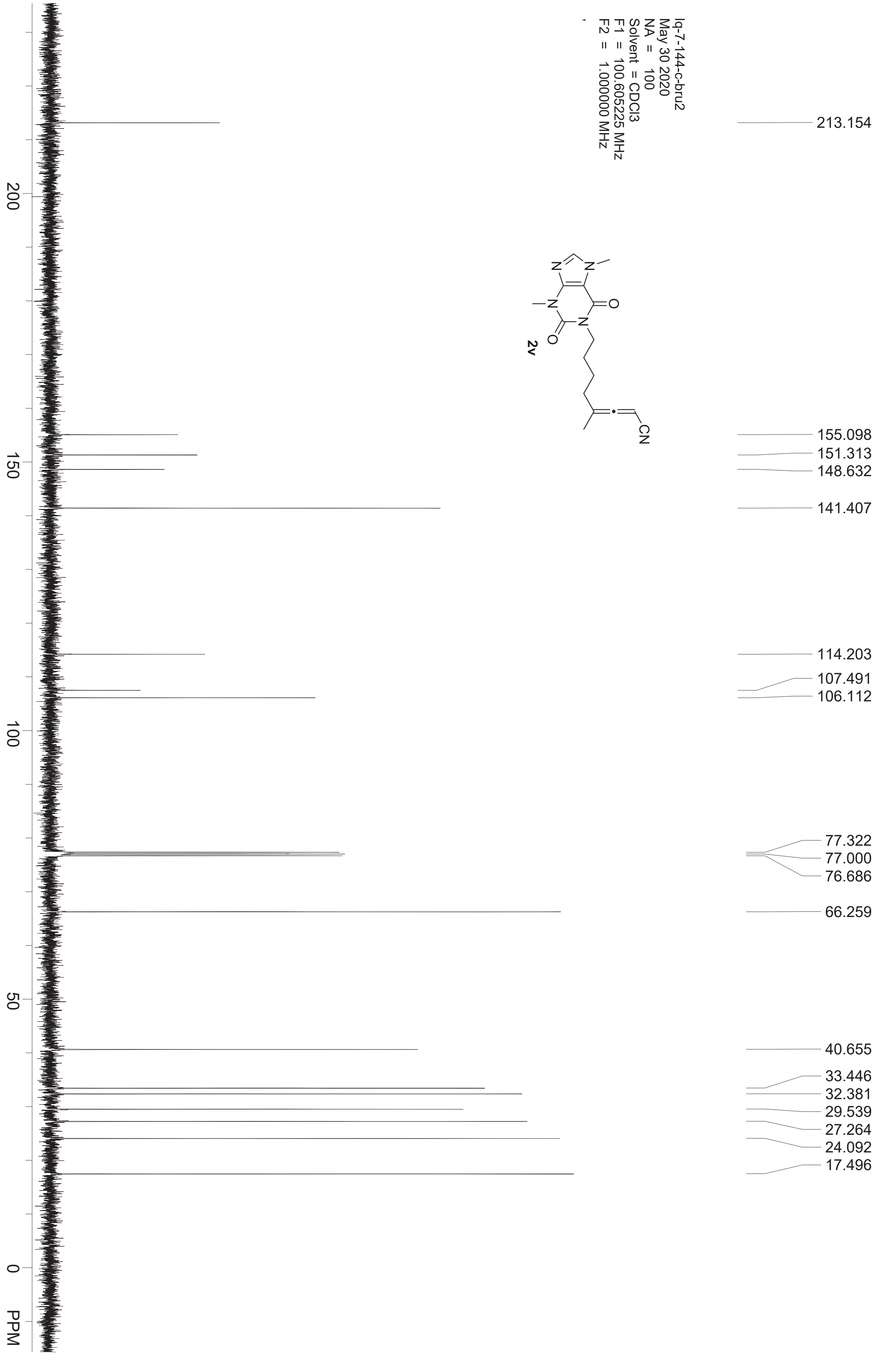

Supplementary Figure 116. ^{13}C NMR (100 MHz, $CDCl_3$) spectrum for **2v**

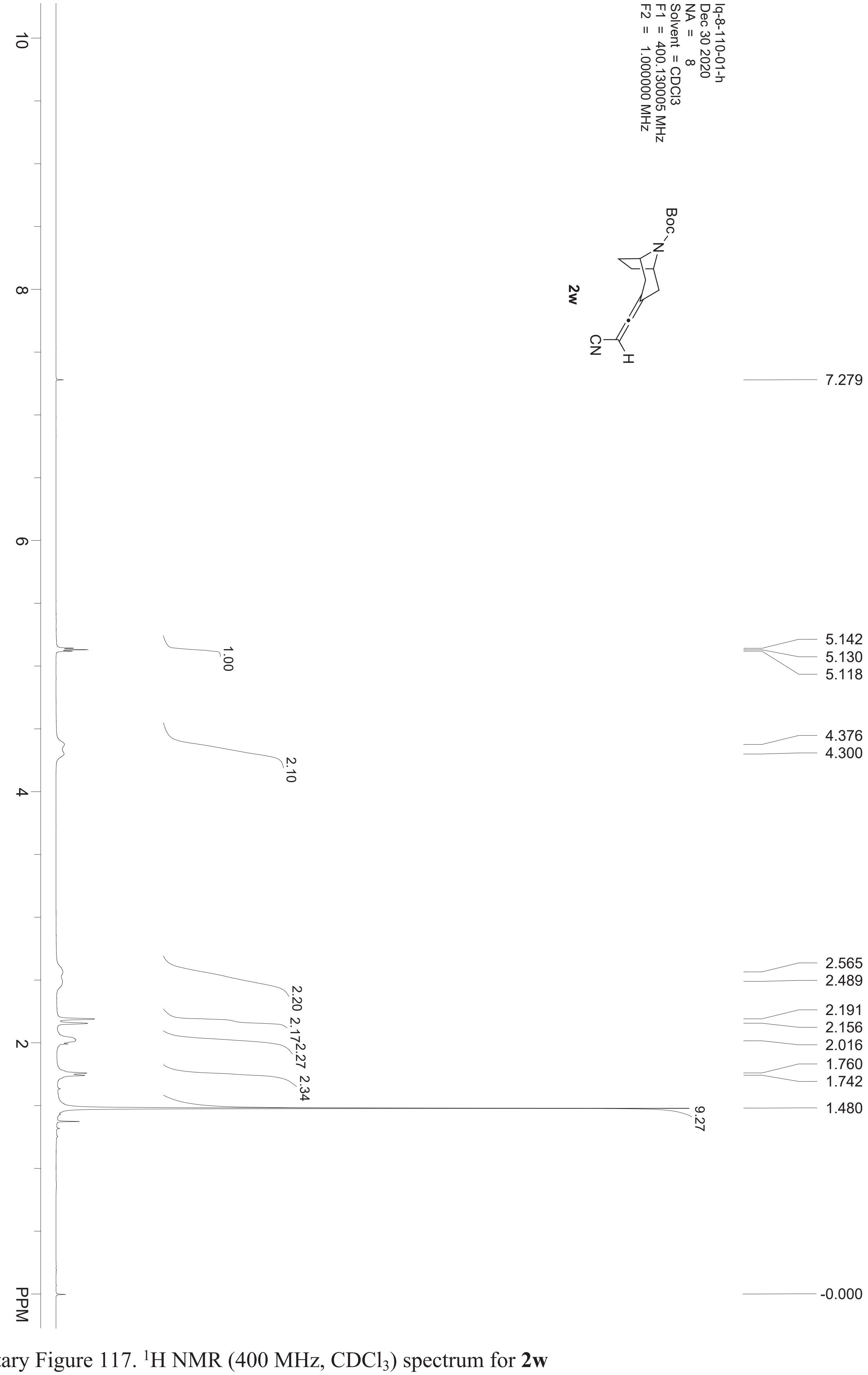

Supplementary Figure 117. ^{1}H NMR (400 MHz, $CDCl_3$) spectrum for **2w**

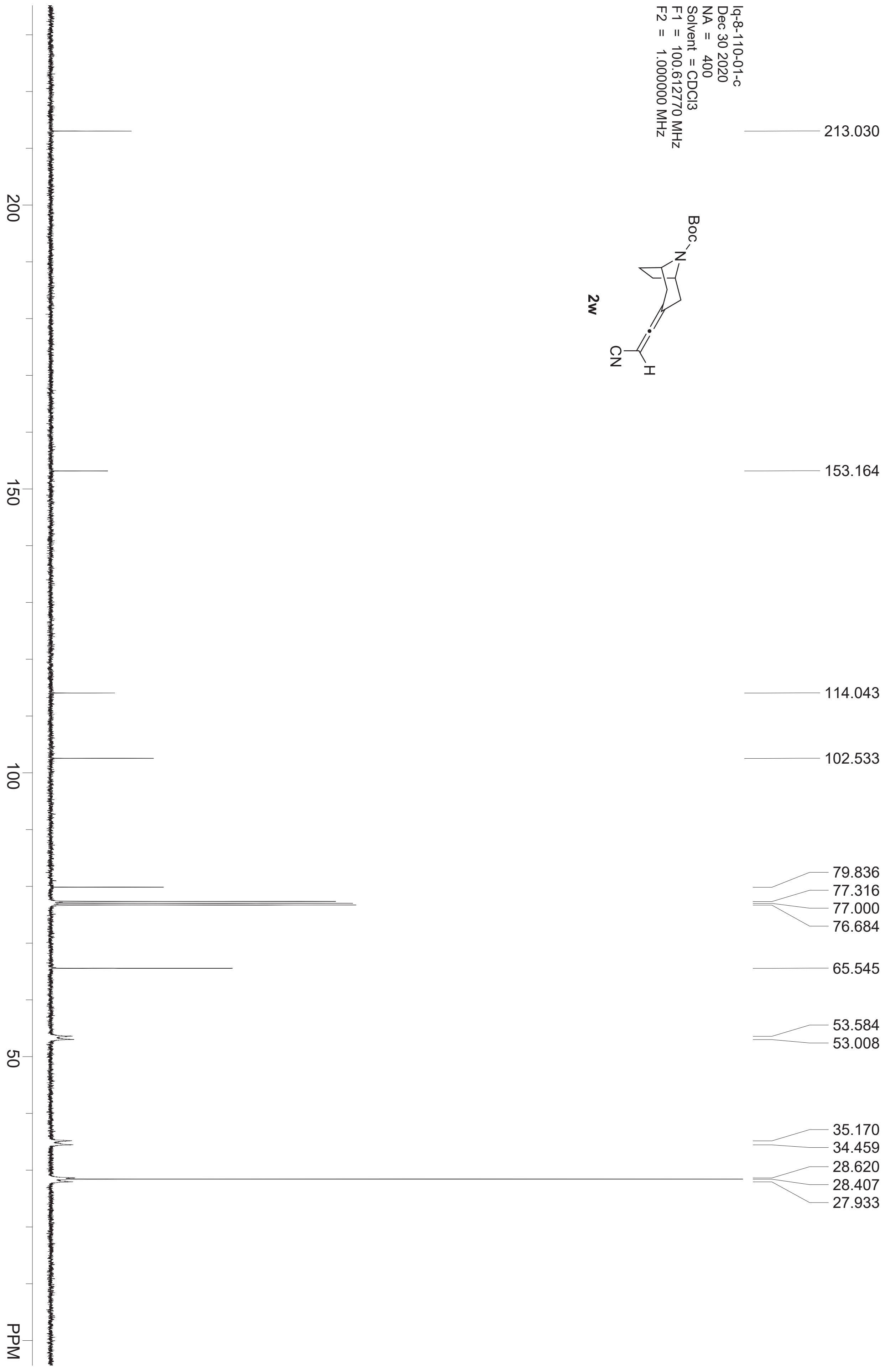

Supplementary Figure 118. ^{13}C NMR (100 MHz, $CDCl_3$) spectrum for **2w**

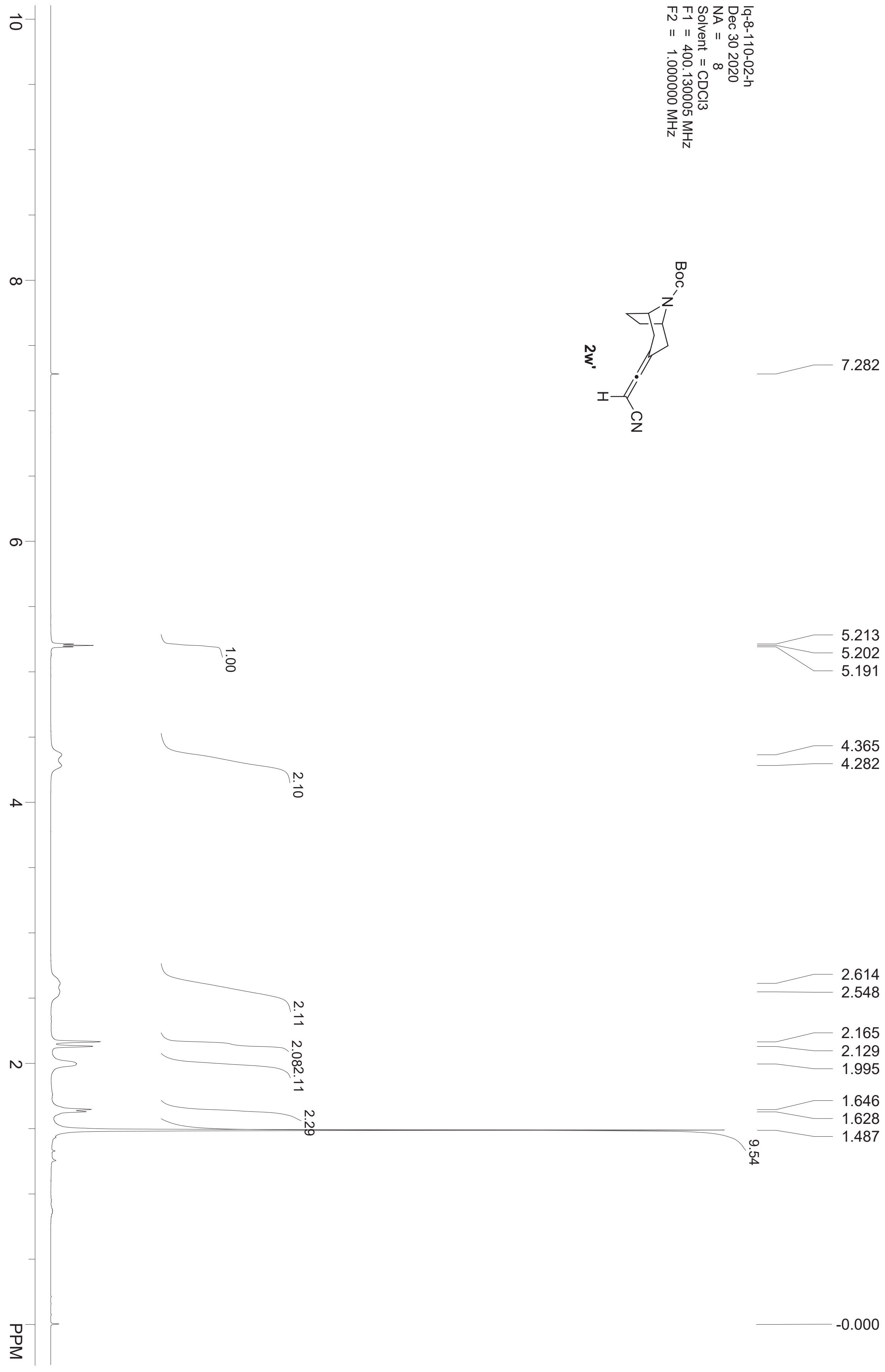

Supplementary Figure 119. ^{1}H NMR (400 MHz, $CDCl_3$) spectrum for **2w'**

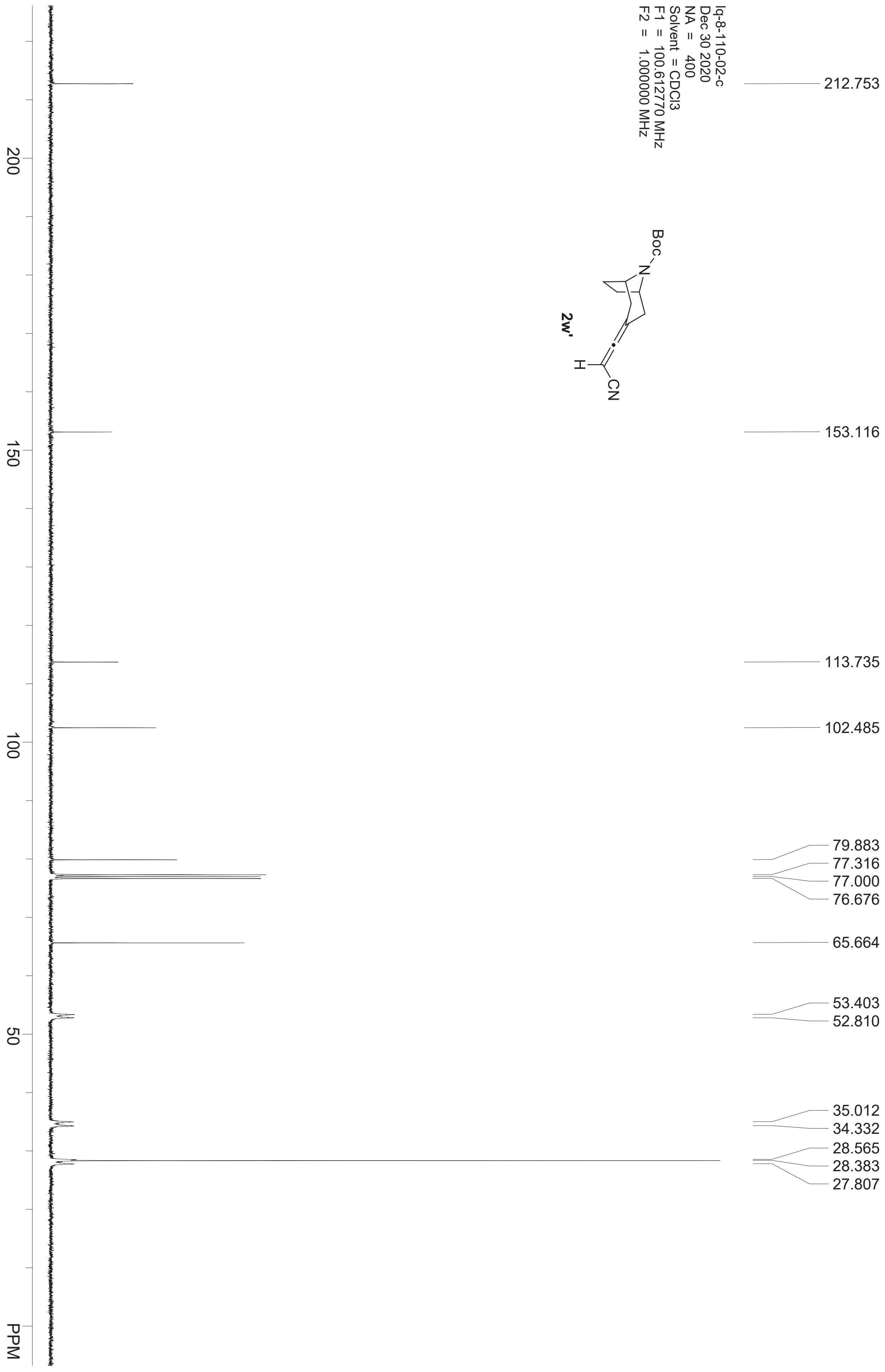

Supplementary Figure 120. ^{13}C NMR (100 MHz, $CDCl_3$) spectrum for **2w'**

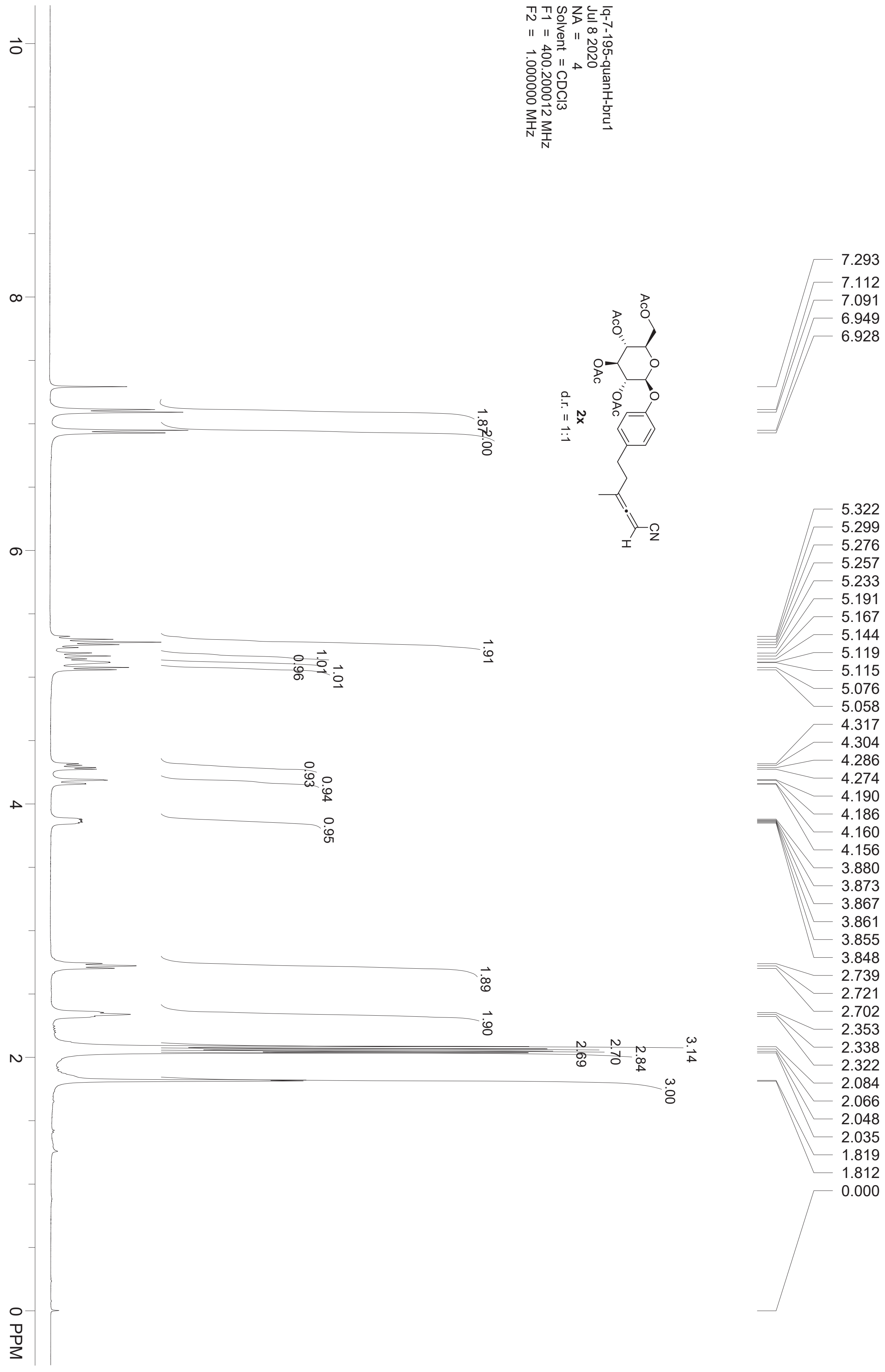

Supplementary Figure 121. ^{1}H NMR (400 MHz, $CDCl_3$) spectrum for **2x**

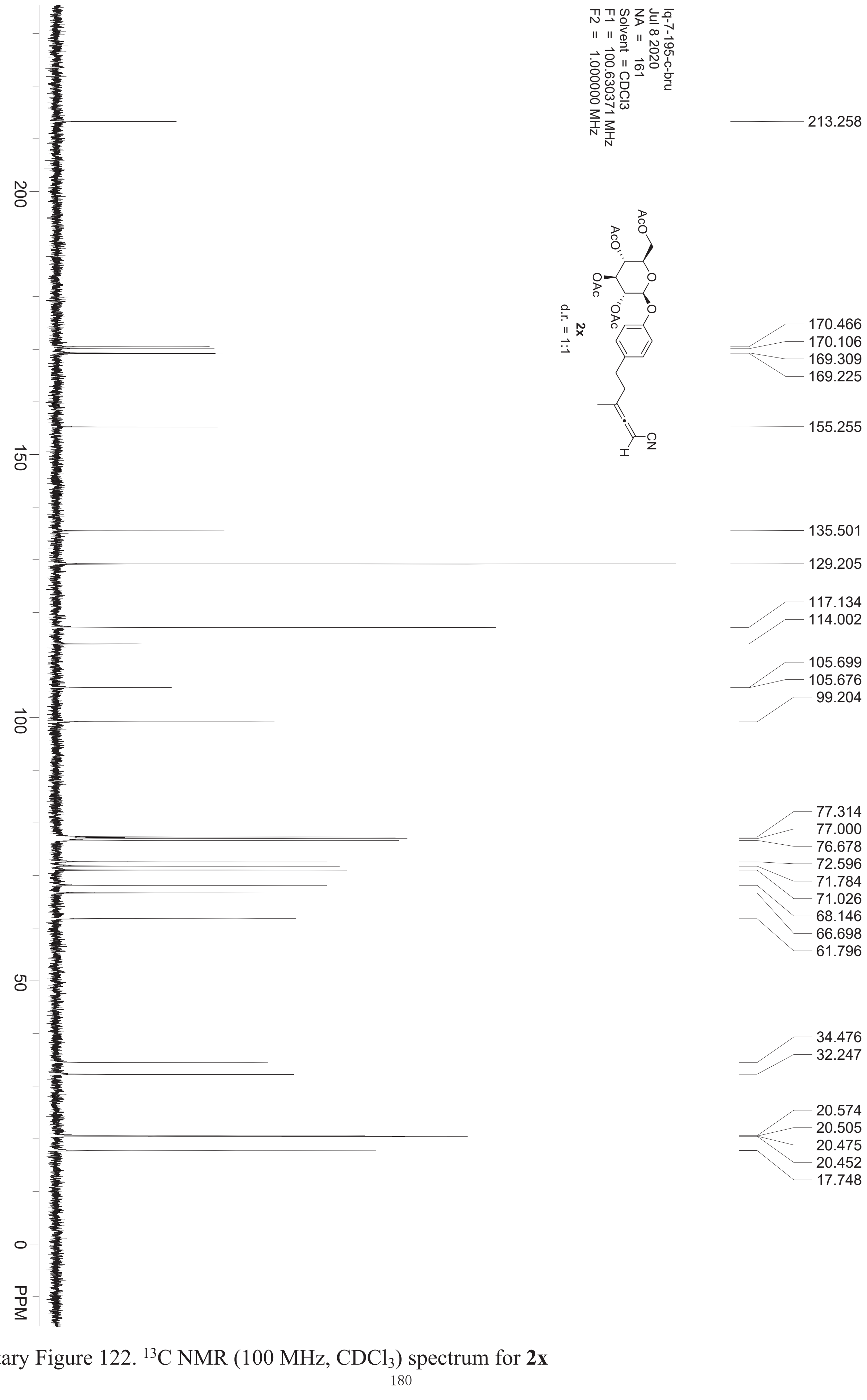

Supplementary Figure 122. ^{13}C NMR (100 MHz, $CDCl_3$) spectrum for **2x**

Default Individual Report

SAMPLE INFORMATION

Sample Name:	lq-7-195-if-80-20-1-214	Acquired By:	System
Sample Type:	Unknown	Sample Set Name:	
Vial:	1	Acq. Method Set:	HPLC
Injection #:	37	Processing Method:	Default
Injection Volume:	10.00 ul	Channel Name:	W2489 ChA
Run Time:	100.0 Minutes	Proc. Chnl. Descr.:	W2489 ChA 214nm
Date Acquired:	7/17/2020 7:34:57 PM EDT		
Date Processed:	7/17/2020 8:05:22 PM EDT		

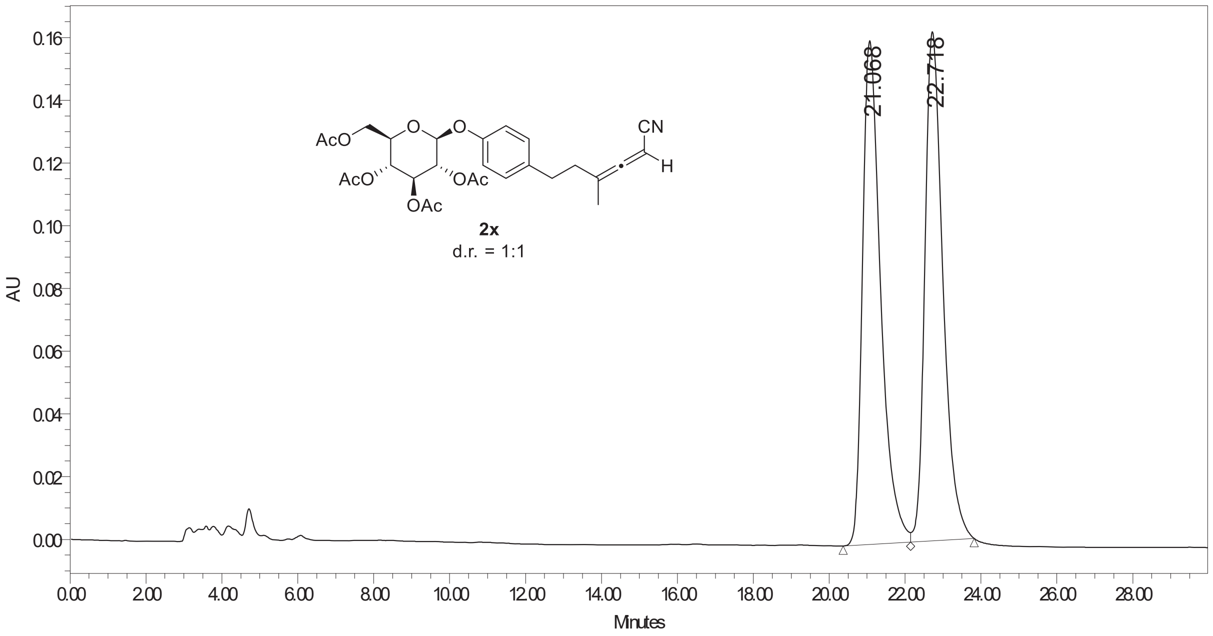

	RT	Area	% Area	Height
1	21.068	5347230	49.92	160625
2	22.718	5363853	50.08	162289

Reported by User: System
Report Method: Default Individual Report
Report Method ID: 1003 1003
Page: 1 of 1

Project Name: HPLC_515
Date Printed:
7/17/2020
8:05:32 PM America/New_York

Supplementary Figure 123. HPLC spectrum for **2x**

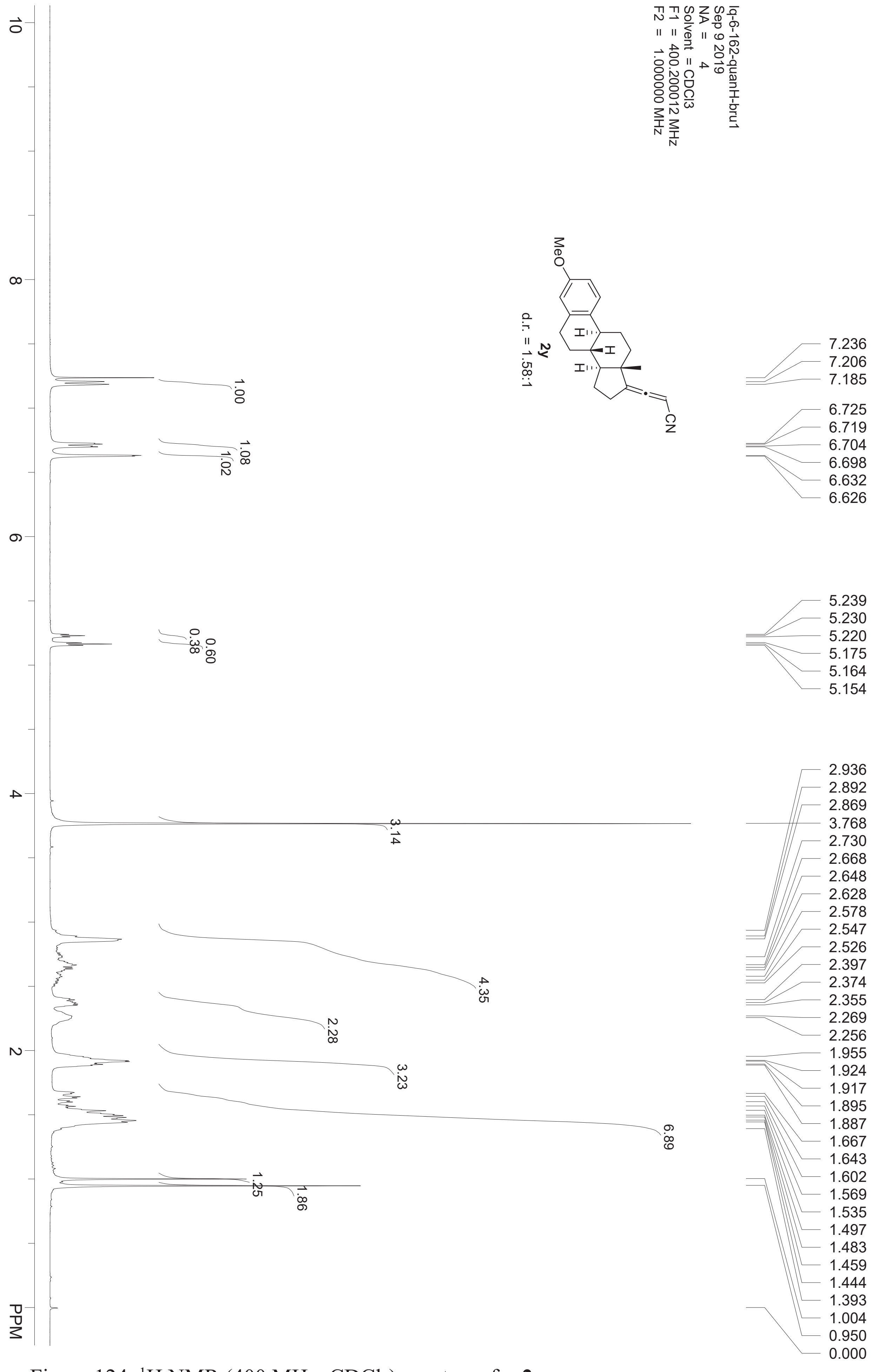

Supplementary Figure 124. ^{1}H NMR (400 MHz, $CDCl_3$) spectrum for **2y**

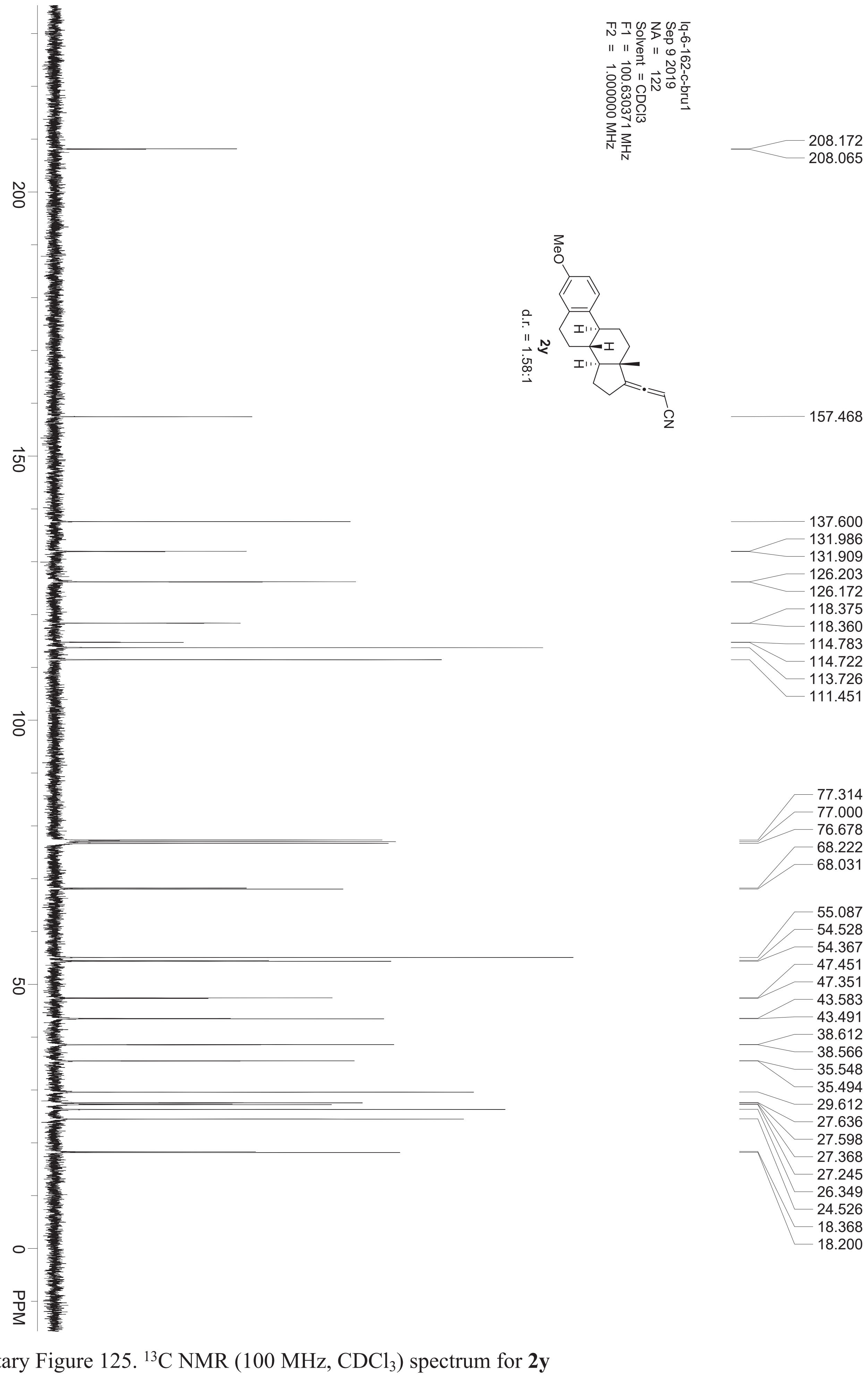

Supplementary Figure 125. ^{13}C NMR (100 MHz, $CDCl_3$) spectrum for **2y**

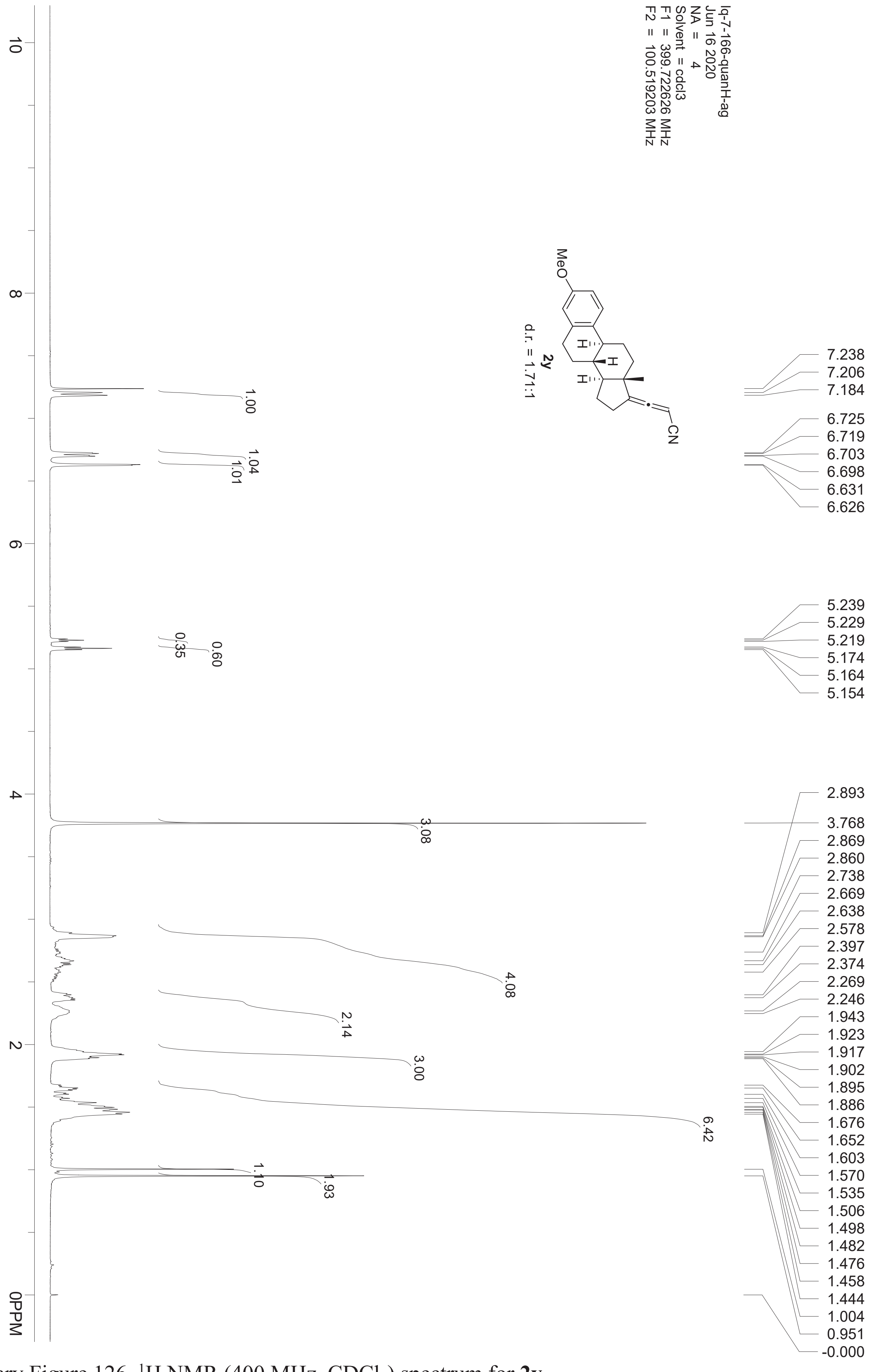

Supplementary Figure 126. ^{1}H NMR (400 MHz, $CDCl_3$) spectrum for **2y**

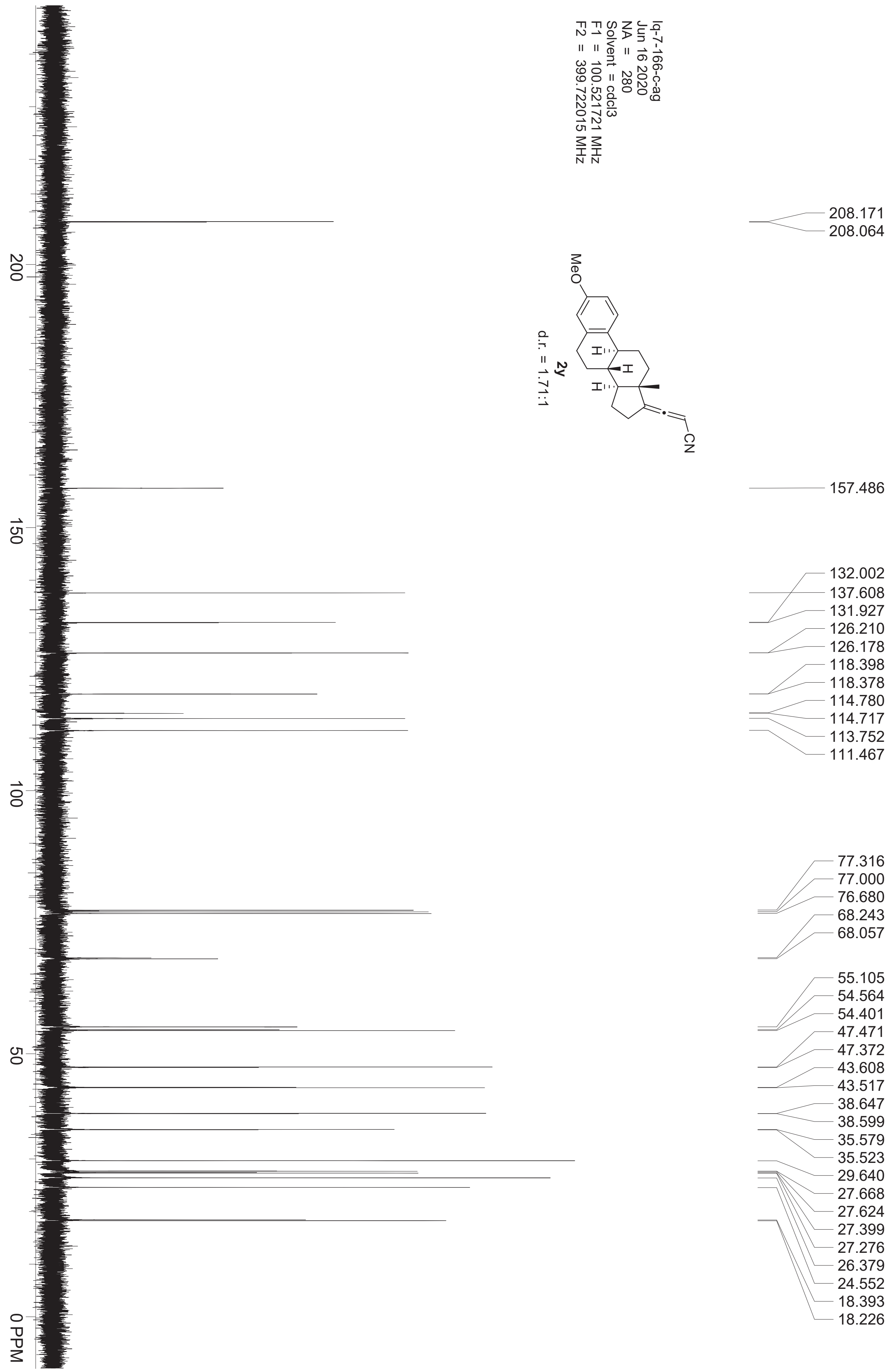

Supplementary Figure 127. ^{13}C NMR (100 MHz, $CDCl_3$) spectrum for **2y**

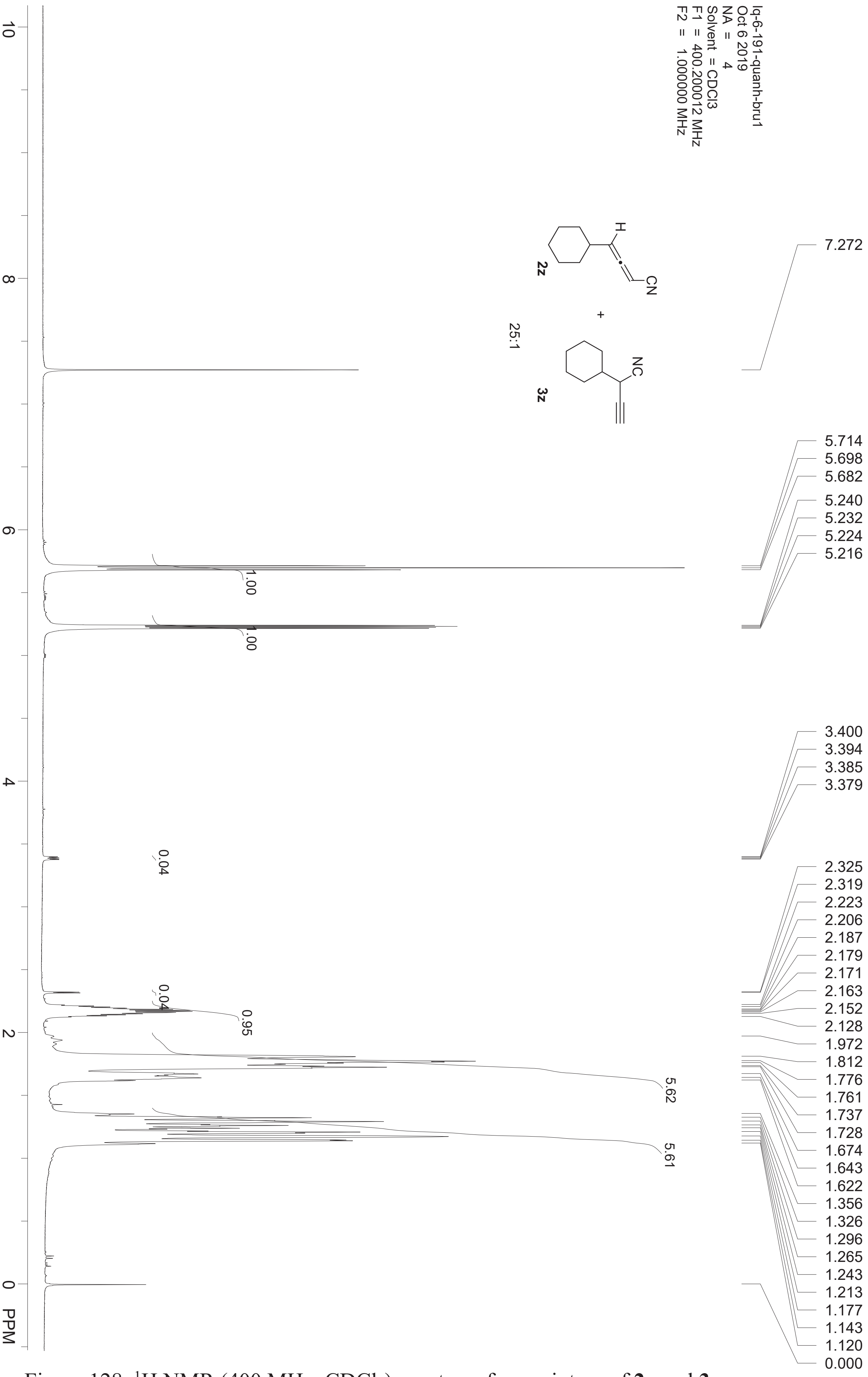

Supplementary Figure 128. ^{1}H NMR (400 MHz, $CDCl_3$) spectrum for a mixture of **2z** and **3z**

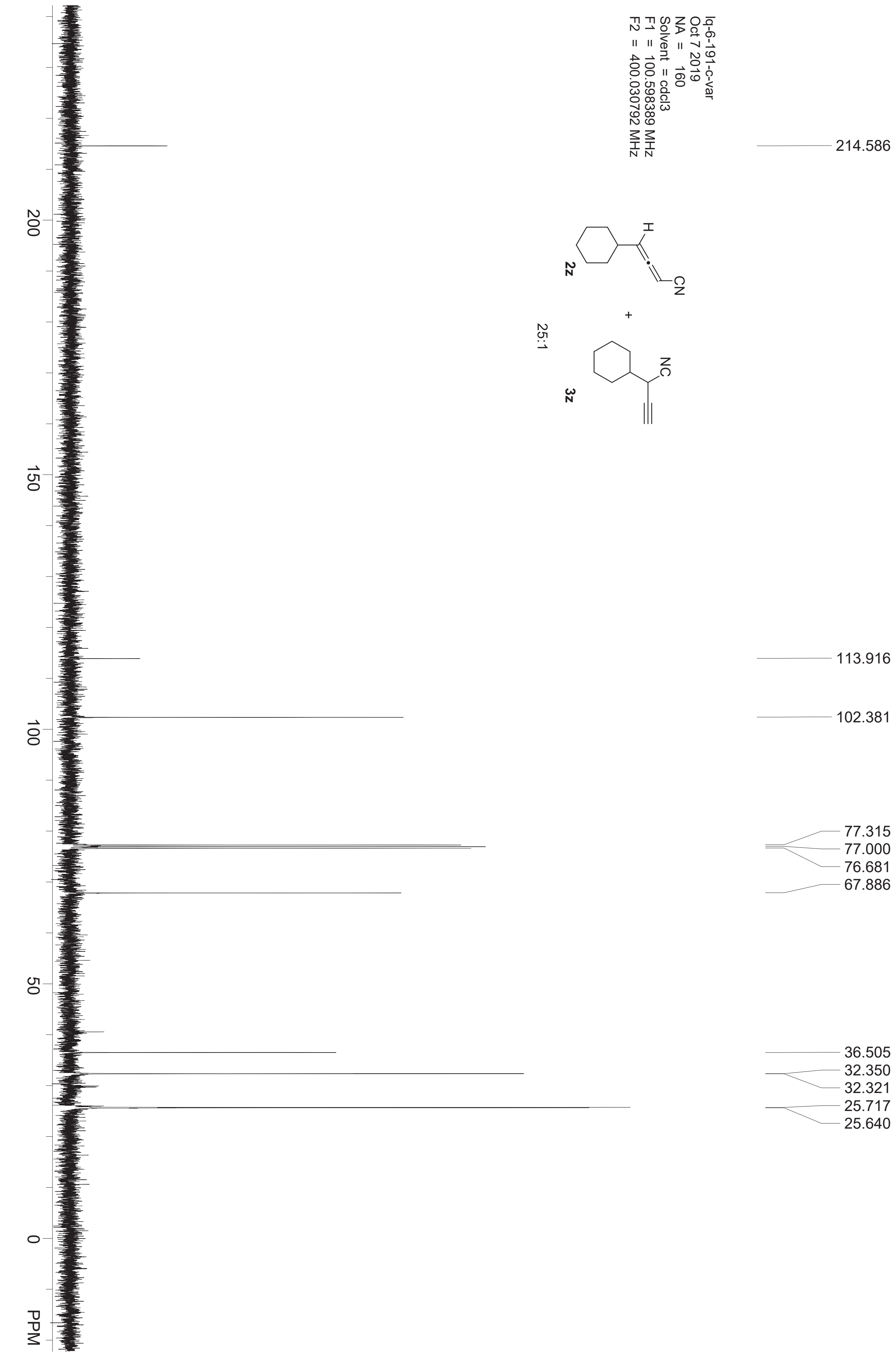

Supplementary Figure 129. ^{13}C NMR (100 MHz, $CDCl_3$) spectrum for a mixture of **2z** and **3z**

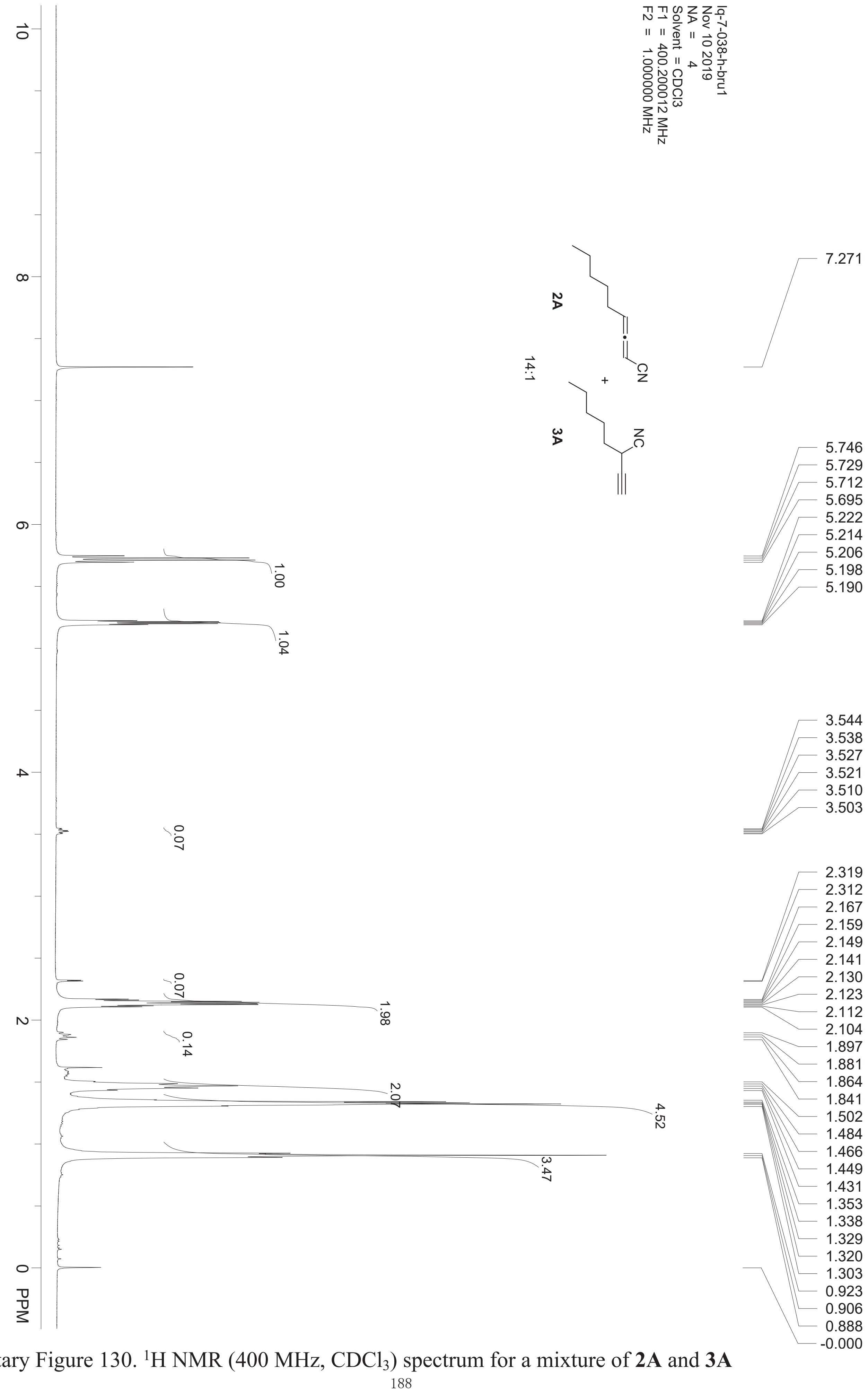

Supplementary Figure 130. ^{1}H NMR (400 MHz, $CDCl_3$) spectrum for a mixture of **2A** and **3A**

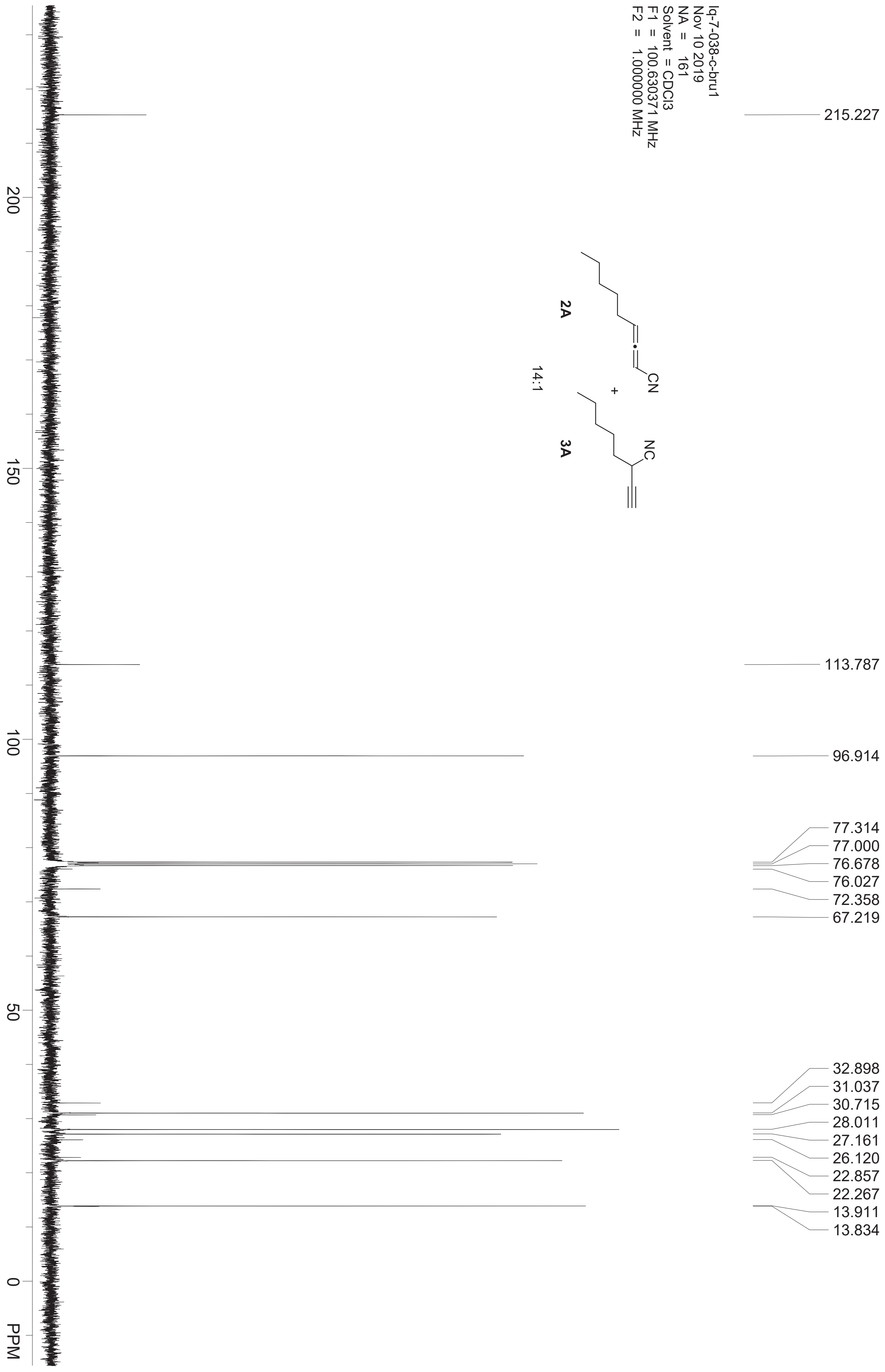

Supplementary Figure 131. ^{13}C NMR (100 MHz, $CDCl_3$) spectrum for a mixture of **2A** and **3A**

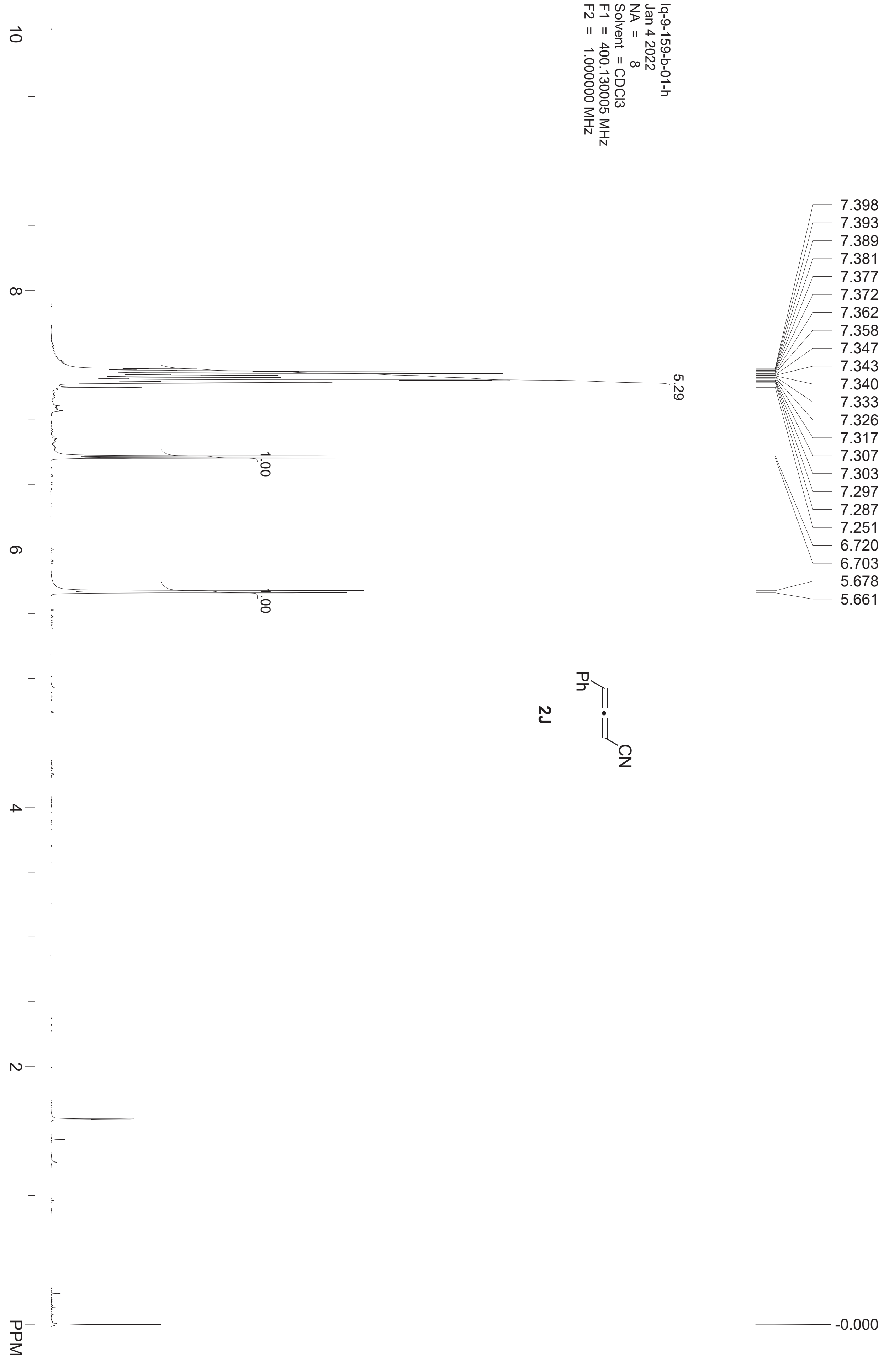

Supplementary Figure 132. ^{1}H NMR (400 MHz, $CDCl_3$) spectrum for **2J**

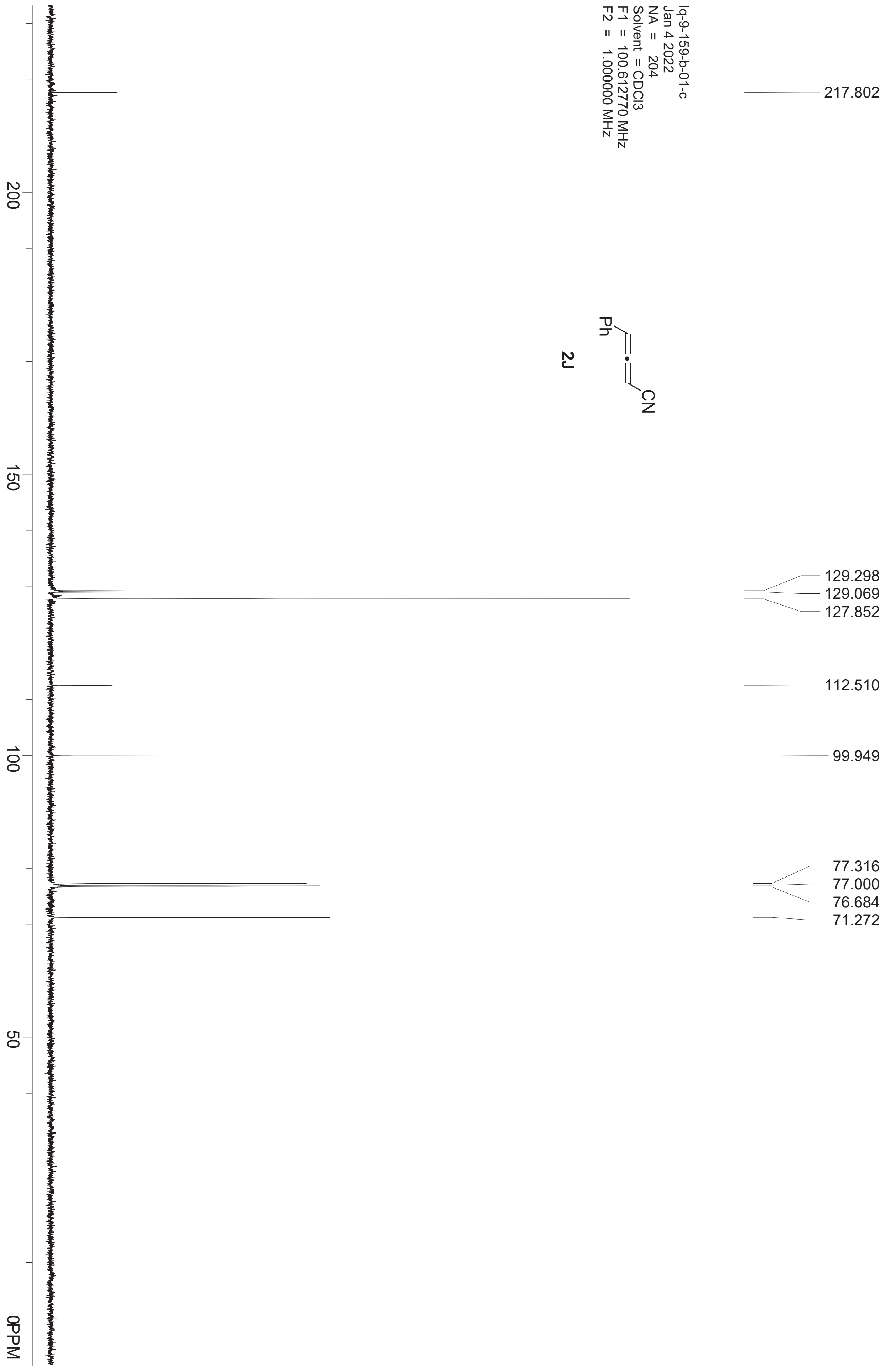

Supplementary Figure 133. ^{13}C NMR (100 MHz, $CDCl_3$) spectrum for **2J**

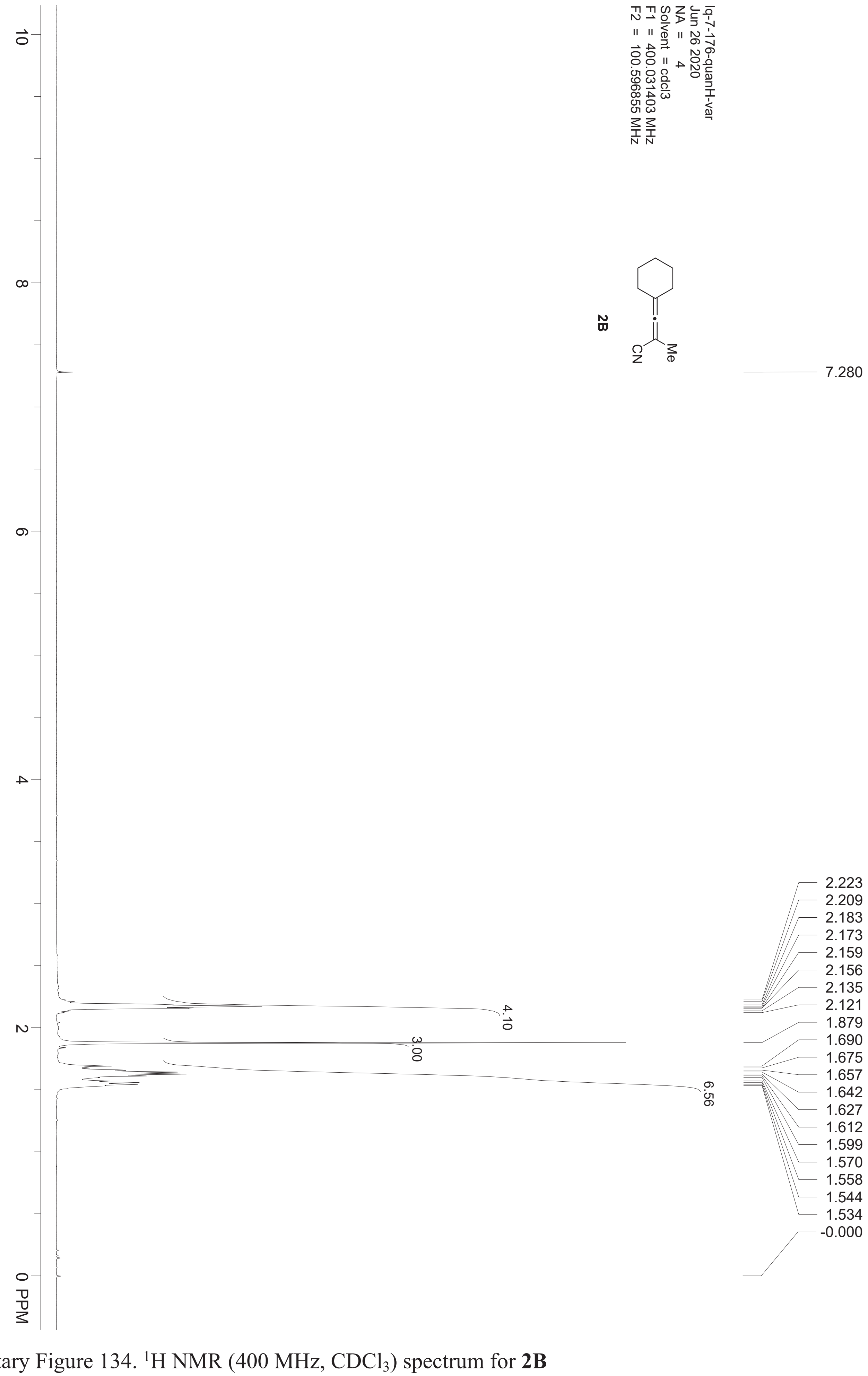

Supplementary Figure 134. ^{1}H NMR (400 MHz, $CDCl_3$) spectrum for **2B**

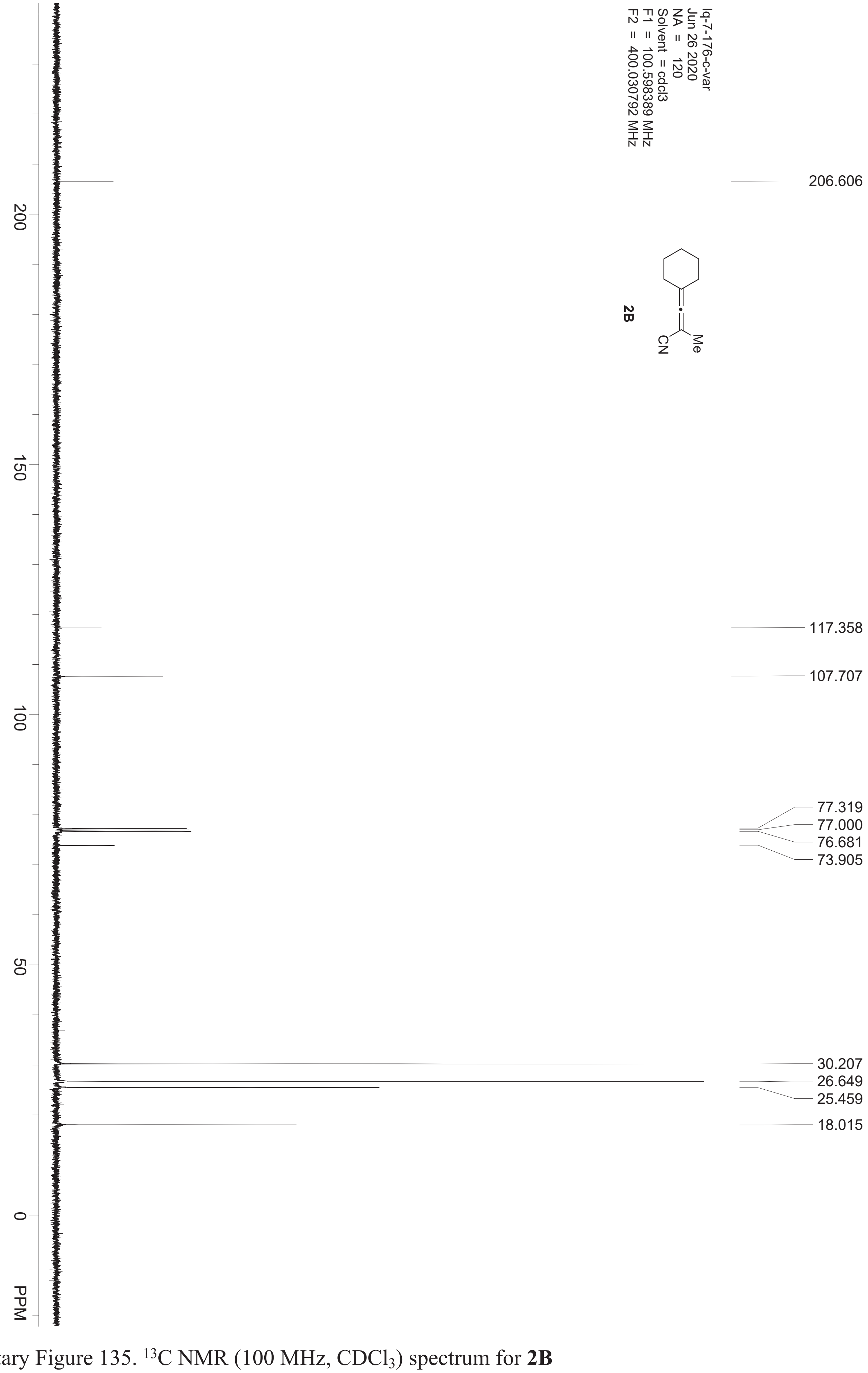

Supplementary Figure 135. ^{13}C NMR (100 MHz, $CDCl_3$) spectrum for **2B**

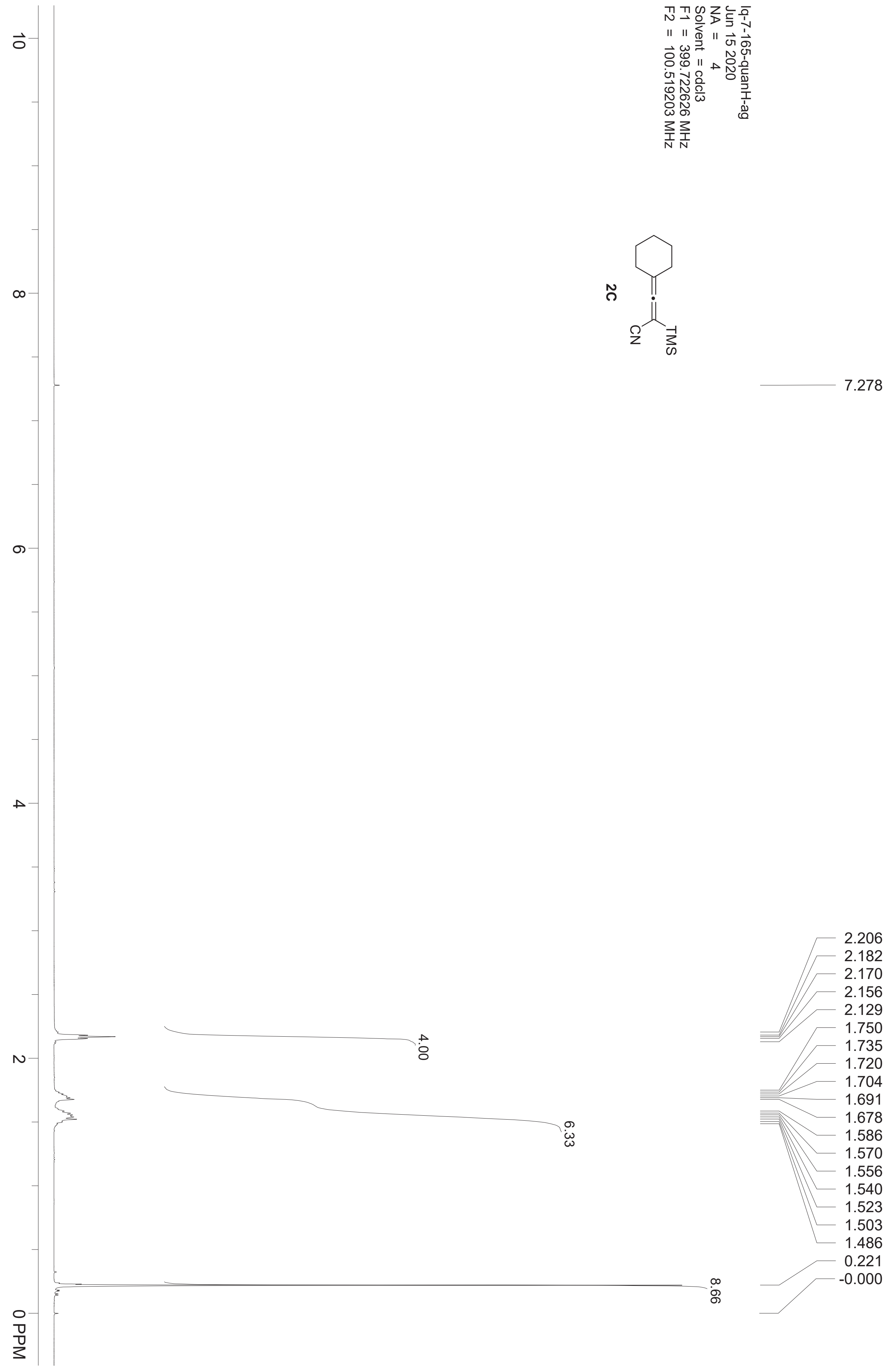

Supplementary Figure 136. ^{1}H NMR (400 MHz, $CDCl_3$) spectrum for **2C**

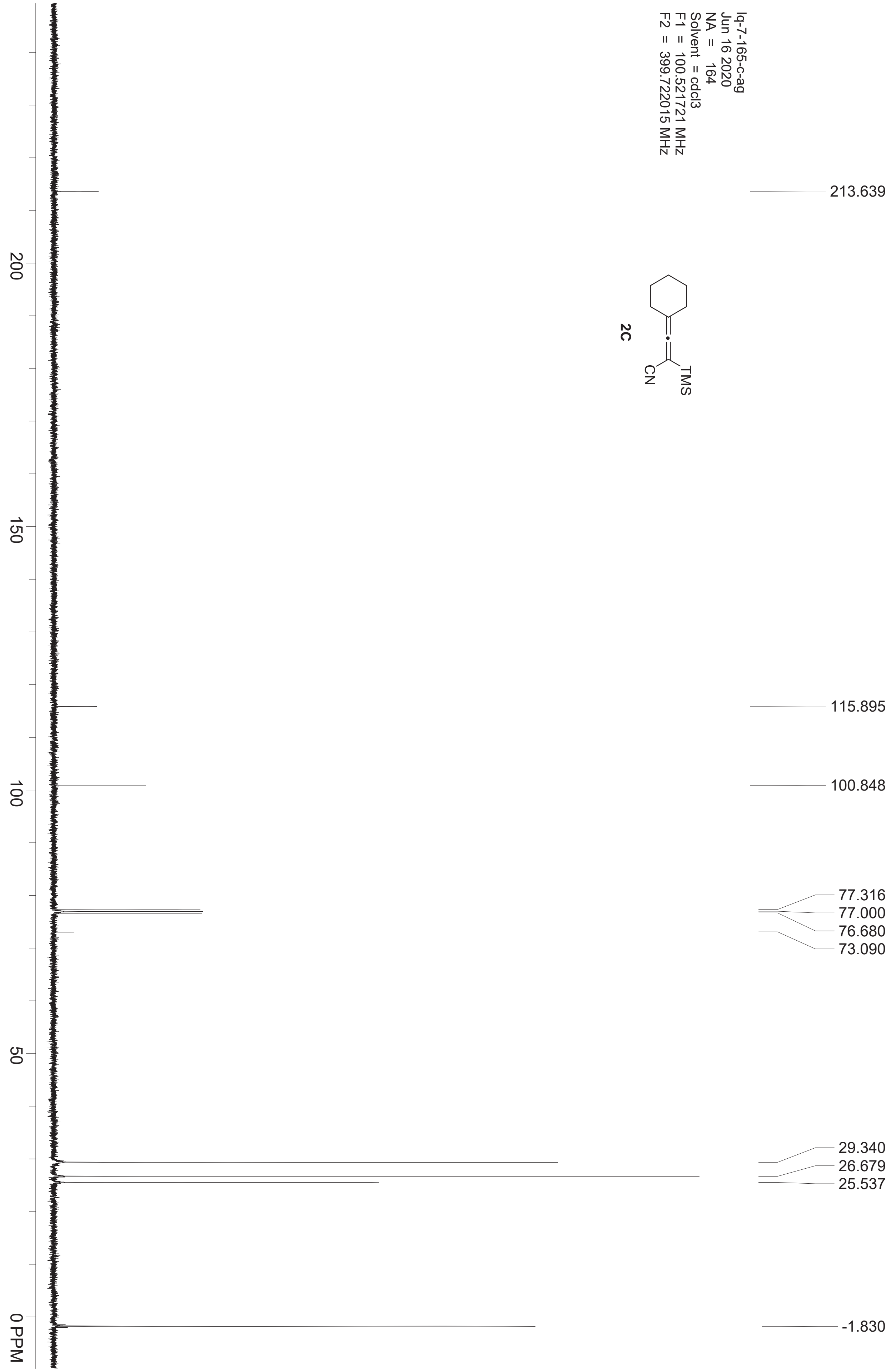

Supplementary Figure 137. ^{13}C NMR (100 MHz, $CDCl_3$) spectrum for **2C**

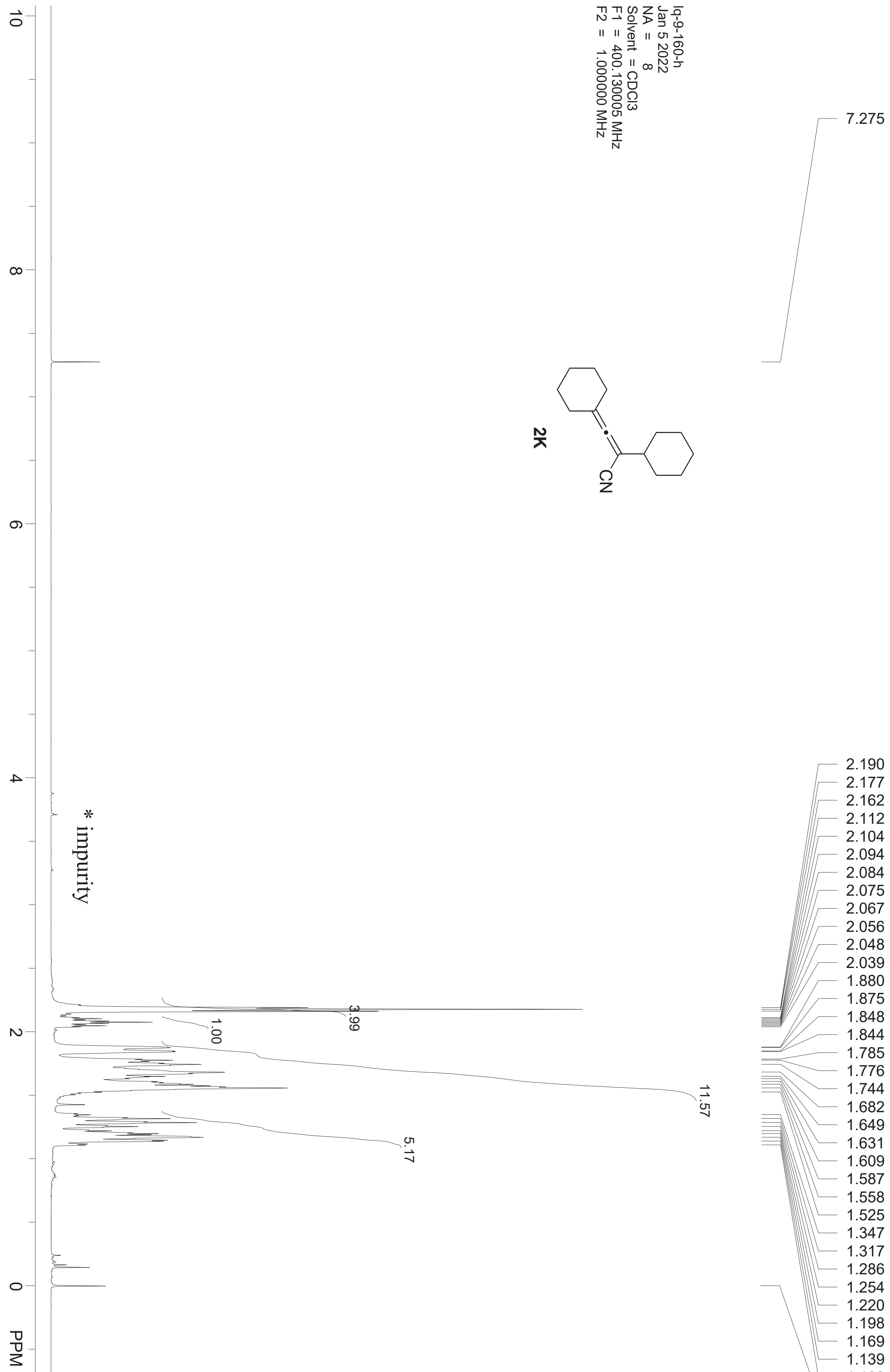

Supplementary Figure 138. ^{1}H NMR (400 MHz, $CDCl_3$) spectrum for **2K**

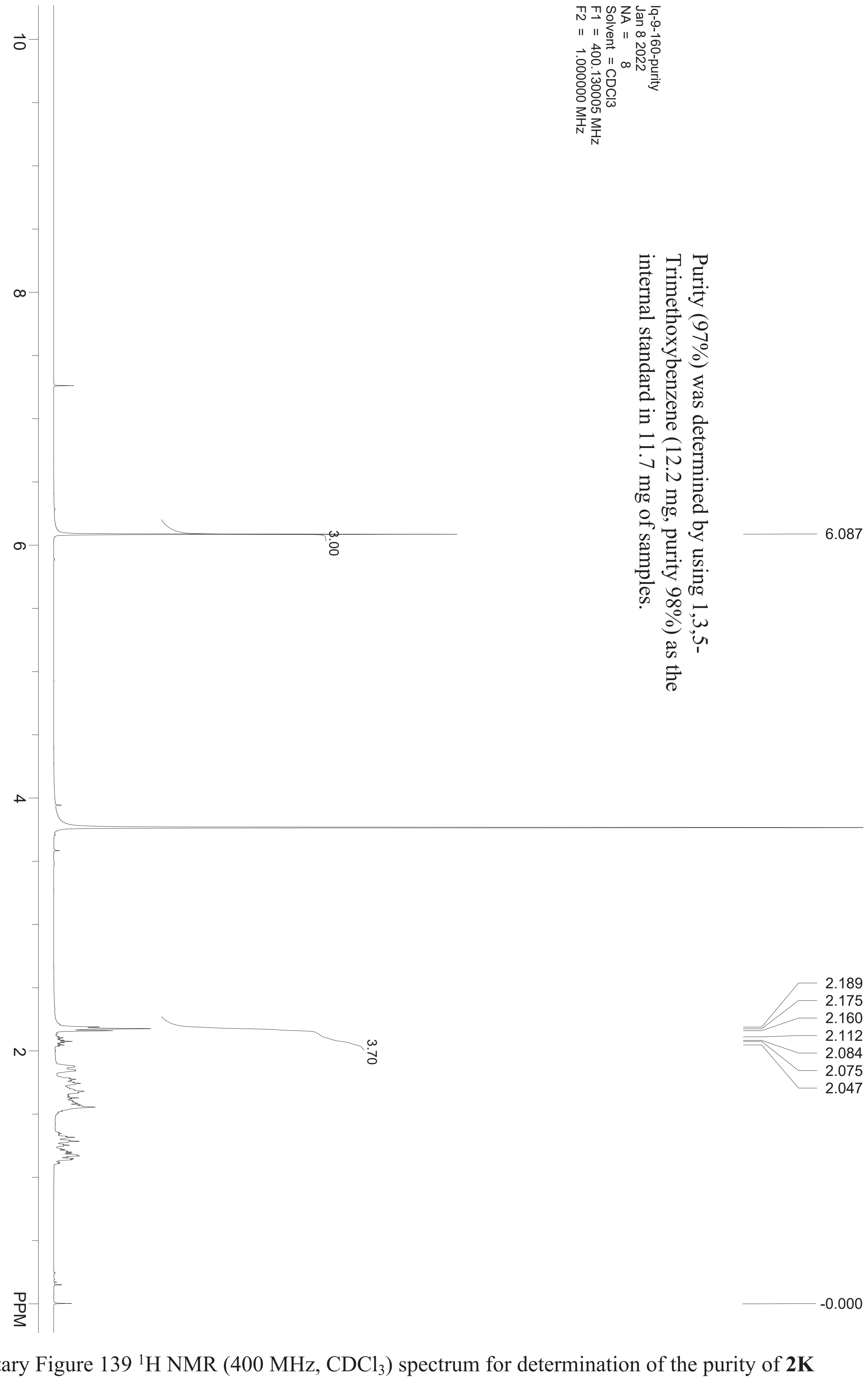

Supplementary Figure 139 ^{1}H NMR (400 MHz, $CDCl_3$) spectrum for determination of the purity of **2K**

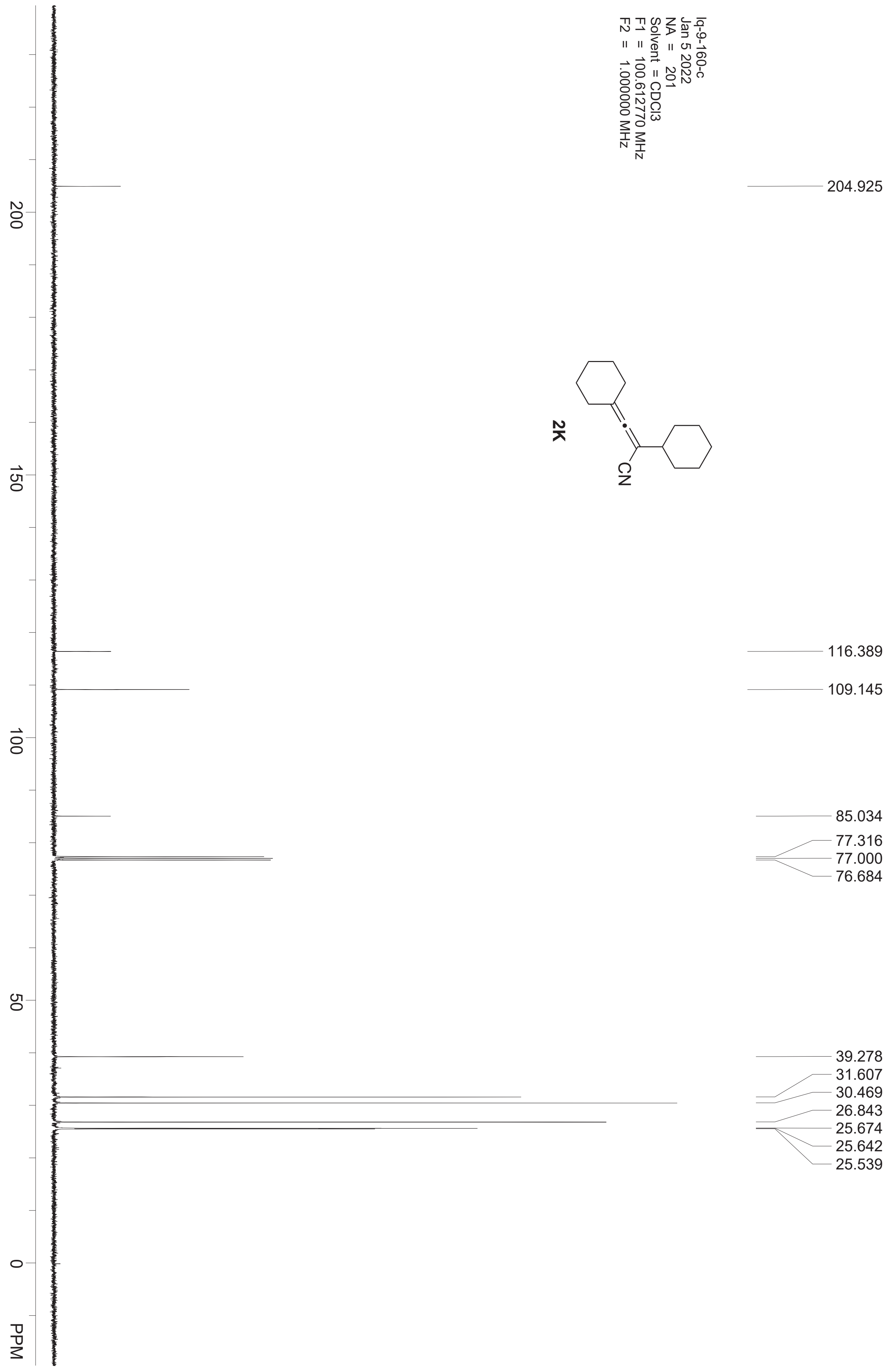

Supplementary Figure 140. ^{13}C NMR (100 MHz, $CDCl_3$) spectrum for **2K**

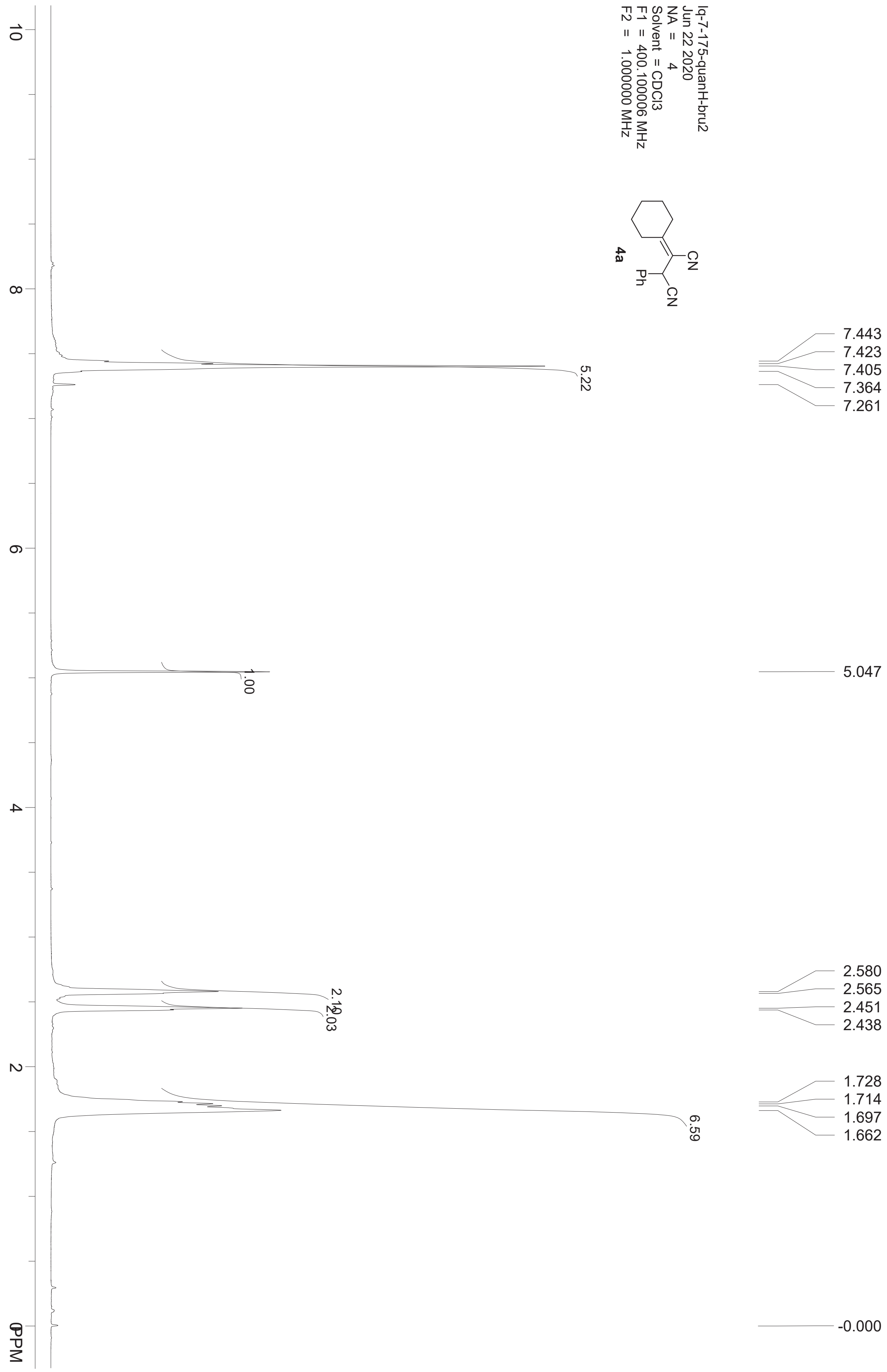

Supplementary Figure 141. ^{1}H NMR (400 MHz, $CDCl_3$) spectrum for **4a**

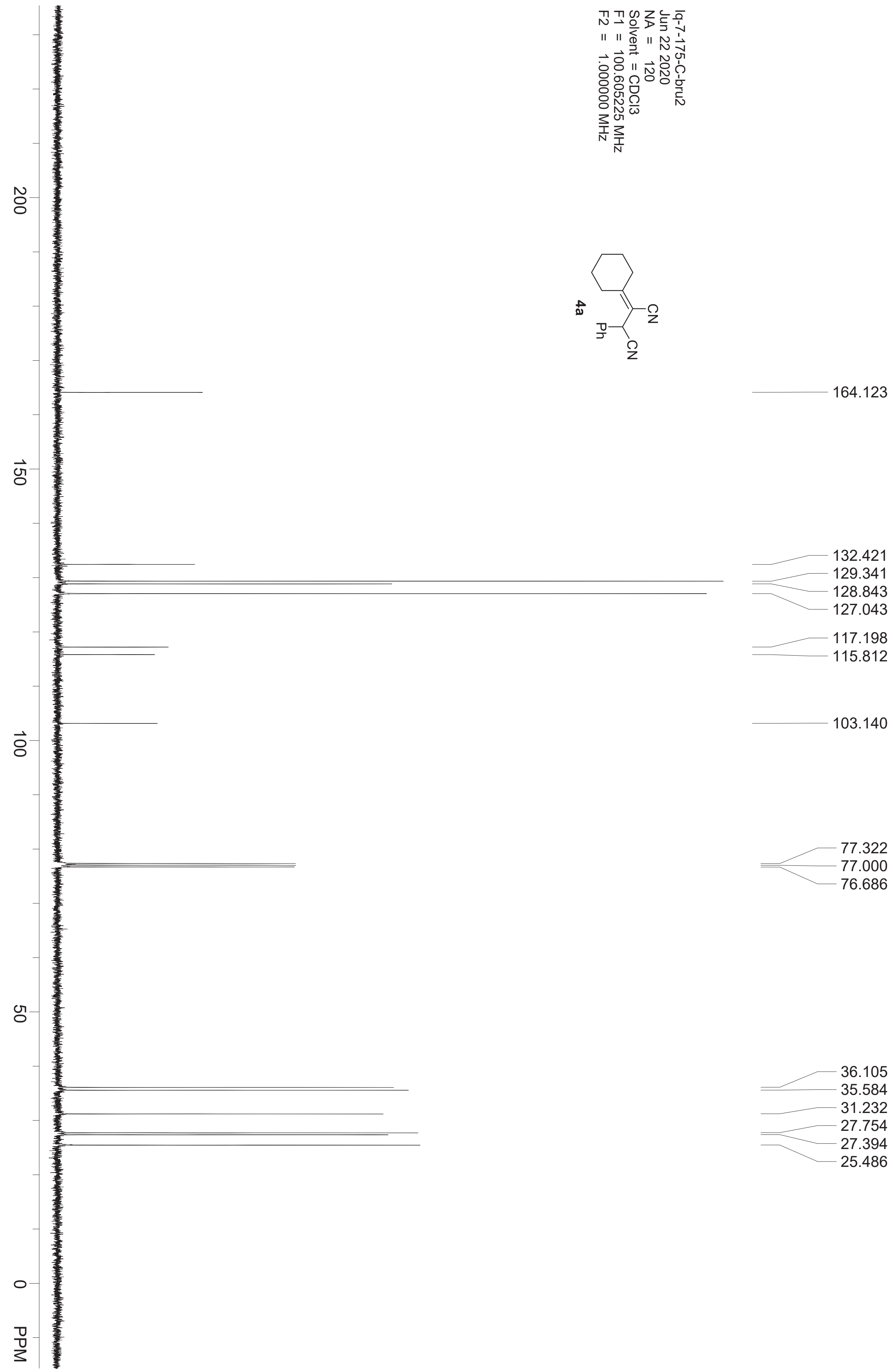

Supplementary Figure 142. ^{13}C NMR (100 MHz, $CDCl_3$) spectrum for **4a**

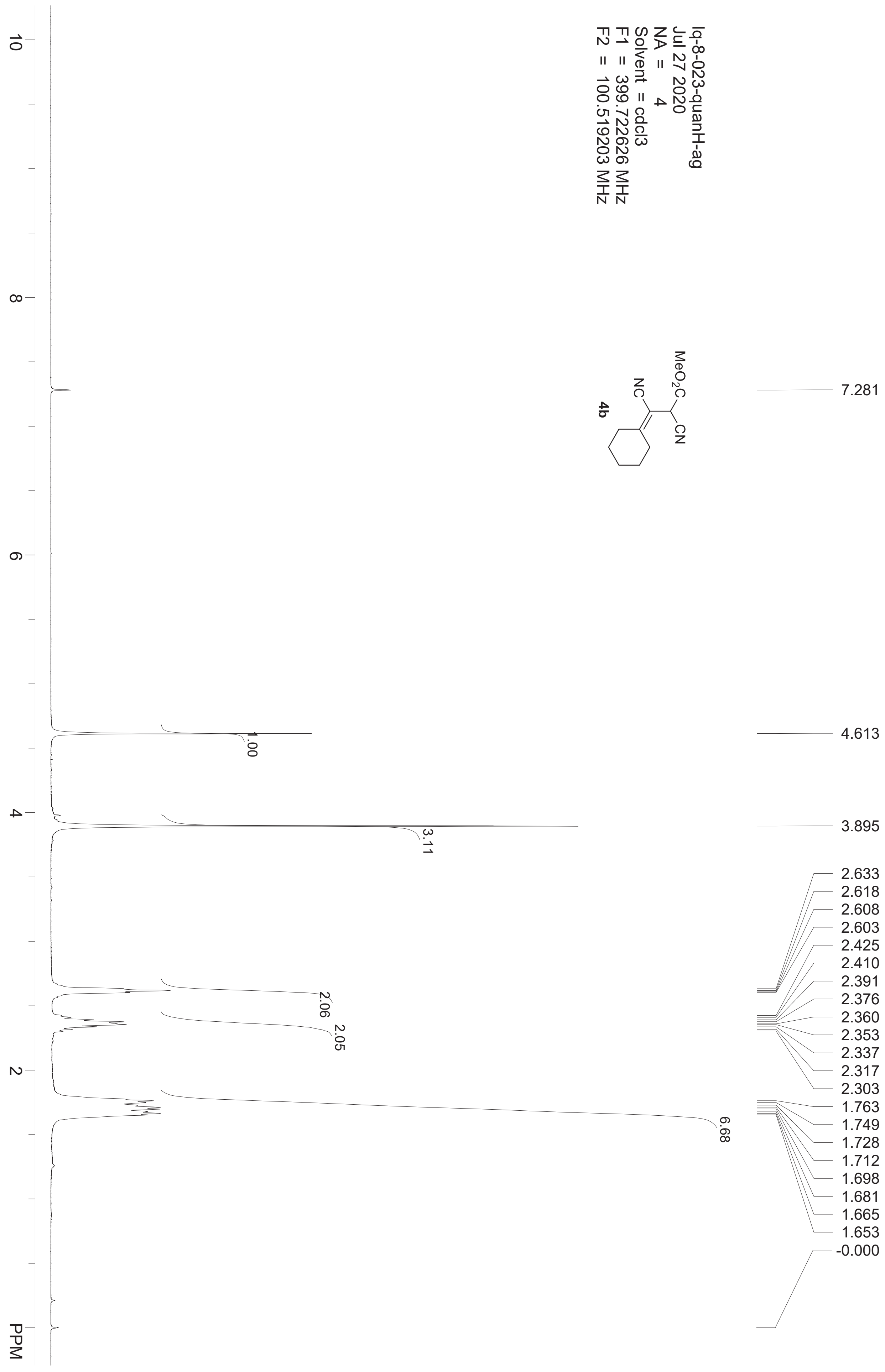

Supplementary Figure 143. ^{1}H NMR (400 MHz, $CDCl_3$) spectrum for **4b**

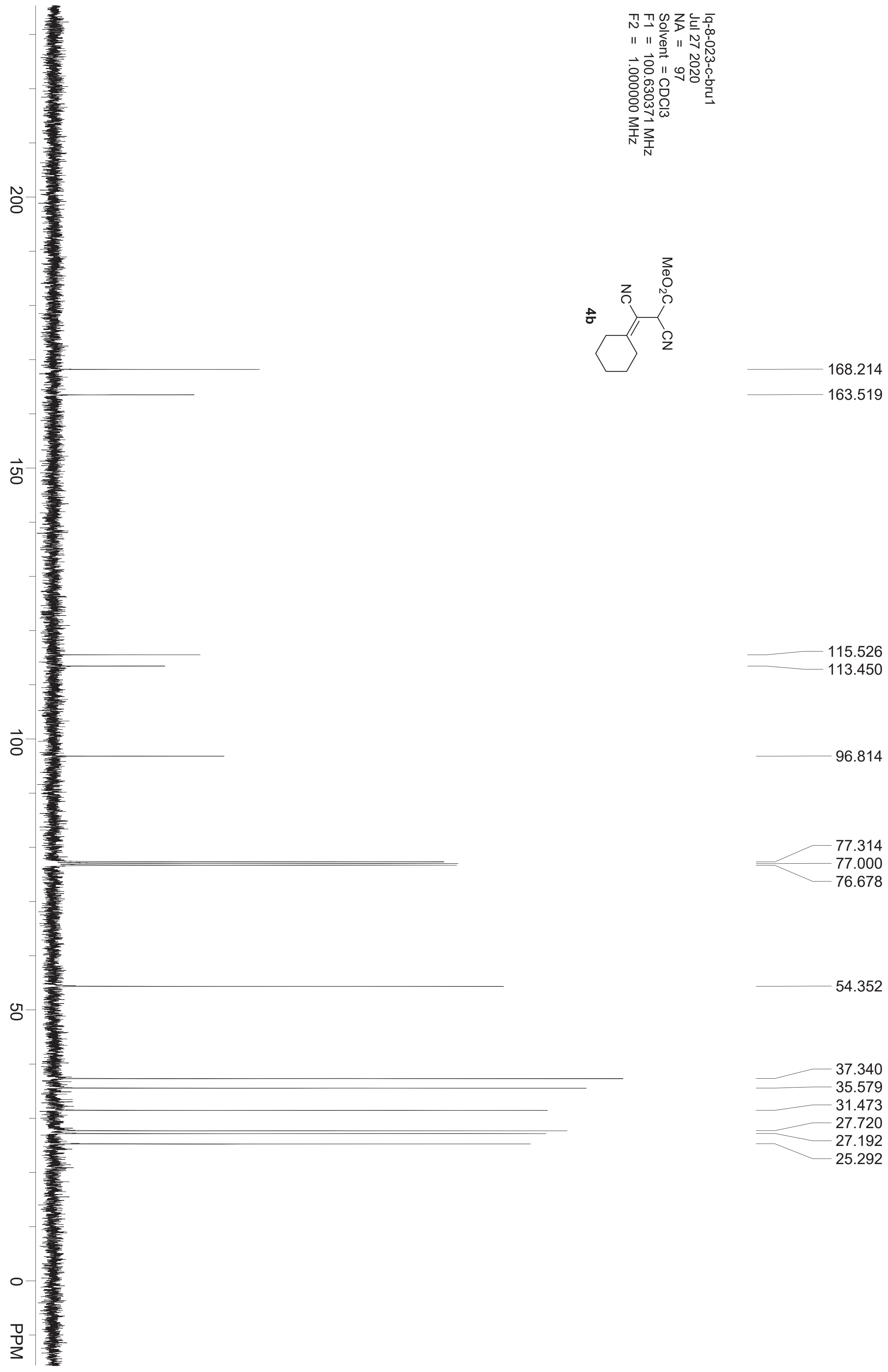

Supplementary Figure 144. ^{13}C NMR (100 MHz, $CDCl_3$) spectrum for **4b**

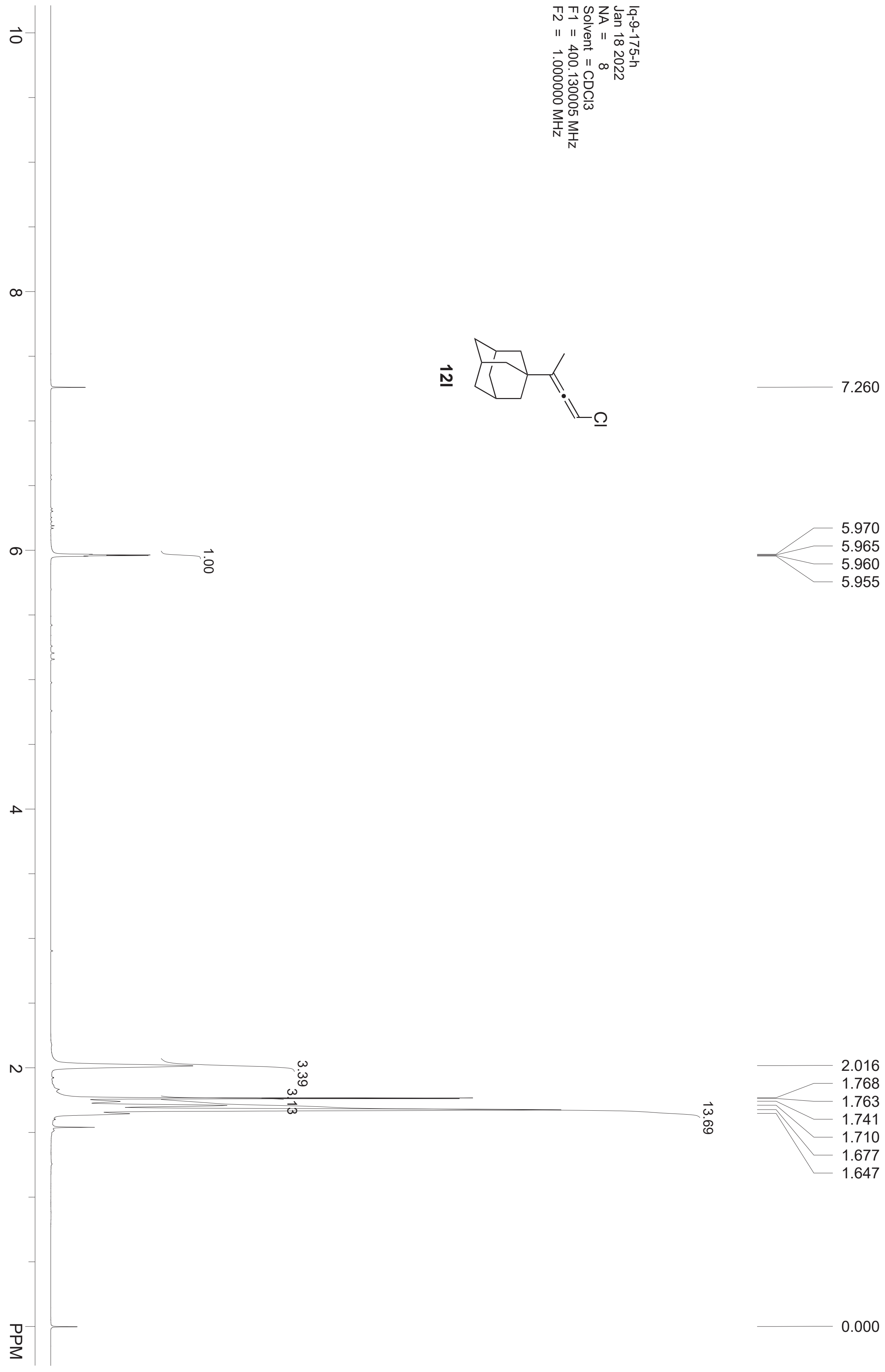

Supplementary Figure 145. ^{1}H NMR (400 MHz, $CDCl_3$) spectrum for **12l**

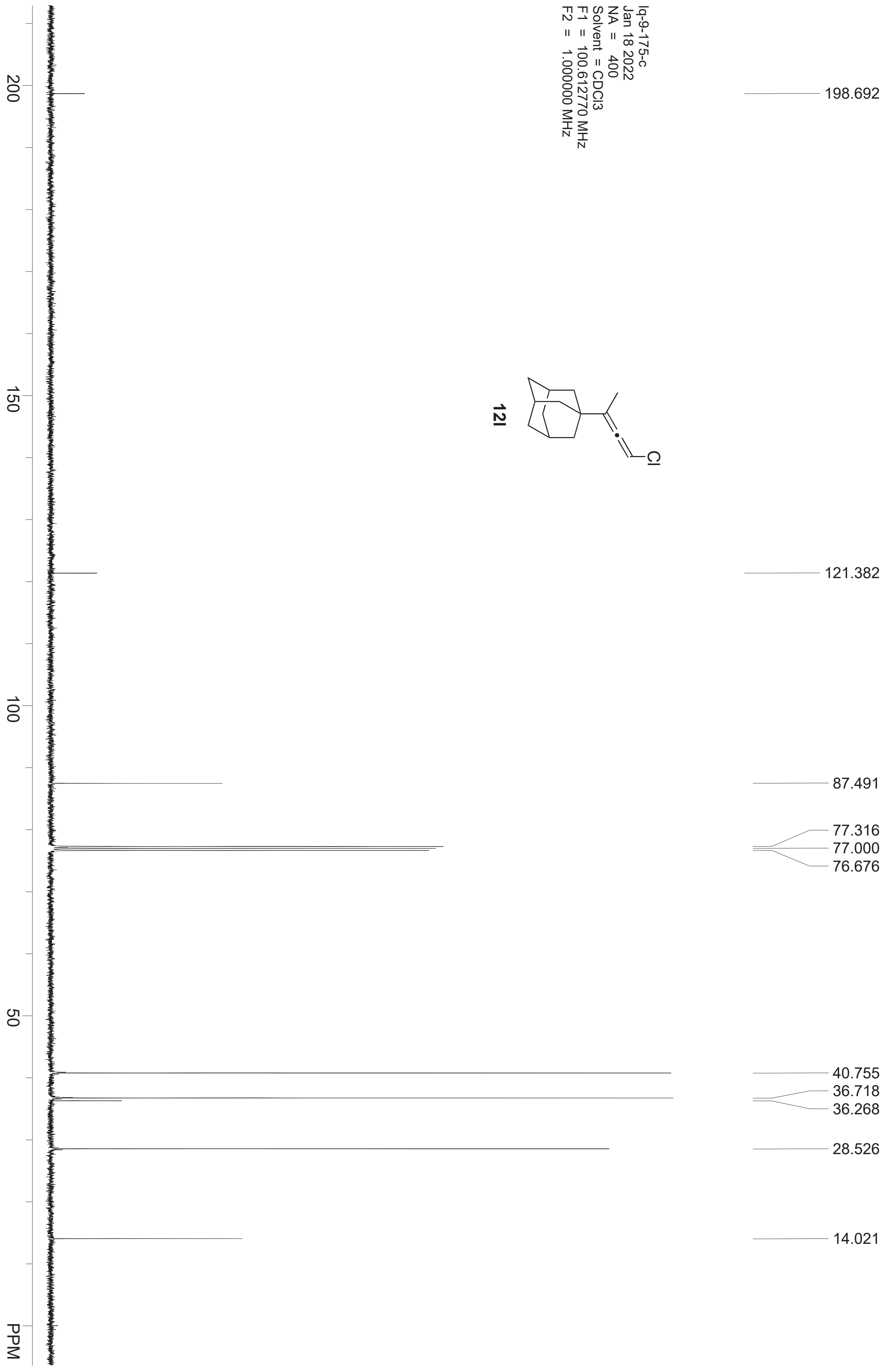

Supplementary Figure 146. ^{13}C NMR (100 MHz, $CDCl_3$) spectrum for **12l**

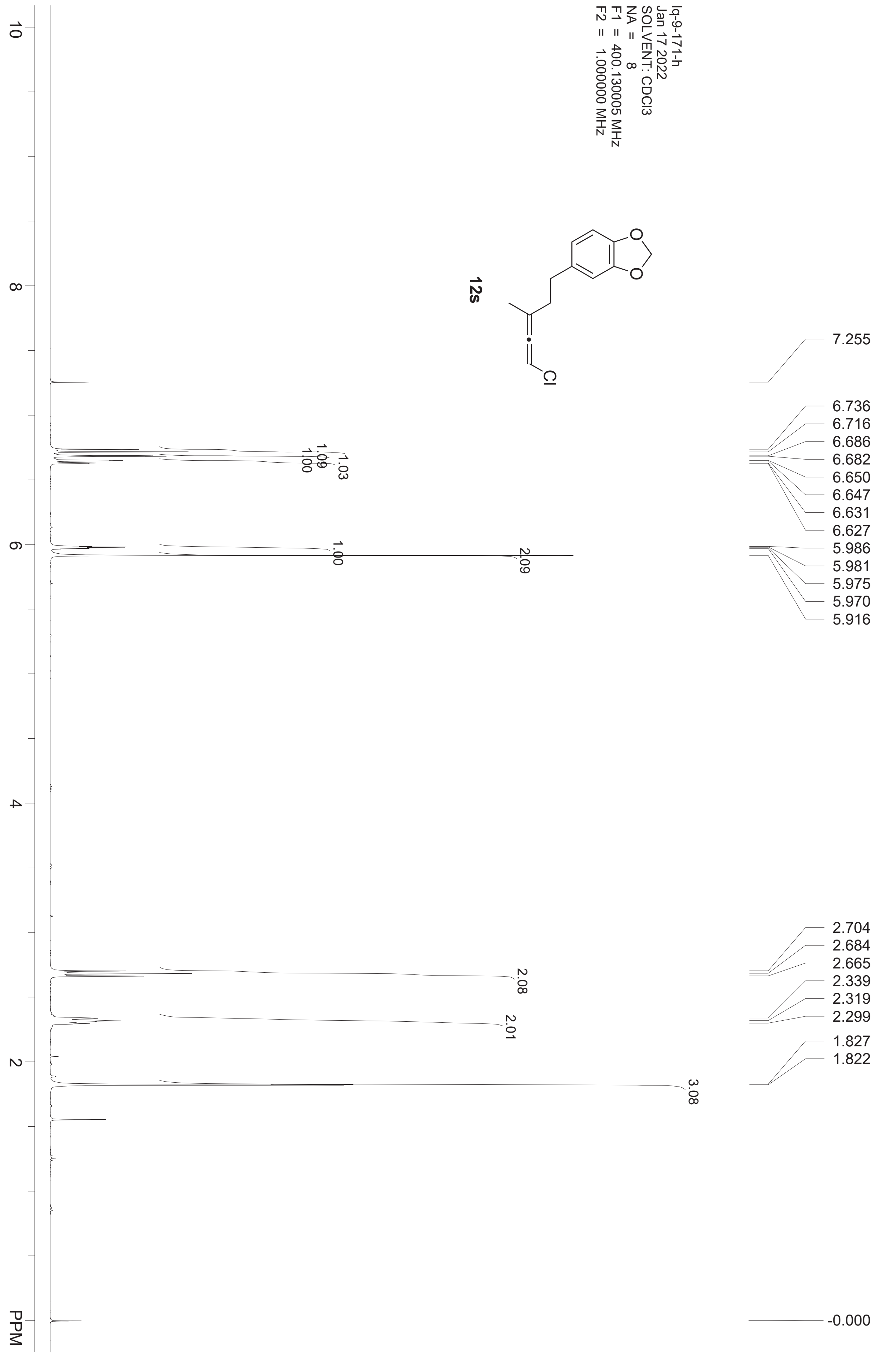

Supplementary Figure 147. ^{1}H NMR (400 MHz, $CDCl_3$) spectrum for **12s**

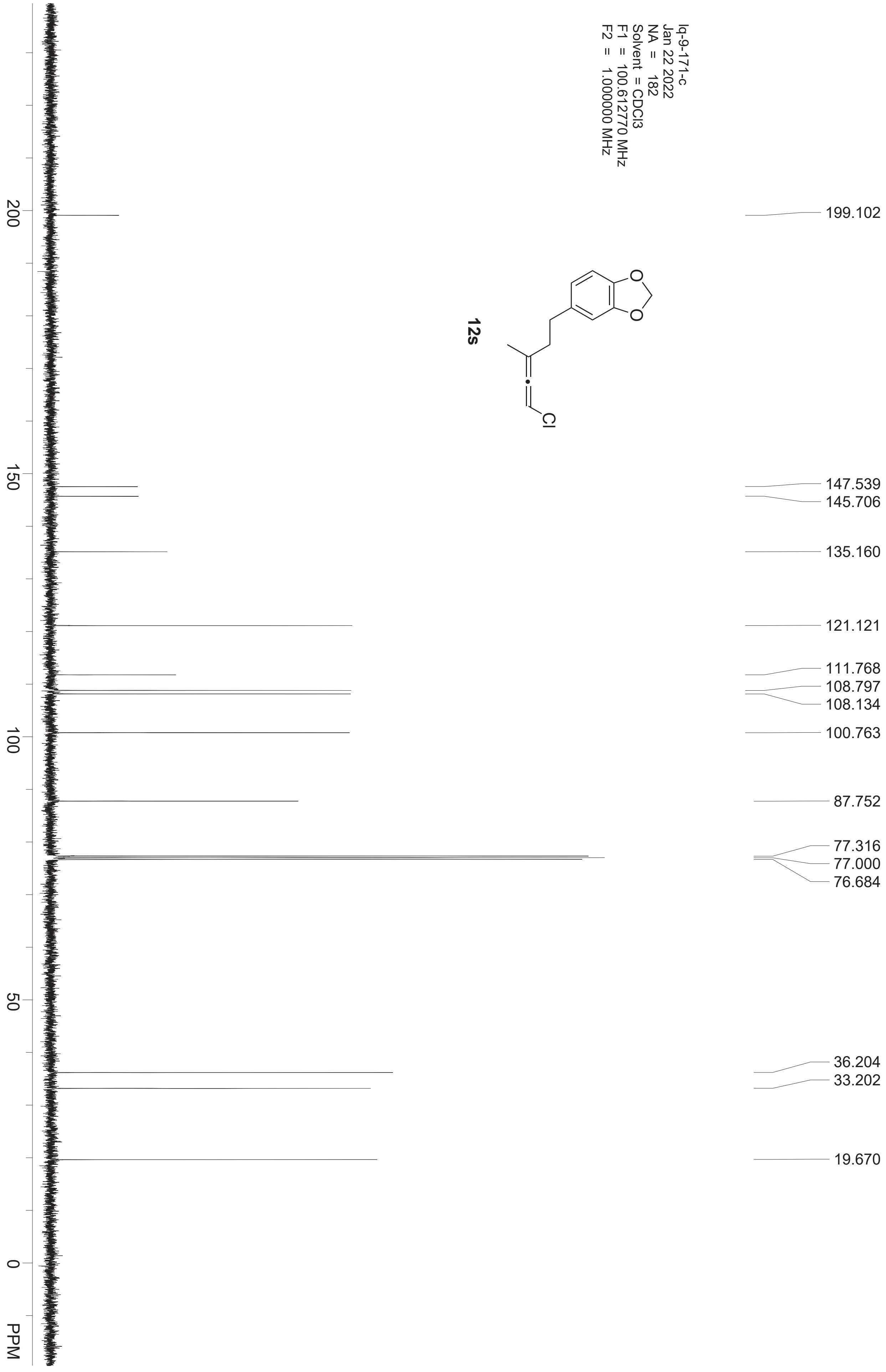

Supplementary Figure 148. ^{13}C NMR (100 MHz, $CDCl_3$) spectrum for **12s**

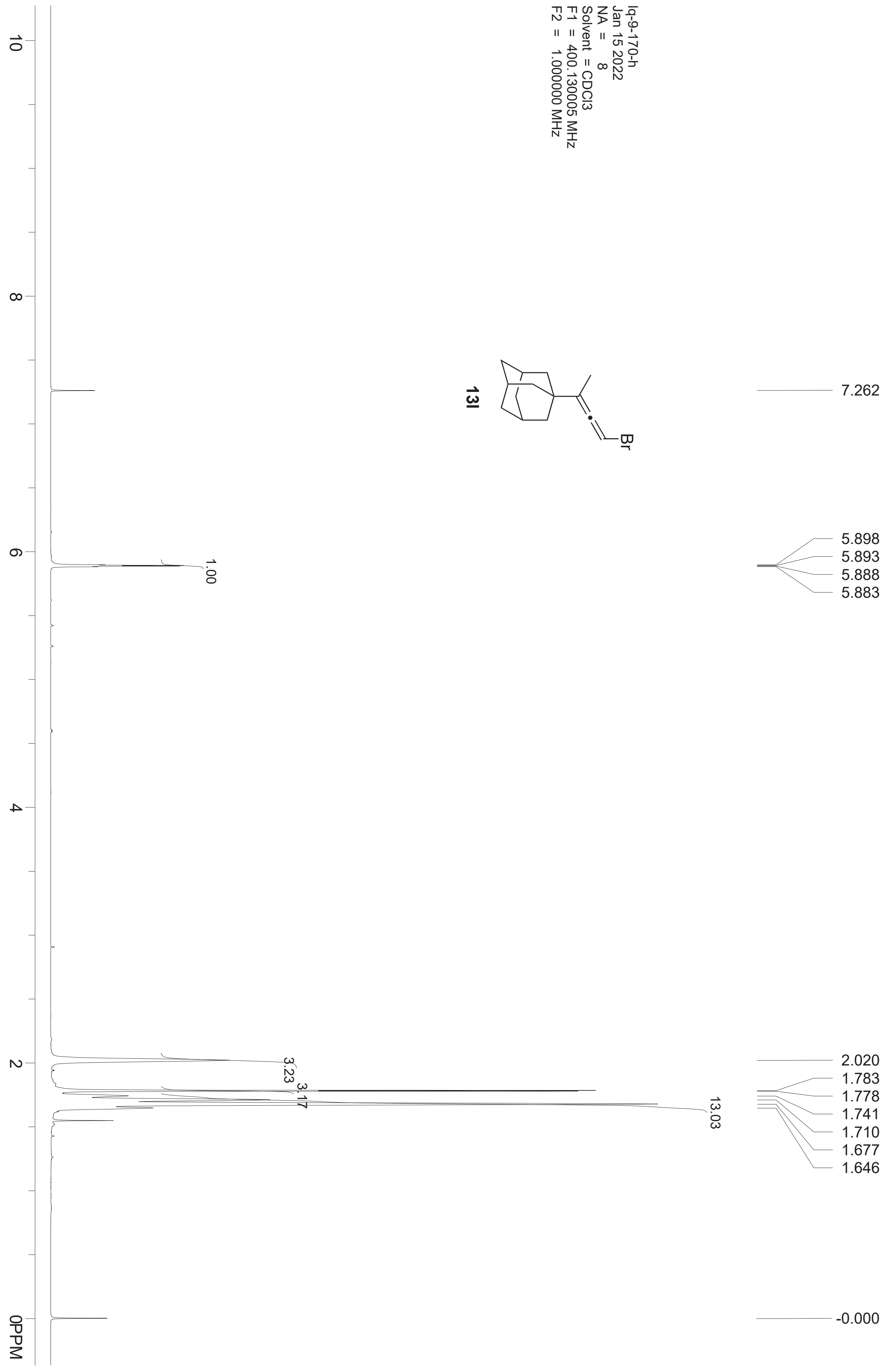

Supplementary Figure 149. ^{1}H NMR (400 MHz, $CDCl_3$) spectrum for **13l**

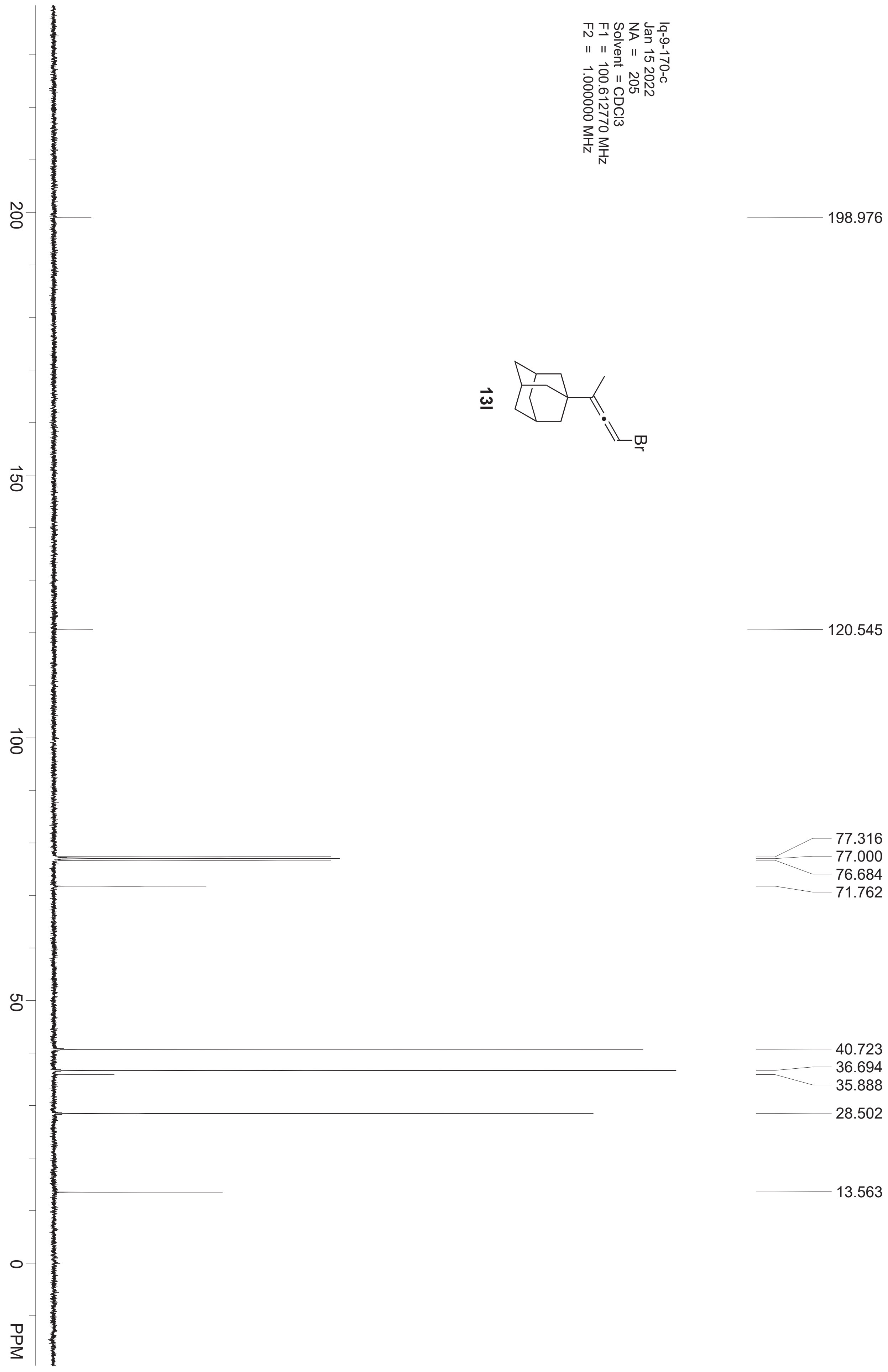

Supplementary Figure 150. ^{13}C NMR (100 MHz, $CDCl_3$) spectrum for **13l**

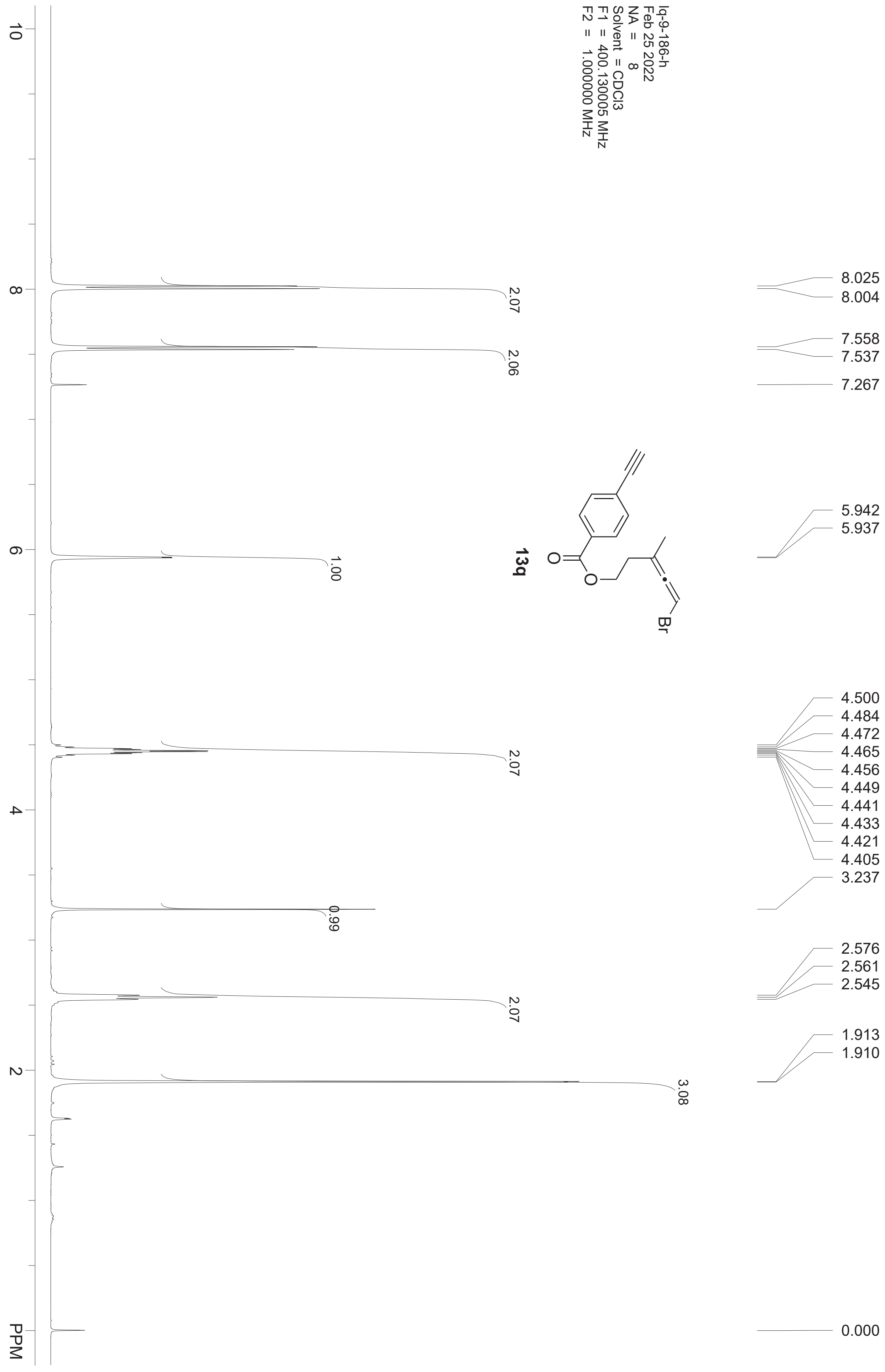

Supplementary Figure 151. ^{1}H NMR (400 MHz, $CDCl_3$) spectrum for **13q**

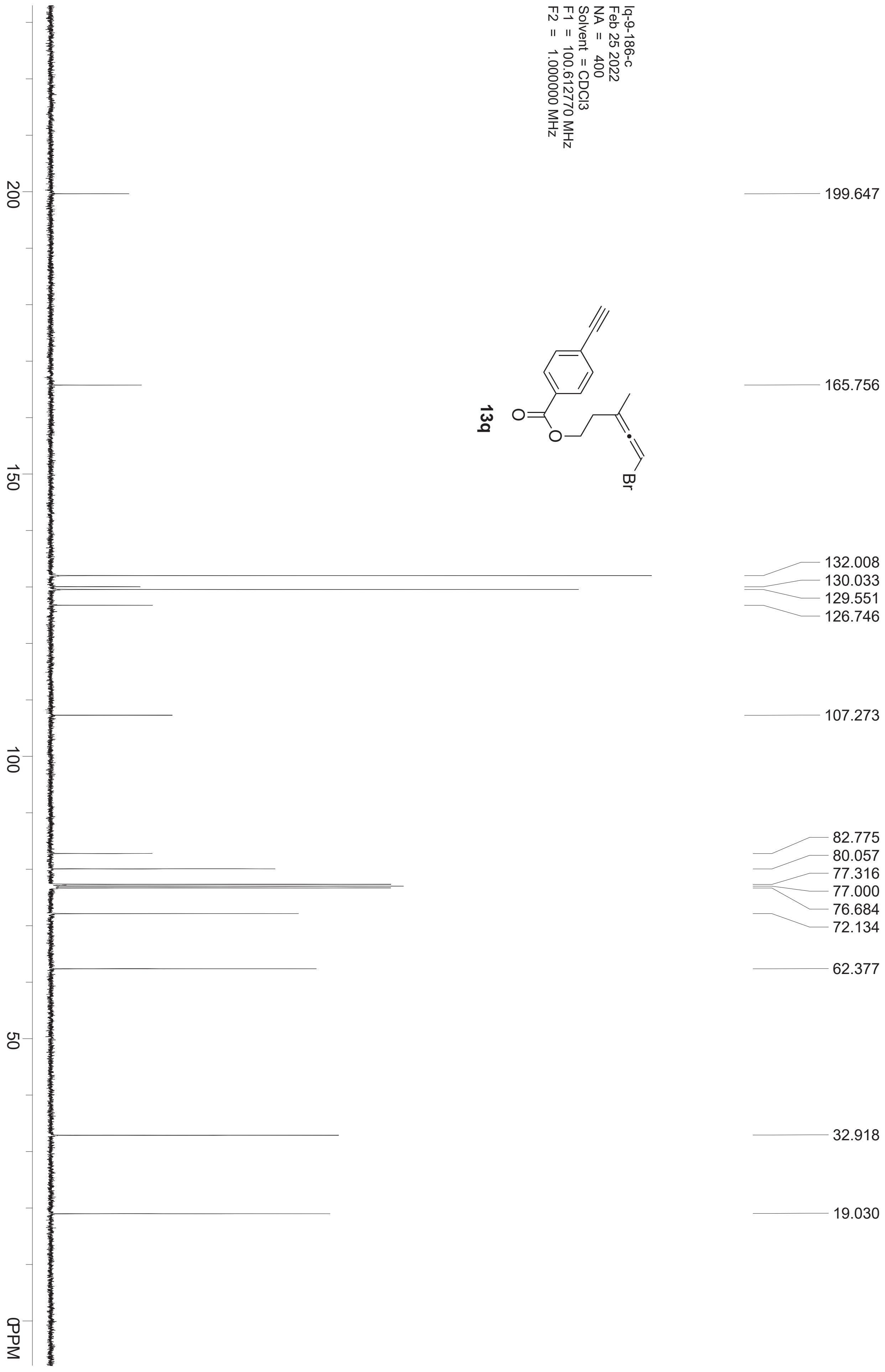

Supplementary Figure 152. ^{13}C NMR (100 MHz, $CDCl_3$) spectrum for **13q**

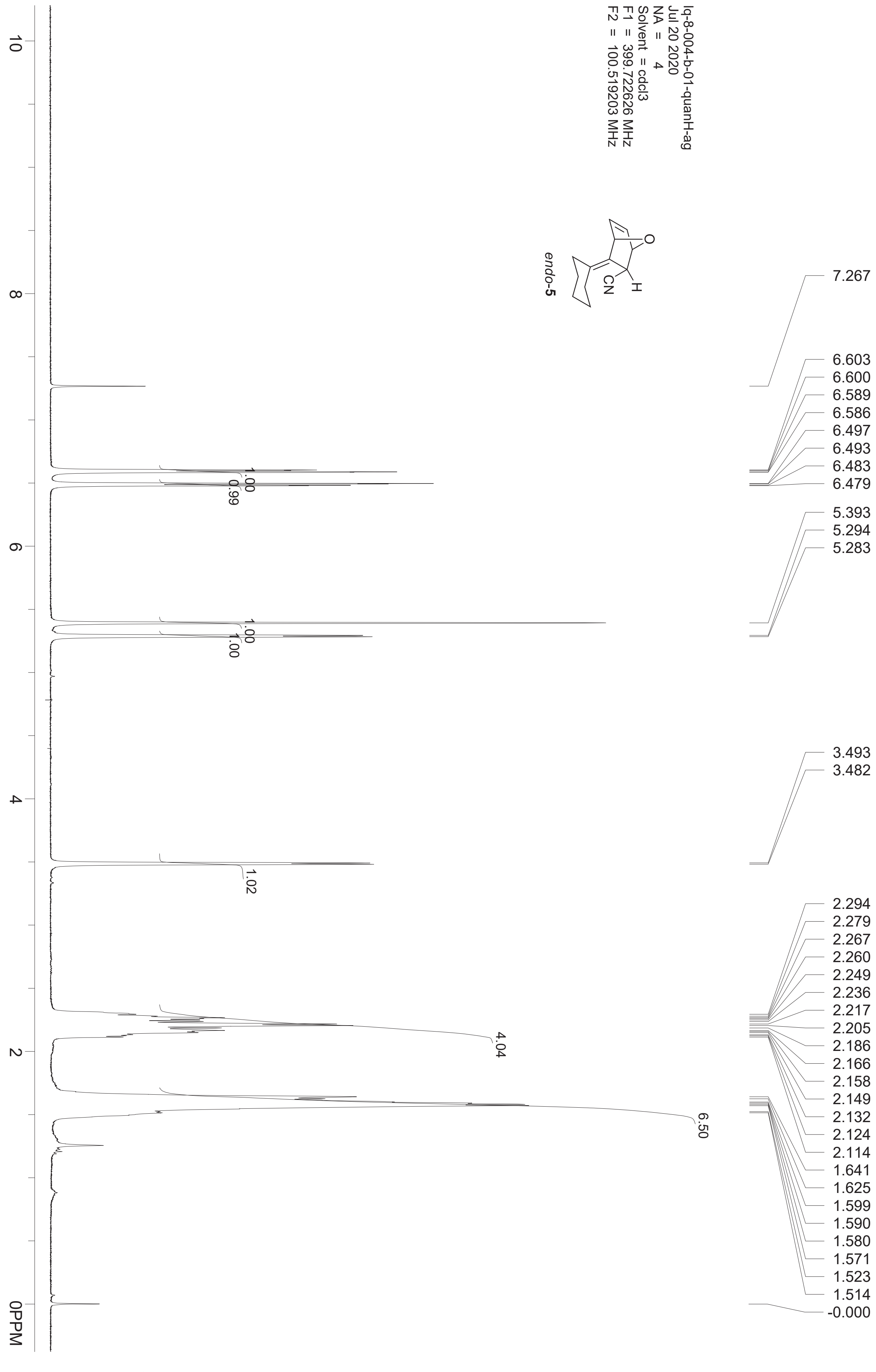

Supplementary Figure 153. ^{1}H NMR (400 MHz, $CDCl_3$) spectrum for *endo*-**5**

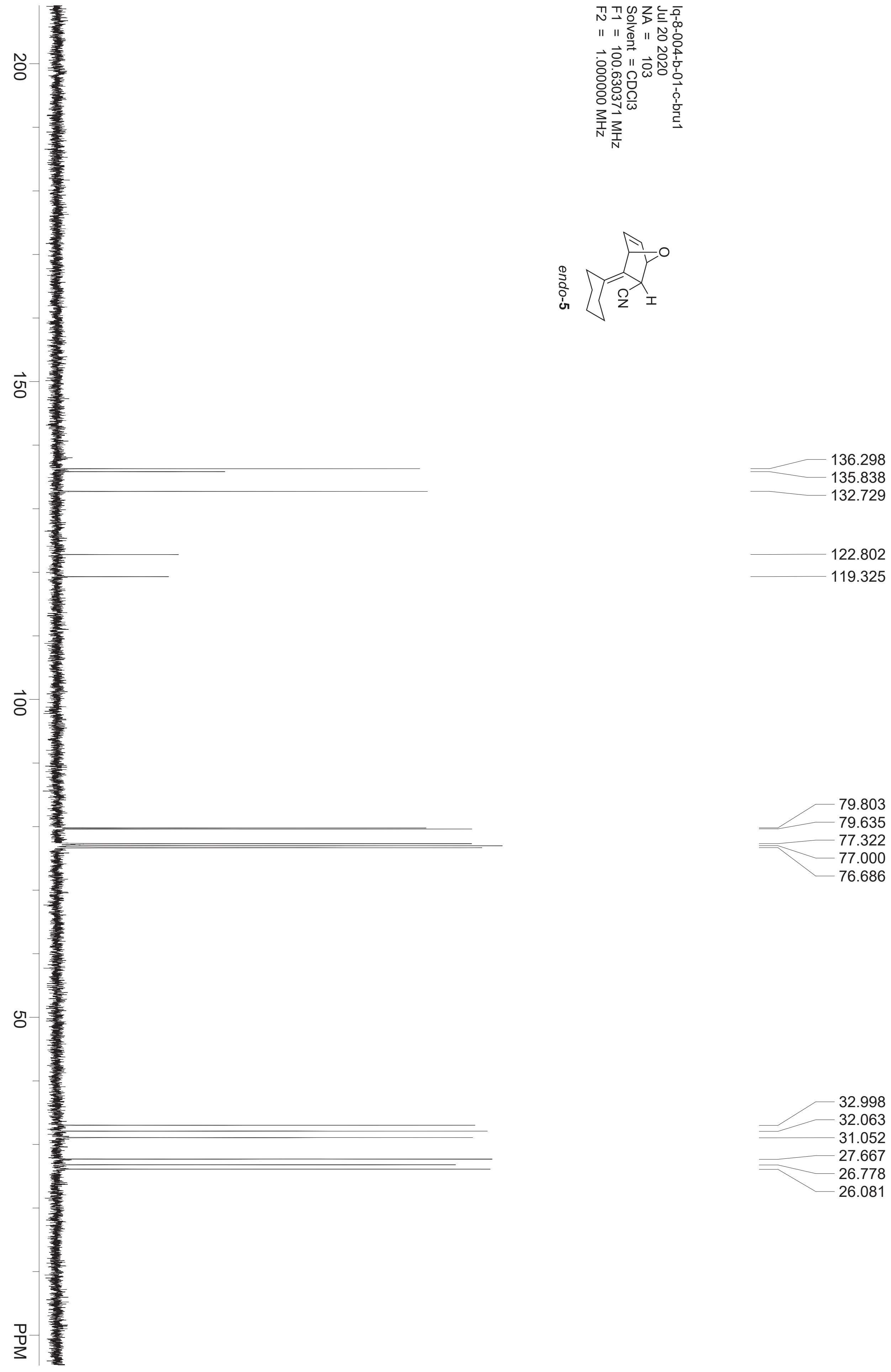

Supplementary Figure 154. ^{13}C NMR (100 MHz, $CDCl_3$) spectrum for *endo*-**5**

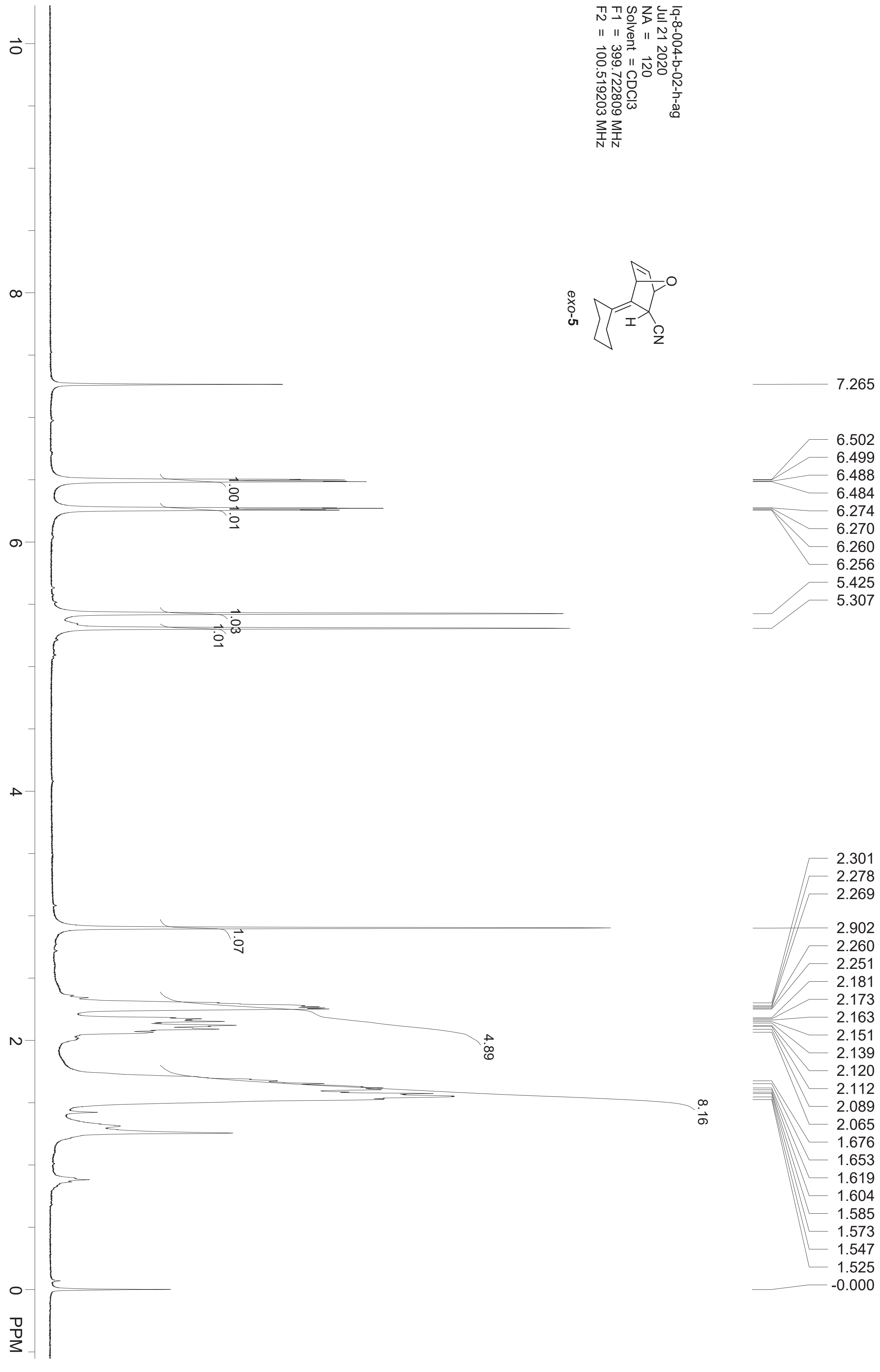

Supplementary Figure 155. ^{1}H NMR (400 MHz, $CDCl_3$) spectrum for *exo*-**5**

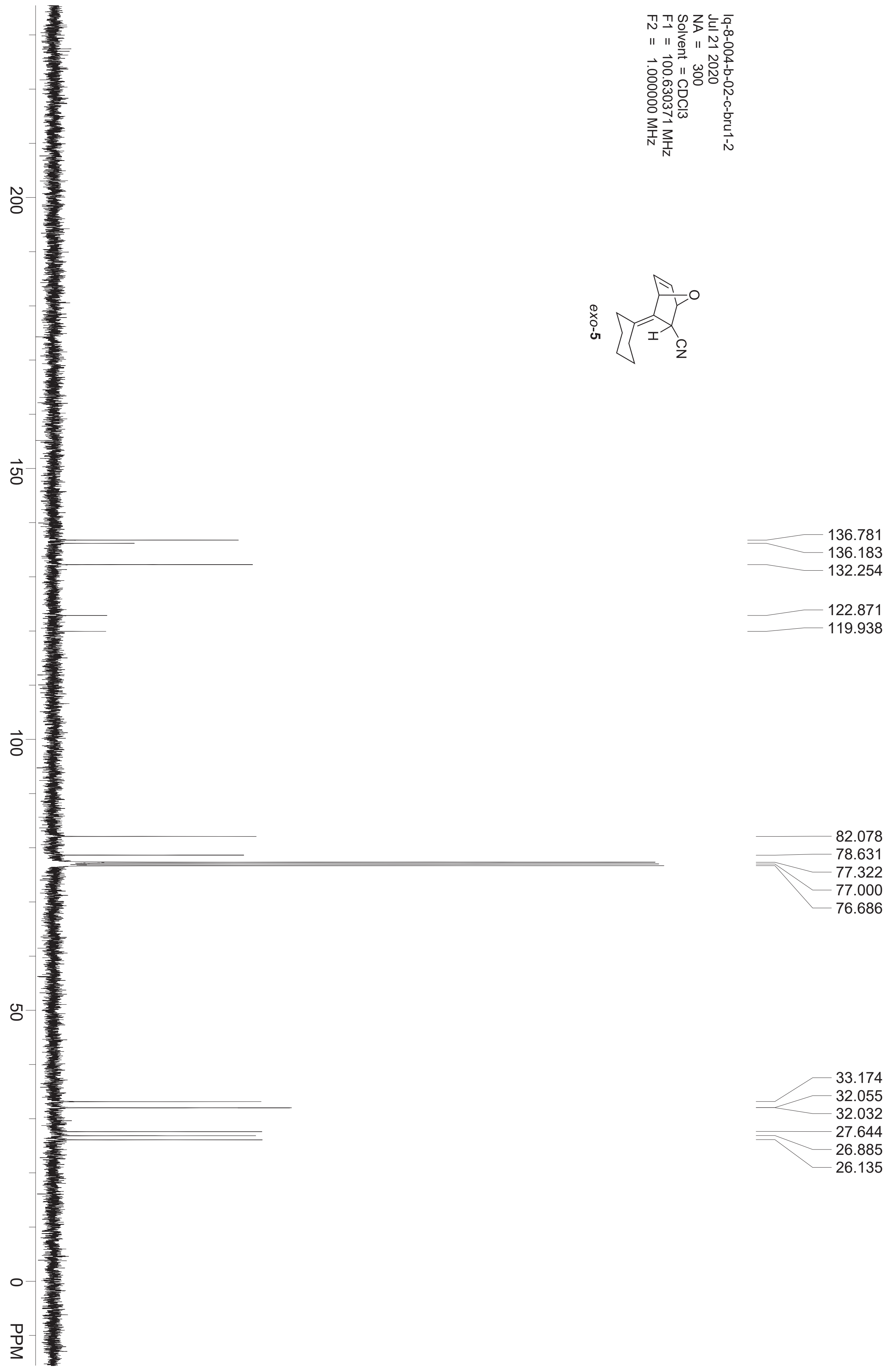

Supplementary Figure 156. ^{13}C NMR (100 MHz, $CDCl_3$) spectrum for *exo*-**5**

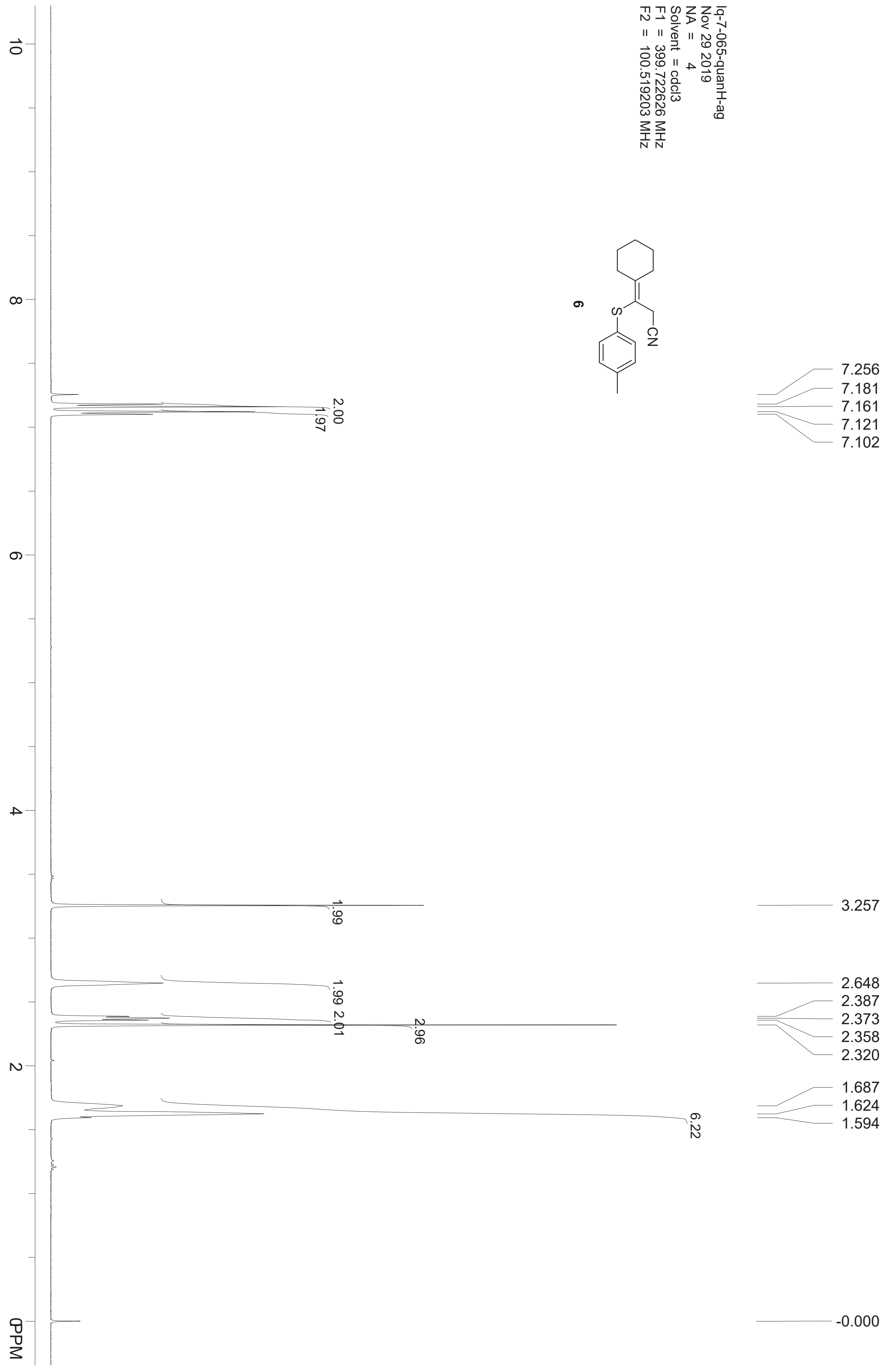

Supplementary Figure 157. ^{1}H NMR (400 MHz, $CDCl_3$) spectrum for **6**

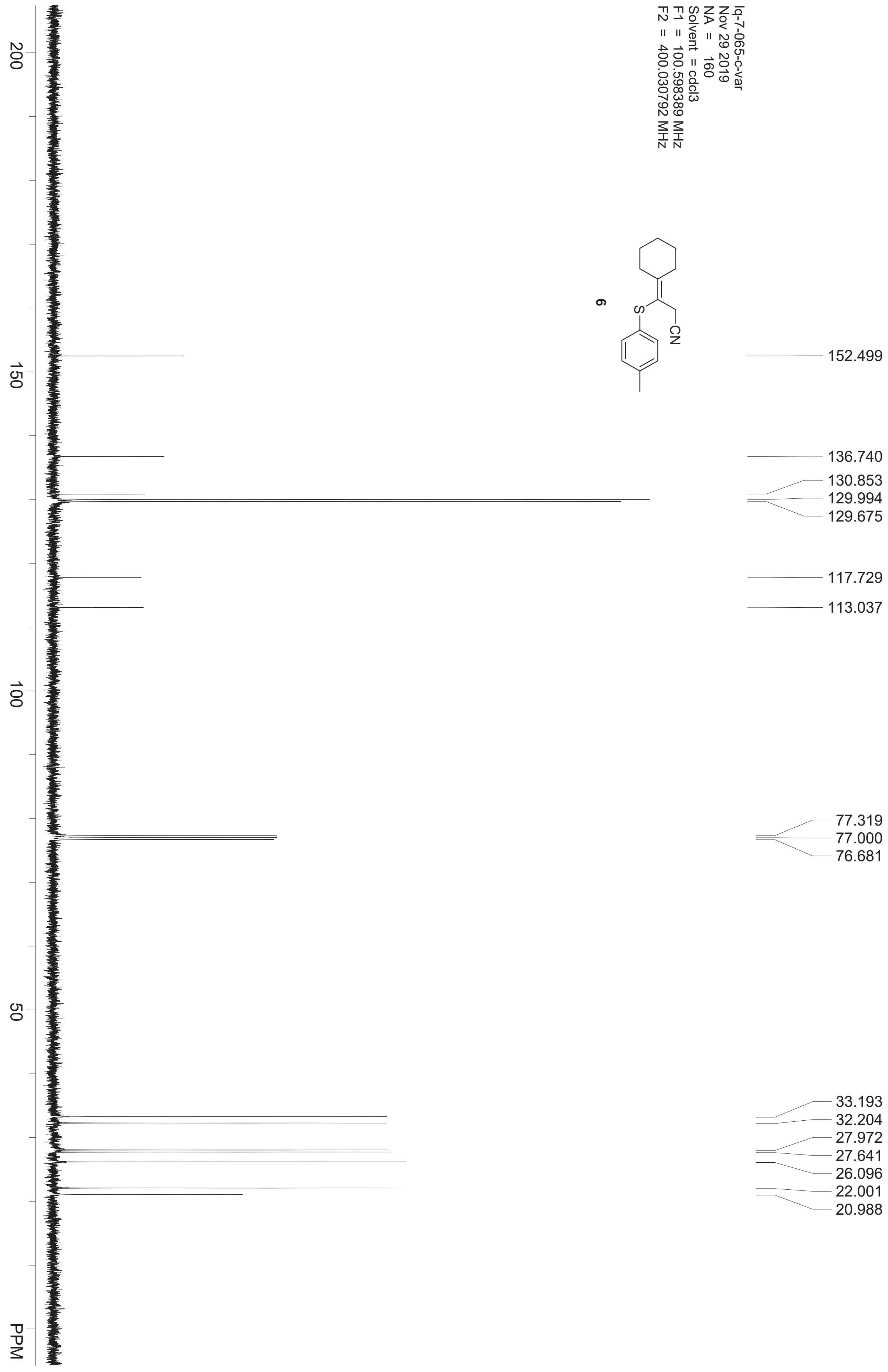

Supplementary Figure 158. ^{13}C NMR (100 MHz, $CDCl_3$) spectrum for **6**

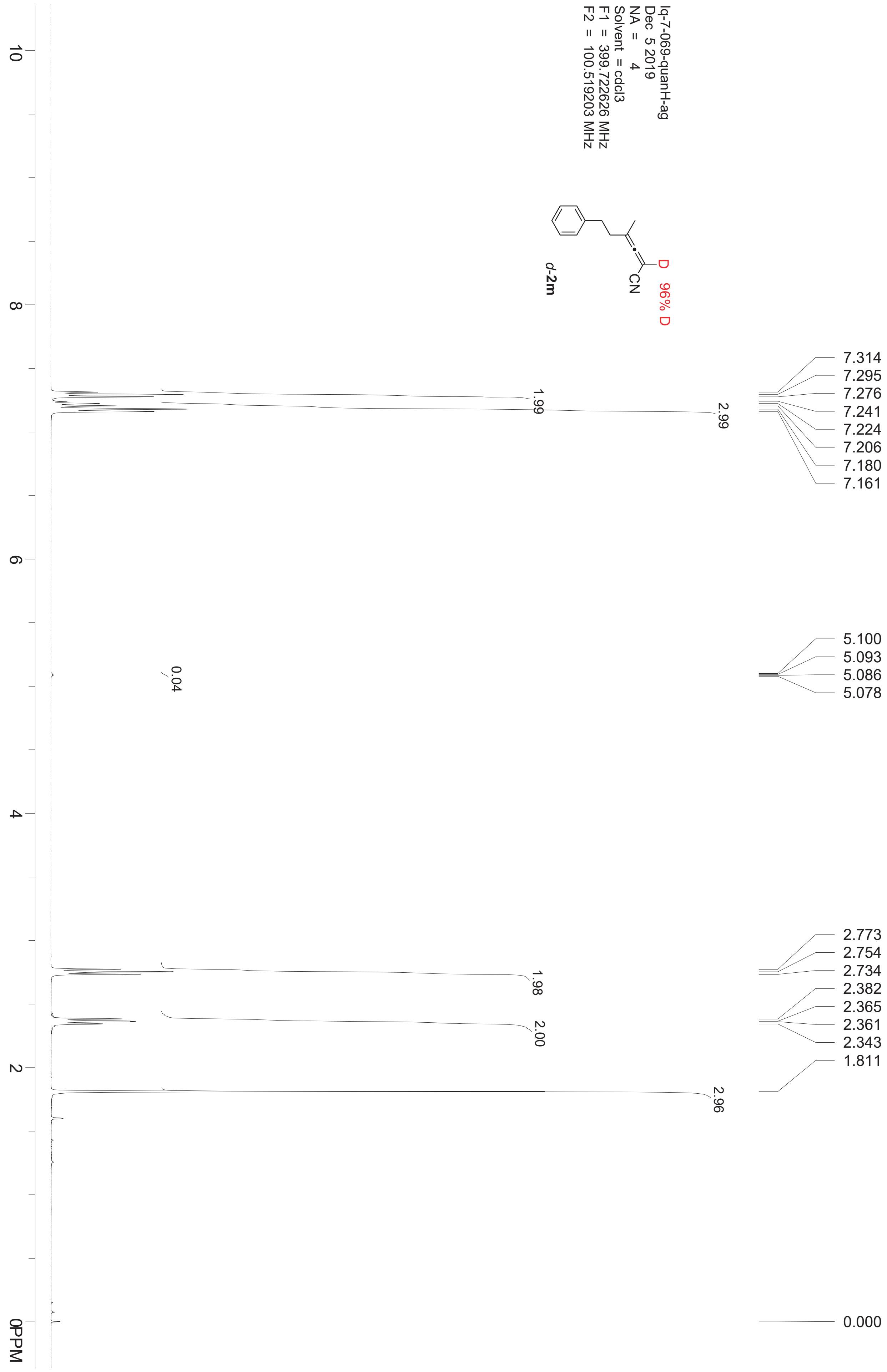

Supplementary Figure 159. ^{1}H NMR (400 MHz, $CDCl_3$) spectrum for *d*-**2m**

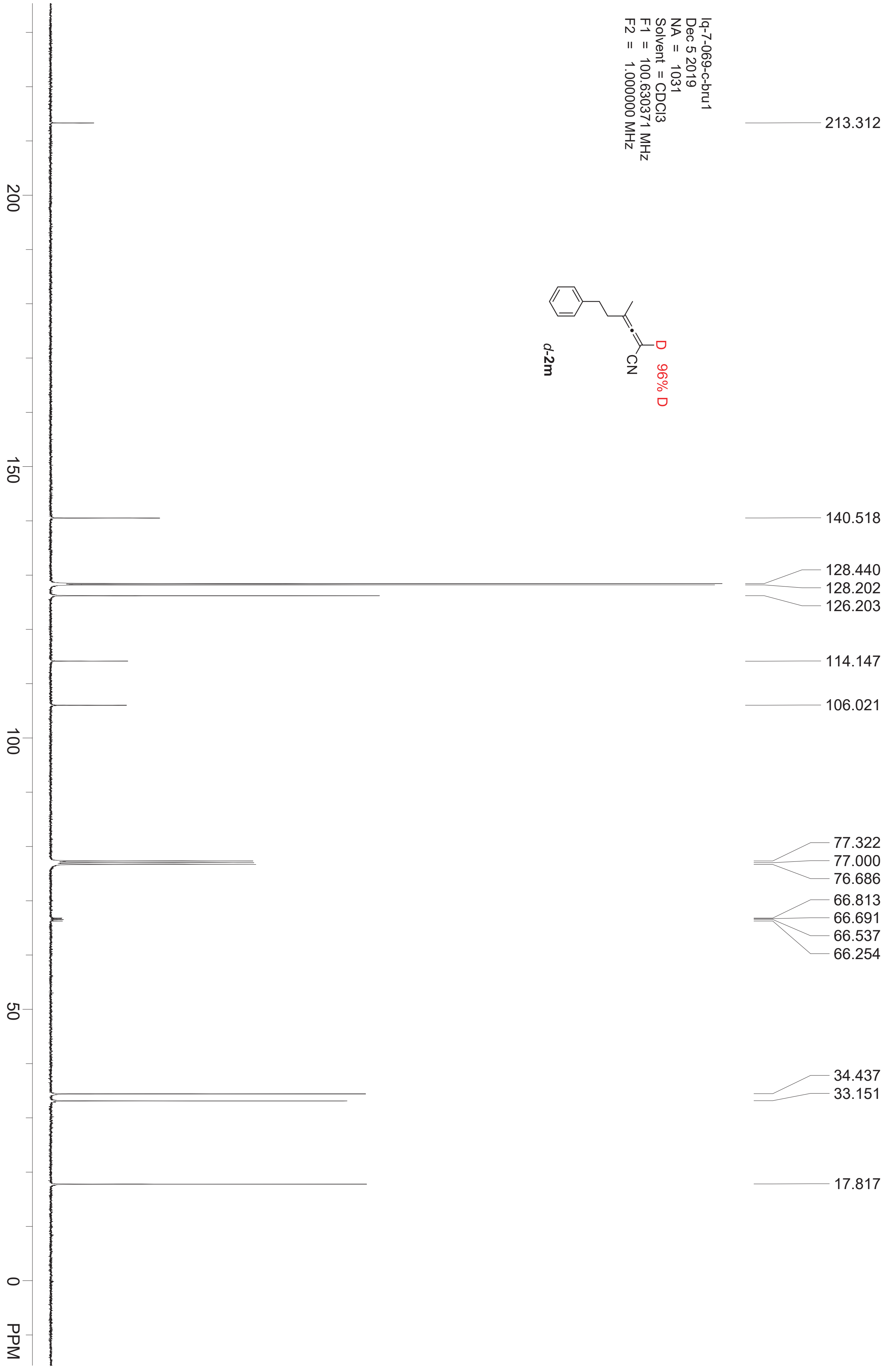

Supplementary Figure 160. ^{13}C NMR (100 MHz, $CDCl_3$) spectrum for *d*-**2m**

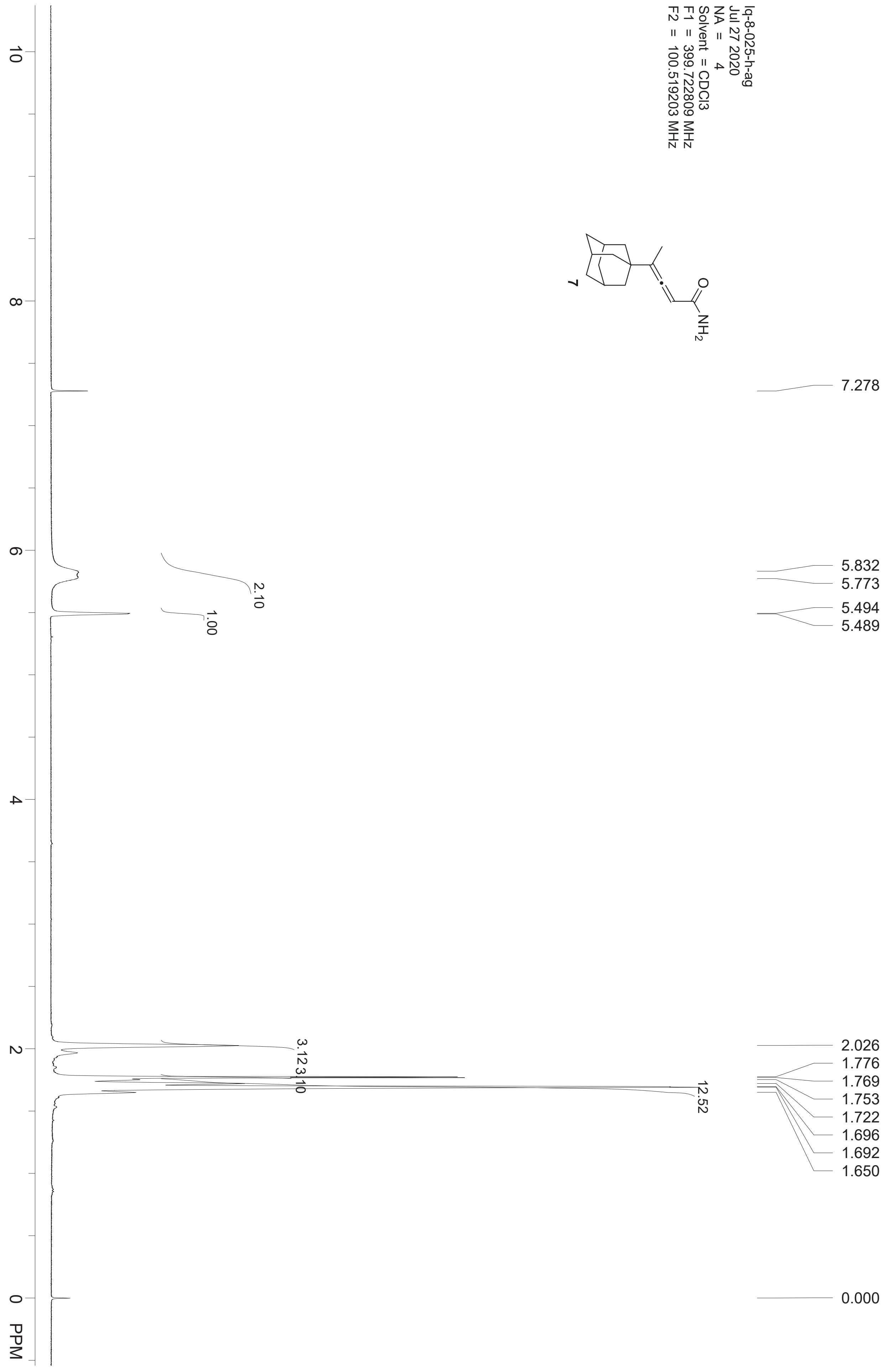

Supplementary Figure 161. ^{1}H NMR (400 MHz, $CDCl_3$) spectrum for **7**

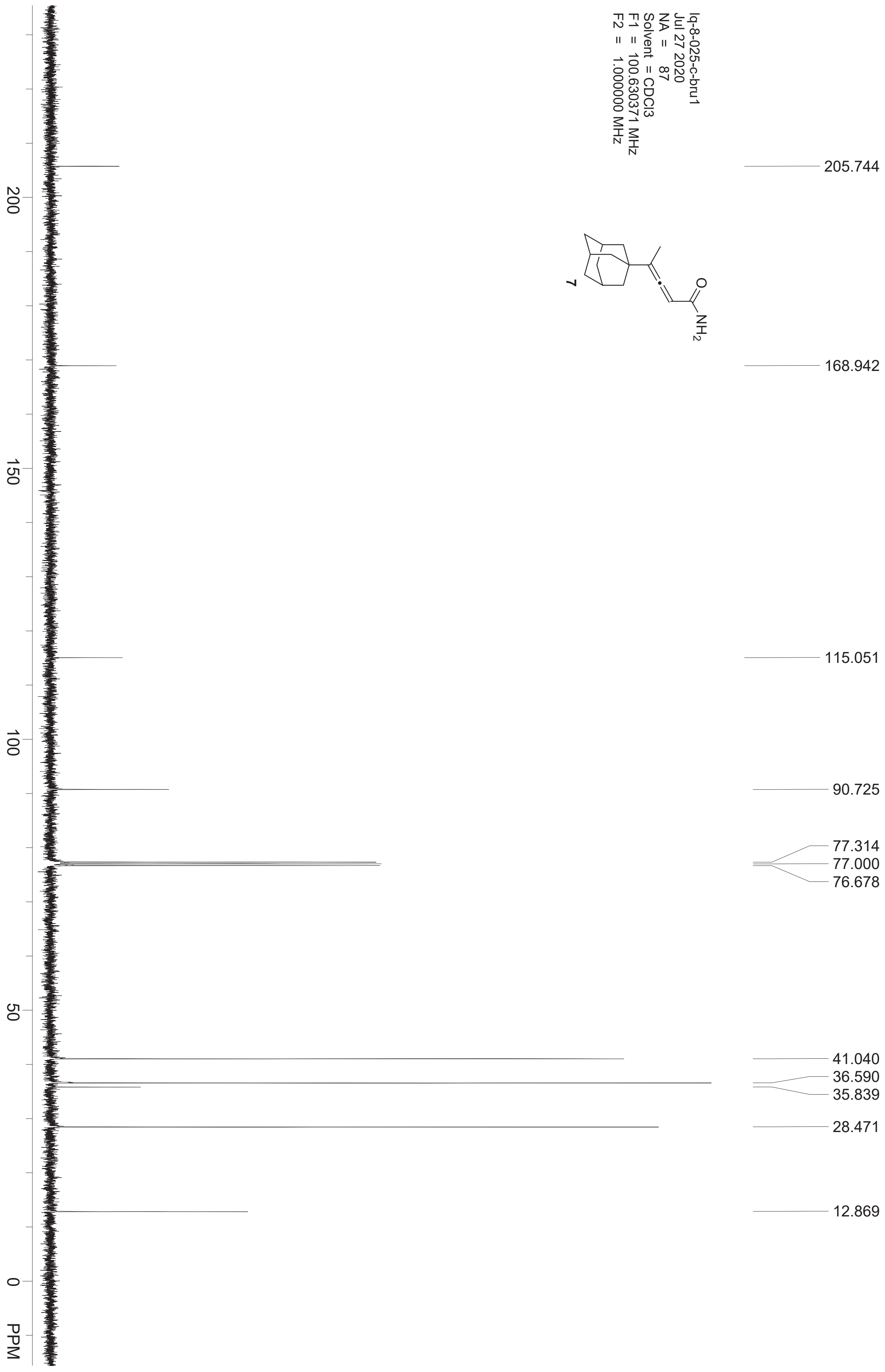

Supplementary Figure 162. ^{13}C NMR (100 MHz, $CDCl_3$) spectrum for **7**

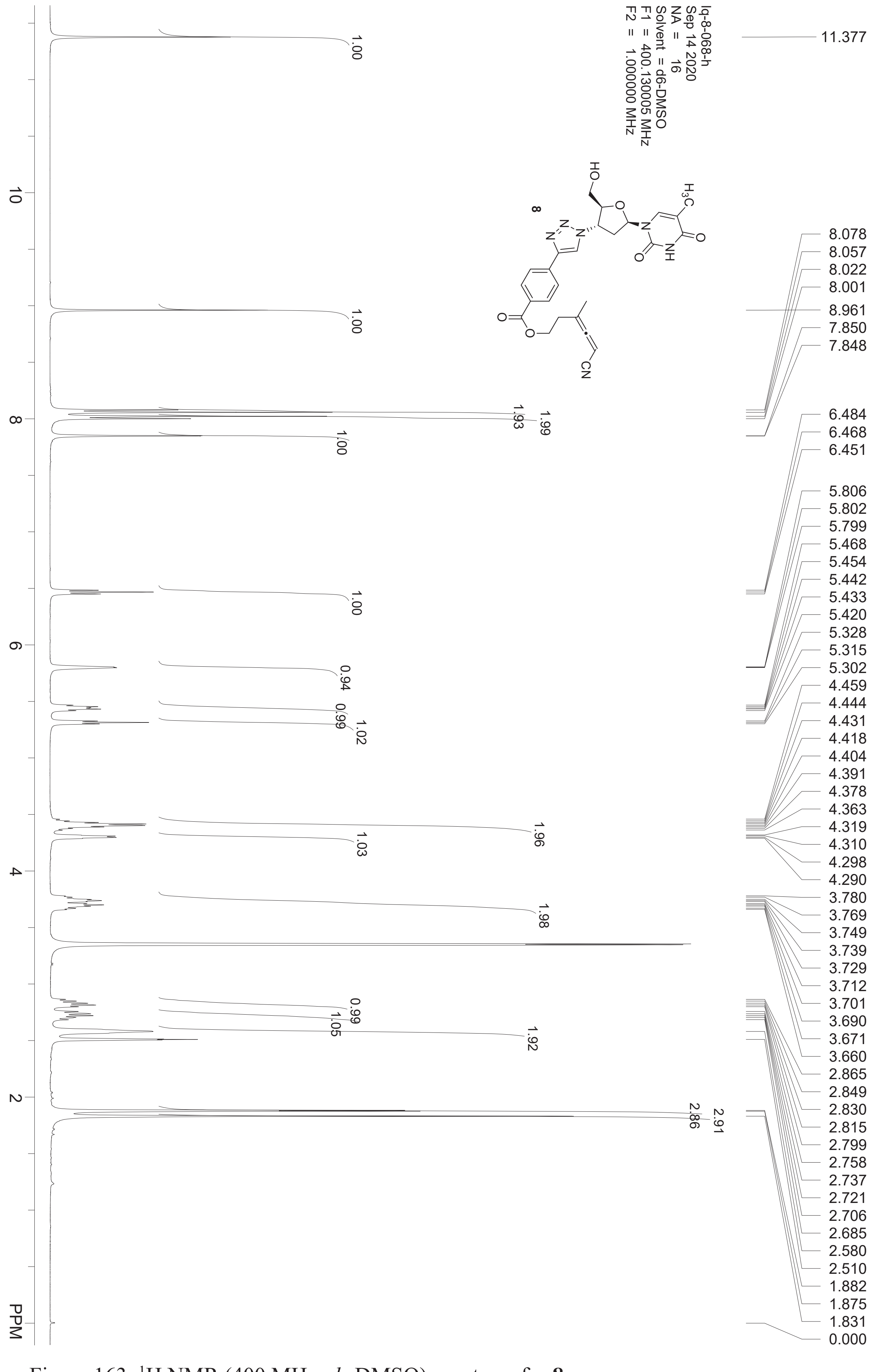

Supplementary Figure 163. ^{1}H NMR (400 MHz, d_6-DMSO) spectrum for **8**

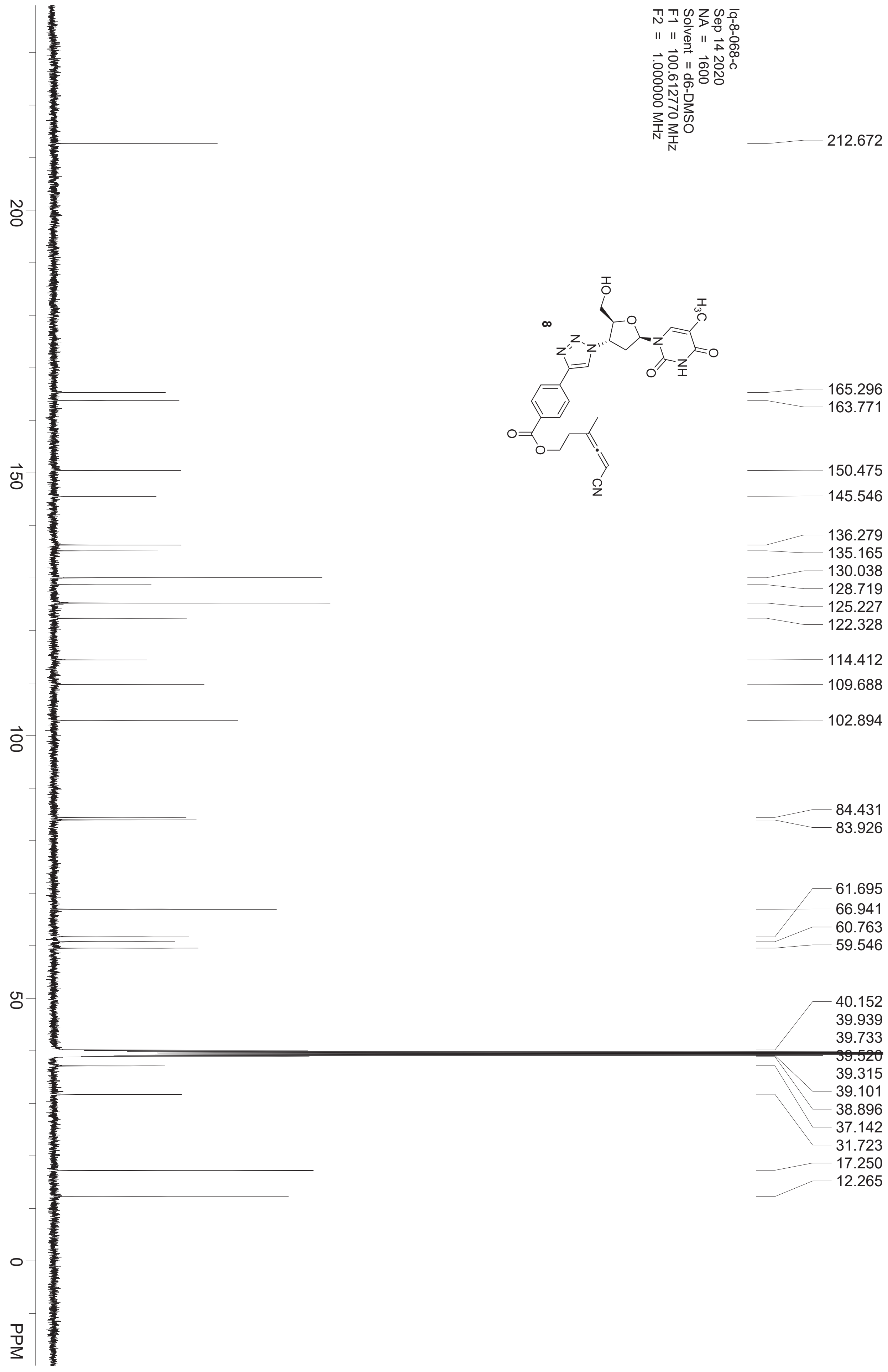

Supplementary Figure 164. ^{13}C NMR (100 MHz, d_6-DMSO) spectrum for **8**

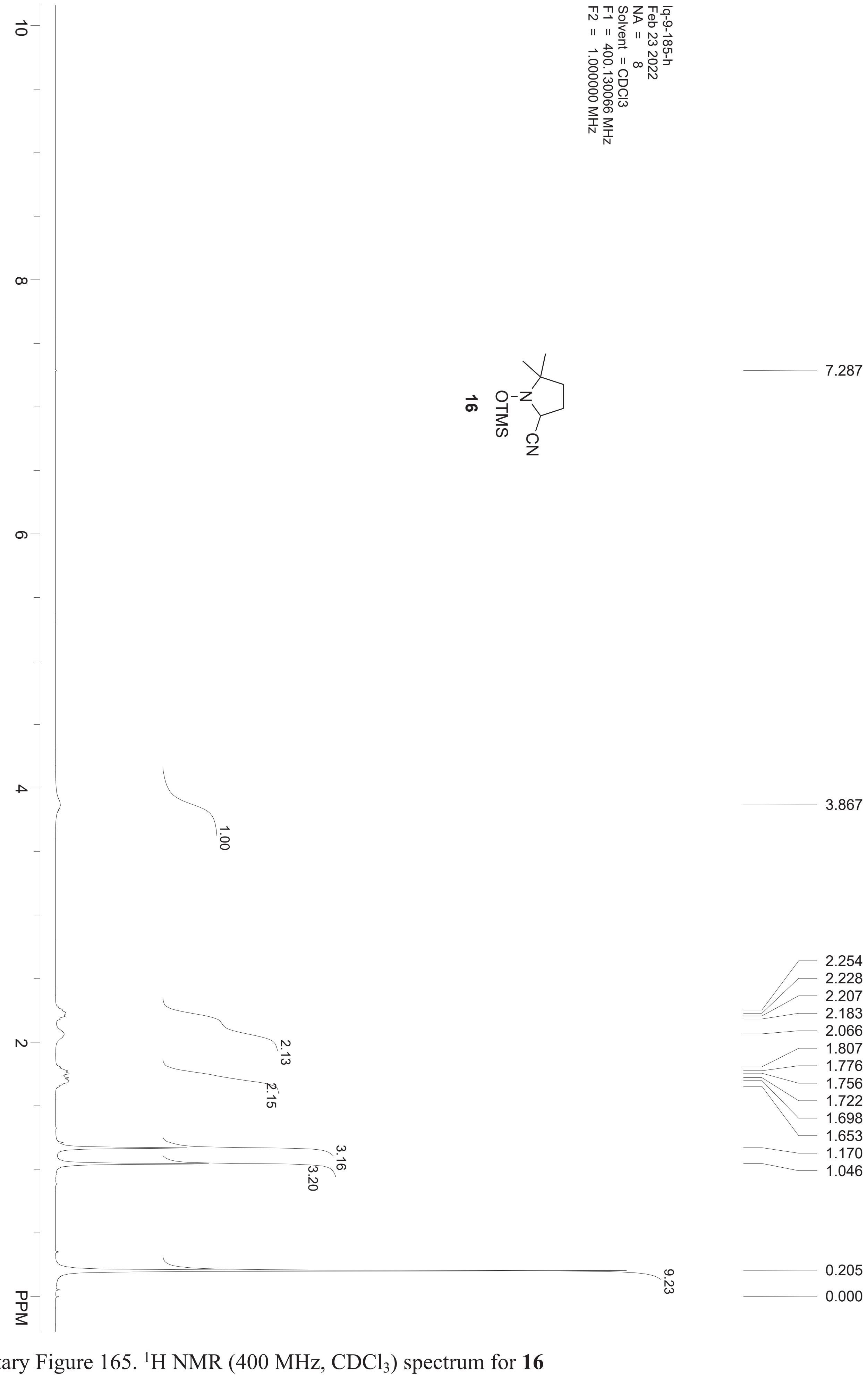

Supplementary Figure 165. ^{1}H NMR (400 MHz, $CDCl_3$) spectrum for **16**

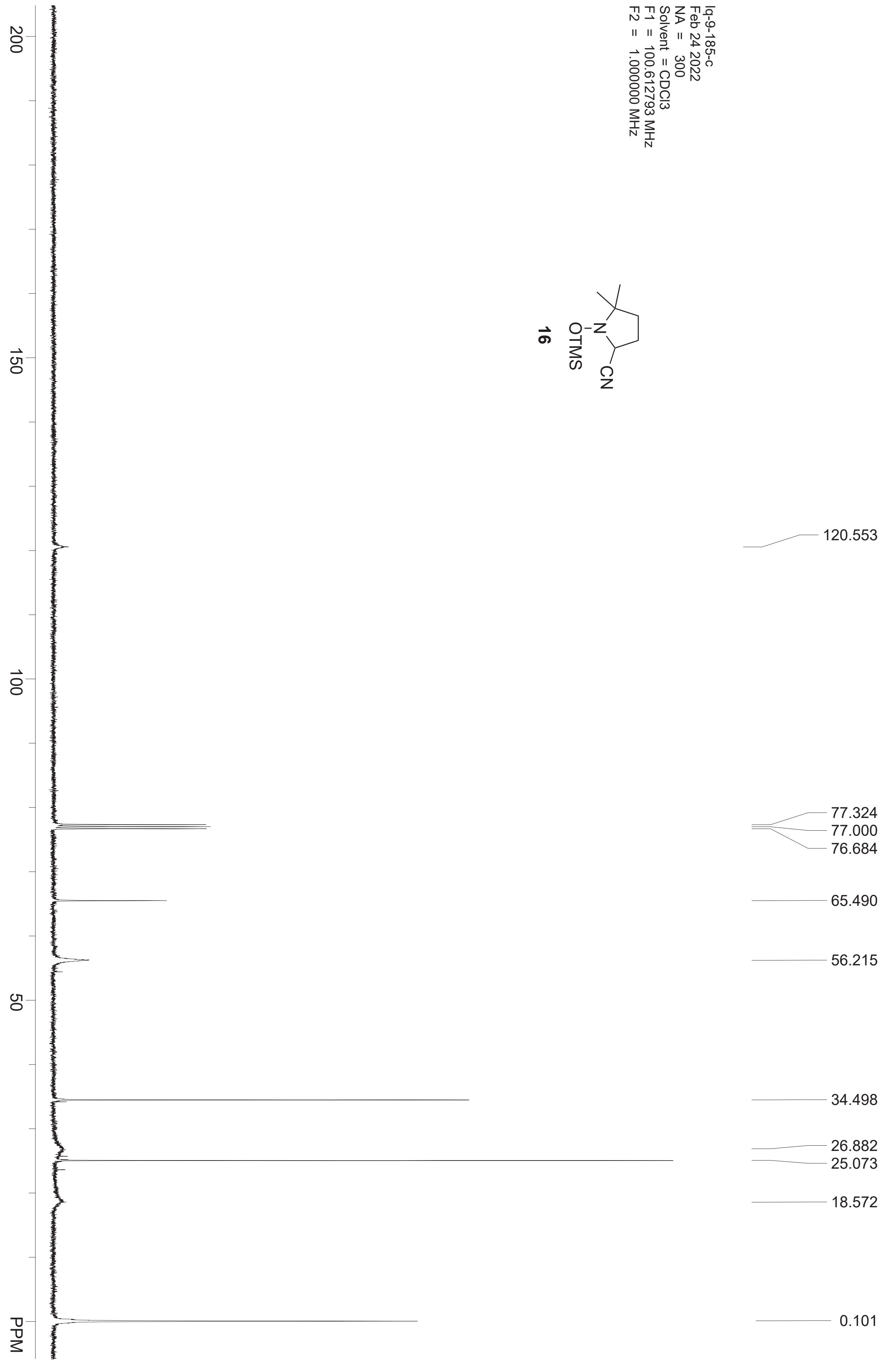

Supplementary Figure 166. ^{13}C NMR (100 MHz, $CDCl_3$) spectrum for **16**

Supplementary References

1. Gronnier, C., Boissonnat, G. & Gagosz, F. Au-catalyzed formation of functionalized quinolines from 2-alkynyl arylazide derivatives. *Org. Lett.* **15**, 4234-4237 (2013).
2. Chenna Reddy, M. L., Patil, V. B., Nawaz Khan, F. R. & Saravanan, V. Synthesis of imidazo[1,2-*a*]pyridines and imidazo[2,1-*b*]thiazoles attached to a cycloalkyl or saturated heterocycle containing a tertiary hydroxy substitution. *Journal of Heterocyclic Chemistry* **56**, 1486-1497 (2019).
3. Gronnier, C., Kramer, S., Odabachian, Y. & Gagosz, F. Cu(i)-catalyzed oxidative cyclization of alkynyl oxiranes and oxetanes. *J. Am. Chem. Soc.* **134**, 828-831 (2012).
4. Hiller, C. *et al.* Functionally selective dopamine D_2/D_3 receptor agonists comprising an enyne moiety. *J. Med. Chem.* **56**, 5130-5141 (2013).
5. Chiarucci, M. et al. Merging synthesis and enantioselective functionalization of indoles by a gold-catalyzed asymmetric cascade reaction. *Angew. Chem. Int. Ed*. **52**, 10850-10853 (2013).
6. Trost, B. M. & Rudd, M. T. An unusual ruthenium-catalyzed dimerization of propargyl alcohols. *J. Am. Chem. Soc.* **123**, 8862-8863 (2001).
7. Ge, Y. *et al.* Ligand-controlled palladium-catalyzed carbonylation of alkynols: Highly selective synthesis of alpha-methylene-beta-lactones. *Angew. Chem. Int. Ed.* **59**, 21585-21590 (2020).
8. Cheng, X., Jiang, X., Yu, Y. & Ma, S. Efficient synthesis of 3-chloromethyl-2(5H)-furanones and 3-chloromethyl-5,6-dihydropyran-2-ones via the $PdCl_2$-catalyzed chlorocyclocarbonylation of 2,3- or 3,4-allenols. *J. Org. Chem.* **73**, 8960-8965 (2008).
9. Ma, S.; Wu, B.; Jiang, X.; Zhao, S. *J. Org. Chem.* **2005**, *70*, 2568-2575. Ma, S., Wu, B., Jiang, X. & Zhao, S. Studies on Pd(II)-catalyzed synthesis of (*Z*)-α-haloalkylidene-beta-lactones from cyclocarbonylation of 2-alkynols and the subsequent coupling reactions. *J. Org. Chem.* **70**, 2568-2575 (2005).
10. Shibata, Y., Noguchi, K. & Tanaka, K. Cationic rhodium(I) complex-catalyzed [3 +

2] and [2 + 1] cycloadditions of propargyl esters with electron-deficient alkynes and alkenes. *J. Am. Chem. Soc.* **132**, 7896-7898 (2010).

11. Tsuji, Y., Taniguchi, M., Yasuda, T., Kawamura, T. & Obora, Y. Palladium-catalyzed cyanation of propargylic carbonates with trimethylsilyl cyanide. *Org. Lett.* **2**, 2635-2637 (2000).
12. Ao, Y. F., Wang, D. X., Zhao, L. & Wang, M. X. Biotransformations of racemic 2,3-allenenitriles in biphasic systems: Synthesis and transformations of enantioenriched axially chiral 2,3-allenoic acids and their derivatives. *J. Org. Chem.* **79**, 3103-3110 (2014)
13. Cismesia, M. A. & Yoon, T. P. Characterizing chain processes in visible light photoredox catalysis. *Chem. Sci.* **6**, 5426-5434 (2015).
14. Strieth-Kalthoff, F., James, M. J., Teders, M., Pitzer, L. & Glorius, F. Energy transfer catalysis mediated by visible light: Principles, applications, directions. *Chem. Soc. Rev.* **47**, 7190-7202 (2018).
15. Zhou, Q. Q., Zou, Y. Q., Lu, L. Q. & Xiao, W. J. Visible-light-induced organic photochemical reactions through energy-transfer pathways. *Angew. Chem. Int. Ed.* **58**, 1586-1604 (2019).
16. Romero, N. A. & Nicewicz, D. A. Organic photoredox catalysis. *Chem. Rev.* **116**, 10075-10166 (2016).
17. Gaussian 09, Revision D.01, Frisch, M. J.; Trucks, G. W.; Schlegel, H. B.; Scuseria, G. E.; Robb, M. A.; Cheeseman, J. R.; Scalmani, G.; Barone, V.; Mennucci, B.; Petersson, G. A.; Nakatsuji, H.; Caricato, M.; Li, X.; Hratchian, H. P.; Izmaylov, A. F.; Bloino, J.; Zheng, G.; Sonnenberg, J. L.; Hada, M.; Ehara, M.; Toyota, K.; Fukuda, R.; Hasegawa, J.; Ishida, M.; Nakajima, T.; Honda, Y.; Kitao, O.; Nakai, H.; Vreven, T.; Montgomery, J. A., Jr.; Peralta, J. E.; Ogliaro, F.; Bearpark, M.; Heyd, J. J.; Brothers, E.; Kudin, K. N.; Staroverov, V. N.; Kobayashi, R.; Normand, J.; Raghavachari, K.; Rendell, A.; Burant, J. C.; Iyengar, S. S.; Tomasi, J.; Cossi, M.; Rega, N.; Millam, J. M.; Klene, M.; Knox, J. E.; Cross, J. B.; Bakken, V.; Adamo, C.; Jaramillo, J.; Gomperts, R.; Stratmann, R. E.; Yazyev, O.; Austin, A. J.; Cammi, R.; Pomelli, C.; Ochterski, J. W.; Martin, R. L.; Morokuma, K.;

Zakrzewski, V. G.; Voth, G. A.; Salvador, P.; Dannenberg, J. J.; Dapprich, S.; Daniels, A. D.; Farkas, Ö.; Foresman, J. B.; Ortiz, J. V.; Cioslowski, J.; Fox, D. J. Gaussian, Inc., Wallingford CT, 2009.

18. Hariharan, P. C. & Pople, J. A. The influence of polarization functions on molecular orbital hydrogenation energies. *Theor. Chim. Acta* **28**, 213-222 (1973).
19. Grimme, S.; Antony, J.; Ehrlich, S. & Krieg, H. A consistent and accurate ab initio parametrization of density functional dispersion correction (DFT-D) for the 94 elements H-Pu. *J. Chem. Phys.* **132**, 154104-154112 (2010).
20. Grimme, S.; Ehrlich, S. & Goerigk, L. Effect of the damping function in dispersion corrected density functional theory. *J. Comput. Chem.* **32**, 1456-1465 (2011).
21. Hay, P. J.; & Wadt, W. R. *Ab initio effective* core potentials for molecular calculations. Potentials for the transition metal atoms Sc to Hg. *J. Chem. Phys.* **82**, 270-283 (1985).
22. Wadt, W. R. & Hay, P. J. *Ab initio effective* core potentials for molecular calculations. Potentials for main group elements Na to Bi. *J. Chem. Phys.* **82**, 284-298 (1985).
23. Hay, P. J. & Wadt, W. R. *Ab initio effective* core potentials for molecular calculations. Potentials for K to Au including the outermost core orbitals. *J. Chem. Phys.* **82**, 299-303 (1985).
24. Fukui, K. The path of chemical reactions - the IRC approach. *Acc. Chem. Res.* **14**, 363-368 (1981).
25. Dunning, Jr. T. H. & Hay, P. J. "Gaussian basis sets for molecular calculations" in *Methods of electronic structure theory* (vol. 3 of *Modern Theoretical Chemistry*), H. F. Schaefer, Ed. 1-27. (Plenum Press, New York, 1977).
26. Fukui, K. Formulation of the reaction coordinate. *J. Phys. Chem.* **74**, 4161-4163 (1970).
27. Gonzalez, C. & Schlegel, H. B. An improved algorithm for reaction path following. *J. Chem. Phys.* **90**, 2154-2161 (1989).
28. Gonzalez, C. & Schlegel, H. B. Reaction path following in mass-weighted internal coordinates. *J. Phys. Chem.* **94**, 5523-5527 (1990).

29. Zhao, Y. & Truhlar, D. G. Density functionals with broad applicability in chemistry. *Acc. Chem. Res.* **41**, 157-167 (2008).

30. Zhao, Y. & Truhlar, D. G. The M06 suite of density functionals for main group thermochemistry, thermochemical kinetics, noncovalent interactions, excited states, and transition elements: two new functionals and systematic testing of four M06-class functionals and 12 other functionals. *Theor. Chem. Acc.* **120**, 215-241 (2008).

31. Andrae, D.; Häußermann, U.; Dolg, M.; Stoll H. & Preuß, H. Energy-adjusted*ab initio* pseudopotentials for the second and third row transition elements. *Theor. Chim. Acta* **77**, 123-141 (1990).

32. Marenich, A. V.; Cramer, C. J. & Truhlar, D. G. Universal solvation model based on solute electron density and on a continuum model of the solvent defined by the bulk dielectric constant and atomic surface tensions. *J. Phys. Chem. B* **113**, 6378-6396 (2009).
