# Supplementary material for: Photo and copper dual catalysis for allene syntheses from propargylic derivatives via one-electron process
Source: Nat Commun. 2022 Jun 8;13:3302. doi: 10.1038/s41467-022-30655-3 (PMC9177964; doi:10.1038/s41467-022-30655-3)
Supplement: Supplementary file 2 — Description of Additional Supplementary Files [file 41467_2022_30655_MOESM2_ESM.docx]

**Description of Additional Supplementary Files**

File Name: Supplementary Data 1

Description: Cartesian coordinates for the optimized structures
