# Supplementary material for: Photo and copper dual catalysis for allene syntheses from propargylic derivatives via one-electron process
Source: Nat Commun. 2022 Jun 8;13:3302. doi: 10.1038/s41467-022-30655-3 (PMC9177964; doi:10.1038/s41467-022-30655-3)
Supplement: Supplementary file 3 — Supplementary Dataset 1 [file 41467_2022_30655_MOESM3_ESM.pdf]

Cartesian coordinates for the optimized structures

Int1

C 1.47388000 -0.00084900 0.16140200

C 2.68199500 -0.00228500 -0.06528600

C 0.12100800 -0.00047400 0.40944800

C -0.67008600 -1.27770800 0.53135800

C -0.66915300 1.27726400 0.53187800

C -1.88210300 -1.26748800 -0.42245200

H -1.04594900 -1.37326800 1.56374500

H -0.03213700 -2.14521000 0.34210700

C -1.88121500 1.26838200 -0.42191100

H -1.04495900 1.37267900 1.56431100

H -0.03053300 2.14435200 0.34299200

C -2.72541500 0.00070900 -0.23697300

H -2.49357400 -2.16215200 -0.25667400

H -1.51970500 -1.31387100 -1.45748300

H -2.49201800 2.16340300 -0.25561100

H -1.51884200 1.31503700 -1.45693900

H -3.56700800 0.00116100 -0.93930000

H -3.15987000 0.00066500 0.77305700

C 4.11407300 0.00081300 -0.32569000

H 4.68670600 0.27841700 0.56892600

H 4.46316100 -0.98777000 -0.64527900

H 4.37682800 0.71637500 -1.11449500

LCu^{II}(CN)2

Cu -0.00006300 1.36082200 0.00001600

C -1.48661400 2.64154600 0.00076400

N -2.46331500 3.28301600 0.00133800

C 0.74085500 -1.50600100 0.00014300

C 2.65155200 -0.17301400 -0.00087600

C 1.51519700 -2.66766200 0.00148000

C -0.74077600 -1.50605100 -0.00013800

C 3.48451800 -1.29051400 0.00015200

H 3.03549700 0.84224700 -0.00169000

C 2.90412500 -2.55556600 0.00146000

H 1.04870700 -3.64472200 0.00270300

C -1.51504300 -2.66776000 -0.00138900

H 4.56036700 -1.15970700 0.00002400

H 3.52102000 -3.44839200 0.00249500

C -2.65155300 -0.17317800 0.00074100

C -2.90397600 -2.55575100 -0.00140200

H -1.04849900 -3.64479600 -0.00252800

C -3.48444400 -1.29073700 -0.00022700

H -3.03558200 0.84204600 0.00146800

H -3.52081200 -3.44862000 -0.00238000

H -4.56030200 -1.16000400 -0.00015700

N 1.31696500 -0.28460000 -0.00085600

N -1.31695100 -0.28467800 0.00075800

C 1.48647400 2.64156900 -0.00073500

N 2.46323400 3.28294900 -0.00127400

TS1_a

C -0.96981800 -3.12612600 -1.34658200

H -0.60671000 -3.36585000 -2.35322500

H -2.05182400 -2.96687100 -1.39560600

H -0.79692500 -3.97264100 -0.67753000

H 5.07203600 0.83280500 -2.66219900

H 4.33455400 1.72818200 -1.33871100

H 2.82664100 0.15865400 -3.49893400

C 0.70068500 -1.13675700 -0.95433300

C -0.26855900 -1.92954400 -0.83716700

C 1.70120000 -0.24026300 -0.97501900

C 1.67983400 0.98226300 -1.85865700

C 2.97028600 -0.45074800 -0.18811400

C 2.91193800 0.99013200 -2.78805200

H 1.72540800 1.87825300 -1.22182600

H 0.74970700 1.03271000 -2.42994300

C 4.20095000 -0.41833900 -1.11788400

H 3.05790900 0.36960400 0.54060300

H 2.91965600 -1.38438300 0.37671600

C 4.21108200 0.84717300 -1.98469100

H 2.92179800 1.91448600 -3.37664500

H 5.11641800 -0.48308600 -0.51971500

H 4.17864000 -1.30417900 -1.76487800

C -1.12966500 1.70450100 -0.07451000

C -2.79849200 0.70286500 -1.35707800

C -1.27129700 2.89944100 -0.78761300

C -0.92264700 -2.59611600 1.64971100

Cu -1.65433100 -1.07219700 0.73951600

N -0.39959300 -3.39937800 2.31780100

C -0.17119600 1.53636100 1.04677800

C -2.97481100 1.85258300 -2.12038700

H -3.41165100 -0.18060600 -1.49439300

C -2.19365500 2.96902400 -1.82708100

H -0.68142300 3.76866000 -0.52529000

C 0.79134100 2.49129800 1.39298500

H -3.71755200 1.87040600 -2.90937300

H -2.31200800 3.88982300 -2.38903100

C 0.55113100 0.11143900 2.73185900

C 1.64671100 2.22418100 2.46016500

H 0.88498500 3.41800700 0.83987200

C 1.52957300 1.01577600 3.14523000

H 0.42219100 -0.86084400 3.19890700

H 2.39971100 2.95136200 2.74706100

H 2.18145800 0.77188200 3.97643600

N -1.88672200 0.62960200 -0.38089300

N -0.27702900 0.37869800 1.72061600

C -3.61178400 -1.20840700 1.05769800

N -4.77990300 -1.23619000 1.01563200

Int2_a

Cu 1.39267400 1.24536500 0.17382900

C 0.65832700 2.92698000 0.56904100

N 0.21821300 3.94162200 0.94238500

C -1.17279500 0.98925100 -0.93107800

C -0.06050900 1.57485200 -1.19243000

C -2.28341800 0.39127700 -0.54069500

C -2.52928100 -1.08814300 -0.73013200

C -3.42096000 1.15805200 0.09652200

C -3.85506800 -1.32648500 -1.47888500

H -2.59587400 -1.55697600 0.26250000

H -1.68960200 -1.55345200 -1.25361000

C -4.74295900 0.90095000 -0.65076000

H -3.52700600 0.80191300 1.13339100

H -3.18438700 2.22355300 0.14216000

C -5.01989700 -0.60171900 -0.79147700

H -4.05568700 -2.40213500 -1.54329200

H -3.75280600 -0.95770400 -2.50755900

H -5.56747600 1.39613300 -0.12617900

H -4.68027100 1.35499900 -1.64798800

H -5.94736600 -0.76440000 -1.35188700

H -5.17586500 -1.03564000 0.20650100

C 1.45094400 -1.67650600 -0.20326500

C 2.80953400 -0.54709000 -1.72031500

C 1.85537100 -2.90540700 -0.73598100

C 0.51205100 -1.56432900 0.94463300

C 3.25111700 -1.73233200 -2.29847300

H 3.17841300 0.41619600 -2.05524900

C 2.75781800 -2.93361800 -1.79420700

H 1.48087400 -3.83234000 -0.32094900

C -0.11145600 -2.66669200 1.54220300

H 3.96711000 -1.70694800 -3.11157000

H 3.07936500 -3.88262200 -2.21059700

C -0.54346600 -0.10370000 2.40240600

C -0.97843500 -2.44766500 2.61018900

H 0.06340200 -3.67465800 1.18681000

C -1.20625800 -1.14486000 3.05226300

H -0.67545200 0.93477500 2.69337100

H -1.47176500 -3.28730200 3.08927800

H -1.87470500 -0.93788800 3.88046300

N 1.92695800 -0.52151600 -0.71604800

N 0.28779400 -0.31834300 1.38447000

C 3.02594300 1.26755300 1.26174800

N 4.02320500 1.18060900 1.86352300

C 0.45319700 2.32899500 -2.37551900

H 0.52371100 3.39403900 -2.13885800

H 1.45013600 1.99042500 -2.67751100

H -0.22427400 2.19165900 -3.22529900

TS2_a

Cu 1.39316300 1.24791000 0.18142300

C 0.59628400 2.90505200 0.56378600

N 0.14704700 3.90496100 0.96646100

C -1.18691500 1.02003800 -0.90745400

C -0.08158600 1.62694900 -1.15756500

C -2.29051400 0.40439200 -0.52831700

C -2.50486300 -1.08128700 -0.70924300

C -3.45163600 1.15395600 0.08701700

C -3.81569400 -1.35156100 -1.47305900

H -2.57455300 -1.54325900 0.28630100

H -1.64968000 -1.53381800 -1.21839900

C -4.75784000 0.86467900 -0.67587200

H -3.56418900 0.80412200 1.12526400

H -3.23676500 2.22425900 0.12712500

C -5.00355700 -0.64424500 -0.80732800

H -3.99425700 -2.43145500 -1.53082700

H -3.70707600 -0.98955400 -2.50350100

H -5.59887000 1.34800800 -0.16670700

H -4.69042800 1.31155300 -1.67603900

H -5.92008100 -0.82969300 -1.37853800

H -5.16437900 -1.07278400 0.19218400

C 1.45685800 -1.67991500 -0.20988800

C 2.79375100 -0.54600800 -1.74124900

C 1.86209600 -2.90786800 -0.74439400

C 0.52700800 -1.57088500 0.94605000

C 3.23582000 -1.72975900 -2.32257800

H 3.15455000 0.41867900 -2.08132100

C 2.75422600 -2.93288300 -1.81151500

H 1.49653700 -3.83628400 -0.32465100

C -0.08485000 -2.67598300 1.55070800

H 3.94325200 -1.70197300 -3.14309900

H 3.07666100 -3.88081700 -2.22970500

C -0.52843100 -0.11446200 2.40846100

C -0.94602600 -2.46072800 2.62407700

H 0.09467100 -3.68352300 1.19645000

C -1.17999000 -1.15854300 3.06480700

H -0.66541900 0.92370600 2.69835600

H -1.43016300 -3.30274800 3.10835700

H -1.84435300 -0.95424100 3.89695300

N 1.92159200 -0.52283900 -0.72811200

N 0.29759900 -0.32533300 1.38522500

C 3.04705800 1.30021900 1.23323000

N 4.05965200 1.23244400 1.81160200

C 0.42864300 2.37814500 -2.34513100

H 1.43801500 2.06144700 -2.62851800

H -0.23419400 2.21051100 -3.20075100

H 0.46870300 3.44801600 -2.12371000

2B

C 3.14744800 -0.60060400 0.00086800

N 4.00690200 -1.38551400 0.00213200

C 0.85185900 0.01346900 -0.00020100

C 2.11367600 0.39828600 -0.00065100

C -0.39470400 -0.37276700 0.00036800

C -1.18417500 -0.58053000 -1.27937300

C -1.18380500 -0.57666600 1.28097000

C -2.45652200 0.28515700 -1.26851400

H -1.47154300 -1.64009600 -1.33706900

H -0.56508000 -0.36297000 -2.15327500

C -2.45608100 0.28915200 1.26783300

H -1.47136500 -1.63595300 1.34202000

H -0.56450700 -0.35651200 2.15400600

C -3.28583600 0.04684500 0.00016800

H -3.05330400 0.07286300 -2.16247000

H -2.16693400 1.34258300 -1.32147400

H -3.05256500 0.07970500 2.16265700

H -2.16638900 1.34672400 1.31731500

H -4.17062000 0.69298100 -0.00074800

H -3.65355500 -0.98880200 0.00185700

C 2.55874300 1.84907100 -0.00276800

H 3.16802000 2.06721500 0.88027500

H 3.16740400 2.06482900 -0.88684200

H 1.68851500 2.50755100 -0.00337500

LCu^{I}CN

Cu 1.57390500 -0.21534700 0.00017800

C -1.35898700 -0.59482200 0.00003700

C -0.26726500 -2.64443600 0.00043500

C -2.60529800 -1.22811900 -0.00028000

C -1.18927500 0.88334000 -0.00005800

C -1.47072600 -3.34685900 -0.00001900

H 0.69001300 -3.15582400 0.00092600

C -2.65910400 -2.61989500 -0.00035700

H -3.52178400 -0.65111900 -0.00045600

C -2.25816900 1.78455500 -0.00027800

H -1.46918300 -4.43088600 -0.00008600

H -3.61837900 -3.12737900 -0.00066700

C 0.34424900 2.62537800 0.00022700

C -1.98954700 3.15158500 -0.00018400

H -3.28309500 1.43439200 -0.00052500

C -0.66588800 3.58611700 0.00011100

H 1.39425300 2.90126000 0.00045400

H -2.80637700 3.86614500 -0.00031100

H -0.41606500 4.64103600 0.00023100

N -0.21324100 -1.30749900 0.00043400

N 0.08775700 1.31362500 0.00019700

C 3.44663200 -0.33866000 -0.00013300

N 4.61514600 -0.40350600 -0.00087900

TS_a

Cu -1.04894900 0.98193500 0.70553800

C -2.43768300 -0.32527700 1.27451600

N -3.03760900 -0.86918700 2.15270300

C -2.30245700 2.34387400 0.05561300

N -3.00566800 3.13429500 -0.44071200

C -1.91243500 -1.37923700 -1.13492300

C -2.91094300 -0.89172300 -0.51179100

N 0.60016500 1.89708900 -0.13370400

C 1.80171200 1.43538900 0.28997300

C 0.53754600 2.92281100 -0.99470700

C 2.99039000 2.02674100 -0.15143400

C 1.68217400 3.53707800 -1.49148200

H -0.46265300 3.24508000 -1.26473200

C 2.92820500 3.08251800 -1.05379600

H 3.94747500 1.67145300 0.21015000

H 1.59265000 4.35753100 -2.19370700

H 3.84166800 3.55038900 -1.40641500

N 0.50161700 -0.05731800 1.62580300

C 1.74307400 0.27762100 1.19613600

C 0.33838200 -1.11115500 2.43961800

C 2.86114200 -0.47977200 1.56463000

C 1.41089700 -1.88501200 2.86984600

H -0.68534800 -1.32786400 2.72419200

C 2.69276100 -1.56766800 2.41282100

H 3.84262300 -0.23365400 1.17837400

H 1.23824200 -2.72252900 3.53547000

H 3.54883100 -2.16598000 2.70723000

C -4.34023900 -0.55845400 -0.71502500

H -4.47883500 0.52566100 -0.64330100

H -4.68103800 -0.89447400 -1.69994000

H -4.95137200 -1.02629100 0.06205100

C -0.78389100 -1.88193200 -1.64080000

C -0.40716900 -3.33557200 -1.45520000

C 0.15745800 -1.06941100 -2.50467000

C 1.05667700 -3.46910100 -0.99564800

H -0.51486900 -3.86324500 -2.41668500

H -1.08814200 -3.81398600 -0.74658800

C 1.62568300 -1.25735500 -2.08183500

H 0.05317600 -1.40612000 -3.54892700

H -0.12210700 -0.01226000 -2.48153600

C 2.00083000 -2.73872900 -1.95855100

H 1.32852600 -4.52853700 -0.92163000

H 1.15823100 -3.04064600 0.01035600

H 2.28606300 -0.74765200 -2.79364400

H 1.77842800 -0.77959100 -1.11029400

H 3.03983200 -2.83659200 -1.62154400

H 1.94591400 -3.21477300 -2.94785600

TS1_b

Cu -0.59385300 0.22153800 0.89722400

C -1.52896500 -1.29544500 1.49376700

N -1.81661500 -2.27831400 2.05722300

C -0.35009700 -1.18183600 -1.64556200

C 0.68333600 -1.59981800 -2.13438900

C 2.01023300 1.15086500 -0.17896400

C 0.35618000 2.77717000 -0.34169200

C 2.97033300 2.05702500 -0.64344000

C 2.35792000 -0.22048200 0.26922000

C 1.25407100 3.72501600 -0.81856500

H -0.68040800 3.02686500 -0.14623700

C 2.58672000 3.35086500 -0.97972400

H 4.01065500 1.76409500 -0.71067200

C 3.59643100 -0.82185000 0.01757700

H 0.91482400 4.73096400 -1.03643100

H 3.32343900 4.06340100 -1.33637400

C 1.61357800 -2.09014900 1.42068400

C 3.82935300 -2.10504800 0.50779800

H 4.35801200 -0.31344200 -0.56149000

C 2.82391800 -2.75584100 1.22106800

H 0.77971700 -2.54526700 1.94803800

H 4.78282800 -2.59139200 0.32667600

H 2.96734400 -3.75583400 1.61495600

N 0.71502000 1.51626600 -0.06279400

N 1.40081900 -0.85471100 0.96589800

C -0.96584100 1.58953500 2.29960000

N -1.22540000 2.53715100 2.93454200

C -1.58062100 -0.72884300 -1.13612800

C -2.06317000 0.64068200 -1.58518400

C -2.69688300 -1.75974700 -1.05841700

C -3.29297900 1.13493200 -0.81162000

H -2.34966100 0.50403100 -2.64294900

H -1.25109500 1.36504100 -1.59309400

C -3.98931800 -1.26194900 -0.39710800

H -2.90686500 -2.00104900 -2.11552000

H -2.33892500 -2.67369300 -0.58575300

C -4.43044600 0.11225900 -0.91325400

H -3.60601300 2.10219500 -1.21993300

H -3.02771500 1.29988000 0.23922600

H -4.77605800 -2.00420300 -0.57058900

H -3.82931800 -1.21510900 0.68209500

H -5.30029500 0.45914400 -0.34573300

H -4.74820400 0.03445200 -1.96309700

C 1.91983300 -2.11073100 -2.70304900

H 1.72580200 -2.68515800 -3.61631500

H 2.61040000 -1.29852200 -2.95400500

H 2.42498600 -2.77340100 -1.99209200

Int2_b

Cu -0.65047700 0.09878000 0.84494300

C -1.48621300 -1.37990600 1.62849100

N -1.75451100 -2.36677200 2.19187300

C -0.37289900 -1.17289600 -1.66752100

C 0.61927900 -1.62100700 -2.20878300

C 1.82999500 1.25812000 -0.21397200

C 0.05557800 2.76563000 -0.23318700

C 2.70358500 2.26707100 -0.63590000

C 2.30502400 -0.10667500 0.12945500

C 0.86814200 3.81043900 -0.65300600

H -0.99659800 2.92145600 -0.02721700

C 2.22014500 3.54945300 -0.86651000

H 3.75896300 2.05667900 -0.75249400

C 3.58792500 -0.57599700 -0.17599700

H 0.44923900 4.80013600 -0.79151500

H 2.89376500 4.33791600 -1.18579100

C 1.74978900 -2.10263100 1.16775700

C 3.94011900 -1.86272500 0.22751200

H 4.29600400 0.03360200 -0.72436200

C 3.01144400 -2.64406200 0.91336200

H 0.97052200 -2.65677600 1.68464300

H 4.92921400 -2.24968200 0.00339200

H 3.25277200 -3.64831700 1.24357200

N 0.51388200 1.51944800 -0.04549600

N 1.42132200 -0.87040900 0.78599200

C -0.43089200 1.11194300 2.54845800

N -0.27070400 1.81282300 3.46983200

C -1.54941200 -0.66224700 -1.06609500

C -2.05245700 0.66271700 -1.63962900

C -2.69637200 -1.66816300 -0.93563200

C -3.22455000 1.24136800 -0.83739200

H -2.41288000 0.42747000 -2.65474300

H -1.24002100 1.37426700 -1.77379200

C -3.94693400 -1.11101400 -0.24260500

H -2.95421000 -1.93343700 -1.97485400

H -2.35424100 -2.57842300 -0.44534200

C -4.38897700 0.24340500 -0.80974700

H -3.54055600 2.19227700 -1.28133500

H -2.90513300 1.45570100 0.19222300

H -4.75643200 -1.84291800 -0.33806300

H -3.74072600 -1.01098900 0.82589700

H -5.21818600 0.64394900 -0.21678300

H -4.76546600 0.11577600 -1.83454500

C 1.82167300 -2.17025800 -2.81730500

H 1.59063700 -2.69413900 -3.75197100

H 2.54988500 -1.38380100 -3.04355000

H 2.30339500 -2.88637700 -2.14263500

TS2_b

Cu -0.60505800 0.28507100 0.89927400

C -1.80484500 -1.04081300 1.46125800

N -2.28198000 -1.95939400 2.00289200

C -0.33729300 -0.62485000 -1.76901800

C 0.68223800 -0.88621000 -2.37921400

C 2.15625800 0.80897700 -0.04869100

C 0.82296500 2.71074000 -0.14778700

C 3.27222500 1.55168900 -0.45045200

C 2.24737900 -0.62801500 0.31337500

C 1.88291700 3.50420600 -0.57247200

H -0.15447000 3.13515900 0.05172900

C 3.13133600 2.90684900 -0.73131200

H 4.24645600 1.08288900 -0.51026100

C 3.36266500 -1.42403800 0.02533400

H 1.73076100 4.56234500 -0.75019000

H 3.99059200 3.49213900 -1.04247400

C 1.16705400 -2.40479500 1.33759700

C 3.35352100 -2.75834400 0.42556300

H 4.21197800 -1.02409900 -0.51540500

C 2.24013900 -3.26294700 1.09555400

H 0.26001000 -2.73529400 1.83663900

H 4.20576700 -3.39573700 0.21155300

H 2.19750000 -4.29608000 1.42181600

N 0.94510400 1.39598600 0.07125400

N 1.18241600 -1.12582400 0.96055200

C -0.67903000 1.54554400 2.42689900

N -0.70633300 2.42302900 3.19904500

C -1.57139600 -0.35292200 -1.13513600

C -2.14504700 1.04605600 -1.32910600

C -2.59070800 -1.48183500 -1.28393700

C -3.49040100 1.26147100 -0.63097000

H -2.28756300 1.15525700 -2.41815100

H -1.42006500 1.81583100 -1.05826500

C -3.99958800 -1.20448600 -0.74196800

H -2.65551000 -1.63615000 -2.37424100

H -2.18439300 -2.40341700 -0.86381200

C -4.50110400 0.20257900 -1.08174600

H -3.85175600 2.26906500 -0.86293200

H -3.35078300 1.21001700 0.45462800

H -4.67725100 -1.96085900 -1.15378700

H -3.99623900 -1.33668800 0.34044900

H -5.47058600 0.37575000 -0.60286600

H -4.66267600 0.29806100 -2.16550100

C 1.91590000 -1.17625800 -3.09326800

H 1.72437500 -1.36811900 -4.15530500

H 2.61777000 -0.33698100 -3.02498700

H 2.40722200 -2.06230800 -2.67681900

3B

C -0.11896900 1.59590600 0.00000100

N -0.35774700 2.73176700 -0.00001000

C 1.58831700 -0.08898000 0.00001000

C 2.77614600 -0.30888800 0.00000500

C 0.13795400 0.13834900 0.00001000

C -0.51351200 -0.48609800 -1.27274600

C -0.51352300 -0.48609200 1.27275400

C -2.03869400 -0.34687700 -1.26645800

H -0.22677700 -1.54375500 -1.28135500

H -0.07477400 -0.02277000 -2.16064300

C -2.03870600 -0.34687000 1.26644900

H -0.22679200 -1.54375000 1.28137000

H -0.07479800 -0.02276500 2.16065800

C -2.65431100 -0.95523200 -0.00000500

H -2.44734700 -0.82929200 -2.16092300

H -2.30517600 0.71522600 -1.33157000

H -2.44736900 -0.82927700 2.16091400

H -2.30518600 0.71523500 1.33155100

H -3.73975200 -0.80892300 -0.00001100

H -2.48190800 -2.04056300 -0.00000100

C 4.21334100 -0.55637400 -0.00000400

H 4.51692200 -1.12670400 0.88438700

H 4.51738300 -1.12398200 -0.88598800

H 4.77154400 0.38588700 0.00158800

TS_b

Cu 0.16291900 -0.41656600 -0.90736200

C -1.70728300 0.38165800 -0.91184400

N -2.35788100 1.02904600 -1.67560400

C -0.40246100 -2.18988500 -1.49748200

N -0.69884200 -3.29658500 -1.72739500

C -0.11827800 -0.44444900 2.11483400

C -1.20483400 -0.31201900 1.58379900

C -2.41490500 -0.11590600 0.85413300

C -3.21107700 -1.38487000 0.56221000

C -3.24243100 1.07790000 1.32309200

C -4.48798900 -1.12084000 -0.24136300

H -3.48105000 -1.81767500 1.53890000

H -2.57082300 -2.11433100 0.06024300

C -4.53699600 1.28681200 0.52902800

H -3.49438800 0.88639800 2.37830800

H -2.62146300 1.97951300 1.31298700

C -5.33690600 -0.01496300 0.39775000

H -5.06054700 -2.05185000 -0.31508500

H -4.21471200 -0.82879400 -1.26040800

H -5.13685600 2.05962800 1.02384700

H -4.28309200 1.65899900 -0.46866300

H -6.24083300 0.16018800 -0.19646000

H -5.67264000 -0.34176200 1.39308100

C 1.14119900 -0.60724000 2.82868500

H 1.61955200 -1.56009600 2.57897600

H 1.84449600 0.19435200 2.57409800

H 0.98644400 -0.57981900 3.91316600

N 2.02021200 -1.02552100 -0.31720700

C 2.88560300 -0.01949900 -0.02066500

C 2.40142200 -2.30670900 -0.18289700

C 4.18618300 -0.30828400 0.41888900

C 3.66619900 -2.65375100 0.27031000

H 1.65596800 -3.04559000 -0.45677900

C 4.57682000 -1.63046700 0.56958100

H 4.88140400 0.49486900 0.63126900

H 3.93463500 -3.69860000 0.37307900

H 5.58018600 -1.86657200 0.90867300

N 1.09942800 1.39585500 -0.72634900

C 2.34430000 1.32999900 -0.17602100

C 0.52648600 2.59487900 -0.93031900

C 3.01689400 2.49703800 0.21586200

C 1.15786700 3.78511000 -0.60026300

H -0.46886900 2.56941800 -1.35847900

C 2.42461200 3.73168600 -0.00279800

H 3.98949000 2.43359900 0.68887000

H 0.66461500 4.72957700 -0.79777600

H 2.93685900 4.64220200 0.29040600
